# Unraveling condition specific gene transcriptional regulatory networks in *Saccharomyces cerevisiae*

**DOI:** 10.1186/1471-2105-7-165

**Published:** 2006-03-21

**Authors:** Hyunsoo Kim, William Hu, Yuval Kluger

**Affiliations:** 1 Department of Cell Biology, NYU School of Medicine, Skirball Institute of Biomolecular Medicine, 540 First Avenue, New York, NY 10016, USA

## Abstract

**Background:**

Gene expression and transcription factor (TF) binding data have been used to reveal gene transcriptional regulatory networks. Existing knowledge of gene regulation can be presented using gene connectivity networks. However, these composite connectivity networks do not specify the range of biological conditions of the activity of each link in the network.

**Results:**

We present a novel method that utilizes the expression and binding patterns of the neighboring nodes of each link in existing experimentally-based, literature-derived gene transcriptional regulatory networks and extend them *in silico *using TF-gene binding motifs and a compendium of large expression data from *Saccharomyces cerevisiae*. Using this method, we predict several hundreds of new transcriptional regulatory TF-gene links, along with experimental conditions in which known and predicted links become active. This approach unravels new links in the yeast gene transcriptional regulatory network by utilizing the known transcriptional regulatory interactions, and is particularly useful for breaking down the composite transcriptional regulatory network to condition specific networks.

**Conclusion:**

Our methods can facilitate future binding experiments, as they can considerably help focus on the TFs that must be surveyed to understand gene regulation.

(Supplemental material and the latest version of the MATLAB implementation of the United Signature Algorithm is available online at [1] or [see Additional files 1, 2, 3, 4, 5, 6, 7, 8, 9, 10])

## Background

Transcriptional regulation is fundamental to translating genetic information into biological function, and is thus critical for understanding cell adaptation, differentiation, and pathological transformation. One challenge is to decipher the intricate network of transcriptional interactions, so as to better appreciate functional relationships and to discern disease states.

Using published high-throughput mRNA expression data, several groups have proposed algorithms to computationally construct transcriptional regulatory networks for *S. cerevisiae *[2]. One approach, as presented by Segal *et al. *[3], is to use a probabilistic expression model, in which regulatory relationships can be deduced by the correlation of co-expression between a DNA-binding transcription regulator and its target gene^3^. Other approaches, such as those recently published by Rice *et al. *[4], use gene perturbation to infer the direction of regulatory effects^4^. However, there are limitations to the reverse-engineering approach to deriving transcriptional regulatory networks from expression data [5]. Correlation matching does not distinguish regulators from target genes. It is difficult to discern whether the correlated target is directly or indirectly regulated. Additional information, such as protein-DNA binding, can be integrated into transcriptional regulatory networks, as described by Bar-Joseph *et al *[5], for validating direct regulator-target interaction.

Recently, the genomic binding sites of 203 yeast transcriptional regulators were identified using ChIP-on-chip experiments under a limited number of growth conditions [6]. The results showed that regulator binding is highly dependent upon the environmental conditions of the cell. These experiments provide information on condition-specific TF binding events, which may be associated with activation or inhibition of the target genes. However, binding between a TF and a target gene in a given condition is not sufficient to predict whether a target gene will be expressed in this condition [7]. Therefore it would be highly advantageous to develop a computational approach for predicting the biological conditions under which each regulator-target gene link in the known regulatory network is active.

**Figure 1:**
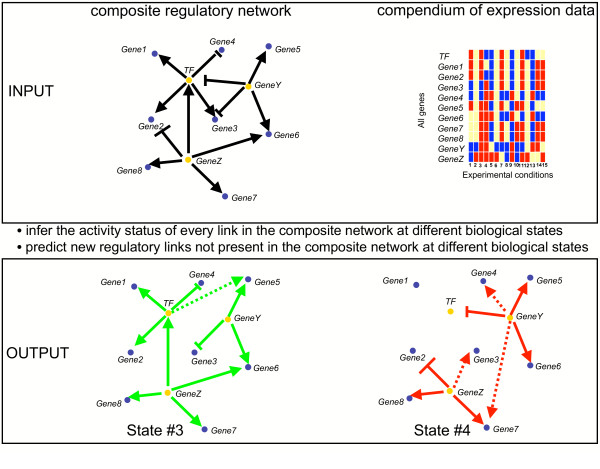
Break-down (decomposition) of the composite regulatory network. The input for our algorithm (upper panel) includes: a) a composite regulatory network published by Milo *et al *[22] (or a joint network obtained by integrating the literature driven gene regulatory networks compiled by Milo *et al. *[22] and Herrgard *et al *[21]). The edges (black links) and nodes of the composite network are illustrated by a graph with 11 genes, where yellow and blue circles represent TFs and non regulating genes respectively (b) microarray gene expression profiles from 387 different experimental conditions involving diploid cells. This is a subset of the experiments stored in a compendium of *S. cerevisiae *gene expression datasets compiled by Ihmels *et al *[9]. The expression dataset is illustrated by a miniature matrix consisting of 11 genes and 15 experimental conditions, whose red, blue and yellow entries correspond to up-regulation, down-regulation and intermediate expression levels, respectively. The output of our algorithm allows us to read out condition specific regulatory networks, as illustrated in the lower panel.

In this paper, we present an approach based on a variant of the signature algorithm [8-10] to extend known transcriptional regulatory networks. Our computational approach, which integrates binding and expression information, enables us not only to identify new condition-specific transcriptional regulatory interactions, but also to predict the conditions in which each link in the known regulatory network is active (as schematized in Fig. 1).

## Results

### The United Signature Algorithm

**Figure 2:**
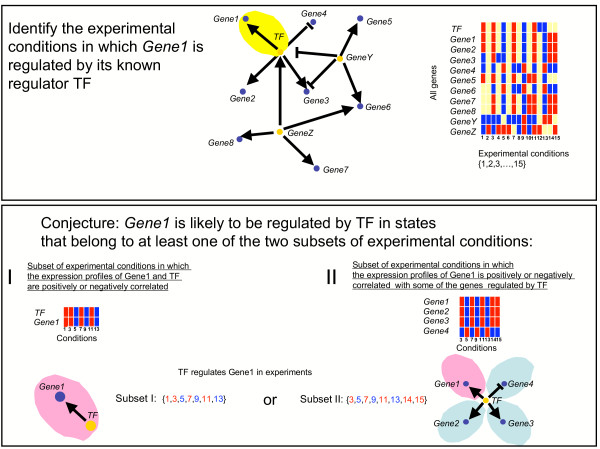
Local network approach for identifying the experimental conditions for gene regulation by its known direct regulator. As in Fig. 1, the input includes the composite network and a gene expression compendium dataset. For each link in the network, as illustrated for the link TF→Gene1 highlighted in yellow, we identify conditions in which the target gene (Gene1) is directly controlled by its regulator (TF), by extracting two types of condition subsets: a) subset of states in which the expression profile of the target gene (highlighted in pink) is positively or negatively correlated with the expression profile of its regulator, b) subset of states in which the expression profile of the target gene (highlighted in pink) is correlated with the expression profiles of other genes (highlighted in light blue) that are known to be regulated by the same TF. In the illustration shown in the lower panel the conditions in which the TF directly regulates the target gene are indicated below the gene expression patterns across these conditions. These conditions are also displayed in the braces shown next to the sub-networks. To differentiate between conditions in which the link is functional/not-functional due to the activation/deactivation of the TF, we mark these conditions in red and blue respectively.

**Figure 3:**
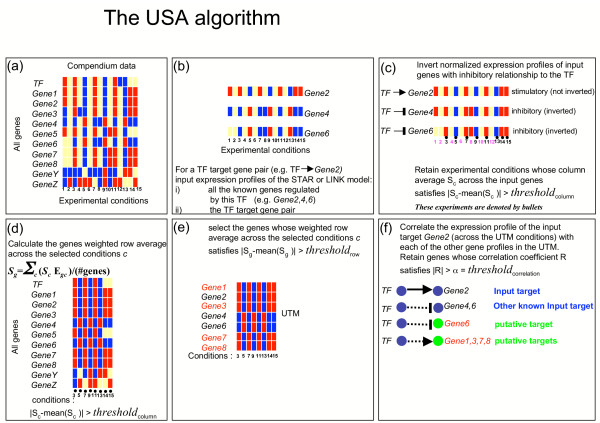
The united signature algorithm (USA). This algorithm is designed to find a subset of conditions in which the input genes are regulating each other or are co-regulated, and to identify additional genes that are potentially co-regulated under the same subset of conditions. The order of the procedures performed in the USA is shown in the following six panels: a) bi-normalization and log transformation of the raw expression data, such that row sums and column sums are equal to zero, b) selection of an input set of gene expression profiles consisting of the target gene and its TF regulator, or the expression profiles of the target gene and all the other known regulated genes controlled by the TF. c) calculation of condition (column) scores by summing (or averaging) the columns of a sub-matrix, whose rows represent the normalized expression profiles of the input genes across all conditions. These rows are first multiplied by +1 for input genes that are stimulated by the TF and by -1 (inversion) for target genes inhibited by the TF. Experimental conditions whose column average S_c _across the input genes satisfies |S_c_-mean(S_c_)| > *threshold*_column _are retained as indicated by black bullets and black experimental IDs below the sub-matrix, d) calculation of gene (row) scores defined as the weighted row average *S*_*g *_= Σ_*c *_(*S*_*c *_E_*gc*_)/(#genes) across the selected conditions *c *e) determination of a sub-matrix of genes and conditions, termed the united transcriptional module (UTM), consisting of gene expression profiles whose weighted row averages satisfy |S_g_-mean(S_g_)| > *threshold*_row _across the selected conditions *c*, f) retaining genes within the UTM whose correlation with the target gene *Gene2*, TF or the centroid gene (a gene that is correlated with the largest number of genes within the UTM) satisfy |R| > α = *threshold*_correlation _where R is the correlation coefficient.

In recent years several bi-clustering techniques have been developed to group genes into transcription modules with similar mRNA expression profiles under a selected range of experimental conditions [11-16]. A unique property of the signature algorithm (SA) of Ihmels *et al. *[8-10] is that it finds a bi-cluster associated with a group of input genes of interest by identifying a stable subset of conditions in which an output set of genes (containing all or part of the input set of genes and some additional genes) are co-expressed. One advantage of this algorithm is that its input does not include expression profiles of irrelevant genes that may introduce noise and obscure the output quality. We develop a variant of the SA called the united signature algorithm (USA), which identifies a united transcription module (UTM) consisting of both negatively and positively correlated genes. This enables us to unravel the conditions in which each known transcriptional regulatory TF-gene target link becomes functional or disabled due to a change in the TF activity (Fig. 2). Moreover, it predicts new condition-specific inhibitory as well as stimulatory transcriptional regulatory relationships between a TF and its putative target genes. The algorithm is described in detail in Methods section, and its steps are illustrated in Fig. 3.

### Predicting novel and condition-specific transcriptional regulatory interactions

**Figure 4:**
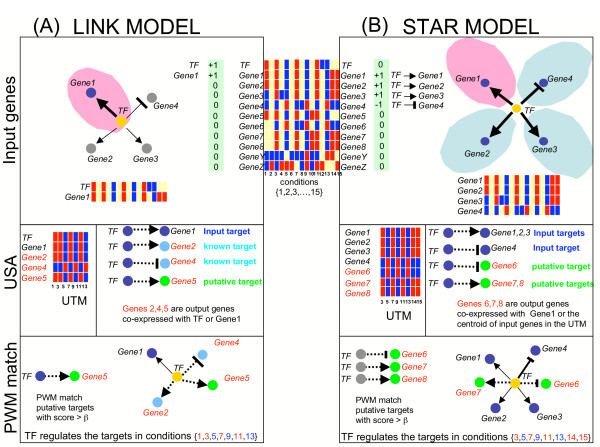
A schema of the LINK and STAR models: **(A) **the LINK model is designed to find a subset of conditions in which the expression profiles of a TF-target gene pair (link highlighted in pink in the upper left panel) are positively or negatively correlated. In addition it finds other known or putative target genes whose expression profiles are correlated with the TF or its target under this subset of conditions. The regulating TF and its target gene (*Gene*_1_) are the core set of input genes inserted into the USA. The blue and yellow nodes in the known local network represent the input gene set employed by the USA. This input set is also indicated in a genome-wide vector that has only two nonzero elements representing the TF (+1) and its target (+1 for activation and -1 for suppression). The dataset we insert into the USA is the yeast compendium data described in Fig. 1 and schematized in the miniature matrix consisting of 11 genes and 15 experimental conditions shown between the left and right panels. The red, blue and yellow entries correspond to up-regulation, down-regulation and intermediate expression levels respectively. The algorithm finds the conditions in which TF and *Gene1 *are correlated. It also finds the additional genes denoted in red (*Gene*_2_, *Gene*_4_, and *Gene*_5_), whose expression profiles across the subset of experimental conditions correlate positively or negatively with the TF or *Gene1*. Altogether, these genes and conditions constitute the UTM shown in the left middle panel. We predict that links of the known regulatory interactions *TF*→*Gene*_2 _and *TF*⊣*Gene*_4 _are functional under the united transcriptional module (UTM) conditions in which the TF, *Gene*_1 _and *Gene*_2 _are up regulated (red pixels in the UTM matrix) and *Gene*_4 _is down regulated (blue pixels). For the predicted link *TF*→*Gene*_5_, we further compute a MATCH score between the *TF *position-specific weight matrix (PWM) and the promoter region of *Gene*_5_. Links with a score higher than a threshold value of 0.94 (i.e. PWM match) are reported along with the experimental conditions supported by their corresponding UTM as illustrated in the lower left panel. **(B) **the STAR model enables us to find an alternative subset of experimental conditions in which *Gene1 *is directly regulated by TF (illustrated by the link highlighted in pink in the right upper panel). It searches for conditions in which the expression profiles of *Gene1 *is positively or negatively correlated with some of the genes regulated by TF (highlighted in light blue). In addition, it is designed to identify new genes that are positively or negatively correlated with the input core of target genes under the same subset of experimental conditions, and whose promoters contain sequences similar to the TF binding site. In the STAR model, we apply the USA (right center panel) to the core set of input genes consisting of all the TF target genes (*Gene*_1,2,3,4_) excluding the *TF *itself. As in the LINK model, the input set of genes is indicated by the nonzero elements of a genome-wide vector (highlighted in green in the upper right panel), which denotes the regulatory relationship between the *TF *and its target genes. In addition to links from the original local (STAR) network, two new links, i.e. *TF*⊣*Gene*_6 _and *TF*→*Gene*_7_, are predicted based on their co-expression with the core genes, and the match between their promoter region and the TF PWM. Each target is predicted to be affected by the TF under the experimental conditions of the respective UTM.

We integrated two complementary local network models for predicting: (a) new transcriptional regulatory interactions, (b) specific conditions in which experimentally known and predicted interactions take place. The first model (LINK model) is based on the assumption that there is a correlation between the mRNA levels of a TF and its target gene under certain conditions [17] (see Figs. 2, 3, 4). This assumption is not universally satisfied, since the activation or repression of a target gene by a TF depends on the TF protein concentration, post-translational modifications, localization, phosphorylation status and other cellular factors. Thus, we implement a second model (the STAR model) that is based on a different notion. In this model we disregard the mRNA expression state of the TF itself and deduce conditions of activity or inactivity of a TF from the expression patterns of its target genes [5] (see Figs. 2, 3, 4).

Applying the USA to these two input sets of genes surrounding a TF-target gene link provides us with the LINK and STAR UTMs consisting of experimental conditions under which the target gene is predicted to be regulated by the TF (see Fig. 4). These UTMs consist of additional genes, whose expression profiles are highly correlated with their corresponding input set of genes (under the conditions supported by the UTM). To identify potential new targets of this TF, we subject these additional putative target genes to another filter that selects genes whose promoter regions contain a sequence that matches with the binding site of this TF. There are different approaches to reduce possible false positives in the last step of matching TF binding sites with gene promoter regions. The most common approaches take into account conservation of these binding sites in other species, or test whether the TF binding sites are significantly over-represented in the target set with respect to the background set [18]. The latter is not always applicable to small input sets employed by the LINK and STAR models.

#### (a) LINK model

The first local network model that we use to generate a UTM, which we shall refer to as the "LINK model" (Fig. 4), is designed to find a subset of experimental conditions which maximizes the correlation (or inverse correlation, as in the case of an inhibitory interaction) between the expression profiles of the TF and its experimentally known target gene. Our input data include: **(i) **a compendium of gene expression profiles arranged in a matrix whose columns represent genome-wide profiles of different experimental conditions, **(ii) **a genome-wide input vector, whose two nonzero elements represent the TF (+1) and its known target gene (+1 for activation, -1 for suppression). As stated above, the genes in each UTM include the known and predicted target genes of the regulator associated with the module. To increase our confidence that the genes within the module found by the USA are regulated by the corresponding TF, we have used two additional parametric constraints to select candidate target genes: **(i) **the absolute value of the Pearson correlation coefficient (|*R*|) between the regulator expression profile and the profile of any candidate target gene across the experiments of the UTM is greater than the correlation threshold value α (Fig. 3f) **(ii) **the MATCH [19,20] score between the position-specific weight matrix (PWM) [6] of the TF binding motif and the target promoter sequence is greater than or equal to a predetermined cutoff β (Fig. 4). We explored the parameter space for α and β, and found that at values of about α = 0.5 for the LINK model and β = 0.94 we are able to optimize the prediction rate.

#### (b) STAR model

The STAR model is a second local network model that we use to generate a UTM (Fig. 4). The input required for this model involves the expression profiles of the target gene associated with the link of interest (see link highlighted by pink background in Fig. 4) and of all the other experimentally known target genes (see links highlighted by light blue background in Fig. 4) that are regulated by the same TF, which is positioned in the center of a star-like local network. The expression profile of the TF is excluded from the input. The input also includes a genome-wide vector, whose elements indicate the type of regulatory relationship between the TF and its target genes (+1 for activation, -1 for repression, zero otherwise). Since we exclude the TF from the input of the STAR model, the element corresponding to this TF in this (genome-wide input) vector is set to zero. We employ the USA to the input set of known target genes, and obtain an output UTM consisting of the majority of the input genes and an additional new set of genes whose regulatory relationships with the TF are yet unknown. The expression profiles of these new genes across the UTM experimental conditions correlate with the corresponding expression profiles of the input genes maintained in the UTM. This alludes to the possibility that these new genes are regulated by the TF associated with this UTM. We used an iterative scheme that involves repeated applications of the USA to eliminate weakly correlated genes (See the Methods section for detailed description of the scheme.). Once we obtain a coherent UTM consisting of a set of highly correlated target genes under a certain subset of conditions, we terminate the iteration. In some cases, the input and output sets of target genes are the same. More often, some links are added or eliminated from the input. Links that are eliminated are likely to be deactivated under the subset of conditions represented in the UTM. Conversely, known and newly predicted target genes are likely to be activated (stimulated or repressed) by the TF under the subset of experimental conditions corresponding to its UTM. Similarly to the LINK model, we implemented two filters to the UTM genes retaining only the ones that are: a) correlated with the gene target associated with the link of interest and b) whose promoter regions have a good match with the TF binding site. We explored the parameter space for α and β, and found an optimal prediction rates at values of about α = 0.7 and β = 0.94 for the STAR model.

### Numerical results

We applied the LINK and STAR models to all the nodes in the composite *Saccharomyces cerevisiae *transcriptional regulatory network, and predicted 160 and 252 new links respectively. There were 53 new links that were predicted by both models. All predictions and their experimental conditions can be found in our online supplementary material section [1] [see Additional files 6, 7, 8, 9]. We compared our predictions with high confidence ChIP-on-chip binding data [6] and with other available manually curated network data [21]. The overlap between the LINK model predictions and ChIP-on-chip binding data [6] was 64 at binding p-values = 0.005 (41 bindings were found at *P *= 0.001 for which the binding site is conserved in at least three related yeast species; we note that PWMs [6] were generated by ChIP-on-chip data using binding events satisfying *P *= 0.001, for which the binding site is conserved across three of four related yeast species). In addition, six links were supported by the curated network data. The overlap between the STAR model predictions and ChIP-on-chip data was 126 at *P *= 0.005 (77 bindings were found at *P *= 0.001 for which the binding site is conserved in at least three related yeast species). Additional 14 links were supported by the curated network data. Table 1 shows predicted regulatory links that overlap with ChIP-on-chip data or other earlier studies. We note that stimulatory/inhibitory regulatory relationships were determined by positive/negative correlations between the regulator and its target gene under the conditions found in the UTM. We constructed a more comprehensive yeast transcriptional regulatory network by integrating the literature-driven gene regulatory networks compiled by Milo *et al. *[22] and Herrgard *et al *[21] [see Additional files 4, 5]. This combined network was used to identify new transcriptional regulatory interactions. All predicted transcriptional regulatory interactions obtained from this combined network and the experimental conditions associated with them can be found in the online supplementary material section [1] [see Additional file 8, 9].

### Extracting condition-specific transcriptional regulatory networks

**Figure 5:**
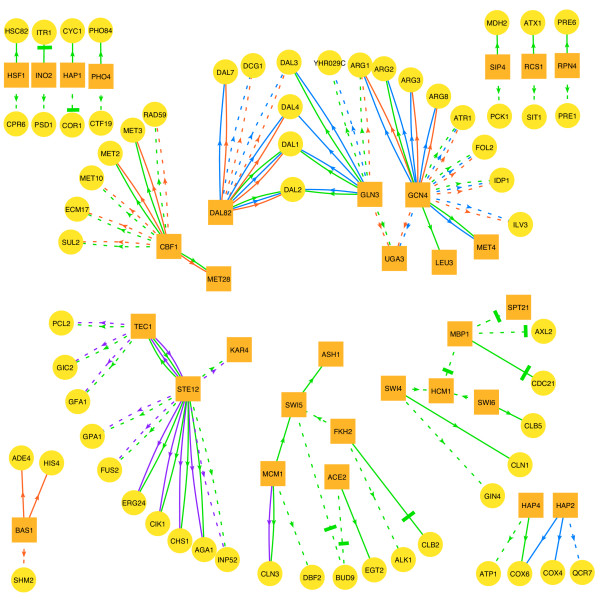
Extending the currently known parts of the regulatory network and breaking it down into state dependent networks [see Additional files 2]. Here we display a representative subset of known and predicted links in the yeast regulatory network along with categories of experimental conditions in which the target genes are controlled by their regulators. To simplify the display, we aggregated the experimental conditions into categories such as cell cycle (green links), amino acid starvation (orange), rapamycin treatment (blue), and alpha-factor treatment (purple). TFs are represented by squares and their target genes by circles. Solid and dotted links indicate the known and predicted regulatory links, respectively. The predicted experimental conditions in a UTM corresponding to a regulatory link tend to contain the experimental condition in which a TF binding on the promoter region of a target gene has been experimentally confirmed[6]. For example, the predicted regulatory links STE12→FUS2 and TEC1→GFA1 are supported by ChIP-on-chip location analyses performed with alpha factor pheromone treatment (purple-dotted links STE12→FUS2 and TEC1→GFA1).

**Figure 6:**
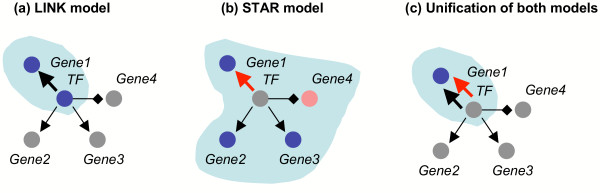
De-composing the composite network into condition specific regulatory links. To predict the experimental conditions for each TF-target gene link in the regulatory network, we unified the set of experimental conditions generated by the LINK and STAR models. We applied both models to the local networks containing the *TF*→*Gene*_1 _link. **(a) **A set of experimental conditions *(A) *in which the *TF*→*Gene*_1 _link is predicted to be active according to the LINK model. Under conditions *A*, *Gene*_1 _and *TF *are over-expressed. **(b) **A set of experimental conditions (*B*) in which the targets are activated by *TF *according to the STAR model. Under conditions *B*, *Genes*_1,2,3 _are stimulated and *Gene*_4 _is suppressed by *TF*. **(c) **Finally, we determine the experimental conditions in which the *TF*→*Gene*_1 _link is active by taking the union of *A *and *B*. The quality of our network de-composition has been assessed by using the union *A *∪ *B*.

We applied our algorithm to extract condition-specific transcriptional regulatory networks of *Saccharomyces cerevisiae*. To simplify the display of the multiple condition-specific networks obtained by breaking down the composite network, we aggregated the conditions from the gene expression compendium into the following categories: (1) cell cycle, (2) amino acid starvation, (3) rapamycin treated, and (4) alpha-factor treated. We compared the active links of our condition-aggregated networks to published ChIP-on-chip TF binding results[6] obtained under similar experimental conditions, such as amino acid starvation by the inhibitor of amino acid biosynthesis sulfometuron methyl, nutrient deprivation with rapamycin, and mating induction with the alpha factor pheromone. Figure 5 shows known (solid) and predicted (dotted) regulatory interactions along with their predicted experimental conditions obtained from the scheme presented in Figure 6. The network composed of the blue links represents an aggregated set of condition-specific networks associated with exposure to rapamycin. This figure shows only some representative links whose readouts for binding in ChIP-on-chip experiments are unlikely to be due to random binding (*P *≤ 0.001, provided that the binding site is conserved across three of four related yeast species). This small *P*-value for filtering binding events leads to a reduction in the number of links in this network. By applying the USA to each link in the network, we can read out the set of conditions under which the regulator stimulates or inhibits the expression of a known or predicted target gene. For example, the three regulatory links between DAL82 and DAL2 indicate that the DAL82→DAL2 link is active under the three categories of experimental conditions considered above. The predicted STE12→FUS2 and STE12→GPA1 binding were supported by ChIP-on-chip data under the experimental condition of mating induced by alpha factor treatment. DAL3 is activated by DAL82 with nutrient deprivation by rapamycin treatment. GFA1 is activated by TEC1 with mating inducing by alpha factor treatment. All experimental conditions for each regulation can be found in the online supplementary materials [1] [see Additional files 6, 7, 8, 9].

**Figure 7:**
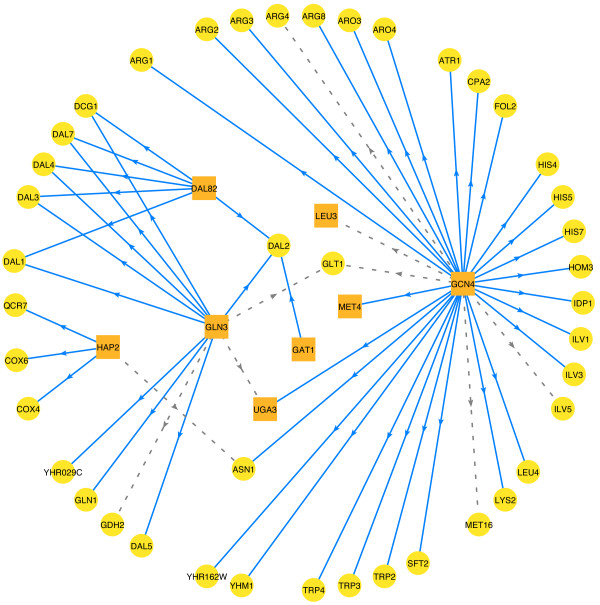
Predicting and validating condition specific networks. To find a condition specific network associated with treatment with rapamycin, we first analyzed all the local networks and identified from the respective UTMs all the links associated with this condition [see Additional files 3]. Here we show the sub-network consisting of links in the literature-driven gene regulatory networks [21, 22] and predicted links, which are supported by ChIP-on-chip binding assays (*P *≤ 0.001) with rapamycin treatment. Blue links represent pairs of TF-target genes bound to each other and predicted to be active in this condition (46/55). Dotted gray links (9/55) correspond to pairs bound to each other, in which our model failed to predict a condition-specific regulatory relationship.

To assess the extent to which the UTM-specified conditional regulatory interactions correspond to actual biological activity during the same specified conditions, we matched condition-specific ChIP-on-chip TF binding data from Harbison *et al *[6] to our predicted condition-specific TF-target gene interactions. Figure 7 summarizes the degree of overlap between our predicted condition-specific links and ChIP-on-chip TF binding data under rapamycin-induced nutrient deprivation. Specifically, 84.64% (46/53) of the (known and predicted) links contained in any of the outputs generated by the LINK and STAR models and confirmed by ChIP-on-chip binding (*P *≤ 0.001 for which the binding site is conserved in at least three related yeast species) under a rapamycin-induced condition belong to UTMs that include a similar condition from the compendium data. Under the conditions of mating inducing by treatment with alpha factor pheromone and amino acid starvation, we obtained prediction ratios of 57.78% and 73.85%, respectively.

### Assessing the quality of the predictions

We assessed the quality of the output obtained from the LINK and STAR models by: **(i) **comparing the predictions of the LINK model to predictions of other control/reference models. We measured the quality of the models by the extent of overlap between the predicted links to TF-target gene pairs confirmed by binding data from location analysis experiments [6] (a microarray method also termed ChIP-on-chip that uncovers the genome-wide location of DNA-bound protein), **(ii) **counting the rate at which we recapture known network links through application of the STAR model to networks in which these links are absent.

#### (a) Evaluating the performance of the LINK model

We evaluated the predictive value of the LINK model by comparing the ratio of predicted links confirmed by ChIP-on-chip data to the total number of predicted links (64/160) with the corresponding ratios generated from the following reference models: **(i) **NULL model in which new links are selected randomly rather than by using the UTM and PWM matching filters as implemented in the LINK model **(ii) **MATCH model in which links are selected randomly, but are filtered by retaining links with high PWM matching scores **(iii) **CORRELATE and MATCH model in which the predictions are based on overall correlations and PWM matching between all TFs and all genes **(iv) **LINK-UTM model in which the PWM matching step is omitted and links are obtained directly from the UTMs generated by the LINK model **(v)** CORRELATE in which the predictions are based on the overall co-expression (correlation across all experimental conditions) between TFs and genes.

In the NULL model, we randomly inserted 1242 new regulatory links (the same number of links predicted by the UTMs generated by the LINK model) into the literature-driven transcription regulatory network [22]. By repeating this procedure 50 times we found a mean overlap less than 1% between the 1242 randomly inserted links and TF-target gene ChIP-on-chip data determined by binding events with *P *≤ 0.005. Random links that were filtered using the MATCH model had on average approximately 6% overlap with ChIP-on-chip data. In the CORRELATE and MATCH model, we determined that a gene is regulated by a TF if the overall correlation between the gene and TF expression profiles (across the entire gene set of experimental conditions) satisfied the condition |*R*| > 0.5 (where *R *is the Pearson correlation coefficient), and if the TF PWM matched with the upstream region of a target gene with a MATCH score of β ≥ 0.94. We found that only 16 out of the 69 TF-gene pairs that satisfy these conditions overlapped with ChIP-on-chip data (approximately 23%). This overlap dropped to approximately 3% if we use correlation as a single filter for selecting TF-target gene pairs. By varying the parametric constraints we obtained similar frequencies of overlap. For example, for |*R*| > 0.4 and β ≥ 0.94, we obtained a ChIP-on-chip validation rate of 23.85% (83 out of 348 predictions). Finally, we found approximately 13% overlap between the experimental binding data and links extracted from the UTMs generated by the LINK model before subjecting them to the PWM-promoter target MATCH filter. Thus, the frequency with which the binding data correspond to the output generated by each of these five reference models was substantially lower than the rate of 40.0% obtained by the LINK method. We summarized the frequencies obtained in these models in Table 2. Since PWMs were derived from a subset of high confidence ChIP-on-chip binding events satisfying *P *≤ 0.001 and provided that the relevant sequence is conserved across three of four related yeast species, we also presented the overlap between the output of each model and binding events with *P *≤ 0.005 excluding these high confidence binding events. Table 2 clearly shows the advantage of using the UTM and PWM matching filters.

#### (b) Evaluating the performance of the STAR model

**Figure 8:**
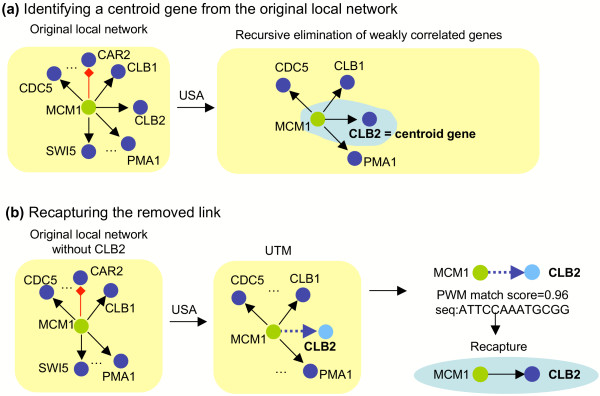
Validation procedure for the STAR model: In order to demonstrate the feasibility of the STAR model, we designed a recapturing scheme. We removed one link at a time from the network and examined whether this link can be recaptured by application of the STAR model to this reduced network. This figure explains the validation procedure using the MCM1 – CLB2 link as an example. **(a) **We first applied the USA to the local (STAR-like) network of MCM1 to find experimental conditions in which the core input genes are over-expressed or under-expressed. We then evaluated the correlations between all the targets of MCM1 under these (UTM) conditions, and removed targets whose correlation with other members of the core set are insignificant. We recursively applied the USA in order to remove MCM1 target genes that are weakly correlated with any other of the MCM1 target genes. At each step of the recursive elimination, we identified a centroid gene that has the largest number of highly correlated genes. We eliminated genes that are not highly correlated with the centroid gene. We continued these iterations until all the remaining target genes are highly correlated with each other. CLB2 was identified as the centroid in the last iteration. **(b) **In the next step we removed the link MCM1→CLB2 associated with the centroid gene, and applied the STAR model described in Figure l(b). As shown, CLB2 reappeared in the UTM generated from the application of the STAR model to a core set of input genes that excludes CLB2. Moreover, the matching score between the PWM of MCM1 and the promoter sequence of CLB2 was high. Overall, we recaptured 36% of the centroid genes by applying this procedure to all the multi-target local networks in the literature-driven gene regulatory network.

As an alternative for evaluating the performance of the STAR model we removed one link at a time from the network compiled by Milo *et al*, and inspected whether application of the model to these reduced networks recaptures the links that were stripped off (Fig. 8). To perform this analysis we had to select TFs that have known PWMs, and whose coherent UTMs consist of at least two genes. These two conditions enable us to: **(i) **apply the STAR model to the truncated local network and examine if the removed link is recaptured in the UTM, **(ii) **inspect the match between the promoter region of the recaptured gene and its regulator. Of the 102 TFs [6] that have PWMs, only 33 TFs satisfied both conditions. Overall, 12 links out of 33 removed links were recaptured. This recapturing rate (36.36%) was slightly lower than the prediction rates for new links. This results from our procedure, whereby we chose to remove the most cohesive (centroid) gene in a UTM. However, this rate was still significantly better than the predictions we obtained by using overall correlations.

By matching the PWMs with the promoter region of the target genes we increase the reliability of our predictions. This step reduces the number of putative targets from 1242 to 160 (87.22% reduction) for the LINK model and from 1039 to 252 (75.75% reduction) for the STAR model.

## Discussion

We present a novel method for identifying new transcriptional regulatory interactions together with the conditions in which these new and experimentally known interactions are active. An important feature of our method is the utilization of a composite regulatory network consisting of known TF-target gene interactions, where these links have been identified in different studies and experimental conditions. To the best of our knowledge, this is the first introduction of a methodology for de-composition of the composite network to condition specific networks. The novelty of this method is that predictions are based on the integration of this *a priori *transcriptional regulatory network information with a compendium of gene expression profiles as well as matching of TF DNA binding motifs (PWMs) to promoter sequences of candidate target genes. Our models employ the united signature algorithm, which efficiently captures both negatively- and positively-correlated genes under specific experimental conditions. This algorithm makes it possible to identify inhibitory and stimulatory regulatory interactions.

This approach confers a number of advantages over previously published transcriptional network studies: (1) it predicts not only a TF-target gene direct regulatory relationship, but also determines whether this relationship is inhibitory or stimulatory, (2) for each regulatory link it provides a unique subset of conditions (selected from a large compendium of experimental conditions) in which it is expected to be active, (3) its prediction rate is relatively high. Improved prediction rates can be attributed to: **(i)** the use of comprehensive biological input that includes all the known transcriptional regulatory links, large gene expression datasets and TF-DNA binding motif information, **(ii)** utilization of PWMs in discriminating between direct and indirect interactions of co-expressed TF-gene pairs, **(iii)** the unique properties of the united signature algorithm (USA) involving the input of known inhibitory and stimulatory TF-gene interactions, which in turn allow us to identify transcriptional modules consisting of negatively as well as positively correlated genes under experimental conditions relevant to the activity or inactivity of TFs.

Methods employing prior knowledge of TF binding motifs are limited by the reliability of binding predictions, which depend on the quality of the PWMs of each TF. The challenge of integrating TF binding and expression array data is further complicated by the fact that, in most cases, the gene expression profiles were not generated under the same experimental conditions used to collect ChIP-on-chip data. We endeavored to match each condition-specific ChIP-on-chip experiment to expression profiles generated under the most similar experimental environment. Nevertheless, collection of binding and expression data is ideally done under the same experimental conditions to reveal the condition specific transcriptional regulatory network [7,23].

Intersections between experimental conditions present in the UTMs of converging links can indirectly reveal some of the combinatorial regulations in the yeast regulatory network. A plausible mechanism for predicting the combination of TFs that regulate a target gene is to use a deconvolution model [24] or regression model in which the expression profiles of its regulators play the role of predictor variables. We attempt to directly model these TF combinations using a reversed-STAR model, in which the central gene is the target gene and all the links in this local network emanate from its regulators. This model led to results with low overlap with ChIP-on-chip experiments (data not shown). Future experiments will allow us to significantly improve the performance of this model by substituting the mRNA expression profiles of the regulators with protein expression and phosphorylation profiles.

## Conclusion

We have presented two models for extending the yeast transcriptional regulatory network. The LINK model is particularly useful when the TF has a single target, whereas the STAR model is applicable when the TF has multiple targets. The LINK model is based on the assumption that mRNA levels of a target gene and its regulator are highly correlated. The picture is more complicated for proteins that undergo post-translational modification (such as phosphorylation) to become functional. With the STAR model the TF expression pattern is not taken into consideration and there is no assumption regarding co-expression of a TF and its target gene at the mRNA level. These models complement each other, and are best used in combination, as shown here.

This approach makes experimentally testable predictions that can considerably narrow the scope of TFs that must be surveyed to understand gene regulation.

## Methods

### Experimental data

We used a compendium of *S. cerevisiae *gene expression data compiled by Ihmels *et al *[9]. The number of experimental conditions in this compendium is 1,011 of which we selected the 387 conditions associated with diploid cell. We used the *S. cerevisiae *transcriptional regulatory network published by Milo *et al *[22], which was derived from the YPD database [25] [see Additional file 4]. We excluded the TF-target pairs whose regulatory relationships alternate between stimulatory and inhibitory control. Genes missing from the compendium microarray expression data were also excluded from this study, leaving us with 6,206 genes of which 115 are TFs. Transcription regulatory binding motifs and probe sequences extracted from ChIP-on-chip experiments [6,26] were used to predict transcriptional regulatory interactions. These data consist of the average binding ratio and the associated *P*-value from the triplicate experiments, which were calculated for each TF by using a weighted average analysis model adapted from Hughes *et al *[27]. The standard symbols and aliases for genes were downloaded from the MIPS Comprehensive Yeast Genome Database [28]. The yeast promoter sequences used in our analysis were downloaded from the website of the location analysis experiments by Harbison et al [6].

### United Signature Algorithm (USA)

The USA is a variant of the signature algorithm (SA) [8-10]. We used notation similar to that described by Ihmels *et al *[9]. We implemented this algorithm using the composite regulatory network and expression data described in the experimental data section (and schematized in the upper panel of Fig. 1) to find transcriptional modules consisting of a set of input genes localized in the composite network and other genes that are co-regulated with the input genes under certain conditions. The flow of the procedures included in the USA is illustrated in Figure 3. Specifically, the USA includes the following steps:

I. Preprocess the gene expression data stored in the expression matrix E ∈ ℜ^*m *× *n *^by computing a two-way matrix normalization [14] accompanied by a log transformation of the gene expression ratios *G *= {g_1_, ..., *g*_*m*_} under experimental conditions C = {c_1_, ..., c_*n*_}, where *m *and *n *denote the total number of genes and experiments, respectively (Fig. 3a). The preprocessed expression of gene *g *under condition *c *is stored in a table represented by the matrix E_*gc*_.

II. Input the expression profile of the target gene associated with the *link of interest *together with the expression profiles of all the other genes known to be regulated by the TF associated with this link, or input the expression profiles of the TF and the target gene of this link (Fig. 3b). This information is represented by the sub-matrix (E_*gc*_)_*g*∈*T*_, whose rows correspond to the log transformed and (zero) centered expression levels of the input genes. Here *T *represents the set of input genes.

III. Multiply the elements of each row in the input sub-matrix (E_*gc*_)_*g*∈*T *_by a weight factor *w*_*g *_that can take one of two values: -1 if the regulatory relationship between the input gene *g *and the TF is inhibitory (see profile inversion in Fig. 3c), or +1 if the TF- target gene relationship is stimulatory. Denote this sub-matrix by (*w*_*g*_E_*gc*_)_*g*∈*T*_.

IV. Define a score *s*_*c *_that measures the extent to which the input genes are up- or down-regulated collectively in each experimental condition. This score is determined by a weighted average involving the expression values of the input genes, i.e. *s*_*c *_= <*w*_*g *_E_*gc*_>_*g*∈*T*_, where <>_*i *_denotes the average with respect to *i *(Fig. 3c)

V. Derive a united transcriptional module condition set (Ω), in which all (or some) of the input genes are up-regulated or down-regulated relative to their collective expression levels in all the conditions in the yeast expression dataset. The set is determined by conditions satisfying the constraint Ω = {*c *∈ *C*: | *s*_*c *_- <*s*_*c*_>_*c*∈*C*_| > *t*_*c *_σ_*c*_}, where <*s*_*c*_>_*c*∈*C *_is the average of condition scores over all conditions, *t*_*c *_is a condition threshold value, and σ_c _is the standard deviation of the condition scores for all experimental conditions (Fig. 3c).

VI. To find putative genes that maybe co-regulated by the TF, which regulates the input genes, a gene score *s*_*g *_is designed to identify genes that are up- and down-regulated across the set of conditions (Ω), and whose pattern of up- and down- regulation is positively or negatively coordinated with a corresponding pattern of the condition scores *s*_*c *_across the condition set Ω. The gene score *s*_*g *_is defined by the weighted average *s*_*g *_= <*s*_*c *_E_*gc*_>_c ∈ Ω _across the condition set Ω. The elements in the weighted average are given by the log-transformed and centered expression levels E_*gc *_multiplied by the corresponding condition scores *s*_*c *_(Fig. 3d)

VII. Determinine the united transcriptional module's (UTM) gene set by selecting genes satisfying the constraint Γ = {*g *∈ *G *: |*s*_*g *_- <*s*_*g*_>_*g*∈*G *_| > *t*_*g *_σ_*g*_} under the selected experimental conditions (Ω), where *t*_*g *_is a gene threshold value, and σ_*g *_is the standard deviation of gene scores for all genes *G *(Fig. 3e).

VIII. Filter the gene list in the UTM by keeping only those genes whose Pearson correlation coefficient with the target gene or the regulator satisfy |R| > α across the experimental conditions of the UTM.

IX. If needed (see STAR model below), iterate steps II-VIII until all the genes in the final UTM are highly correlated with the target gene associated with the link of interest (or highly correlated with the centroid of the input genes within the UTM)

As *t*_*g *_increases, the number of genes in the UTM decreases. As *t*_*c *_increases, the number of conditions in the UTM decreases. We used the condition threshold *t*_*c *_= 1.5, which minimizes false-negatives in our prediction of putative transcriptional regulations and maximizes consistency of our new regulatory link predictions with ChIP-on-chip data. To demonstrate how changes in these parameters affect the results we provide a table (see supplementary material [1] or [see Additional file 10]) showing the coverage and overlap with ChIP-on-chip data in several slices in the parameter space.

### LINK model

This model involves the application of the USA to each link in the known regulatory network (left panel of Fig. 4), such that the list of input genes consists of the regulator and its target gene. To generate UTMs associated with each link we used condition and gene thresholds of *t*_*c *_= 1.5 and of *t*_*g *_= 4.0 respectively. We further filtered the gene list by keeping only those genes whose correlation coefficient with the regulator (or alternatively with the target gene of interest) satisfied |*R*| > 0.5 across the experimental conditions of the UTM. Then, we evaluated the match between the position-specific weight matrix (PWM) [6] of the regulator with the upstream intergenic region of each putative target gene, and retained genes with a MATCH score equal to or greater than a predetermined cutoff β. Any TF-gene pair that belongs to a UTM, has |*R*| > 0.5, and have a MATCH score ≥ 0.94 was selected as a putative transcriptional regulatory link.

### STAR model

In this model, we applied the USA to an input set of genes including the target gene of the LINK model and all the other target genes known to be regulated by the same TF (right panel of Fig. 4). This model is applicable when the number of target genes is ≥ 2. In order to obtain a cluster of highly correlated target genes, we used the following iterative elimination scheme: We first obtained a UTM and computed all the correlations between its member genes across the conditions contained in the UTM. We then retained genes whose correlation with the target gene associated with the link of interest satisfied |*R*| > 0.7, where R is the Pearson correlation coefficient (alternatively, we retained genes whose correlation with some of the other genes within the UTM satisfied |*R*| > 0.7 and identified a centroid gene whose average correlation with all of these genes is the highest). Input genes that are weakly correlated with the target (or centroid) gene were eliminated from the next iteration. We iterated the USA until all the genes in the final UTM were highly correlated with the target (or centroid) gene. As in the LINK model, the PWM[6] of the regulator was used to search for a binding site sequence in the promoter regions of the target genes of the final UTM. Genes that belong to the final UTMs that have a high matching score with their respective PWMs are predicted to be targets of the corresponding TFs. To obtain UTMs with genes that were significantly correlated, we used a range of *t*_*g *_= (3,4).

## Authors' contributions

All authors participated in the design of the study and writing of the manuscript. All authors read and approved the final manuscript.

## Supplementary Material

Additional File 1 overview of supplemental data Click here for file

Additional File 2 experimental conditions for each link in figure 5. These are the experimental conditions in which the links are likely to be active. Click here for file

Additional File 3 experimental conditions for each link in figure 7. These are the experimental conditions in which the links are likely to be active. Click here for file

Additional File 4 Alon's transcriptional regulatory sub-network. Sparse representation of the Alon's network where column one represents the TF and column two represents the target. Entry of 1(2) corresponds to activation (suppression) Click here for file

Additional File 5 The Union of Alon's and Palsson's transcriptional regulatory sub-networks. Sparse representation of this unified network where column one represents the TF and column two represents the target. Entry of 1(2) corresponds to activation (suppression) Click here for file

Additional File 6 Predicted transcriptional regulatory links obtained by applying the LINK model to Alon's network. Each link is accompanied by a list of experiments in which it is likely to be functional. Click here for file

Additional File 7 Predicted transcriptional regulatory links obtained by applying the LINK model to the combined network obtained by unifying Alon's and Palsson's networks. Each link is accompanied by a list of experiments in which it is likely to be functional. Click here for file

Additional File 8 Predicted transcriptional regulatory links obtained by applying the STAR model to Alon's network. Each link is accompanied by a list of experiments in which it is likely to be functional. Click here for file

Additional File 9 Predicted transcriptional regulatory links obtained by applying the STAR model to the combined network obtained by unifying Alon's and Palsson's networks. Each link is accompanied by a list of experiments in which it is likely to be functional. Click here for file

Additional File 10 Exploring the parameter space. Overlap between our condition specific predicted networks and condition specific ChIP-on-chip data. Click here for file

## Figures and Tables

**Table 1:** Examples of predicted TF-target gene pairs generated by the LINK and STAR models to the *S. cerevisiae *transcriptional regulatory network published by Milo et al. [22]. The table shows the overlap of these links with: a) high confidence ChIP-on-chip data [6] at P ≤ 0.001 and with sequence conservation across at least 3 yeast species, b) moderate confidence ChIP-on-chip data at P ≤ 0.005 but excluding the high confidence binding events in (a), or c) the literature-driven gene regulatory network constructed by Herrgard et al. [21]

	Links predicted by the LINK model
Overlap with ChIP-on-chip data at *P *≤ 0.001 with sequence conservation in at least 3 yeast species	ACE2→CST13, GAT1→DAL2, GCN4→ATR1, GCN4→FOL2, GCN4→IDP1, GCN4→ISU1, GLN3→ARG1, GLN3→UGA3, GLN3→YHR029C, ROX1⊣YLR413W, STE12→FUS2, STE12→GPA1, STE12→INP52, STE12→KAR4, STE12→TEC1, SWI4→OCH1, SWI5→YPL158C, TEC1→GFA1, TEC1→GIC2, TEC1→PCL2, TEC1→STE12, UME6→YOR291W, YAP1→CYT2

Overlap with ChIP-on-chip data at *P *≤ 0.005 excluding events at *P *≤ 0.001 with sequence conservation across at least 3 yeast species	ACE2→SCW11, ASH1→HSP150, ASH1→PIR1, ASH1→PIR3, DAL80→YLR053C, GAT1→PUT1, GCN4→YMC1, GCN4→ALD5, GCN4→YMC2, GCN4→CAF16, GCN4→BAT1, GCN4→ORT1, STE12→ASG7, STE12→YDR249C, STE12→MFA2, TEC1→PRM1, TEC1→PRM6, TEC1→AGA2, TEC1→KAR5, TEC1→PRP39, YAP1→AAD6, YAP1→GTT2, YAP1→YLR460C

Literature supported	BAS1→HIS7, DAL80→GAP1, MSN4→TPS2, STE12→MFA2, STE12→TEC1, TEC1→STE12

	
	Links predicted by the STAR model

Overlap with ChIP-on-chip data at *P *≤ 0.001 with sequence conservation in at least 3 yeast species	ACE2⊣BUD9, ADR1→PXA1, BAS1→HIS4, BAS1→SHM2, FKH2→ALK1, FKH2→SWI5, GAT1→DAL2, GCN4→ATR1, GCN4→FOL2, GCN4→IDP1, GCN4→ILV3, GCN4→UGA3, GCR1→CDC19, GLN3→CPS1, HAP4→ATP1, HSF1→CPR6, MCM1→YNL058C, MSN2→TSL1, MSN4→TSL1, PHO4→PHO86, RPN4→PUP2, STE12→FUS2, STE12→GPA1, STE12→INP52, STE12→KAR4, STE12→TEC1, SWI5⊣CYK3

Overlap with ChIP-on-chip data at *P *≤ 0.005 excluding events at *P *≤ 0.001 with sequence conservation across at least 3 yeast species	ABF2→RPS28A, ACE2→FAA3, ACE2→PRY3, ACE2→SCW11, DAL80→YLR053C, FKH2→ACE2, FKH2→HOF1, FKH2→YLR190W, FKH2→YOR315W, FKH2→YPL141C, GAT1→DAL3, GAT1→DAL5, GAT1→DAL7, GAT1→MEP2, GAT1→PUT1, GCN4→ALD5, GCN4→BAT1, GCN4→CAF16, GCN4→ORT1, GCN4→YMC1, GCN4→YMC2, GCN4→YNL129W, GLN3→MEP2, GLN3→OPT2, GLN3→YGR125W, GLN3→YMR088C, HAP4→NDI1, HAP4→SDH1, HSF1→HSP10, HSF1→HSP60, HSF1→TSL1, LEU3→BAT1, MCM1→BUD4, MCM1→YOR315W, RAP1⊣GPM1, RAP1⊣PGI1, RCS1→ARN1, RCS1→TAF1, RPN4→RPN12, RPN4→RPN6, SKN7⊣DDR48, SKN7⊣GPX2, STE12→ASG7, STE12→MFA2, SWI5⊣FAA3, SWI5⊣PIR1, SWI5⊣PRY3, SWI5⊣TEC1, YAP1→AAD6

Literature supported	BAS1→ADE1, BAS1→ADE13, BAS1→ADE17, BAS1→ADE2, BAS1→ADE5,7, BAS1→HIS1, BAS1→HIS4, BAS1→HIS7, DAL80→GAP1, GCR1→CDC19, HAP4→SDH1, HSF1→SSA4, STE12→MFA2, STE12→TEC1

**Table 2:** Overlap between ChIP-on-chip events with *P *≤ 0.005 (or *P *≤ 0.005 excluding high confidence binding events with *P *≤ 0.001 and with sequence conservation across at least 3 yeast species) and predicted links obtained by the LINK model and by other five reference models. The table shows that less than 1% of all possible randomly selected links occur in binding experiments. Moreover, only 3.32% of the TF-gene pairs with overall expression correlation of greater (or smaller) than 0.5 (or -0.5) overlap with binding experimental data. The overlap increases to 13.37% when we consider highly correlated TF-gene pairs under the experimental conditions of the UTMs generated by the LINK model. By filtering the predicted links via the PWM matching, we discriminate between direct and indirect predicted interactions of co-expressed TF-gene pairs. As shown in the table, 40% (14.37%) of the new links predicted by the LINK model overlap with ChIP-on-chip binding all events with P ≤ 0.005 (*P *≤ 0.005 excluding high confidence binding events with *P *≤ 0.001 and with sequence conservation across at least 3 yeast species). This is a substantially higher rate than the 23.19% (7.25%) rate obtained by using a simpler approach that combines correlation with PWM-promoter matching, but disregards information about other experimentally known links.

	Random links	Correlated links	LINK-UTM links
**Without PWM matching** ** ChIP *P *≤ 0.005**	0.81%	3.32%	13.37%
**With PWM matching ** **ChIP*P *≤ 0.005**	6.03%	23.19%	40.00%
**Without PWM matching** **ChIP *P *≤ 0.005 excluding high confidence binding events**	0.53%	1.05%	8.62%
**With PWM matching** **ChIP *P *≤ 0.005 excluding high confidence binding events**	3.56%	7.25%	14.37%
