# Supplementary material for: Unraveling condition specific gene transcriptional regulatory networks in Saccharomyces cerevisiae
Source: BMC Bioinformatics. 2006 Mar 21;7:165. doi: 10.1186/1471-2105-7-165 (PMC1488875; doi:10.1186/1471-2105-7-165)
Supplement: Additional File 1 — overview of supplemental data [file 1471-2105-7-165-S1.pdf]

Unraveling condition specific gene transcriptional regulatory networks in *Saccharomyces cerevisiae*

Hyunsoo Kim, William Hu, and Yuval Kluger*

Department of Cell Biology, NYU School of Medicine, Skirball Institute of Biomolecular Medicine, 540 First Avenue, 3rd Floor, New York, NY 10016, USA. *Correspondence should be addressed to Y.K. (kluger@saturn.med.nyu.edu).

Biological Data

- Gene expression compendium data: The *Saccharomyces cerevisiae* gene expression compendium data contains 6,206 genes and 384 experimental conditions. This is a subset of the Ihmels' compendium (Ihmels, J. et al. Nat Genet 31, 370-7 (2002), Ihmels, J. et al. Bioinformatics 20, 1993-2003 (2004)) which includes only experiments with diploid cells and excludes knockout experiments.
- Gene regulatory network: We used two literature-driven gene regulatory networks:
    - **Alon's network**: Milo, R. et al. Network motifs: simple building blocks of complex networks. Science 298, 824-7 (2002)
    - **Palsson's network**: Herrgard, M.J., Covert, M.W. & Palsson, B.O. Reconciling gene expression data with known genome-scale regulatory network structures. Genome Res 13, 2423-34 (2003)

Supplementary Materials

- Extending Alon's network
    - The regulatory links and associated experimental conditions predicted by the LINK model and the STAR model. The output is organized as follows: a) the predicted regulatory link is represented by TF -*-> gene (activation) or TF -*-| gene (suppression) b) The experimental conditions associated with this links are indicated below the link.
- Extending Alon's network + Palsson's network
    - To construct a more comprehensive yeast transcriptional regulatory network we integrated the literature-driven gene regulatory network compiled by (Milo et al. 2002) and (Herrgard et al. 2003). This network, which is based on experimental evidence, includes 144 TFs and 1411 TF-target gene interactions. This combined network was used to identify new transcriptional regulatory interactions. By applying the LINK and STAR models to this extended network, we predicted 244 and 419 new links, respectively. There were 84 new links that were predicted under both models. As before, we compared our predictions with published ChIP-on-chip data4, and found an overlap of 97 at $P < 0.005$ (62 bindings were found at $P < 0.001$ and 35 at $0.001 < P < 0.005$) between this data and the links predicted in the LINK model. Then overlap of 97 at $P < 0.005$ (62 bindings were found at $P < 0.001$ and 35 at $0.001 < P < 0.005$) between this data and the links predicted in the LINK model. The overlap between the STAR model predictions and ChIP-on-chip data was 190 at $P < 0.005$ (120 bindings were found at $P < 0.001$ and 70 at $0.001 < P < 0.005$).
    - The regulatory links and associated experimental conditions predicted by the LINK model and

the STAR model. About 45% (190 out of 419) of the predicted transcriptional regulatory links in the STAR model are supported by published ChIP-on-chip experimental data. Similarly, 39.75% (97 out of 244) of the predictions obtained by the LINK model are supported by ChIP-on-chip experiments.

  - Each link in Figure 4-5 represents a regulatory interaction that is active under an aggregate of predicted conditions from the following categories: (1) cell cycle (green), (2) amino acid starvation (orange), (3) rapamycin treated (blue), and (4) alpha-factor treated (purple). Here we display the specific experimental conditions associated with the regulartory links in Figure 3 and Figure 5. The known regulatory links are represented by TF --> gene (activation) or TF --| gene (suppression). These links are accompanied with their predicted experimental conditions. The predicted regulatory links are represented by TF -*-> gene or TF -*-| gene.

- MATLAB code (requires Bioinformatics toolbox and MATLAB version 7.0 or higher)
- Exploring the parameter space

NYU School of Medicine
Department of Cell Biology
540 First Ave., 3rd Floor
New York, NY 10016

Skirball Institute of Biomolecular Medicine
Email: kluger@saturn.med.nyu.edu
