# Supplementary material for: Unraveling condition specific gene transcriptional regulatory networks in Saccharomyces cerevisiae
Source: BMC Bioinformatics. 2006 Mar 21;7:165. doi: 10.1186/1471-2105-7-165 (PMC1488875; doi:10.1186/1471-2105-7-165)
Supplement: Additional File 2 — experimental conditions for each link in figure 5. These are the experimental conditions in which the links are likely to be active. [file 1471-2105-7-165-S2.pdf]

```
Each link in this file  (e.g. ACE2 -*-| BUD9) is followed by a list of the predicted experimental conditions in which it is likely to be active.

The number experimental conditions is indicated next to the link (e.g. ACE2 -*-| BUD9 exp. conditions:17)
The index of each experimental condition is shown in parenthesis (e.g. (1) 5. Expression during the cell cycle)

The total number of links in this figure is 85.


ACE2 -*-| BUD9 exp. conditions:17


    (1) 5. Expression during the cell cycle (alpha factor arrest and release)(10)
    (2) 5. Expression during the cell cycle (alpha factor arrest and release)(11)
    (3) 5. Expression during the cell cycle (alpha factor arrest and release)(12)
    (4) 5. Expression during the cell cycle (alpha factor arrest and release)(13)
    (5) 6. Expression during the cell cycle (cdc15 arrest and release)(2)
    (6) 6. Expression during the cell cycle (cdc15 arrest and release)(10)
    (7) 6. Expression during the cell cycle (cdc15 arrest and release)(11)
    (8) 6. Expression during the cell cycle (cdc15 arrest and release)(12)
    (9) 6. Expression during the cell cycle (cdc15 arrest and release)(22)
    (10) 6. Expression during the cell cycle (cdc15 arrest and release)(23)
    (11) 7. Expression during the cell Cycle (cdc28)(10)
    (12) 7. Expression during the cell Cycle (cdc28)(11)
    (13) 7. Expression during the cell Cycle (cdc28)(12)
    (14) 7. Expression during the cell Cycle (cdc28)(17)
    (15) 8. Expression during the cell cycle (cell size selection and release)(12)
    (16) 8. Expression during the cell cycle (cell size selection and release)(13)
    (17) 8. Expression during the cell cycle (cell size selection and release)(14)


ACE2 --> EGT2 exp. conditions:29


    (1) 4. Cell Cycle: Expression in response to Clb2p (set 2, 30 min)(1)
    (2) 5. Expression during the cell cycle (alpha factor arrest and release)(10)
    (3) 5. Expression during the cell cycle (alpha factor arrest and release)(11)
    (4) 5. Expression during the cell cycle (alpha factor arrest and release)(12)
    (5) 5. Expression during the cell cycle (alpha factor arrest and release)(13)
    (6) 5. Expression during the cell cycle (alpha factor arrest and release)(15)
    (7) 5. Expression during the cell cycle (alpha factor arrest and release)(16)
    (8) 5. Expression during the cell cycle (alpha factor arrest and release)(17)
    (9) 5. Expression during the cell cycle (alpha factor arrest and release)(18)
    (10) 6. Expression during the cell cycle (cdc15 arrest and release)(2)
    (11) 6. Expression during the cell cycle (cdc15 arrest and release)(10)
    (12) 6. Expression during the cell cycle (cdc15 arrest and release)(11)
    (13) 6. Expression during the cell cycle (cdc15 arrest and release)(12)
    (14) 6. Expression during the cell cycle (cdc15 arrest and release)(22)
    (15) 6. Expression during the cell cycle (cdc15 arrest and release)(23)
    (16) 7. Expression during the cell Cycle (cdc28)(10)
    (17) 7. Expression during the cell Cycle (cdc28)(11)
    (18) 7. Expression during the cell Cycle (cdc28)(12)
    (19) 7. Expression during the cell Cycle (cdc28)(17)
    (20) 8. Expression during the cell cycle (cell size selection and release)(10)
    (21) 8. Expression during the cell cycle (cell size selection and release)(11)
    (22) 8. Expression during the cell cycle (cell size selection and release)(12)
    (23) 8. Expression during the cell cycle (cell size selection and release)(13)
    (24) 8. Expression during the cell cycle (cell size selection and release)(14)
    (25) 26. Fink: Expression in diploid high copy TEC1(1)
    (26) PHO81c vs WT exp1(1)
    (27) 497. Brown enviromental changes :Heat Shock 000 minutes  hs-2(1)
    (28) wt_plus_gamma_90_min
    (29) DES460 (wt) - mock irradiation - 30 min


BAS1 --> ADE4 exp. conditions:22


    (1) 11. Expression during diauxic shift: 9h,11h,13h,15h,17h,19h,21h(3)
    (2) 89. Expression in response to 3-aminotriazole(1)
    (3) 95. Expression in response to 50ug/mL FK506(1)
    (4) 395. Rosetta 2000: Expression in response to 2-deoxy-D-glucose(1)
    (5) 402. Rosetta 2000: Expression in response to Itraconazole(1)
    (6) 407. Rosetta 2000: Expression in response to Tunicamycin(1)
    (7) 483. Expression in response to alkali: 10,20,40,60,80,100 min(1)
    (8) 483. Expression in response to alkali: 10,20,40,60,80,100 min(2)
    (9) 539. Brown enviromental changes :1 mM Menadione (105 min) redo(1)
    (10) 540. Brown enviromental changes :1 mM Menadione (120 min)redo(1)
    (11) 572. Brown enviromental changes :Hypo-osmotic shock - 5 min(1)
    (12) 579. Brown enviromental changes :aa starv 1 h(1)
    (13) 580. Brown enviromental changes :aa starv 2 h(1)
    (14) 581. Brown enviromental changes :aa starv 4 h(1)
    (15) 582. Brown enviromental changes :aa starv 6 h(1)
    (16) 583. Brown enviromental changes :Nitrogen Depletion 30 min.(1)
    (17) 584. Brown enviromental changes :Nitrogen Depletion 1 h(1)
    (18) 585. Brown enviromental changes :Nitrogen Depletion 2 h(1)
    (19) 586. Brown enviromental changes :Nitrogen Depletion 4 h(1)
    (20) 595. Brown enviromental changes :diauxic shift timecourse(1)
    (21) 602. Brown enviromental changes :YPD 4 h ypd-2(1)
    (22) 613. Brown enviromental changes :YPD stationary phase 8 h ypd-1(1)


BAS1 --> HIS4 exp. conditions:22


    (1) 11. Expression during diauxic shift: 9h,11h,13h,15h,17h,19h,21h(3)
    (2) 89. Expression in response to 3-aminotriazole(1)
    (3) 95. Expression in response to 50ug/mL FK506(1)
    (4) 395. Rosetta 2000: Expression in response to 2-deoxy-D-glucose(1)
    (5) 402. Rosetta 2000: Expression in response to Itraconazole(1)
    (6) 407. Rosetta 2000: Expression in response to Tunicamycin(1)
    (7) 483. Expression in response to alkali: 10,20,40,60,80,100 min(1)
    (8) 483. Expression in response to alkali: 10,20,40,60,80,100 min(2)
    (9) 539. Brown enviromental changes :1 mM Menadione (105 min) redo(1)
    (10) 540. Brown enviromental changes :1 mM Menadione (120 min)redo(1)
    (11) 572. Brown enviromental changes :Hypo-osmotic shock - 5 min(1)
    (12) 579. Brown enviromental changes :aa starv 1 h(1)
    (13) 580. Brown enviromental changes :aa starv 2 h(1)
    (14) 581. Brown enviromental changes :aa starv 4 h(1)
    (15) 582. Brown enviromental changes :aa starv 6 h(1)
    (16) 583. Brown enviromental changes :Nitrogen Depletion 30 min.(1)
    (17) 584. Brown enviromental changes :Nitrogen Depletion 1 h(1)
    (18) 585. Brown enviromental changes :Nitrogen Depletion 2 h(1)
```

(19) 586. Brown enviromental changes :Nitrogen Depletion 4 h(1)
(20) 595. Brown enviromental changes :diauxic shift timecourse(1)
(21) 602. Brown enviromental changes :YPD 4 h ypd-2(1)
(22) 613. Brown enviromental changes :YPD stationary phase 8 h ypd-1(1)

BAS1 -*-> SHM2 exp. conditions:22

(1) 11. Expression during diauxic shift: 9h,11h,13h,15h,17h,19h,21h(3)
(2) 89. Expression in response to 3-aminotriazole(1)
(3) 95. Expression in response to 50ug/mL FK506(1)
(4) 395. Rosetta 2000: Expression in response to 2-deoxy-D-glucose(1)
(5) 402. Rosetta 2000: Expression in response to Itraconazole(1)
(6) 407. Rosetta 2000: Expression in response to Tunicamycin(1)
(7) 483. Expression in response to alkali: 10,20,40,60,80,100 min(1)
(8) 483. Expression in response to alkali: 10,20,40,60,80,100 min(2)
(9) 539. Brown enviromental changes :1 mM Menadione (105 min) redo(1)
(10) 540. Brown enviromental changes :1 mM Menadione (120 min)redo(1)
(11) 572. Brown enviromental changes :Hypo-osmotic shock - 5 min(1)
(12) 579. Brown enviromental changes :aa starv 1 h(1)
(13) 580. Brown enviromental changes :aa starv 2 h(1)
(14) 581. Brown enviromental changes :aa starv 4 h(1)
(15) 582. Brown enviromental changes :aa starv 6 h(1)
(16) 583. Brown enviromental changes :Nitrogen Depletion 30 min.(1)
(17) 584. Brown enviromental changes :Nitrogen Depletion 1 h(1)
(18) 585. Brown enviromental changes :Nitrogen Depletion 2 h(1)
(19) 586. Brown enviromental changes :Nitrogen Depletion 4 h(1)
(20) 595. Brown enviromental changes :diauxic shift timecourse(1)
(21) 602. Brown enviromental changes :YPD 4 h ypd-2(1)
(22) 613. Brown enviromental changes :YPD stationary phase 8 h ypd-1(1)

CBF1 -*-> ECM17 exp. conditions:20

(1) 5. Expression during the cell cycle (alpha factor arrest and release)(7)
(2) 7. Expression during the cell Cycle (cdc28)(5)
(3) 7. Expression during the cell Cycle (cdc28)(6)
(4) 74. Expression in response to overproduction of activated Rho1p(1)
(5) 385. Rosetta 2000: Expression in cells with AUR1 under tet promoter(1)
(6) 387. Rosetta 2000: Expression in cells with ERG11 under tet promoter(1)
(7) 392. Rosetta 2000: Expression in cells with PMA1 under tet promoter(1)
(8) 407. Rosetta 2000: Expression in response to Tunicamycin(1)
(9) 578. Brown enviromental changes :aa starv 0.5 h(1)
(10) 579. Brown enviromental changes :aa starv 1 h(1)
(11) 580. Brown enviromental changes :aa starv 2 h(1)
(12) 581. Brown enviromental changes :aa starv 4 h(1)
(13) 582. Brown enviromental changes :aa starv 6 h(1)
(14) 583. Brown enviromental changes :Nitrogen Depletion 30 min.(1)
(15) 584. Brown enviromental changes :Nitrogen Depletion 1 h(1)
(16) 585. Brown enviromental changes :Nitrogen Depletion 2 h(1)
(17) 586. Brown enviromental changes :Nitrogen Depletion 4 h(1)
(18) DES460 + 0.2% MMS - 45 min
(19) wt_plus_gamma_5_min
(20) wt_plus_gamma_60_min

CBF1 -*-> MET10 exp. conditions:20

(1) 5. Expression during the cell cycle (alpha factor arrest and release)(7)
(2) 7. Expression during the cell Cycle (cdc28)(5)
(3) 7. Expression during the cell Cycle (cdc28)(6)
(4) 74. Expression in response to overproduction of activated Rho1p(1)
(5) 385. Rosetta 2000: Expression in cells with AUR1 under tet promoter(1)
(6) 387. Rosetta 2000: Expression in cells with ERG11 under tet promoter(1)
(7) 392. Rosetta 2000: Expression in cells with PMA1 under tet promoter(1)
(8) 407. Rosetta 2000: Expression in response to Tunicamycin(1)
(9) 578. Brown enviromental changes :aa starv 0.5 h(1)
(10) 579. Brown enviromental changes :aa starv 1 h(1)
(11) 580. Brown enviromental changes :aa starv 2 h(1)
(12) 581. Brown enviromental changes :aa starv 4 h(1)
(13) 582. Brown enviromental changes :aa starv 6 h(1)
(14) 583. Brown enviromental changes :Nitrogen Depletion 30 min.(1)
(15) 584. Brown enviromental changes :Nitrogen Depletion 1 h(1)
(16) 585. Brown enviromental changes :Nitrogen Depletion 2 h(1)
(17) 586. Brown enviromental changes :Nitrogen Depletion 4 h(1)
(18) DES460 + 0.2% MMS - 45 min
(19) wt_plus_gamma_5_min
(20) wt_plus_gamma_60_min

CBF1 --> MET2 exp. conditions:20

(1) 5. Expression during the cell cycle (alpha factor arrest and release)(7)
(2) 7. Expression during the cell Cycle (cdc28)(5)
(3) 7. Expression during the cell Cycle (cdc28)(6)
(4) 74. Expression in response to overproduction of activated Rho1p(1)
(5) 385. Rosetta 2000: Expression in cells with AUR1 under tet promoter(1)
(6) 387. Rosetta 2000: Expression in cells with ERG11 under tet promoter(1)
(7) 392. Rosetta 2000: Expression in cells with PMA1 under tet promoter(1)
(8) 407. Rosetta 2000: Expression in response to Tunicamycin(1)
(9) 578. Brown enviromental changes :aa starv 0.5 h(1)
(10) 579. Brown enviromental changes :aa starv 1 h(1)
(11) 580. Brown enviromental changes :aa starv 2 h(1)
(12) 581. Brown enviromental changes :aa starv 4 h(1)
(13) 582. Brown enviromental changes :aa starv 6 h(1)
(14) 583. Brown enviromental changes :Nitrogen Depletion 30 min.(1)
(15) 584. Brown enviromental changes :Nitrogen Depletion 1 h(1)
(16) 585. Brown enviromental changes :Nitrogen Depletion 2 h(1)
(17) 586. Brown enviromental changes :Nitrogen Depletion 4 h(1)
(18) DES460 + 0.2% MMS - 45 min
(19) wt_plus_gamma_5_min
(20) wt_plus_gamma_60_min

CBF1 --> MET28 exp. conditions:32

(1) 5. Expression during the cell cycle (alpha factor arrest and release)(6)

(2) 5. Expression during the cell cycle (alpha factor arrest and release)(7)
(3) 7. Expression during the cell Cycle (cdc28)(4)
(4) 7. Expression during the cell Cycle (cdc28)(5)
(5) 7. Expression during the cell Cycle (cdc28)(6)
(6) 8. Expression during the cell cycle (cell size selection and release)(2)
(7) 74. Expression in response to overproduction of activated Rho1p(1)
(8) 385. Rosetta 2000: Expression in cells with AUR1 under tet promoter(1)
(9) 387. Rosetta 2000: Expression in cells with ERG11 under tet promoter(1)
(10) 392. Rosetta 2000: Expression in cells with PMA1 under tet promoter(1)
(11) 404. Rosetta 2000: Expression in response to MMS(1)
(12) 407. Rosetta 2000: Expression in response to Tunicamycin(1)
(13) 494. Brown enviromental changes :Heat Shock 60 minutes hs-1(1)
(14) 578. Brown enviromental changes :aa starv 0.5 h(1)
(15) 579. Brown enviromental changes :aa starv 1 h(1)
(16) 580. Brown enviromental changes :aa starv 2 h(1)
(17) 581. Brown enviromental changes :aa starv 4 h(1)
(18) 582. Brown enviromental changes :aa starv 6 h(1)
(19) 583. Brown enviromental changes :Nitrogen Depletion 30 min.(1)
(20) 584. Brown enviromental changes :Nitrogen Depletion 1 h(1)
(21) 585. Brown enviromental changes :Nitrogen Depletion 2 h(1)
(22) 586. Brown enviromental changes :Nitrogen Depletion 4 h(1)
(23) 591. Brown enviromental changes :Nitrogen Depletion 3 d(1)
(24) 672. Expression in response to carbonyl cyanide m-chlorophenylhydrazone (CCCP) 90min(1)
(25) 673. Expression in response to oligomycin 60min(1)
(26) 674. Expression in response to oligomycin 120min(1)
(27) DES460 + 0.02% MMS - 15 min
(28) DES460 + 0.02% MMS - 30 min
(29) DES460 + 0.2% MMS - 45 min
(30) DES460 + 0.02% MMS - 60 min
(31) wt_plus_gamma_5_min
(32) wt_plus_gamma_60_min

CBF1 --> MET3 exp. conditions:20

(1) 5. Expression during the cell cycle (alpha factor arrest and release)(7)
(2) 7. Expression during the cell Cycle (cdc28)(5)
(3) 7. Expression during the cell Cycle (cdc28)(6)
(4) 74. Expression in response to overproduction of activated Rho1p(1)
(5) 385. Rosetta 2000: Expression in cells with AUR1 under tet promoter(1)
(6) 387. Rosetta 2000: Expression in cells with ERG11 under tet promoter(1)
(7) 392. Rosetta 2000: Expression in cells with PMA1 under tet promoter(1)
(8) 407. Rosetta 2000: Expression in response to Tunicamycin(1)
(9) 578. Brown enviromental changes :aa starv 0.5 h(1)
(10) 579. Brown enviromental changes :aa starv 1 h(1)
(11) 580. Brown enviromental changes :aa starv 2 h(1)
(12) 581. Brown enviromental changes :aa starv 4 h(1)
(13) 582. Brown enviromental changes :aa starv 6 h(1)
(14) 583. Brown enviromental changes :Nitrogen Depletion 30 min.(1)
(15) 584. Brown enviromental changes :Nitrogen Depletion 1 h(1)
(16) 585. Brown enviromental changes :Nitrogen Depletion 2 h(1)
(17) 586. Brown enviromental changes :Nitrogen Depletion 4 h(1)
(18) DES460 + 0.2% MMS - 45 min
(19) wt_plus_gamma_5_min
(20) wt_plus_gamma_60_min

CBF1 -*-> RAD59 exp. conditions:20

(1) 5. Expression during the cell cycle (alpha factor arrest and release)(7)
(2) 7. Expression during the cell Cycle (cdc28)(5)
(3) 7. Expression during the cell Cycle (cdc28)(6)
(4) 74. Expression in response to overproduction of activated Rho1p(1)
(5) 385. Rosetta 2000: Expression in cells with AUR1 under tet promoter(1)
(6) 387. Rosetta 2000: Expression in cells with ERG11 under tet promoter(1)
(7) 392. Rosetta 2000: Expression in cells with PMA1 under tet promoter(1)
(8) 407. Rosetta 2000: Expression in response to Tunicamycin(1)
(9) 578. Brown enviromental changes :aa starv 0.5 h(1)
(10) 579. Brown enviromental changes :aa starv 1 h(1)
(11) 580. Brown enviromental changes :aa starv 2 h(1)
(12) 581. Brown enviromental changes :aa starv 4 h(1)
(13) 582. Brown enviromental changes :aa starv 6 h(1)
(14) 583. Brown enviromental changes :Nitrogen Depletion 30 min.(1)
(15) 584. Brown enviromental changes :Nitrogen Depletion 1 h(1)
(16) 585. Brown enviromental changes :Nitrogen Depletion 2 h(1)
(17) 586. Brown enviromental changes :Nitrogen Depletion 4 h(1)
(18) DES460 + 0.2% MMS - 45 min
(19) wt_plus_gamma_5_min
(20) wt_plus_gamma_60_min

CBF1 -*-> SUL2 exp. conditions:20

(1) 5. Expression during the cell cycle (alpha factor arrest and release)(7)
(2) 7. Expression during the cell Cycle (cdc28)(5)
(3) 7. Expression during the cell Cycle (cdc28)(6)
(4) 74. Expression in response to overproduction of activated Rho1p(1)
(5) 385. Rosetta 2000: Expression in cells with AUR1 under tet promoter(1)
(6) 387. Rosetta 2000: Expression in cells with ERG11 under tet promoter(1)
(7) 392. Rosetta 2000: Expression in cells with PMA1 under tet promoter(1)
(8) 407. Rosetta 2000: Expression in response to Tunicamycin(1)
(9) 578. Brown enviromental changes :aa starv 0.5 h(1)
(10) 579. Brown enviromental changes :aa starv 1 h(1)
(11) 580. Brown enviromental changes :aa starv 2 h(1)
(12) 581. Brown enviromental changes :aa starv 4 h(1)
(13) 582. Brown enviromental changes :aa starv 6 h(1)
(14) 583. Brown enviromental changes :Nitrogen Depletion 30 min.(1)
(15) 584. Brown enviromental changes :Nitrogen Depletion 1 h(1)
(16) 585. Brown enviromental changes :Nitrogen Depletion 2 h(1)
(17) 586. Brown enviromental changes :Nitrogen Depletion 4 h(1)
(18) DES460 + 0.2% MMS - 45 min
(19) wt_plus_gamma_5_min
(20) wt_plus_gamma_60_min

DAL82 --> DAL1 exp. conditions:31

(1) 5. Expression during the cell cycle (alpha factor arrest and release)(5)

(2) 7. Expression during the cell Cycle (cdc28)(16)
(3) 26. Fink: Expression in diploid high copy TEC1(1)
(4) 49. Expression in response to 50 nM alpha-factor: 0,15,30,45,60,90,120 min(5)
(5) 428. Expression in strain PM38 (wild type) in response to 30 min 50 nM treatment with rapamycin in YPD(1)
(6) 429. Expression in strain YHE711 (wild type) in response to 30 min 50 nM treatment with rapamycin in YPD(1)
(7) 439. Expression in strain Jk9-3da (wild type) in response to 30 min 50 nM treatment with rapamycin in YPD(1)
(8) 442. Expression in strain PM38 (wild type) in response to 30 min 50 nM treatment with rapamycin in YPD(1)
(9) 479. Expression in diploid cells in response to rapamycin (100nM) for: 15min,30min,90min,120min(1)
(10) 479. Expression in diploid cells in response to rapamycin (100nM) for: 15min,30min,90min,120min(2)
(11) 531. Brown enviromental changes :constant 0.32 mM H2O2 (120 min) redo(1)
(12) 569. Brown enviromental changes :1M sorbitol - 60 min(1)
(13) 578. Brown enviromental changes :aa starv 0.5 h(1)
(14) 579. Brown enviromental changes :aa starv 1 h(1)
(15) 580. Brown enviromental changes :aa starv 2 h(1)
(16) 581. Brown enviromental changes :aa starv 4 h(1)
(17) 582. Brown enviromental changes :aa starv 6 h(1)
(18) 583. Brown enviromental changes :Nitrogen Depletion 30 min.(1)
(19) 584. Brown enviromental changes :Nitrogen Depletion 1 h(1)
(20) 585. Brown enviromental changes :Nitrogen Depletion 2 h(1)
(21) 586. Brown enviromental changes :Nitrogen Depletion 4 h(1)
(22) 587. Brown enviromental changes :Nitrogen Depletion 8 h(1)
(23) 588. Brown enviromental changes :Nitrogen Depletion 12 h(1)
(24) 589. Brown enviromental changes :Nitrogen Depletion 1 d(1)
(25) 590. Brown enviromental changes :Nitrogen Depletion 2 d(1)
(26) 591. Brown enviromental changes :Nitrogen Depletion 3 d(1)
(27) 592. Brown enviromental changes :Nitrogen Depletion 5 d(1)
(28) 681. Expression in response to 0.4M NaCl for 10 min in wild type(1)
(29) 100 microM CuSO4 30 min
(30) MAC1-up (C)
(31) wt+gal

DAL82 --> DAL2 exp. conditions:29

(1) 5. Expression during the cell cycle (alpha factor arrest and release)(5)
(2) 6. Expression during the cell cycle (cdc15 arrest and release)(9)
(3) 49. Expression in response to 50 nM alpha-factor: 0,15,30,45,60,90,120 min(5)
(4) 428. Expression in strain PM38 (wild type) in response to 30 min 50 nM treatment with rapamycin in YPD(1)
(5) 429. Expression in strain YHE711 (wild type) in response to 30 min 50 nM treatment with rapamycin in YPD(1)
(6) 439. Expression in strain Jk9-3da (wild type) in response to 30 min 50 nM treatment with rapamycin in YPD(1)
(7) 442. Expression in strain PM38 (wild type) in response to 30 min 50 nM treatment with rapamycin in YPD(1)
(8) 479. Expression in diploid cells in response to rapamycin (100nM) for: 15min,30min,90min,120min(1)
(9) 479. Expression in diploid cells in response to rapamycin (100nM) for: 15min,30min,90min,120min(2)
(10) 531. Brown enviromental changes :constant 0.32 mM H2O2 (120 min) redo(1)
(11) 569. Brown enviromental changes :1M sorbitol - 60 min(1)
(12) 570. Brown enviromental changes :1M sorbitol - 90 min(1)
(13) 578. Brown enviromental changes :aa starv 0.5 h(1)
(14) 579. Brown enviromental changes :aa starv 1 h(1)
(15) 580. Brown enviromental changes :aa starv 2 h(1)
(16) 581. Brown enviromental changes :aa starv 4 h(1)
(17) 582. Brown enviromental changes :aa starv 6 h(1)
(18) 583. Brown enviromental changes :Nitrogen Depletion 30 min.(1)
(19) 584. Brown enviromental changes :Nitrogen Depletion 1 h(1)
(20) 585. Brown enviromental changes :Nitrogen Depletion 2 h(1)
(21) 586. Brown enviromental changes :Nitrogen Depletion 4 h(1)
(22) 587. Brown enviromental changes :Nitrogen Depletion 8 h(1)
(23) 588. Brown enviromental changes :Nitrogen Depletion 12 h(1)
(24) 589. Brown enviromental changes :Nitrogen Depletion 1 d(1)
(25) 590. Brown enviromental changes :Nitrogen Depletion 2 d(1)
(26) 591. Brown enviromental changes :Nitrogen Depletion 3 d(1)
(27) 592. Brown enviromental changes :Nitrogen Depletion 5 d(1)
(28) 100 microM BCS 60 min
(29) wt+gal

DAL82 -*-> DAL3 exp. conditions:21

(1) 49. Expression in response to 50 nM alpha-factor: 0,15,30,45,60,90,120 min(5)
(2) 428. Expression in strain PM38 (wild type) in response to 30 min 50 nM treatment with rapamycin in YPD(1)
(3) 429. Expression in strain YHE711 (wild type) in response to 30 min 50 nM treatment with rapamycin in YPD(1)
(4) 439. Expression in strain Jk9-3da (wild type) in response to 30 min 50 nM treatment with rapamycin in YPD(1)
(5) 442. Expression in strain PM38 (wild type) in response to 30 min 50 nM treatment with rapamycin in YPD(1)
(6) 479. Expression in diploid cells in response to rapamycin (100nM) for: 15min,30min,90min,120min(2)
(7) 578. Brown enviromental changes :aa starv 0.5 h(1)
(8) 579. Brown enviromental changes :aa starv 1 h(1)
(9) 580. Brown enviromental changes :aa starv 2 h(1)
(10) 581. Brown enviromental changes :aa starv 4 h(1)
(11) 582. Brown enviromental changes :aa starv 6 h(1)
(12) 583. Brown enviromental changes :Nitrogen Depletion 30 min.(1)
(13) 584. Brown enviromental changes :Nitrogen Depletion 1 h(1)
(14) 585. Brown enviromental changes :Nitrogen Depletion 2 h(1)
(15) 586. Brown enviromental changes :Nitrogen Depletion 4 h(1)
(16) 587. Brown enviromental changes :Nitrogen Depletion 8 h(1)
(17) 588. Brown enviromental changes :Nitrogen Depletion 12 h(1)
(18) 589. Brown enviromental changes :Nitrogen Depletion 1 d(1)
(19) 590. Brown enviromental changes :Nitrogen Depletion 2 d(1)
(20) 591. Brown enviromental changes :Nitrogen Depletion 3 d(1)
(21) 592. Brown enviromental changes :Nitrogen Depletion 5 d(1)

DAL82 --> DAL4 exp. conditions:28

(1) 49. Expression in response to 50 nM alpha-factor: 0,15,30,45,60,90,120 min(5)
(2) 428. Expression in strain PM38 (wild type) in response to 30 min 50 nM treatment with rapamycin in YPD(1)
(3) 429. Expression in strain YHE711 (wild type) in response to 30 min 50 nM treatment with rapamycin in YPD(1)
(4) 439. Expression in strain Jk9-3da (wild type) in response to 30 min 50 nM treatment with rapamycin in YPD(1)
(5) 442. Expression in strain PM38 (wild type) in response to 30 min 50 nM treatment with rapamycin in YPD(1)
(6) 479. Expression in diploid cells in response to rapamycin (100nM) for: 15min,30min,90min,120min(2)
(7) 531. Brown enviromental changes :constant 0.32 mM H2O2 (120 min) redo(1)
(8) 569. Brown enviromental changes :1M sorbitol - 60 min(1)
(9) 578. Brown enviromental changes :aa starv 0.5 h(1)
(10) 579. Brown enviromental changes :aa starv 1 h(1)
(11) 580. Brown enviromental changes :aa starv 2 h(1)
(12) 581. Brown enviromental changes :aa starv 4 h(1)
(13) 582. Brown enviromental changes :aa starv 6 h(1)
(14) 583. Brown enviromental changes :Nitrogen Depletion 30 min.(1)
(15) 584. Brown enviromental changes :Nitrogen Depletion 1 h(1)
(16) 585. Brown enviromental changes :Nitrogen Depletion 2 h(1)
(17) 586. Brown enviromental changes :Nitrogen Depletion 4 h(1)

(18) 587. Brown enviromental changes :Nitrogen Depletion 8 h(1)
(19) 588. Brown enviromental changes :Nitrogen Depletion 12 h(1)
(20) 589. Brown enviromental changes :Nitrogen Depletion 1 d(1)
(21) 590. Brown enviromental changes :Nitrogen Depletion 2 d(1)
(22) 591. Brown enviromental changes :Nitrogen Depletion 3 d(1)
(23) 592. Brown enviromental changes :Nitrogen Depletion 5 d(1)
(24) 100 microM BCS 60 min
(25) 100 microM CuSO4 30 min
(26) MAC1-up (B)
(27) MAC1-up (C)
(28) wt+gal

DAL82 --> DAL7 exp. conditions:28

(1) 49. Expression in response to 50 nM alpha-factor: 0,15,30,45,60,90,120 min(5)
(2) 428. Expression in strain PM38 (wild type) in response to 30 min 50 nM treatment with rapamycin in YPD(1)
(3) 429. Expression in strain YHE711 (wild type) in response to 30 min 50 nM treatment with rapamycin in YPD(1)
(4) 439. Expression in strain Jk9-3da (wild type) in response to 30 min 50 nM treatment with rapamycin in YPD(1)
(5) 442. Expression in strain PM38 (wild type) in response to 30 min 50 nM treatment with rapamycin in YPD(1)
(6) 479. Expression in diploid cells in response to rapamycin (100nM) for: 15min,30min,90min,120min(2)
(7) 531. Brown enviromental changes :constant 0.32 mM H2O2 (120 min) redo(1)
(8) 569. Brown enviromental changes :1M sorbitol - 60 min(1)
(9) 578. Brown enviromental changes :aa starv 0.5 h(1)
(10) 579. Brown enviromental changes :aa starv 1 h(1)
(11) 580. Brown enviromental changes :aa starv 2 h(1)
(12) 581. Brown enviromental changes :aa starv 4 h(1)
(13) 582. Brown enviromental changes :aa starv 6 h(1)
(14) 583. Brown enviromental changes :Nitrogen Depletion 30 min.(1)
(15) 584. Brown enviromental changes :Nitrogen Depletion 1 h(1)
(16) 585. Brown enviromental changes :Nitrogen Depletion 2 h(1)
(17) 586. Brown enviromental changes :Nitrogen Depletion 4 h(1)
(18) 587. Brown enviromental changes :Nitrogen Depletion 8 h(1)
(19) 588. Brown enviromental changes :Nitrogen Depletion 12 h(1)
(20) 589. Brown enviromental changes :Nitrogen Depletion 1 d(1)
(21) 590. Brown enviromental changes :Nitrogen Depletion 2 d(1)
(22) 591. Brown enviromental changes :Nitrogen Depletion 3 d(1)
(23) 592. Brown enviromental changes :Nitrogen Depletion 5 d(1)
(24) 100 microM BCS 60 min
(25) 100 microM CuSO4 30 min
(26) MAC1-up (B)
(27) MAC1-up (C)
(28) wt+gal

DAL82 -*-> DCG1 exp. conditions:28

(1) 49. Expression in response to 50 nM alpha-factor: 0,15,30,45,60,90,120 min(5)
(2) 428. Expression in strain PM38 (wild type) in response to 30 min 50 nM treatment with rapamycin in YPD(1)
(3) 429. Expression in strain YHE711 (wild type) in response to 30 min 50 nM treatment with rapamycin in YPD(1)
(4) 439. Expression in strain Jk9-3da (wild type) in response to 30 min 50 nM treatment with rapamycin in YPD(1)
(5) 442. Expression in strain PM38 (wild type) in response to 30 min 50 nM treatment with rapamycin in YPD(1)
(6) 479. Expression in diploid cells in response to rapamycin (100nM) for: 15min,30min,90min,120min(2)
(7) 531. Brown enviromental changes :constant 0.32 mM H2O2 (120 min) redo(1)
(8) 569. Brown enviromental changes :1M sorbitol - 60 min(1)
(9) 578. Brown enviromental changes :aa starv 0.5 h(1)
(10) 579. Brown enviromental changes :aa starv 1 h(1)
(11) 580. Brown enviromental changes :aa starv 2 h(1)
(12) 581. Brown enviromental changes :aa starv 4 h(1)
(13) 582. Brown enviromental changes :aa starv 6 h(1)
(14) 583. Brown enviromental changes :Nitrogen Depletion 30 min.(1)
(15) 584. Brown enviromental changes :Nitrogen Depletion 1 h(1)
(16) 585. Brown enviromental changes :Nitrogen Depletion 2 h(1)
(17) 586. Brown enviromental changes :Nitrogen Depletion 4 h(1)
(18) 587. Brown enviromental changes :Nitrogen Depletion 8 h(1)
(19) 588. Brown enviromental changes :Nitrogen Depletion 12 h(1)
(20) 589. Brown enviromental changes :Nitrogen Depletion 1 d(1)
(21) 590. Brown enviromental changes :Nitrogen Depletion 2 d(1)
(22) 591. Brown enviromental changes :Nitrogen Depletion 3 d(1)
(23) 592. Brown enviromental changes :Nitrogen Depletion 5 d(1)
(24) 100 microM BCS 60 min
(25) 100 microM CuSO4 30 min
(26) MAC1-up (B)
(27) MAC1-up (C)
(28) wt+gal

FKH2 -*-> ALK1 exp. conditions:18

(1) 5. Expression during the cell cycle (alpha factor arrest and release)(1)
(2) 5. Expression during the cell cycle (alpha factor arrest and release)(3)
(3) 5. Expression during the cell cycle (alpha factor arrest and release)(4)
(4) 5. Expression during the cell cycle (alpha factor arrest and release)(5)
(5) 6. Expression during the cell cycle (cdc15 arrest and release)(3)
(6) 6. Expression during the cell cycle (cdc15 arrest and release)(11)
(7) 8. Expression during the cell cycle (cell size selection and release)(2)
(8) 8. Expression during the cell cycle (cell size selection and release)(4)
(9) 8. Expression during the cell cycle (cell size selection and release)(5)
(10) 49. Expression in response to 50 nM alpha-factor: 0,15,30,45,60,90,120 min(4)
(11) 49. Expression in response to 50 nM alpha-factor: 0,15,30,45,60,90,120 min(6)
(12) 387. Rosetta 2000: Expression in cells with ERG11 under tet promoter(1)
(13) 430. Expression in strain PM38 (wild type), glucose versus ethanol: strain was shifted from medium containing dextrose as carbon source, ammonium sulfate as nirogen source, supplemented with leucine and uracil to same medium for 30 min, compared to a shift to a medium with synthetic ethanol instead of glucose for 30 min(1)
(14) 590. Brown enviromental changes :Nitrogen Depletion 2 d(1)
(15) 592. Brown enviromental changes :Nitrogen Depletion 5 d(1)
(16) 607. Brown enviromental changes :YPD 1 d ypd-2(1)
(17) 617. Brown enviromental changes :YPD stationary phase 3 d ypd-1(1)
(18) DES460 + 0.02% MMS - 60 min

FKH2 --| CLB2 exp. conditions:18

(1) 5. Expression during the cell cycle (alpha factor arrest and release)(1)
(2) 5. Expression during the cell cycle (alpha factor arrest and release)(3)
(3) 5. Expression during the cell cycle (alpha factor arrest and release)(4)
(4) 5. Expression during the cell cycle (alpha factor arrest and release)(5)
(5) 6. Expression during the cell cycle (cdc15 arrest and release)(3)
(6) 6. Expression during the cell cycle (cdc15 arrest and release)(11)

(7) 8. Expression during the cell cycle (cell size selection and release)(2)
(8) 8. Expression during the cell cycle (cell size selection and release)(4)
(9) 8. Expression during the cell cycle (cell size selection and release)(5)
(10) 49. Expression in response to 50 nM alpha-factor: 0,15,30,45,60,90,120 min(4)
(11) 49. Expression in response to 50 nM alpha-factor: 0,15,30,45,60,90,120 min(6)
(12) 387. Rosetta 2000: Expression in cells with ERG11 under tet promoter(1)
(13) 430. Expression in strain PM38 (wild type), glucose versus ethanol: strain was shifted from medium containing dextrose as carbon source, ammonium sulfate as nirogen source, supplemented with leucine and uracil to same medium for 30 min, compared to a shift to a medium with synthetic ethanol instead of glucose for 30 min(1)
(14) 590. Brown enviromental changes :Nitrogen Depletion 2 d(1)
(15) 592. Brown enviromental changes :Nitrogen Depletion 5 d(1)
(16) 607. Brown enviromental changes :YPD 1 d ypd-2(1)
(17) 617. Brown enviromental changes :YPD stationary phase 3 d ypd-1(1)
(18) DES460 + 0.02% MMS - 60 min

FKH2 -*-> SWI5 exp. conditions:18

(1) 5. Expression during the cell cycle (alpha factor arrest and release)(1)
(2) 5. Expression during the cell cycle (alpha factor arrest and release)(3)
(3) 5. Expression during the cell cycle (alpha factor arrest and release)(4)
(4) 5. Expression during the cell cycle (alpha factor arrest and release)(5)
(5) 6. Expression during the cell cycle (cdc15 arrest and release)(3)
(6) 6. Expression during the cell cycle (cdc15 arrest and release)(11)
(7) 8. Expression during the cell cycle (cell size selection and release)(2)
(8) 8. Expression during the cell cycle (cell size selection and release)(4)
(9) 8. Expression during the cell cycle (cell size selection and release)(5)
(10) 49. Expression in response to 50 nM alpha-factor: 0,15,30,45,60,90,120 min(4)
(11) 49. Expression in response to 50 nM alpha-factor: 0,15,30,45,60,90,120 min(6)
(12) 387. Rosetta 2000: Expression in cells with ERG11 under tet promoter(1)
(13) 430. Expression in strain PM38 (wild type), glucose versus ethanol: strain was shifted from medium containing dextrose as carbon source, ammonium sulfate as nirogen source, supplemented with leucine and uracil to same medium for 30 min, compared to a shift to a medium with synthetic ethanol instead of glucose for 30 min(1)
(14) 590. Brown enviromental changes :Nitrogen Depletion 2 d(1)
(15) 592. Brown enviromental changes :Nitrogen Depletion 5 d(1)
(16) 607. Brown enviromental changes :YPD 1 d ypd-2(1)
(17) 617. Brown enviromental changes :YPD stationary phase 3 d ypd-1(1)
(18) DES460 + 0.02% MMS - 60 min

GCN4 --> ARG1 exp. conditions:26

(1) 89. Expression in response to 3-aminotriazole(1)
(2) 95. Expression in response to 50ug/mL FK506(1)
(3) 332. Rosetta 2000: Expression in cells with CMD1 under tet promoter(1)
(4) 387. Rosetta 2000: Expression in cells with ERG11 under tet promoter(1)
(5) 395. Rosetta 2000: Expression in response to 2-deoxy-D-glucose(1)
(6) 401. Rosetta 2000: Expression in response to HU(1)
(7) 402. Rosetta 2000: Expression in response to Itraconazole(1)
(8) 403. Rosetta 2000: Expression in response to Lovastatin(1)
(9) 406. Rosetta 2000: Expression in response to Terbinafine(1)
(10) 407. Rosetta 2000: Expression in response to Tunicamycin(1)
(11) 445. Expression in response to 0.1% MMS for 60 min (average of 3 experiments)(1)
(12) 446. Expression in response to 0.1% MMS for 10 min(1)
(13) 447. Expression in response to 0.1% MMS for 30 min(1)
(14) 448. Expression in response to 0.1% MMS for 60 min(1)
(15) 449. Expression in response to 0.1% MMS for 60 min(1)
(16) 462. Expression in response to 0.05% MMS for 60 min(1)
(17) 463. Expression in response to 0.1% MMS for 60 min(1)
(18) 479. Expression in diploid cells in response to rapamycin (100nM) for: 15min,30min,90min,120min(3)
(19) 533. Brown enviromental changes :1 mM Menadione (10 min)redo(1)
(20) 579. Brown enviromental changes :aa starv 1 h(1)
(21) 580. Brown enviromental changes :aa starv 2 h(1)
(22) 581. Brown enviromental changes :aa starv 4 h(1)
(23) 584. Brown enviromental changes :Nitrogen Depletion 1 h(1)
(24) 585. Brown enviromental changes :Nitrogen Depletion 2 h(1)
(25) 586. Brown enviromental changes :Nitrogen Depletion 4 h(1)
(26) DES460 + 0.02% MMS - 15 min

GCN4 --> ARG2 exp. conditions:24

(1) 5. Expression during the cell cycle (alpha factor arrest and release)(11)
(2) 6. Expression during the cell cycle (cdc15 arrest and release)(15)
(3) 54. Expression in response to overproduction of Ste5p(1)
(4) 89. Expression in response to 3-aminotriazole(1)
(5) 95. Expression in response to 50ug/mL FK506(1)
(6) 402. Rosetta 2000: Expression in response to Itraconazole(1)
(7) 403. Rosetta 2000: Expression in response to Lovastatin(1)
(8) 406. Rosetta 2000: Expression in response to Terbinafine(1)
(9) 407. Rosetta 2000: Expression in response to Tunicamycin(1)
(10) 429. Expression in strain YHE711 (wild type) in response to 30 min 50 nM treatment with rapamycin in YPD(1)
(11) 446. Expression in response to 0.1% MMS for 10 min(1)
(12) 447. Expression in response to 0.1% MMS for 30 min(1)
(13) 452. Expression in response to low 4NQO (2 microgram/ml) for 60 min(1)
(14) 479. Expression in diploid cells in response to rapamycin (100nM) for: 15min,30min,90min,120min(2)
(15) 481. Expression in response to heat shock: 15,30,45,60,120 min(2)
(16) 481. Expression in response to heat shock: 15,30,45,60,120 min(4)
(17) 537. Brown enviromental changes :1 mM Menadione (50 min)redo(1)
(18) 552. Brown enviromental changes :dtt 030 min dtt-2(1)
(19) 564. Brown enviromental changes :1.5 mM diamide (90 min)(1)
(20) 612. Brown enviromental changes :YPD stationary phase 4 h ypd-1(1)
(21) 684. Expression in response to 0.8M NaCl for 10 min in wild type(1)
(22) DES460 + 0.02% MMS - 5 min
(23) DES460 + 0.02% MMS - 15 min
(24) 100 microM BCS 30 min

GCN4 --> ARG3 exp. conditions:26

(1) 89. Expression in response to 3-aminotriazole(1)
(2) 95. Expression in response to 50ug/mL FK506(1)
(3) 332. Rosetta 2000: Expression in cells with CMD1 under tet promoter(1)
(4) 387. Rosetta 2000: Expression in cells with ERG11 under tet promoter(1)
(5) 395. Rosetta 2000: Expression in response to 2-deoxy-D-glucose(1)
(6) 401. Rosetta 2000: Expression in response to HU(1)
(7) 402. Rosetta 2000: Expression in response to Itraconazole(1)
(8) 403. Rosetta 2000: Expression in response to Lovastatin(1)
(9) 406. Rosetta 2000: Expression in response to Terbinafine(1)
(10) 407. Rosetta 2000: Expression in response to Tunicamycin(1)

(11) 445. Expression in response to 0.1% MMS for 60 min (average of 3 experiments)(1)
(12) 446. Expression in response to 0.1% MMS for 10 min(1)
(13) 447. Expression in response to 0.1% MMS for 30 min(1)
(14) 448. Expression in response to 0.1% MMS for 60 min(1)
(15) 449. Expression in response to 0.1% MMS for 60 min(1)
(16) 462. Expression in response to 0.05% MMS for 60 min(1)
(17) 463. Expression in response to 0.1% MMS for 60 min(1)
(18) 479. Expression in diploid cells in response to rapamycin (100nM) for: 15min,30min,90min,120min(3)
(19) 533. Brown enviromental changes :1 mM Menadione (10 min)redo(1)
(20) 579. Brown enviromental changes :aa starv 1 h(1)
(21) 580. Brown enviromental changes :aa starv 2 h(1)
(22) 581. Brown enviromental changes :aa starv 4 h(1)
(23) 584. Brown enviromental changes :Nitrogen Depletion 1 h(1)
(24) 585. Brown enviromental changes :Nitrogen Depletion 2 h(1)
(25) 586. Brown enviromental changes :Nitrogen Depletion 4 h(1)
(26) DES460 + 0.02% MMS - 15 min

GCN4 --> ARG8 exp. conditions:26

(1) 89. Expression in response to 3-aminotriazole(1)
(2) 95. Expression in response to 50ug/mL FK506(1)
(3) 332. Rosetta 2000: Expression in cells with CMD1 under tet promoter(1)
(4) 387. Rosetta 2000: Expression in cells with ERG11 under tet promoter(1)
(5) 395. Rosetta 2000: Expression in response to 2-deoxy-D-glucose(1)
(6) 401. Rosetta 2000: Expression in response to HU(1)
(7) 402. Rosetta 2000: Expression in response to Itraconazole(1)
(8) 403. Rosetta 2000: Expression in response to Lovastatin(1)
(9) 406. Rosetta 2000: Expression in response to Terbinafine(1)
(10) 407. Rosetta 2000: Expression in response to Tunicamycin(1)
(11) 445. Expression in response to 0.1% MMS for 60 min (average of 3 experiments)(1)
(12) 446. Expression in response to 0.1% MMS for 10 min(1)
(13) 447. Expression in response to 0.1% MMS for 30 min(1)
(14) 448. Expression in response to 0.1% MMS for 60 min(1)
(15) 449. Expression in response to 0.1% MMS for 60 min(1)
(16) 462. Expression in response to 0.05% MMS for 60 min(1)
(17) 463. Expression in response to 0.1% MMS for 60 min(1)
(18) 479. Expression in diploid cells in response to rapamycin (100nM) for: 15min,30min,90min,120min(3)
(19) 533. Brown enviromental changes :1 mM Menadione (10 min)redo(1)
(20) 579. Brown enviromental changes :aa starv 1 h(1)
(21) 580. Brown enviromental changes :aa starv 2 h(1)
(22) 581. Brown enviromental changes :aa starv 4 h(1)
(23) 584. Brown enviromental changes :Nitrogen Depletion 1 h(1)
(24) 585. Brown enviromental changes :Nitrogen Depletion 2 h(1)
(25) 586. Brown enviromental changes :Nitrogen Depletion 4 h(1)
(26) DES460 + 0.02% MMS - 15 min

GCN4 -*-> ATR1 exp. conditions:39

(1) 5. Expression during the cell cycle (alpha factor arrest and release)(11)
(2) 7. Expression during the cell Cycle (cdc28)(10)
(3) 89. Expression in response to 3-aminotriazole(1)
(4) 95. Expression in response to 50ug/mL FK506(1)
(5) 332. Rosetta 2000: Expression in cells with CMD1 under tet promoter(1)
(6) 387. Rosetta 2000: Expression in cells with ERG11 under tet promoter(1)
(7) 395. Rosetta 2000: Expression in response to 2-deoxy-D-glucose(1)
(8) 401. Rosetta 2000: Expression in response to HU(1)
(9) 402. Rosetta 2000: Expression in response to Itraconazole(1)
(10) 403. Rosetta 2000: Expression in response to Lovastatin(1)
(11) 406. Rosetta 2000: Expression in response to Terbinafine(1)
(12) 407. Rosetta 2000: Expression in response to Tunicamycin(1)
(13) 445. Expression in response to 0.1% MMS for 60 min (average of 3 experiments)(1)
(14) 446. Expression in response to 0.1% MMS for 10 min(1)
(15) 447. Expression in response to 0.1% MMS for 30 min(1)
(16) 448. Expression in response to 0.1% MMS for 60 min(1)
(17) 449. Expression in response to 0.1% MMS for 60 min(1)
(18) 462. Expression in response to 0.05% MMS for 60 min(1)
(19) 463. Expression in response to 0.1% MMS for 60 min(1)
(20) 479. Expression in diploid cells in response to rapamycin (100nM) for: 15min,30min,90min,120min(2)
(21) 479. Expression in diploid cells in response to rapamycin (100nM) for: 15min,30min,90min,120min(3)
(22) 504. Brown enviromental changes :37C to 25C shock - 30 min(1)
(23) 506. Brown enviromental changes :37C to 25C shock - 60 min(1)
(24) 523. Brown enviromental changes :constant 0.32 mM H2O2 (10 min) redo(1)
(25) 533. Brown enviromental changes :1 mM Menadione (10 min)redo(1)
(26) 552. Brown enviromental changes :dtt 030 min dtt-2(1)
(27) 579. Brown enviromental changes :aa starv 1 h(1)
(28) 580. Brown enviromental changes :aa starv 2 h(1)
(29) 581. Brown enviromental changes :aa starv 4 h(1)
(30) 584. Brown enviromental changes :Nitrogen Depletion 1 h(1)
(31) 585. Brown enviromental changes :Nitrogen Depletion 2 h(1)
(32) 586. Brown enviromental changes :Nitrogen Depletion 4 h(1)
(33) 595. Brown enviromental changes :diauxic shift timecourse(1)
(34) 611. Brown enviromental changes :YPD stationary phase 2 h ypd-1(1)
(35) 612. Brown enviromental changes :YPD stationary phase 4 h ypd-1(1)
(36) 684. Expression in response to 0.8M NaCl for 10 min in wild type(1)
(37) DES460 + 0.02% MMS - 5 min
(38) DES460 + 0.02% MMS - 15 min
(39) 100 microM BCS 30 min

GCN4 -*-> FOL2 exp. conditions:39

(1) 5. Expression during the cell cycle (alpha factor arrest and release)(11)
(2) 7. Expression during the cell Cycle (cdc28)(10)
(3) 89. Expression in response to 3-aminotriazole(1)
(4) 95. Expression in response to 50ug/mL FK506(1)
(5) 332. Rosetta 2000: Expression in cells with CMD1 under tet promoter(1)
(6) 387. Rosetta 2000: Expression in cells with ERG11 under tet promoter(1)
(7) 395. Rosetta 2000: Expression in response to 2-deoxy-D-glucose(1)
(8) 401. Rosetta 2000: Expression in response to HU(1)
(9) 402. Rosetta 2000: Expression in response to Itraconazole(1)
(10) 403. Rosetta 2000: Expression in response to Lovastatin(1)
(11) 406. Rosetta 2000: Expression in response to Terbinafine(1)
(12) 407. Rosetta 2000: Expression in response to Tunicamycin(1)
(13) 445. Expression in response to 0.1% MMS for 60 min (average of 3 experiments)(1)
(14) 446. Expression in response to 0.1% MMS for 10 min(1)
(15) 447. Expression in response to 0.1% MMS for 30 min(1)
(16) 448. Expression in response to 0.1% MMS for 60 min(1)

(17) 449. Expression in response to 0.1% MMS for 60 min(1)
(18) 462. Expression in response to 0.05% MMS for 60 min(1)
(19) 463. Expression in response to 0.1% MMS for 60 min(1)
(20) 479. Expression in diploid cells in response to rapamycin (100nM) for: 15min,30min,90min,120min(2)
(21) 479. Expression in diploid cells in response to rapamycin (100nM) for: 15min,30min,90min,120min(3)
(22) 504. Brown enviromental changes :37C to 25C shock - 30 min(1)
(23) 506. Brown enviromental changes :37C to 25C shock - 60 min(1)
(24) 523. Brown enviromental changes :constant 0.32 mM H2O2 (10 min) redo(1)
(25) 533. Brown enviromental changes :1 mM Menadione (10 min)redo(1)
(26) 552. Brown enviromental changes :dtt 030 min dtt-2(1)
(27) 579. Brown enviromental changes :aa starv 1 h(1)
(28) 580. Brown enviromental changes :aa starv 2 h(1)
(29) 581. Brown enviromental changes :aa starv 4 h(1)
(30) 584. Brown enviromental changes :Nitrogen Depletion 1 h(1)
(31) 585. Brown enviromental changes :Nitrogen Depletion 2 h(1)
(32) 586. Brown enviromental changes :Nitrogen Depletion 4 h(1)
(33) 595. Brown enviromental changes :diauxic shift timecourse(1)
(34) 611. Brown enviromental changes :YPD stationary phase 2 h ypd-1(1)
(35) 612. Brown enviromental changes :YPD stationary phase 4 h ypd-1(1)
(36) 684. Expression in response to 0.8M NaCl for 10 min in wild type(1)
(37) DES460 + 0.02% MMS - 5 min
(38) DES460 + 0.02% MMS - 15 min
(39) 100 microM BCS 30 min

GCN4 -*-> IDP1 exp. conditions:34

(1) 8. Expression during the cell cycle (cell size selection and release)(3)
(2) 89. Expression in response to 3-aminotriazole(1)
(3) 95. Expression in response to 50ug/mL FK506(1)
(4) 332. Rosetta 2000: Expression in cells with CMD1 under tet promoter(1)
(5) 387. Rosetta 2000: Expression in cells with ERG11 under tet promoter(1)
(6) 395. Rosetta 2000: Expression in response to 2-deoxy-D-glucose(1)
(7) 401. Rosetta 2000: Expression in response to HU(1)
(8) 402. Rosetta 2000: Expression in response to Itraconazole(1)
(9) 403. Rosetta 2000: Expression in response to Lovastatin(1)
(10) 406. Rosetta 2000: Expression in response to Terbinafine(1)
(11) 407. Rosetta 2000: Expression in response to Tunicamycin(1)
(12) PHO81c vs WT exp2(1)
(13) 429. Expression in strain YHE711 (wild type) in response to 30 min 50 nM treatment with rapamycin in YPD(1)
(14) 445. Expression in response to 0.1% MMS for 60 min (average of 3 experiments)(1)
(15) 446. Expression in response to 0.1% MMS for 10 min(1)
(16) 447. Expression in response to 0.1% MMS for 30 min(1)
(17) 448. Expression in response to 0.1% MMS for 60 min(1)
(18) 449. Expression in response to 0.1% MMS for 60 min(1)
(19) 462. Expression in response to 0.05% MMS for 60 min(1)
(20) 463. Expression in response to 0.1% MMS for 60 min(1)
(21) 479. Expression in diploid cells in response to rapamycin (100nM) for: 15min,30min,90min,120min(2)
(22) 479. Expression in diploid cells in response to rapamycin (100nM) for: 15min,30min,90min,120min(3)
(23) 533. Brown enviromental changes :1 mM Menadione (10 min)redo(1)
(24) 579. Brown enviromental changes :aa starv 1 h(1)
(25) 580. Brown enviromental changes :aa starv 2 h(1)
(26) 581. Brown enviromental changes :aa starv 4 h(1)
(27) 582. Brown enviromental changes :aa starv 6 h(1)
(28) 584. Brown enviromental changes :Nitrogen Depletion 1 h(1)
(29) 585. Brown enviromental changes :Nitrogen Depletion 2 h(1)
(30) 586. Brown enviromental changes :Nitrogen Depletion 4 h(1)
(31) 612. Brown enviromental changes :YPD stationary phase 4 h ypd-1(1)
(32) 684. Expression in response to 0.8M NaCl for 10 min in wild type(1)
(33) DES460 + 0.02% MMS - 5 min
(34) DES460 + 0.02% MMS - 15 min

GCN4 -*-> ILV3 exp. conditions:26

(1) 89. Expression in response to 3-aminotriazole(1)
(2) 95. Expression in response to 50ug/mL FK506(1)
(3) 332. Rosetta 2000: Expression in cells with CMD1 under tet promoter(1)
(4) 387. Rosetta 2000: Expression in cells with ERG11 under tet promoter(1)
(5) 395. Rosetta 2000: Expression in response to 2-deoxy-D-glucose(1)
(6) 401. Rosetta 2000: Expression in response to HU(1)
(7) 402. Rosetta 2000: Expression in response to Itraconazole(1)
(8) 403. Rosetta 2000: Expression in response to Lovastatin(1)
(9) 406. Rosetta 2000: Expression in response to Terbinafine(1)
(10) 407. Rosetta 2000: Expression in response to Tunicamycin(1)
(11) 445. Expression in response to 0.1% MMS for 60 min (average of 3 experiments)(1)
(12) 446. Expression in response to 0.1% MMS for 10 min(1)
(13) 447. Expression in response to 0.1% MMS for 30 min(1)
(14) 448. Expression in response to 0.1% MMS for 60 min(1)
(15) 449. Expression in response to 0.1% MMS for 60 min(1)
(16) 462. Expression in response to 0.05% MMS for 60 min(1)
(17) 463. Expression in response to 0.1% MMS for 60 min(1)
(18) 479. Expression in diploid cells in response to rapamycin (100nM) for: 15min,30min,90min,120min(3)
(19) 533. Brown enviromental changes :1 mM Menadione (10 min)redo(1)
(20) 579. Brown enviromental changes :aa starv 1 h(1)
(21) 580. Brown enviromental changes :aa starv 2 h(1)
(22) 581. Brown enviromental changes :aa starv 4 h(1)
(23) 584. Brown enviromental changes :Nitrogen Depletion 1 h(1)
(24) 585. Brown enviromental changes :Nitrogen Depletion 2 h(1)
(25) 586. Brown enviromental changes :Nitrogen Depletion 4 h(1)
(26) DES460 + 0.02% MMS - 15 min

GCN4 --> LEU3 exp. conditions:25

(1) 6. Expression during the cell cycle (cdc15 arrest and release)(15)
(2) 6. Expression during the cell cycle (cdc15 arrest and release)(20)
(3) 7. Expression during the cell Cycle (cdc28)(10)
(4) 8. Expression during the cell cycle (cell size selection and release)(4)
(5) 8. Expression during the cell cycle (cell size selection and release)(5)
(6) 89. Expression in response to 3-aminotriazole(1)
(7) 394. Rosetta 2000: Expression in cells with YEF3 under tet promoter(1)
(8) 402. Rosetta 2000: Expression in response to Itraconazole(1)
(9) 403. Rosetta 2000: Expression in response to Lovastatin(1)
(10) 406. Rosetta 2000: Expression in response to Terbinafine(1)
(11) 407. Rosetta 2000: Expression in response to Tunicamycin(1)
(12) 446. Expression in response to 0.1% MMS for 10 min(1)
(13) 481. Expression in response to heat shock: 15,30,45,60,120 min(2)
(14) 481. Expression in response to heat shock: 15,30,45,60,120 min(4)

(15) 497. Brown enviromental changes :Heat Shock 000 minutes hs-2(1)
(16) 504. Brown enviromental changes :37C to 25C shock - 30 min(1)
(17) 551. Brown enviromental changes :dtt 015 min dtt-2(1)
(18) 556. Brown enviromental changes :dtt 480 min dtt-2(1)
(19) 575. Brown enviromental changes :Hypo-osmotic shock - 45 min(1)
(20) 581. Brown enviromental changes :aa starv 4 h(1)
(21) 681. Expression in response to 0.4M NaCl for 10 min in wild type(1)
(22) 684. Expression in response to 0.8M NaCl for 10 min in wild type(1)
(23) DES460 (wt) - mock irradiation - 30 min
(24) MHY1 (crt1) vs CRY1 (wild type)
(25) 100 microM BCS 30 min

GCN4 --> MET4 exp. conditions:28

(1) 6. Expression during the cell cycle (cdc15 arrest and release)(15)
(2) 11. Expression during diauxic shift: 9h,11h,13h,15h,17h,19h,21h(3)
(3) 95. Expression in response to 50ug/mL FK506(1)
(4) 387. Rosetta 2000: Expression in cells with ERG11 under tet promoter(1)
(5) 402. Rosetta 2000: Expression in response to Itraconazole(1)
(6) 403. Rosetta 2000: Expression in response to Lovastatin(1)
(7) 406. Rosetta 2000: Expression in response to Terbinafine(1)
(8) 407. Rosetta 2000: Expression in response to Tunicamycin(1)
(9) 479. Expression in diploid cells in response to rapamycin (100nM) for: 15min,30min,90min,120min(2)
(10) 479. Expression in diploid cells in response to rapamycin (100nM) for: 15min,30min,90min,120min(3)
(11) 479. Expression in diploid cells in response to rapamycin (100nM) for: 15min,30min,90min,120min(4)
(12) 481. Expression in response to heat shock: 15,30,45,60,120 min(2)
(13) 481. Expression in response to heat shock: 15,30,45,60,120 min(4)
(14) 575. Brown enviromental changes :Hypo-osmotic shock - 45 min(1)
(15) 581. Brown enviromental changes :aa starv 4 h(1)
(16) 582. Brown enviromental changes :aa starv 6 h(1)
(17) 586. Brown enviromental changes :Nitrogen Depletion 4 h(1)
(18) 588. Brown enviromental changes :Nitrogen Depletion 12 h(1)
(19) 589. Brown enviromental changes :Nitrogen Depletion 1 d(1)
(20) 590. Brown enviromental changes :Nitrogen Depletion 2 d(1)
(21) 595. Brown enviromental changes :diauxic shift timecourse(1)
(22) 601. Brown enviromental changes :YPD 2 h ypd-2(1)
(23) 602. Brown enviromental changes :YPD 4 h ypd-2(1)
(24) 611. Brown enviromental changes :YPD stationary phase 2 h ypd-1(1)
(25) 612. Brown enviromental changes :YPD stationary phase 4 h ypd-1(1)
(26) 681. Expression in response to 0.4M NaCl for 10 min in wild type(1)
(27) DES460 (wt) - mock irradiation - 30 min
(28) 100 microM BCS 30 min

GCN4 -*-> UGA3 exp. conditions:26

(1) 89. Expression in response to 3-aminotriazole(1)
(2) 95. Expression in response to 50ug/mL FK506(1)
(3) 332. Rosetta 2000: Expression in cells with CMD1 under tet promoter(1)
(4) 387. Rosetta 2000: Expression in cells with ERG11 under tet promoter(1)
(5) 395. Rosetta 2000: Expression in response to 2-deoxy-D-glucose(1)
(6) 401. Rosetta 2000: Expression in response to HU(1)
(7) 402. Rosetta 2000: Expression in response to Itraconazole(1)
(8) 403. Rosetta 2000: Expression in response to Lovastatin(1)
(9) 406. Rosetta 2000: Expression in response to Terbinafine(1)
(10) 407. Rosetta 2000: Expression in response to Tunicamycin(1)
(11) 445. Expression in response to 0.1% MMS for 60 min (average of 3 experiments)(1)
(12) 446. Expression in response to 0.1% MMS for 10 min(1)
(13) 447. Expression in response to 0.1% MMS for 30 min(1)
(14) 448. Expression in response to 0.1% MMS for 60 min(1)
(15) 449. Expression in response to 0.1% MMS for 60 min(1)
(16) 462. Expression in response to 0.05% MMS for 60 min(1)
(17) 463. Expression in response to 0.1% MMS for 60 min(1)
(18) 479. Expression in diploid cells in response to rapamycin (100nM) for: 15min,30min,90min,120min(3)
(19) 533. Brown enviromental changes :1 mM Menadione (10 min)redo(1)
(20) 579. Brown enviromental changes :aa starv 1 h(1)
(21) 580. Brown enviromental changes :aa starv 2 h(1)
(22) 581. Brown enviromental changes :aa starv 4 h(1)
(23) 584. Brown enviromental changes :Nitrogen Depletion 1 h(1)
(24) 585. Brown enviromental changes :Nitrogen Depletion 2 h(1)
(25) 586. Brown enviromental changes :Nitrogen Depletion 4 h(1)
(26) DES460 + 0.02% MMS - 15 min

GLN3 -*-> ARG1 exp. conditions:22

(1) 6. Expression during the cell cycle (cdc15 arrest and release)(22)
(2) 6. Expression during the cell cycle (cdc15 arrest and release)(23)
(3) 89. Expression in response to 3-aminotriazole(1)
(4) 95. Expression in response to 50ug/mL FK506(1)
(5) 387. Rosetta 2000: Expression in cells with ERG11 under tet promoter(1)
(6) 402. Rosetta 2000: Expression in response to Itraconazole(1)
(7) 429. Expression in strain YHE711 (wild type) in response to 30 min 50 nM treatment with rapamycin in YPD(1)
(8) 439. Expression in strain Jk9-3da (wild type) in response to 30 min 50 nM treatment with rapamycin in YPD(1)
(9) 479. Expression in diploid cells in response to rapamycin (100nM) for: 15min,30min,90min,120min(1)
(10) 487. Expression in response to sorbitol: 15 30 45 90 120 min(3)
(11) 572. Brown enviromental changes :Hypo-osmotic shock - 5 min(1)
(12) 579. Brown enviromental changes :aa starv 1 h(1)
(13) 580. Brown enviromental changes :aa starv 2 h(1)
(14) 581. Brown enviromental changes :aa starv 4 h(1)
(15) 582. Brown enviromental changes :aa starv 6 h(1)
(16) 583. Brown enviromental changes :Nitrogen Depletion 30 min.(1)
(17) 584. Brown enviromental changes :Nitrogen Depletion 1 h(1)
(18) 585. Brown enviromental changes :Nitrogen Depletion 2 h(1)
(19) 586. Brown enviromental changes :Nitrogen Depletion 4 h(1)
(20) 670. Expression in response to antimycin 60min(1)
(21) 671. Expression in response to antimycin 120min(1)
(22) 672. Expression in response to carbonyl cyanide m-chlorophenylhydrazone (CCCP) 90min(1)

GLN3 --> DAL1 exp. conditions:31

(1) 7. Expression during the cell Cycle (cdc28)(6)
(2) 89. Expression in response to 3-aminotriazole(1)
(3) 95. Expression in response to 50ug/mL FK506(1)
(4) 387. Rosetta 2000: Expression in cells with ERG11 under tet promoter(1)
(5) 392. Rosetta 2000: Expression in cells with PMA1 under tet promoter(1)

(6) 401. Rosetta 2000: Expression in response to HU(1)
(7) 402. Rosetta 2000: Expression in response to Itraconazole(1)
(8) 428. Expression in strain PM38 (wild type) in response to 30 min 50 nM treatment with rapamycin in YPD(1)
(9) 429. Expression in strain YHE711 (wild type) in response to 30 min 50 nM treatment with rapamycin in YPD(1)
(10) 439. Expression in strain Jk9-3da (wild type) in response to 30 min 50 nM treatment with rapamycin in YPD(1)
(11) 442. Expression in strain PM38 (wild type) in response to 30 min 50 nM treatment with rapamycin in YPD(1)
(12) 479. Expression in diploid cells in response to rapamycin (100nM) for: 15min,30min,90min,120min(1)
(13) 479. Expression in diploid cells in response to rapamycin (100nM) for: 15min,30min,90min,120min(2)
(14) 495. Brown enviromental changes :Heat Shock 80 minutes hs-1(1)
(15) 570. Brown enviromental changes :1M sorbitol - 90 min(1)
(16) 578. Brown enviromental changes :aa starv 0.5 h(1)
(17) 579. Brown enviromental changes :aa starv 1 h(1)
(18) 580. Brown enviromental changes :aa starv 2 h(1)
(19) 581. Brown enviromental changes :aa starv 4 h(1)
(20) 582. Brown enviromental changes :aa starv 6 h(1)
(21) 583. Brown enviromental changes :Nitrogen Depletion 30 min.(1)
(22) 584. Brown enviromental changes :Nitrogen Depletion 1 h(1)
(23) 585. Brown enviromental changes :Nitrogen Depletion 2 h(1)
(24) 586. Brown enviromental changes :Nitrogen Depletion 4 h(1)
(25) 587. Brown enviromental changes :Nitrogen Depletion 8 h(1)
(26) 588. Brown enviromental changes :Nitrogen Depletion 12 h(1)
(27) 589. Brown enviromental changes :Nitrogen Depletion 1 d(1)
(28) 590. Brown enviromental changes :Nitrogen Depletion 2 d(1)
(29) 591. Brown enviromental changes :Nitrogen Depletion 3 d(1)
(30) 592. Brown enviromental changes :Nitrogen Depletion 5 d(1)
(31) 671. Expression in response to antimycin 120min(1)

GLN3 --> DAL2 exp. conditions:34

(1) 5. Expression during the cell cycle (alpha factor arrest and release)(11)
(2) 89. Expression in response to 3-aminotriazole(1)
(3) 95. Expression in response to 50ug/mL FK506(1)
(4) 387. Rosetta 2000: Expression in cells with ERG11 under tet promoter(1)
(5) 402. Rosetta 2000: Expression in response to Itraconazole(1)
(6) 406. Rosetta 2000: Expression in response to Terbinafine(1)
(7) 407. Rosetta 2000: Expression in response to Tunicamycin(1)
(8) 428. Expression in strain PM38 (wild type) in response to 30 min 50 nM treatment with rapamycin in YPD(1)
(9) 429. Expression in strain YHE711 (wild type) in response to 30 min 50 nM treatment with rapamycin in YPD(1)
(10) 439. Expression in strain Jk9-3da (wild type) in response to 30 min 50 nM treatment with rapamycin in YPD(1)
(11) 442. Expression in strain PM38 (wild type) in response to 30 min 50 nM treatment with rapamycin in YPD(1)
(12) 477. Expression in response to trichostatin A (TSA): 15min,30min,60min,120min(1)
(13) 477. Expression in response to trichostatin A (TSA): 15min,30min,60min,120min(3)
(14) 479. Expression in diploid cells in response to rapamycin (100nM) for: 15min,30min,90min,120min(2)
(15) 479. Expression in diploid cells in response to rapamycin (100nM) for: 15min,30min,90min,120min(4)
(16) 578. Brown enviromental changes :aa starv 0.5 h(1)
(17) 579. Brown enviromental changes :aa starv 1 h(1)
(18) 580. Brown enviromental changes :aa starv 2 h(1)
(19) 581. Brown enviromental changes :aa starv 4 h(1)
(20) 582. Brown enviromental changes :aa starv 6 h(1)
(21) 583. Brown enviromental changes :Nitrogen Depletion 30 min.(1)
(22) 584. Brown enviromental changes :Nitrogen Depletion 1 h(1)
(23) 585. Brown enviromental changes :Nitrogen Depletion 2 h(1)
(24) 586. Brown enviromental changes :Nitrogen Depletion 4 h(1)
(25) 587. Brown enviromental changes :Nitrogen Depletion 8 h(1)
(26) 588. Brown enviromental changes :Nitrogen Depletion 12 h(1)
(27) 589. Brown enviromental changes :Nitrogen Depletion 1 d(1)
(28) 590. Brown enviromental changes :Nitrogen Depletion 2 d(1)
(29) 591. Brown enviromental changes :Nitrogen Depletion 3 d(1)
(30) 592. Brown enviromental changes :Nitrogen Depletion 5 d(1)
(31) 670. Expression in response to antimycin 60min(1)
(32) 671. Expression in response to antimycin 120min(1)
(33) 675. Expression in response to propionate(1)
(34) DES460 (wt) - mock irradiation - 30 min

GLN3 --> DAL3 exp. conditions:34

(1) 5. Expression during the cell cycle (alpha factor arrest and release)(11)
(2) 89. Expression in response to 3-aminotriazole(1)
(3) 95. Expression in response to 50ug/mL FK506(1)
(4) 387. Rosetta 2000: Expression in cells with ERG11 under tet promoter(1)
(5) 402. Rosetta 2000: Expression in response to Itraconazole(1)
(6) 406. Rosetta 2000: Expression in response to Terbinafine(1)
(7) 407. Rosetta 2000: Expression in response to Tunicamycin(1)
(8) 428. Expression in strain PM38 (wild type) in response to 30 min 50 nM treatment with rapamycin in YPD(1)
(9) 429. Expression in strain YHE711 (wild type) in response to 30 min 50 nM treatment with rapamycin in YPD(1)
(10) 439. Expression in strain Jk9-3da (wild type) in response to 30 min 50 nM treatment with rapamycin in YPD(1)
(11) 442. Expression in strain PM38 (wild type) in response to 30 min 50 nM treatment with rapamycin in YPD(1)
(12) 477. Expression in response to trichostatin A (TSA): 15min,30min,60min,120min(1)
(13) 477. Expression in response to trichostatin A (TSA): 15min,30min,60min,120min(3)
(14) 479. Expression in diploid cells in response to rapamycin (100nM) for: 15min,30min,90min,120min(2)
(15) 479. Expression in diploid cells in response to rapamycin (100nM) for: 15min,30min,90min,120min(4)
(16) 578. Brown enviromental changes :aa starv 0.5 h(1)
(17) 579. Brown enviromental changes :aa starv 1 h(1)
(18) 580. Brown enviromental changes :aa starv 2 h(1)
(19) 581. Brown enviromental changes :aa starv 4 h(1)
(20) 582. Brown enviromental changes :aa starv 6 h(1)
(21) 583. Brown enviromental changes :Nitrogen Depletion 30 min.(1)
(22) 584. Brown enviromental changes :Nitrogen Depletion 1 h(1)
(23) 585. Brown enviromental changes :Nitrogen Depletion 2 h(1)
(24) 586. Brown enviromental changes :Nitrogen Depletion 4 h(1)
(25) 587. Brown enviromental changes :Nitrogen Depletion 8 h(1)
(26) 588. Brown enviromental changes :Nitrogen Depletion 12 h(1)
(27) 589. Brown enviromental changes :Nitrogen Depletion 1 d(1)
(28) 590. Brown enviromental changes :Nitrogen Depletion 2 d(1)
(29) 591. Brown enviromental changes :Nitrogen Depletion 3 d(1)
(30) 592. Brown enviromental changes :Nitrogen Depletion 5 d(1)
(31) 670. Expression in response to antimycin 60min(1)
(32) 671. Expression in response to antimycin 120min(1)
(33) 675. Expression in response to propionate(1)
(34) DES460 (wt) - mock irradiation - 30 min

GLN3 --> DAL4 exp. conditions:29

(1) 49. Expression in response to 50 nM alpha-factor: 0,15,30,45,60,90,120 min(5)
(2) 89. Expression in response to 3-aminotriazole(1)
(3) 95. Expression in response to 50ug/mL FK506(1)

(4) 407. Rosetta 2000: Expression in response to Tunicamycin(1)
(5) 428. Expression in strain PM38 (wild type) in response to 30 min 50 nM treatment with rapamycin in YPD(1)
(6) 429. Expression in strain YHE711 (wild type) in response to 30 min 50 nM treatment with rapamycin in YPD(1)
(7) 439. Expression in strain Jk9-3da (wild type) in response to 30 min 50 nM treatment with rapamycin in YPD(1)
(8) 442. Expression in strain PM38 (wild type) in response to 30 min 50 nM treatment with rapamycin in YPD(1)
(9) 479. Expression in diploid cells in response to rapamycin (100nM) for: 15min,30min,90min,120min(1)
(10) 479. Expression in diploid cells in response to rapamycin (100nM) for: 15min,30min,90min,120min(2)
(11) 578. Brown enviromental changes :aa starv 0.5 h(1)
(12) 579. Brown enviromental changes :aa starv 1 h(1)
(13) 580. Brown enviromental changes :aa starv 2 h(1)
(14) 581. Brown enviromental changes :aa starv 4 h(1)
(15) 582. Brown enviromental changes :aa starv 6 h(1)
(16) 583. Brown enviromental changes :Nitrogen Depletion 30 min.(1)
(17) 584. Brown enviromental changes :Nitrogen Depletion 1 h(1)
(18) 585. Brown enviromental changes :Nitrogen Depletion 2 h(1)
(19) 586. Brown enviromental changes :Nitrogen Depletion 4 h(1)
(20) 587. Brown enviromental changes :Nitrogen Depletion 8 h(1)
(21) 588. Brown enviromental changes :Nitrogen Depletion 12 h(1)
(22) 589. Brown enviromental changes :Nitrogen Depletion 1 d(1)
(23) 590. Brown enviromental changes :Nitrogen Depletion 2 d(1)
(24) 591. Brown enviromental changes :Nitrogen Depletion 3 d(1)
(25) 592. Brown enviromental changes :Nitrogen Depletion 5 d(1)
(26) 670. Expression in response to antimycin 60min(1)
(27) 671. Expression in response to antimycin 120min(1)
(28) DES460 (wt) - mock irradiation - 30 min
(29) MAC1-up (B)

GLN3 -*-> UGA3 exp. conditions:27

(1) 5. Expression during the cell cycle (alpha factor arrest and release)(16)
(2) 6. Expression during the cell cycle (cdc15 arrest and release)(8)
(3) 6. Expression during the cell cycle (cdc15 arrest and release)(10)
(4) 7. Expression during the cell Cycle (cdc28)(10)
(5) 11. Expression during diauxic shift: 9h,11h,13h,15h,17h,19h,21h(2)
(6) 89. Expression in response to 3-aminotriazole(1)
(7) 95. Expression in response to 50ug/mL FK506(1)
(8) 387. Rosetta 2000: Expression in cells with ERG11 under tet promoter(1)
(9) 395. Rosetta 2000: Expression in response to 2-deoxy-D-glucose(1)
(10) 402. Rosetta 2000: Expression in response to Itraconazole(1)
(11) 403. Rosetta 2000: Expression in response to Lovastatin(1)
(12) 406. Rosetta 2000: Expression in response to Terbinafine(1)
(13) 407. Rosetta 2000: Expression in response to Tunicamycin(1)
(14) 579. Brown enviromental changes :aa starv 1 h(1)
(15) 580. Brown enviromental changes :aa starv 2 h(1)
(16) 581. Brown enviromental changes :aa starv 4 h(1)
(17) 585. Brown enviromental changes :Nitrogen Depletion 2 h(1)
(18) 586. Brown enviromental changes :Nitrogen Depletion 4 h(1)
(19) 588. Brown enviromental changes :Nitrogen Depletion 12 h(1)
(20) 591. Brown enviromental changes :Nitrogen Depletion 3 d(1)
(21) 594. Brown enviromental changes :diauxic shift timecourse(1)
(22) 670. Expression in response to antimycin 60min(1)
(23) 671. Expression in response to antimycin 120min(1)
(24) 672. Expression in response to carbonyl cyanide m-chlorophenylhydrazone (CCCP) 90min(1)
(25) 675. Expression in response to propionate(1)
(26) DES460 (wt) - mock irradiation - 30 min
(27) DES460 (wt) - mock irradiation - 90 min

GLN3 -*-> YHR029C exp. conditions:32

(1) 5. Expression during the cell cycle (alpha factor arrest and release)(17)
(2) 89. Expression in response to 3-aminotriazole(1)
(3) 95. Expression in response to 50ug/mL FK506(1)
(4) 387. Rosetta 2000: Expression in cells with ERG11 under tet promoter(1)
(5) 402. Rosetta 2000: Expression in response to Itraconazole(1)
(6) 406. Rosetta 2000: Expression in response to Terbinafine(1)
(7) 407. Rosetta 2000: Expression in response to Tunicamycin(1)
(8) 445. Expression in response to 0.1% MMS for 60 min (average of 3 experiments)(1)
(9) 449. Expression in response to 0.1% MMS for 60 min(1)
(10) 451. Expression in response to BCNU (200 micromolar) for 60 min(1)
(11) 455. Expression in response to high MNNG (27 microgram/ml) for 60 min(1)
(12) 456. Expression in response to high 4NQO (8 microgram/ml) for 60 min(1)
(13) 464. Expression in response to 0.2% MMS for 60 min(1)
(14) 479. Expression in diploid cells in response to rapamycin (100nM) for: 15min,30min,90min,120min(1)
(15) 479. Expression in diploid cells in response to rapamycin (100nM) for: 15min,30min,90min,120min(2)
(16) 479. Expression in diploid cells in response to rapamycin (100nM) for: 15min,30min,90min,120min(3)
(17) 579. Brown enviromental changes :aa starv 1 h(1)
(18) 581. Brown enviromental changes :aa starv 4 h(1)
(19) 582. Brown enviromental changes :aa starv 6 h(1)
(20) 584. Brown enviromental changes :Nitrogen Depletion 1 h(1)
(21) 585. Brown enviromental changes :Nitrogen Depletion 2 h(1)
(22) 586. Brown enviromental changes :Nitrogen Depletion 4 h(1)
(23) 587. Brown enviromental changes :Nitrogen Depletion 8 h(1)
(24) 588. Brown enviromental changes :Nitrogen Depletion 12 h(1)
(25) 589. Brown enviromental changes :Nitrogen Depletion 1 d(1)
(26) 590. Brown enviromental changes :Nitrogen Depletion 2 d(1)
(27) 591. Brown enviromental changes :Nitrogen Depletion 3 d(1)
(28) 592. Brown enviromental changes :Nitrogen Depletion 5 d(1)
(29) 670. Expression in response to antimycin 60min(1)
(30) 671. Expression in response to antimycin 120min(1)
(31) 672. Expression in response to carbonyl cyanide m-chlorophenylhydrazone (CCCP) 90min(1)
(32) DES460 (wt) - mock irradiation - 30 min

HAP1 -*-| COR1 exp. conditions:24

(1) 7. Expression during the cell Cycle (cdc28)(16)
(2) 7. Expression during the cell Cycle (cdc28)(17)
(3) 8. Expression during the cell cycle (cell size selection and release)(8)
(4) 11. Expression during diauxic shift: 9h,11h,13h,15h,17h,19h,21h(7)
(5) 386. Rosetta 2000: Expression in cells with CDC42 under tet promoter(1)
(6) 388. Rosetta 2000: Expression in cells with FKS1 under tet promoter(1)
(7) 390. Rosetta 2000: Expression in cells with IDI1 under tet promoter(1)
(8) 450. Expression in response to low MNNG (8 microgram/ml) for 60 min(1)
(9) 527. Brown enviromental changes :constant 0.32 mM H2O2 (50 min) redo(1)
(10) 530. Brown enviromental changes :constant 0.32 mM H2O2 (100 min) redo(1)
(11) 531. Brown enviromental changes :constant 0.32 mM H2O2 (120 min) redo(1)
(12) 532. Brown enviromental changes :constant 0.32 mM H2O2 (160 min) redo(1)

(13) 556. Brown enviromental changes :dtt 480 min dtt-2(1)
(14) 557. Brown enviromental changes :1.5 mM diamide (5 min)(1)
(15) 558. Brown enviromental changes :1.5 mM diamide (10 min)(1)
(16) 599. Brown enviromental changes :diauxic shift timecourse(1)
(17) 600. Brown enviromental changes :diauxic shift timecourse(1)
(18) 602. Brown enviromental changes :YPD 4 h ypd-2(1)
(19) 604. Brown enviromental changes :YPD 8 h ypd-2(1)
(20) 605. Brown enviromental changes :YPD 10 h ypd-2(1)
(21) 606. Brown enviromental changes :YPD 12 h ypd-2(1)
(22) 613. Brown enviromental changes :YPD stationary phase 8 h ypd-1(1)
(23) 614. Brown enviromental changes :YPD stationary phase 12 h ypd-1(1)
(24) 615. Brown enviromental changes :YPD stationary phase 1 d ypd-1(1)

HAP1 --> CYC1 exp. conditions:24

(1) 7. Expression during the cell Cycle (cdc28)(16)
(2) 7. Expression during the cell Cycle (cdc28)(17)
(3) 8. Expression during the cell cycle (cell size selection and release)(8)
(4) 11. Expression during diauxic shift: 9h,11h,13h,15h,17h,19h,21h(7)
(5) 386. Rosetta 2000: Expression in cells with CDC42 under tet promoter(1)
(6) 388. Rosetta 2000: Expression in cells with FKS1 under tet promoter(1)
(7) 390. Rosetta 2000: Expression in cells with IDI1 under tet promoter(1)
(8) 450. Expression in response to low MNNG (8 microgram/ml) for 60 min(1)
(9) 527. Brown enviromental changes :constant 0.32 mM H2O2 (50 min) redo(1)
(10) 530. Brown enviromental changes :constant 0.32 mM H2O2 (100 min) redo(1)
(11) 531. Brown enviromental changes :constant 0.32 mM H2O2 (120 min) redo(1)
(12) 532. Brown enviromental changes :constant 0.32 mM H2O2 (160 min) redo(1)
(13) 556. Brown enviromental changes :dtt 480 min dtt-2(1)
(14) 557. Brown enviromental changes :1.5 mM diamide (5 min)(1)
(15) 558. Brown enviromental changes :1.5 mM diamide (10 min)(1)
(16) 599. Brown enviromental changes :diauxic shift timecourse(1)
(17) 600. Brown enviromental changes :diauxic shift timecourse(1)
(18) 602. Brown enviromental changes :YPD 4 h ypd-2(1)
(19) 604. Brown enviromental changes :YPD 8 h ypd-2(1)
(20) 605. Brown enviromental changes :YPD 10 h ypd-2(1)
(21) 606. Brown enviromental changes :YPD 12 h ypd-2(1)
(22) 613. Brown enviromental changes :YPD stationary phase 8 h ypd-1(1)
(23) 614. Brown enviromental changes :YPD stationary phase 12 h ypd-1(1)
(24) 615. Brown enviromental changes :YPD stationary phase 1 d ypd-1(1)

HAP2 --> COX4 exp. conditions:22

(1) 11. Expression during diauxic shift: 9h,11h,13h,15h,17h,19h,21h(5)
(2) 11. Expression during diauxic shift: 9h,11h,13h,15h,17h,19h,21h(6)
(3) 11. Expression during diauxic shift: 9h,11h,13h,15h,17h,19h,21h(7)
(4) 390. Rosetta 2000: Expression in cells with IDI1 under tet promoter(1)
(5) 428. Expression in strain PM38 (wild type) in response to 30 min 50 nM treatment with rapamycin in YPD(1)
(6) 479. Expression in diploid cells in response to rapamycin (100nM) for: 15min,30min,90min,120min(3)
(7) 597. Brown enviromental changes :diauxic shift timecourse(1)
(8) 598. Brown enviromental changes :diauxic shift timecourse(1)
(9) 599. Brown enviromental changes :diauxic shift timecourse(1)
(10) 600. Brown enviromental changes :diauxic shift timecourse(1)
(11) 602. Brown enviromental changes :YPD 4 h ypd-2(1)
(12) 603. Brown enviromental changes :YPD 6 h ypd-2(1)
(13) 604. Brown enviromental changes :YPD 8 h ypd-2(1)
(14) 605. Brown enviromental changes :YPD 10 h ypd-2(1)
(15) 606. Brown enviromental changes :YPD 12 h ypd-2(1)
(16) 608. Brown enviromental changes :YPD 2 d ypd-2(1)
(17) 612. Brown enviromental changes :YPD stationary phase 4 h ypd-1(1)
(18) 613. Brown enviromental changes :YPD stationary phase 8 h ypd-1(1)
(19) 614. Brown enviromental changes :YPD stationary phase 12 h ypd-1(1)
(20) 615. Brown enviromental changes :YPD stationary phase 1 d ypd-1(1)
(21) 616. Brown enviromental changes :YPD stationary phase 2 d ypd-1(1)
(22) 617. Brown enviromental changes :YPD stationary phase 3 d ypd-1(1)

HAP2 --> COX6 exp. conditions:22

(1) 11. Expression during diauxic shift: 9h,11h,13h,15h,17h,19h,21h(5)
(2) 11. Expression during diauxic shift: 9h,11h,13h,15h,17h,19h,21h(6)
(3) 11. Expression during diauxic shift: 9h,11h,13h,15h,17h,19h,21h(7)
(4) 390. Rosetta 2000: Expression in cells with IDI1 under tet promoter(1)
(5) 428. Expression in strain PM38 (wild type) in response to 30 min 50 nM treatment with rapamycin in YPD(1)
(6) 479. Expression in diploid cells in response to rapamycin (100nM) for: 15min,30min,90min,120min(3)
(7) 597. Brown enviromental changes :diauxic shift timecourse(1)
(8) 598. Brown enviromental changes :diauxic shift timecourse(1)
(9) 599. Brown enviromental changes :diauxic shift timecourse(1)
(10) 600. Brown enviromental changes :diauxic shift timecourse(1)
(11) 602. Brown enviromental changes :YPD 4 h ypd-2(1)
(12) 603. Brown enviromental changes :YPD 6 h ypd-2(1)
(13) 604. Brown enviromental changes :YPD 8 h ypd-2(1)
(14) 605. Brown enviromental changes :YPD 10 h ypd-2(1)
(15) 606. Brown enviromental changes :YPD 12 h ypd-2(1)
(16) 608. Brown enviromental changes :YPD 2 d ypd-2(1)
(17) 612. Brown enviromental changes :YPD stationary phase 4 h ypd-1(1)
(18) 613. Brown enviromental changes :YPD stationary phase 8 h ypd-1(1)
(19) 614. Brown enviromental changes :YPD stationary phase 12 h ypd-1(1)
(20) 615. Brown enviromental changes :YPD stationary phase 1 d ypd-1(1)
(21) 616. Brown enviromental changes :YPD stationary phase 2 d ypd-1(1)
(22) 617. Brown enviromental changes :YPD stationary phase 3 d ypd-1(1)

HAP2 -*-> QCR7 exp. conditions:22

(1) 11. Expression during diauxic shift: 9h,11h,13h,15h,17h,19h,21h(5)
(2) 11. Expression during diauxic shift: 9h,11h,13h,15h,17h,19h,21h(6)
(3) 11. Expression during diauxic shift: 9h,11h,13h,15h,17h,19h,21h(7)
(4) 390. Rosetta 2000: Expression in cells with IDI1 under tet promoter(1)
(5) 428. Expression in strain PM38 (wild type) in response to 30 min 50 nM treatment with rapamycin in YPD(1)
(6) 479. Expression in diploid cells in response to rapamycin (100nM) for: 15min,30min,90min,120min(3)
(7) 597. Brown enviromental changes :diauxic shift timecourse(1)
(8) 598. Brown enviromental changes :diauxic shift timecourse(1)
(9) 599. Brown enviromental changes :diauxic shift timecourse(1)
(10) 600. Brown enviromental changes :diauxic shift timecourse(1)
(11) 602. Brown enviromental changes :YPD 4 h ypd-2(1)
(12) 603. Brown enviromental changes :YPD 6 h ypd-2(1)

(13) 604. Brown enviromental changes :YPD 8 h ypd-2(1)
(14) 605. Brown enviromental changes :YPD 10 h ypd-2(1)
(15) 606. Brown enviromental changes :YPD 12 h ypd-2(1)
(16) 608. Brown enviromental changes :YPD 2 d ypd-2(1)
(17) 612. Brown enviromental changes :YPD stationary phase 4 h ypd-1(1)
(18) 613. Brown enviromental changes :YPD stationary phase 8 h ypd-1(1)
(19) 614. Brown enviromental changes :YPD stationary phase 12 h ypd-1(1)
(20) 615. Brown enviromental changes :YPD stationary phase 1 d ypd-1(1)
(21) 616. Brown enviromental changes :YPD stationary phase 2 d ypd-1(1)
(22) 617. Brown enviromental changes :YPD stationary phase 3 d ypd-1(1)

HAP4 -*-> ATP1 exp. conditions:43

(1) 6. Expression during the cell cycle (cdc15 arrest and release)(15)
(2) 7. Expression during the cell Cycle (cdc28)(10)
(3) 7. Expression during the cell Cycle (cdc28)(15)
(4) 7. Expression during the cell Cycle (cdc28)(17)
(5) 11. Expression during diauxic shift: 9h,11h,13h,15h,17h,19h,21h(1)
(6) 11. Expression during diauxic shift: 9h,11h,13h,15h,17h,19h,21h(2)
(7) 11. Expression during diauxic shift: 9h,11h,13h,15h,17h,19h,21h(3)
(8) 11. Expression during diauxic shift: 9h,11h,13h,15h,17h,19h,21h(5)
(9) 11. Expression during diauxic shift: 9h,11h,13h,15h,17h,19h,21h(6)
(10) 11. Expression during diauxic shift: 9h,11h,13h,15h,17h,19h,21h(7)
(11) 390. Rosetta 2000: Expression in cells with IDI1 under tet promoter(1)
(12) 393. Rosetta 2000: Expression in cells with RHO1 under tet promoter(1)
(13) 428. Expression in strain PM38 (wild type) in response to 30 min 50 nM treatment with rapamycin in YPD(1)
(14) 479. Expression in diploid cells in response to rapamycin (100nM) for: 15min,30min,90min,120min(3)
(15) 482. Expression in response to acid: 10,20,40,60,80,100 min(1)
(16) 482. Expression in response to acid: 10,20,40,60,80,100 min(5)
(17) 488. Brown enviromental changes :Heat Shock 05 minutes hs-1(1)
(18) 517. Brown enviromental changes :29C +1M sorbitol to 33C + 1M sorbitol - 5 minutes(1)
(19) 532. Brown enviromental changes :constant 0.32 mM H2O2 (160 min) redo(1)
(20) 556. Brown enviromental changes :dtt 480 min dtt-2(1)
(21) 558. Brown enviromental changes :1.5 mM diamide (10 min)(1)
(22) 568. Brown enviromental changes :1M sorbitol - 45 min (1)
(23) 593. Brown enviromental changes :Diauxic Shift Timecourse(1)
(24) 594. Brown enviromental changes :diauxic shift timecourse(1)
(25) 595. Brown enviromental changes :diauxic shift timecourse(1)
(26) 597. Brown enviromental changes :diauxic shift timecourse(1)
(27) 598. Brown enviromental changes :diauxic shift timecourse(1)
(28) 599. Brown enviromental changes :diauxic shift timecourse(1)
(29) 600. Brown enviromental changes :diauxic shift timecourse(1)
(30) 602. Brown enviromental changes :YPD 4 h ypd-2(1)
(31) 603. Brown enviromental changes :YPD 6 h ypd-2(1)
(32) 604. Brown enviromental changes :YPD 8 h ypd-2(1)
(33) 605. Brown enviromental changes :YPD 10 h ypd-2(1)
(34) 606. Brown enviromental changes :YPD 12 h ypd-2(1)
(35) 608. Brown enviromental changes :YPD 2 d ypd-2(1)
(36) 611. Brown enviromental changes :YPD stationary phase 2 h ypd-1(1)
(37) 612. Brown enviromental changes :YPD stationary phase 4 h ypd-1(1)
(38) 613. Brown enviromental changes :YPD stationary phase 8 h ypd-1(1)
(39) 614. Brown enviromental changes :YPD stationary phase 12 h ypd-1(1)
(40) 615. Brown enviromental changes :YPD stationary phase 1 d ypd-1(1)
(41) 616. Brown enviromental changes :YPD stationary phase 2 d ypd-1(1)
(42) 617. Brown enviromental changes :YPD stationary phase 3 d ypd-1(1)
(43) 100 microM BCS 60 min

HAP4 --> COX6 exp. conditions:40

(1) 6. Expression during the cell cycle (cdc15 arrest and release)(15)
(2) 7. Expression during the cell Cycle (cdc28)(15)
(3) 7. Expression during the cell Cycle (cdc28)(16)
(4) 7. Expression during the cell Cycle (cdc28)(17)
(5) 11. Expression during diauxic shift: 9h,11h,13h,15h,17h,19h,21h(5)
(6) 11. Expression during diauxic shift: 9h,11h,13h,15h,17h,19h,21h(6)
(7) 11. Expression during diauxic shift: 9h,11h,13h,15h,17h,19h,21h(7)
(8) 388. Rosetta 2000: Expression in cells with FKS1 under tet promoter(1)
(9) 390. Rosetta 2000: Expression in cells with IDI1 under tet promoter(1)
(10) 428. Expression in strain PM38 (wild type) in response to 30 min 50 nM treatment with rapamycin in YPD(1)
(11) 479. Expression in diploid cells in response to rapamycin (100nM) for: 15min,30min,90min,120min(3)
(12) 482. Expression in response to acid: 10,20,40,60,80,100 min(1)
(13) 482. Expression in response to acid: 10,20,40,60,80,100 min(5)
(14) 488. Brown enviromental changes :Heat Shock 05 minutes hs-1(1)
(15) 494. Brown enviromental changes :Heat Shock 60 minutes hs-1(1)
(16) 517. Brown enviromental changes :29C +1M sorbitol to 33C + 1M sorbitol - 5 minutes(1)
(17) 531. Brown enviromental changes :constant 0.32 mM H2O2 (120 min) redo(1)
(18) 532. Brown enviromental changes :constant 0.32 mM H2O2 (160 min) redo(1)
(19) 536. Brown enviromental changes :1mM Menadione (40 min) redo(1)
(20) 556. Brown enviromental changes :dtt 480 min dtt-2(1)
(21) 557. Brown enviromental changes :1.5 mM diamide (5 min)(1)
(22) 558. Brown enviromental changes :1.5 mM diamide (10 min)(1)
(23) 568. Brown enviromental changes :1M sorbitol - 45 min (1)
(24) 597. Brown enviromental changes :diauxic shift timecourse(1)
(25) 598. Brown enviromental changes :diauxic shift timecourse(1)
(26) 599. Brown enviromental changes :diauxic shift timecourse(1)
(27) 600. Brown enviromental changes :diauxic shift timecourse(1)
(28) 602. Brown enviromental changes :YPD 4 h ypd-2(1)
(29) 603. Brown enviromental changes :YPD 6 h ypd-2(1)
(30) 604. Brown enviromental changes :YPD 8 h ypd-2(1)
(31) 605. Brown enviromental changes :YPD 10 h ypd-2(1)
(32) 606. Brown enviromental changes :YPD 12 h ypd-2(1)
(33) 608. Brown enviromental changes :YPD 2 d ypd-2(1)
(34) 611. Brown enviromental changes :YPD stationary phase 2 h ypd-1(1)
(35) 612. Brown enviromental changes :YPD stationary phase 4 h ypd-1(1)
(36) 613. Brown enviromental changes :YPD stationary phase 8 h ypd-1(1)
(37) 614. Brown enviromental changes :YPD stationary phase 12 h ypd-1(1)
(38) 615. Brown enviromental changes :YPD stationary phase 1 d ypd-1(1)
(39) 616. Brown enviromental changes :YPD stationary phase 2 d ypd-1(1)
(40) 617. Brown enviromental changes :YPD stationary phase 3 d ypd-1(1)

HSF1 -*-> CPR6 exp. conditions:31

(1) 6. Expression during the cell cycle (cdc15 arrest and release)(22)
(2) 6. Expression during the cell cycle (cdc15 arrest and release)(23)
(3) 6. Expression during the cell cycle (cdc15 arrest and release)(24)
(4) 481. Expression in response to heat shock: 15,30,45,60,120 min(1)

(5) 481. Expression in response to heat shock: 15,30,45,60,120 min(2)
(6) 488. Brown enviromental changes :Heat Shock 05 minutes hs-1(1)
(7) 489. Brown enviromental changes :Heat Shock 10 minutes hs-1(1)
(8) 490. Brown enviromental changes :Heat Shock 15 minutes hs-1(1)
(9) 491. Brown enviromental changes :Heat Shock 20 minutes hs-1(1)
(10) 492. Brown enviromental changes :Heat Shock 30 minutes hs-1(1)
(11) 493. Brown enviromental changes :Heat Shock 40 minutes hs-1(1)
(12) 494. Brown enviromental changes :Heat Shock 60 minutes hs-1(1)
(13) 495. Brown enviromental changes :Heat Shock 80 minutes hs-1(1)
(14) 508. Brown enviromental changes :heat shock 17 to 37, 20 minutes(1)
(15) 509. Brown enviromental changes :heat shock 21 to 37, 20 minutes(1)
(16) 510. Brown enviromental changes :heat shock 25 to 37, 20 minutes(1)
(17) 511. Brown enviromental changes :heat shock 29 to 37, 20 minutes(1)
(18) 512. Brown enviromental changes :heat shock 33 to 37, 20 minutes(1)
(19) 514. Brown enviromental changes :29C to 33C - 15 minutes(1)
(20) 517. Brown enviromental changes :29C +1M sorbitol to 33C + 1M sorbitol - 5 minutes(1)
(21) 518. Brown enviromental changes :29C +1M sorbitol to 33C + 1M sorbitol - 15 minutes(1)
(22) 557. Brown enviromental changes :1.5 mM diamide (5 min)(1)
(23) 558. Brown enviromental changes :1.5 mM diamide (10 min)(1)
(24) 559. Brown enviromental changes :1.5 mM diamide (20 min)(1)
(25) 561. Brown enviromental changes :1.5 mM diamide (40 min)(1)
(26) 562. Brown enviromental changes :1.5 mM diamide (50 min)(1)
(27) 563. Brown enviromental changes :1.5 mM diamide (60 min)(1)
(28) 564. Brown enviromental changes :1.5 mM diamide (90 min)(1)
(29) 567. Brown enviromental changes :1M sorbitol - 30 min(1)
(30) 568. Brown enviromental changes :1M sorbitol - 45 min (1)
(31) 623. Brown enviromental changes :DBY7286 37degree heat - 20 min(1)

HSF1 --> HSC82 exp. conditions:31

(1) 6. Expression during the cell cycle (cdc15 arrest and release)(22)
(2) 6. Expression during the cell cycle (cdc15 arrest and release)(23)
(3) 6. Expression during the cell cycle (cdc15 arrest and release)(24)
(4) 481. Expression in response to heat shock: 15,30,45,60,120 min(1)
(5) 481. Expression in response to heat shock: 15,30,45,60,120 min(2)
(6) 488. Brown enviromental changes :Heat Shock 05 minutes hs-1(1)
(7) 489. Brown enviromental changes :Heat Shock 10 minutes hs-1(1)
(8) 490. Brown enviromental changes :Heat Shock 15 minutes hs-1(1)
(9) 491. Brown enviromental changes :Heat Shock 20 minutes hs-1(1)
(10) 492. Brown enviromental changes :Heat Shock 30 minutes hs-1(1)
(11) 493. Brown enviromental changes :Heat Shock 40 minutes hs-1(1)
(12) 494. Brown enviromental changes :Heat Shock 60 minutes hs-1(1)
(13) 495. Brown enviromental changes :Heat Shock 80 minutes hs-1(1)
(14) 508. Brown enviromental changes :heat shock 17 to 37, 20 minutes(1)
(15) 509. Brown enviromental changes :heat shock 21 to 37, 20 minutes(1)
(16) 510. Brown enviromental changes :heat shock 25 to 37, 20 minutes(1)
(17) 511. Brown enviromental changes :heat shock 29 to 37, 20 minutes(1)
(18) 512. Brown enviromental changes :heat shock 33 to 37, 20 minutes(1)
(19) 514. Brown enviromental changes :29C to 33C - 15 minutes(1)
(20) 517. Brown enviromental changes :29C +1M sorbitol to 33C + 1M sorbitol - 5 minutes(1)
(21) 518. Brown enviromental changes :29C +1M sorbitol to 33C + 1M sorbitol - 15 minutes(1)
(22) 557. Brown enviromental changes :1.5 mM diamide (5 min)(1)
(23) 558. Brown enviromental changes :1.5 mM diamide (10 min)(1)
(24) 559. Brown enviromental changes :1.5 mM diamide (20 min)(1)
(25) 561. Brown enviromental changes :1.5 mM diamide (40 min)(1)
(26) 562. Brown enviromental changes :1.5 mM diamide (50 min)(1)
(27) 563. Brown enviromental changes :1.5 mM diamide (60 min)(1)
(28) 564. Brown enviromental changes :1.5 mM diamide (90 min)(1)
(29) 567. Brown enviromental changes :1M sorbitol - 30 min(1)
(30) 568. Brown enviromental changes :1M sorbitol - 45 min (1)
(31) 623. Brown enviromental changes :DBY7286 37degree heat - 20 min(1)

INO2 --| ITR1 exp. conditions:23

(1) 5. Expression during the cell cycle (alpha factor arrest and release)(18)
(2) 8. Expression during the cell cycle (cell size selection and release)(1)
(3) 13. Expression in cells overexpressing Yap1p(1)
(4) 74. Expression in response to overproduction of activated Rho1p(1)
(5) 89. Expression in response to 3-aminotriazole(1)
(6) 332. Rosetta 2000: Expression in cells with CMD1 under tet promoter(1)
(7) 389. Rosetta 2000: Expression in cells with HMG2 under tet promoter(1)
(8) 391. Rosetta 2000: Expression in cells with KAR2 under tet promoter(1)
(9) 392. Rosetta 2000: Expression in cells with PMA1 under tet promoter(1)
(10) 394. Rosetta 2000: Expression in cells with YEF3 under tet promoter(1)
(11) 395. Rosetta 2000: Expression in response to 2-deoxy-D-glucose(1)
(12) 397. Rosetta 2000: Expression in response to Cycloheximide white(1)
(13) 400. Rosetta 2000: Expression in response to Glucosamine(1)
(14) 403. Rosetta 2000: Expression in response to Lovastatin(1)
(15) 406. Rosetta 2000: Expression in response to Terbinafine(1)
(16) 407. Rosetta 2000: Expression in response to Tunicamycin(1)
(17) 446. Expression in response to 0.1% MMS for 10 min(1)
(18) 449. Expression in response to 0.1% MMS for 60 min(1)
(19) 485. Expression in response to peroxide: 10,20,40,60,120 min(2)
(20) 635. Brown enviromental changes :YAP1 overexpression(1)
(21) 673. Expression in response to oligomycin 60min(1)
(22) 674. Expression in response to oligomycin 120min(1)
(23) wt_plus_gamma_90_min

INO2 -*-> PSD1 exp. conditions:23

(1) 5. Expression during the cell cycle (alpha factor arrest and release)(18)
(2) 8. Expression during the cell cycle (cell size selection and release)(1)
(3) 13. Expression in cells overexpressing Yap1p(1)
(4) 74. Expression in response to overproduction of activated Rho1p(1)
(5) 89. Expression in response to 3-aminotriazole(1)
(6) 332. Rosetta 2000: Expression in cells with CMD1 under tet promoter(1)
(7) 389. Rosetta 2000: Expression in cells with HMG2 under tet promoter(1)
(8) 391. Rosetta 2000: Expression in cells with KAR2 under tet promoter(1)
(9) 392. Rosetta 2000: Expression in cells with PMA1 under tet promoter(1)
(10) 394. Rosetta 2000: Expression in cells with YEF3 under tet promoter(1)
(11) 395. Rosetta 2000: Expression in response to 2-deoxy-D-glucose(1)
(12) 397. Rosetta 2000: Expression in response to Cycloheximide white(1)
(13) 400. Rosetta 2000: Expression in response to Glucosamine(1)
(14) 403. Rosetta 2000: Expression in response to Lovastatin(1)
(15) 406. Rosetta 2000: Expression in response to Terbinafine(1)
(16) 407. Rosetta 2000: Expression in response to Tunicamycin(1)

(17) 446. Expression in response to 0.1% MMS for 10 min(1)
(18) 449. Expression in response to 0.1% MMS for 60 min(1)
(19) 485. Expression in response to peroxide: 10,20,40,60,120 min(2)
(20) 635. Brown enviromental changes :YAP1 overexpression(1)
(21) 673. Expression in response to oligomycin 60min(1)
(22) 674. Expression in response to oligomycin 120min(1)
(23) wt_plus_gamma_90_min

MBP1 -*-| AXL2 exp. conditions:21

(1) 3. Cell Cycle: Expression in response to Clb2p (set 1, 40 min)(1)
(2) 5. Expression during the cell cycle (alpha factor arrest and release)(1)
(3) 5. Expression during the cell cycle (alpha factor arrest and release)(8)
(4) 5. Expression during the cell cycle (alpha factor arrest and release)(9)
(5) 5. Expression during the cell cycle (alpha factor arrest and release)(10)
(6) 5. Expression during the cell cycle (alpha factor arrest and release)(17)
(7) 5. Expression during the cell cycle (alpha factor arrest and release)(18)
(8) 6. Expression during the cell cycle (cdc15 arrest and release)(8)
(9) 7. Expression during the cell Cycle (cdc28)(7)
(10) 7. Expression during the cell Cycle (cdc28)(8)
(11) 7. Expression during the cell Cycle (cdc28)(9)
(12) 8. Expression during the cell cycle (cell size selection and release)(2)
(13) 48. Expression in response to 0.15,0.5,1.5,5,15.8,50,158,500 nM alpha-factor(4)
(14) 48. Expression in response to 0.15,0.5,1.5,5,15.8,50,158,500 nM alpha-factor(5)
(15) 48. Expression in response to 0.15,0.5,1.5,5,15.8,50,158,500 nM alpha-factor(6)
(16) 48. Expression in response to 0.15,0.5,1.5,5,15.8,50,158,500 nM alpha-factor(7)
(17) 48. Expression in response to 0.15,0.5,1.5,5,15.8,50,158,500 nM alpha-factor(8)
(18) 49. Expression in response to 50 nM alpha-factor: 0,15,30,45,60,90,120 min(2)
(19) 49. Expression in response to 50 nM alpha-factor: 0,15,30,45,60,90,120 min(3)
(20) 49. Expression in response to 50 nM alpha-factor: 0,15,30,45,60,90,120 min(4)
(21) 49. Expression in response to 50 nM alpha-factor: 0,15,30,45,60,90,120 min(6)

MBP1 --| CDC21 exp. conditions:21

(1) 3. Cell Cycle: Expression in response to Clb2p (set 1, 40 min)(1)
(2) 5. Expression during the cell cycle (alpha factor arrest and release)(1)
(3) 5. Expression during the cell cycle (alpha factor arrest and release)(8)
(4) 5. Expression during the cell cycle (alpha factor arrest and release)(9)
(5) 5. Expression during the cell cycle (alpha factor arrest and release)(10)
(6) 5. Expression during the cell cycle (alpha factor arrest and release)(17)
(7) 5. Expression during the cell cycle (alpha factor arrest and release)(18)
(8) 6. Expression during the cell cycle (cdc15 arrest and release)(8)
(9) 7. Expression during the cell Cycle (cdc28)(7)
(10) 7. Expression during the cell Cycle (cdc28)(8)
(11) 7. Expression during the cell Cycle (cdc28)(9)
(12) 8. Expression during the cell cycle (cell size selection and release)(2)
(13) 48. Expression in response to 0.15,0.5,1.5,5,15.8,50,158,500 nM alpha-factor(4)
(14) 48. Expression in response to 0.15,0.5,1.5,5,15.8,50,158,500 nM alpha-factor(5)
(15) 48. Expression in response to 0.15,0.5,1.5,5,15.8,50,158,500 nM alpha-factor(6)
(16) 48. Expression in response to 0.15,0.5,1.5,5,15.8,50,158,500 nM alpha-factor(7)
(17) 48. Expression in response to 0.15,0.5,1.5,5,15.8,50,158,500 nM alpha-factor(8)
(18) 49. Expression in response to 50 nM alpha-factor: 0,15,30,45,60,90,120 min(2)
(19) 49. Expression in response to 50 nM alpha-factor: 0,15,30,45,60,90,120 min(3)
(20) 49. Expression in response to 50 nM alpha-factor: 0,15,30,45,60,90,120 min(4)
(21) 49. Expression in response to 50 nM alpha-factor: 0,15,30,45,60,90,120 min(6)

MBP1 -*-| HCM1 exp. conditions:21

(1) 3. Cell Cycle: Expression in response to Clb2p (set 1, 40 min)(1)
(2) 5. Expression during the cell cycle (alpha factor arrest and release)(1)
(3) 5. Expression during the cell cycle (alpha factor arrest and release)(8)
(4) 5. Expression during the cell cycle (alpha factor arrest and release)(9)
(5) 5. Expression during the cell cycle (alpha factor arrest and release)(10)
(6) 5. Expression during the cell cycle (alpha factor arrest and release)(17)
(7) 5. Expression during the cell cycle (alpha factor arrest and release)(18)
(8) 6. Expression during the cell cycle (cdc15 arrest and release)(8)
(9) 7. Expression during the cell Cycle (cdc28)(7)
(10) 7. Expression during the cell Cycle (cdc28)(8)
(11) 7. Expression during the cell Cycle (cdc28)(9)
(12) 8. Expression during the cell cycle (cell size selection and release)(2)
(13) 48. Expression in response to 0.15,0.5,1.5,5,15.8,50,158,500 nM alpha-factor(4)
(14) 48. Expression in response to 0.15,0.5,1.5,5,15.8,50,158,500 nM alpha-factor(5)
(15) 48. Expression in response to 0.15,0.5,1.5,5,15.8,50,158,500 nM alpha-factor(6)
(16) 48. Expression in response to 0.15,0.5,1.5,5,15.8,50,158,500 nM alpha-factor(7)
(17) 48. Expression in response to 0.15,0.5,1.5,5,15.8,50,158,500 nM alpha-factor(8)
(18) 49. Expression in response to 50 nM alpha-factor: 0,15,30,45,60,90,120 min(2)
(19) 49. Expression in response to 50 nM alpha-factor: 0,15,30,45,60,90,120 min(3)
(20) 49. Expression in response to 50 nM alpha-factor: 0,15,30,45,60,90,120 min(4)
(21) 49. Expression in response to 50 nM alpha-factor: 0,15,30,45,60,90,120 min(6)

MBP1 -*-| SPT21 exp. conditions:21

(1) 3. Cell Cycle: Expression in response to Clb2p (set 1, 40 min)(1)
(2) 5. Expression during the cell cycle (alpha factor arrest and release)(1)
(3) 5. Expression during the cell cycle (alpha factor arrest and release)(8)
(4) 5. Expression during the cell cycle (alpha factor arrest and release)(9)
(5) 5. Expression during the cell cycle (alpha factor arrest and release)(10)
(6) 5. Expression during the cell cycle (alpha factor arrest and release)(17)
(7) 5. Expression during the cell cycle (alpha factor arrest and release)(18)
(8) 6. Expression during the cell cycle (cdc15 arrest and release)(8)
(9) 7. Expression during the cell Cycle (cdc28)(7)
(10) 7. Expression during the cell Cycle (cdc28)(8)
(11) 7. Expression during the cell Cycle (cdc28)(9)
(12) 8. Expression during the cell cycle (cell size selection and release)(2)
(13) 48. Expression in response to 0.15,0.5,1.5,5,15.8,50,158,500 nM alpha-factor(4)
(14) 48. Expression in response to 0.15,0.5,1.5,5,15.8,50,158,500 nM alpha-factor(5)
(15) 48. Expression in response to 0.15,0.5,1.5,5,15.8,50,158,500 nM alpha-factor(6)
(16) 48. Expression in response to 0.15,0.5,1.5,5,15.8,50,158,500 nM alpha-factor(7)
(17) 48. Expression in response to 0.15,0.5,1.5,5,15.8,50,158,500 nM alpha-factor(8)
(18) 49. Expression in response to 50 nM alpha-factor: 0,15,30,45,60,90,120 min(2)
(19) 49. Expression in response to 50 nM alpha-factor: 0,15,30,45,60,90,120 min(3)
(20) 49. Expression in response to 50 nM alpha-factor: 0,15,30,45,60,90,120 min(4)
(21) 49. Expression in response to 50 nM alpha-factor: 0,15,30,45,60,90,120 min(6)

MCM1 --> CLN3 exp. conditions:23

(1) 1. Cell cycle: Expression in response to Cln3p (set 1)(1)
(2) 2. Cell Cycle: Expression in response to Cln3p (set 2)(1)
(3) 4. Cell Cycle: Expression in response to Clb2p (set 2, 30 min)(1)
(4) 5. Expression during the cell cycle (alpha factor arrest and release)(2)
(5) 5. Expression during the cell cycle (alpha factor arrest and release)(10)
(6) 5. Expression during the cell cycle (alpha factor arrest and release)(11)
(7) 6. Expression during the cell cycle (cdc15 arrest and release)(1)
(8) 6. Expression during the cell cycle (cdc15 arrest and release)(2)
(9) 7. Expression during the cell Cycle (cdc28)(10)
(10) 8. Expression during the cell cycle (cell size selection and release)(13)
(11) 49. Expression in response to 50 nM alpha-factor: 0,15,30,45,60,90,120 min(7)
(12) 386. Rosetta 2000: Expression in cells with CDC42 under tet promoter(1)
(13) 388. Rosetta 2000: Expression in cells with FKS1 under tet promoter(1)
(14) 391. Rosetta 2000: Expression in cells with KAR2 under tet promoter(1)
(15) 446. Expression in response to 0.1% MMS for 10 min(1)
(16) 447. Expression in response to 0.1% MMS for 30 min(1)
(17) 477. Expression in response to trichostatin A (TSA): 15min,30min,60min,120min(1)
(18) 481. Expression in response to heat shock: 15,30,45,60,120 min(5)
(19) 556. Brown enviromental changes :dtt 480 min dtt-2(1)
(20) 674. Expression in response to oligomycin 120min(1)
(21) 681. Expression in response to 0.4M NaCl for 10 min in wild type(1)
(22) (Rich Media 2% Glucose YPD-185588) wt 5mM aF, 30 min.
(23) (Var.) Rich Media 2% Glucose YPD-Average wt 5mM aF, 30 min.

MCM1 -*-> DBF2 exp. conditions:27

(1) 3. Cell Cycle: Expression in response to Clb2p (set 1, 40 min)(1)
(2) 4. Cell Cycle: Expression in response to Clb2p (set 2, 30 min)(1)
(3) 5. Expression during the cell cycle (alpha factor arrest and release)(8)
(4) 5. Expression during the cell cycle (alpha factor arrest and release)(9)
(5) 5. Expression during the cell cycle (alpha factor arrest and release)(10)
(6) 5. Expression during the cell cycle (alpha factor arrest and release)(16)
(7) 5. Expression during the cell cycle (alpha factor arrest and release)(17)
(8) 5. Expression during the cell cycle (alpha factor arrest and release)(18)
(9) 6. Expression during the cell cycle (cdc15 arrest and release)(6)
(10) 6. Expression during the cell cycle (cdc15 arrest and release)(7)
(11) 6. Expression during the cell cycle (cdc15 arrest and release)(8)
(12) 6. Expression during the cell cycle (cdc15 arrest and release)(16)
(13) 6. Expression during the cell cycle (cdc15 arrest and release)(18)
(14) 6. Expression during the cell cycle (cdc15 arrest and release)(20)
(15) 7. Expression during the cell Cycle (cdc28)(7)
(16) 7. Expression during the cell Cycle (cdc28)(8)
(17) 7. Expression during the cell Cycle (cdc28)(9)
(18) 7. Expression during the cell Cycle (cdc28)(16)
(19) 7. Expression during the cell Cycle (cdc28)(17)
(20) 8. Expression during the cell cycle (cell size selection and release)(8)
(21) 8. Expression during the cell cycle (cell size selection and release)(9)
(22) 8. Expression during the cell cycle (cell size selection and release)(10)
(23) 8. Expression during the cell cycle (cell size selection and release)(11)
(24) 8. Expression during the cell cycle (cell size selection and release)(12)
(25) 8. Expression during the cell cycle (cell size selection and release)(13)
(26) 8. Expression during the cell cycle (cell size selection and release)(14)
(27) 556. Brown enviromental changes :dtt 480 min dtt-2(1)

MCM1 --> SWI5 exp. conditions:27

(1) 3. Cell Cycle: Expression in response to Clb2p (set 1, 40 min)(1)
(2) 4. Cell Cycle: Expression in response to Clb2p (set 2, 30 min)(1)
(3) 5. Expression during the cell cycle (alpha factor arrest and release)(8)
(4) 5. Expression during the cell cycle (alpha factor arrest and release)(9)
(5) 5. Expression during the cell cycle (alpha factor arrest and release)(10)
(6) 5. Expression during the cell cycle (alpha factor arrest and release)(16)
(7) 5. Expression during the cell cycle (alpha factor arrest and release)(17)
(8) 5. Expression during the cell cycle (alpha factor arrest and release)(18)
(9) 6. Expression during the cell cycle (cdc15 arrest and release)(6)
(10) 6. Expression during the cell cycle (cdc15 arrest and release)(7)
(11) 6. Expression during the cell cycle (cdc15 arrest and release)(8)
(12) 6. Expression during the cell cycle (cdc15 arrest and release)(16)
(13) 6. Expression during the cell cycle (cdc15 arrest and release)(18)
(14) 6. Expression during the cell cycle (cdc15 arrest and release)(20)
(15) 7. Expression during the cell Cycle (cdc28)(7)
(16) 7. Expression during the cell Cycle (cdc28)(8)
(17) 7. Expression during the cell Cycle (cdc28)(9)
(18) 7. Expression during the cell Cycle (cdc28)(16)
(19) 7. Expression during the cell Cycle (cdc28)(17)
(20) 8. Expression during the cell cycle (cell size selection and release)(8)
(21) 8. Expression during the cell cycle (cell size selection and release)(9)
(22) 8. Expression during the cell cycle (cell size selection and release)(10)
(23) 8. Expression during the cell cycle (cell size selection and release)(11)
(24) 8. Expression during the cell cycle (cell size selection and release)(12)
(25) 8. Expression during the cell cycle (cell size selection and release)(13)
(26) 8. Expression during the cell cycle (cell size selection and release)(14)
(27) 556. Brown enviromental changes :dtt 480 min dtt-2(1)

PHO4 -*-> CTF19 exp. conditions:16

(1) 3. Cell Cycle: Expression in response to Clb2p (set 1, 40 min)(1)
(2) 4. Cell Cycle: Expression in response to Clb2p (set 2, 30 min)(1)
(3) 5. Expression during the cell cycle (alpha factor arrest and release)(9)
(4) 5. Expression during the cell cycle (alpha factor arrest and release)(10)
(5) 5. Expression during the cell cycle (alpha factor arrest and release)(11)
(6) 5. Expression during the cell cycle (alpha factor arrest and release)(12)
(7) PHO4c vs WT(1)
(8) pho80 vs WT(1)
(9) pho85 vs WT(1)
(10) PHO81c vs WT exp1(1)
(11) PHO81c vs WT exp2(1)
(12) 573. Brown enviromental changes :Hypo-osmotic shock - 15 min(1)
(13) 574. Brown enviromental changes :Hypo-osmotic shock - 30 min(1)
(14) 575. Brown enviromental changes :Hypo-osmotic shock - 45 min(1)
(15) MAC1-up (B)
(16) MAC1-up (C)

PHO4 --> PHO84 exp. conditions:16

(1) 3. Cell Cycle: Expression in response to Clb2p (set 1, 40 min)(1)
(2) 4. Cell Cycle: Expression in response to Clb2p (set 2, 30 min)(1)
(3) 5. Expression during the cell cycle (alpha factor arrest and release)(9)
(4) 5. Expression during the cell cycle (alpha factor arrest and release)(10)
(5) 5. Expression during the cell cycle (alpha factor arrest and release)(11)
(6) 5. Expression during the cell cycle (alpha factor arrest and release)(12)
(7) PHO4c vs WT(1)
(8) pho80 vs WT(1)
(9) pho85 vs WT(1)
(10) PHO81c vs WT exp1(1)
(11) PHO81c vs WT exp2(1)
(12) 573. Brown enviromental changes :Hypo-osmotic shock - 15 min(1)
(13) 574. Brown enviromental changes :Hypo-osmotic shock - 30 min(1)
(14) 575. Brown enviromental changes :Hypo-osmotic shock - 45 min(1)
(15) MAC1-up (B)
(16) MAC1-up (C)

RCS1 --> ATX1 exp. conditions:21

(1) 5. Expression during the cell cycle (alpha factor arrest and release)(13)
(2) 5. Expression during the cell cycle (alpha factor arrest and release)(17)
(3) 6. Expression during the cell cycle (cdc15 arrest and release)(10)
(4) 6. Expression during the cell cycle (cdc15 arrest and release)(24)
(5) 7. Expression during the cell Cycle (cdc28)(16)
(6) 7. Expression during the cell Cycle (cdc28)(17)
(7) 450. Expression in response to low MNNG (8 microgram/ml) for 60 min(1)
(8) 451. Expression in response to BCNU (200 micromolar) for 60 min(1)
(9) 483. Expression in response to alkali: 10,20,40,60,80,100 min(1)
(10) 483. Expression in response to alkali: 10,20,40,60,80,100 min(2)
(11) 483. Expression in response to alkali: 10,20,40,60,80,100 min(6)
(12) 487. Expression in response to sorbitol: 15 30 45 90 120 min(3)
(13) 516. Brown enviromental changes :33C vs. 30C - 90 minutes(1)
(14) 519. Brown enviromental changes :29C +1M sorbitol to 33C + 1M sorbitol - 30 minutes(1)
(15) 536. Brown enviromental changes :1mM Menadione (40 min) redo(1)
(16) 537. Brown enviromental changes :1 mM Menadione (50 min)redo(1)
(17) 538. Brown enviromental changes :1 mM Menadione (80 min) redo(1)
(18) 575. Brown enviromental changes :Hypo-osmotic shock - 45 min(1)
(19) wt_plus_gamma_20_min
(20) DES460 (wild type) + heat 20 min
(21) 100 microM CuSO4 30 min (B)

RCS1 -*-> SIT1 exp. conditions:27

(1) 5. Expression during the cell cycle (alpha factor arrest and release)(10)
(2) 5. Expression during the cell cycle (alpha factor arrest and release)(11)
(3) 5. Expression during the cell cycle (alpha factor arrest and release)(13)
(4) 5. Expression during the cell cycle (alpha factor arrest and release)(17)
(5) 6. Expression during the cell cycle (cdc15 arrest and release)(7)
(6) 6. Expression during the cell cycle (cdc15 arrest and release)(8)
(7) 6. Expression during the cell cycle (cdc15 arrest and release)(10)
(8) 6. Expression during the cell cycle (cdc15 arrest and release)(17)
(9) 7. Expression during the cell Cycle (cdc28)(13)
(10) 8. Expression during the cell cycle (cell size selection and release)(2)
(11) 406. Rosetta 2000: Expression in response to Terbinafine(1)
(12) PHO81c vs WT exp1(1)
(13) 446. Expression in response to 0.1% MMS for 10 min(1)
(14) 451. Expression in response to BCNU (200 micromolar) for 60 min(1)
(15) 483. Expression in response to alkali: 10,20,40,60,80,100 min(6)
(16) 487. Expression in response to sorbitol: 15 30 45 90 120 min(3)
(17) 516. Brown enviromental changes :33C vs. 30C - 90 minutes(1)
(18) 570. Brown enviromental changes :1M sorbitol - 90 min(1)
(19) 573. Brown enviromental changes :Hypo-osmotic shock - 15 min(1)
(20) 575. Brown enviromental changes :Hypo-osmotic shock - 45 min(1)
(21) 586. Brown enviromental changes :Nitrogen Depletion 4 h(1)
(22) wt_plus_gamma_20_min
(23) wt_plus_gamma_30_min
(24) DES460 (wt) - mock irradiation - 30 min
(25) DES460 (wt) - mock irradiation - 90 min
(26) 100 microM CuSO4 30 min (B)
(27) wt-gal

RPN4 -*-> PRE1 exp. conditions:33

(1) 6. Expression during the cell cycle (cdc15 arrest and release)(22)
(2) 6. Expression during the cell cycle (cdc15 arrest and release)(23)
(3) 6. Expression during the cell cycle (cdc15 arrest and release)(24)
(4) 7. Expression during the cell Cycle (cdc28)(10)
(5) 7. Expression during the cell Cycle (cdc28)(11)
(6) 8. Expression during the cell cycle (cell size selection and release)(2)
(7) 445. Expression in response to 0.1% MMS for 60 min (average of 3 experiments)(1)
(8) 447. Expression in response to 0.1% MMS for 30 min(1)
(9) 448. Expression in response to 0.1% MMS for 60 min(1)
(10) 449. Expression in response to 0.1% MMS for 60 min(1)
(11) 450. Expression in response to low MNNG (8 microgram/ml) for 60 min(1)
(12) 451. Expression in response to BCNU (200 micromolar) for 60 min(1)
(13) 462. Expression in response to 0.05% MMS for 60 min(1)
(14) 463. Expression in response to 0.1% MMS for 60 min(1)
(15) 464. Expression in response to 0.2% MMS for 60 min(1)
(16) 479. Expression in diploid cells in response to rapamycin (100nM) for: 15min,30min,90min,120min(2)
(17) 481. Expression in response to heat shock: 15,30,45,60,120 min(1)
(18) 481. Expression in response to heat shock: 15,30,45,60,120 min(2)
(19) 481. Expression in response to heat shock: 15,30,45,60,120 min(3)
(20) 481. Expression in response to heat shock: 15,30,45,60,120 min(4)
(21) 561. Brown enviromental changes :1.5 mM diamide (40 min)(1)
(22) 562. Brown enviromental changes :1.5 mM diamide (50 min)(1)
(23) 563. Brown enviromental changes :1.5 mM diamide (60 min)(1)
(24) 685. Expression in response to 0.8M NaCl for 20 min in wild type(1)
(25) DES460 + 0.02% MMS - 5 min
(26) DES460 + 0.02% MMS - 30 min
(27) DES460 + 0.2% MMS - 45 min
(28) DES460 + 0.02% MMS - 60 min

(29) DES460 + 0.02% MMS - 90 min
(30) DES460 + 0.02% MMS - 120 min
(31) wt_plus_gamma_10_min
(32) wt_plus_gamma_30_min
(33) DES460 (wild type) + heat 20 min

RPN4 --> PRE6 exp. conditions:48

(1) 6. Expression during the cell cycle (cdc15 arrest and release)(22)
(2) 6. Expression during the cell cycle (cdc15 arrest and release)(23)
(3) 6. Expression during the cell cycle (cdc15 arrest and release)(24)
(4) 7. Expression during the cell Cycle (cdc28)(10)
(5) 7. Expression during the cell Cycle (cdc28)(11)
(6) 8. Expression during the cell cycle (cell size selection and release)(1)
(7) 8. Expression during the cell cycle (cell size selection and release)(2)
(8) 445. Expression in response to 0.1% MMS for 60 min (average of 3 experiments)(1)
(9) 447. Expression in response to 0.1% MMS for 30 min(1)
(10) 448. Expression in response to 0.1% MMS for 60 min(1)
(11) 449. Expression in response to 0.1% MMS for 60 min(1)
(12) 450. Expression in response to low MNNG (8 microgram/ml) for 60 min(1)
(13) 451. Expression in response to BCNU (200 micromolar) for 60 min(1)
(14) 462. Expression in response to 0.05% MMS for 60 min(1)
(15) 463. Expression in response to 0.1% MMS for 60 min(1)
(16) 464. Expression in response to 0.2% MMS for 60 min(1)
(17) 479. Expression in diploid cells in response to rapamycin (100nM) for: 15min,30min,90min,120min(2)
(18) 481. Expression in response to heat shock: 15,30,45,60,120 min(1)
(19) 481. Expression in response to heat shock: 15,30,45,60,120 min(2)
(20) 481. Expression in response to heat shock: 15,30,45,60,120 min(3)
(21) 481. Expression in response to heat shock: 15,30,45,60,120 min(4)
(22) 482. Expression in response to acid: 10,20,40,60,80,100 min(4)
(23) 485. Expression in response to peroxide: 10,20,40,60,120 min(4)
(24) 486. Expression in response to NaCl: 15 30 45 60 120 min(3)
(25) 486. Expression in response to NaCl: 15 30 45 60 120 min(4)
(26) 487. Expression in response to sorbitol: 15 30 45 90 120 min(3)
(27) 533. Brown enviromental changes :1 mM Menadione (10 min)redo(1)
(28) 557. Brown enviromental changes :1.5 mM diamide (5 min)(1)
(29) 558. Brown enviromental changes :1.5 mM diamide (10 min)(1)
(30) 559. Brown enviromental changes :1.5 mM diamide (20 min)(1)
(31) 560. Brown enviromental changes :1.5 mM diamide (30 min)(1)
(32) 561. Brown enviromental changes :1.5 mM diamide (40 min)(1)
(33) 562. Brown enviromental changes :1.5 mM diamide (50 min)(1)
(34) 563. Brown enviromental changes :1.5 mM diamide (60 min)(1)
(35) 564. Brown enviromental changes :1.5 mM diamide (90 min)(1)
(36) 565. Brown enviromental changes :1M sorbitol - 5 min(1)
(37) 635. Brown enviromental changes :YAP1 overexpression(1)
(38) 681. Expression in response to 0.4M NaCl for 10 min in wild type(1)
(39) 685. Expression in response to 0.8M NaCl for 20 min in wild type(1)
(40) DES460 + 0.02% MMS - 5 min
(41) DES460 + 0.02% MMS - 30 min
(42) DES460 + 0.2% MMS - 45 min
(43) DES460 + 0.02% MMS - 60 min
(44) DES460 + 0.02% MMS - 90 min
(45) DES460 + 0.02% MMS - 120 min
(46) wt_plus_gamma_10_min
(47) wt_plus_gamma_30_min
(48) DES460 (wild type) + heat 20 min

SIP4 --> MDH2 exp. conditions:23

(1) 5. Expression during the cell cycle (alpha factor arrest and release)(2)
(2) 7. Expression during the cell Cycle (cdc28)(9)
(3) 8. Expression during the cell cycle (cell size selection and release)(8)
(4) 8. Expression during the cell cycle (cell size selection and release)(9)
(5) 8. Expression during the cell cycle (cell size selection and release)(13)
(6) 13. Expression in cells overexpressing Yap1p(1)
(7) 74. Expression in response to overproduction of activated Rho1p(1)
(8) 482. Expression in response to acid: 10,20,40,60,80,100 min(3)
(9) 483. Expression in response to alkali: 10,20,40,60,80,100 min(5)
(10) 483. Expression in response to alkali: 10,20,40,60,80,100 min(6)
(11) 504. Brown enviromental changes :37C to 25C shock - 30 min(1)
(12) 505. Brown enviromental changes :37C to 25C shock - 45 min(1)
(13) 506. Brown enviromental changes :37C to 25C shock - 60 min(1)
(14) 607. Brown enviromental changes :YPD 1 d ypd-2(1)
(15) 615. Brown enviromental changes :YPD stationary phase 1 d ypd-1(1)
(16) 616. Brown enviromental changes :YPD stationary phase 2 d ypd-1(1)
(17) 617. Brown enviromental changes :YPD stationary phase 3 d ypd-1(1)
(18) 635. Brown enviromental changes :YAP1 overexpression(1)
(19) 684. Expression in response to 0.8M NaCl for 10 min in wild type(1)
(20) wt_plus_gamma_5_min
(21) wt_plus_gamma_10_min
(22) wt_plus_gamma_20_min
(23) 100 microM BCS 30 min

SIP4 -*-> PCK1 exp. conditions:23

(1) 8. Expression during the cell cycle (cell size selection and release)(9)
(2) 8. Expression during the cell cycle (cell size selection and release)(13)
(3) 11. Expression during diauxic shift: 9h,11h,13h,15h,17h,19h,21h(7)
(4) 49. Expression in response to 50 nM alpha-factor: 0,15,30,45,60,90,120 min(2)
(5) 387. Rosetta 2000: Expression in cells with ERG11 under tet promoter(1)
(6) 392. Rosetta 2000: Expression in cells with PMA1 under tet promoter(1)
(7) 528. Brown enviromental changes :constant 0.32 mM H2O2 (60 min) redo(1)
(8) 567. Brown enviromental changes :1M sorbitol - 30 min(1)
(9) 599. Brown enviromental changes :diauxic shift timecourse(1)
(10) 600. Brown enviromental changes :diauxic shift timecourse(1)
(11) 604. Brown enviromental changes :YPD 8 h ypd-2(1)
(12) 605. Brown enviromental changes :YPD 10 h ypd-2(1)
(13) 606. Brown enviromental changes :YPD 12 h ypd-2(1)
(14) 607. Brown enviromental changes :YPD 1 d ypd-2(1)
(15) 608. Brown enviromental changes :YPD 2 d ypd-2(1)
(16) 614. Brown enviromental changes :YPD stationary phase 12 h ypd-1(1)
(17) 615. Brown enviromental changes :YPD stationary phase 1 d ypd-1(1)
(18) 616. Brown enviromental changes :YPD stationary phase 2 d ypd-1(1)
(19) 617. Brown enviromental changes :YPD stationary phase 3 d ypd-1(1)
(20) 618. Brown enviromental changes :YPD stationary phase 5 d ypd-1(1)
(21) 675. Expression in response to propionate(1)

(22) wt_plus_gamma_20_min
(23) wt_plus_gamma_120_min

STE12 --> AGA1 exp. conditions:33

(1) 5. Expression during the cell cycle (alpha factor arrest and release)(1)
(2) 11. Expression during diauxic shift: 9h,11h,13h,15h,17h,19h,21h(3)
(3) 26. Fink: Expression in diploid high copy TEC1(1)
(4) 48. Expression in response to 0.15,0.5,1.5,5,15.8,50,158,500 nM alpha-factor(3)
(5) 48. Expression in response to 0.15,0.5,1.5,5,15.8,50,158,500 nM alpha-factor(4)
(6) 48. Expression in response to 0.15,0.5,1.5,5,15.8,50,158,500 nM alpha-factor(5)
(7) 48. Expression in response to 0.15,0.5,1.5,5,15.8,50,158,500 nM alpha-factor(6)
(8) 48. Expression in response to 0.15,0.5,1.5,5,15.8,50,158,500 nM alpha-factor(7)
(9) 48. Expression in response to 0.15,0.5,1.5,5,15.8,50,158,500 nM alpha-factor(8)
(10) 49. Expression in response to 50 nM alpha-factor: 0,15,30,45,60,90,120 min(1)
(11) 49. Expression in response to 50 nM alpha-factor: 0,15,30,45,60,90,120 min(2)
(12) 49. Expression in response to 50 nM alpha-factor: 0,15,30,45,60,90,120 min(3)
(13) 49. Expression in response to 50 nM alpha-factor: 0,15,30,45,60,90,120 min(4)
(14) 49. Expression in response to 50 nM alpha-factor: 0,15,30,45,60,90,120 min(5)
(15) 49. Expression in response to 50 nM alpha-factor: 0,15,30,45,60,90,120 min(6)
(16) 49. Expression in response to 50 nM alpha-factor: 0,15,30,45,60,90,120 min(7)
(17) 53. Expression in response to overproduction of Ste4p(1)
(18) 54. Expression in response to overproduction of Ste5p(1)
(19) 55. Expression in response to overproduction of Ste11p(1)
(20) 56. Expression in response to overproduction of Ste12p(1)
(21) 395. Rosetta 2000: Expression in response to 2-deoxy-D-glucose(1)
(22) 404. Rosetta 2000: Expression in response to MMS(1)
(23) 407. Rosetta 2000: Expression in response to Tunicamycin(1)
(24) 563. Brown enviromental changes :1.5 mM diamide (60 min)(1)
(25) 572. Brown enviromental changes :Hypo-osmotic shock - 5 min(1)
(26) 595. Brown enviromental changes :diauxic shift timecourse(1)
(27) 681. Expression in response to 0.4M NaCl for 10 min in wild type(1)
(28) 685. Expression in response to 0.8M NaCl for 20 min in wild type(1)
(29) (Rich Media 2% Glucose YPD-185588) wt 5mM aF, 30 min.
(30) (Rich Media 2% Glucose YPD-185769) wt 5mM aF, 30 min.
(31) Rich Media 2% Glucose YPD-Average wt 5mM aF, 30 min.
(32) (Var.) Rich Media 2% Glucose YPD-Average wt 5mM aF, 30 min.
(33) 100 microM BCS 30 min

STE12 --> CHS1 exp. conditions:30

(1) 4. Cell Cycle: Expression in response to Clb2p (set 2, 30 min)(1)
(2) 5. Expression during the cell cycle (alpha factor arrest and release)(1)
(3) 5. Expression during the cell cycle (alpha factor arrest and release)(11)
(4) 5. Expression during the cell cycle (alpha factor arrest and release)(12)
(5) 5. Expression during the cell cycle (alpha factor arrest and release)(13)
(6) 6. Expression during the cell cycle (cdc15 arrest and release)(22)
(7) 6. Expression during the cell cycle (cdc15 arrest and release)(23)
(8) 6. Expression during the cell cycle (cdc15 arrest and release)(24)
(9) 8. Expression during the cell cycle (cell size selection and release)(12)
(10) 48. Expression in response to 0.15,0.5,1.5,5,15.8,50,158,500 nM alpha-factor(3)
(11) 48. Expression in response to 0.15,0.5,1.5,5,15.8,50,158,500 nM alpha-factor(4)
(12) 48. Expression in response to 0.15,0.5,1.5,5,15.8,50,158,500 nM alpha-factor(5)
(13) 48. Expression in response to 0.15,0.5,1.5,5,15.8,50,158,500 nM alpha-factor(6)
(14) 48. Expression in response to 0.15,0.5,1.5,5,15.8,50,158,500 nM alpha-factor(7)
(15) 48. Expression in response to 0.15,0.5,1.5,5,15.8,50,158,500 nM alpha-factor(8)
(16) 49. Expression in response to 50 nM alpha-factor: 0,15,30,45,60,90,120 min(1)
(17) 49. Expression in response to 50 nM alpha-factor: 0,15,30,45,60,90,120 min(2)
(18) 49. Expression in response to 50 nM alpha-factor: 0,15,30,45,60,90,120 min(3)
(19) 49. Expression in response to 50 nM alpha-factor: 0,15,30,45,60,90,120 min(4)
(20) 49. Expression in response to 50 nM alpha-factor: 0,15,30,45,60,90,120 min(5)
(21) 49. Expression in response to 50 nM alpha-factor: 0,15,30,45,60,90,120 min(6)
(22) 49. Expression in response to 50 nM alpha-factor: 0,15,30,45,60,90,120 min(7)
(23) 53. Expression in response to overproduction of Ste4p(1)
(24) 54. Expression in response to overproduction of Ste5p(1)
(25) 55. Expression in response to overproduction of Ste11p(1)
(26) 56. Expression in response to overproduction of Ste12p(1)
(27) 533. Brown enviromental changes :1 mM Menadione (10 min)redo(1)
(28) (Rich Media 2% Glucose YPD-185588) wt 5mM aF, 30 min.
(29) (Rich Media 2% Glucose YPD-185769) wt 5mM aF, 30 min.
(30) Rich Media 2% Glucose YPD-Average wt 5mM aF, 30 min.

STE12 --> CIK1 exp. conditions:26

(1) 5. Expression during the cell cycle (alpha factor arrest and release)(1)
(2) 11. Expression during diauxic shift: 9h,11h,13h,15h,17h,19h,21h(3)
(3) 26. Fink: Expression in diploid high copy TEC1(1)
(4) 48. Expression in response to 0.15,0.5,1.5,5,15.8,50,158,500 nM alpha-factor(4)
(5) 48. Expression in response to 0.15,0.5,1.5,5,15.8,50,158,500 nM alpha-factor(5)
(6) 48. Expression in response to 0.15,0.5,1.5,5,15.8,50,158,500 nM alpha-factor(6)
(7) 48. Expression in response to 0.15,0.5,1.5,5,15.8,50,158,500 nM alpha-factor(7)
(8) 49. Expression in response to 50 nM alpha-factor: 0,15,30,45,60,90,120 min(1)
(9) 49. Expression in response to 50 nM alpha-factor: 0,15,30,45,60,90,120 min(2)
(10) 49. Expression in response to 50 nM alpha-factor: 0,15,30,45,60,90,120 min(3)
(11) 49. Expression in response to 50 nM alpha-factor: 0,15,30,45,60,90,120 min(4)
(12) 49. Expression in response to 50 nM alpha-factor: 0,15,30,45,60,90,120 min(6)
(13) 49. Expression in response to 50 nM alpha-factor: 0,15,30,45,60,90,120 min(7)
(14) 53. Expression in response to overproduction of Ste4p(1)
(15) 54. Expression in response to overproduction of Ste5p(1)
(16) 56. Expression in response to overproduction of Ste12p(1)
(17) 395. Rosetta 2000: Expression in response to 2-deoxy-D-glucose(1)
(18) 404. Rosetta 2000: Expression in response to MMS(1)
(19) 407. Rosetta 2000: Expression in response to Tunicamycin(1)
(20) 563. Brown enviromental changes :1.5 mM diamide (60 min)(1)
(21) 572. Brown enviromental changes :Hypo-osmotic shock - 5 min(1)
(22) 595. Brown enviromental changes :diauxic shift timecourse(1)
(23) 681. Expression in response to 0.4M NaCl for 10 min in wild type(1)
(24) 685. Expression in response to 0.8M NaCl for 20 min in wild type(1)
(25) (Var.) Rich Media 2% Glucose YPD-Average wt 5mM aF, 30 min.
(26) 100 microM BCS 30 min

STE12 --> ERG24 exp. conditions:26

(1) 5. Expression during the cell cycle (alpha factor arrest and release)(1)

(2) 11. Expression during diauxic shift: 9h,11h,13h,15h,17h,19h,21h(2)
(3) 11. Expression during diauxic shift: 9h,11h,13h,15h,17h,19h,21h(3)
(4) 48. Expression in response to 0.15,0.5,1.5,5,15.8,50,158,500 nM alpha-factor(4)
(5) 48. Expression in response to 0.15,0.5,1.5,5,15.8,50,158,500 nM alpha-factor(5)
(6) 48. Expression in response to 0.15,0.5,1.5,5,15.8,50,158,500 nM alpha-factor(6)
(7) 48. Expression in response to 0.15,0.5,1.5,5,15.8,50,158,500 nM alpha-factor(7)
(8) 48. Expression in response to 0.15,0.5,1.5,5,15.8,50,158,500 nM alpha-factor(8)
(9) 49. Expression in response to 50 nM alpha-factor: 0,15,30,45,60,90,120 min(1)
(10) 49. Expression in response to 50 nM alpha-factor: 0,15,30,45,60,90,120 min(2)
(11) 49. Expression in response to 50 nM alpha-factor: 0,15,30,45,60,90,120 min(3)
(12) 49. Expression in response to 50 nM alpha-factor: 0,15,30,45,60,90,120 min(4)
(13) 49. Expression in response to 50 nM alpha-factor: 0,15,30,45,60,90,120 min(6)
(14) 49. Expression in response to 50 nM alpha-factor: 0,15,30,45,60,90,120 min(7)
(15) 54. Expression in response to overproduction of Ste5p(1)
(16) 56. Expression in response to overproduction of Ste12p(1)
(17) 406. Rosetta 2000: Expression in response to Terbinafine(1)
(18) 453. Expression in response to gama-ray exposure (30 kilorad) for 60 min(1)
(19) 455. Expression in response to high MNNG (27 microgram/ml) for 60 min(1)
(20) 456. Expression in response to high 4NQO (8 microgram/ml) for 60 min(1)
(21) 533. Brown enviromental changes :1 mM Menadione (10 min)redo(1)
(22) 534. Brown enviromental changes :1 mM Menadione (20 min) redo(1)
(23) 594. Brown enviromental changes :diauxic shift timecourse(1)
(24) 595. Brown enviromental changes :diauxic shift timecourse(1)
(25) wt_plus_gamma_30_min
(26) 100 microM BCS 60 min

STE12 -*-> FUS2 exp. conditions:33

(1) 5. Expression during the cell cycle (alpha factor arrest and release)(1)
(2) 11. Expression during diauxic shift: 9h,11h,13h,15h,17h,19h,21h(3)
(3) 26. Fink: Expression in diploid high copy TEC1(1)
(4) 48. Expression in response to 0.15,0.5,1.5,5,15.8,50,158,500 nM alpha-factor(3)
(5) 48. Expression in response to 0.15,0.5,1.5,5,15.8,50,158,500 nM alpha-factor(4)
(6) 48. Expression in response to 0.15,0.5,1.5,5,15.8,50,158,500 nM alpha-factor(5)
(7) 48. Expression in response to 0.15,0.5,1.5,5,15.8,50,158,500 nM alpha-factor(6)
(8) 48. Expression in response to 0.15,0.5,1.5,5,15.8,50,158,500 nM alpha-factor(7)
(9) 48. Expression in response to 0.15,0.5,1.5,5,15.8,50,158,500 nM alpha-factor(8)
(10) 49. Expression in response to 50 nM alpha-factor: 0,15,30,45,60,90,120 min(1)
(11) 49. Expression in response to 50 nM alpha-factor: 0,15,30,45,60,90,120 min(2)
(12) 49. Expression in response to 50 nM alpha-factor: 0,15,30,45,60,90,120 min(3)
(13) 49. Expression in response to 50 nM alpha-factor: 0,15,30,45,60,90,120 min(4)
(14) 49. Expression in response to 50 nM alpha-factor: 0,15,30,45,60,90,120 min(5)
(15) 49. Expression in response to 50 nM alpha-factor: 0,15,30,45,60,90,120 min(6)
(16) 49. Expression in response to 50 nM alpha-factor: 0,15,30,45,60,90,120 min(7)
(17) 53. Expression in response to overproduction of Ste4p(1)
(18) 54. Expression in response to overproduction of Ste5p(1)
(19) 55. Expression in response to overproduction of Ste11p(1)
(20) 56. Expression in response to overproduction of Ste12p(1)
(21) 395. Rosetta 2000: Expression in response to 2-deoxy-D-glucose(1)
(22) 404. Rosetta 2000: Expression in response to MMS(1)
(23) 407. Rosetta 2000: Expression in response to Tunicamycin(1)
(24) 563. Brown enviromental changes :1.5 mM diamide (60 min)(1)
(25) 572. Brown enviromental changes :Hypo-osmotic shock - 5 min(1)
(26) 595. Brown enviromental changes :diauxic shift timecourse(1)
(27) 681. Expression in response to 0.4M NaCl for 10 min in wild type(1)
(28) 685. Expression in response to 0.8M NaCl for 20 min in wild type(1)
(29) (Rich Media 2% Glucose YPD-185588) wt 5mM aF, 30 min.
(30) (Rich Media 2% Glucose YPD-185769) wt 5mM aF, 30 min.
(31) Rich Media 2% Glucose YPD-Average wt 5mM aF, 30 min.
(32) (Var.) Rich Media 2% Glucose YPD-Average wt 5mM aF, 30 min.
(33) 100 microM BCS 30 min

STE12 -*-> GPA1 exp. conditions:33

(1) 5. Expression during the cell cycle (alpha factor arrest and release)(1)
(2) 5. Expression during the cell cycle (alpha factor arrest and release)(12)
(3) 5. Expression during the cell cycle (alpha factor arrest and release)(13)
(4) 6. Expression during the cell cycle (cdc15 arrest and release)(23)
(5) 8. Expression during the cell cycle (cell size selection and release)(4)
(6) 8. Expression during the cell cycle (cell size selection and release)(14)
(7) 48. Expression in response to 0.15,0.5,1.5,5,15.8,50,158,500 nM alpha-factor(3)
(8) 48. Expression in response to 0.15,0.5,1.5,5,15.8,50,158,500 nM alpha-factor(4)
(9) 48. Expression in response to 0.15,0.5,1.5,5,15.8,50,158,500 nM alpha-factor(5)
(10) 48. Expression in response to 0.15,0.5,1.5,5,15.8,50,158,500 nM alpha-factor(6)
(11) 48. Expression in response to 0.15,0.5,1.5,5,15.8,50,158,500 nM alpha-factor(7)
(12) 48. Expression in response to 0.15,0.5,1.5,5,15.8,50,158,500 nM alpha-factor(8)
(13) 49. Expression in response to 50 nM alpha-factor: 0,15,30,45,60,90,120 min(1)
(14) 49. Expression in response to 50 nM alpha-factor: 0,15,30,45,60,90,120 min(2)
(15) 49. Expression in response to 50 nM alpha-factor: 0,15,30,45,60,90,120 min(3)
(16) 49. Expression in response to 50 nM alpha-factor: 0,15,30,45,60,90,120 min(4)
(17) 49. Expression in response to 50 nM alpha-factor: 0,15,30,45,60,90,120 min(5)
(18) 49. Expression in response to 50 nM alpha-factor: 0,15,30,45,60,90,120 min(6)
(19) 49. Expression in response to 50 nM alpha-factor: 0,15,30,45,60,90,120 min(7)
(20) 53. Expression in response to overproduction of Ste4p(1)
(21) 54. Expression in response to overproduction of Ste5p(1)
(22) 55. Expression in response to overproduction of Ste11p(1)
(23) 56. Expression in response to overproduction of Ste12p(1)
(24) 487. Expression in response to sorbitol: 15 30 45 90 120 min(4)
(25) 487. Expression in response to sorbitol: 15 30 45 90 120 min(5)
(26) 564. Brown enviromental changes :1.5 mM diamide (90 min)(1)
(27) 565. Brown enviromental changes :1M sorbitol - 5 min(1)
(28) 567. Brown enviromental changes :1M sorbitol - 30 min(1)
(29) 570. Brown enviromental changes :1M sorbitol - 90 min(1)
(30) 685. Expression in response to 0.8M NaCl for 20 min in wild type(1)
(31) (Rich Media 2% Glucose YPD-185588) wt 5mM aF, 30 min.
(32) (Rich Media 2% Glucose YPD-185769) wt 5mM aF, 30 min.
(33) Rich Media 2% Glucose YPD-Average wt 5mM aF, 30 min.

STE12 -*-> INP52 exp. conditions:18

(1) 5. Expression during the cell cycle (alpha factor arrest and release)(1)
(2) 6. Expression during the cell cycle (cdc15 arrest and release)(14)
(3) 7. Expression during the cell Cycle (cdc28)(5)
(4) 8. Expression during the cell cycle (cell size selection and release)(7)
(5) 48. Expression in response to 0.15,0.5,1.5,5,15.8,50,158,500 nM alpha-factor(5)
(6) 48. Expression in response to 0.15,0.5,1.5,5,15.8,50,158,500 nM alpha-factor(6)

(7) 48. Expression in response to 0.15,0.5,1.5,5,15.8,50,158,500 nM alpha-factor(7)
(8) 48. Expression in response to 0.15,0.5,1.5,5,15.8,50,158,500 nM alpha-factor(8)
(9) 49. Expression in response to 50 nM alpha-factor: 0,15,30,45,60,90,120 min(1)
(10) 49. Expression in response to 50 nM alpha-factor: 0,15,30,45,60,90,120 min(3)
(11) 49. Expression in response to 50 nM alpha-factor: 0,15,30,45,60,90,120 min(4)
(12) 49. Expression in response to 50 nM alpha-factor: 0,15,30,45,60,90,120 min(5)
(13) 49. Expression in response to 50 nM alpha-factor: 0,15,30,45,60,90,120 min(6)
(14) 49. Expression in response to 50 nM alpha-factor: 0,15,30,45,60,90,120 min(7)
(15) 53. Expression in response to overproduction of Ste4p(1)
(16) 56. Expression in response to overproduction of Ste12p(1)
(17) 502. Brown enviromental changes :Heat Shock 060 minutes hs-2(1)
(18) MAC1-up (B)

STE12 -*-> KAR4 exp. conditions:33

(1) 5. Expression during the cell cycle (alpha factor arrest and release)(1)
(2) 11. Expression during diauxic shift: 9h,11h,13h,15h,17h,19h,21h(3)
(3) 26. Fink: Expression in diploid high copy TEC1(1)
(4) 48. Expression in response to 0.15,0.5,1.5,5,15.8,50,158,500 nM alpha-factor(3)
(5) 48. Expression in response to 0.15,0.5,1.5,5,15.8,50,158,500 nM alpha-factor(4)
(6) 48. Expression in response to 0.15,0.5,1.5,5,15.8,50,158,500 nM alpha-factor(5)
(7) 48. Expression in response to 0.15,0.5,1.5,5,15.8,50,158,500 nM alpha-factor(6)
(8) 48. Expression in response to 0.15,0.5,1.5,5,15.8,50,158,500 nM alpha-factor(7)
(9) 48. Expression in response to 0.15,0.5,1.5,5,15.8,50,158,500 nM alpha-factor(8)
(10) 49. Expression in response to 50 nM alpha-factor: 0,15,30,45,60,90,120 min(1)
(11) 49. Expression in response to 50 nM alpha-factor: 0,15,30,45,60,90,120 min(2)
(12) 49. Expression in response to 50 nM alpha-factor: 0,15,30,45,60,90,120 min(3)
(13) 49. Expression in response to 50 nM alpha-factor: 0,15,30,45,60,90,120 min(4)
(14) 49. Expression in response to 50 nM alpha-factor: 0,15,30,45,60,90,120 min(5)
(15) 49. Expression in response to 50 nM alpha-factor: 0,15,30,45,60,90,120 min(6)
(16) 49. Expression in response to 50 nM alpha-factor: 0,15,30,45,60,90,120 min(7)
(17) 53. Expression in response to overproduction of Ste4p(1)
(18) 54. Expression in response to overproduction of Ste5p(1)
(19) 55. Expression in response to overproduction of Ste11p(1)
(20) 56. Expression in response to overproduction of Ste12p(1)
(21) 395. Rosetta 2000: Expression in response to 2-deoxy-D-glucose(1)
(22) 404. Rosetta 2000: Expression in response to MMS(1)
(23) 407. Rosetta 2000: Expression in response to Tunicamycin(1)
(24) 563. Brown enviromental changes :1.5 mM diamide (60 min)(1)
(25) 572. Brown enviromental changes :Hypo-osmotic shock - 5 min(1)
(26) 595. Brown enviromental changes :diauxic shift timecourse(1)
(27) 681. Expression in response to 0.4M NaCl for 10 min in wild type(1)
(28) 685. Expression in response to 0.8M NaCl for 20 min in wild type(1)
(29) (Rich Media 2% Glucose YPD-185588) wt 5mM aF, 30 min.
(30) (Rich Media 2% Glucose YPD-185769) wt 5mM aF, 30 min.
(31) Rich Media 2% Glucose YPD-Average wt 5mM aF, 30 min.
(32) (Var.) Rich Media 2% Glucose YPD-Average wt 5mM aF, 30 min.
(33) 100 microM BCS 30 min

STE12 --> TEC1 exp. conditions:38

(1) 5. Expression during the cell cycle (alpha factor arrest and release)(1)
(2) 5. Expression during the cell cycle (alpha factor arrest and release)(10)
(3) 5. Expression during the cell cycle (alpha factor arrest and release)(11)
(4) 5. Expression during the cell cycle (alpha factor arrest and release)(12)
(5) 6. Expression during the cell cycle (cdc15 arrest and release)(20)
(6) 6. Expression during the cell cycle (cdc15 arrest and release)(23)
(7) 7. Expression during the cell Cycle (cdc28)(1)
(8) 7. Expression during the cell Cycle (cdc28)(17)
(9) 8. Expression during the cell cycle (cell size selection and release)(12)
(10) 8. Expression during the cell cycle (cell size selection and release)(14)
(11) 48. Expression in response to 0.15,0.5,1.5,5,15.8,50,158,500 nM alpha-factor(3)
(12) 48. Expression in response to 0.15,0.5,1.5,5,15.8,50,158,500 nM alpha-factor(4)
(13) 48. Expression in response to 0.15,0.5,1.5,5,15.8,50,158,500 nM alpha-factor(5)
(14) 48. Expression in response to 0.15,0.5,1.5,5,15.8,50,158,500 nM alpha-factor(6)
(15) 48. Expression in response to 0.15,0.5,1.5,5,15.8,50,158,500 nM alpha-factor(7)
(16) 48. Expression in response to 0.15,0.5,1.5,5,15.8,50,158,500 nM alpha-factor(8)
(17) 49. Expression in response to 50 nM alpha-factor: 0,15,30,45,60,90,120 min(1)
(18) 49. Expression in response to 50 nM alpha-factor: 0,15,30,45,60,90,120 min(2)
(19) 49. Expression in response to 50 nM alpha-factor: 0,15,30,45,60,90,120 min(3)
(20) 49. Expression in response to 50 nM alpha-factor: 0,15,30,45,60,90,120 min(4)
(21) 49. Expression in response to 50 nM alpha-factor: 0,15,30,45,60,90,120 min(5)
(22) 49. Expression in response to 50 nM alpha-factor: 0,15,30,45,60,90,120 min(6)
(23) 49. Expression in response to 50 nM alpha-factor: 0,15,30,45,60,90,120 min(7)
(24) 53. Expression in response to overproduction of Ste4p(1)
(25) 54. Expression in response to overproduction of Ste5p(1)
(26) 55. Expression in response to overproduction of Ste11p(1)
(27) 56. Expression in response to overproduction of Ste12p(1)
(28) 405. Rosetta 2000: Expression in response to Nikkomycin Z(1)
(29) 565. Brown enviromental changes :1M sorbitol - 5 min(1)
(30) 674. Expression in response to oligomycin 120min(1)
(31) 681. Expression in response to 0.4M NaCl for 10 min in wild type(1)
(32) 683. Expression in response to 0.4M NaCl for 20 min in wild type(1)
(33) 685. Expression in response to 0.8M NaCl for 20 min in wild type(1)
(34) (Rich Media 2% Glucose YPD-185588) wt 5mM aF, 30 min.
(35) (Rich Media 2% Glucose YPD-185769) wt 5mM aF, 30 min.
(36) Rich Media 2% Glucose YPD-Average wt 5mM aF, 30 min.
(37) DES460 (wt) - mock irradiation - 5 min
(38) DES460 (wt) - mock irradiation - 30 min

SWI4 --> CLN1 exp. conditions:31

(1) 1. Cell cycle: Expression in response to Cln3p (set 1)(1)
(2) 2. Cell Cycle: Expression in response to Cln3p (set 2)(1)
(3) 5. Expression during the cell cycle (alpha factor arrest and release)(3)
(4) 5. Expression during the cell cycle (alpha factor arrest and release)(4)
(5) 5. Expression during the cell cycle (alpha factor arrest and release)(5)
(6) 5. Expression during the cell cycle (alpha factor arrest and release)(11)
(7) 5. Expression during the cell cycle (alpha factor arrest and release)(12)
(8) 5. Expression during the cell cycle (alpha factor arrest and release)(13)
(9) 6. Expression during the cell cycle (cdc15 arrest and release)(2)
(10) 6. Expression during the cell cycle (cdc15 arrest and release)(3)
(11) 6. Expression during the cell cycle (cdc15 arrest and release)(10)
(12) 6. Expression during the cell cycle (cdc15 arrest and release)(11)
(13) 6. Expression during the cell cycle (cdc15 arrest and release)(12)
(14) 6. Expression during the cell cycle (cdc15 arrest and release)(13)

(15) 6. Expression during the cell cycle (cdc15 arrest and release)(14)
(16) 6. Expression during the cell cycle (cdc15 arrest and release)(17)
(17) 7. Expression during the cell Cycle (cdc28)(3)
(18) 7. Expression during the cell Cycle (cdc28)(11)
(19) 7. Expression during the cell Cycle (cdc28)(12)
(20) 8. Expression during the cell cycle (cell size selection and release)(6)
(21) 8. Expression during the cell cycle (cell size selection and release)(7)
(22) 8. Expression during the cell cycle (cell size selection and release)(8)
(23) 8. Expression during the cell cycle (cell size selection and release)(9)
(24) 8. Expression during the cell cycle (cell size selection and release)(10)
(25) 8. Expression during the cell cycle (cell size selection and release)(14)
(26) 386. Rosetta 2000: Expression in cells with CDC42 under tet promoter(1)
(27) 482. Expression in response to acid: 10,20,40,60,80,100 min(4)
(28) 482. Expression in response to acid: 10,20,40,60,80,100 min(6)
(29) 571. Brown enviromental changes :1M sorbitol - 120 min(1)
(30) DES460 (wt) - mock irradiation - 30 min
(31) DES460 (wt) - mock irradiation - 60 min

SWI4 -*-> GIN4 exp. conditions:26

(1) 1. Cell cycle: Expression in response to Cln3p (set 1)(1)
(2) 5. Expression during the cell cycle (alpha factor arrest and release)(3)
(3) 5. Expression during the cell cycle (alpha factor arrest and release)(4)
(4) 5. Expression during the cell cycle (alpha factor arrest and release)(5)
(5) 5. Expression during the cell cycle (alpha factor arrest and release)(11)
(6) 5. Expression during the cell cycle (alpha factor arrest and release)(12)
(7) 5. Expression during the cell cycle (alpha factor arrest and release)(13)
(8) 6. Expression during the cell cycle (cdc15 arrest and release)(12)
(9) 6. Expression during the cell cycle (cdc15 arrest and release)(23)
(10) 7. Expression during the cell Cycle (cdc28)(3)
(11) 7. Expression during the cell Cycle (cdc28)(10)
(12) 7. Expression during the cell Cycle (cdc28)(11)
(13) 389. Rosetta 2000: Expression in cells with HMG2 under tet promoter(1)
(14) 448. Expression in response to 0.1% MMS for 60 min(1)
(15) 482. Expression in response to acid: 10,20,40,60,80,100 min(4)
(16) 482. Expression in response to acid: 10,20,40,60,80,100 min(6)
(17) 483. Expression in response to alkali: 10,20,40,60,80,100 min(6)
(18) 486. Expression in response to NaCl: 15 30 45 60 120 min(4)
(19) 486. Expression in response to NaCl: 15 30 45 60 120 min(5)
(20) 487. Expression in response to sorbitol: 15 30 45 90 120 min(2)
(21) 487. Expression in response to sorbitol: 15 30 45 90 120 min(3)
(22) 487. Expression in response to sorbitol: 15 30 45 90 120 min(4)
(23) 487. Expression in response to sorbitol: 15 30 45 90 120 min(5)
(24) DES460 (wt) - mock irradiation - 5 min
(25) DES460 (wt) - mock irradiation - 30 min
(26) DES460 (wt) - mock irradiation - 60 min

SWI4 -*-> HCM1 exp. conditions:31

(1) 1. Cell cycle: Expression in response to Cln3p (set 1)(1)
(2) 2. Cell Cycle: Expression in response to Cln3p (set 2)(1)
(3) 5. Expression during the cell cycle (alpha factor arrest and release)(3)
(4) 5. Expression during the cell cycle (alpha factor arrest and release)(4)
(5) 5. Expression during the cell cycle (alpha factor arrest and release)(5)
(6) 5. Expression during the cell cycle (alpha factor arrest and release)(11)
(7) 5. Expression during the cell cycle (alpha factor arrest and release)(12)
(8) 5. Expression during the cell cycle (alpha factor arrest and release)(13)
(9) 6. Expression during the cell cycle (cdc15 arrest and release)(2)
(10) 6. Expression during the cell cycle (cdc15 arrest and release)(3)
(11) 6. Expression during the cell cycle (cdc15 arrest and release)(10)
(12) 6. Expression during the cell cycle (cdc15 arrest and release)(11)
(13) 6. Expression during the cell cycle (cdc15 arrest and release)(12)
(14) 6. Expression during the cell cycle (cdc15 arrest and release)(13)
(15) 6. Expression during the cell cycle (cdc15 arrest and release)(14)
(16) 6. Expression during the cell cycle (cdc15 arrest and release)(17)
(17) 7. Expression during the cell Cycle (cdc28)(3)
(18) 7. Expression during the cell Cycle (cdc28)(11)
(19) 7. Expression during the cell Cycle (cdc28)(12)
(20) 8. Expression during the cell cycle (cell size selection and release)(6)
(21) 8. Expression during the cell cycle (cell size selection and release)(7)
(22) 8. Expression during the cell cycle (cell size selection and release)(8)
(23) 8. Expression during the cell cycle (cell size selection and release)(9)
(24) 8. Expression during the cell cycle (cell size selection and release)(10)
(25) 8. Expression during the cell cycle (cell size selection and release)(14)
(26) 386. Rosetta 2000: Expression in cells with CDC42 under tet promoter(1)
(27) 482. Expression in response to acid: 10,20,40,60,80,100 min(4)
(28) 482. Expression in response to acid: 10,20,40,60,80,100 min(6)
(29) 571. Brown enviromental changes :1M sorbitol - 120 min(1)
(30) DES460 (wt) - mock irradiation - 30 min
(31) DES460 (wt) - mock irradiation - 60 min

SWI5 --> ASH1 exp. conditions:28

(1) 3. Cell Cycle: Expression in response to Clb2p (set 1, 40 min)(1)
(2) 4. Cell Cycle: Expression in response to Clb2p (set 2, 30 min)(1)
(3) 5. Expression during the cell cycle (alpha factor arrest and release)(10)
(4) 5. Expression during the cell cycle (alpha factor arrest and release)(11)
(5) 5. Expression during the cell cycle (alpha factor arrest and release)(12)
(6) 5. Expression during the cell cycle (alpha factor arrest and release)(13)
(7) 5. Expression during the cell cycle (alpha factor arrest and release)(16)
(8) 5. Expression during the cell cycle (alpha factor arrest and release)(18)
(9) 6. Expression during the cell cycle (cdc15 arrest and release)(1)
(10) 6. Expression during the cell cycle (cdc15 arrest and release)(2)
(11) 6. Expression during the cell cycle (cdc15 arrest and release)(10)
(12) 6. Expression during the cell cycle (cdc15 arrest and release)(11)
(13) 6. Expression during the cell cycle (cdc15 arrest and release)(12)
(14) 6. Expression during the cell cycle (cdc15 arrest and release)(20)
(15) 6. Expression during the cell cycle (cdc15 arrest and release)(22)
(16) 6. Expression during the cell cycle (cdc15 arrest and release)(23)
(17) 7. Expression during the cell Cycle (cdc28)(9)
(18) 7. Expression during the cell Cycle (cdc28)(10)
(19) 7. Expression during the cell Cycle (cdc28)(11)
(20) 7. Expression during the cell Cycle (cdc28)(12)
(21) 7. Expression during the cell Cycle (cdc28)(16)
(22) 7. Expression during the cell Cycle (cdc28)(17)
(23) 8. Expression during the cell cycle (cell size selection and release)(11)

(24) 8. Expression during the cell cycle (cell size selection and release)(12)
(25) 8. Expression during the cell cycle (cell size selection and release)(13)
(26) 8. Expression during the cell cycle (cell size selection and release)(14)
(27) 487. Expression in response to sorbitol: 15 30 45 90 120 min(5)
(28) 556. Brown enviromental changes :dtt 480 min dtt-2(1)

SWI5 -*-| BUD9 exp. conditions:18

(1) 5. Expression during the cell cycle (alpha factor arrest and release)(10)
(2) 5. Expression during the cell cycle (alpha factor arrest and release)(11)
(3) 5. Expression during the cell cycle (alpha factor arrest and release)(12)
(4) 5. Expression during the cell cycle (alpha factor arrest and release)(13)
(5) 6. Expression during the cell cycle (cdc15 arrest and release)(1)
(6) 6. Expression during the cell cycle (cdc15 arrest and release)(2)
(7) 6. Expression during the cell cycle (cdc15 arrest and release)(10)
(8) 6. Expression during the cell cycle (cdc15 arrest and release)(11)
(9) 6. Expression during the cell cycle (cdc15 arrest and release)(12)
(10) 6. Expression during the cell cycle (cdc15 arrest and release)(22)
(11) 6. Expression during the cell cycle (cdc15 arrest and release)(23)
(12) 7. Expression during the cell Cycle (cdc28)(10)
(13) 7. Expression during the cell Cycle (cdc28)(11)
(14) 7. Expression during the cell Cycle (cdc28)(12)
(15) 7. Expression during the cell Cycle (cdc28)(17)
(16) 8. Expression during the cell cycle (cell size selection and release)(12)
(17) 8. Expression during the cell cycle (cell size selection and release)(14)
(18) 556. Brown enviromental changes :dtt 480 min dtt-2(1)

SWI6 --> CLB5 exp. conditions:45

(1) 1. Cell cycle: Expression in response to Cln3p (set 1)(1)
(2) 2. Cell Cycle: Expression in response to Cln3p (set 2)(1)
(3) 5. Expression during the cell cycle (alpha factor arrest and release)(3)
(4) 5. Expression during the cell cycle (alpha factor arrest and release)(4)
(5) 5. Expression during the cell cycle (alpha factor arrest and release)(5)
(6) 5. Expression during the cell cycle (alpha factor arrest and release)(11)
(7) 5. Expression during the cell cycle (alpha factor arrest and release)(12)
(8) 5. Expression during the cell cycle (alpha factor arrest and release)(13)
(9) 6. Expression during the cell cycle (cdc15 arrest and release)(2)
(10) 6. Expression during the cell cycle (cdc15 arrest and release)(3)
(11) 6. Expression during the cell cycle (cdc15 arrest and release)(4)
(12) 6. Expression during the cell cycle (cdc15 arrest and release)(11)
(13) 6. Expression during the cell cycle (cdc15 arrest and release)(12)
(14) 6. Expression during the cell cycle (cdc15 arrest and release)(13)
(15) 6. Expression during the cell cycle (cdc15 arrest and release)(14)
(16) 7. Expression during the cell Cycle (cdc28)(3)
(17) 7. Expression during the cell Cycle (cdc28)(4)
(18) 7. Expression during the cell Cycle (cdc28)(11)
(19) 7. Expression during the cell Cycle (cdc28)(12)
(20) 8. Expression during the cell cycle (cell size selection and release)(6)
(21) 8. Expression during the cell cycle (cell size selection and release)(7)
(22) 8. Expression during the cell cycle (cell size selection and release)(8)
(23) 8. Expression during the cell cycle (cell size selection and release)(10)
(24) 11. Expression during diauxic shift: 9h,11h,13h,15h,17h,19h,21h(6)
(25) PHO81c vs WT exp1(1)
(26) 452. Expression in response to low 4NQO (2 microgram/ml) for 60 min(1)
(27) 453. Expression in response to gama-ray exposure (30 kilorad) for 60 min(1)
(28) 456. Expression in response to high 4NQO (8 microgram/ml) for 60 min(1)
(29) 481. Expression in response to heat shock: 15,30,45,60,120 min(4)
(30) 482. Expression in response to acid: 10,20,40,60,80,100 min(4)
(31) 502. Brown enviromental changes :Heat Shock 060 minutes hs-2(1)
(32) 569. Brown enviromental changes :1M sorbitol - 60 min(1)
(33) 571. Brown enviromental changes :1M sorbitol - 120 min(1)
(34) 584. Brown enviromental changes :Nitrogen Depletion 1 h(1)
(35) 585. Brown enviromental changes :Nitrogen Depletion 2 h(1)
(36) 586. Brown enviromental changes :Nitrogen Depletion 4 h(1)
(37) 598. Brown enviromental changes :diauxic shift timecourse(1)
(38) 601. Brown enviromental changes :YPD 2 h ypd-2(1)
(39) 672. Expression in response to carbonyl cyanide m-chlorophenylhydrazone (CCCP) 90min(1)
(40) 681. Expression in response to 0.4M NaCl for 10 min in wild type(1)
(41) (Var.) Rich Media 2% Glucose YPD-Average wt 5mM aF, 30 min.
(42) DES460 (wt) - mock irradiation - 30 min
(43) DES460 (wt) - mock irradiation - 60 min
(44) wt-gal
(45) wt+gal

SWI6 -*-> HCM1 exp. conditions:26

(1) 1. Cell cycle: Expression in response to Cln3p (set 1)(1)
(2) 2. Cell Cycle: Expression in response to Cln3p (set 2)(1)
(3) 5. Expression during the cell cycle (alpha factor arrest and release)(3)
(4) 5. Expression during the cell cycle (alpha factor arrest and release)(4)
(5) 5. Expression during the cell cycle (alpha factor arrest and release)(5)
(6) 5. Expression during the cell cycle (alpha factor arrest and release)(11)
(7) 5. Expression during the cell cycle (alpha factor arrest and release)(12)
(8) 5. Expression during the cell cycle (alpha factor arrest and release)(13)
(9) 6. Expression during the cell cycle (cdc15 arrest and release)(2)
(10) 6. Expression during the cell cycle (cdc15 arrest and release)(3)
(11) 6. Expression during the cell cycle (cdc15 arrest and release)(11)
(12) 6. Expression during the cell cycle (cdc15 arrest and release)(12)
(13) 6. Expression during the cell cycle (cdc15 arrest and release)(13)
(14) 6. Expression during the cell cycle (cdc15 arrest and release)(14)
(15) 7. Expression during the cell Cycle (cdc28)(3)
(16) 7. Expression during the cell Cycle (cdc28)(4)
(17) 7. Expression during the cell Cycle (cdc28)(11)
(18) 7. Expression during the cell Cycle (cdc28)(12)
(19) 8. Expression during the cell cycle (cell size selection and release)(6)
(20) 8. Expression during the cell cycle (cell size selection and release)(7)
(21) 8. Expression during the cell cycle (cell size selection and release)(8)
(22) 8. Expression during the cell cycle (cell size selection and release)(10)
(23) 482. Expression in response to acid: 10,20,40,60,80,100 min(4)
(24) 502. Brown enviromental changes :Heat Shock 060 minutes hs-2(1)
(25) 569. Brown enviromental changes :1M sorbitol - 60 min(1)
(26) 571. Brown enviromental changes :1M sorbitol - 120 min(1)

TEC1 -*-> GFA1 exp. conditions:29

(1) 5. Expression during the cell cycle (alpha factor arrest and release)(1)
(2) 6. Expression during the cell cycle (cdc15 arrest and release)(22)
(3) 6. Expression during the cell cycle (cdc15 arrest and release)(23)
(4) 6. Expression during the cell cycle (cdc15 arrest and release)(24)
(5) 7. Expression during the cell Cycle (cdc28)(17)
(6) 26. Fink: Expression in diploid high copy TEC1(1)
(7) 48. Expression in response to 0.15,0.5,1.5,5,15.8,50,158,500 nM alpha-factor(4)
(8) 48. Expression in response to 0.15,0.5,1.5,5,15.8,50,158,500 nM alpha-factor(5)
(9) 48. Expression in response to 0.15,0.5,1.5,5,15.8,50,158,500 nM alpha-factor(6)
(10) 49. Expression in response to 50 nM alpha-factor: 0,15,30,45,60,90,120 min(1)
(11) 49. Expression in response to 50 nM alpha-factor: 0,15,30,45,60,90,120 min(2)
(12) 49. Expression in response to 50 nM alpha-factor: 0,15,30,45,60,90,120 min(3)
(13) 49. Expression in response to 50 nM alpha-factor: 0,15,30,45,60,90,120 min(7)
(14) 53. Expression in response to overproduction of Ste4p(1)
(15) 54. Expression in response to overproduction of Ste5p(1)
(16) 332. Rosetta 2000: Expression in cells with CMD1 under tet promoter(1)
(17) 395. Rosetta 2000: Expression in response to 2-deoxy-D-glucose(1)
(18) 400. Rosetta 2000: Expression in response to Glucosamine(1)
(19) 407. Rosetta 2000: Expression in response to Tunicamycin(1)
(20) 493. Brown enviromental changes :Heat Shock 40 minutes hs-1(1)
(21) 523. Brown enviromental changes :constant 0.32 mM H2O2 (10 min) redo(1)
(22) 561. Brown enviromental changes :1.5 mM diamide (40 min)(1)
(23) 562. Brown enviromental changes :1.5 mM diamide (50 min)(1)
(24) 564. Brown enviromental changes :1.5 mM diamide (90 min)(1)
(25) 572. Brown enviromental changes :Hypo-osmotic shock - 5 min(1)
(26) (Rich Media 2% Glucose YPD-185588) wt 5mM aF, 30 min.
(27) (Rich Media 2% Glucose YPD-185769) wt 5mM aF, 30 min.
(28) Rich Media 2% Glucose YPD-Average wt 5mM aF, 30 min.
(29) wt+gal

TEC1 -*-> GIC2 exp. conditions:26

(1) 5. Expression during the cell cycle (alpha factor arrest and release)(1)
(2) 5. Expression during the cell cycle (alpha factor arrest and release)(13)
(3) 5. Expression during the cell cycle (alpha factor arrest and release)(15)
(4) 7. Expression during the cell Cycle (cdc28)(11)
(5) 8. Expression during the cell cycle (cell size selection and release)(8)
(6) 8. Expression during the cell cycle (cell size selection and release)(9)
(7) 8. Expression during the cell cycle (cell size selection and release)(10)
(8) 8. Expression during the cell cycle (cell size selection and release)(12)
(9) 11. Expression during diauxic shift: 9h,11h,13h,15h,17h,19h,21h(3)
(10) 26. Fink: Expression in diploid high copy TEC1(1)
(11) 48. Expression in response to 0.15,0.5,1.5,5,15.8,50,158,500 nM alpha-factor(5)
(12) 48. Expression in response to 0.15,0.5,1.5,5,15.8,50,158,500 nM alpha-factor(6)
(13) 48. Expression in response to 0.15,0.5,1.5,5,15.8,50,158,500 nM alpha-factor(8)
(14) 49. Expression in response to 50 nM alpha-factor: 0,15,30,45,60,90,120 min(1)
(15) 49. Expression in response to 50 nM alpha-factor: 0,15,30,45,60,90,120 min(2)
(16) 49. Expression in response to 50 nM alpha-factor: 0,15,30,45,60,90,120 min(3)
(17) 49. Expression in response to 50 nM alpha-factor: 0,15,30,45,60,90,120 min(7)
(18) 53. Expression in response to overproduction of Ste4p(1)
(19) 407. Rosetta 2000: Expression in response to Tunicamycin(1)
(20) 523. Brown enviromental changes :constant 0.32 mM H2O2 (10 min) redo(1)
(21) 595. Brown enviromental changes :diauxic shift timecourse(1)
(22) (Rich Media 2% Glucose YPD-185769) wt 5mM aF, 30 min.
(23) Rich Media 2% Glucose YPD-Average wt 5mM aF, 30 min.
(24) wt_plus_gamma_10_min
(25) DES460 (wt) - mock irradiation - 30 min
(26) 100 microM BCS 60 min

TEC1 -*-> PCL2 exp. conditions:26

(1) 5. Expression during the cell cycle (alpha factor arrest and release)(1)
(2) 5. Expression during the cell cycle (alpha factor arrest and release)(13)
(3) 6. Expression during the cell cycle (cdc15 arrest and release)(10)
(4) 6. Expression during the cell cycle (cdc15 arrest and release)(22)
(5) 6. Expression during the cell cycle (cdc15 arrest and release)(23)
(6) 6. Expression during the cell cycle (cdc15 arrest and release)(24)
(7) 7. Expression during the cell Cycle (cdc28)(17)
(8) 8. Expression during the cell cycle (cell size selection and release)(4)
(9) 26. Fink: Expression in diploid high copy TEC1(1)
(10) 48. Expression in response to 0.15,0.5,1.5,5,15.8,50,158,500 nM alpha-factor(3)
(11) 48. Expression in response to 0.15,0.5,1.5,5,15.8,50,158,500 nM alpha-factor(4)
(12) 48. Expression in response to 0.15,0.5,1.5,5,15.8,50,158,500 nM alpha-factor(5)
(13) 48. Expression in response to 0.15,0.5,1.5,5,15.8,50,158,500 nM alpha-factor(6)
(14) 49. Expression in response to 50 nM alpha-factor: 0,15,30,45,60,90,120 min(1)
(15) 49. Expression in response to 50 nM alpha-factor: 0,15,30,45,60,90,120 min(2)
(16) 49. Expression in response to 50 nM alpha-factor: 0,15,30,45,60,90,120 min(3)
(17) 49. Expression in response to 50 nM alpha-factor: 0,15,30,45,60,90,120 min(7)
(18) 55. Expression in response to overproduction of Ste11p(1)
(19) 56. Expression in response to overproduction of Ste12p(1)
(20) 407. Rosetta 2000: Expression in response to Tunicamycin(1)
(21) 487. Expression in response to sorbitol: 15 30 45 90 120 min(4)
(22) 487. Expression in response to sorbitol: 15 30 45 90 120 min(5)
(23) 523. Brown enviromental changes :constant 0.32 mM H2O2 (10 min) redo(1)
(24) 562. Brown enviromental changes :1.5 mM diamide (50 min)(1)
(25) 564. Brown enviromental changes :1.5 mM diamide (90 min)(1)
(26) 570. Brown enviromental changes :1M sorbitol - 90 min(1)

TEC1 --> STE12 exp. conditions:26

(1) 5. Expression during the cell cycle (alpha factor arrest and release)(1)
(2) 5. Expression during the cell cycle (alpha factor arrest and release)(13)
(3) 6. Expression during the cell cycle (cdc15 arrest and release)(10)
(4) 6. Expression during the cell cycle (cdc15 arrest and release)(22)
(5) 6. Expression during the cell cycle (cdc15 arrest and release)(23)
(6) 6. Expression during the cell cycle (cdc15 arrest and release)(24)
(7) 7. Expression during the cell Cycle (cdc28)(17)
(8) 8. Expression during the cell cycle (cell size selection and release)(4)
(9) 26. Fink: Expression in diploid high copy TEC1(1)
(10) 48. Expression in response to 0.15,0.5,1.5,5,15.8,50,158,500 nM alpha-factor(3)
(11) 48. Expression in response to 0.15,0.5,1.5,5,15.8,50,158,500 nM alpha-factor(4)
(12) 48. Expression in response to 0.15,0.5,1.5,5,15.8,50,158,500 nM alpha-factor(5)
(13) 48. Expression in response to 0.15,0.5,1.5,5,15.8,50,158,500 nM alpha-factor(6)
(14) 49. Expression in response to 50 nM alpha-factor: 0,15,30,45,60,90,120 min(1)

```
(15) 49. Expression in response to 50 nM alpha-factor: 0,15,30,45,60,90,120 min(2)
(16) 49. Expression in response to 50 nM alpha-factor: 0,15,30,45,60,90,120 min(3)
(17) 49. Expression in response to 50 nM alpha-factor: 0,15,30,45,60,90,120 min(7)
(18) 55. Expression in response to overproduction of Ste11p(1)
(19) 56. Expression in response to overproduction of Ste12p(1)
(20) 407. Rosetta 2000: Expression in response to Tunicamycin(1)
(21) 487. Expression in response to sorbitol: 15 30 45 90 120 min(4)
(22) 487. Expression in response to sorbitol: 15 30 45 90 120 min(5)
(23) 523. Brown enviromental changes :constant 0.32 mM H2O2 (10 min) redo(1)
(24) 562. Brown enviromental changes :1.5 mM diamide (50 min)(1)
(25) 564. Brown enviromental changes :1.5 mM diamide (90 min)(1)
(26) 570. Brown enviromental changes :1M sorbitol - 90 min(1)
```
