# Supplementary material for: Unraveling condition specific gene transcriptional regulatory networks in Saccharomyces cerevisiae
Source: BMC Bioinformatics. 2006 Mar 21;7:165. doi: 10.1186/1471-2105-7-165 (PMC1488875; doi:10.1186/1471-2105-7-165)
Supplement: Additional File 3 — experimental conditions for each link in figure 7. These are the experimental conditions in which the links are likely to be active. [file 1471-2105-7-165-S3.pdf]

Predicted experimental conditions for regulartory links in Figure 5.

Each link in this file (e.g. DAL82 --> DAL1) is followed by a list of the predicted experimental conditions in which it is likely to be active.

The number experimental conditions is indicated next to the link (e.g. DAL82 --> DAL1 exp. conditions:31)
The index of each experimental condition is shown in parenthesis (e.g. (1) 5. Expression during the cell cycle)

The total number of links in this figure is 51.

DAL82 --> DAL1 exp. conditions:31

(1) 5. Expression during the cell cycle (alpha factor arrest and release)(5)
(2) 7. Expression during the cell Cycle (cdc28)(16)
(3) 26. Fink: Expression in diploid high copy TEC1(1)
(4) 49. Expression in response to 50 nM alpha-factor: 0,15,30,45,60,90,120 min(5)
(5) 428. Expression in strain PM38 (wild type) in response to 30 min 50 nM treatment with rapamycin in YPD(1)
(6) 429. Expression in strain YHE711 (wild type) in response to 30 min 50 nM treatment with rapamycin in YPD(1)
(7) 439. Expression in strain Jk9-3da (wild type) in response to 30 min 50 nM treatment with rapamycin in YPD(1)
(8) 442. Expression in strain PM38 (wild type) in response to 30 min 50 nM treatment with rapamycin in YPD(1)
(9) 479. Expression in diploid cells in response to rapamycin (100nM) for: 15min,30min,90min,120min(1)
(10) 479. Expression in diploid cells in response to rapamycin (100nM) for: 15min,30min,90min,120min(2)
(11) 531. Brown enviromental changes :constant 0.32 mM H2O2 (120 min) redo(1)
(12) 569. Brown enviromental changes :1M sorbitol - 60 min(1)
(13) 578. Brown enviromental changes :aa starv 0.5 h(1)
(14) 579. Brown enviromental changes :aa starv 1 h(1)
(15) 580. Brown enviromental changes :aa starv 2 h(1)
(16) 581. Brown enviromental changes :aa starv 4 h(1)
(17) 582. Brown enviromental changes :aa starv 6 h(1)
(18) 583. Brown enviromental changes :Nitrogen Depletion 30 min.(1)
(19) 584. Brown enviromental changes :Nitrogen Depletion 1 h(1)
(20) 585. Brown enviromental changes :Nitrogen Depletion 2 h(1)
(21) 586. Brown enviromental changes :Nitrogen Depletion 4 h(1)
(22) 587. Brown enviromental changes :Nitrogen Depletion 8 h(1)
(23) 588. Brown enviromental changes :Nitrogen Depletion 12 h(1)
(24) 589. Brown enviromental changes :Nitrogen Depletion 1 d(1)
(25) 590. Brown enviromental changes :Nitrogen Depletion 2 d(1)
(26) 591. Brown enviromental changes :Nitrogen Depletion 3 d(1)
(27) 592. Brown enviromental changes :Nitrogen Depletion 5 d(1)
(28) 681. Expression in response to 0.4M NaCl for 10 min in wild type(1)
(29) 100 microM CuSO4 30 min
(30) MAC1-up (C)
(31) wt+gal

DAL82 --> DAL2 exp. conditions:29

(1) 5. Expression during the cell cycle (alpha factor arrest and release)(5)
(2) 6. Expression during the cell cycle (cdc15 arrest and release)(9)
(3) 49. Expression in response to 50 nM alpha-factor: 0,15,30,45,60,90,120 min(5)
(4) 428. Expression in strain PM38 (wild type) in response to 30 min 50 nM treatment with rapamycin in YPD(1)
(5) 429. Expression in strain YHE711 (wild type) in response to 30 min 50 nM treatment with rapamycin in YPD(1)
(6) 439. Expression in strain Jk9-3da (wild type) in response to 30 min 50 nM treatment with rapamycin in YPD(1)
(7) 442. Expression in strain PM38 (wild type) in response to 30 min 50 nM treatment with rapamycin in YPD(1)
(8) 479. Expression in diploid cells in response to rapamycin (100nM) for: 15min,30min,90min,120min(1)
(9) 479. Expression in diploid cells in response to rapamycin (100nM) for: 15min,30min,90min,120min(2)
(10) 531. Brown enviromental changes :constant 0.32 mM H2O2 (120 min) redo(1)
(11) 569. Brown enviromental changes :1M sorbitol - 60 min(1)
(12) 570. Brown enviromental changes :1M sorbitol - 90 min(1)
(13) 578. Brown enviromental changes :aa starv 0.5 h(1)
(14) 579. Brown enviromental changes :aa starv 1 h(1)
(15) 580. Brown enviromental changes :aa starv 2 h(1)
(16) 581. Brown enviromental changes :aa starv 4 h(1)
(17) 582. Brown enviromental changes :aa starv 6 h(1)
(18) 583. Brown enviromental changes :Nitrogen Depletion 30 min.(1)
(19) 584. Brown enviromental changes :Nitrogen Depletion 1 h(1)
(20) 585. Brown enviromental changes :Nitrogen Depletion 2 h(1)
(21) 586. Brown enviromental changes :Nitrogen Depletion 4 h(1)
(22) 587. Brown enviromental changes :Nitrogen Depletion 8 h(1)
(23) 588. Brown enviromental changes :Nitrogen Depletion 12 h(1)
(24) 589. Brown enviromental changes :Nitrogen Depletion 1 d(1)
(25) 590. Brown enviromental changes :Nitrogen Depletion 2 d(1)
(26) 591. Brown enviromental changes :Nitrogen Depletion 3 d(1)
(27) 592. Brown enviromental changes :Nitrogen Depletion 5 d(1)
(28) 100 microM BCS 60 min
(29) wt+gal

DAL82 -*-> DAL3 exp. conditions:21

(1) 49. Expression in response to 50 nM alpha-factor: 0,15,30,45,60,90,120 min(5)
(2) 428. Expression in strain PM38 (wild type) in response to 30 min 50 nM treatment with rapamycin in YPD(1)
(3) 429. Expression in strain YHE711 (wild type) in response to 30 min 50 nM treatment with rapamycin in YPD(1)
(4) 439. Expression in strain Jk9-3da (wild type) in response to 30 min 50 nM treatment with rapamycin in YPD(1)
(5) 442. Expression in strain PM38 (wild type) in response to 30 min 50 nM treatment with rapamycin in YPD(1)
(6) 479. Expression in diploid cells in response to rapamycin (100nM) for: 15min,30min,90min,120min(2)
(7) 578. Brown enviromental changes :aa starv 0.5 h(1)
(8) 579. Brown enviromental changes :aa starv 1 h(1)
(9) 580. Brown enviromental changes :aa starv 2 h(1)
(10) 581. Brown enviromental changes :aa starv 4 h(1)
(11) 582. Brown enviromental changes :aa starv 6 h(1)
(12) 583. Brown enviromental changes :Nitrogen Depletion 30 min.(1)
(13) 584. Brown enviromental changes :Nitrogen Depletion 1 h(1)
(14) 585. Brown enviromental changes :Nitrogen Depletion 2 h(1)
(15) 586. Brown enviromental changes :Nitrogen Depletion 4 h(1)
(16) 587. Brown enviromental changes :Nitrogen Depletion 8 h(1)
(17) 588. Brown enviromental changes :Nitrogen Depletion 12 h(1)
(18) 589. Brown enviromental changes :Nitrogen Depletion 1 d(1)
(19) 590. Brown enviromental changes :Nitrogen Depletion 2 d(1)
(20) 591. Brown enviromental changes :Nitrogen Depletion 3 d(1)
(21) 592. Brown enviromental changes :Nitrogen Depletion 5 d(1)

DAL82 --> DAL4 exp. conditions:28

(1) 49. Expression in response to 50 nM alpha-factor: 0,15,30,45,60,90,120 min(5)
(2) 428. Expression in strain PM38 (wild type) in response to 30 min 50 nM treatment with rapamycin in YPD(1)

(3) 429. Expression in strain YHE711 (wild type) in response to 30 min 50 nM treatment with rapamycin in YPD(1)
(4) 439. Expression in strain Jk9-3da (wild type) in response to 30 min 50 nM treatment with rapamycin in YPD(1)
(5) 442. Expression in strain PM38 (wild type) in response to 30 min 50 nM treatment with rapamycin in YPD(1)
(6) 479. Expression in diploid cells in response to rapamycin (100nM) for: 15min,30min,90min,120min(2)
(7) 531. Brown enviromental changes :constant 0.32 mM H2O2 (120 min) redo(1)
(8) 569. Brown enviromental changes :1M sorbitol - 60 min(1)
(9) 578. Brown enviromental changes :aa starv 0.5 h(1)
(10) 579. Brown enviromental changes :aa starv 1 h(1)
(11) 580. Brown enviromental changes :aa starv 2 h(1)
(12) 581. Brown enviromental changes :aa starv 4 h(1)
(13) 582. Brown enviromental changes :aa starv 6 h(1)
(14) 583. Brown enviromental changes :Nitrogen Depletion 30 min.(1)
(15) 584. Brown enviromental changes :Nitrogen Depletion 1 h(1)
(16) 585. Brown enviromental changes :Nitrogen Depletion 2 h(1)
(17) 586. Brown enviromental changes :Nitrogen Depletion 4 h(1)
(18) 587. Brown enviromental changes :Nitrogen Depletion 8 h(1)
(19) 588. Brown enviromental changes :Nitrogen Depletion 12 h(1)
(20) 589. Brown enviromental changes :Nitrogen Depletion 1 d(1)
(21) 590. Brown enviromental changes :Nitrogen Depletion 2 d(1)
(22) 591. Brown enviromental changes :Nitrogen Depletion 3 d(1)
(23) 592. Brown enviromental changes :Nitrogen Depletion 5 d(1)
(24) 100 microM BCS 60 min
(25) 100 microM CuSO4 30 min
(26) MAC1-up (B)
(27) MAC1-up (C)
(28) wt+gal

DAL82 --> DAL7 exp. conditions:28

(1) 49. Expression in response to 50 nM alpha-factor: 0,15,30,45,60,90,120 min(5)
(2) 428. Expression in strain PM38 (wild type) in response to 30 min 50 nM treatment with rapamycin in YPD(1)
(3) 429. Expression in strain YHE711 (wild type) in response to 30 min 50 nM treatment with rapamycin in YPD(1)
(4) 439. Expression in strain Jk9-3da (wild type) in response to 30 min 50 nM treatment with rapamycin in YPD(1)
(5) 442. Expression in strain PM38 (wild type) in response to 30 min 50 nM treatment with rapamycin in YPD(1)
(6) 479. Expression in diploid cells in response to rapamycin (100nM) for: 15min,30min,90min,120min(2)
(7) 531. Brown enviromental changes :constant 0.32 mM H2O2 (120 min) redo(1)
(8) 569. Brown enviromental changes :1M sorbitol - 60 min(1)
(9) 578. Brown enviromental changes :aa starv 0.5 h(1)
(10) 579. Brown enviromental changes :aa starv 1 h(1)
(11) 580. Brown enviromental changes :aa starv 2 h(1)
(12) 581. Brown enviromental changes :aa starv 4 h(1)
(13) 582. Brown enviromental changes :aa starv 6 h(1)
(14) 583. Brown enviromental changes :Nitrogen Depletion 30 min.(1)
(15) 584. Brown enviromental changes :Nitrogen Depletion 1 h(1)
(16) 585. Brown enviromental changes :Nitrogen Depletion 2 h(1)
(17) 586. Brown enviromental changes :Nitrogen Depletion 4 h(1)
(18) 587. Brown enviromental changes :Nitrogen Depletion 8 h(1)
(19) 588. Brown enviromental changes :Nitrogen Depletion 12 h(1)
(20) 589. Brown enviromental changes :Nitrogen Depletion 1 d(1)
(21) 590. Brown enviromental changes :Nitrogen Depletion 2 d(1)
(22) 591. Brown enviromental changes :Nitrogen Depletion 3 d(1)
(23) 592. Brown enviromental changes :Nitrogen Depletion 5 d(1)
(24) 100 microM BCS 60 min
(25) 100 microM CuSO4 30 min
(26) MAC1-up (B)
(27) MAC1-up (C)
(28) wt+gal

DAL82 -*-> DCG1 exp. conditions:28

(1) 49. Expression in response to 50 nM alpha-factor: 0,15,30,45,60,90,120 min(5)
(2) 428. Expression in strain PM38 (wild type) in response to 30 min 50 nM treatment with rapamycin in YPD(1)
(3) 429. Expression in strain YHE711 (wild type) in response to 30 min 50 nM treatment with rapamycin in YPD(1)
(4) 439. Expression in strain Jk9-3da (wild type) in response to 30 min 50 nM treatment with rapamycin in YPD(1)
(5) 442. Expression in strain PM38 (wild type) in response to 30 min 50 nM treatment with rapamycin in YPD(1)
(6) 479. Expression in diploid cells in response to rapamycin (100nM) for: 15min,30min,90min,120min(2)
(7) 531. Brown enviromental changes :constant 0.32 mM H2O2 (120 min) redo(1)
(8) 569. Brown enviromental changes :1M sorbitol - 60 min(1)
(9) 578. Brown enviromental changes :aa starv 0.5 h(1)
(10) 579. Brown enviromental changes :aa starv 1 h(1)
(11) 580. Brown enviromental changes :aa starv 2 h(1)
(12) 581. Brown enviromental changes :aa starv 4 h(1)
(13) 582. Brown enviromental changes :aa starv 6 h(1)
(14) 583. Brown enviromental changes :Nitrogen Depletion 30 min.(1)
(15) 584. Brown enviromental changes :Nitrogen Depletion 1 h(1)
(16) 585. Brown enviromental changes :Nitrogen Depletion 2 h(1)
(17) 586. Brown enviromental changes :Nitrogen Depletion 4 h(1)
(18) 587. Brown enviromental changes :Nitrogen Depletion 8 h(1)
(19) 588. Brown enviromental changes :Nitrogen Depletion 12 h(1)
(20) 589. Brown enviromental changes :Nitrogen Depletion 1 d(1)
(21) 590. Brown enviromental changes :Nitrogen Depletion 2 d(1)
(22) 591. Brown enviromental changes :Nitrogen Depletion 3 d(1)
(23) 592. Brown enviromental changes :Nitrogen Depletion 5 d(1)
(24) 100 microM BCS 60 min
(25) 100 microM CuSO4 30 min
(26) MAC1-up (B)
(27) MAC1-up (C)
(28) wt+gal

GAT1 -*-> DAL2 exp. conditions:27

(1) 428. Expression in strain PM38 (wild type) in response to 30 min 50 nM treatment with rapamycin in YPD(1)
(2) 429. Expression in strain YHE711 (wild type) in response to 30 min 50 nM treatment with rapamycin in YPD(1)
(3) 439. Expression in strain Jk9-3da (wild type) in response to 30 min 50 nM treatment with rapamycin in YPD(1)
(4) 442. Expression in strain PM38 (wild type) in response to 30 min 50 nM treatment with rapamycin in YPD(1)
(5) 477. Expression in response to trichostatin A (TSA): 15min,30min,60min,120min(1)
(6) 477. Expression in response to trichostatin A (TSA): 15min,30min,60min,120min(2)
(7) 477. Expression in response to trichostatin A (TSA): 15min,30min,60min,120min(3)
(8) 477. Expression in response to trichostatin A (TSA): 15min,30min,60min,120min(4)
(9) 479. Expression in diploid cells in response to rapamycin (100nM) for: 15min,30min,90min,120min(1)
(10) 479. Expression in diploid cells in response to rapamycin (100nM) for: 15min,30min,90min,120min(2)
(11) 479. Expression in diploid cells in response to rapamycin (100nM) for: 15min,30min,90min,120min(4)
(12) 578. Brown enviromental changes :aa starv 0.5 h(1)
(13) 579. Brown enviromental changes :aa starv 1 h(1)
(14) 580. Brown enviromental changes :aa starv 2 h(1)
(15) 581. Brown enviromental changes :aa starv 4 h(1)

(16) 582. Brown enviromental changes :aa starv 6 h(1)
(17) 583. Brown enviromental changes :Nitrogen Depletion 30 min.(1)
(18) 584. Brown enviromental changes :Nitrogen Depletion 1 h(1)
(19) 585. Brown enviromental changes :Nitrogen Depletion 2 h(1)
(20) 586. Brown enviromental changes :Nitrogen Depletion 4 h(1)
(21) 587. Brown enviromental changes :Nitrogen Depletion 8 h(1)
(22) 588. Brown enviromental changes :Nitrogen Depletion 12 h(1)
(23) 589. Brown enviromental changes :Nitrogen Depletion 1 d(1)
(24) 590. Brown enviromental changes :Nitrogen Depletion 2 d(1)
(25) 591. Brown enviromental changes :Nitrogen Depletion 3 d(1)
(26) 592. Brown enviromental changes :Nitrogen Depletion 5 d(1)
(27) 617. Brown enviromental changes :YPD stationary phase 3 d ypd-1(1)

GCN4 --> ARG1 exp. conditions:26

(1) 89. Expression in response to 3-aminotriazole(1)
(2) 95. Expression in response to 50ug/mL FK506(1)
(3) 332. Rosetta 2000: Expression in cells with CMD1 under tet promoter(1)
(4) 387. Rosetta 2000: Expression in cells with ERG11 under tet promoter(1)
(5) 395. Rosetta 2000: Expression in response to 2-deoxy-D-glucose(1)
(6) 401. Rosetta 2000: Expression in response to HU(1)
(7) 402. Rosetta 2000: Expression in response to Itraconazole(1)
(8) 403. Rosetta 2000: Expression in response to Lovastatin(1)
(9) 406. Rosetta 2000: Expression in response to Terbinafine(1)
(10) 407. Rosetta 2000: Expression in response to Tunicamycin(1)
(11) 445. Expression in response to 0.1% MMS for 60 min (average of 3 experiments)(1)
(12) 446. Expression in response to 0.1% MMS for 10 min(1)
(13) 447. Expression in response to 0.1% MMS for 30 min(1)
(14) 448. Expression in response to 0.1% MMS for 60 min(1)
(15) 449. Expression in response to 0.1% MMS for 60 min(1)
(16) 462. Expression in response to 0.05% MMS for 60 min(1)
(17) 463. Expression in response to 0.1% MMS for 60 min(1)
(18) 479. Expression in diploid cells in response to rapamycin (100nM) for: 15min,30min,90min,120min(3)
(19) 533. Brown enviromental changes :1 mM Menadione (10 min)redo(1)
(20) 579. Brown enviromental changes :aa starv 1 h(1)
(21) 580. Brown enviromental changes :aa starv 2 h(1)
(22) 581. Brown enviromental changes :aa starv 4 h(1)
(23) 584. Brown enviromental changes :Nitrogen Depletion 1 h(1)
(24) 585. Brown enviromental changes :Nitrogen Depletion 2 h(1)
(25) 586. Brown enviromental changes :Nitrogen Depletion 4 h(1)
(26) DES460 + 0.02% MMS - 15 min

GCN4 --> ARG2 exp. conditions:24

(1) 5. Expression during the cell cycle (alpha factor arrest and release)(11)
(2) 6. Expression during the cell cycle (cdc15 arrest and release)(15)
(3) 54. Expression in response to overproduction of Ste5p(1)
(4) 89. Expression in response to 3-aminotriazole(1)
(5) 95. Expression in response to 50ug/mL FK506(1)
(6) 402. Rosetta 2000: Expression in response to Itraconazole(1)
(7) 403. Rosetta 2000: Expression in response to Lovastatin(1)
(8) 406. Rosetta 2000: Expression in response to Terbinafine(1)
(9) 407. Rosetta 2000: Expression in response to Tunicamycin(1)
(10) 429. Expression in strain YHE711 (wild type) in response to 30 min 50 nM treatment with rapamycin in YPD(1)
(11) 446. Expression in response to 0.1% MMS for 10 min(1)
(12) 447. Expression in response to 0.1% MMS for 30 min(1)
(13) 452. Expression in response to low 4NQO (2 microgram/ml) for 60 min(1)
(14) 479. Expression in diploid cells in response to rapamycin (100nM) for: 15min,30min,90min,120min(2)
(15) 481. Expression in response to heat shock: 15,30,45,60,120 min(2)
(16) 481. Expression in response to heat shock: 15,30,45,60,120 min(4)
(17) 537. Brown enviromental changes :1 mM Menadione (50 min)redo(1)
(18) 552. Brown enviromental changes :dtt 030 min dtt-2(1)
(19) 564. Brown enviromental changes :1.5 mM diamide (90 min)(1)
(20) 612. Brown enviromental changes :YPD stationary phase 4 h ypd-1(1)
(21) 684. Expression in response to 0.8M NaCl for 10 min in wild type(1)
(22) DES460 + 0.02% MMS - 5 min
(23) DES460 + 0.02% MMS - 15 min
(24) 100 microM BCS 30 min

GCN4 --> ARG3 exp. conditions:26

(1) 89. Expression in response to 3-aminotriazole(1)
(2) 95. Expression in response to 50ug/mL FK506(1)
(3) 332. Rosetta 2000: Expression in cells with CMD1 under tet promoter(1)
(4) 387. Rosetta 2000: Expression in cells with ERG11 under tet promoter(1)
(5) 395. Rosetta 2000: Expression in response to 2-deoxy-D-glucose(1)
(6) 401. Rosetta 2000: Expression in response to HU(1)
(7) 402. Rosetta 2000: Expression in response to Itraconazole(1)
(8) 403. Rosetta 2000: Expression in response to Lovastatin(1)
(9) 406. Rosetta 2000: Expression in response to Terbinafine(1)
(10) 407. Rosetta 2000: Expression in response to Tunicamycin(1)
(11) 445. Expression in response to 0.1% MMS for 60 min (average of 3 experiments)(1)
(12) 446. Expression in response to 0.1% MMS for 10 min(1)
(13) 447. Expression in response to 0.1% MMS for 30 min(1)
(14) 448. Expression in response to 0.1% MMS for 60 min(1)
(15) 449. Expression in response to 0.1% MMS for 60 min(1)
(16) 462. Expression in response to 0.05% MMS for 60 min(1)
(17) 463. Expression in response to 0.1% MMS for 60 min(1)
(18) 479. Expression in diploid cells in response to rapamycin (100nM) for: 15min,30min,90min,120min(3)
(19) 533. Brown enviromental changes :1 mM Menadione (10 min)redo(1)
(20) 579. Brown enviromental changes :aa starv 1 h(1)
(21) 580. Brown enviromental changes :aa starv 2 h(1)
(22) 581. Brown enviromental changes :aa starv 4 h(1)
(23) 584. Brown enviromental changes :Nitrogen Depletion 1 h(1)
(24) 585. Brown enviromental changes :Nitrogen Depletion 2 h(1)
(25) 586. Brown enviromental changes :Nitrogen Depletion 4 h(1)
(26) DES460 + 0.02% MMS - 15 min

GCN4 --> ARG4 exp. conditions:26

(1) 387. Rosetta 2000: Expression in cells with ERG11 under tet promoter(1)
(2) 392. Rosetta 2000: Expression in cells with PMA1 under tet promoter(1)
(3) 395. Rosetta 2000: Expression in response to 2-deoxy-D-glucose(1)
(4) 402. Rosetta 2000: Expression in response to Itraconazole(1)

(5) 406. Rosetta 2000: Expression in response to Terbinafine(1)
(6) 407. Rosetta 2000: Expression in response to Tunicamycin(1)
(7) 445. Expression in response to 0.1% MMS for 60 min (average of 3 experiments)(1)
(8) 446. Expression in response to 0.1% MMS for 10 min(1)
(9) 447. Expression in response to 0.1% MMS for 30 min(1)
(10) 448. Expression in response to 0.1% MMS for 60 min(1)
(11) 449. Expression in response to 0.1% MMS for 60 min(1)
(12) 450. Expression in response to low MNNG (8 microgram/ml) for 60 min(1)
(13) 462. Expression in response to 0.05% MMS for 60 min(1)
(14) 504. Brown enviromental changes :37C to 25C shock - 30 min(1)
(15) 523. Brown enviromental changes :constant 0.32 mM H2O2 (10 min) redo(1)
(16) 533. Brown enviromental changes :1 mM Menadione (10 min)redo(1)
(17) 534. Brown enviromental changes :1 mM Menadione (20 min) redo(1)
(18) 535. Brown enviromental changes :1 mM Menadione (30 min) redo(1)
(19) 537. Brown enviromental changes :1 mM Menadione (50 min)redo(1)
(20) 538. Brown enviromental changes :1 mM Menadione (80 min) redo(1)
(21) 581. Brown enviromental changes :aa starv 4 h(1)
(22) 582. Brown enviromental changes :aa starv 6 h(1)
(23) 585. Brown enviromental changes :Nitrogen Depletion 2 h(1)
(24) 612. Brown enviromental changes :YPD stationary phase 4 h ypd-1(1)
(25) DES460 + 0.02% MMS - 5 min
(26) DES460 + 0.02% MMS - 15 min

GCN4 --> ARG8 exp. conditions:26

(1) 89. Expression in response to 3-aminotriazole(1)
(2) 95. Expression in response to 50ug/mL FK506(1)
(3) 332. Rosetta 2000: Expression in cells with CMD1 under tet promoter(1)
(4) 387. Rosetta 2000: Expression in cells with ERG11 under tet promoter(1)
(5) 395. Rosetta 2000: Expression in response to 2-deoxy-D-glucose(1)
(6) 401. Rosetta 2000: Expression in response to HU(1)
(7) 402. Rosetta 2000: Expression in response to Itraconazole(1)
(8) 403. Rosetta 2000: Expression in response to Lovastatin(1)
(9) 406. Rosetta 2000: Expression in response to Terbinafine(1)
(10) 407. Rosetta 2000: Expression in response to Tunicamycin(1)
(11) 445. Expression in response to 0.1% MMS for 60 min (average of 3 experiments)(1)
(12) 446. Expression in response to 0.1% MMS for 10 min(1)
(13) 447. Expression in response to 0.1% MMS for 30 min(1)
(14) 448. Expression in response to 0.1% MMS for 60 min(1)
(15) 449. Expression in response to 0.1% MMS for 60 min(1)
(16) 462. Expression in response to 0.05% MMS for 60 min(1)
(17) 463. Expression in response to 0.1% MMS for 60 min(1)
(18) 479. Expression in diploid cells in response to rapamycin (100nM) for: 15min,30min,90min,120min(3)
(19) 533. Brown enviromental changes :1 mM Menadione (10 min)redo(1)
(20) 579. Brown enviromental changes :aa starv 1 h(1)
(21) 580. Brown enviromental changes :aa starv 2 h(1)
(22) 581. Brown enviromental changes :aa starv 4 h(1)
(23) 584. Brown enviromental changes :Nitrogen Depletion 1 h(1)
(24) 585. Brown enviromental changes :Nitrogen Depletion 2 h(1)
(25) 586. Brown enviromental changes :Nitrogen Depletion 4 h(1)
(26) DES460 + 0.02% MMS - 15 min

GCN4 --> ARO3 exp. conditions:39

(1) 5. Expression during the cell cycle (alpha factor arrest and release)(11)
(2) 7. Expression during the cell Cycle (cdc28)(10)
(3) 89. Expression in response to 3-aminotriazole(1)
(4) 95. Expression in response to 50ug/mL FK506(1)
(5) 332. Rosetta 2000: Expression in cells with CMD1 under tet promoter(1)
(6) 387. Rosetta 2000: Expression in cells with ERG11 under tet promoter(1)
(7) 395. Rosetta 2000: Expression in response to 2-deoxy-D-glucose(1)
(8) 401. Rosetta 2000: Expression in response to HU(1)
(9) 402. Rosetta 2000: Expression in response to Itraconazole(1)
(10) 403. Rosetta 2000: Expression in response to Lovastatin(1)
(11) 406. Rosetta 2000: Expression in response to Terbinafine(1)
(12) 407. Rosetta 2000: Expression in response to Tunicamycin(1)
(13) 445. Expression in response to 0.1% MMS for 60 min (average of 3 experiments)(1)
(14) 446. Expression in response to 0.1% MMS for 10 min(1)
(15) 447. Expression in response to 0.1% MMS for 30 min(1)
(16) 448. Expression in response to 0.1% MMS for 60 min(1)
(17) 449. Expression in response to 0.1% MMS for 60 min(1)
(18) 462. Expression in response to 0.05% MMS for 60 min(1)
(19) 463. Expression in response to 0.1% MMS for 60 min(1)
(20) 479. Expression in diploid cells in response to rapamycin (100nM) for: 15min,30min,90min,120min(2)
(21) 479. Expression in diploid cells in response to rapamycin (100nM) for: 15min,30min,90min,120min(3)
(22) 504. Brown enviromental changes :37C to 25C shock - 30 min(1)
(23) 506. Brown enviromental changes :37C to 25C shock - 60 min(1)
(24) 523. Brown enviromental changes :constant 0.32 mM H2O2 (10 min) redo(1)
(25) 533. Brown enviromental changes :1 mM Menadione (10 min)redo(1)
(26) 552. Brown enviromental changes :dtt 030 min dtt-2(1)
(27) 579. Brown enviromental changes :aa starv 1 h(1)
(28) 580. Brown enviromental changes :aa starv 2 h(1)
(29) 581. Brown enviromental changes :aa starv 4 h(1)
(30) 584. Brown enviromental changes :Nitrogen Depletion 1 h(1)
(31) 585. Brown enviromental changes :Nitrogen Depletion 2 h(1)
(32) 586. Brown enviromental changes :Nitrogen Depletion 4 h(1)
(33) 595. Brown enviromental changes :diauxic shift timecourse(1)
(34) 611. Brown enviromental changes :YPD stationary phase 2 h ypd-1(1)
(35) 612. Brown enviromental changes :YPD stationary phase 4 h ypd-1(1)
(36) 684. Expression in response to 0.8M NaCl for 10 min in wild type(1)
(37) DES460 + 0.02% MMS - 5 min
(38) DES460 + 0.02% MMS - 15 min
(39) 100 microM BCS 30 min

GCN4 --> ARO4 exp. conditions:39

(1) 5. Expression during the cell cycle (alpha factor arrest and release)(11)
(2) 7. Expression during the cell Cycle (cdc28)(10)
(3) 89. Expression in response to 3-aminotriazole(1)
(4) 95. Expression in response to 50ug/mL FK506(1)
(5) 332. Rosetta 2000: Expression in cells with CMD1 under tet promoter(1)
(6) 387. Rosetta 2000: Expression in cells with ERG11 under tet promoter(1)
(7) 395. Rosetta 2000: Expression in response to 2-deoxy-D-glucose(1)
(8) 401. Rosetta 2000: Expression in response to HU(1)
(9) 402. Rosetta 2000: Expression in response to Itraconazole(1)
(10) 403. Rosetta 2000: Expression in response to Lovastatin(1)

(11) 406. Rosetta 2000: Expression in response to Terbinafine(1)
(12) 407. Rosetta 2000: Expression in response to Tunicamycin(1)
(13) 445. Expression in response to 0.1% MMS for 60 min (average of 3 experiments)(1)
(14) 446. Expression in response to 0.1% MMS for 10 min(1)
(15) 447. Expression in response to 0.1% MMS for 30 min(1)
(16) 448. Expression in response to 0.1% MMS for 60 min(1)
(17) 449. Expression in response to 0.1% MMS for 60 min(1)
(18) 462. Expression in response to 0.05% MMS for 60 min(1)
(19) 463. Expression in response to 0.1% MMS for 60 min(1)
(20) 479. Expression in diploid cells in response to rapamycin (100nM) for: 15min,30min,90min,120min(2)
(21) 479. Expression in diploid cells in response to rapamycin (100nM) for: 15min,30min,90min,120min(3)
(22) 504. Brown enviromental changes :37C to 25C shock - 30 min(1)
(23) 506. Brown enviromental changes :37C to 25C shock - 60 min(1)
(24) 523. Brown enviromental changes :constant 0.32 mM H2O2 (10 min) redo(1)
(25) 533. Brown enviromental changes :1 mM Menadione (10 min)redo(1)
(26) 552. Brown enviromental changes :dtt 030 min dtt-2(1)
(27) 579. Brown enviromental changes :aa starv 1 h(1)
(28) 580. Brown enviromental changes :aa starv 2 h(1)
(29) 581. Brown enviromental changes :aa starv 4 h(1)
(30) 584. Brown enviromental changes :Nitrogen Depletion 1 h(1)
(31) 585. Brown enviromental changes :Nitrogen Depletion 2 h(1)
(32) 586. Brown enviromental changes :Nitrogen Depletion 4 h(1)
(33) 595. Brown enviromental changes :diauxic shift timecourse(1)
(34) 611. Brown enviromental changes :YPD stationary phase 2 h ypd-1(1)
(35) 612. Brown enviromental changes :YPD stationary phase 4 h ypd-1(1)
(36) 684. Expression in response to 0.8M NaCl for 10 min in wild type(1)
(37) DES460 + 0.02% MMS - 5 min
(38) DES460 + 0.02% MMS - 15 min
(39) 100 microM BCS 30 min

GCN4 --> ASN1 exp. conditions:45

(1) 6. Expression during the cell cycle (cdc15 arrest and release)(15)
(2) 89. Expression in response to 3-aminotriazole(1)
(3) 95. Expression in response to 50ug/mL FK506(1)
(4) 332. Rosetta 2000: Expression in cells with CMD1 under tet promoter(1)
(5) 387. Rosetta 2000: Expression in cells with ERG11 under tet promoter(1)
(6) 395. Rosetta 2000: Expression in response to 2-deoxy-D-glucose(1)
(7) 401. Rosetta 2000: Expression in response to HU(1)
(8) 402. Rosetta 2000: Expression in response to Itraconazole(1)
(9) 403. Rosetta 2000: Expression in response to Lovastatin(1)
(10) 406. Rosetta 2000: Expression in response to Terbinafine(1)
(11) 407. Rosetta 2000: Expression in response to Tunicamycin(1)
(12) 445. Expression in response to 0.1% MMS for 60 min (average of 3 experiments)(1)
(13) 446. Expression in response to 0.1% MMS for 10 min(1)
(14) 447. Expression in response to 0.1% MMS for 30 min(1)
(15) 448. Expression in response to 0.1% MMS for 60 min(1)
(16) 449. Expression in response to 0.1% MMS for 60 min(1)
(17) 462. Expression in response to 0.05% MMS for 60 min(1)
(18) 463. Expression in response to 0.1% MMS for 60 min(1)
(19) 479. Expression in diploid cells in response to rapamycin (100nM) for: 15min,30min,90min,120min(2)
(20) 479. Expression in diploid cells in response to rapamycin (100nM) for: 15min,30min,90min,120min(3)
(21) 503. Brown enviromental changes :37C to 25C shock - 15 min(1)
(22) 507. Brown enviromental changes :37C to 25C shock - 90 min(1)
(23) 533. Brown enviromental changes :1 mM Menadione (10 min)redo(1)
(24) 537. Brown enviromental changes :1 mM Menadione (50 min)redo(1)
(25) 538. Brown enviromental changes :1 mM Menadione (80 min) redo(1)
(26) 540. Brown enviromental changes :1 mM Menadione (120 min)redo(1)
(27) 550. Brown enviromental changes :dtt 000 min dtt-2(1)
(28) 551. Brown enviromental changes :dtt 015 min dtt-2(1)
(29) 552. Brown enviromental changes :dtt 030 min dtt-2(1)
(30) 571. Brown enviromental changes :1M sorbitol - 120 min(1)
(31) 579. Brown enviromental changes :aa starv 1 h(1)
(32) 580. Brown enviromental changes :aa starv 2 h(1)
(33) 581. Brown enviromental changes :aa starv 4 h(1)
(34) 584. Brown enviromental changes :Nitrogen Depletion 1 h(1)
(35) 585. Brown enviromental changes :Nitrogen Depletion 2 h(1)
(36) 586. Brown enviromental changes :Nitrogen Depletion 4 h(1)
(37) 602. Brown enviromental changes :YPD 4 h ypd-2(1)
(38) 612. Brown enviromental changes :YPD stationary phase 4 h ypd-1(1)
(39) 613. Brown enviromental changes :YPD stationary phase 8 h ypd-1(1)
(40) 684. Expression in response to 0.8M NaCl for 10 min in wild type(1)
(41) DES460 + 0.02% MMS - 5 min
(42) DES460 + 0.02% MMS - 15 min
(43) wt_plus_gamma_120_min
(44) DES460 (wt) - mock irradiation - 30 min
(45) 100 microM BCS 30 min

GCN4 -*-> ATR1 exp. conditions:39

(1) 5. Expression during the cell cycle (alpha factor arrest and release)(11)
(2) 7. Expression during the cell Cycle (cdc28)(10)
(3) 89. Expression in response to 3-aminotriazole(1)
(4) 95. Expression in response to 50ug/mL FK506(1)
(5) 332. Rosetta 2000: Expression in cells with CMD1 under tet promoter(1)
(6) 387. Rosetta 2000: Expression in cells with ERG11 under tet promoter(1)
(7) 395. Rosetta 2000: Expression in response to 2-deoxy-D-glucose(1)
(8) 401. Rosetta 2000: Expression in response to HU(1)
(9) 402. Rosetta 2000: Expression in response to Itraconazole(1)
(10) 403. Rosetta 2000: Expression in response to Lovastatin(1)
(11) 406. Rosetta 2000: Expression in response to Terbinafine(1)
(12) 407. Rosetta 2000: Expression in response to Tunicamycin(1)
(13) 445. Expression in response to 0.1% MMS for 60 min (average of 3 experiments)(1)
(14) 446. Expression in response to 0.1% MMS for 10 min(1)
(15) 447. Expression in response to 0.1% MMS for 30 min(1)
(16) 448. Expression in response to 0.1% MMS for 60 min(1)
(17) 449. Expression in response to 0.1% MMS for 60 min(1)
(18) 462. Expression in response to 0.05% MMS for 60 min(1)
(19) 463. Expression in response to 0.1% MMS for 60 min(1)
(20) 479. Expression in diploid cells in response to rapamycin (100nM) for: 15min,30min,90min,120min(2)
(21) 479. Expression in diploid cells in response to rapamycin (100nM) for: 15min,30min,90min,120min(3)
(22) 504. Brown enviromental changes :37C to 25C shock - 30 min(1)
(23) 506. Brown enviromental changes :37C to 25C shock - 60 min(1)
(24) 523. Brown enviromental changes :constant 0.32 mM H2O2 (10 min) redo(1)
(25) 533. Brown enviromental changes :1 mM Menadione (10 min)redo(1)
(26) 552. Brown enviromental changes :dtt 030 min dtt-2(1)
(27) 579. Brown enviromental changes :aa starv 1 h(1)
(28) 580. Brown enviromental changes :aa starv 2 h(1)

(29) 581. Brown enviromental changes :aa starv 4 h(1)
(30) 584. Brown enviromental changes :Nitrogen Depletion 1 h(1)
(31) 585. Brown enviromental changes :Nitrogen Depletion 2 h(1)
(32) 586. Brown enviromental changes :Nitrogen Depletion 4 h(1)
(33) 595. Brown enviromental changes :diauxic shift timecourse(1)
(34) 611. Brown enviromental changes :YPD stationary phase 2 h ypd-1(1)
(35) 612. Brown enviromental changes :YPD stationary phase 4 h ypd-1(1)
(36) 684. Expression in response to 0.8M NaCl for 10 min in wild type(1)
(37) DES460 + 0.02% MMS - 5 min
(38) DES460 + 0.02% MMS - 15 min
(39) 100 microM BCS 30 min

GCN4 --> CPA2 exp. conditions:37

(1) 89. Expression in response to 3-aminotriazole(1)
(2) 95. Expression in response to 50ug/mL FK506(1)
(3) 332. Rosetta 2000: Expression in cells with CMD1 under tet promoter(1)
(4) 387. Rosetta 2000: Expression in cells with ERG11 under tet promoter(1)
(5) 392. Rosetta 2000: Expression in cells with PMA1 under tet promoter(1)
(6) 395. Rosetta 2000: Expression in response to 2-deoxy-D-glucose(1)
(7) 401. Rosetta 2000: Expression in response to HU(1)
(8) 402. Rosetta 2000: Expression in response to Itraconazole(1)
(9) 403. Rosetta 2000: Expression in response to Lovastatin(1)
(10) 406. Rosetta 2000: Expression in response to Terbinafine(1)
(11) 407. Rosetta 2000: Expression in response to Tunicamycin(1)
(12) 445. Expression in response to 0.1% MMS for 60 min (average of 3 experiments)(1)
(13) 446. Expression in response to 0.1% MMS for 10 min(1)
(14) 447. Expression in response to 0.1% MMS for 30 min(1)
(15) 448. Expression in response to 0.1% MMS for 60 min(1)
(16) 449. Expression in response to 0.1% MMS for 60 min(1)
(17) 452. Expression in response to low 4NQO (2 microgram/ml) for 60 min(1)
(18) 462. Expression in response to 0.05% MMS for 60 min(1)
(19) 463. Expression in response to 0.1% MMS for 60 min(1)
(20) 479. Expression in diploid cells in response to rapamycin (100nM) for: 15min,30min,90min,120min(3)
(21) 481. Expression in response to heat shock: 15,30,45,60,120 min(4)
(22) 523. Brown enviromental changes :constant 0.32 mM H2O2 (10 min) redo(1)
(23) 533. Brown enviromental changes :1 mM Menadione (10 min)redo(1)
(24) 546. Brown enviromental changes :2.5mM DTT 060 min dtt-1(1)
(25) 572. Brown enviromental changes :Hypo-osmotic shock - 5 min(1)
(26) 579. Brown enviromental changes :aa starv 1 h(1)
(27) 580. Brown enviromental changes :aa starv 2 h(1)
(28) 581. Brown enviromental changes :aa starv 4 h(1)
(29) 582. Brown enviromental changes :aa starv 6 h(1)
(30) 583. Brown enviromental changes :Nitrogen Depletion 30 min.(1)
(31) 584. Brown enviromental changes :Nitrogen Depletion 1 h(1)
(32) 585. Brown enviromental changes :Nitrogen Depletion 2 h(1)
(33) 586. Brown enviromental changes :Nitrogen Depletion 4 h(1)
(34) 591. Brown enviromental changes :Nitrogen Depletion 3 d(1)
(35) 602. Brown enviromental changes :YPD 4 h ypd-2(1)
(36) DES460 + 0.02% MMS - 15 min
(37) wt_plus_gamma_60_min

GCN4 -*-> FOL2 exp. conditions:39

(1) 5. Expression during the cell cycle (alpha factor arrest and release)(11)
(2) 7. Expression during the cell Cycle (cdc28)(10)
(3) 89. Expression in response to 3-aminotriazole(1)
(4) 95. Expression in response to 50ug/mL FK506(1)
(5) 332. Rosetta 2000: Expression in cells with CMD1 under tet promoter(1)
(6) 387. Rosetta 2000: Expression in cells with ERG11 under tet promoter(1)
(7) 395. Rosetta 2000: Expression in response to 2-deoxy-D-glucose(1)
(8) 401. Rosetta 2000: Expression in response to HU(1)
(9) 402. Rosetta 2000: Expression in response to Itraconazole(1)
(10) 403. Rosetta 2000: Expression in response to Lovastatin(1)
(11) 406. Rosetta 2000: Expression in response to Terbinafine(1)
(12) 407. Rosetta 2000: Expression in response to Tunicamycin(1)
(13) 445. Expression in response to 0.1% MMS for 60 min (average of 3 experiments)(1)
(14) 446. Expression in response to 0.1% MMS for 10 min(1)
(15) 447. Expression in response to 0.1% MMS for 30 min(1)
(16) 448. Expression in response to 0.1% MMS for 60 min(1)
(17) 449. Expression in response to 0.1% MMS for 60 min(1)
(18) 462. Expression in response to 0.05% MMS for 60 min(1)
(19) 463. Expression in response to 0.1% MMS for 60 min(1)
(20) 479. Expression in diploid cells in response to rapamycin (100nM) for: 15min,30min,90min,120min(2)
(21) 479. Expression in diploid cells in response to rapamycin (100nM) for: 15min,30min,90min,120min(3)
(22) 504. Brown enviromental changes :37C to 25C shock - 30 min(1)
(23) 506. Brown enviromental changes :37C to 25C shock - 60 min(1)
(24) 523. Brown enviromental changes :constant 0.32 mM H2O2 (10 min) redo(1)
(25) 533. Brown enviromental changes :1 mM Menadione (10 min)redo(1)
(26) 552. Brown enviromental changes :dtt 030 min dtt-2(1)
(27) 579. Brown enviromental changes :aa starv 1 h(1)
(28) 580. Brown enviromental changes :aa starv 2 h(1)
(29) 581. Brown enviromental changes :aa starv 4 h(1)
(30) 584. Brown enviromental changes :Nitrogen Depletion 1 h(1)
(31) 585. Brown enviromental changes :Nitrogen Depletion 2 h(1)
(32) 586. Brown enviromental changes :Nitrogen Depletion 4 h(1)
(33) 595. Brown enviromental changes :diauxic shift timecourse(1)
(34) 611. Brown enviromental changes :YPD stationary phase 2 h ypd-1(1)
(35) 612. Brown enviromental changes :YPD stationary phase 4 h ypd-1(1)
(36) 684. Expression in response to 0.8M NaCl for 10 min in wild type(1)
(37) DES460 + 0.02% MMS - 5 min
(38) DES460 + 0.02% MMS - 15 min
(39) 100 microM BCS 30 min

GCN4 --> GLT1 exp. conditions:26

(1) 5. Expression during the cell cycle (alpha factor arrest and release)(16)
(2) 5. Expression during the cell cycle (alpha factor arrest and release)(18)
(3) 6. Expression during the cell cycle (cdc15 arrest and release)(7)
(4) 7. Expression during the cell Cycle (cdc28)(10)
(5) 11. Expression during diauxic shift: 9h,11h,13h,15h,17h,19h,21h(2)
(6) 395. Rosetta 2000: Expression in response to 2-deoxy-D-glucose(1)
(7) 402. Rosetta 2000: Expression in response to Itraconazole(1)
(8) 403. Rosetta 2000: Expression in response to Lovastatin(1)
(9) 406. Rosetta 2000: Expression in response to Terbinafine(1)
(10) 407. Rosetta 2000: Expression in response to Tunicamycin(1)

```
    (11) 497. Brown enviromental changes :Heat Shock 000 minutes  hs-2(1)
    (12) 505. Brown enviromental changes :37C to 25C shock - 45 min(1)
    (13) 507. Brown enviromental changes :37C to 25C shock - 90 min(1)
    (14) 550. Brown enviromental changes :dtt 000 min  dtt-2(1)
    (15) 551. Brown enviromental changes :dtt 015 min dtt-2(1)
    (16) 580. Brown enviromental changes :aa starv 2 h(1)
    (17) 581. Brown enviromental changes :aa starv 4 h(1)
    (18) 586. Brown enviromental changes :Nitrogen Depletion 4 h(1)
    (19) 594. Brown enviromental changes :diauxic shift timecourse(1)
    (20) 602. Brown enviromental changes :YPD 4 h ypd-2(1)
    (21) 612. Brown enviromental changes :YPD stationary phase 4 h ypd-1(1)
    (22) 613. Brown enviromental changes :YPD stationary phase 8 h ypd-1(1)
    (23) 681. Expression in response to 0.4M NaCl for 10 min in wild type(1)
    (24) 684. Expression in response to 0.8M NaCl for 10 min in wild type(1)
    (25) DES460 + 0.02% MMS - 5 min
    (26) DES460 (wt) - mock irradiation - 30 min


GCN4 --> HIS4  exp. conditions:45


    (1) 6. Expression during the cell cycle (cdc15 arrest and release)(15)
    (2) 89. Expression in response to 3-aminotriazole(1)
    (3) 95. Expression in response to 50ug/mL FK506(1)
    (4) 332. Rosetta 2000: Expression in cells with CMD1 under tet promoter(1)
    (5) 387. Rosetta 2000: Expression in cells with ERG11 under tet promoter(1)
    (6) 395. Rosetta 2000: Expression in response to 2-deoxy-D-glucose(1)
    (7) 401. Rosetta 2000: Expression in response to HU(1)
    (8) 402. Rosetta 2000: Expression in response to Itraconazole(1)
    (9) 403. Rosetta 2000: Expression in response to Lovastatin(1)
    (10) 406. Rosetta 2000: Expression in response to Terbinafine(1)
    (11) 407. Rosetta 2000: Expression in response to Tunicamycin(1)
    (12) 445. Expression in response to 0.1% MMS for 60 min (average of 3 experiments)(1)
    (13) 446. Expression in response to 0.1% MMS for 10 min(1)
    (14) 447. Expression in response to 0.1% MMS for 30 min(1)
    (15) 448. Expression in response to 0.1% MMS for 60 min(1)
    (16) 449. Expression in response to 0.1% MMS for 60 min(1)
    (17) 462. Expression in response to 0.05% MMS for 60 min(1)
    (18) 463. Expression in response to 0.1% MMS for 60 min(1)
    (19) 479. Expression in diploid cells in response to rapamycin (100nM) for: 15min,30min,90min,120min(2)
    (20) 479. Expression in diploid cells in response to rapamycin (100nM) for: 15min,30min,90min,120min(3)
    (21) 503. Brown enviromental changes :37C to 25C shock - 15 min(1)
    (22) 507. Brown enviromental changes :37C to 25C shock - 90 min(1)
    (23) 533. Brown enviromental changes :1 mM Menadione (10 min)redo(1)
    (24) 537. Brown enviromental changes :1 mM Menadione (50 min)redo(1)
    (25) 538. Brown enviromental changes :1 mM Menadione (80 min) redo(1)
    (26) 540. Brown enviromental changes :1 mM Menadione (120 min)redo(1)
    (27) 550. Brown enviromental changes :dtt 000 min  dtt-2(1)
    (28) 551. Brown enviromental changes :dtt 015 min dtt-2(1)
    (29) 552. Brown enviromental changes :dtt 030 min  dtt-2(1)
    (30) 571. Brown enviromental changes :1M sorbitol - 120 min(1)
    (31) 579. Brown enviromental changes :aa starv 1 h(1)
    (32) 580. Brown enviromental changes :aa starv 2 h(1)
    (33) 581. Brown enviromental changes :aa starv 4 h(1)
    (34) 584. Brown enviromental changes :Nitrogen Depletion 1 h(1)
    (35) 585. Brown enviromental changes :Nitrogen Depletion 2 h(1)
    (36) 586. Brown enviromental changes :Nitrogen Depletion 4 h(1)
    (37) 602. Brown enviromental changes :YPD 4 h ypd-2(1)
    (38) 612. Brown enviromental changes :YPD stationary phase 4 h ypd-1(1)
    (39) 613. Brown enviromental changes :YPD stationary phase 8 h ypd-1(1)
    (40) 684. Expression in response to 0.8M NaCl for 10 min in wild type(1)
    (41) DES460 + 0.02% MMS - 5 min
    (42) DES460 + 0.02% MMS - 15 min
    (43) wt_plus_gamma_120_min
    (44) DES460 (wt) - mock irradiation - 30 min
    (45) 100 microM BCS 30 min


GCN4 --> HIS5  exp. conditions:37


    (1) 89. Expression in response to 3-aminotriazole(1)
    (2) 95. Expression in response to 50ug/mL FK506(1)
    (3) 332. Rosetta 2000: Expression in cells with CMD1 under tet promoter(1)
    (4) 387. Rosetta 2000: Expression in cells with ERG11 under tet promoter(1)
    (5) 392. Rosetta 2000: Expression in cells with PMA1 under tet promoter(1)
    (6) 395. Rosetta 2000: Expression in response to 2-deoxy-D-glucose(1)
    (7) 401. Rosetta 2000: Expression in response to HU(1)
    (8) 402. Rosetta 2000: Expression in response to Itraconazole(1)
    (9) 403. Rosetta 2000: Expression in response to Lovastatin(1)
    (10) 406. Rosetta 2000: Expression in response to Terbinafine(1)
    (11) 407. Rosetta 2000: Expression in response to Tunicamycin(1)
    (12) 445. Expression in response to 0.1% MMS for 60 min (average of 3 experiments)(1)
    (13) 446. Expression in response to 0.1% MMS for 10 min(1)
    (14) 447. Expression in response to 0.1% MMS for 30 min(1)
    (15) 448. Expression in response to 0.1% MMS for 60 min(1)
    (16) 449. Expression in response to 0.1% MMS for 60 min(1)
    (17) 452. Expression in response to low 4NQO (2 microgram/ml) for 60 min(1)
    (18) 462. Expression in response to 0.05% MMS for 60 min(1)
    (19) 463. Expression in response to 0.1% MMS for 60 min(1)
    (20) 479. Expression in diploid cells in response to rapamycin (100nM) for: 15min,30min,90min,120min(3)
    (21) 481. Expression in response to heat shock: 15,30,45,60,120 min(4)
    (22) 523. Brown enviromental changes :constant 0.32 mM H2O2 (10 min) redo(1)
    (23) 533. Brown enviromental changes :1 mM Menadione (10 min)redo(1)
    (24) 546. Brown enviromental changes :2.5mM DTT 060 min dtt-1(1)
    (25) 572. Brown enviromental changes :Hypo-osmotic shock - 5 min(1)
    (26) 579. Brown enviromental changes :aa starv 1 h(1)
    (27) 580. Brown enviromental changes :aa starv 2 h(1)
    (28) 581. Brown enviromental changes :aa starv 4 h(1)
    (29) 582. Brown enviromental changes :aa starv 6 h(1)
    (30) 583. Brown enviromental changes :Nitrogen Depletion 30 min.(1)
    (31) 584. Brown enviromental changes :Nitrogen Depletion 1 h(1)
    (32) 585. Brown enviromental changes :Nitrogen Depletion 2 h(1)
    (33) 586. Brown enviromental changes :Nitrogen Depletion 4 h(1)
    (34) 591. Brown enviromental changes :Nitrogen Depletion 3 d(1)
    (35) 602. Brown enviromental changes :YPD 4 h ypd-2(1)
    (36) DES460 + 0.02% MMS - 15 min
    (37) wt_plus_gamma_60_min


GCN4 --> HIS7  exp. conditions:40
```

(1) 7. Expression during the cell Cycle (cdc28)(10)
(2) 11. Expression during diauxic shift: 9h,11h,13h,15h,17h,19h,21h(3)
(3) 89. Expression in response to 3-aminotriazole(1)
(4) 95. Expression in response to 50ug/mL FK506(1)
(5) 332. Rosetta 2000: Expression in cells with CMD1 under tet promoter(1)
(6) 387. Rosetta 2000: Expression in cells with ERG11 under tet promoter(1)
(7) 395. Rosetta 2000: Expression in response to 2-deoxy-D-glucose(1)
(8) 401. Rosetta 2000: Expression in response to HU(1)
(9) 402. Rosetta 2000: Expression in response to Itraconazole(1)
(10) 403. Rosetta 2000: Expression in response to Lovastatin(1)
(11) 406. Rosetta 2000: Expression in response to Terbinafine(1)
(12) 407. Rosetta 2000: Expression in response to Tunicamycin(1)
(13) 445. Expression in response to 0.1% MMS for 60 min (average of 3 experiments)(1)
(14) 446. Expression in response to 0.1% MMS for 10 min(1)
(15) 447. Expression in response to 0.1% MMS for 30 min(1)
(16) 448. Expression in response to 0.1% MMS for 60 min(1)
(17) 449. Expression in response to 0.1% MMS for 60 min(1)
(18) 462. Expression in response to 0.05% MMS for 60 min(1)
(19) 463. Expression in response to 0.1% MMS for 60 min(1)
(20) 479. Expression in diploid cells in response to rapamycin (100nM) for: 15min,30min,90min,120min(3)
(21) 533. Brown enviromental changes :1 mM Menadione (10 min)redo(1)
(22) 551. Brown enviromental changes :dtt 015 min dtt-2(1)
(23) 552. Brown enviromental changes :dtt 030 min dtt-2(1)
(24) 572. Brown enviromental changes :Hypo-osmotic shock - 5 min(1)
(25) 573. Brown enviromental changes :Hypo-osmotic shock - 15 min(1)
(26) 575. Brown enviromental changes :Hypo-osmotic shock - 45 min(1)
(27) 579. Brown enviromental changes :aa starv 1 h(1)
(28) 580. Brown enviromental changes :aa starv 2 h(1)
(29) 581. Brown enviromental changes :aa starv 4 h(1)
(30) 582. Brown enviromental changes :aa starv 6 h(1)
(31) 584. Brown enviromental changes :Nitrogen Depletion 1 h(1)
(32) 585. Brown enviromental changes :Nitrogen Depletion 2 h(1)
(33) 586. Brown enviromental changes :Nitrogen Depletion 4 h(1)
(34) 595. Brown enviromental changes :diauxic shift timecourse(1)
(35) 602. Brown enviromental changes :YPD 4 h ypd-2(1)
(36) 611. Brown enviromental changes :YPD stationary phase 2 h ypd-1(1)
(37) 612. Brown enviromental changes :YPD stationary phase 4 h ypd-1(1)
(38) DES460 + 0.02% MMS - 5 min
(39) DES460 + 0.02% MMS - 15 min
(40) 100 microM BCS 30 min

GCN4 --> HOM3 exp. conditions:34

(1) 8. Expression during the cell cycle (cell size selection and release)(3)
(2) 89. Expression in response to 3-aminotriazole(1)
(3) 95. Expression in response to 50ug/mL FK506(1)
(4) 332. Rosetta 2000: Expression in cells with CMD1 under tet promoter(1)
(5) 387. Rosetta 2000: Expression in cells with ERG11 under tet promoter(1)
(6) 395. Rosetta 2000: Expression in response to 2-deoxy-D-glucose(1)
(7) 401. Rosetta 2000: Expression in response to HU(1)
(8) 402. Rosetta 2000: Expression in response to Itraconazole(1)
(9) 403. Rosetta 2000: Expression in response to Lovastatin(1)
(10) 406. Rosetta 2000: Expression in response to Terbinafine(1)
(11) 407. Rosetta 2000: Expression in response to Tunicamycin(1)
(12) PHO81c vs WT exp2(1)
(13) 429. Expression in strain YHE711 (wild type) in response to 30 min 50 nM treatment with rapamycin in YPD(1)
(14) 445. Expression in response to 0.1% MMS for 60 min (average of 3 experiments)(1)
(15) 446. Expression in response to 0.1% MMS for 10 min(1)
(16) 447. Expression in response to 0.1% MMS for 30 min(1)
(17) 448. Expression in response to 0.1% MMS for 60 min(1)
(18) 449. Expression in response to 0.1% MMS for 60 min(1)
(19) 462. Expression in response to 0.05% MMS for 60 min(1)
(20) 463. Expression in response to 0.1% MMS for 60 min(1)
(21) 479. Expression in diploid cells in response to rapamycin (100nM) for: 15min,30min,90min,120min(2)
(22) 479. Expression in diploid cells in response to rapamycin (100nM) for: 15min,30min,90min,120min(3)
(23) 533. Brown enviromental changes :1 mM Menadione (10 min)redo(1)
(24) 579. Brown enviromental changes :aa starv 1 h(1)
(25) 580. Brown enviromental changes :aa starv 2 h(1)
(26) 581. Brown enviromental changes :aa starv 4 h(1)
(27) 582. Brown enviromental changes :aa starv 6 h(1)
(28) 584. Brown enviromental changes :Nitrogen Depletion 1 h(1)
(29) 585. Brown enviromental changes :Nitrogen Depletion 2 h(1)
(30) 586. Brown enviromental changes :Nitrogen Depletion 4 h(1)
(31) 612. Brown enviromental changes :YPD stationary phase 4 h ypd-1(1)
(32) 684. Expression in response to 0.8M NaCl for 10 min in wild type(1)
(33) DES460 + 0.02% MMS - 5 min
(34) DES460 + 0.02% MMS - 15 min

GCN4 -*-> IDP1 exp. conditions:34

(1) 8. Expression during the cell cycle (cell size selection and release)(3)
(2) 89. Expression in response to 3-aminotriazole(1)
(3) 95. Expression in response to 50ug/mL FK506(1)
(4) 332. Rosetta 2000: Expression in cells with CMD1 under tet promoter(1)
(5) 387. Rosetta 2000: Expression in cells with ERG11 under tet promoter(1)
(6) 395. Rosetta 2000: Expression in response to 2-deoxy-D-glucose(1)
(7) 401. Rosetta 2000: Expression in response to HU(1)
(8) 402. Rosetta 2000: Expression in response to Itraconazole(1)
(9) 403. Rosetta 2000: Expression in response to Lovastatin(1)
(10) 406. Rosetta 2000: Expression in response to Terbinafine(1)
(11) 407. Rosetta 2000: Expression in response to Tunicamycin(1)
(12) PHO81c vs WT exp2(1)
(13) 429. Expression in strain YHE711 (wild type) in response to 30 min 50 nM treatment with rapamycin in YPD(1)
(14) 445. Expression in response to 0.1% MMS for 60 min (average of 3 experiments)(1)
(15) 446. Expression in response to 0.1% MMS for 10 min(1)
(16) 447. Expression in response to 0.1% MMS for 30 min(1)
(17) 448. Expression in response to 0.1% MMS for 60 min(1)
(18) 449. Expression in response to 0.1% MMS for 60 min(1)
(19) 462. Expression in response to 0.05% MMS for 60 min(1)
(20) 463. Expression in response to 0.1% MMS for 60 min(1)
(21) 479. Expression in diploid cells in response to rapamycin (100nM) for: 15min,30min,90min,120min(2)
(22) 479. Expression in diploid cells in response to rapamycin (100nM) for: 15min,30min,90min,120min(3)
(23) 533. Brown enviromental changes :1 mM Menadione (10 min)redo(1)
(24) 579. Brown enviromental changes :aa starv 1 h(1)
(25) 580. Brown enviromental changes :aa starv 2 h(1)
(26) 581. Brown enviromental changes :aa starv 4 h(1)
(27) 582. Brown enviromental changes :aa starv 6 h(1)

(28) 584. Brown enviromental changes :Nitrogen Depletion 1 h(1)
(29) 585. Brown enviromental changes :Nitrogen Depletion 2 h(1)
(30) 586. Brown enviromental changes :Nitrogen Depletion 4 h(1)
(31) 612. Brown enviromental changes :YPD stationary phase 4 h ypd-1(1)
(32) 684. Expression in response to 0.8M NaCl for 10 min in wild type(1)
(33) DES460 + 0.02% MMS - 5 min
(34) DES460 + 0.02% MMS - 15 min

GCN4 --> ILV1 exp. conditions:48

(1) 6. Expression during the cell cycle (cdc15 arrest and release)(15)
(2) 8. Expression during the cell cycle (cell size selection and release)(3)
(3) 11. Expression during diauxic shift: 9h,11h,13h,15h,17h,19h,21h(3)
(4) 89. Expression in response to 3-aminotriazole(1)
(5) 95. Expression in response to 50ug/mL FK506(1)
(6) 332. Rosetta 2000: Expression in cells with CMD1 under tet promoter(1)
(7) 387. Rosetta 2000: Expression in cells with ERG11 under tet promoter(1)
(8) 389. Rosetta 2000: Expression in cells with HMG2 under tet promoter(1)
(9) 395. Rosetta 2000: Expression in response to 2-deoxy-D-glucose(1)
(10) 400. Rosetta 2000: Expression in response to Glucosamine(1)
(11) 401. Rosetta 2000: Expression in response to HU(1)
(12) 402. Rosetta 2000: Expression in response to Itraconazole(1)
(13) 403. Rosetta 2000: Expression in response to Lovastatin(1)
(14) 406. Rosetta 2000: Expression in response to Terbinafine(1)
(15) 407. Rosetta 2000: Expression in response to Tunicamycin(1)
(16) 445. Expression in response to 0.1% MMS for 60 min (average of 3 experiments)(1)
(17) 446. Expression in response to 0.1% MMS for 10 min(1)
(18) 447. Expression in response to 0.1% MMS for 30 min(1)
(19) 448. Expression in response to 0.1% MMS for 60 min(1)
(20) 449. Expression in response to 0.1% MMS for 60 min(1)
(21) 462. Expression in response to 0.05% MMS for 60 min(1)
(22) 463. Expression in response to 0.1% MMS for 60 min(1)
(23) 479. Expression in diploid cells in response to rapamycin (100nM) for: 15min,30min,90min,120min(2)
(24) 479. Expression in diploid cells in response to rapamycin (100nM) for: 15min,30min,90min,120min(3)
(25) 483. Expression in response to alkali: 10,20,40,60,80,100 min(6)
(26) 516. Brown enviromental changes :33C vs. 30C - 90 minutes(1)
(27) 523. Brown enviromental changes :constant 0.32 mM H2O2 (10 min) redo(1)
(28) 533. Brown enviromental changes :1 mM Menadione (10 min)redo(1)
(29) 534. Brown enviromental changes :1 mM Menadione (20 min) redo(1)
(30) 536. Brown enviromental changes :1mM Menadione (40 min) redo(1)
(31) 537. Brown enviromental changes :1 mM Menadione (50 min)redo(1)
(32) 538. Brown enviromental changes :1 mM Menadione (80 min) redo(1)
(33) 551. Brown enviromental changes :dtt 015 min dtt-2(1)
(34) 570. Brown enviromental changes :1M sorbitol - 90 min(1)
(35) 579. Brown enviromental changes :aa starv 1 h(1)
(36) 580. Brown enviromental changes :aa starv 2 h(1)
(37) 581. Brown enviromental changes :aa starv 4 h(1)
(38) 584. Brown enviromental changes :Nitrogen Depletion 1 h(1)
(39) 585. Brown enviromental changes :Nitrogen Depletion 2 h(1)
(40) 586. Brown enviromental changes :Nitrogen Depletion 4 h(1)
(41) 595. Brown enviromental changes :diauxic shift timecourse(1)
(42) 611. Brown enviromental changes :YPD stationary phase 2 h ypd-1(1)
(43) 612. Brown enviromental changes :YPD stationary phase 4 h ypd-1(1)
(44) 613. Brown enviromental changes :YPD stationary phase 8 h ypd-1(1)
(45) 684. Expression in response to 0.8M NaCl for 10 min in wild type(1)
(46) DES460 + 0.02% MMS - 15 min
(47) DES460 (wt) - mock irradiation - 30 min
(48) 100 microM BCS 30 min

GCN4 -*-> ILV3 exp. conditions:26

(1) 89. Expression in response to 3-aminotriazole(1)
(2) 95. Expression in response to 50ug/mL FK506(1)
(3) 332. Rosetta 2000: Expression in cells with CMD1 under tet promoter(1)
(4) 387. Rosetta 2000: Expression in cells with ERG11 under tet promoter(1)
(5) 395. Rosetta 2000: Expression in response to 2-deoxy-D-glucose(1)
(6) 401. Rosetta 2000: Expression in response to HU(1)
(7) 402. Rosetta 2000: Expression in response to Itraconazole(1)
(8) 403. Rosetta 2000: Expression in response to Lovastatin(1)
(9) 406. Rosetta 2000: Expression in response to Terbinafine(1)
(10) 407. Rosetta 2000: Expression in response to Tunicamycin(1)
(11) 445. Expression in response to 0.1% MMS for 60 min (average of 3 experiments)(1)
(12) 446. Expression in response to 0.1% MMS for 10 min(1)
(13) 447. Expression in response to 0.1% MMS for 30 min(1)
(14) 448. Expression in response to 0.1% MMS for 60 min(1)
(15) 449. Expression in response to 0.1% MMS for 60 min(1)
(16) 462. Expression in response to 0.05% MMS for 60 min(1)
(17) 463. Expression in response to 0.1% MMS for 60 min(1)
(18) 479. Expression in diploid cells in response to rapamycin (100nM) for: 15min,30min,90min,120min(3)
(19) 533. Brown enviromental changes :1 mM Menadione (10 min)redo(1)
(20) 579. Brown enviromental changes :aa starv 1 h(1)
(21) 580. Brown enviromental changes :aa starv 2 h(1)
(22) 581. Brown enviromental changes :aa starv 4 h(1)
(23) 584. Brown enviromental changes :Nitrogen Depletion 1 h(1)
(24) 585. Brown enviromental changes :Nitrogen Depletion 2 h(1)
(25) 586. Brown enviromental changes :Nitrogen Depletion 4 h(1)
(26) DES460 + 0.02% MMS - 15 min

GCN4 --> ILV5 exp. conditions:23

(1) 7. Expression during the cell Cycle (cdc28)(10)
(2) 402. Rosetta 2000: Expression in response to Itraconazole(1)
(3) 403. Rosetta 2000: Expression in response to Lovastatin(1)
(4) 407. Rosetta 2000: Expression in response to Tunicamycin(1)
(5) 446. Expression in response to 0.1% MMS for 10 min(1)
(6) 497. Brown enviromental changes :Heat Shock 000 minutes hs-2(1)
(7) 498. Brown enviromental changes :Heat Shock 000 minutes hs-2(1)
(8) 504. Brown enviromental changes :37C to 25C shock - 30 min(1)
(9) 506. Brown enviromental changes :37C to 25C shock - 60 min(1)
(10) 507. Brown enviromental changes :37C to 25C shock - 90 min(1)
(11) 552. Brown enviromental changes :dtt 030 min dtt-2(1)
(12) 573. Brown enviromental changes :Hypo-osmotic shock - 15 min(1)
(13) 575. Brown enviromental changes :Hypo-osmotic shock - 45 min(1)
(14) 576. Brown enviromental changes :Hypo-osmotic shock - 60 min(1)
(15) 581. Brown enviromental changes :aa starv 4 h(1)
(16) 585. Brown enviromental changes :Nitrogen Depletion 2 h(1)

(17) 586. Brown enviromental changes :Nitrogen Depletion 4 h(1)
(18) 602. Brown enviromental changes :YPD 4 h ypd-2(1)
(19) 612. Brown enviromental changes :YPD stationary phase 4 h ypd-1(1)
(20) 681. Expression in response to 0.4M NaCl for 10 min in wild type(1)
(21) 684. Expression in response to 0.8M NaCl for 10 min in wild type(1)
(22) DES460 (wt) - mock irradiation - 30 min
(23) 100 microM BCS 30 min

GCN4 --> LEU3 exp. conditions:25

(1) 6. Expression during the cell cycle (cdc15 arrest and release)(15)
(2) 6. Expression during the cell cycle (cdc15 arrest and release)(20)
(3) 7. Expression during the cell Cycle (cdc28)(10)
(4) 8. Expression during the cell cycle (cell size selection and release)(4)
(5) 8. Expression during the cell cycle (cell size selection and release)(5)
(6) 89. Expression in response to 3-aminotriazole(1)
(7) 394. Rosetta 2000: Expression in cells with YEF3 under tet promoter(1)
(8) 402. Rosetta 2000: Expression in response to Itraconazole(1)
(9) 403. Rosetta 2000: Expression in response to Lovastatin(1)
(10) 406. Rosetta 2000: Expression in response to Terbinafine(1)
(11) 407. Rosetta 2000: Expression in response to Tunicamycin(1)
(12) 446. Expression in response to 0.1% MMS for 10 min(1)
(13) 481. Expression in response to heat shock: 15,30,45,60,120 min(2)
(14) 481. Expression in response to heat shock: 15,30,45,60,120 min(4)
(15) 497. Brown enviromental changes :Heat Shock 000 minutes hs-2(1)
(16) 504. Brown enviromental changes :37C to 25C shock - 30 min(1)
(17) 551. Brown enviromental changes :dtt 015 min dtt-2(1)
(18) 556. Brown enviromental changes :dtt 480 min dtt-2(1)
(19) 575. Brown enviromental changes :Hypo-osmotic shock - 45 min(1)
(20) 581. Brown enviromental changes :aa starv 4 h(1)
(21) 681. Expression in response to 0.4M NaCl for 10 min in wild type(1)
(22) 684. Expression in response to 0.8M NaCl for 10 min in wild type(1)
(23) DES460 (wt) - mock irradiation - 30 min
(24) MHY1 (crt1) vs CRY1 (wild type)
(25) 100 microM BCS 30 min

GCN4 --> LEU4 exp. conditions:28

(1) 5. Expression during the cell cycle (alpha factor arrest and release)(11)
(2) 7. Expression during the cell Cycle (cdc28)(10)
(3) 89. Expression in response to 3-aminotriazole(1)
(4) 95. Expression in response to 50ug/mL FK506(1)
(5) 387. Rosetta 2000: Expression in cells with ERG11 under tet promoter(1)
(6) 401. Rosetta 2000: Expression in response to HU(1)
(7) 402. Rosetta 2000: Expression in response to Itraconazole(1)
(8) 403. Rosetta 2000: Expression in response to Lovastatin(1)
(9) 406. Rosetta 2000: Expression in response to Terbinafine(1)
(10) 407. Rosetta 2000: Expression in response to Tunicamycin(1)
(11) 446. Expression in response to 0.1% MMS for 10 min(1)
(12) 447. Expression in response to 0.1% MMS for 30 min(1)
(13) 448. Expression in response to 0.1% MMS for 60 min(1)
(14) 449. Expression in response to 0.1% MMS for 60 min(1)
(15) 479. Expression in diploid cells in response to rapamycin (100nM) for: 15min,30min,90min,120min(2)
(16) 479. Expression in diploid cells in response to rapamycin (100nM) for: 15min,30min,90min,120min(3)
(17) 504. Brown enviromental changes :37C to 25C shock - 30 min(1)
(18) 506. Brown enviromental changes :37C to 25C shock - 60 min(1)
(19) 523. Brown enviromental changes :constant 0.32 mM H2O2 (10 min) redo(1)
(20) 552. Brown enviromental changes :dtt 030 min dtt-2(1)
(21) 581. Brown enviromental changes :aa starv 4 h(1)
(22) 595. Brown enviromental changes :diauxic shift timecourse(1)
(23) 611. Brown enviromental changes :YPD stationary phase 2 h ypd-1(1)
(24) 612. Brown enviromental changes :YPD stationary phase 4 h ypd-1(1)
(25) 684. Expression in response to 0.8M NaCl for 10 min in wild type(1)
(26) DES460 + 0.02% MMS - 5 min
(27) DES460 + 0.02% MMS - 15 min
(28) 100 microM BCS 30 min

GCN4 --> LYS2 exp. conditions:44

(1) 5. Expression during the cell cycle (alpha factor arrest and release)(17)
(2) 7. Expression during the cell Cycle (cdc28)(10)
(3) 8. Expression during the cell cycle (cell size selection and release)(3)
(4) 11. Expression during diauxic shift: 9h,11h,13h,15h,17h,19h,21h(2)
(5) 89. Expression in response to 3-aminotriazole(1)
(6) 95. Expression in response to 50ug/mL FK506(1)
(7) 332. Rosetta 2000: Expression in cells with CMD1 under tet promoter(1)
(8) 387. Rosetta 2000: Expression in cells with ERG11 under tet promoter(1)
(9) 395. Rosetta 2000: Expression in response to 2-deoxy-D-glucose(1)
(10) 401. Rosetta 2000: Expression in response to HU(1)
(11) 402. Rosetta 2000: Expression in response to Itraconazole(1)
(12) 403. Rosetta 2000: Expression in response to Lovastatin(1)
(13) 406. Rosetta 2000: Expression in response to Terbinafine(1)
(14) 407. Rosetta 2000: Expression in response to Tunicamycin(1)
(15) 445. Expression in response to 0.1% MMS for 60 min (average of 3 experiments)(1)
(16) 446. Expression in response to 0.1% MMS for 10 min(1)
(17) 447. Expression in response to 0.1% MMS for 30 min(1)
(18) 448. Expression in response to 0.1% MMS for 60 min(1)
(19) 449. Expression in response to 0.1% MMS for 60 min(1)
(20) 462. Expression in response to 0.05% MMS for 60 min(1)
(21) 463. Expression in response to 0.1% MMS for 60 min(1)
(22) 477. Expression in response to trichostatin A (TSA): 15min,30min,60min,120min(3)
(23) 479. Expression in diploid cells in response to rapamycin (100nM) for: 15min,30min,90min,120min(3)
(24) 530. Brown enviromental changes :constant 0.32 mM H2O2 (100 min) redo(1)
(25) 533. Brown enviromental changes :1 mM Menadione (10 min)redo(1)
(26) 551. Brown enviromental changes :dtt 015 min dtt-2(1)
(27) 570. Brown enviromental changes :1M sorbitol - 90 min(1)
(28) 575. Brown enviromental changes :Hypo-osmotic shock - 45 min(1)
(29) 576. Brown enviromental changes :Hypo-osmotic shock - 60 min(1)
(30) 579. Brown enviromental changes :aa starv 1 h(1)
(31) 580. Brown enviromental changes :aa starv 2 h(1)
(32) 581. Brown enviromental changes :aa starv 4 h(1)
(33) 584. Brown enviromental changes :Nitrogen Depletion 1 h(1)
(34) 585. Brown enviromental changes :Nitrogen Depletion 2 h(1)
(35) 586. Brown enviromental changes :Nitrogen Depletion 4 h(1)
(36) 594. Brown enviromental changes :diauxic shift timecourse(1)
(37) 684. Expression in response to 0.8M NaCl for 10 min in wild type(1)

(38) DES460 + 0.02% MMS - 5 min
(39) DES460 + 0.02% MMS - 15 min
(40) DES460 (wt) - mock irradiation - 30 min
(41) DES460 (wt) - mock irradiation - 60 min
(42) MHY1 (crt1) vs CRY1 (wild type)
(43) MHY1 (crt1) vs. CRY1 (wild type) - log phase
(44) 100 microM BCS 30 min

GCN4 --> MET16 exp. conditions:25

(1) 89. Expression in response to 3-aminotriazole(1)
(2) 95. Expression in response to 50ug/mL FK506(1)
(3) 387. Rosetta 2000: Expression in cells with ERG11 under tet promoter(1)
(4) 392. Rosetta 2000: Expression in cells with PMA1 under tet promoter(1)
(5) 395. Rosetta 2000: Expression in response to 2-deoxy-D-glucose(1)
(6) 402. Rosetta 2000: Expression in response to Itraconazole(1)
(7) 403. Rosetta 2000: Expression in response to Lovastatin(1)
(8) 407. Rosetta 2000: Expression in response to Tunicamycin(1)
(9) 446. Expression in response to 0.1% MMS for 10 min(1)
(10) 452. Expression in response to low 4NQO (2 microgram/ml) for 60 min(1)
(11) 481. Expression in response to heat shock: 15,30,45,60,120 min(4)
(12) 523. Brown enviromental changes :constant 0.32 mM H2O2 (10 min) redo(1)
(13) 546. Brown enviromental changes :2.5mM DTT 060 min dtt-1(1)
(14) 572. Brown enviromental changes :Hypo-osmotic shock - 5 min(1)
(15) 579. Brown enviromental changes :aa starv 1 h(1)
(16) 580. Brown enviromental changes :aa starv 2 h(1)
(17) 581. Brown enviromental changes :aa starv 4 h(1)
(18) 582. Brown enviromental changes :aa starv 6 h(1)
(19) 583. Brown enviromental changes :Nitrogen Depletion 30 min.(1)
(20) 584. Brown enviromental changes :Nitrogen Depletion 1 h(1)
(21) 585. Brown enviromental changes :Nitrogen Depletion 2 h(1)
(22) 586. Brown enviromental changes :Nitrogen Depletion 4 h(1)
(23) 591. Brown enviromental changes :Nitrogen Depletion 3 d(1)
(24) 602. Brown enviromental changes :YPD 4 h ypd-2(1)
(25) wt_plus_gamma_60_min

GCN4 --> MET4 exp. conditions:28

(1) 6. Expression during the cell cycle (cdc15 arrest and release)(15)
(2) 11. Expression during diauxic shift: 9h,11h,13h,15h,17h,19h,21h(3)
(3) 95. Expression in response to 50ug/mL FK506(1)
(4) 387. Rosetta 2000: Expression in cells with ERG11 under tet promoter(1)
(5) 402. Rosetta 2000: Expression in response to Itraconazole(1)
(6) 403. Rosetta 2000: Expression in response to Lovastatin(1)
(7) 406. Rosetta 2000: Expression in response to Terbinafine(1)
(8) 407. Rosetta 2000: Expression in response to Tunicamycin(1)
(9) 479. Expression in diploid cells in response to rapamycin (100nM) for: 15min,30min,90min,120min(2)
(10) 479. Expression in diploid cells in response to rapamycin (100nM) for: 15min,30min,90min,120min(3)
(11) 479. Expression in diploid cells in response to rapamycin (100nM) for: 15min,30min,90min,120min(4)
(12) 481. Expression in response to heat shock: 15,30,45,60,120 min(2)
(13) 481. Expression in response to heat shock: 15,30,45,60,120 min(4)
(14) 575. Brown enviromental changes :Hypo-osmotic shock - 45 min(1)
(15) 581. Brown enviromental changes :aa starv 4 h(1)
(16) 582. Brown enviromental changes :aa starv 6 h(1)
(17) 586. Brown enviromental changes :Nitrogen Depletion 4 h(1)
(18) 588. Brown enviromental changes :Nitrogen Depletion 12 h(1)
(19) 589. Brown enviromental changes :Nitrogen Depletion 1 d(1)
(20) 590. Brown enviromental changes :Nitrogen Depletion 2 d(1)
(21) 595. Brown enviromental changes :diauxic shift timecourse(1)
(22) 601. Brown enviromental changes :YPD 2 h ypd-2(1)
(23) 602. Brown enviromental changes :YPD 4 h ypd-2(1)
(24) 611. Brown enviromental changes :YPD stationary phase 2 h ypd-1(1)
(25) 612. Brown enviromental changes :YPD stationary phase 4 h ypd-1(1)
(26) 681. Expression in response to 0.4M NaCl for 10 min in wild type(1)
(27) DES460 (wt) - mock irradiation - 30 min
(28) 100 microM BCS 30 min

GCN4 -*-> SFT2 exp. conditions:25

(1) 89. Expression in response to 3-aminotriazole(1)
(2) 95. Expression in response to 50ug/mL FK506(1)
(3) 387. Rosetta 2000: Expression in cells with ERG11 under tet promoter(1)
(4) 401. Rosetta 2000: Expression in response to HU(1)
(5) 402. Rosetta 2000: Expression in response to Itraconazole(1)
(6) 403. Rosetta 2000: Expression in response to Lovastatin(1)
(7) 406. Rosetta 2000: Expression in response to Terbinafine(1)
(8) 407. Rosetta 2000: Expression in response to Tunicamycin(1)
(9) 446. Expression in response to 0.1% MMS for 10 min(1)
(10) 450. Expression in response to low MNNG (8 microgram/ml) for 60 min(1)
(11) 479. Expression in diploid cells in response to rapamycin (100nM) for: 15min,30min,90min,120min(2)
(12) 479. Expression in diploid cells in response to rapamycin (100nM) for: 15min,30min,90min,120min(3)
(13) 523. Brown enviromental changes :constant 0.32 mM H2O2 (10 min) redo(1)
(14) 533. Brown enviromental changes :1 mM Menadione (10 min)redo(1)
(15) 537. Brown enviromental changes :1 mM Menadione (50 min)redo(1)
(16) 538. Brown enviromental changes :1 mM Menadione (80 min) redo(1)
(17) 572. Brown enviromental changes :Hypo-osmotic shock - 5 min(1)
(18) 581. Brown enviromental changes :aa starv 4 h(1)
(19) 582. Brown enviromental changes :aa starv 6 h(1)
(20) 612. Brown enviromental changes :YPD stationary phase 4 h ypd-1(1)
(21) 613. Brown enviromental changes :YPD stationary phase 8 h ypd-1(1)
(22) 684. Expression in response to 0.8M NaCl for 10 min in wild type(1)
(23) DES460 + 0.02% MMS - 5 min
(24) DES460 + 0.02% MMS - 15 min
(25) 100 microM BCS 30 min

GCN4 --> TRP2 exp. conditions:43

(1) 1. Cell cycle: Expression in response to Cln3p (set 1)(1)
(2) 8. Expression during the cell cycle (cell size selection and release)(3)
(3) 11. Expression during diauxic shift: 9h,11h,13h,15h,17h,19h,21h(3)
(4) 89. Expression in response to 3-aminotriazole(1)
(5) 95. Expression in response to 50ug/mL FK506(1)
(6) 332. Rosetta 2000: Expression in cells with CMD1 under tet promoter(1)
(7) 387. Rosetta 2000: Expression in cells with ERG11 under tet promoter(1)

(8) 395. Rosetta 2000: Expression in response to 2-deoxy-D-glucose(1)
(9) 401. Rosetta 2000: Expression in response to HU(1)
(10) 402. Rosetta 2000: Expression in response to Itraconazole(1)
(11) 403. Rosetta 2000: Expression in response to Lovastatin(1)
(12) 406. Rosetta 2000: Expression in response to Terbinafine(1)
(13) 407. Rosetta 2000: Expression in response to Tunicamycin(1)
(14) 445. Expression in response to 0.1% MMS for 60 min (average of 3 experiments)(1)
(15) 446. Expression in response to 0.1% MMS for 10 min(1)
(16) 447. Expression in response to 0.1% MMS for 30 min(1)
(17) 448. Expression in response to 0.1% MMS for 60 min(1)
(18) 449. Expression in response to 0.1% MMS for 60 min(1)
(19) 462. Expression in response to 0.05% MMS for 60 min(1)
(20) 463. Expression in response to 0.1% MMS for 60 min(1)
(21) 479. Expression in diploid cells in response to rapamycin (100nM) for: 15min,30min,90min,120min(2)
(22) 479. Expression in diploid cells in response to rapamycin (100nM) for: 15min,30min,90min,120min(3)
(23) 504. Brown enviromental changes :37C to 25C shock - 30 min(1)
(24) 523. Brown enviromental changes :constant 0.32 mM H2O2 (10 min) redo(1)
(25) 533. Brown enviromental changes :1 mM Menadione (10 min)redo(1)
(26) 537. Brown enviromental changes :1 mM Menadione (50 min)redo(1)
(27) 538. Brown enviromental changes :1 mM Menadione (80 min) redo(1)
(28) 550. Brown enviromental changes :dtt 000 min dtt-2(1)
(29) 551. Brown enviromental changes :dtt 015 min dtt-2(1)
(30) 552. Brown enviromental changes :dtt 030 min dtt-2(1)
(31) 579. Brown enviromental changes :aa starv 1 h(1)
(32) 580. Brown enviromental changes :aa starv 2 h(1)
(33) 581. Brown enviromental changes :aa starv 4 h(1)
(34) 584. Brown enviromental changes :Nitrogen Depletion 1 h(1)
(35) 585. Brown enviromental changes :Nitrogen Depletion 2 h(1)
(36) 586. Brown enviromental changes :Nitrogen Depletion 4 h(1)
(37) 595. Brown enviromental changes :diauxic shift timecourse(1)
(38) 611. Brown enviromental changes :YPD stationary phase 2 h ypd-1(1)
(39) 612. Brown enviromental changes :YPD stationary phase 4 h ypd-1(1)
(40) 684. Expression in response to 0.8M NaCl for 10 min in wild type(1)
(41) DES460 + 0.02% MMS - 15 min
(42) MHY1 (crt1) vs CRY1 (wild type)
(43) 100 microM BCS 30 min

GCN4 --> TRP3 exp. conditions:46

(1) 7. Expression during the cell Cycle (cdc28)(10)
(2) 7. Expression during the cell Cycle (cdc28)(17)
(3) 89. Expression in response to 3-aminotriazole(1)
(4) 95. Expression in response to 50ug/mL FK506(1)
(5) 332. Rosetta 2000: Expression in cells with CMD1 under tet promoter(1)
(6) 387. Rosetta 2000: Expression in cells with ERG11 under tet promoter(1)
(7) 395. Rosetta 2000: Expression in response to 2-deoxy-D-glucose(1)
(8) 401. Rosetta 2000: Expression in response to HU(1)
(9) 402. Rosetta 2000: Expression in response to Itraconazole(1)
(10) 403. Rosetta 2000: Expression in response to Lovastatin(1)
(11) 406. Rosetta 2000: Expression in response to Terbinafine(1)
(12) 407. Rosetta 2000: Expression in response to Tunicamycin(1)
(13) 429. Expression in strain YHE711 (wild type) in response to 30 min 50 nM treatment with rapamycin in YPD(1)
(14) 445. Expression in response to 0.1% MMS for 60 min (average of 3 experiments)(1)
(15) 446. Expression in response to 0.1% MMS for 10 min(1)
(16) 447. Expression in response to 0.1% MMS for 30 min(1)
(17) 448. Expression in response to 0.1% MMS for 60 min(1)
(18) 449. Expression in response to 0.1% MMS for 60 min(1)
(19) 450. Expression in response to low MNNG (8 microgram/ml) for 60 min(1)
(20) 462. Expression in response to 0.05% MMS for 60 min(1)
(21) 463. Expression in response to 0.1% MMS for 60 min(1)
(22) 479. Expression in diploid cells in response to rapamycin (100nM) for: 15min,30min,90min,120min(2)
(23) 479. Expression in diploid cells in response to rapamycin (100nM) for: 15min,30min,90min,120min(3)
(24) 503. Brown enviromental changes :37C to 25C shock - 15 min(1)
(25) 504. Brown enviromental changes :37C to 25C shock - 30 min(1)
(26) 505. Brown enviromental changes :37C to 25C shock - 45 min(1)
(27) 506. Brown enviromental changes :37C to 25C shock - 60 min(1)
(28) 507. Brown enviromental changes :37C to 25C shock - 90 min(1)
(29) 533. Brown enviromental changes :1 mM Menadione (10 min)redo(1)
(30) 537. Brown enviromental changes :1 mM Menadione (50 min)redo(1)
(31) 550. Brown enviromental changes :dtt 000 min dtt-2(1)
(32) 552. Brown enviromental changes :dtt 030 min dtt-2(1)
(33) 579. Brown enviromental changes :aa starv 1 h(1)
(34) 580. Brown enviromental changes :aa starv 2 h(1)
(35) 581. Brown enviromental changes :aa starv 4 h(1)
(36) 584. Brown enviromental changes :Nitrogen Depletion 1 h(1)
(37) 585. Brown enviromental changes :Nitrogen Depletion 2 h(1)
(38) 586. Brown enviromental changes :Nitrogen Depletion 4 h(1)
(39) 611. Brown enviromental changes :YPD stationary phase 2 h ypd-1(1)
(40) 612. Brown enviromental changes :YPD stationary phase 4 h ypd-1(1)
(41) 613. Brown enviromental changes :YPD stationary phase 8 h ypd-1(1)
(42) 681. Expression in response to 0.4M NaCl for 10 min in wild type(1)
(43) 684. Expression in response to 0.8M NaCl for 10 min in wild type(1)
(44) DES460 + 0.02% MMS - 5 min
(45) DES460 + 0.02% MMS - 15 min
(46) MHY1 (crt1) vs CRY1 (wild type)

GCN4 --> TRP4 exp. conditions:48

(1) 6. Expression during the cell cycle (cdc15 arrest and release)(15)
(2) 8. Expression during the cell cycle (cell size selection and release)(3)
(3) 11. Expression during diauxic shift: 9h,11h,13h,15h,17h,19h,21h(3)
(4) 89. Expression in response to 3-aminotriazole(1)
(5) 95. Expression in response to 50ug/mL FK506(1)
(6) 332. Rosetta 2000: Expression in cells with CMD1 under tet promoter(1)
(7) 387. Rosetta 2000: Expression in cells with ERG11 under tet promoter(1)
(8) 389. Rosetta 2000: Expression in cells with HMG2 under tet promoter(1)
(9) 395. Rosetta 2000: Expression in response to 2-deoxy-D-glucose(1)
(10) 400. Rosetta 2000: Expression in response to Glucosamine(1)
(11) 401. Rosetta 2000: Expression in response to HU(1)
(12) 402. Rosetta 2000: Expression in response to Itraconazole(1)
(13) 403. Rosetta 2000: Expression in response to Lovastatin(1)
(14) 406. Rosetta 2000: Expression in response to Terbinafine(1)
(15) 407. Rosetta 2000: Expression in response to Tunicamycin(1)
(16) 445. Expression in response to 0.1% MMS for 60 min (average of 3 experiments)(1)
(17) 446. Expression in response to 0.1% MMS for 10 min(1)
(18) 447. Expression in response to 0.1% MMS for 30 min(1)
(19) 448. Expression in response to 0.1% MMS for 60 min(1)
(20) 449. Expression in response to 0.1% MMS for 60 min(1)

(21) 462. Expression in response to 0.05% MMS for 60 min(1)
(22) 463. Expression in response to 0.1% MMS for 60 min(1)
(23) 479. Expression in diploid cells in response to rapamycin (100nM) for: 15min,30min,90min,120min(2)
(24) 479. Expression in diploid cells in response to rapamycin (100nM) for: 15min,30min,90min,120min(3)
(25) 483. Expression in response to alkali: 10,20,40,60,80,100 min(6)
(26) 516. Brown enviromental changes :33C vs. 30C - 90 minutes(1)
(27) 523. Brown enviromental changes :constant 0.32 mM H2O2 (10 min) redo(1)
(28) 533. Brown enviromental changes :1 mM Menadione (10 min)redo(1)
(29) 534. Brown enviromental changes :1 mM Menadione (20 min) redo(1)
(30) 536. Brown enviromental changes :1mM Menadione (40 min) redo(1)
(31) 537. Brown enviromental changes :1 mM Menadione (50 min)redo(1)
(32) 538. Brown enviromental changes :1 mM Menadione (80 min) redo(1)
(33) 551. Brown enviromental changes :dtt 015 min dtt-2(1)
(34) 570. Brown enviromental changes :1M sorbitol - 90 min(1)
(35) 579. Brown enviromental changes :aa starv 1 h(1)
(36) 580. Brown enviromental changes :aa starv 2 h(1)
(37) 581. Brown enviromental changes :aa starv 4 h(1)
(38) 584. Brown enviromental changes :Nitrogen Depletion 1 h(1)
(39) 585. Brown enviromental changes :Nitrogen Depletion 2 h(1)
(40) 586. Brown enviromental changes :Nitrogen Depletion 4 h(1)
(41) 595. Brown enviromental changes :diauxic shift timecourse(1)
(42) 611. Brown enviromental changes :YPD stationary phase 2 h ypd-1(1)
(43) 612. Brown enviromental changes :YPD stationary phase 4 h ypd-1(1)
(44) 613. Brown enviromental changes :YPD stationary phase 8 h ypd-1(1)
(45) 684. Expression in response to 0.8M NaCl for 10 min in wild type(1)
(46) DES460 + 0.02% MMS - 15 min
(47) DES460 (wt) - mock irradiation - 30 min
(48) 100 microM BCS 30 min

GCN4 -*-> UGA3 exp. conditions:26

(1) 89. Expression in response to 3-aminotriazole(1)
(2) 95. Expression in response to 50ug/mL FK506(1)
(3) 332. Rosetta 2000: Expression in cells with CMD1 under tet promoter(1)
(4) 387. Rosetta 2000: Expression in cells with ERG11 under tet promoter(1)
(5) 395. Rosetta 2000: Expression in response to 2-deoxy-D-glucose(1)
(6) 401. Rosetta 2000: Expression in response to HU(1)
(7) 402. Rosetta 2000: Expression in response to Itraconazole(1)
(8) 403. Rosetta 2000: Expression in response to Lovastatin(1)
(9) 406. Rosetta 2000: Expression in response to Terbinafine(1)
(10) 407. Rosetta 2000: Expression in response to Tunicamycin(1)
(11) 445. Expression in response to 0.1% MMS for 60 min (average of 3 experiments)(1)
(12) 446. Expression in response to 0.1% MMS for 10 min(1)
(13) 447. Expression in response to 0.1% MMS for 30 min(1)
(14) 448. Expression in response to 0.1% MMS for 60 min(1)
(15) 449. Expression in response to 0.1% MMS for 60 min(1)
(16) 462. Expression in response to 0.05% MMS for 60 min(1)
(17) 463. Expression in response to 0.1% MMS for 60 min(1)
(18) 479. Expression in diploid cells in response to rapamycin (100nM) for: 15min,30min,90min,120min(3)
(19) 533. Brown enviromental changes :1 mM Menadione (10 min)redo(1)
(20) 579. Brown enviromental changes :aa starv 1 h(1)
(21) 580. Brown enviromental changes :aa starv 2 h(1)
(22) 581. Brown enviromental changes :aa starv 4 h(1)
(23) 584. Brown enviromental changes :Nitrogen Depletion 1 h(1)
(24) 585. Brown enviromental changes :Nitrogen Depletion 2 h(1)
(25) 586. Brown enviromental changes :Nitrogen Depletion 4 h(1)
(26) DES460 + 0.02% MMS - 15 min

GCN4 -*-> YHM1 exp. conditions:48

(1) 6. Expression during the cell cycle (cdc15 arrest and release)(15)
(2) 8. Expression during the cell cycle (cell size selection and release)(3)
(3) 11. Expression during diauxic shift: 9h,11h,13h,15h,17h,19h,21h(3)
(4) 89. Expression in response to 3-aminotriazole(1)
(5) 95. Expression in response to 50ug/mL FK506(1)
(6) 332. Rosetta 2000: Expression in cells with CMD1 under tet promoter(1)
(7) 387. Rosetta 2000: Expression in cells with ERG11 under tet promoter(1)
(8) 389. Rosetta 2000: Expression in cells with HMG2 under tet promoter(1)
(9) 395. Rosetta 2000: Expression in response to 2-deoxy-D-glucose(1)
(10) 400. Rosetta 2000: Expression in response to Glucosamine(1)
(11) 401. Rosetta 2000: Expression in response to HU(1)
(12) 402. Rosetta 2000: Expression in response to Itraconazole(1)
(13) 403. Rosetta 2000: Expression in response to Lovastatin(1)
(14) 406. Rosetta 2000: Expression in response to Terbinafine(1)
(15) 407. Rosetta 2000: Expression in response to Tunicamycin(1)
(16) 445. Expression in response to 0.1% MMS for 60 min (average of 3 experiments)(1)
(17) 446. Expression in response to 0.1% MMS for 10 min(1)
(18) 447. Expression in response to 0.1% MMS for 30 min(1)
(19) 448. Expression in response to 0.1% MMS for 60 min(1)
(20) 449. Expression in response to 0.1% MMS for 60 min(1)
(21) 462. Expression in response to 0.05% MMS for 60 min(1)
(22) 463. Expression in response to 0.1% MMS for 60 min(1)
(23) 479. Expression in diploid cells in response to rapamycin (100nM) for: 15min,30min,90min,120min(2)
(24) 479. Expression in diploid cells in response to rapamycin (100nM) for: 15min,30min,90min,120min(3)
(25) 483. Expression in response to alkali: 10,20,40,60,80,100 min(6)
(26) 516. Brown enviromental changes :33C vs. 30C - 90 minutes(1)
(27) 523. Brown enviromental changes :constant 0.32 mM H2O2 (10 min) redo(1)
(28) 533. Brown enviromental changes :1 mM Menadione (10 min)redo(1)
(29) 534. Brown enviromental changes :1 mM Menadione (20 min) redo(1)
(30) 536. Brown enviromental changes :1mM Menadione (40 min) redo(1)
(31) 537. Brown enviromental changes :1 mM Menadione (50 min)redo(1)
(32) 538. Brown enviromental changes :1 mM Menadione (80 min) redo(1)
(33) 551. Brown enviromental changes :dtt 015 min dtt-2(1)
(34) 570. Brown enviromental changes :1M sorbitol - 90 min(1)
(35) 579. Brown enviromental changes :aa starv 1 h(1)
(36) 580. Brown enviromental changes :aa starv 2 h(1)
(37) 581. Brown enviromental changes :aa starv 4 h(1)
(38) 584. Brown enviromental changes :Nitrogen Depletion 1 h(1)
(39) 585. Brown enviromental changes :Nitrogen Depletion 2 h(1)
(40) 586. Brown enviromental changes :Nitrogen Depletion 4 h(1)
(41) 595. Brown enviromental changes :diauxic shift timecourse(1)
(42) 611. Brown enviromental changes :YPD stationary phase 2 h ypd-1(1)
(43) 612. Brown enviromental changes :YPD stationary phase 4 h ypd-1(1)
(44) 613. Brown enviromental changes :YPD stationary phase 8 h ypd-1(1)
(45) 684. Expression in response to 0.8M NaCl for 10 min in wild type(1)
(46) DES460 + 0.02% MMS - 15 min
(47) DES460 (wt) - mock irradiation - 30 min
(48) 100 microM BCS 30 min

GCN4 -*-> YHR162W exp. conditions:26

(1) 89. Expression in response to 3-aminotriazole(1)
(2) 95. Expression in response to 50ug/mL FK506(1)
(3) 332. Rosetta 2000: Expression in cells with CMD1 under tet promoter(1)
(4) 387. Rosetta 2000: Expression in cells with ERG11 under tet promoter(1)
(5) 395. Rosetta 2000: Expression in response to 2-deoxy-D-glucose(1)
(6) 401. Rosetta 2000: Expression in response to HU(1)
(7) 402. Rosetta 2000: Expression in response to Itraconazole(1)
(8) 403. Rosetta 2000: Expression in response to Lovastatin(1)
(9) 406. Rosetta 2000: Expression in response to Terbinafine(1)
(10) 407. Rosetta 2000: Expression in response to Tunicamycin(1)
(11) 445. Expression in response to 0.1% MMS for 60 min (average of 3 experiments)(1)
(12) 446. Expression in response to 0.1% MMS for 10 min(1)
(13) 447. Expression in response to 0.1% MMS for 30 min(1)
(14) 448. Expression in response to 0.1% MMS for 60 min(1)
(15) 449. Expression in response to 0.1% MMS for 60 min(1)
(16) 462. Expression in response to 0.05% MMS for 60 min(1)
(17) 463. Expression in response to 0.1% MMS for 60 min(1)
(18) 479. Expression in diploid cells in response to rapamycin (100nM) for: 15min,30min,90min,120min(3)
(19) 533. Brown enviromental changes :1 mM Menadione (10 min)redo(1)
(20) 579. Brown enviromental changes :aa starv 1 h(1)
(21) 580. Brown enviromental changes :aa starv 2 h(1)
(22) 581. Brown enviromental changes :aa starv 4 h(1)
(23) 584. Brown enviromental changes :Nitrogen Depletion 1 h(1)
(24) 585. Brown enviromental changes :Nitrogen Depletion 2 h(1)
(25) 586. Brown enviromental changes :Nitrogen Depletion 4 h(1)
(26) DES460 + 0.02% MMS - 15 min

GLN3 --> DAL1 exp. conditions:31

(1) 7. Expression during the cell Cycle (cdc28)(6)
(2) 89. Expression in response to 3-aminotriazole(1)
(3) 95. Expression in response to 50ug/mL FK506(1)
(4) 387. Rosetta 2000: Expression in cells with ERG11 under tet promoter(1)
(5) 392. Rosetta 2000: Expression in cells with PMA1 under tet promoter(1)
(6) 401. Rosetta 2000: Expression in response to HU(1)
(7) 402. Rosetta 2000: Expression in response to Itraconazole(1)
(8) 428. Expression in strain PM38 (wild type) in response to 30 min 50 nM treatment with rapamycin in YPD(1)
(9) 429. Expression in strain YHE711 (wild type) in response to 30 min 50 nM treatment with rapamycin in YPD(1)
(10) 439. Expression in strain Jk9-3da (wild type) in response to 30 min 50 nM treatment with rapamycin in YPD(1)
(11) 442. Expression in strain PM38 (wild type) in response to 30 min 50 nM treatment with rapamycin in YPD(1)
(12) 479. Expression in diploid cells in response to rapamycin (100nM) for: 15min,30min,90min,120min(1)
(13) 479. Expression in diploid cells in response to rapamycin (100nM) for: 15min,30min,90min,120min(2)
(14) 495. Brown enviromental changes :Heat Shock 80 minutes hs-1(1)
(15) 570. Brown enviromental changes :1M sorbitol - 90 min(1)
(16) 578. Brown enviromental changes :aa starv 0.5 h(1)
(17) 579. Brown enviromental changes :aa starv 1 h(1)
(18) 580. Brown enviromental changes :aa starv 2 h(1)
(19) 581. Brown enviromental changes :aa starv 4 h(1)
(20) 582. Brown enviromental changes :aa starv 6 h(1)
(21) 583. Brown enviromental changes :Nitrogen Depletion 30 min.(1)
(22) 584. Brown enviromental changes :Nitrogen Depletion 1 h(1)
(23) 585. Brown enviromental changes :Nitrogen Depletion 2 h(1)
(24) 586. Brown enviromental changes :Nitrogen Depletion 4 h(1)
(25) 587. Brown enviromental changes :Nitrogen Depletion 8 h(1)
(26) 588. Brown enviromental changes :Nitrogen Depletion 12 h(1)
(27) 589. Brown enviromental changes :Nitrogen Depletion 1 d(1)
(28) 590. Brown enviromental changes :Nitrogen Depletion 2 d(1)
(29) 591. Brown enviromental changes :Nitrogen Depletion 3 d(1)
(30) 592. Brown enviromental changes :Nitrogen Depletion 5 d(1)
(31) 671. Expression in response to antimycin 120min(1)

GLN3 --> DAL2 exp. conditions:34

(1) 5. Expression during the cell cycle (alpha factor arrest and release)(11)
(2) 89. Expression in response to 3-aminotriazole(1)
(3) 95. Expression in response to 50ug/mL FK506(1)
(4) 387. Rosetta 2000: Expression in cells with ERG11 under tet promoter(1)
(5) 402. Rosetta 2000: Expression in response to Itraconazole(1)
(6) 406. Rosetta 2000: Expression in response to Terbinafine(1)
(7) 407. Rosetta 2000: Expression in response to Tunicamycin(1)
(8) 428. Expression in strain PM38 (wild type) in response to 30 min 50 nM treatment with rapamycin in YPD(1)
(9) 429. Expression in strain YHE711 (wild type) in response to 30 min 50 nM treatment with rapamycin in YPD(1)
(10) 439. Expression in strain Jk9-3da (wild type) in response to 30 min 50 nM treatment with rapamycin in YPD(1)
(11) 442. Expression in strain PM38 (wild type) in response to 30 min 50 nM treatment with rapamycin in YPD(1)
(12) 477. Expression in response to trichostatin A (TSA): 15min,30min,60min,120min(1)
(13) 477. Expression in response to trichostatin A (TSA): 15min,30min,60min,120min(3)
(14) 479. Expression in diploid cells in response to rapamycin (100nM) for: 15min,30min,90min,120min(2)
(15) 479. Expression in diploid cells in response to rapamycin (100nM) for: 15min,30min,90min,120min(4)
(16) 578. Brown enviromental changes :aa starv 0.5 h(1)
(17) 579. Brown enviromental changes :aa starv 1 h(1)
(18) 580. Brown enviromental changes :aa starv 2 h(1)
(19) 581. Brown enviromental changes :aa starv 4 h(1)
(20) 582. Brown enviromental changes :aa starv 6 h(1)
(21) 583. Brown enviromental changes :Nitrogen Depletion 30 min.(1)
(22) 584. Brown enviromental changes :Nitrogen Depletion 1 h(1)
(23) 585. Brown enviromental changes :Nitrogen Depletion 2 h(1)
(24) 586. Brown enviromental changes :Nitrogen Depletion 4 h(1)
(25) 587. Brown enviromental changes :Nitrogen Depletion 8 h(1)
(26) 588. Brown enviromental changes :Nitrogen Depletion 12 h(1)
(27) 589. Brown enviromental changes :Nitrogen Depletion 1 d(1)
(28) 590. Brown enviromental changes :Nitrogen Depletion 2 d(1)
(29) 591. Brown enviromental changes :Nitrogen Depletion 3 d(1)
(30) 592. Brown enviromental changes :Nitrogen Depletion 5 d(1)
(31) 670. Expression in response to antimycin 60min(1)
(32) 671. Expression in response to antimycin 120min(1)
(33) 675. Expression in response to propionate(1)
(34) DES460 (wt) - mock irradiation - 30 min

GLN3 --> DAL3 exp. conditions:34

(1) 5. Expression during the cell cycle (alpha factor arrest and release)(11)

(2) 89. Expression in response to 3-aminotriazole(1)
(3) 95. Expression in response to 50ug/mL FK506(1)
(4) 387. Rosetta 2000: Expression in cells with ERG11 under tet promoter(1)
(5) 402. Rosetta 2000: Expression in response to Itraconazole(1)
(6) 406. Rosetta 2000: Expression in response to Terbinafine(1)
(7) 407. Rosetta 2000: Expression in response to Tunicamycin(1)
(8) 428. Expression in strain PM38 (wild type) in response to 30 min 50 nM treatment with rapamycin in YPD(1)
(9) 429. Expression in strain YHE711 (wild type) in response to 30 min 50 nM treatment with rapamycin in YPD(1)
(10) 439. Expression in strain Jk9-3da (wild type) in response to 30 min 50 nM treatment with rapamycin in YPD(1)
(11) 442. Expression in strain PM38 (wild type) in response to 30 min 50 nM treatment with rapamycin in YPD(1)
(12) 477. Expression in response to trichostatin A (TSA): 15min,30min,60min,120min(1)
(13) 477. Expression in response to trichostatin A (TSA): 15min,30min,60min,120min(3)
(14) 479. Expression in diploid cells in response to rapamycin (100nM) for: 15min,30min,90min,120min(2)
(15) 479. Expression in diploid cells in response to rapamycin (100nM) for: 15min,30min,90min,120min(4)
(16) 578. Brown enviromental changes :aa starv 0.5 h(1)
(17) 579. Brown enviromental changes :aa starv 1 h(1)
(18) 580. Brown enviromental changes :aa starv 2 h(1)
(19) 581. Brown enviromental changes :aa starv 4 h(1)
(20) 582. Brown enviromental changes :aa starv 6 h(1)
(21) 583. Brown enviromental changes :Nitrogen Depletion 30 min.(1)
(22) 584. Brown enviromental changes :Nitrogen Depletion 1 h(1)
(23) 585. Brown enviromental changes :Nitrogen Depletion 2 h(1)
(24) 586. Brown enviromental changes :Nitrogen Depletion 4 h(1)
(25) 587. Brown enviromental changes :Nitrogen Depletion 8 h(1)
(26) 588. Brown enviromental changes :Nitrogen Depletion 12 h(1)
(27) 589. Brown enviromental changes :Nitrogen Depletion 1 d(1)
(28) 590. Brown enviromental changes :Nitrogen Depletion 2 d(1)
(29) 591. Brown enviromental changes :Nitrogen Depletion 3 d(1)
(30) 592. Brown enviromental changes :Nitrogen Depletion 5 d(1)
(31) 670. Expression in response to antimycin 60min(1)
(32) 671. Expression in response to antimycin 120min(1)
(33) 675. Expression in response to propionate(1)
(34) DES460 (wt) - mock irradiation - 30 min

GLN3 --> DAL4 exp. conditions:29

(1) 49. Expression in response to 50 nM alpha-factor: 0,15,30,45,60,90,120 min(5)
(2) 89. Expression in response to 3-aminotriazole(1)
(3) 95. Expression in response to 50ug/mL FK506(1)
(4) 407. Rosetta 2000: Expression in response to Tunicamycin(1)
(5) 428. Expression in strain PM38 (wild type) in response to 30 min 50 nM treatment with rapamycin in YPD(1)
(6) 429. Expression in strain YHE711 (wild type) in response to 30 min 50 nM treatment with rapamycin in YPD(1)
(7) 439. Expression in strain Jk9-3da (wild type) in response to 30 min 50 nM treatment with rapamycin in YPD(1)
(8) 442. Expression in strain PM38 (wild type) in response to 30 min 50 nM treatment with rapamycin in YPD(1)
(9) 479. Expression in diploid cells in response to rapamycin (100nM) for: 15min,30min,90min,120min(1)
(10) 479. Expression in diploid cells in response to rapamycin (100nM) for: 15min,30min,90min,120min(2)
(11) 578. Brown enviromental changes :aa starv 0.5 h(1)
(12) 579. Brown enviromental changes :aa starv 1 h(1)
(13) 580. Brown enviromental changes :aa starv 2 h(1)
(14) 581. Brown enviromental changes :aa starv 4 h(1)
(15) 582. Brown enviromental changes :aa starv 6 h(1)
(16) 583. Brown enviromental changes :Nitrogen Depletion 30 min.(1)
(17) 584. Brown enviromental changes :Nitrogen Depletion 1 h(1)
(18) 585. Brown enviromental changes :Nitrogen Depletion 2 h(1)
(19) 586. Brown enviromental changes :Nitrogen Depletion 4 h(1)
(20) 587. Brown enviromental changes :Nitrogen Depletion 8 h(1)
(21) 588. Brown enviromental changes :Nitrogen Depletion 12 h(1)
(22) 589. Brown enviromental changes :Nitrogen Depletion 1 d(1)
(23) 590. Brown enviromental changes :Nitrogen Depletion 2 d(1)
(24) 591. Brown enviromental changes :Nitrogen Depletion 3 d(1)
(25) 592. Brown enviromental changes :Nitrogen Depletion 5 d(1)
(26) 670. Expression in response to antimycin 60min(1)
(27) 671. Expression in response to antimycin 120min(1)
(28) DES460 (wt) - mock irradiation - 30 min
(29) MAC1-up (B)

GLN3 --> DAL5 exp. conditions:34

(1) 5. Expression during the cell cycle (alpha factor arrest and release)(11)
(2) 89. Expression in response to 3-aminotriazole(1)
(3) 95. Expression in response to 50ug/mL FK506(1)
(4) 387. Rosetta 2000: Expression in cells with ERG11 under tet promoter(1)
(5) 402. Rosetta 2000: Expression in response to Itraconazole(1)
(6) 406. Rosetta 2000: Expression in response to Terbinafine(1)
(7) 407. Rosetta 2000: Expression in response to Tunicamycin(1)
(8) 428. Expression in strain PM38 (wild type) in response to 30 min 50 nM treatment with rapamycin in YPD(1)
(9) 429. Expression in strain YHE711 (wild type) in response to 30 min 50 nM treatment with rapamycin in YPD(1)
(10) 439. Expression in strain Jk9-3da (wild type) in response to 30 min 50 nM treatment with rapamycin in YPD(1)
(11) 442. Expression in strain PM38 (wild type) in response to 30 min 50 nM treatment with rapamycin in YPD(1)
(12) 477. Expression in response to trichostatin A (TSA): 15min,30min,60min,120min(1)
(13) 477. Expression in response to trichostatin A (TSA): 15min,30min,60min,120min(3)
(14) 479. Expression in diploid cells in response to rapamycin (100nM) for: 15min,30min,90min,120min(2)
(15) 479. Expression in diploid cells in response to rapamycin (100nM) for: 15min,30min,90min,120min(4)
(16) 578. Brown enviromental changes :aa starv 0.5 h(1)
(17) 579. Brown enviromental changes :aa starv 1 h(1)
(18) 580. Brown enviromental changes :aa starv 2 h(1)
(19) 581. Brown enviromental changes :aa starv 4 h(1)
(20) 582. Brown enviromental changes :aa starv 6 h(1)
(21) 583. Brown enviromental changes :Nitrogen Depletion 30 min.(1)
(22) 584. Brown enviromental changes :Nitrogen Depletion 1 h(1)
(23) 585. Brown enviromental changes :Nitrogen Depletion 2 h(1)
(24) 586. Brown enviromental changes :Nitrogen Depletion 4 h(1)
(25) 587. Brown enviromental changes :Nitrogen Depletion 8 h(1)
(26) 588. Brown enviromental changes :Nitrogen Depletion 12 h(1)
(27) 589. Brown enviromental changes :Nitrogen Depletion 1 d(1)
(28) 590. Brown enviromental changes :Nitrogen Depletion 2 d(1)
(29) 591. Brown enviromental changes :Nitrogen Depletion 3 d(1)
(30) 592. Brown enviromental changes :Nitrogen Depletion 5 d(1)
(31) 670. Expression in response to antimycin 60min(1)
(32) 671. Expression in response to antimycin 120min(1)
(33) 675. Expression in response to propionate(1)
(34) DES460 (wt) - mock irradiation - 30 min

GLN3 --> DAL7 exp. conditions:35

(1) 8. Expression during the cell cycle (cell size selection and release)(1)

(2) 89. Expression in response to 3-aminotriazole(1)
(3) 95. Expression in response to 50ug/mL FK506(1)
(4) 387. Rosetta 2000: Expression in cells with ERG11 under tet promoter(1)
(5) 392. Rosetta 2000: Expression in cells with PMA1 under tet promoter(1)
(6) 402. Rosetta 2000: Expression in response to Itraconazole(1)
(7) 407. Rosetta 2000: Expression in response to Tunicamycin(1)
(8) 428. Expression in strain PM38 (wild type) in response to 30 min 50 nM treatment with rapamycin in YPD(1)
(9) 429. Expression in strain YHE711 (wild type) in response to 30 min 50 nM treatment with rapamycin in YPD(1)
(10) 439. Expression in strain Jk9-3da (wild type) in response to 30 min 50 nM treatment with rapamycin in YPD(1)
(11) 442. Expression in strain PM38 (wild type) in response to 30 min 50 nM treatment with rapamycin in YPD(1)
(12) 479. Expression in diploid cells in response to rapamycin (100nM) for: 15min,30min,90min,120min(2)
(13) 486. Expression in response to NaCl: 15 30 45 60 120 min(4)
(14) 495. Brown enviromental changes :Heat Shock 80 minutes hs-1(1)
(15) 578. Brown enviromental changes :aa starv 0.5 h(1)
(16) 579. Brown enviromental changes :aa starv 1 h(1)
(17) 580. Brown enviromental changes :aa starv 2 h(1)
(18) 581. Brown enviromental changes :aa starv 4 h(1)
(19) 582. Brown enviromental changes :aa starv 6 h(1)
(20) 583. Brown enviromental changes :Nitrogen Depletion 30 min.(1)
(21) 584. Brown enviromental changes :Nitrogen Depletion 1 h(1)
(22) 585. Brown enviromental changes :Nitrogen Depletion 2 h(1)
(23) 586. Brown enviromental changes :Nitrogen Depletion 4 h(1)
(24) 587. Brown enviromental changes :Nitrogen Depletion 8 h(1)
(25) 588. Brown enviromental changes :Nitrogen Depletion 12 h(1)
(26) 589. Brown enviromental changes :Nitrogen Depletion 1 d(1)
(27) 590. Brown enviromental changes :Nitrogen Depletion 2 d(1)
(28) 591. Brown enviromental changes :Nitrogen Depletion 3 d(1)
(29) 592. Brown enviromental changes :Nitrogen Depletion 5 d(1)
(30) 607. Brown enviromental changes :YPD 1 d ypd-2(1)
(31) 615. Brown enviromental changes :YPD stationary phase 1 d ypd-1(1)
(32) 616. Brown enviromental changes :YPD stationary phase 2 d ypd-1(1)
(33) 617. Brown enviromental changes :YPD stationary phase 3 d ypd-1(1)
(34) 670. Expression in response to antimycin 60min(1)
(35) 671. Expression in response to antimycin 120min(1)

GLN3 --> DCG1 exp. conditions:35

(1) 89. Expression in response to 3-aminotriazole(1)
(2) 95. Expression in response to 50ug/mL FK506(1)
(3) 387. Rosetta 2000: Expression in cells with ERG11 under tet promoter(1)
(4) 395. Rosetta 2000: Expression in response to 2-deoxy-D-glucose(1)
(5) 401. Rosetta 2000: Expression in response to HU(1)
(6) 402. Rosetta 2000: Expression in response to Itraconazole(1)
(7) 406. Rosetta 2000: Expression in response to Terbinafine(1)
(8) 407. Rosetta 2000: Expression in response to Tunicamycin(1)
(9) 428. Expression in strain PM38 (wild type) in response to 30 min 50 nM treatment with rapamycin in YPD(1)
(10) 429. Expression in strain YHE711 (wild type) in response to 30 min 50 nM treatment with rapamycin in YPD(1)
(11) 439. Expression in strain Jk9-3da (wild type) in response to 30 min 50 nM treatment with rapamycin in YPD(1)
(12) 442. Expression in strain PM38 (wild type) in response to 30 min 50 nM treatment with rapamycin in YPD(1)
(13) 479. Expression in diploid cells in response to rapamycin (100nM) for: 15min,30min,90min,120min(1)
(14) 479. Expression in diploid cells in response to rapamycin (100nM) for: 15min,30min,90min,120min(2)
(15) 556. Brown enviromental changes :dtt 480 min dtt-2(1)
(16) 578. Brown enviromental changes :aa starv 0.5 h(1)
(17) 579. Brown enviromental changes :aa starv 1 h(1)
(18) 580. Brown enviromental changes :aa starv 2 h(1)
(19) 581. Brown enviromental changes :aa starv 4 h(1)
(20) 582. Brown enviromental changes :aa starv 6 h(1)
(21) 583. Brown enviromental changes :Nitrogen Depletion 30 min.(1)
(22) 584. Brown enviromental changes :Nitrogen Depletion 1 h(1)
(23) 585. Brown enviromental changes :Nitrogen Depletion 2 h(1)
(24) 586. Brown enviromental changes :Nitrogen Depletion 4 h(1)
(25) 587. Brown enviromental changes :Nitrogen Depletion 8 h(1)
(26) 588. Brown enviromental changes :Nitrogen Depletion 12 h(1)
(27) 589. Brown enviromental changes :Nitrogen Depletion 1 d(1)
(28) 590. Brown enviromental changes :Nitrogen Depletion 2 d(1)
(29) 591. Brown enviromental changes :Nitrogen Depletion 3 d(1)
(30) 592. Brown enviromental changes :Nitrogen Depletion 5 d(1)
(31) 670. Expression in response to antimycin 60min(1)
(32) 671. Expression in response to antimycin 120min(1)
(33) 672. Expression in response to carbonyl cyanide m-chlorophenylhydrazone (CCCP) 90min(1)
(34) 675. Expression in response to propionate(1)
(35) DES460 + 0.02% MMS - 15 min

GLN3 --> GLN1 exp. conditions:22

(1) 6. Expression during the cell cycle (cdc15 arrest and release)(22)
(2) 6. Expression during the cell cycle (cdc15 arrest and release)(23)
(3) 89. Expression in response to 3-aminotriazole(1)
(4) 95. Expression in response to 50ug/mL FK506(1)
(5) 387. Rosetta 2000: Expression in cells with ERG11 under tet promoter(1)
(6) 402. Rosetta 2000: Expression in response to Itraconazole(1)
(7) 429. Expression in strain YHE711 (wild type) in response to 30 min 50 nM treatment with rapamycin in YPD(1)
(8) 439. Expression in strain Jk9-3da (wild type) in response to 30 min 50 nM treatment with rapamycin in YPD(1)
(9) 479. Expression in diploid cells in response to rapamycin (100nM) for: 15min,30min,90min,120min(1)
(10) 487. Expression in response to sorbitol: 15 30 45 90 120 min(3)
(11) 572. Brown enviromental changes :Hypo-osmotic shock - 5 min(1)
(12) 579. Brown enviromental changes :aa starv 1 h(1)
(13) 580. Brown enviromental changes :aa starv 2 h(1)
(14) 581. Brown enviromental changes :aa starv 4 h(1)
(15) 582. Brown enviromental changes :aa starv 6 h(1)
(16) 583. Brown enviromental changes :Nitrogen Depletion 30 min.(1)
(17) 584. Brown enviromental changes :Nitrogen Depletion 1 h(1)
(18) 585. Brown enviromental changes :Nitrogen Depletion 2 h(1)
(19) 586. Brown enviromental changes :Nitrogen Depletion 4 h(1)
(20) 670. Expression in response to antimycin 60min(1)
(21) 671. Expression in response to antimycin 120min(1)
(22) 672. Expression in response to carbonyl cyanide m-chlorophenylhydrazone (CCCP) 90min(1)

GLN3 --> GLT1 exp. conditions:27

(1) 5. Expression during the cell cycle (alpha factor arrest and release)(16)
(2) 6. Expression during the cell cycle (cdc15 arrest and release)(8)

(3) 6. Expression during the cell cycle (cdc15 arrest and release)(10)
(4) 7. Expression during the cell Cycle (cdc28)(10)
(5) 11. Expression during diauxic shift: 9h,11h,13h,15h,17h,19h,21h(2)
(6) 89. Expression in response to 3-aminotriazole(1)
(7) 95. Expression in response to 50ug/mL FK506(1)
(8) 387. Rosetta 2000: Expression in cells with ERG11 under tet promoter(1)
(9) 395. Rosetta 2000: Expression in response to 2-deoxy-D-glucose(1)
(10) 402. Rosetta 2000: Expression in response to Itraconazole(1)
(11) 403. Rosetta 2000: Expression in response to Lovastatin(1)
(12) 406. Rosetta 2000: Expression in response to Terbinafine(1)
(13) 407. Rosetta 2000: Expression in response to Tunicamycin(1)
(14) 579. Brown enviromental changes :aa starv 1 h(1)
(15) 580. Brown enviromental changes :aa starv 2 h(1)
(16) 581. Brown enviromental changes :aa starv 4 h(1)
(17) 585. Brown enviromental changes :Nitrogen Depletion 2 h(1)
(18) 586. Brown enviromental changes :Nitrogen Depletion 4 h(1)
(19) 588. Brown enviromental changes :Nitrogen Depletion 12 h(1)
(20) 591. Brown enviromental changes :Nitrogen Depletion 3 d(1)
(21) 594. Brown enviromental changes :diauxic shift timecourse(1)
(22) 670. Expression in response to antimycin 60min(1)
(23) 671. Expression in response to antimycin 120min(1)
(24) 672. Expression in response to carbonyl cyanide m-chlorophenylhydrazone (CCCP) 90min(1)
(25) 675. Expression in response to propionate(1)
(26) DES460 (wt) - mock irradiation - 30 min
(27) DES460 (wt) - mock irradiation - 90 min

GLN3 -*-> UGA3 exp. conditions:27

(1) 5. Expression during the cell cycle (alpha factor arrest and release)(16)
(2) 6. Expression during the cell cycle (cdc15 arrest and release)(8)
(3) 6. Expression during the cell cycle (cdc15 arrest and release)(10)
(4) 7. Expression during the cell Cycle (cdc28)(10)
(5) 11. Expression during diauxic shift: 9h,11h,13h,15h,17h,19h,21h(2)
(6) 89. Expression in response to 3-aminotriazole(1)
(7) 95. Expression in response to 50ug/mL FK506(1)
(8) 387. Rosetta 2000: Expression in cells with ERG11 under tet promoter(1)
(9) 395. Rosetta 2000: Expression in response to 2-deoxy-D-glucose(1)
(10) 402. Rosetta 2000: Expression in response to Itraconazole(1)
(11) 403. Rosetta 2000: Expression in response to Lovastatin(1)
(12) 406. Rosetta 2000: Expression in response to Terbinafine(1)
(13) 407. Rosetta 2000: Expression in response to Tunicamycin(1)
(14) 579. Brown enviromental changes :aa starv 1 h(1)
(15) 580. Brown enviromental changes :aa starv 2 h(1)
(16) 581. Brown enviromental changes :aa starv 4 h(1)
(17) 585. Brown enviromental changes :Nitrogen Depletion 2 h(1)
(18) 586. Brown enviromental changes :Nitrogen Depletion 4 h(1)
(19) 588. Brown enviromental changes :Nitrogen Depletion 12 h(1)
(20) 591. Brown enviromental changes :Nitrogen Depletion 3 d(1)
(21) 594. Brown enviromental changes :diauxic shift timecourse(1)
(22) 670. Expression in response to antimycin 60min(1)
(23) 671. Expression in response to antimycin 120min(1)
(24) 672. Expression in response to carbonyl cyanide m-chlorophenylhydrazone (CCCP) 90min(1)
(25) 675. Expression in response to propionate(1)
(26) DES460 (wt) - mock irradiation - 30 min
(27) DES460 (wt) - mock irradiation - 90 min

GLN3 -*-> YHR029C exp. conditions:32

(1) 5. Expression during the cell cycle (alpha factor arrest and release)(17)
(2) 89. Expression in response to 3-aminotriazole(1)
(3) 95. Expression in response to 50ug/mL FK506(1)
(4) 387. Rosetta 2000: Expression in cells with ERG11 under tet promoter(1)
(5) 402. Rosetta 2000: Expression in response to Itraconazole(1)
(6) 406. Rosetta 2000: Expression in response to Terbinafine(1)
(7) 407. Rosetta 2000: Expression in response to Tunicamycin(1)
(8) 445. Expression in response to 0.1% MMS for 60 min (average of 3 experiments)(1)
(9) 449. Expression in response to 0.1% MMS for 60 min(1)
(10) 451. Expression in response to BCNU (200 micromolar) for 60 min(1)
(11) 455. Expression in response to high MNNG (27 microgram/ml) for 60 min(1)
(12) 456. Expression in response to high 4NQO (8 microgram/ml) for 60 min(1)
(13) 464. Expression in response to 0.2% MMS for 60 min(1)
(14) 479. Expression in diploid cells in response to rapamycin (100nM) for: 15min,30min,90min,120min(1)
(15) 479. Expression in diploid cells in response to rapamycin (100nM) for: 15min,30min,90min,120min(2)
(16) 479. Expression in diploid cells in response to rapamycin (100nM) for: 15min,30min,90min,120min(3)
(17) 579. Brown enviromental changes :aa starv 1 h(1)
(18) 581. Brown enviromental changes :aa starv 4 h(1)
(19) 582. Brown enviromental changes :aa starv 6 h(1)
(20) 584. Brown enviromental changes :Nitrogen Depletion 1 h(1)
(21) 585. Brown enviromental changes :Nitrogen Depletion 2 h(1)
(22) 586. Brown enviromental changes :Nitrogen Depletion 4 h(1)
(23) 587. Brown enviromental changes :Nitrogen Depletion 8 h(1)
(24) 588. Brown enviromental changes :Nitrogen Depletion 12 h(1)
(25) 589. Brown enviromental changes :Nitrogen Depletion 1 d(1)
(26) 590. Brown enviromental changes :Nitrogen Depletion 2 d(1)
(27) 591. Brown enviromental changes :Nitrogen Depletion 3 d(1)
(28) 592. Brown enviromental changes :Nitrogen Depletion 5 d(1)
(29) 670. Expression in response to antimycin 60min(1)
(30) 671. Expression in response to antimycin 120min(1)
(31) 672. Expression in response to carbonyl cyanide m-chlorophenylhydrazone (CCCP) 90min(1)
(32) DES460 (wt) - mock irradiation - 30 min

HAP2 --> COX4 exp. conditions:22

(1) 11. Expression during diauxic shift: 9h,11h,13h,15h,17h,19h,21h(5)
(2) 11. Expression during diauxic shift: 9h,11h,13h,15h,17h,19h,21h(6)
(3) 11. Expression during diauxic shift: 9h,11h,13h,15h,17h,19h,21h(7)
(4) 390. Rosetta 2000: Expression in cells with IDI1 under tet promoter(1)
(5) 428. Expression in strain PM38 (wild type) in response to 30 min 50 nM treatment with rapamycin in YPD(1)
(6) 479. Expression in diploid cells in response to rapamycin (100nM) for: 15min,30min,90min,120min(3)
(7) 597. Brown enviromental changes :diauxic shift timecourse(1)
(8) 598. Brown enviromental changes :diauxic shift timecourse(1)
(9) 599. Brown enviromental changes :diauxic shift timecourse(1)

(10) 600. Brown enviromental changes :diauxic shift timecourse(1)
(11) 602. Brown enviromental changes :YPD 4 h ypd-2(1)
(12) 603. Brown enviromental changes :YPD 6 h ypd-2(1)
(13) 604. Brown enviromental changes :YPD 8 h ypd-2(1)
(14) 605. Brown enviromental changes :YPD 10 h ypd-2(1)
(15) 606. Brown enviromental changes :YPD 12 h ypd-2(1)
(16) 608. Brown enviromental changes :YPD 2 d ypd-2(1)
(17) 612. Brown enviromental changes :YPD stationary phase 4 h ypd-1(1)
(18) 613. Brown enviromental changes :YPD stationary phase 8 h ypd-1(1)
(19) 614. Brown enviromental changes :YPD stationary phase 12 h ypd-1(1)
(20) 615. Brown enviromental changes :YPD stationary phase 1 d ypd-1(1)
(21) 616. Brown enviromental changes :YPD stationary phase 2 d ypd-1(1)
(22) 617. Brown enviromental changes :YPD stationary phase 3 d ypd-1(1)

HAP2 --> COX6 exp. conditions:22

(1) 11. Expression during diauxic shift: 9h,11h,13h,15h,17h,19h,21h(5)
(2) 11. Expression during diauxic shift: 9h,11h,13h,15h,17h,19h,21h(6)
(3) 11. Expression during diauxic shift: 9h,11h,13h,15h,17h,19h,21h(7)
(4) 390. Rosetta 2000: Expression in cells with IDI1 under tet promoter(1)
(5) 428. Expression in strain PM38 (wild type) in response to 30 min 50 nM treatment with rapamycin in YPD(1)
(6) 479. Expression in diploid cells in response to rapamycin (100nM) for: 15min,30min,90min,120min(3)
(7) 597. Brown enviromental changes :diauxic shift timecourse(1)
(8) 598. Brown enviromental changes :diauxic shift timecourse(1)
(9) 599. Brown enviromental changes :diauxic shift timecourse(1)
(10) 600. Brown enviromental changes :diauxic shift timecourse(1)
(11) 602. Brown enviromental changes :YPD 4 h ypd-2(1)
(12) 603. Brown enviromental changes :YPD 6 h ypd-2(1)
(13) 604. Brown enviromental changes :YPD 8 h ypd-2(1)
(14) 605. Brown enviromental changes :YPD 10 h ypd-2(1)
(15) 606. Brown enviromental changes :YPD 12 h ypd-2(1)
(16) 608. Brown enviromental changes :YPD 2 d ypd-2(1)
(17) 612. Brown enviromental changes :YPD stationary phase 4 h ypd-1(1)
(18) 613. Brown enviromental changes :YPD stationary phase 8 h ypd-1(1)
(19) 614. Brown enviromental changes :YPD stationary phase 12 h ypd-1(1)
(20) 615. Brown enviromental changes :YPD stationary phase 1 d ypd-1(1)
(21) 616. Brown enviromental changes :YPD stationary phase 2 d ypd-1(1)
(22) 617. Brown enviromental changes :YPD stationary phase 3 d ypd-1(1)

HAP2 -*-> QCR7 exp. conditions:22

(1) 11. Expression during diauxic shift: 9h,11h,13h,15h,17h,19h,21h(5)
(2) 11. Expression during diauxic shift: 9h,11h,13h,15h,17h,19h,21h(6)
(3) 11. Expression during diauxic shift: 9h,11h,13h,15h,17h,19h,21h(7)
(4) 390. Rosetta 2000: Expression in cells with IDI1 under tet promoter(1)
(5) 428. Expression in strain PM38 (wild type) in response to 30 min 50 nM treatment with rapamycin in YPD(1)
(6) 479. Expression in diploid cells in response to rapamycin (100nM) for: 15min,30min,90min,120min(3)
(7) 597. Brown enviromental changes :diauxic shift timecourse(1)
(8) 598. Brown enviromental changes :diauxic shift timecourse(1)
(9) 599. Brown enviromental changes :diauxic shift timecourse(1)
(10) 600. Brown enviromental changes :diauxic shift timecourse(1)
(11) 602. Brown enviromental changes :YPD 4 h ypd-2(1)
(12) 603. Brown enviromental changes :YPD 6 h ypd-2(1)
(13) 604. Brown enviromental changes :YPD 8 h ypd-2(1)
(14) 605. Brown enviromental changes :YPD 10 h ypd-2(1)
(15) 606. Brown enviromental changes :YPD 12 h ypd-2(1)
(16) 608. Brown enviromental changes :YPD 2 d ypd-2(1)
(17) 612. Brown enviromental changes :YPD stationary phase 4 h ypd-1(1)
(18) 613. Brown enviromental changes :YPD stationary phase 8 h ypd-1(1)
(19) 614. Brown enviromental changes :YPD stationary phase 12 h ypd-1(1)
(20) 615. Brown enviromental changes :YPD stationary phase 1 d ypd-1(1)
(21) 616. Brown enviromental changes :YPD stationary phase 2 d ypd-1(1)
(22) 617. Brown enviromental changes :YPD stationary phase 3 d ypd-1(1)
