# Supplementary material for: Unraveling condition specific gene transcriptional regulatory networks in Saccharomyces cerevisiae
Source: BMC Bioinformatics. 2006 Mar 21;7:165. doi: 10.1186/1471-2105-7-165 (PMC1488875; doi:10.1186/1471-2105-7-165)
Supplement: Additional File 5 — The Union of Alon's and Palsson's transcriptional regulatory sub-networks. Sparse representation of this unified network where column one represents the TF and column two represents the target. Entry of 1(2) corresponds to activation (suppression) [file 1471-2105-7-165-S5.pdf]

COLUMN 1: TF
COLUMN 2: TARGET
COLOMN 3: 1=ACTIVATION, 2=SUPPRESSION

YCR097W	YCL066W	2
YCR097W	YIR019C	2
YCR097W	YGR044C	2
YCR097W	YDR103W	2
YCR097W	YNL283C	2
YKL112W	YAL054C	1
YKL112W	YOL086C	1
YKL112W	YAL038W	1
YKL112W	YHR051W	1
YKL112W	YOR204W	1
YKL112W	YCR012W	1
YKL112W	YIL160C	1
YKL112W	YJL166W	1
YKL112W	YBR031W	1
YKL112W	YDR012W	1
YKL112W	YGR059W	1
YKL112W	YFL037W	1
YLR131C	YKL185W	1
YLR131C	YJL194W	1
YLR131C	YLR286C	1
YLR131C	YHR053C	1
YLR131C	YHR055C	1
YLR131C	YNL327W	1
YLR131C	YDL227C	1
YLR131C	YMR043W	1
YLR131C	YDL127W	1
YLR131C	YDL179W	1
YLR131C	YGR044C	1
YLR131C	YLR079W	1
YDR448W	YJL153C	1
YDR448W	YBL103C	1
YDR448W	YIL162W	1
YDR216W	YAL054C	1
YDR216W	YOL086C	1
YDR216W	YMR303C	1
YDR216W	YDR256C	1
YDR216W	YKR009C	1
YDR216W	YHL032C	1
YDR216W	YKL197C	1
YDR216W	YIL160C	1
YMR042W	YOL058W	2
YMR042W	YJL088W	2
YMR042W	YER069W	2
YMR042W	YOL140W	2
YMR042W	YPL111W	1
YMR042W	YLR438W	2
YML099C	YOL058W	2
YML099C	YJL088W	2
YML099C	YER069W	2
YML099C	YOL140W	2
YML099C	YPL111W	1
YML099C	YLR438W	2
YDR421W	YDR380W	1
YDR421W	YHR137W	1
YKL185W	YLR131C	2
YKL185W	YIR019C	1
YKL185W	YDR146C	2
YKR099W	YAR015W	1
YKR099W	YNL220W	1
YKR099W	YLR359W	1
YKR099W	YMR120C	1
YKR099W	YOR128C	1
YKR099W	YGR204W	1
YKR099W	YMR300C	1
YKR099W	YGL234W	1
YKR099W	YGR061C	1
YKR099W	YDR408C	1
YKR099W	YER055C	1
YKR099W	YCL030C	1
YKR099W	YIL020C	1
YKR099W	YBR248C	1
YDR423C	YGR088W	1
YDR423C	YJL101C	1
YDR423C	YOR202W	1
YDR423C	YFL014W	1
YDR423C	YOR208W	1
YDR423C	YAL005C	1
YDR423C	YBR126C	1
YDR423C	YDR074W	1
YDR423C	YMR261C	1
YDR423C	YLR043C	1
YDR423C	YGR209C	1
YDR423C	YML100W	1
YDR423C	YOL052CA	1
YFL028C	YOL086C	2
YFL028C	YLR342W	1
YFL028C	YDL227C	1
YKR036C	YOL086C	2
YKR036C	YLR342W	1
YKR036C	YBR020W	2
YKR036C	YDL227C	1
YMR280C	YAL054C	1
YMR280C	YOR125C	1
YMR280C	YLR377C	1
YMR280C	YLR174W	2
YMR280C	YKL217W	2
YMR280C	YOL126C	1
YMR280C	YGL035C	1
YMR280C	YGL209W	1
YMR280C	YKR097W	1
YJR060W	YOR065W	1
YJR060W	YLR081W	1
YJR060W	YKL001C	1
YJR060W	YPR167C	1
YJR060W	YLR303W	1
YJR060W	YNL277W	1
YJR060W	YIR017C	1
YJR060W	YJR010W	1

YJR060W	YNL103W	1
YJR060W	YBR213W	1
YJR060W	YJL166W	2
YJR060W	YDR502C	1
YJR060W	YDR007W	1
YAL021C	YOL086C	2
YAL021C	YMR303C	1
YAL021C	YHR119W	1
YDL165W	YCL027W	2
YCR093W	YJR048W	2
YCR093W	YCL027W	2
YCR093W	YOR202W	2
YCR094W	YJR048W	2
YLR098C	YCL064C	1
YOR028C	YDR040C	1
YNL027W	YNL027W	1
YNL027W	YDR040C	1
YNL027W	YGR032W	1
YNL027W	YGL006W	1
YGL166W	YOR031W	1
YGL166W	YHR053C	1
YGL166W	YHR055C	1
YGL166W	YJR104C	1
YPL177C	YMR058W	1
YPL177C	YKR093W	2
YBR112C	YCR005C	1
YBR112C	YHR051W	2
YBR112C	YJR048W	2
YBR112C	YAR050W	2
YBR112C	YHR211W	2
YBR112C	YAL063C	2
YBR112C	YLR256W	2
YBR112C	YHR094C	2
YBR112C	YDR345C	2
YBR112C	YER065C	1
YBR112C	YJR094C	2
YBR112C	YIR019C	2
YBR112C	YBR182C	2
YBR112C	YIL162W	2
YBR112C	YDR172W	2
YKR034W	YEL063C	2
YKR034W	YIR027C	2
YKR034W	YIR029W	2
YKR034W	YIR032C	2
YKR034W	YIR028W	2
YKR034W	YJR152W	2
YKR034W	YIR031C	2
YKR034W	YIR030C	2
YKR034W	YBR208C	2
YKR034W	YHL016C	2
YKR034W	YKR039W	2
YKR034W	YFL021W	2
YKR034W	YOR375C	2
YKR034W	YER040W	2
YKR034W	YJL110C	2
YKR034W	YLR142W	2
YKR034W	YHR037W	2
YKR034W	YOR348C	2
YKR034W	YGR019W	2
YKR034W	YBR006W	2
YKR034W	YDL210W	2
YIR023W	YCL025C	1
YIR023W	YIR027C	1
YIR023W	YIR029W	1
YIR023W	YIR028W	1
YIR023W	YIR031C	1
YIR023W	YBR208C	1
YIR023W	YHL016C	1
YIR023W	YGR019W	1
YIR023W	YBR006W	1
YIR023W	YDL210W	1
YNL314W	YIR027C	1
YNL314W	YIR029W	1
YNL314W	YIR028W	1
YNL314W	YIR031C	1
YNL314W	YBR208C	1
YNL314W	YHL016C	1
YGL043W	YLR420W	2
YNL068C	YPR119W	2
YER109C	YIR019C	1
YGL254W	YPL092W	1
YOL051W	YBR020W	1
YOL051W	YBR019C	1
YOL051W	YLR081W	1
YOL051W	YBR018C	1
YOL051W	YCL066W	2
YOL051W	YCL067C	1
YOL051W	YPL187W	1
YOL051W	YDL140C	1
YPL248C	YBR021W	1
YPL248C	YBR020W	1
YPL248C	YBR019C	1
YPL248C	YLR081W	1
YPL248C	YDR009W	1
YPL248C	YBR018C	1
YPL248C	YML051W	1
YPL248C	YOR120W	1
YPL248C	YNL239W	1
YPL248C	YGL035C	1
YPL248C	YGL209W	1
YPL248C	YDR277C	1
YPL248C	YGL134W	1
YPL248C	YMR105C	1
YFL021W	YKR034W	1
YFL021W	YKR039W	1
YFL021W	YPR035W	1
YEL009C	YAR015W	1
YEL009C	YOR128C	1
YEL009C	YMR300C	1
YEL009C	YDR408C	1
YEL009C	YOL058W	1
YEL009C	YJL071W	1

YEL009C	YJL088W	1
YEL009C	YHR018C	1
YEL009C	YER069W	1
YEL009C	YOL140W	1
YEL009C	YDR035W	1
YEL009C	YBR249C	1
YEL009C	YPR060C	1
YEL009C	YPR145W	1
YEL009C	YGR124W	1
YEL009C	YOR303W	1
YEL009C	YJR109C	1
YEL009C	YIR027C	1
YEL009C	YJR152W	1
YEL009C	YMR062C	1
YEL009C	YAL044C	1
YEL009C	YPR035W	1
YEL009C	YDL171C	1
YEL009C	YPR005C	1
YEL009C	YER055C	1
YEL009C	YFR025C	1
YEL009C	YOR202W	1
YEL009C	YCL030C	1
YEL009C	YIL116W	1
YEL009C	YBR248C	1
YEL009C	YDR158W	1
YEL009C	YER052C	1
YEL009C	YER086W	1
YEL009C	YMR108W	1
YEL009C	YLR355C	1
YEL009C	YDR037W	1
YEL009C	YLR451W	1
YEL009C	YNL104C	1
YEL009C	YFL018C	1
YEL009C	YIR034C	1
YEL009C	YBR115C	1
YEL009C	YPR167C	1
YEL009C	YLR303W	1
YEL009C	YNL103W	1
YEL009C	YDR300C	1
YEL009C	YOR184W	1
YEL009C	YLR058C	1
YEL009C	YHR025W	1
YEL009C	YER090W	1
YEL009C	YKL211C	1
YEL009C	YDR354W	1
YEL009C	YGL026C	1
YPL075W	YOL086C	1
YPL075W	YCL050C	1
YPL075W	YAL038W	1
YPL075W	YJR048W	1
YPL075W	YGR254W	1
YPL075W	YHR174W	1
YPL075W	YCL040W	1
YPL075W	YKL152C	1
YPL075W	YCL030C	1
YPL075W	YLR044C	1
YPL075W	YCR012W	1
YPL075W	YGL031C	1
YPL075W	YCR031C	1
YPL075W	YJL191W	1
YPL075W	YLR372W	1
YPL075W	YPR080W	1
YPL075W	YBR118W	1
YPL075W	YDR050C	1
YDR096W	YOR386W	2
YER040W	YLR155C	1
YER040W	YLR157C	1
YER040W	YLR158C	1
YER040W	YLR160C	1
YER040W	YEL063C	1
YER040W	YIR027C	1
YER040W	YIR029W	1
YER040W	YIR032C	1
YER040W	YIR028W	1
YER040W	YJR152W	1
YER040W	YIR031C	1
YER040W	YKR034W	1
YER040W	YIR030C	1
YER040W	YBR208C	1
YER040W	YHL016C	1
YER040W	YBR021W	1
YER040W	YKR039W	1
YER040W	YFL021W	1
YER040W	YOR375C	1
YER040W	YDL215C	1
YER040W	YPR035W	1
YER040W	YDL171C	1
YER040W	YJL110C	1
YER040W	YEL060C	1
YER040W	YLR142W	1
YER040W	YHR037W	1
YER040W	YOR348C	1
YER040W	YGR019W	1
YER040W	YDL210W	1
YJL110C	YKR034W	2
YJL110C	YKR039W	2
YJL110C	YER040W	2
YJL110C	YDL210W	2
YFL031W	YDR518W	1
YFL031W	YDR519W	1
YFL031W	YJR094C	1
YFL031W	YJL034W	1
YFL031W	YCL043C	1
YOL089C	YDR040C	1
YLR256W	YLR304C	1
YLR256W	YGR088W	1
YLR256W	YML054C	1
YLR256W	YJR048W	1
YLR256W	YEL039C	1
YLR256W	YBR112C	2
YLR256W	YOR065W	1
YLR256W	YHR007C	2

YLR256W	YDR044W	1
YLR256W	YML075C	1
YLR256W	YEL034W	1
YLR256W	YPR191W	1
YLR256W	YPR065W	1
YLR256W	YJR104C	1
YLR256W	YHR008C	1
YGL237C	YMR056C	1
YGL237C	YLR304C	1
YGL237C	YPR145W	1
YGL237C	YNR001C	1
YGL237C	YGL187C	1
YGL237C	YHR051W	1
YGL237C	YJR048W	1
YGL237C	YOR065W	1
YGL237C	YOR375C	1
YGL237C	YDR232W	1
YGL237C	YDL205C	1
YGL237C	YIL125W	1
YGL237C	YDR148C	1
YGL237C	YFL018C	1
YGL237C	YBL030C	1
YGL237C	YDL230W	1
YGL237C	YPR191W	1
YGL237C	YJL166W	1
YGL237C	YML091C	1
YGL237C	YKL148C	1
YGL237C	YKL141W	1
YGL237C	YDR178W	1
YGL237C	YHR008C	1
YGL237C	YGR059W	1
YBL021C	YPR145W	1
YBL021C	YNR001C	1
YBL021C	YGL187C	1
YBL021C	YHR051W	1
YBL021C	YJR048W	1
YBL021C	YOR065W	1
YBL021C	YOR375C	1
YBL021C	YDR232W	1
YBL021C	YDL205C	1
YBL021C	YIL125W	1
YBL021C	YDR148C	1
YBL021C	YBL030C	1
YBL021C	YDL230W	1
YBL021C	YPR191W	1
YBL021C	YJL166W	1
YBL021C	YML091C	1
YBL021C	YKL148C	1
YBL021C	YKL141W	1
YBL021C	YHR008C	1
YBL021C	YGR059W	1
YKL109W	YNR001C	1
YKL109W	YGL187C	1
YKL109W	YHR051W	1
YKL109W	YJR048W	1
YKL109W	YOR065W	1
YKL109W	YOR375C	1
YKL109W	YDR232W	1
YKL109W	YDL205C	1
YKL109W	YIL125W	1
YKL109W	YDR148C	1
YKL109W	YFL018C	1
YKL109W	YKL085W	1
YKL109W	YGL035C	1
YKL109W	YGL209W	1
YKL109W	YBL030C	1
YKL109W	YDL230W	1
YKL109W	YPR191W	1
YKL109W	YJL166W	1
YKL109W	YML091C	1
YKL109W	YKL148C	1
YKL109W	YKL141W	1
YKL109W	YHR008C	1
YKL109W	YGR059W	1
YOR358W	YHR008C	1
YCR065W	YMR198W	2
YCR065W	YGR098C	2
YCR065W	YDR356W	2
YCR065W	YDR113C	2
YNL021W	YPL028W	2
YNL021W	YDL181W	2
YNL021W	YJL153C	2
YNL021W	YBR092C	2
YNL021W	YBR093C	2
YBL008W	YDR225W	2
YBL008W	YML010W	2
YCL066W	YCR097W	2
YCL066W	YOL051W	2
YCL066W	YCL067C	2
YCL066W	YLR113W	1
YCL066W	YGL089C	1
YCL066W	YPL187W	1
YCL066W	YIR019C	2
YCL066W	YNL216W	2
YCL066W	YJR004C	1
YCL066W	YIL099W	1
YCL066W	YKL178C	1
YCL066W	YCR084C	2
YCL066W	YNL283C	2
YCL067C	YIL015W	2
YCL067C	YCL066W	2
YCL067C	YGL089C	2
YCL067C	YPL187W	1
YCL067C	YNL216W	1
YCL067C	YGR044C	2
YCL067C	YLR452C	2
YCL067C	YHR084W	2
YCL067C	YFL026W	2
YCL067C	YOR212W	2
YCL067C	YDR103W	2
YCL067C	YKL209C	2
YCL067C	YDR007W	2

```
YGL073W    YHR053C    1
YGL073W    YHR055C    1
YGL073W    YMR173W    1
YGL073W    YMR186W    1
YGL073W    YLL026W    1
YGL073W    YFL014W    1
YGL073W    YBR072W    1
YGL073W    YPL240C    1
YGL073W    YJL034W    1
YGL073W    YIL148W    1
YGL073W    YNL007C    1
YGL073W    YAL005C    1
YGL073W    YBL075C    1
YGL073W    YER103W    1
YGL073W    YPL106C    1
YGL073W    YLL039C    1
YGL073W    YDR207C    1
YJR094C    YBR112C    1
YJR094C    YDR403W    1
YJR094C    YFL031W    1
YJR094C    YIL072W    1
YJR094C    YJL106W    1
YJR094C    YNL210W    1
YJR094C    YMR133W    1
YJR094C    YHL027W    1
YJR094C    YMR139W    1
YJR094C    YHL024W    1
YJR094C    YGR044C    1
YJR094C    YIL099W    1
YJR094C    YOR159C    1
YJR094C    YHL022C    1
YJR094C    YHR014W    1
YJR094C    YDR523C    1
YJR094C    YDR522C    1
YJR094C    YOR313C    1
YJR094C    YCR084C    1
YJR094C    YDR207C    1
YGL192W    YJR094C    1
YDR123C    YNR016C    2
YDR123C    YDR226W    1
YDR123C    YER026C    2
YDR123C    YGR157W    2
YDR123C    YLR133W    2
YDR123C    YKL182W    2
YDR123C    YPL231W    2
YDR123C    YGL077C    2
YDR123C    YJL153C    2
YDR123C    YDR497C    2
YDR123C    YJR073C    2
YDR123C    YBR093C    2
YDR123C    YGL008C    1
YDR123C    YDR207C    2
YOL108C    YNR016C    1
YOL108C    YER026C    1
YOL108C    YGR157W    1
YOL108C    YLR133W    1
YOL108C    YKL182W    1
YOL108C    YPL231W    1
YOL108C    YGL077C    1
YOL108C    YJL153C    1
YOL108C    YOL108C    1
YOL108C    YDR497C    1
YOL108C    YJR073C    1
YOL108C    YBR093C    1
YKL032C    YIL111W    2
YCL055W    YMR198W    1
YCL055W    YPR141C    1
YLR451W    YDR046C    2
YLR451W    YOR375C    2
YLR451W    YER086W    2
YLR451W    YMR108W    1
YLR451W    YLR355C    1
YLR451W    YGL009C    2
YLR451W    YCL018W    2
YLR451W    YNL104C    2
YDR034C    YIR034C    1
YDR034C    YIL094C    1
YDR034C    YBR115C    1
YDR034C    YGL154C    1
YDR034C    YNR050C    1
YMR021C    YLR213C    1
YMR021C    YDR256C    1
YMR021C    YPR124W    1
YMR021C    YLR411W    1
YMR021C    YGR088W    1
YMR021C    YLR214W    1
YMR021C    YOL152W    1
YMR021C    YFR055W    1
YMR021C    YJL217W    1
YGR288W    YGR289C    1
YGR288W    YGR292W    1
YGR288W    YHL009C    1
YBR297W    YBR298C    1
YBR297W    YBR299W    1
YDL056W    YDL102W    2
YDL056W    YOR074C    2
YDL056W    YJL194W    2
YDL056W    YDL164C    2
YDL056W    YPR120C    2
YDL056W    YGR109C    2
YDL056W    YMR199W    2
YDL056W    YPL256C    2
YDL056W    YNL102W    2
YMR043W    YLR131C    1
YMR043W    YER069W    1
YMR043W    YPR200C    1
YMR043W    YIL015W    1
YMR043W    YPL111W    1
YMR043W    YLR438W    2
YMR043W    YKR066C    1
YMR043W    YBR160W    1
YMR043W    YLR274W    1
```

YMR043W	YBR202W	1
YMR043W	YMR001C	1
YMR043W	YJL194W	1
YMR043W	YGR108W	1
YMR043W	YPR119W	1
YMR043W	YAL040C	1
YMR043W	YDR403W	1
YMR043W	YJL157C	1
YMR043W	YJL159W	1
YMR043W	YNL277W	1
YMR043W	YGL089C	1
YMR043W	YDR461W	1
YMR043W	YNL145W	1
YMR043W	YPL187W	1
YMR043W	YKR097W	1
YMR043W	YPR113W	1
YMR043W	YGL008C	1
YMR043W	YFL026W	1
YMR043W	YKL178C	1
YMR043W	YKL209C	1
YMR043W	YER111C	1
YMR043W	YDR146C	1
YIR017C	YJR060W	1
YIR017C	YFR030W	1
YIR017C	YKL001C	1
YIR017C	YPR167C	1
YIR017C	YJR010W	1
YIR017C	YNL103W	1
YIR017C	YIR018W	1
YNL103W	YJR060W	1
YNL103W	YEL009C	1
YNL103W	YKL001C	1
YNL103W	YPR167C	1
YNL103W	YLR303W	1
YNL103W	YNL277W	1
YNL103W	YIR017C	1
YNL103W	YJR010W	1
YNL103W	YER091C	1
YGL035C	YMR280C	2
YGL035C	YJR048W	2
YGL035C	YHR043C	2
YGL035C	YDR040C	2
YGL035C	YLR377C	2
YGL035C	YLL043W	2
YGL035C	YBR020W	2
YGL035C	YBR019C	2
YGL035C	YDR009W	2
YGL035C	YPL248C	2
YGL035C	YGL237C	2
YGL035C	YKL109W	2
YGL035C	YFR053C	2
YGL035C	YHR094C	2
YGL035C	YEL069C	2
YGL035C	YMR011W	2
YGL035C	YDR345C	2
YGL035C	YHR092C	2
YGL035C	YKL217W	2
YGL035C	YBR299W	2
YGL035C	YGL209W	2
YGL035C	YLR044C	2
YGL035C	YBR050C	2
YGL035C	YIL162W	2
YGL035C	YDR146C	2
YGL035C	YBR126C	2
YGL035C	YBR101C	2
YGL035C	YDR516C	2
YGL035C	YEL070W	2
YGL035C	YFL054C	2
YGL035C	YKR075C	2
YGL035C	YLR042C	2
YGL209W	YMR280C	2
YGL209W	YHR043C	2
YGL209W	YDR040C	2
YGL209W	YLR377C	2
YGL209W	YLL043W	2
YGL209W	YBR020W	2
YGL209W	YBR019C	2
YGL209W	YDR009W	2
YGL209W	YPL248C	2
YGL209W	YKL109W	2
YGL209W	YHR094C	2
YGL209W	YEL069C	2
YGL209W	YMR011W	2
YGL209W	YDR345C	2
YGL209W	YHR092C	2
YGL209W	YKL217W	2
YGL209W	YGL035C	2
YGL209W	YLR044C	2
YGL209W	YBR050C	2
YGL209W	YIL162W	2
YGL209W	YDR146C	2
YGL209W	YBR126C	2
YGL209W	YBR101C	2
YGL209W	YDR516C	2
YGL209W	YEL070W	2
YGL209W	YFL054C	2
YGL209W	YKR075C	2
YGL209W	YLR042C	2
YPL082C	YCL030C	1
YPL082C	YPL089C	1
YMR070W	YBR085W	2
YMR070W	YNR044W	2
YMR070W	YJR047C	2
YMR070W	YJR048W	1
YMR070W	YCL027W	2
YMR070W	YDR044W	2
YMR070W	YPR141C	2
YMR070W	YCL018W	1
YMR070W	YLR452C	2
YMR070W	YIL162W	1
YOL116W	YFL014W	1
YOL116W	YIR019C	1

YOL116W	YNL283C	1
YMR037C	YMR170C	1
YMR037C	YMR169C	1
YMR037C	YBR149W	1
YMR037C	YDR155C	1
YMR037C	YGR088W	1
YMR037C	YHR043C	1
YMR037C	YAL062W	1
YMR037C	YCL040W	1
YMR037C	YML004C	1
YMR037C	YHR104W	1
YMR037C	YCL035C	1
YMR037C	YER062C	1
YMR037C	YLL026W	1
YMR037C	YFL014W	1
YMR037C	YBR072W	1
YMR037C	YDR171W	1
YMR037C	YDR258C	1
YMR037C	YFR053C	1
YMR037C	YFL016C	1
YMR037C	YGL036W	1
YMR037C	YMR105C	1
YMR037C	YGL037C	1
YMR037C	YNL098C	1
YMR037C	YIL066C	1
YMR037C	YHR008C	1
YMR037C	YHR139C	1
YMR037C	YBL075C	1
YMR037C	YER103W	1
YMR037C	YBR117C	1
YMR037C	YBR126C	1
YMR037C	YDR074W	1
YMR037C	YDR513W	1
YMR037C	YLL039C	1
YMR037C	YGR086C	1
YMR037C	YKL151C	1
YMR037C	YNL077W	1
YMR037C	YOL052CA	1
YKL062W	YMR170C	1
YKL062W	YMR169C	1
YKL062W	YBR149W	1
YKL062W	YDR155C	1
YKL062W	YGR088W	1
YKL062W	YHR043C	1
YKL062W	YAL062W	1
YKL062W	YCL040W	1
YKL062W	YML004C	1
YKL062W	YHR104W	1
YKL062W	YCL035C	1
YKL062W	YER062C	1
YKL062W	YLL026W	1
YKL062W	YFL014W	1
YKL062W	YBR072W	1
YKL062W	YDR171W	1
YKL062W	YDR258C	1
YKL062W	YFR053C	1
YKL062W	YFL016C	1
YKL062W	YGL036W	1
YKL062W	YMR105C	1
YKL062W	YGL037C	1
YKL062W	YNL098C	1
YKL062W	YIL066C	1
YKL062W	YHR008C	1
YKL062W	YHR139C	1
YKL062W	YBL075C	1
YKL062W	YER103W	1
YKL062W	YBR117C	1
YKL062W	YBR126C	1
YKL062W	YDR074W	1
YKL062W	YDR513W	1
YKL062W	YGR086C	1
YKL062W	YNL077W	1
YKL062W	YOL052CA	1
YDR277C	YHR094C	1
YDR277C	YDR345C	1
YHR124W	YOR339C	1
YDR176W	YJL153C	1
YDR176W	YBL103C	1
YDR176W	YIL162W	1
YDR043C	YIR019C	2
YAL051W	YIL120W	1
YBR279W	YPL256C	1
YGL013C	YBR008C	1
YGL013C	YMR307W	1
YGL013C	YOL156W	1
YGL013C	YJL219W	1
YGL013C	YDR072C	1
YGL013C	YOR328W	1
YGL013C	YIL013C	1
YGL013C	YDR406W	1
YGL013C	YBL005W	1
YGL013C	YOR153W	1
YGL013C	YDR011W	1
YGL013C	YKL209C	1
YGL013C	YIL101C	1
YGL013C	YGR281W	1
YGL013C	YOR162C	1
YBL005W	YBR008C	1
YBL005W	YOL156W	1
YBL005W	YJL219W	1
YBL005W	YDR072C	1
YBL005W	YGL013C	1
YBL005W	YOR328W	1
YBL005W	YDR406W	1
YBL005W	YBL005W	1
YBL005W	YOR153W	1
YBL005W	YDR011W	1
YBL005W	YGR281W	1
YGL025C	YFL039C	1
YGL025C	YBR020W	1
YGL025C	YBR072W	2
YGL025C	YBR093C	1

YDL106C	YAR015W	1
YDL106C	YNL220W	1
YDL106C	YLR359W	1
YDL106C	YMR120C	1
YDL106C	YOR128C	1
YDL106C	YGR204W	1
YDL106C	YMR300C	1
YDL106C	YGL234W	1
YDL106C	YGR061C	1
YDL106C	YDR408C	1
YDL106C	YJR048W	1
YDL106C	YAR050W	2
YDL106C	YKR102W	2
YDL106C	YCL030C	1
YDL106C	YBR248C	1
YDL106C	YDL227C	1
YDL106C	YNL104C	1
YDL106C	YAR071W	1
YDL106C	YHR215W	1
YDL106C	YBR093C	1
YDL106C	YDR481C	1
YDL106C	YGR233C	1
YDL106C	YDR354W	1
YDL106C	YKL216W	1
YDL106C	YEL021W	1
YNL097C	YBR093C	2
YFR034C	YKL001C	1
YFR034C	YAR071W	1
YFR034C	YHR215W	1
YFR034C	YBR093C	1
YFR034C	YDR481C	1
YFR034C	YGR233C	1
YFR034C	YML123C	1
YFR034C	YPL031C	1
YFR034C	YLR142W	2
YFR034C	YHR037W	2
YFR034C	YHR136C	1
YOR363C	YML042W	1
YOR363C	YNR001C	1
YOR363C	YOR100C	1
YOR363C	YDR256C	1
YOR363C	YOR180C	1
YOR363C	YLR284C	1
YOR363C	YER015W	1
YOR363C	YKR009C	1
YOR363C	YNL009W	1
YOR363C	YDL078C	1
YOR363C	YOL147C	1
YOR363C	YDR244W	1
YOR363C	YNL329C	1
YOR363C	YIL160C	1
YOR363C	YGL205W	1
YOR363C	YKL188C	1
YOR363C	YNL202W	1
YOR363C	YJR019C	1
YOR363C	YBR159W	1
YOR363C	YJL218W	1
YOR363C	YOL002C	1
YOR363C	YPL095C	1
YNR052C	YOL086C	2
YNR052C	YCR012W	1
YLR014C	YKL216W	1
YLR014C	YMR271C	1
YLR014C	YEL021W	1
YLR014C	YLR420W	1
YKL015W	YLR142W	1
YKL015W	YHR037W	1
YNL216W	YBR085W	2
YNL216W	YOL086C	1
YNL216W	YPL111W	1
YNL216W	YAL038W	1
YNL216W	YGR254W	1
YNL216W	YHR174W	1
YNL216W	YDL022W	1
YNL216W	YCL030C	1
YNL216W	YCL066W	1
YNL216W	YCL067C	1
YNL216W	YCR012W	1
YNL216W	YGL008C	1
YNL216W	YBR049C	1
YNL216W	YBL027W	2
YNL216W	YDR382W	2
YNL216W	YDR064W	1
YNL216W	YPL090C	1
YNL216W	YNL309W	1
YGL071W	YNL259C	1
YGL071W	YDR270W	1
YGL071W	YMR058W	1
YGL071W	YDR534C	1
YGL071W	YOR382W	1
YGL071W	YOR383C	1
YGL071W	YLR214W	1
YGL071W	YKL220C	1
YGL071W	YBR207W	1
YGL071W	YER145C	1
YGL071W	YLR136C	1
YBR049C	YFL039C	1
YBR049C	YAL038W	1
YBR049C	YDL164C	1
YBR049C	YGR254W	1
YBR049C	YKL182W	1
YBR049C	YPL231W	1
YBR049C	YBR020W	1
YBR049C	YBR019C	1
YBR049C	YMR186W	1
YBR049C	YER086W	1
YBR049C	YCR012W	1
YBR049C	YNL216W	1
YBR049C	YDL140C	1
YBR049C	YOL004W	1
YBR049C	YDR146C	1
YBR049C	YOL006C	1

```
YBR049C   YDR050C   1
YBR049C   YGL026C   1
YLR176C   YLR176C   2
YKL038W   YHR094C   2
YKL038W   YMR011W   2
YKL038W   YDR345C   2
YKL038W   YHR092C   2
YKL038W   YGL062W   1
YHL027W   YDR403W   1
YHL027W   YDR402C   1
YHL027W   YIL072W   1
YHL027W   YJR094C   1
YHL027W   YJL106W   1
YGR044C   YCR097W   1
YGR044C   YLR131C   1
YGR044C   YPL256C   1
YGR044C   YCL067C   1
YGR044C   YJR094C   2
YGR044C   YOL004W   1
YGR044C   YDR146C   1
YGR044C   YCR084C   1
YGR044C   YDR207C   1
YPR065W   YBR085W   2
YPR065W   YJR047C   2
YPR065W   YBL045C   2
YPR065W   YIL111W   2
YPR065W   YDR155C   2
YPR065W   YEL039C   2
YPR065W   YHR007C   2
YPR065W   YJR040W   2
YPR065W   YLR256W   2
YPR065W   YDR044W   2
YPR065W   YLR450W   2
YPR065W   YHR042W   2
YPR065W   YPR072W   2
YPR065W   YGL055W   2
YPR065W   YPR065W   2
YPR065W   YGL162W   2
YPR065W   YCR084C   2
YBL093C   YBR020W   1
YER169W   YOR386W   2
YDL020C   YOL038W   1
YDL020C   YHR027C   1
YOL067C   YLR304C   1
YOL067C   YNR001C   1
YOL067C   YCR005C   1
YOL067C   YEL071W   1
YOL067C   YNL037C   1
YOL067C   YOR136W   1
YGL252C   YLR304C   1
YGL252C   YNR001C   1
YGL252C   YCR005C   1
YGL252C   YOR136W   1
YBL103C   YLR304C   1
YBL103C   YDR448W   1
YBL103C   YNR001C   1
YBL103C   YCR005C   1
YBL103C   YNL037C   1
YBL103C   YOR136W   1
YBL103C   YDR176W   1
YIL084C   YJL106W   2
YIL084C   YJL153C   2
YIL084C   YKL209C   1
YHR119W   YJL071W   2
YHR119W   YAL020C   2
YHR119W   YAL021C   2
YHR119W   YHR164C   2
YHR119W   YHR043C   2
YHR119W   YJL221C   2
YHR119W   YJL165C   2
YHR119W   YMR290C   2
YHR119W   YDR044W   2
YHR119W   YML075C   2
YHR119W   YAL029C   2
YHR119W   YNL289W   2
YHR119W   YKL019W   2
YHR119W   YNL290W   2
YHR119W   YAL030W   2
YHR119W   YHR163W   2
YHR119W   YJL127C   2
YHR119W   YDR463W   2
YHR119W   YPL016W   2
YHR119W   YJL164C   2
YHR119W   YGR094W   2
YHR119W   YBR061C   2
YOR140W   YIL162W   2
YOL004W   YMR303C   1
YOL004W   YIL015W   1
YOL004W   YCL027W   2
YOL004W   YDL227C   1
YOL004W   YIL072W   2
YOL004W   YJL106W   2
YOL004W   YJL153C   2
YOL004W   YIR019C   1
YOL004W   YBR093C   1
YOL004W   YBR049C   2
YOL004W   YGR044C   1
YOL004W   YHL022C   1
YOL004W   YHR014W   2
YOL004W   YFL026W   2
YOL004W   YKL178C   2
YOL004W   YKL209C   2
YOL004W   YPL016W   1
YOL004W   YDR207C   2
YJL089W   YOL126C   1
YHR206W   YLR109W   1
YHR206W   YKR066C   1
YHR206W   YGR088W   1
YHR206W   YAL012W   2
YHR206W   YML070W   1
YHR206W   YLL001W   1
YHR206W   YOL151W   2
```

YHR206W	YDR258C	1
YHR206W	YPL240C	1
YHR206W	YDL182W	1
YHR206W	YMR105C	2
YHR206W	YJR104C	1
YHR206W	YLR354C	1
YHR206W	YDR353W	1
YHR206W	YGR209C	1
YHR206W	YML028W	1
YHR206W	YBR025C	1
YHR206W	YDR453C	1
YHR206W	YMR318C	2
YHR206W	YNL134C	2
YHR206W	YNL241C	1
YNL167C	YPR005C	2
YNL167C	YOR202W	2
YNL167C	YIL162W	2
YOR290C	YOL086C	1
YOR290C	YMR303C	1
YOR290C	YER026C	1
YOR290C	YGR157W	1
YOR290C	YLR133W	1
YOR290C	YLR286C	1
YOR290C	YBR020W	1
YOR290C	YBR019C	1
YOR290C	YJL153C	1
YOR290C	YJR073C	1
YOR290C	YAR071W	1
YOR290C	YBR093C	1
YOR290C	YEL060C	2
YOR290C	YIL162W	1
YBR289W	YIL015W	1
YBR289W	YDL227C	1
YBR289W	YPL187W	1
YBR289W	YAR071W	1
YBR289W	YBR093C	1
YBR289W	YEL060C	2
YBR289W	YIL162W	1
YHL025W	YOL086C	1
YHL025W	YMR303C	1
YHL025W	YIL015W	1
YHL025W	YBR020W	1
YHL025W	YBR019C	1
YHL025W	YDL227C	1
YHL025W	YJL153C	1
YHL025W	YAR071W	1
YHL025W	YBR093C	1
YHL025W	YIL162W	1
YGL207W	YFL039C	1
YGL207W	YMR199W	1
YGL207W	YPL256C	1
YGL207W	YAL040C	1
YGL207W	YDL227C	1
YGL207W	YDR225W	1
YGL207W	YDR224C	1
YGL207W	YCL018W	1
YGL207W	YDR143C	1
YGL207W	YGL207W	1
YGL207W	YER111C	1
YGL207W	YLR182W	1
YER161C	YJL153C	2
YMR179W	YMR303C	1
YMR179W	YBR009C	1
YMR179W	YNL030W	1
YMR179W	YDR225W	1
YMR179W	YBL002W	1
YMR179W	YBR093C	1
YDR392W	YCL030C	1
YGR063C	YDR225W	1
YGR063C	YDR224C	1
YML010W	YBL008W	2
YML010W	YDR225W	1
YML010W	YDR224C	1
YGR116W	YMR303C	2
YGR116W	YAL021C	2
YGR116W	YDR225W	1
YGR116W	YDR224C	1
YDR308C	YFL039C	1
YDR308C	YBR020W	1
YDR308C	YJL052W	1
YHR084W	YDR085C	1
YHR084W	YNR044W	1
YHR084W	YIL015W	1
YHR084W	YNL166C	1
YHR084W	YLR438W	1
YHR084W	YNL192W	1
YHR084W	YMR198W	1
YHR084W	YMR199W	1
YHR084W	YNL051W	1
YHR084W	YMR173W	1
YHR084W	YDL160C	1
YHR084W	YNL280C	1
YHR084W	YJL157C	1
YHR084W	YBR040W	1
YHR084W	YCR089W	1
YHR084W	YCL027W	1
YHR084W	YBL016W	1
YHR084W	YIR013C	1
YHR084W	YDR309C	1
YHR084W	YCL067C	1
YHR084W	YBL002W	1
YHR084W	YKL189W	1
YHR084W	YER019W	1
YHR084W	YPR141C	1
YHR084W	YMR065W	1
YHR084W	YGL089C	1
YHR084W	YDR461W	1
YHR084W	YNL145W	1
YHR084W	YPL187W	1
YHR084W	YLR332W	1
YHR084W	YGL178W	1
YHR084W	YIR019C	1

YHR084W	YDL127W	1
YHR084W	YBL017C	1
YHR084W	YKL127W	1
YHR084W	YJR153W	1
YHR084W	YGR233C	1
YHR084W	YNL279W	1
YHR084W	YIL037C	1
YHR084W	YPL192C	1
YHR084W	YIL117C	1
YHR084W	YDR055W	1
YHR084W	YGR213C	1
YHR084W	YOR077W	1
YHR084W	YBR070C	1
YHR084W	YHR205W	1
YHR084W	YLR403W	1
YHR084W	YER018C	1
YHR084W	YHR152W	1
YHR084W	YCR018C	1
YHR084W	YOR247W	1
YHR084W	YER103W	1
YHR084W	YLR452C	1
YHR084W	YHR084W	1
YHR084W	YFL026W	1
YHR084W	YKL178C	1
YHR084W	YKL209C	1
YHR084W	YBR083W	1
YHR084W	YBR117C	1
YHR084W	YOR248W	1
YHR084W	YML100W	1
YHR084W	YCR084C	1
YHR084W	YNL283C	1
YHR084W	YDL222C	1
YHR084W	YEL033W	1
YHR084W	YGR149W	1
YHR084W	YHL021C	1
YHR084W	YHR156C	1
YHR084W	YIL083C	1
YHR084W	YIL169C	1
YHR084W	YJL017W	1
YHR084W	YJL142C	1
YHR084W	YLR042C	1
YHR084W	YLR414C	1
YHR084W	YNL159C	1
YHR084W	YOL155C	1
YHR084W	YOR129C	1
YHR084W	YOR343C	1
YHR084W	YPL114W	1
YHR084W	YLR120C	1
YDR463W	YBR068C	1
YDR310C	YPR054W	2
YDR310C	YGR059W	2
YPL016W	YOL086C	1
YPL016W	YMR303C	1
YPL016W	YBR020W	1
YPL016W	YBR019C	1
YPL016W	YJL153C	1
YPL016W	YIR019C	1
YPL016W	YHR119W	1
YPL016W	YOL004W	1
YPL016W	YKL209C	1
YPL016W	YIL162W	1
YJL176C	YOL086C	1
YJL176C	YMR303C	1
YJL176C	YBR020W	1
YJL176C	YBR019C	1
YJL176C	YDL227C	1
YJL176C	YJL153C	1
YJL176C	YIL162W	1
YER111C	YCR002C	1
YER111C	YJR076C	1
YER111C	YMR199W	1
YER111C	YPL256C	1
YER111C	YLR286C	1
YER111C	YMR043W	1
YER111C	YNL289W	1
YER111C	YGL207W	1
YDR146C	YKL185W	1
YDR146C	YJL194W	1
YDR146C	YLR286C	1
YDR146C	YNL327W	1
YDR146C	YDL227C	1
YDR146C	YMR043W	1
YDR146C	YGL035C	1
YDR146C	YGL209W	1
YDR146C	YDL127W	1
YDR146C	YDL179W	1
YDR146C	YBR049C	1
YDR146C	YGR044C	1
YDR146C	YLR079W	1
YLR182W	YOR074C	1
YLR182W	YJR057W	1
YLR182W	YDL164C	1
YLR182W	YPR120C	1
YLR182W	YGR109C	1
YLR182W	YMR199W	1
YLR182W	YPL256C	1
YLR182W	YLR286C	1
YLR182W	YNL289W	1
YLR182W	YDL127W	1
YLR182W	YNL102W	1
YLR182W	YKL045W	1
YLR182W	YER070W	1
YLR182W	YGL207W	1
YBR198C	YMR303C	1
YBR083W	YNL166C	1
YBR083W	YLR438W	1
YBR083W	YMR199W	1
YBR083W	YNL051W	1
YBR083W	YMR173W	1
YBR083W	YDL160C	1
YBR083W	YDL161W	1
YBR083W	YIR013C	1

YBR083W	YBL002W	1
YBR083W	YOR099W	1
YBR083W	YKR061W	1
YBR083W	YLR332W	1
YBR083W	YGL178W	1
YBR083W	YIR019C	1
YBR083W	YJR153W	1
YBR083W	YIL117C	1
YBR083W	YDR055W	1
YBR083W	YER070W	1
YBR083W	YGR213C	1
YBR083W	YOR077W	1
YBR083W	YBR070C	1
YBR083W	YLR403W	1
YBR083W	YHR152W	1
YBR083W	YCR018C	1
YBR083W	YOR247W	1
YBR083W	YER103W	1
YBR083W	YHR084W	1
YBR083W	YGL162W	1
YBR083W	YBR117C	1
YBR083W	YOR248W	1
YBR083W	YML100W	1
YBR083W	YNL283C	1
YBR083W	YDL222C	1
YBR083W	YEL033W	1
YBR083W	YGR149W	1
YBR083W	YHL021C	1
YBR083W	YHR156C	1
YBR083W	YJL017W	1
YBR083W	YJL142C	1
YBR083W	YLR042C	1
YBR083W	YLR414C	1
YBR083W	YNL159C	1
YBR083W	YOR225W	1
YBR083W	YPL114W	1
YBR083W	YLR120C	1
YBR240C	YCR020C	1
YBR240C	YBR092C	1
YBR240C	YOL055C	1
YBR240C	YPL258C	1
YBR240C	YPR121W	1
YBR240C	YPL214C	1
YBR240C	YOR143C	1
YBR240C	YLR004C	1
YCR084C	YJR047C	2
YCR084C	YIL015W	2
YCR084C	YJR048W	2
YCR084C	YEL039C	2
YCR084C	YDR403W	2
YCR084C	YDR402C	2
YCR084C	YDR040C	2
YCR084C	YBR020W	2
YCR084C	YBR019C	2
YCR084C	YPR005C	2
YCR084C	YCL066W	2
YCR084C	YDL227C	2
YCR084C	YJR094C	2
YCR084C	YGL089C	2
YCR084C	YPL187W	2
YCR084C	YIR019C	2
YCR084C	YCR104W	2
YCR084C	YLR461W	2
YCR084C	YFL020C	2
YCR084C	YGR044C	2
YCR084C	YER070W	2
YCR084C	YIL066C	2
YCR084C	YPR065W	2
YCR084C	YLR452C	2
YCR084C	YHR084W	2
YCR084C	YFL026W	2
YCR084C	YOR212W	2
YCR084C	YDR103W	2
YCR084C	YKL209C	2
YCR084C	YIL162W	2
YCR084C	YDR172W	2
YOR344C	YOL086C	1
YOR344C	YHR174W	1
YDL170W	YGR019W	1
YDL170W	YBR006W	1
YDL170W	YDL210W	1
YDR207C	YAL054C	2
YDR207C	YIL015W	2
YDR207C	YPL111W	2
YDR207C	YLR438W	1
YDR207C	YAL038W	2
YDR207C	YER026C	1
YDR207C	YGR157W	1
YDR207C	YDR256C	2
YDR207C	YDR403W	2
YDR207C	YER179W	2
YDR207C	YKR009C	2
YDR207C	YBR020W	2
YDR207C	YIL072W	2
YDR207C	YGL073W	2
YDR207C	YPL240C	2
YDR207C	YMR108W	2
YDR207C	YJR094C	2
YDR207C	YJL106W	2
YDR207C	YJL153C	2
YDR207C	YDR123C	1
YDR207C	YOR351C	2
YDR207C	YNL210W	2
YDR207C	YJR073C	1
YDR207C	YKL197C	2
YDR207C	YOR386W	1
YDR207C	YIL160C	2
YDR207C	YGL205W	2
YDR207C	YGR258C	1
YDR207C	YPL153C	1
YDR207C	YJR052W	1
YDR207C	YLR329W	2

YDR207C	YMR133W	2
YDR207C	YLR263W	2
YDR207C	YHL024W	2
YDR207C	YGR044C	2
YDR207C	YOL004W	2
YDR207C	YGL213C	2
YDR207C	YNL196C	1
YDR207C	YHL022C	2
YDR207C	YHR152W	2
YDR207C	YHR014W	2
YDR207C	YHR153C	2
YDR207C	YDR522C	2
YDR207C	YOL006C	2
YDR207C	YER044CA	2
YDR207C	YDR285W	2
YOR230W	YJL106W	2
YIL101C	YPR119W	2
YIL101C	YMR199W	2
YIL101C	YAL040C	2
YIL101C	YAL012W	2
YIL101C	YHR050W	2
YML007W	YLR109W	1
YML007W	YKR066C	1
YML007W	YDL126C	1
YML007W	YGR088W	1
YML007W	YAL012W	1
YML007W	YML070W	1
YML007W	YBR008C	1
YML007W	YPL091W	1
YML007W	YBR244W	1
YML007W	YOL151W	1
YML007W	YJL101C	1
YML007W	YOR202W	1
YML007W	YFL014W	1
YML007W	YDR258C	1
YML007W	YPL240C	1
YML007W	YDL182W	1
YML007W	YPL171C	1
YML007W	YMR105C	1
YML007W	YDR032C	1
YML007W	YOR208W	1
YML007W	YFR004W	1
YML007W	YKL145W	1
YML007W	YJR104C	1
YML007W	YHR008C	1
YML007W	YAL005C	1
YML007W	YLR354C	1
YML007W	YDR074W	1
YML007W	YDR353W	1
YML007W	YLR043C	1
YML007W	YGR209C	1
YML007W	YML028W	1
YML007W	YKL210W	1
YML007W	YDR135C	1
YML007W	YDR453C	1
YML007W	YMR318C	1
YML007W	YNL134C	1
YML007W	YNL274C	1
YML007W	YOL052CA	1
YML007W	YNL241C	1
YDR259C	YDR040C	1
YOR162C	YDR011W	1
YOR162C	YGR281W	1
YOR162C	YOR162C	1
YJL056C	YGL256W	1
YJL056C	YDR284C	1
YJL056C	YMR319C	1
YJL056C	YKL165C	1
YJL056C	YOL002C	1
YJL056C	YOL154W	1
YJL056C	YJL056C	1
YJL056C	YMR243C	1
YJL056C	YNR039C	1
YJL056C	YGL255W	1
YJL056C	YLR130C	1
YJL056C	YKL175W	1
