# Supplementary material for: Unraveling condition specific gene transcriptional regulatory networks in Saccharomyces cerevisiae
Source: BMC Bioinformatics. 2006 Mar 21;7:165. doi: 10.1186/1471-2105-7-165 (PMC1488875; doi:10.1186/1471-2105-7-165)
Supplement: Additional File 6 — Predicted transcriptional regulatory links obtained by applying the LINK model to Alon's network. Each link is accompanied by a list of experiments in which it is likely to be functional. [file 1471-2105-7-165-S6.pdf]

Predicted transcriptional regulatory links obtained by applying the LINK model to Alon's network (Milo, R. et al. Science 298, 824-7, 2002).

Each link in this file (e.g. ABF1 -*-> HHO1) is followed by a list of the predicted experimental conditions in which it is likely to be active.

ABF1 -*-> HHO1

(c) 1. Cell cycle: Expression in response to Cln3p (set 1)(1)
(c) 2. Cell Cycle: Expression in response to Cln3p (set 2)(1)
(c) 5. Expression during the cell cycle (alpha factor arrest and release)(6)
(c) 5. Expression during the cell cycle (alpha factor arrest and release)(7)
(c) 5. Expression during the cell cycle (alpha factor arrest and release)(16)
(c) 6. Expression during the cell cycle (cdc15 arrest and release)(16)
(c) 6. Expression during the cell cycle (cdc15 arrest and release)(18)
(c) 8. Expression during the cell cycle (cell size selection and release)(8)
(c) 8. Expression during the cell cycle (cell size selection and release)(9)
(c) 8. Expression during the cell cycle (cell size selection and release)(10)
(c) 11. Expression during diauxic shift: 9h,11h,13h,15h,17h,19h,21h(2)
(c) 26. Fink: Expression in diploid high copy TEC1(1)
(c) 487. Expression in response to sorbitol: 15 30 45 90 120 min(4)
(c) 487. Expression in response to sorbitol: 15 30 45 90 120 min(5)
(c) 611. Brown enviromental changes :YPD stationary phase 2 h ypd-1(1)
(c) DES460 (wt) - mock irradiation - 30 min
(c) DES460 (wt) - mock irradiation - 60 min
(c) 100 microM BCS 30 min
(c) 100 microM BCS 60 min
(c) MAC1-up (C)
(c) wt-gal

ACE2 -*-> CST13

(c) 4. Cell Cycle: Expression in response to Clb2p (set 2, 30 min)(1)
(c) 5. Expression during the cell cycle (alpha factor arrest and release)(13)
(c) 5. Expression during the cell cycle (alpha factor arrest and release)(15)
(c) 5. Expression during the cell cycle (alpha factor arrest and release)(16)
(c) 5. Expression during the cell cycle (alpha factor arrest and release)(17)
(c) 5. Expression during the cell cycle (alpha factor arrest and release)(18)
(c) 6. Expression during the cell cycle (cdc15 arrest and release)(22)
(c) 6. Expression during the cell cycle (cdc15 arrest and release)(23)
(c) 8. Expression during the cell cycle (cell size selection and release)(10)
(c) 8. Expression during the cell cycle (cell size selection and release)(11)
(c) 8. Expression during the cell cycle (cell size selection and release)(12)
(c) 8. Expression during the cell cycle (cell size selection and release)(13)
(c) 8. Expression during the cell cycle (cell size selection and release)(14)
(c) 26. Fink: Expression in diploid high copy TEC1(1)
(c) PHO81c vs WT exp1(1)
(c) 497. Brown enviromental changes :Heat Shock 000 minutes hs-2(1)
(c) wt_plus_gamma_90_min
(c) DES460 (wt) - mock irradiation - 30 min

ACE2 -*-> JIP1

(c) 4. Cell Cycle: Expression in response to Clb2p (set 2, 30 min)(1)
(c) 5. Expression during the cell cycle (alpha factor arrest and release)(13)
(c) 5. Expression during the cell cycle (alpha factor arrest and release)(15)
(c) 5. Expression during the cell cycle (alpha factor arrest and release)(16)
(c) 5. Expression during the cell cycle (alpha factor arrest and release)(17)
(c) 5. Expression during the cell cycle (alpha factor arrest and release)(18)
(c) 6. Expression during the cell cycle (cdc15 arrest and release)(22)
(c) 6. Expression during the cell cycle (cdc15 arrest and release)(23)
(c) 8. Expression during the cell cycle (cell size selection and release)(10)
(c) 8. Expression during the cell cycle (cell size selection and release)(11)
(c) 8. Expression during the cell cycle (cell size selection and release)(12)
(c) 8. Expression during the cell cycle (cell size selection and release)(13)
(c) 8. Expression during the cell cycle (cell size selection and release)(14)
(c) 26. Fink: Expression in diploid high copy TEC1(1)
(c) PHO81c vs WT exp1(1)
(c) 497. Brown enviromental changes :Heat Shock 000 minutes hs-2(1)
(c) wt_plus_gamma_90_min
(c) DES460 (wt) - mock irradiation - 30 min

ACE2 -*-> SCW11

(c) 4. Cell Cycle: Expression in response to Clb2p (set 2, 30 min)(1)
(c) 5. Expression during the cell cycle (alpha factor arrest and release)(13)
(c) 5. Expression during the cell cycle (alpha factor arrest and release)(15)
(c) 5. Expression during the cell cycle (alpha factor arrest and release)(16)
(c) 5. Expression during the cell cycle (alpha factor arrest and release)(17)
(c) 5. Expression during the cell cycle (alpha factor arrest and release)(18)
(c) 6. Expression during the cell cycle (cdc15 arrest and release)(22)
(c) 6. Expression during the cell cycle (cdc15 arrest and release)(23)
(c) 8. Expression during the cell cycle (cell size selection and release)(10)
(c) 8. Expression during the cell cycle (cell size selection and release)(11)
(c) 8. Expression during the cell cycle (cell size selection and release)(12)
(c) 8. Expression during the cell cycle (cell size selection and release)(13)
(c) 8. Expression during the cell cycle (cell size selection and release)(14)
(c) 26. Fink: Expression in diploid high copy TEC1(1)
(c) PHO81c vs WT exp1(1)
(c) 497. Brown enviromental changes :Heat Shock 000 minutes hs-2(1)
(c) wt_plus_gamma_90_min
(c) DES460 (wt) - mock irradiation - 30 min

ACE2 -*-> SUN4

(c) 4. Cell Cycle: Expression in response to Clb2p (set 2, 30 min)(1)
(c) 5. Expression during the cell cycle (alpha factor arrest and release)(13)
(c) 5. Expression during the cell cycle (alpha factor arrest and release)(15)
(c) 5. Expression during the cell cycle (alpha factor arrest and release)(16)
(c) 5. Expression during the cell cycle (alpha factor arrest and release)(17)
(c) 5. Expression during the cell cycle (alpha factor arrest and release)(18)
(c) 6. Expression during the cell cycle (cdc15 arrest and release)(22)
(c) 6. Expression during the cell cycle (cdc15 arrest and release)(23)
(c) 8. Expression during the cell cycle (cell size selection and release)(10)
(c) 8. Expression during the cell cycle (cell size selection and release)(11)

```
    (c) 8. Expression during the cell cycle (cell size selection and release)(12)
    (c) 8. Expression during the cell cycle (cell size selection and release)(13)
    (c) 8. Expression during the cell cycle (cell size selection and release)(14)
    (c) 26. Fink: Expression in diploid high copy TEC1(1)
    (c) PHO81c vs WT exp1(1)
    (c) 497. Brown enviromental changes :Heat Shock 000 minutes  hs-2(1)
    (c) wt_plus_gamma_90_min
    (c) DES460 (wt) - mock irradiation - 30 min


ACE2 -*-> YOR264W


    (c) 4. Cell Cycle: Expression in response to Clb2p (set 2, 30 min)(1)
    (c) 5. Expression during the cell cycle (alpha factor arrest and release)(13)
    (c) 5. Expression during the cell cycle (alpha factor arrest and release)(15)
    (c) 5. Expression during the cell cycle (alpha factor arrest and release)(16)
    (c) 5. Expression during the cell cycle (alpha factor arrest and release)(17)
    (c) 5. Expression during the cell cycle (alpha factor arrest and release)(18)
    (c) 6. Expression during the cell cycle (cdc15 arrest and release)(22)
    (c) 6. Expression during the cell cycle (cdc15 arrest and release)(23)
    (c) 8. Expression during the cell cycle (cell size selection and release)(10)
    (c) 8. Expression during the cell cycle (cell size selection and release)(11)
    (c) 8. Expression during the cell cycle (cell size selection and release)(12)
    (c) 8. Expression during the cell cycle (cell size selection and release)(13)
    (c) 8. Expression during the cell cycle (cell size selection and release)(14)
    (c) 26. Fink: Expression in diploid high copy TEC1(1)
    (c) PHO81c vs WT exp1(1)
    (c) 497. Brown enviromental changes :Heat Shock 000 minutes  hs-2(1)
    (c) wt_plus_gamma_90_min
    (c) DES460 (wt) - mock irradiation - 30 min


ASH1 -*-> CDC46


    (c) 5. Expression during the cell cycle (alpha factor arrest and release)(2)
    (c) 5. Expression during the cell cycle (alpha factor arrest and release)(10)
    (c) 5. Expression during the cell cycle (alpha factor arrest and release)(11)
    (c) 5. Expression during the cell cycle (alpha factor arrest and release)(12)
    (c) 6. Expression during the cell cycle (cdc15 arrest and release)(2)
    (c) 6. Expression during the cell cycle (cdc15 arrest and release)(9)
    (c) 6. Expression during the cell cycle (cdc15 arrest and release)(10)
    (c) 6. Expression during the cell cycle (cdc15 arrest and release)(20)
    (c) 6. Expression during the cell cycle (cdc15 arrest and release)(21)
    (c) 7. Expression during the cell Cycle (cdc28)(9)
    (c) 7. Expression during the cell Cycle (cdc28)(10)
    (c) 7. Expression during the cell Cycle (cdc28)(11)
    (c) 8. Expression during the cell cycle (cell size selection and release)(4)
    (c) 8. Expression during the cell cycle (cell size selection and release)(14)
    (c) 26. Fink: Expression in diploid high copy TEC1(1)
    (c) 576. Brown enviromental changes :Hypo-osmotic shock - 60 min(1)
    (c) 100 microM BCS 30 min
    (c) MAC1-up (C)


ASH1 -*-> CDC54


    (c) 5. Expression during the cell cycle (alpha factor arrest and release)(2)
    (c) 5. Expression during the cell cycle (alpha factor arrest and release)(10)
    (c) 5. Expression during the cell cycle (alpha factor arrest and release)(11)
    (c) 5. Expression during the cell cycle (alpha factor arrest and release)(12)
    (c) 6. Expression during the cell cycle (cdc15 arrest and release)(2)
    (c) 6. Expression during the cell cycle (cdc15 arrest and release)(9)
    (c) 6. Expression during the cell cycle (cdc15 arrest and release)(10)
    (c) 6. Expression during the cell cycle (cdc15 arrest and release)(20)
    (c) 6. Expression during the cell cycle (cdc15 arrest and release)(21)
    (c) 7. Expression during the cell Cycle (cdc28)(9)
    (c) 7. Expression during the cell Cycle (cdc28)(10)
    (c) 7. Expression during the cell Cycle (cdc28)(11)
    (c) 8. Expression during the cell cycle (cell size selection and release)(4)
    (c) 8. Expression during the cell cycle (cell size selection and release)(14)
    (c) 26. Fink: Expression in diploid high copy TEC1(1)
    (c) 576. Brown enviromental changes :Hypo-osmotic shock - 60 min(1)
    (c) 100 microM BCS 30 min
    (c) MAC1-up (C)


ASH1 -*-> CYK3


    (c) 5. Expression during the cell cycle (alpha factor arrest and release)(2)
    (c) 5. Expression during the cell cycle (alpha factor arrest and release)(10)
    (c) 5. Expression during the cell cycle (alpha factor arrest and release)(11)
    (c) 5. Expression during the cell cycle (alpha factor arrest and release)(12)
    (c) 6. Expression during the cell cycle (cdc15 arrest and release)(2)
    (c) 6. Expression during the cell cycle (cdc15 arrest and release)(9)
    (c) 6. Expression during the cell cycle (cdc15 arrest and release)(10)
    (c) 6. Expression during the cell cycle (cdc15 arrest and release)(20)
    (c) 6. Expression during the cell cycle (cdc15 arrest and release)(21)
    (c) 7. Expression during the cell Cycle (cdc28)(9)
    (c) 7. Expression during the cell Cycle (cdc28)(10)
    (c) 7. Expression during the cell Cycle (cdc28)(11)
    (c) 8. Expression during the cell cycle (cell size selection and release)(4)
    (c) 8. Expression during the cell cycle (cell size selection and release)(14)
    (c) 26. Fink: Expression in diploid high copy TEC1(1)
    (c) 576. Brown enviromental changes :Hypo-osmotic shock - 60 min(1)
    (c) 100 microM BCS 30 min
    (c) MAC1-up (C)


ASH1 -*-> HSP150


    (c) 5. Expression during the cell cycle (alpha factor arrest and release)(2)
    (c) 5. Expression during the cell cycle (alpha factor arrest and release)(10)
    (c) 5. Expression during the cell cycle (alpha factor arrest and release)(11)
    (c) 5. Expression during the cell cycle (alpha factor arrest and release)(12)
    (c) 6. Expression during the cell cycle (cdc15 arrest and release)(2)
    (c) 6. Expression during the cell cycle (cdc15 arrest and release)(9)
    (c) 6. Expression during the cell cycle (cdc15 arrest and release)(10)
```

(c) 6. Expression during the cell cycle (cdc15 arrest and release)(20)
(c) 6. Expression during the cell cycle (cdc15 arrest and release)(21)
(c) 7. Expression during the cell Cycle (cdc28)(9)
(c) 7. Expression during the cell Cycle (cdc28)(10)
(c) 7. Expression during the cell Cycle (cdc28)(11)
(c) 8. Expression during the cell cycle (cell size selection and release)(4)
(c) 8. Expression during the cell cycle (cell size selection and release)(14)
(c) 26. Fink: Expression in diploid high copy TEC1(1)
(c) 576. Brown enviromental changes :Hypo-osmotic shock - 60 min(1)
(c) 100 microM BCS 30 min
(c) MAC1-up (C)

ASH1 -*-> PCL9

(c) 5. Expression during the cell cycle (alpha factor arrest and release)(2)
(c) 5. Expression during the cell cycle (alpha factor arrest and release)(10)
(c) 5. Expression during the cell cycle (alpha factor arrest and release)(11)
(c) 5. Expression during the cell cycle (alpha factor arrest and release)(12)
(c) 6. Expression during the cell cycle (cdc15 arrest and release)(2)
(c) 6. Expression during the cell cycle (cdc15 arrest and release)(9)
(c) 6. Expression during the cell cycle (cdc15 arrest and release)(10)
(c) 6. Expression during the cell cycle (cdc15 arrest and release)(20)
(c) 6. Expression during the cell cycle (cdc15 arrest and release)(21)
(c) 7. Expression during the cell Cycle (cdc28)(9)
(c) 7. Expression during the cell Cycle (cdc28)(10)
(c) 7. Expression during the cell Cycle (cdc28)(11)
(c) 8. Expression during the cell cycle (cell size selection and release)(4)
(c) 8. Expression during the cell cycle (cell size selection and release)(14)
(c) 26. Fink: Expression in diploid high copy TEC1(1)
(c) 576. Brown enviromental changes :Hypo-osmotic shock - 60 min(1)
(c) 100 microM BCS 30 min
(c) MAC1-up (C)

ASH1 -*-> PIR1

(c) 5. Expression during the cell cycle (alpha factor arrest and release)(2)
(c) 5. Expression during the cell cycle (alpha factor arrest and release)(10)
(c) 5. Expression during the cell cycle (alpha factor arrest and release)(11)
(c) 5. Expression during the cell cycle (alpha factor arrest and release)(12)
(c) 6. Expression during the cell cycle (cdc15 arrest and release)(2)
(c) 6. Expression during the cell cycle (cdc15 arrest and release)(9)
(c) 6. Expression during the cell cycle (cdc15 arrest and release)(10)
(c) 6. Expression during the cell cycle (cdc15 arrest and release)(20)
(c) 6. Expression during the cell cycle (cdc15 arrest and release)(21)
(c) 7. Expression during the cell Cycle (cdc28)(9)
(c) 7. Expression during the cell Cycle (cdc28)(10)
(c) 7. Expression during the cell Cycle (cdc28)(11)
(c) 8. Expression during the cell cycle (cell size selection and release)(4)
(c) 8. Expression during the cell cycle (cell size selection and release)(14)
(c) 26. Fink: Expression in diploid high copy TEC1(1)
(c) 576. Brown enviromental changes :Hypo-osmotic shock - 60 min(1)
(c) 100 microM BCS 30 min
(c) MAC1-up (C)

ASH1 -*-> PIR3

(c) 5. Expression during the cell cycle (alpha factor arrest and release)(2)
(c) 5. Expression during the cell cycle (alpha factor arrest and release)(10)
(c) 5. Expression during the cell cycle (alpha factor arrest and release)(11)
(c) 5. Expression during the cell cycle (alpha factor arrest and release)(12)
(c) 6. Expression during the cell cycle (cdc15 arrest and release)(2)
(c) 6. Expression during the cell cycle (cdc15 arrest and release)(9)
(c) 6. Expression during the cell cycle (cdc15 arrest and release)(10)
(c) 6. Expression during the cell cycle (cdc15 arrest and release)(20)
(c) 6. Expression during the cell cycle (cdc15 arrest and release)(21)
(c) 7. Expression during the cell Cycle (cdc28)(9)
(c) 7. Expression during the cell Cycle (cdc28)(10)
(c) 7. Expression during the cell Cycle (cdc28)(11)
(c) 8. Expression during the cell cycle (cell size selection and release)(4)
(c) 8. Expression during the cell cycle (cell size selection and release)(14)
(c) 26. Fink: Expression in diploid high copy TEC1(1)
(c) 576. Brown enviromental changes :Hypo-osmotic shock - 60 min(1)
(c) 100 microM BCS 30 min
(c) MAC1-up (C)

ASH1 -*-> SIC1

(c) 5. Expression during the cell cycle (alpha factor arrest and release)(2)
(c) 5. Expression during the cell cycle (alpha factor arrest and release)(10)
(c) 5. Expression during the cell cycle (alpha factor arrest and release)(11)
(c) 5. Expression during the cell cycle (alpha factor arrest and release)(12)
(c) 6. Expression during the cell cycle (cdc15 arrest and release)(2)
(c) 6. Expression during the cell cycle (cdc15 arrest and release)(9)
(c) 6. Expression during the cell cycle (cdc15 arrest and release)(10)
(c) 6. Expression during the cell cycle (cdc15 arrest and release)(20)
(c) 6. Expression during the cell cycle (cdc15 arrest and release)(21)
(c) 7. Expression during the cell Cycle (cdc28)(9)
(c) 7. Expression during the cell Cycle (cdc28)(10)
(c) 7. Expression during the cell Cycle (cdc28)(11)
(c) 8. Expression during the cell cycle (cell size selection and release)(4)
(c) 8. Expression during the cell cycle (cell size selection and release)(14)
(c) 26. Fink: Expression in diploid high copy TEC1(1)
(c) 576. Brown enviromental changes :Hypo-osmotic shock - 60 min(1)
(c) 100 microM BCS 30 min
(c) MAC1-up (C)

BAS1 -*-> HIS7

(c) 8. Expression during the cell cycle (cell size selection and release)(10)
(c) 8. Expression during the cell cycle (cell size selection and release)(12)
(c) 11. Expression during diauxic shift: 9h,11h,13h,15h,17h,19h,21h(3)
(c) 48. Expression in response to 0.15,0.5,1.5,5,15.8,50,158,500 nM alpha-factor(6)

(c) 89. Expression in response to 3-aminotriazole(1)
(c) 95. Expression in response to 50ug/mL FK506(1)
(c) 387. Rosetta 2000: Expression in cells with ERG11 under tet promoter(1)
(c) 395. Rosetta 2000: Expression in response to 2-deoxy-D-glucose(1)
(c) 402. Rosetta 2000: Expression in response to Itraconazole(1)
(c) 407. Rosetta 2000: Expression in response to Tunicamycin(1)
(c) 481. Expression in response to heat shock: 15,30,45,60,120 min(2)
(c) 503. Brown enviromental changes :37C to 25C shock - 15 min(1)
(c) 572. Brown enviromental changes :Hypo-osmotic shock - 5 min(1)
(c) 579. Brown enviromental changes :aa starv 1 h(1)
(c) 580. Brown enviromental changes :aa starv 2 h(1)
(c) 581. Brown enviromental changes :aa starv 4 h(1)
(c) 585. Brown enviromental changes :Nitrogen Depletion 2 h(1)
(c) 586. Brown enviromental changes :Nitrogen Depletion 4 h(1)
(c) 595. Brown enviromental changes :diauxic shift timecourse(1)
(c) 613. Brown enviromental changes :YPD stationary phase 8 h ypd-1(1)
(c) 670. Expression in response to antimycin 60min(1)
(c) 684. Expression in response to 0.8M NaCl for 10 min in wild type(1)
(c) wt_plus_gamma_10_min

DAL80 -*-> YKR033C

(c) 6. Expression during the cell cycle (cdc15 arrest and release)(16)
(c) 6. Expression during the cell cycle (cdc15 arrest and release)(22)
(c) 6. Expression during the cell cycle (cdc15 arrest and release)(24)
(c) PHO81c vs WT exp1(1)
(c) 429. Expression in strain YHE711 (wild type) in response to 30 min 50 nM treatment with rapamycin in YPD(1)
(c) 430. Expression in strain PM38 (wild type), glucose versus ethanol: strain was shifted from medium containing dextrose as carbon source, ammonium sulfate as nirogen source, supplemented with leucine and uracil to same medium for 30 min, compared to a shift to a medium with synthetic ethanol instead of glucose for 30 min(1)
(c) 439. Expression in strain Jk9-3da (wild type) in response to 30 min 50 nM treatment with rapamycin in YPD(1)
(c) 442. Expression in strain PM38 (wild type) in response to 30 min 50 nM treatment with rapamycin in YPD(1)
(c) 499. Brown enviromental changes :Heat Shock 005 minutes hs-2(1)
(c) 504. Brown enviromental changes :37C to 25C shock - 30 min(1)
(c) 526. Brown enviromental changes :constant 0.32 mM H2O2 (40 min) rescan(1)
(c) 544. Brown enviromental changes :2.5mM DTT 030 min dtt-1(1)
(c) 546. Brown enviromental changes :2.5mM DTT 060 min dtt-1(1)
(c) 548. Brown enviromental changes :2.5mM DTT 120 min dtt-1(1)
(c) 581. Brown enviromental changes :aa starv 4 h(1)
(c) 582. Brown enviromental changes :aa starv 6 h(1)
(c) 585. Brown enviromental changes :Nitrogen Depletion 2 h(1)
(c) 586. Brown enviromental changes :Nitrogen Depletion 4 h(1)
(c) 587. Brown enviromental changes :Nitrogen Depletion 8 h(1)
(c) 588. Brown enviromental changes :Nitrogen Depletion 12 h(1)
(c) 589. Brown enviromental changes :Nitrogen Depletion 1 d(1)
(c) 590. Brown enviromental changes :Nitrogen Depletion 2 d(1)
(c) 591. Brown enviromental changes :Nitrogen Depletion 3 d(1)
(c) 607. Brown enviromental changes :YPD 1 d ypd-2(1)
(c) MAC1-up (B)

DAL80 -*-| TKL1

(c) 6. Expression during the cell cycle (cdc15 arrest and release)(16)
(c) 6. Expression during the cell cycle (cdc15 arrest and release)(23)
(c) 6. Expression during the cell cycle (cdc15 arrest and release)(24)
(c) 429. Expression in strain YHE711 (wild type) in response to 30 min 50 nM treatment with rapamycin in YPD(1)
(c) 430. Expression in strain PM38 (wild type), glucose versus ethanol: strain was shifted from medium containing dextrose as carbon source, ammonium sulfate as nirogen source, supplemented with leucine and uracil to same medium for 30 min, compared to a shift to a medium with synthetic ethanol instead of glucose for 30 min(1)
(c) 446. Expression in response to 0.1% MMS for 10 min(1)
(c) 447. Expression in response to 0.1% MMS for 30 min(1)
(c) 495. Brown enviromental changes :Heat Shock 80 minutes hs-1(1)
(c) 504. Brown enviromental changes :37C to 25C shock - 30 min(1)
(c) 569. Brown enviromental changes :1M sorbitol - 60 min(1)
(c) 587. Brown enviromental changes :Nitrogen Depletion 8 h(1)
(c) 588. Brown enviromental changes :Nitrogen Depletion 12 h(1)
(c) 589. Brown enviromental changes :Nitrogen Depletion 1 d(1)
(c) 590. Brown enviromental changes :Nitrogen Depletion 2 d(1)
(c) 591. Brown enviromental changes :Nitrogen Depletion 3 d(1)
(c) 607. Brown enviromental changes :YPD 1 d ypd-2(1)
(c) 610. Brown enviromental changes :YPD 5 d ypd-2(1)
(c) wt_plus_gamma_45_min
(c) MAC1-up (B)
(c) MAC1-up (C)

DAL80 -*-> SUL1

(c) 5. Expression during the cell cycle (alpha factor arrest and release)(14)
(c) 5. Expression during the cell cycle (alpha factor arrest and release)(16)
(c) 6. Expression during the cell cycle (cdc15 arrest and release)(2)
(c) 7. Expression during the cell Cycle (cdc28)(3)
(c) 7. Expression during the cell Cycle (cdc28)(4)
(c) PHO4c vs WT(1)
(c) PHO81c vs WT exp1(1)
(c) 481. Expression in response to heat shock: 15,30,45,60,120 min(4)
(c) 504. Brown enviromental changes :37C to 25C shock - 30 min(1)
(c) 513. Brown enviromental changes :29C to 33C - 5 minutes(1)
(c) 579. Brown enviromental changes :aa starv 1 h(1)
(c) 580. Brown enviromental changes :aa starv 2 h(1)
(c) 581. Brown enviromental changes :aa starv 4 h(1)
(c) 582. Brown enviromental changes :aa starv 6 h(1)
(c) 583. Brown enviromental changes :Nitrogen Depletion 30 min.(1)
(c) 584. Brown enviromental changes :Nitrogen Depletion 1 h(1)
(c) 585. Brown enviromental changes :Nitrogen Depletion 2 h(1)
(c) 586. Brown enviromental changes :Nitrogen Depletion 4 h(1)
(c) 588. Brown enviromental changes :Nitrogen Depletion 12 h(1)
(c) 589. Brown enviromental changes :Nitrogen Depletion 1 d(1)
(c) 590. Brown enviromental changes :Nitrogen Depletion 2 d(1)
(c) 591. Brown enviromental changes :Nitrogen Depletion 3 d(1)
(c) MAC1-up (B)

DAL80 -*-> YEL072W

(c) 5. Expression during the cell cycle (alpha factor arrest and release)(14)
(c) 5. Expression during the cell cycle (alpha factor arrest and release)(16)
(c) 6. Expression during the cell cycle (cdc15 arrest and release)(2)

```
    (c) 7. Expression during the cell Cycle (cdc28)(3)
    (c) 7. Expression during the cell Cycle (cdc28)(4)
    (c) PHO4c vs WT(1)
    (c) PHO81c vs WT exp1(1)
    (c) 481. Expression in response to heat shock: 15,30,45,60,120 min(4)
    (c) 504. Brown enviromental changes :37C to 25C shock - 30 min(1)
    (c) 513. Brown enviromental changes :29C to 33C - 5 minutes(1)
    (c) 579. Brown enviromental changes :aa starv 1 h(1)
    (c) 580. Brown enviromental changes :aa starv 2 h(1)
    (c) 581. Brown enviromental changes :aa starv 4 h(1)
    (c) 582. Brown enviromental changes :aa starv 6 h(1)
    (c) 583. Brown enviromental changes :Nitrogen Depletion 30 min.(1)
    (c) 584. Brown enviromental changes :Nitrogen Depletion 1 h(1)
    (c) 585. Brown enviromental changes :Nitrogen Depletion 2 h(1)
    (c) 586. Brown enviromental changes :Nitrogen Depletion 4 h(1)
    (c) 588. Brown enviromental changes :Nitrogen Depletion 12 h(1)
    (c) 589. Brown enviromental changes :Nitrogen Depletion 1 d(1)
    (c) 590. Brown enviromental changes :Nitrogen Depletion 2 d(1)
    (c) 591. Brown enviromental changes :Nitrogen Depletion 3 d(1)
    (c) MAC1-up (B)


DAL80 -*-> YLR053C


    (c) 5. Expression during the cell cycle (alpha factor arrest and release)(14)
    (c) 5. Expression during the cell cycle (alpha factor arrest and release)(16)
    (c) 6. Expression during the cell cycle (cdc15 arrest and release)(2)
    (c) 7. Expression during the cell Cycle (cdc28)(3)
    (c) 7. Expression during the cell Cycle (cdc28)(4)
    (c) PHO4c vs WT(1)
    (c) PHO81c vs WT exp1(1)
    (c) 481. Expression in response to heat shock: 15,30,45,60,120 min(4)
    (c) 504. Brown enviromental changes :37C to 25C shock - 30 min(1)
    (c) 513. Brown enviromental changes :29C to 33C - 5 minutes(1)
    (c) 579. Brown enviromental changes :aa starv 1 h(1)
    (c) 580. Brown enviromental changes :aa starv 2 h(1)
    (c) 581. Brown enviromental changes :aa starv 4 h(1)
    (c) 582. Brown enviromental changes :aa starv 6 h(1)
    (c) 583. Brown enviromental changes :Nitrogen Depletion 30 min.(1)
    (c) 584. Brown enviromental changes :Nitrogen Depletion 1 h(1)
    (c) 585. Brown enviromental changes :Nitrogen Depletion 2 h(1)
    (c) 586. Brown enviromental changes :Nitrogen Depletion 4 h(1)
    (c) 588. Brown enviromental changes :Nitrogen Depletion 12 h(1)
    (c) 589. Brown enviromental changes :Nitrogen Depletion 1 d(1)
    (c) 590. Brown enviromental changes :Nitrogen Depletion 2 d(1)
    (c) 591. Brown enviromental changes :Nitrogen Depletion 3 d(1)
    (c) MAC1-up (B)


DAL80 -*-| BAP2


    (c) 5. Expression during the cell cycle (alpha factor arrest and release)(14)
    (c) 5. Expression during the cell cycle (alpha factor arrest and release)(16)
    (c) 6. Expression during the cell cycle (cdc15 arrest and release)(2)
    (c) 7. Expression during the cell Cycle (cdc28)(3)
    (c) 7. Expression during the cell Cycle (cdc28)(4)
    (c) PHO4c vs WT(1)
    (c) PHO81c vs WT exp1(1)
    (c) 481. Expression in response to heat shock: 15,30,45,60,120 min(4)
    (c) 504. Brown enviromental changes :37C to 25C shock - 30 min(1)
    (c) 513. Brown enviromental changes :29C to 33C - 5 minutes(1)
    (c) 579. Brown enviromental changes :aa starv 1 h(1)
    (c) 580. Brown enviromental changes :aa starv 2 h(1)
    (c) 581. Brown enviromental changes :aa starv 4 h(1)
    (c) 582. Brown enviromental changes :aa starv 6 h(1)
    (c) 583. Brown enviromental changes :Nitrogen Depletion 30 min.(1)
    (c) 584. Brown enviromental changes :Nitrogen Depletion 1 h(1)
    (c) 585. Brown enviromental changes :Nitrogen Depletion 2 h(1)
    (c) 586. Brown enviromental changes :Nitrogen Depletion 4 h(1)
    (c) 588. Brown enviromental changes :Nitrogen Depletion 12 h(1)
    (c) 589. Brown enviromental changes :Nitrogen Depletion 1 d(1)
    (c) 590. Brown enviromental changes :Nitrogen Depletion 2 d(1)
    (c) 591. Brown enviromental changes :Nitrogen Depletion 3 d(1)
    (c) MAC1-up (B)


DAL80 -*-> ECM17


    (c) 6. Expression during the cell cycle (cdc15 arrest and release)(16)
    (c) 6. Expression during the cell cycle (cdc15 arrest and release)(22)
    (c) 6. Expression during the cell cycle (cdc15 arrest and release)(23)
    (c) 6. Expression during the cell cycle (cdc15 arrest and release)(24)
    (c) 7. Expression during the cell Cycle (cdc28)(6)
    (c) 8. Expression during the cell cycle (cell size selection and release)(1)
    (c) 54. Expression in response to overproduction of Ste5p(1)
    (c) 72. Expression in response to overproduction of activated Bni1p(1)
    (c) PHO81c vs WT exp1(1)
    (c) 504. Brown enviromental changes :37C to 25C shock - 30 min(1)
    (c) 547. Brown enviromental changes :2.5mM DTT 090 min dtt-1(1)
    (c) 579. Brown enviromental changes :aa starv 1 h(1)
    (c) 580. Brown enviromental changes :aa starv 2 h(1)
    (c) 581. Brown enviromental changes :aa starv 4 h(1)
    (c) 582. Brown enviromental changes :aa starv 6 h(1)
    (c) 583. Brown enviromental changes :Nitrogen Depletion 30 min.(1)
    (c) 584. Brown enviromental changes :Nitrogen Depletion 1 h(1)
    (c) 585. Brown enviromental changes :Nitrogen Depletion 2 h(1)
    (c) 586. Brown enviromental changes :Nitrogen Depletion 4 h(1)
    (c) 587. Brown enviromental changes :Nitrogen Depletion 8 h(1)
    (c) 588. Brown enviromental changes :Nitrogen Depletion 12 h(1)
    (c) 589. Brown enviromental changes :Nitrogen Depletion 1 d(1)
    (c) 590. Brown enviromental changes :Nitrogen Depletion 2 d(1)
    (c) 591. Brown enviromental changes :Nitrogen Depletion 3 d(1)


DAL80 -*-> GAP1


    (c) 6. Expression during the cell cycle (cdc15 arrest and release)(16)
    (c) 6. Expression during the cell cycle (cdc15 arrest and release)(22)
```

(c) 6. Expression during the cell cycle (cdc15 arrest and release)(23)
(c) 6. Expression during the cell cycle (cdc15 arrest and release)(24)
(c) 7. Expression during the cell Cycle (cdc28)(6)
(c) 8. Expression during the cell cycle (cell size selection and release)(1)
(c) 54. Expression in response to overproduction of Ste5p(1)
(c) 72. Expression in response to overproduction of activated Bni1p(1)
(c) PHO81c vs WT exp1(1)
(c) 504. Brown enviromental changes :37C to 25C shock - 30 min(1)
(c) 547. Brown enviromental changes :2.5mM DTT 090 min dtt-1(1)
(c) 579. Brown enviromental changes :aa starv 1 h(1)
(c) 580. Brown enviromental changes :aa starv 2 h(1)
(c) 581. Brown enviromental changes :aa starv 4 h(1)
(c) 582. Brown enviromental changes :aa starv 6 h(1)
(c) 583. Brown enviromental changes :Nitrogen Depletion 30 min.(1)
(c) 584. Brown enviromental changes :Nitrogen Depletion 1 h(1)
(c) 585. Brown enviromental changes :Nitrogen Depletion 2 h(1)
(c) 586. Brown enviromental changes :Nitrogen Depletion 4 h(1)
(c) 587. Brown enviromental changes :Nitrogen Depletion 8 h(1)
(c) 588. Brown enviromental changes :Nitrogen Depletion 12 h(1)
(c) 589. Brown enviromental changes :Nitrogen Depletion 1 d(1)
(c) 590. Brown enviromental changes :Nitrogen Depletion 2 d(1)
(c) 591. Brown enviromental changes :Nitrogen Depletion 3 d(1)

DAL80 -*-> YNL276C

(c) 6. Expression during the cell cycle (cdc15 arrest and release)(16)
(c) 6. Expression during the cell cycle (cdc15 arrest and release)(22)
(c) 6. Expression during the cell cycle (cdc15 arrest and release)(23)
(c) 6. Expression during the cell cycle (cdc15 arrest and release)(24)
(c) 7. Expression during the cell Cycle (cdc28)(6)
(c) 8. Expression during the cell cycle (cell size selection and release)(1)
(c) 54. Expression in response to overproduction of Ste5p(1)
(c) 72. Expression in response to overproduction of activated Bni1p(1)
(c) PHO81c vs WT exp1(1)
(c) 504. Brown enviromental changes :37C to 25C shock - 30 min(1)
(c) 547. Brown enviromental changes :2.5mM DTT 090 min dtt-1(1)
(c) 579. Brown enviromental changes :aa starv 1 h(1)
(c) 580. Brown enviromental changes :aa starv 2 h(1)
(c) 581. Brown enviromental changes :aa starv 4 h(1)
(c) 582. Brown enviromental changes :aa starv 6 h(1)
(c) 583. Brown enviromental changes :Nitrogen Depletion 30 min.(1)
(c) 584. Brown enviromental changes :Nitrogen Depletion 1 h(1)
(c) 585. Brown enviromental changes :Nitrogen Depletion 2 h(1)
(c) 586. Brown enviromental changes :Nitrogen Depletion 4 h(1)
(c) 587. Brown enviromental changes :Nitrogen Depletion 8 h(1)
(c) 588. Brown enviromental changes :Nitrogen Depletion 12 h(1)
(c) 589. Brown enviromental changes :Nitrogen Depletion 1 d(1)
(c) 590. Brown enviromental changes :Nitrogen Depletion 2 d(1)
(c) 591. Brown enviromental changes :Nitrogen Depletion 3 d(1)

DAL80 -*-| TAT1

(c) 6. Expression during the cell cycle (cdc15 arrest and release)(16)
(c) 6. Expression during the cell cycle (cdc15 arrest and release)(22)
(c) 6. Expression during the cell cycle (cdc15 arrest and release)(23)
(c) 6. Expression during the cell cycle (cdc15 arrest and release)(24)
(c) 7. Expression during the cell Cycle (cdc28)(6)
(c) 8. Expression during the cell cycle (cell size selection and release)(1)
(c) 54. Expression in response to overproduction of Ste5p(1)
(c) 72. Expression in response to overproduction of activated Bni1p(1)
(c) PHO81c vs WT exp1(1)
(c) 504. Brown enviromental changes :37C to 25C shock - 30 min(1)
(c) 547. Brown enviromental changes :2.5mM DTT 090 min dtt-1(1)
(c) 579. Brown enviromental changes :aa starv 1 h(1)
(c) 580. Brown enviromental changes :aa starv 2 h(1)
(c) 581. Brown enviromental changes :aa starv 4 h(1)
(c) 582. Brown enviromental changes :aa starv 6 h(1)
(c) 583. Brown enviromental changes :Nitrogen Depletion 30 min.(1)
(c) 584. Brown enviromental changes :Nitrogen Depletion 1 h(1)
(c) 585. Brown enviromental changes :Nitrogen Depletion 2 h(1)
(c) 586. Brown enviromental changes :Nitrogen Depletion 4 h(1)
(c) 587. Brown enviromental changes :Nitrogen Depletion 8 h(1)
(c) 588. Brown enviromental changes :Nitrogen Depletion 12 h(1)
(c) 589. Brown enviromental changes :Nitrogen Depletion 1 d(1)
(c) 590. Brown enviromental changes :Nitrogen Depletion 2 d(1)
(c) 591. Brown enviromental changes :Nitrogen Depletion 3 d(1)

DAL80 -*-| TAT2

(c) 6. Expression during the cell cycle (cdc15 arrest and release)(16)
(c) 6. Expression during the cell cycle (cdc15 arrest and release)(22)
(c) 6. Expression during the cell cycle (cdc15 arrest and release)(23)
(c) 6. Expression during the cell cycle (cdc15 arrest and release)(24)
(c) 7. Expression during the cell Cycle (cdc28)(6)
(c) 8. Expression during the cell cycle (cell size selection and release)(1)
(c) 54. Expression in response to overproduction of Ste5p(1)
(c) 72. Expression in response to overproduction of activated Bni1p(1)
(c) PHO81c vs WT exp1(1)
(c) 504. Brown enviromental changes :37C to 25C shock - 30 min(1)
(c) 547. Brown enviromental changes :2.5mM DTT 090 min dtt-1(1)
(c) 579. Brown enviromental changes :aa starv 1 h(1)
(c) 580. Brown enviromental changes :aa starv 2 h(1)
(c) 581. Brown enviromental changes :aa starv 4 h(1)
(c) 582. Brown enviromental changes :aa starv 6 h(1)
(c) 583. Brown enviromental changes :Nitrogen Depletion 30 min.(1)
(c) 584. Brown enviromental changes :Nitrogen Depletion 1 h(1)
(c) 585. Brown enviromental changes :Nitrogen Depletion 2 h(1)
(c) 586. Brown enviromental changes :Nitrogen Depletion 4 h(1)
(c) 587. Brown enviromental changes :Nitrogen Depletion 8 h(1)
(c) 588. Brown enviromental changes :Nitrogen Depletion 12 h(1)
(c) 589. Brown enviromental changes :Nitrogen Depletion 1 d(1)
(c) 590. Brown enviromental changes :Nitrogen Depletion 2 d(1)
(c) 591. Brown enviromental changes :Nitrogen Depletion 3 d(1)

DAL80 -*-> MEP2

(c) 6. Expression during the cell cycle (cdc15 arrest and release)(8)
(c) 6. Expression during the cell cycle (cdc15 arrest and release)(14)
(c) 6. Expression during the cell cycle (cdc15 arrest and release)(16)
(c) 7. Expression during the cell Cycle (cdc28)(14)
(c) 7. Expression during the cell Cycle (cdc28)(16)
(c) 7. Expression during the cell Cycle (cdc28)(17)
(c) 11. Expression during diauxic shift: 9h,11h,13h,15h,17h,19h,21h(1)
(c) 394. Rosetta 2000: Expression in cells with YEF3 under tet promoter(1)
(c) 395. Rosetta 2000: Expression in response to 2-deoxy-D-glucose(1)
(c) 397. Rosetta 2000: Expression in response to Cycloheximide white(1)
(c) 400. Rosetta 2000: Expression in response to Glucosamine(1)
(c) 428. Expression in strain PM38 (wild type) in response to 30 min 50 nM treatment with rapamycin in YPD(1)
(c) 429. Expression in strain YHE711 (wild type) in response to 30 min 50 nM treatment with rapamycin in YPD(1)
(c) 430. Expression in strain PM38 (wild type), glucose versus ethanol: strain was shifted from medium containing dextrose as carbon source, ammonium sulfate as nirogen source, supplemented with leucine and uracil to same medium for 30 min, compared to a shift to a medium with synthetic ethanol instead of glucose for 30 min(1)
(c) 496. Brown enviromental changes :Heat Shock 000 minutes hs-2(1)
(c) 498. Brown enviromental changes :Heat Shock 000 minutes hs-2(1)
(c) 504. Brown enviromental changes :37C to 25C shock - 30 min(1)
(c) 581. Brown enviromental changes :aa starv 4 h(1)
(c) 582. Brown enviromental changes :aa starv 6 h(1)
(c) 583. Brown enviromental changes :Nitrogen Depletion 30 min.(1)
(c) 584. Brown enviromental changes :Nitrogen Depletion 1 h(1)
(c) 585. Brown enviromental changes :Nitrogen Depletion 2 h(1)
(c) 586. Brown enviromental changes :Nitrogen Depletion 4 h(1)
(c) 587. Brown enviromental changes :Nitrogen Depletion 8 h(1)
(c) 588. Brown enviromental changes :Nitrogen Depletion 12 h(1)
(c) 590. Brown enviromental changes :Nitrogen Depletion 2 d(1)
(c) 593. Brown enviromental changes :Diauxic Shift Timecourse(1)
(c) MAC1-up (B)

GAT1 -*-> DAL2

(c) 428. Expression in strain PM38 (wild type) in response to 30 min 50 nM treatment with rapamycin in YPD(1)
(c) 429. Expression in strain YHE711 (wild type) in response to 30 min 50 nM treatment with rapamycin in YPD(1)
(c) 439. Expression in strain Jk9-3da (wild type) in response to 30 min 50 nM treatment with rapamycin in YPD(1)
(c) 442. Expression in strain PM38 (wild type) in response to 30 min 50 nM treatment with rapamycin in YPD(1)
(c) 477. Expression in response to trichostatin A (TSA): 15min,30min,60min,120min(1)
(c) 477. Expression in response to trichostatin A (TSA): 15min,30min,60min,120min(2)
(c) 477. Expression in response to trichostatin A (TSA): 15min,30min,60min,120min(3)
(c) 477. Expression in response to trichostatin A (TSA): 15min,30min,60min,120min(4)
(c) 479. Expression in diploid cells in response to rapamycin (100nM) for: 15min,30min,90min,120min(1)
(c) 479. Expression in diploid cells in response to rapamycin (100nM) for: 15min,30min,90min,120min(2)
(c) 479. Expression in diploid cells in response to rapamycin (100nM) for: 15min,30min,90min,120min(4)
(c) 578. Brown enviromental changes :aa starv 0.5 h(1)
(c) 579. Brown enviromental changes :aa starv 1 h(1)
(c) 580. Brown enviromental changes :aa starv 2 h(1)
(c) 581. Brown enviromental changes :aa starv 4 h(1)
(c) 582. Brown enviromental changes :aa starv 6 h(1)
(c) 583. Brown enviromental changes :Nitrogen Depletion 30 min.(1)
(c) 584. Brown enviromental changes :Nitrogen Depletion 1 h(1)
(c) 585. Brown enviromental changes :Nitrogen Depletion 2 h(1)
(c) 586. Brown enviromental changes :Nitrogen Depletion 4 h(1)
(c) 587. Brown enviromental changes :Nitrogen Depletion 8 h(1)
(c) 588. Brown enviromental changes :Nitrogen Depletion 12 h(1)
(c) 589. Brown enviromental changes :Nitrogen Depletion 1 d(1)
(c) 590. Brown enviromental changes :Nitrogen Depletion 2 d(1)
(c) 591. Brown enviromental changes :Nitrogen Depletion 3 d(1)
(c) 592. Brown enviromental changes :Nitrogen Depletion 5 d(1)
(c) 617. Brown enviromental changes :YPD stationary phase 3 d ypd-1(1)

GAT1 -*-> MEP1

(c) 428. Expression in strain PM38 (wild type) in response to 30 min 50 nM treatment with rapamycin in YPD(1)
(c) 429. Expression in strain YHE711 (wild type) in response to 30 min 50 nM treatment with rapamycin in YPD(1)
(c) 439. Expression in strain Jk9-3da (wild type) in response to 30 min 50 nM treatment with rapamycin in YPD(1)
(c) 442. Expression in strain PM38 (wild type) in response to 30 min 50 nM treatment with rapamycin in YPD(1)
(c) 477. Expression in response to trichostatin A (TSA): 15min,30min,60min,120min(1)
(c) 477. Expression in response to trichostatin A (TSA): 15min,30min,60min,120min(2)
(c) 477. Expression in response to trichostatin A (TSA): 15min,30min,60min,120min(3)
(c) 477. Expression in response to trichostatin A (TSA): 15min,30min,60min,120min(4)
(c) 479. Expression in diploid cells in response to rapamycin (100nM) for: 15min,30min,90min,120min(1)
(c) 479. Expression in diploid cells in response to rapamycin (100nM) for: 15min,30min,90min,120min(2)
(c) 479. Expression in diploid cells in response to rapamycin (100nM) for: 15min,30min,90min,120min(4)
(c) 578. Brown enviromental changes :aa starv 0.5 h(1)
(c) 579. Brown enviromental changes :aa starv 1 h(1)
(c) 580. Brown enviromental changes :aa starv 2 h(1)
(c) 581. Brown enviromental changes :aa starv 4 h(1)
(c) 582. Brown enviromental changes :aa starv 6 h(1)
(c) 583. Brown enviromental changes :Nitrogen Depletion 30 min.(1)
(c) 584. Brown enviromental changes :Nitrogen Depletion 1 h(1)
(c) 585. Brown enviromental changes :Nitrogen Depletion 2 h(1)
(c) 586. Brown enviromental changes :Nitrogen Depletion 4 h(1)
(c) 587. Brown enviromental changes :Nitrogen Depletion 8 h(1)
(c) 588. Brown enviromental changes :Nitrogen Depletion 12 h(1)
(c) 589. Brown enviromental changes :Nitrogen Depletion 1 d(1)
(c) 590. Brown enviromental changes :Nitrogen Depletion 2 d(1)
(c) 591. Brown enviromental changes :Nitrogen Depletion 3 d(1)
(c) 592. Brown enviromental changes :Nitrogen Depletion 5 d(1)
(c) 617. Brown enviromental changes :YPD stationary phase 3 d ypd-1(1)

GAT1 -*-> PUT1

(c) 428. Expression in strain PM38 (wild type) in response to 30 min 50 nM treatment with rapamycin in YPD(1)
(c) 429. Expression in strain YHE711 (wild type) in response to 30 min 50 nM treatment with rapamycin in YPD(1)
(c) 439. Expression in strain Jk9-3da (wild type) in response to 30 min 50 nM treatment with rapamycin in YPD(1)
(c) 442. Expression in strain PM38 (wild type) in response to 30 min 50 nM treatment with rapamycin in YPD(1)
(c) 477. Expression in response to trichostatin A (TSA): 15min,30min,60min,120min(1)
(c) 477. Expression in response to trichostatin A (TSA): 15min,30min,60min,120min(2)
(c) 477. Expression in response to trichostatin A (TSA): 15min,30min,60min,120min(3)
(c) 477. Expression in response to trichostatin A (TSA): 15min,30min,60min,120min(4)
(c) 479. Expression in diploid cells in response to rapamycin (100nM) for: 15min,30min,90min,120min(1)
(c) 479. Expression in diploid cells in response to rapamycin (100nM) for: 15min,30min,90min,120min(2)
(c) 479. Expression in diploid cells in response to rapamycin (100nM) for: 15min,30min,90min,120min(4)
(c) 578. Brown enviromental changes :aa starv 0.5 h(1)

(c) 579. Brown enviromental changes :aa starv 1 h(1)
(c) 580. Brown enviromental changes :aa starv 2 h(1)
(c) 581. Brown enviromental changes :aa starv 4 h(1)
(c) 582. Brown enviromental changes :aa starv 6 h(1)
(c) 583. Brown enviromental changes :Nitrogen Depletion 30 min.(1)
(c) 584. Brown enviromental changes :Nitrogen Depletion 1 h(1)
(c) 585. Brown enviromental changes :Nitrogen Depletion 2 h(1)
(c) 586. Brown enviromental changes :Nitrogen Depletion 4 h(1)
(c) 587. Brown enviromental changes :Nitrogen Depletion 8 h(1)
(c) 588. Brown enviromental changes :Nitrogen Depletion 12 h(1)
(c) 589. Brown enviromental changes :Nitrogen Depletion 1 d(1)
(c) 590. Brown enviromental changes :Nitrogen Depletion 2 d(1)
(c) 591. Brown enviromental changes :Nitrogen Depletion 3 d(1)
(c) 592. Brown enviromental changes :Nitrogen Depletion 5 d(1)
(c) 617. Brown enviromental changes :YPD stationary phase 3 d ypd-1(1)

GAT1 -*-> YEL072W

(c) 428. Expression in strain PM38 (wild type) in response to 30 min 50 nM treatment with rapamycin in YPD(1)
(c) 429. Expression in strain YHE711 (wild type) in response to 30 min 50 nM treatment with rapamycin in YPD(1)
(c) 439. Expression in strain Jk9-3da (wild type) in response to 30 min 50 nM treatment with rapamycin in YPD(1)
(c) 442. Expression in strain PM38 (wild type) in response to 30 min 50 nM treatment with rapamycin in YPD(1)
(c) 477. Expression in response to trichostatin A (TSA): 15min,30min,60min,120min(1)
(c) 477. Expression in response to trichostatin A (TSA): 15min,30min,60min,120min(2)
(c) 477. Expression in response to trichostatin A (TSA): 15min,30min,60min,120min(3)
(c) 477. Expression in response to trichostatin A (TSA): 15min,30min,60min,120min(4)
(c) 479. Expression in diploid cells in response to rapamycin (100nM) for: 15min,30min,90min,120min(1)
(c) 479. Expression in diploid cells in response to rapamycin (100nM) for: 15min,30min,90min,120min(2)
(c) 479. Expression in diploid cells in response to rapamycin (100nM) for: 15min,30min,90min,120min(4)
(c) 578. Brown enviromental changes :aa starv 0.5 h(1)
(c) 579. Brown enviromental changes :aa starv 1 h(1)
(c) 580. Brown enviromental changes :aa starv 2 h(1)
(c) 581. Brown enviromental changes :aa starv 4 h(1)
(c) 582. Brown enviromental changes :aa starv 6 h(1)
(c) 583. Brown enviromental changes :Nitrogen Depletion 30 min.(1)
(c) 584. Brown enviromental changes :Nitrogen Depletion 1 h(1)
(c) 585. Brown enviromental changes :Nitrogen Depletion 2 h(1)
(c) 586. Brown enviromental changes :Nitrogen Depletion 4 h(1)
(c) 587. Brown enviromental changes :Nitrogen Depletion 8 h(1)
(c) 588. Brown enviromental changes :Nitrogen Depletion 12 h(1)
(c) 589. Brown enviromental changes :Nitrogen Depletion 1 d(1)
(c) 590. Brown enviromental changes :Nitrogen Depletion 2 d(1)
(c) 591. Brown enviromental changes :Nitrogen Depletion 3 d(1)
(c) 592. Brown enviromental changes :Nitrogen Depletion 5 d(1)
(c) 617. Brown enviromental changes :YPD stationary phase 3 d ypd-1(1)

GAT1 -*-> YMR088C

(c) 428. Expression in strain PM38 (wild type) in response to 30 min 50 nM treatment with rapamycin in YPD(1)
(c) 429. Expression in strain YHE711 (wild type) in response to 30 min 50 nM treatment with rapamycin in YPD(1)
(c) 439. Expression in strain Jk9-3da (wild type) in response to 30 min 50 nM treatment with rapamycin in YPD(1)
(c) 442. Expression in strain PM38 (wild type) in response to 30 min 50 nM treatment with rapamycin in YPD(1)
(c) 477. Expression in response to trichostatin A (TSA): 15min,30min,60min,120min(1)
(c) 477. Expression in response to trichostatin A (TSA): 15min,30min,60min,120min(2)
(c) 477. Expression in response to trichostatin A (TSA): 15min,30min,60min,120min(3)
(c) 477. Expression in response to trichostatin A (TSA): 15min,30min,60min,120min(4)
(c) 479. Expression in diploid cells in response to rapamycin (100nM) for: 15min,30min,90min,120min(1)
(c) 479. Expression in diploid cells in response to rapamycin (100nM) for: 15min,30min,90min,120min(2)
(c) 479. Expression in diploid cells in response to rapamycin (100nM) for: 15min,30min,90min,120min(4)
(c) 578. Brown enviromental changes :aa starv 0.5 h(1)
(c) 579. Brown enviromental changes :aa starv 1 h(1)
(c) 580. Brown enviromental changes :aa starv 2 h(1)
(c) 581. Brown enviromental changes :aa starv 4 h(1)
(c) 582. Brown enviromental changes :aa starv 6 h(1)
(c) 583. Brown enviromental changes :Nitrogen Depletion 30 min.(1)
(c) 584. Brown enviromental changes :Nitrogen Depletion 1 h(1)
(c) 585. Brown enviromental changes :Nitrogen Depletion 2 h(1)
(c) 586. Brown enviromental changes :Nitrogen Depletion 4 h(1)
(c) 587. Brown enviromental changes :Nitrogen Depletion 8 h(1)
(c) 588. Brown enviromental changes :Nitrogen Depletion 12 h(1)
(c) 589. Brown enviromental changes :Nitrogen Depletion 1 d(1)
(c) 590. Brown enviromental changes :Nitrogen Depletion 2 d(1)
(c) 591. Brown enviromental changes :Nitrogen Depletion 3 d(1)
(c) 592. Brown enviromental changes :Nitrogen Depletion 5 d(1)
(c) 617. Brown enviromental changes :YPD stationary phase 3 d ypd-1(1)

GAT1 -*-> CIT2

(c) 428. Expression in strain PM38 (wild type) in response to 30 min 50 nM treatment with rapamycin in YPD(1)
(c) 429. Expression in strain YHE711 (wild type) in response to 30 min 50 nM treatment with rapamycin in YPD(1)
(c) 439. Expression in strain Jk9-3da (wild type) in response to 30 min 50 nM treatment with rapamycin in YPD(1)
(c) 442. Expression in strain PM38 (wild type) in response to 30 min 50 nM treatment with rapamycin in YPD(1)
(c) 477. Expression in response to trichostatin A (TSA): 15min,30min,60min,120min(1)
(c) 477. Expression in response to trichostatin A (TSA): 15min,30min,60min,120min(2)
(c) 477. Expression in response to trichostatin A (TSA): 15min,30min,60min,120min(3)
(c) 477. Expression in response to trichostatin A (TSA): 15min,30min,60min,120min(4)
(c) 479. Expression in diploid cells in response to rapamycin (100nM) for: 15min,30min,90min,120min(2)
(c) 479. Expression in diploid cells in response to rapamycin (100nM) for: 15min,30min,90min,120min(4)
(c) 578. Brown enviromental changes :aa starv 0.5 h(1)
(c) 579. Brown enviromental changes :aa starv 1 h(1)
(c) 580. Brown enviromental changes :aa starv 2 h(1)
(c) 581. Brown enviromental changes :aa starv 4 h(1)
(c) 582. Brown enviromental changes :aa starv 6 h(1)
(c) 583. Brown enviromental changes :Nitrogen Depletion 30 min.(1)
(c) 584. Brown enviromental changes :Nitrogen Depletion 1 h(1)
(c) 585. Brown enviromental changes :Nitrogen Depletion 2 h(1)
(c) 586. Brown enviromental changes :Nitrogen Depletion 4 h(1)

GCN4 -*-> ADE12

(c) 5. Expression during the cell cycle (alpha factor arrest and release)(2)
(c) 6. Expression during the cell cycle (cdc15 arrest and release)(12)
(c) 11. Expression during diauxic shift: 9h,11h,13h,15h,17h,19h,21h(3)
(c) 89. Expression in response to 3-aminotriazole(1)

(c) 402. Rosetta 2000: Expression in response to Itraconazole(1)
(c) 407. Rosetta 2000: Expression in response to Tunicamycin(1)
(c) 538. Brown enviromental changes :1 mM Menadione (80 min) redo(1)
(c) 539. Brown enviromental changes :1 mM Menadione (105 min) redo(1)
(c) 540. Brown enviromental changes :1 mM Menadione (120 min)redo(1)
(c) 541. Brown enviromental changes :1 mM Menadione (160 min) redo(1)
(c) 552. Brown enviromental changes :dtt 030 min dtt-2(1)
(c) 556. Brown enviromental changes :dtt 480 min dtt-2(1)
(c) 572. Brown enviromental changes :Hypo-osmotic shock - 5 min(1)
(c) 579. Brown enviromental changes :aa starv 1 h(1)
(c) 580. Brown enviromental changes :aa starv 2 h(1)
(c) 581. Brown enviromental changes :aa starv 4 h(1)
(c) 582. Brown enviromental changes :aa starv 6 h(1)
(c) 583. Brown enviromental changes :Nitrogen Depletion 30 min.(1)
(c) 585. Brown enviromental changes :Nitrogen Depletion 2 h(1)
(c) 586. Brown enviromental changes :Nitrogen Depletion 4 h(1)
(c) 595. Brown enviromental changes :diauxic shift timecourse(1)
(c) 601. Brown enviromental changes :YPD 2 h ypd-2(1)
(c) 602. Brown enviromental changes :YPD 4 h ypd-2(1)
(c) 612. Brown enviromental changes :YPD stationary phase 4 h ypd-1(1)
(c) 613. Brown enviromental changes :YPD stationary phase 8 h ypd-1(1)
(c) 681. Expression in response to 0.4M NaCl for 10 min in wild type(1)
(c) 684. Expression in response to 0.8M NaCl for 10 min in wild type(1)
(c) 100 microM BCS 30 min

GCN4 -*-> ADE13

(c) 5. Expression during the cell cycle (alpha factor arrest and release)(2)
(c) 6. Expression during the cell cycle (cdc15 arrest and release)(12)
(c) 11. Expression during diauxic shift: 9h,11h,13h,15h,17h,19h,21h(3)
(c) 89. Expression in response to 3-aminotriazole(1)
(c) 402. Rosetta 2000: Expression in response to Itraconazole(1)
(c) 407. Rosetta 2000: Expression in response to Tunicamycin(1)
(c) 538. Brown enviromental changes :1 mM Menadione (80 min) redo(1)
(c) 539. Brown enviromental changes :1 mM Menadione (105 min) redo(1)
(c) 540. Brown enviromental changes :1 mM Menadione (120 min)redo(1)
(c) 541. Brown enviromental changes :1 mM Menadione (160 min) redo(1)
(c) 552. Brown enviromental changes :dtt 030 min dtt-2(1)
(c) 556. Brown enviromental changes :dtt 480 min dtt-2(1)
(c) 572. Brown enviromental changes :Hypo-osmotic shock - 5 min(1)
(c) 579. Brown enviromental changes :aa starv 1 h(1)
(c) 580. Brown enviromental changes :aa starv 2 h(1)
(c) 581. Brown enviromental changes :aa starv 4 h(1)
(c) 582. Brown enviromental changes :aa starv 6 h(1)
(c) 583. Brown enviromental changes :Nitrogen Depletion 30 min.(1)
(c) 585. Brown enviromental changes :Nitrogen Depletion 2 h(1)
(c) 586. Brown enviromental changes :Nitrogen Depletion 4 h(1)
(c) 595. Brown enviromental changes :diauxic shift timecourse(1)
(c) 601. Brown enviromental changes :YPD 2 h ypd-2(1)
(c) 602. Brown enviromental changes :YPD 4 h ypd-2(1)
(c) 612. Brown enviromental changes :YPD stationary phase 4 h ypd-1(1)
(c) 613. Brown enviromental changes :YPD stationary phase 8 h ypd-1(1)
(c) 681. Expression in response to 0.4M NaCl for 10 min in wild type(1)
(c) 684. Expression in response to 0.8M NaCl for 10 min in wild type(1)
(c) 100 microM BCS 30 min

GCN4 -*-> ADE17

(c) 5. Expression during the cell cycle (alpha factor arrest and release)(2)
(c) 6. Expression during the cell cycle (cdc15 arrest and release)(12)
(c) 11. Expression during diauxic shift: 9h,11h,13h,15h,17h,19h,21h(3)
(c) 89. Expression in response to 3-aminotriazole(1)
(c) 402. Rosetta 2000: Expression in response to Itraconazole(1)
(c) 407. Rosetta 2000: Expression in response to Tunicamycin(1)
(c) 538. Brown enviromental changes :1 mM Menadione (80 min) redo(1)
(c) 539. Brown enviromental changes :1 mM Menadione (105 min) redo(1)
(c) 540. Brown enviromental changes :1 mM Menadione (120 min)redo(1)
(c) 541. Brown enviromental changes :1 mM Menadione (160 min) redo(1)
(c) 552. Brown enviromental changes :dtt 030 min dtt-2(1)
(c) 556. Brown enviromental changes :dtt 480 min dtt-2(1)
(c) 572. Brown enviromental changes :Hypo-osmotic shock - 5 min(1)
(c) 579. Brown enviromental changes :aa starv 1 h(1)
(c) 580. Brown enviromental changes :aa starv 2 h(1)
(c) 581. Brown enviromental changes :aa starv 4 h(1)
(c) 582. Brown enviromental changes :aa starv 6 h(1)
(c) 583. Brown enviromental changes :Nitrogen Depletion 30 min.(1)
(c) 585. Brown enviromental changes :Nitrogen Depletion 2 h(1)
(c) 586. Brown enviromental changes :Nitrogen Depletion 4 h(1)
(c) 595. Brown enviromental changes :diauxic shift timecourse(1)
(c) 601. Brown enviromental changes :YPD 2 h ypd-2(1)
(c) 602. Brown enviromental changes :YPD 4 h ypd-2(1)
(c) 612. Brown enviromental changes :YPD stationary phase 4 h ypd-1(1)
(c) 613. Brown enviromental changes :YPD stationary phase 8 h ypd-1(1)
(c) 681. Expression in response to 0.4M NaCl for 10 min in wild type(1)
(c) 684. Expression in response to 0.8M NaCl for 10 min in wild type(1)
(c) 100 microM BCS 30 min

GCN4 -*-> ETF-BETA

(c) 5. Expression during the cell cycle (alpha factor arrest and release)(2)
(c) 6. Expression during the cell cycle (cdc15 arrest and release)(12)
(c) 11. Expression during diauxic shift: 9h,11h,13h,15h,17h,19h,21h(3)
(c) 89. Expression in response to 3-aminotriazole(1)
(c) 402. Rosetta 2000: Expression in response to Itraconazole(1)
(c) 407. Rosetta 2000: Expression in response to Tunicamycin(1)
(c) 538. Brown enviromental changes :1 mM Menadione (80 min) redo(1)
(c) 539. Brown enviromental changes :1 mM Menadione (105 min) redo(1)
(c) 540. Brown enviromental changes :1 mM Menadione (120 min)redo(1)
(c) 541. Brown enviromental changes :1 mM Menadione (160 min) redo(1)
(c) 552. Brown enviromental changes :dtt 030 min dtt-2(1)
(c) 556. Brown enviromental changes :dtt 480 min dtt-2(1)
(c) 572. Brown enviromental changes :Hypo-osmotic shock - 5 min(1)
(c) 579. Brown enviromental changes :aa starv 1 h(1)
(c) 580. Brown enviromental changes :aa starv 2 h(1)
(c) 581. Brown enviromental changes :aa starv 4 h(1)
(c) 582. Brown enviromental changes :aa starv 6 h(1)

(c) 583. Brown enviromental changes :Nitrogen Depletion 30 min.(1)
(c) 585. Brown enviromental changes :Nitrogen Depletion 2 h(1)
(c) 586. Brown enviromental changes :Nitrogen Depletion 4 h(1)
(c) 595. Brown enviromental changes :diauxic shift timecourse(1)
(c) 601. Brown enviromental changes :YPD 2 h ypd-2(1)
(c) 602. Brown enviromental changes :YPD 4 h ypd-2(1)
(c) 612. Brown enviromental changes :YPD stationary phase 4 h ypd-1(1)
(c) 613. Brown enviromental changes :YPD stationary phase 8 h ypd-1(1)
(c) 681. Expression in response to 0.4M NaCl for 10 min in wild type(1)
(c) 684. Expression in response to 0.8M NaCl for 10 min in wild type(1)
(c) 100 microM BCS 30 min

GCN4 -*-> LYS12

(c) 5. Expression during the cell cycle (alpha factor arrest and release)(2)
(c) 6. Expression during the cell cycle (cdc15 arrest and release)(12)
(c) 11. Expression during diauxic shift: 9h,11h,13h,15h,17h,19h,21h(3)
(c) 89. Expression in response to 3-aminotriazole(1)
(c) 402. Rosetta 2000: Expression in response to Itraconazole(1)
(c) 407. Rosetta 2000: Expression in response to Tunicamycin(1)
(c) 538. Brown enviromental changes :1 mM Menadione (80 min) redo(1)
(c) 539. Brown enviromental changes :1 mM Menadione (105 min) redo(1)
(c) 540. Brown enviromental changes :1 mM Menadione (120 min)redo(1)
(c) 541. Brown enviromental changes :1 mM Menadione (160 min) redo(1)
(c) 552. Brown enviromental changes :dtt 030 min dtt-2(1)
(c) 556. Brown enviromental changes :dtt 480 min dtt-2(1)
(c) 572. Brown enviromental changes :Hypo-osmotic shock - 5 min(1)
(c) 579. Brown enviromental changes :aa starv 1 h(1)
(c) 580. Brown enviromental changes :aa starv 2 h(1)
(c) 581. Brown enviromental changes :aa starv 4 h(1)
(c) 582. Brown enviromental changes :aa starv 6 h(1)
(c) 583. Brown enviromental changes :Nitrogen Depletion 30 min.(1)
(c) 585. Brown enviromental changes :Nitrogen Depletion 2 h(1)
(c) 586. Brown enviromental changes :Nitrogen Depletion 4 h(1)
(c) 595. Brown enviromental changes :diauxic shift timecourse(1)
(c) 601. Brown enviromental changes :YPD 2 h ypd-2(1)
(c) 602. Brown enviromental changes :YPD 4 h ypd-2(1)
(c) 612. Brown enviromental changes :YPD stationary phase 4 h ypd-1(1)
(c) 613. Brown enviromental changes :YPD stationary phase 8 h ypd-1(1)
(c) 681. Expression in response to 0.4M NaCl for 10 min in wild type(1)
(c) 684. Expression in response to 0.8M NaCl for 10 min in wild type(1)
(c) 100 microM BCS 30 min

GCN4 -*-> MTD1

(c) 5. Expression during the cell cycle (alpha factor arrest and release)(2)
(c) 6. Expression during the cell cycle (cdc15 arrest and release)(12)
(c) 11. Expression during diauxic shift: 9h,11h,13h,15h,17h,19h,21h(3)
(c) 89. Expression in response to 3-aminotriazole(1)
(c) 402. Rosetta 2000: Expression in response to Itraconazole(1)
(c) 407. Rosetta 2000: Expression in response to Tunicamycin(1)
(c) 538. Brown enviromental changes :1 mM Menadione (80 min) redo(1)
(c) 539. Brown enviromental changes :1 mM Menadione (105 min) redo(1)
(c) 540. Brown enviromental changes :1 mM Menadione (120 min)redo(1)
(c) 541. Brown enviromental changes :1 mM Menadione (160 min) redo(1)
(c) 552. Brown enviromental changes :dtt 030 min dtt-2(1)
(c) 556. Brown enviromental changes :dtt 480 min dtt-2(1)
(c) 572. Brown enviromental changes :Hypo-osmotic shock - 5 min(1)
(c) 579. Brown enviromental changes :aa starv 1 h(1)
(c) 580. Brown enviromental changes :aa starv 2 h(1)
(c) 581. Brown enviromental changes :aa starv 4 h(1)
(c) 582. Brown enviromental changes :aa starv 6 h(1)
(c) 583. Brown enviromental changes :Nitrogen Depletion 30 min.(1)
(c) 585. Brown enviromental changes :Nitrogen Depletion 2 h(1)
(c) 586. Brown enviromental changes :Nitrogen Depletion 4 h(1)
(c) 595. Brown enviromental changes :diauxic shift timecourse(1)
(c) 601. Brown enviromental changes :YPD 2 h ypd-2(1)
(c) 602. Brown enviromental changes :YPD 4 h ypd-2(1)
(c) 612. Brown enviromental changes :YPD stationary phase 4 h ypd-1(1)
(c) 613. Brown enviromental changes :YPD stationary phase 8 h ypd-1(1)
(c) 681. Expression in response to 0.4M NaCl for 10 min in wild type(1)
(c) 684. Expression in response to 0.8M NaCl for 10 min in wild type(1)
(c) 100 microM BCS 30 min

GCN4 -*-> AAT2

(c) 89. Expression in response to 3-aminotriazole(1)
(c) 95. Expression in response to 50ug/mL FK506(1)
(c) 389. Rosetta 2000: Expression in cells with HMG2 under tet promoter(1)
(c) 401. Rosetta 2000: Expression in response to HU(1)
(c) 402. Rosetta 2000: Expression in response to Itraconazole(1)
(c) 403. Rosetta 2000: Expression in response to Lovastatin(1)
(c) 406. Rosetta 2000: Expression in response to Terbinafine(1)
(c) 407. Rosetta 2000: Expression in response to Tunicamycin(1)
(c) 429. Expression in strain YHE711 (wild type) in response to 30 min 50 nM treatment with rapamycin in YPD(1)
(c) 441. Expression in wild type versus strain TS19-4c under steady state conditions in YPD(1)
(c) 446. Expression in response to 0.1% MMS for 10 min(1)
(c) 447. Expression in response to 0.1% MMS for 30 min(1)
(c) 449. Expression in response to 0.1% MMS for 60 min(1)
(c) 450. Expression in response to low MNNG (8 microgram/ml) for 60 min(1)
(c) 479. Expression in diploid cells in response to rapamycin (100nM) for: 15min,30min,90min,120min(2)
(c) 479. Expression in diploid cells in response to rapamycin (100nM) for: 15min,30min,90min,120min(4)
(c) 486. Expression in response to NaCl: 15 30 45 60 120 min(4)
(c) 523. Brown enviromental changes :constant 0.32 mM H2O2 (10 min) redo(1)
(c) 551. Brown enviromental changes :dtt 015 min dtt-2(1)
(c) 579. Brown enviromental changes :aa starv 1 h(1)
(c) 580. Brown enviromental changes :aa starv 2 h(1)
(c) 581. Brown enviromental changes :aa starv 4 h(1)
(c) 582. Brown enviromental changes :aa starv 6 h(1)
(c) 583. Brown enviromental changes :Nitrogen Depletion 30 min.(1)
(c) 585. Brown enviromental changes :Nitrogen Depletion 2 h(1)
(c) 586. Brown enviromental changes :Nitrogen Depletion 4 h(1)
(c) 602. Brown enviromental changes :YPD 4 h ypd-2(1)
(c) 612. Brown enviromental changes :YPD stationary phase 4 h ypd-1(1)
(c) DES460 + 0.02% MMS - 5 min
(c) DES460 + 0.02% MMS - 15 min

(c) 100 microM BCS 30 min

GCN4 -*-> ATR1

(c) 89. Expression in response to 3-aminotriazole(1)
(c) 95. Expression in response to 50ug/mL FK506(1)
(c) 389. Rosetta 2000: Expression in cells with HMG2 under tet promoter(1)
(c) 401. Rosetta 2000: Expression in response to HU(1)
(c) 402. Rosetta 2000: Expression in response to Itraconazole(1)
(c) 403. Rosetta 2000: Expression in response to Lovastatin(1)
(c) 406. Rosetta 2000: Expression in response to Terbinafine(1)
(c) 407. Rosetta 2000: Expression in response to Tunicamycin(1)
(c) 429. Expression in strain YHE711 (wild type) in response to 30 min 50 nM treatment with rapamycin in YPD(1)
(c) 441. Expression in wild type versus strain TS19-4c under steady state conditions in YPD(1)
(c) 446. Expression in response to 0.1% MMS for 10 min(1)
(c) 447. Expression in response to 0.1% MMS for 30 min(1)
(c) 449. Expression in response to 0.1% MMS for 60 min(1)
(c) 450. Expression in response to low MNNG (8 microgram/ml) for 60 min(1)
(c) 479. Expression in diploid cells in response to rapamycin (100nM) for: 15min,30min,90min,120min(2)
(c) 479. Expression in diploid cells in response to rapamycin (100nM) for: 15min,30min,90min,120min(4)
(c) 486. Expression in response to NaCl: 15 30 45 60 120 min(4)
(c) 523. Brown enviromental changes :constant 0.32 mM H2O2 (10 min) redo(1)
(c) 551. Brown enviromental changes :dtt 015 min dtt-2(1)
(c) 579. Brown enviromental changes :aa starv 1 h(1)
(c) 580. Brown enviromental changes :aa starv 2 h(1)
(c) 581. Brown enviromental changes :aa starv 4 h(1)
(c) 582. Brown enviromental changes :aa starv 6 h(1)
(c) 583. Brown enviromental changes :Nitrogen Depletion 30 min.(1)
(c) 585. Brown enviromental changes :Nitrogen Depletion 2 h(1)
(c) 586. Brown enviromental changes :Nitrogen Depletion 4 h(1)
(c) 602. Brown enviromental changes :YPD 4 h ypd-2(1)
(c) 612. Brown enviromental changes :YPD stationary phase 4 h ypd-1(1)
(c) DES460 + 0.02% MMS - 5 min
(c) DES460 + 0.02% MMS - 15 min
(c) 100 microM BCS 30 min

GCN4 -*-> IDP1

(c) 89. Expression in response to 3-aminotriazole(1)
(c) 95. Expression in response to 50ug/mL FK506(1)
(c) 389. Rosetta 2000: Expression in cells with HMG2 under tet promoter(1)
(c) 401. Rosetta 2000: Expression in response to HU(1)
(c) 402. Rosetta 2000: Expression in response to Itraconazole(1)
(c) 403. Rosetta 2000: Expression in response to Lovastatin(1)
(c) 406. Rosetta 2000: Expression in response to Terbinafine(1)
(c) 407. Rosetta 2000: Expression in response to Tunicamycin(1)
(c) 429. Expression in strain YHE711 (wild type) in response to 30 min 50 nM treatment with rapamycin in YPD(1)
(c) 441. Expression in wild type versus strain TS19-4c under steady state conditions in YPD(1)
(c) 446. Expression in response to 0.1% MMS for 10 min(1)
(c) 447. Expression in response to 0.1% MMS for 30 min(1)
(c) 449. Expression in response to 0.1% MMS for 60 min(1)
(c) 450. Expression in response to low MNNG (8 microgram/ml) for 60 min(1)
(c) 479. Expression in diploid cells in response to rapamycin (100nM) for: 15min,30min,90min,120min(2)
(c) 479. Expression in diploid cells in response to rapamycin (100nM) for: 15min,30min,90min,120min(4)
(c) 486. Expression in response to NaCl: 15 30 45 60 120 min(4)
(c) 523. Brown enviromental changes :constant 0.32 mM H2O2 (10 min) redo(1)
(c) 551. Brown enviromental changes :dtt 015 min dtt-2(1)
(c) 579. Brown enviromental changes :aa starv 1 h(1)
(c) 580. Brown enviromental changes :aa starv 2 h(1)
(c) 581. Brown enviromental changes :aa starv 4 h(1)
(c) 582. Brown enviromental changes :aa starv 6 h(1)
(c) 583. Brown enviromental changes :Nitrogen Depletion 30 min.(1)
(c) 585. Brown enviromental changes :Nitrogen Depletion 2 h(1)
(c) 586. Brown enviromental changes :Nitrogen Depletion 4 h(1)
(c) 602. Brown enviromental changes :YPD 4 h ypd-2(1)
(c) 612. Brown enviromental changes :YPD stationary phase 4 h ypd-1(1)
(c) DES460 + 0.02% MMS - 5 min
(c) DES460 + 0.02% MMS - 15 min
(c) 100 microM BCS 30 min

GCN4 -*-> ODC2

(c) 89. Expression in response to 3-aminotriazole(1)
(c) 95. Expression in response to 50ug/mL FK506(1)
(c) 389. Rosetta 2000: Expression in cells with HMG2 under tet promoter(1)
(c) 401. Rosetta 2000: Expression in response to HU(1)
(c) 402. Rosetta 2000: Expression in response to Itraconazole(1)
(c) 403. Rosetta 2000: Expression in response to Lovastatin(1)
(c) 406. Rosetta 2000: Expression in response to Terbinafine(1)
(c) 407. Rosetta 2000: Expression in response to Tunicamycin(1)
(c) 429. Expression in strain YHE711 (wild type) in response to 30 min 50 nM treatment with rapamycin in YPD(1)
(c) 441. Expression in wild type versus strain TS19-4c under steady state conditions in YPD(1)
(c) 446. Expression in response to 0.1% MMS for 10 min(1)
(c) 447. Expression in response to 0.1% MMS for 30 min(1)
(c) 449. Expression in response to 0.1% MMS for 60 min(1)
(c) 450. Expression in response to low MNNG (8 microgram/ml) for 60 min(1)
(c) 479. Expression in diploid cells in response to rapamycin (100nM) for: 15min,30min,90min,120min(2)
(c) 479. Expression in diploid cells in response to rapamycin (100nM) for: 15min,30min,90min,120min(4)
(c) 486. Expression in response to NaCl: 15 30 45 60 120 min(4)
(c) 523. Brown enviromental changes :constant 0.32 mM H2O2 (10 min) redo(1)
(c) 551. Brown enviromental changes :dtt 015 min dtt-2(1)
(c) 579. Brown enviromental changes :aa starv 1 h(1)
(c) 580. Brown enviromental changes :aa starv 2 h(1)
(c) 581. Brown enviromental changes :aa starv 4 h(1)
(c) 582. Brown enviromental changes :aa starv 6 h(1)
(c) 583. Brown enviromental changes :Nitrogen Depletion 30 min.(1)
(c) 585. Brown enviromental changes :Nitrogen Depletion 2 h(1)
(c) 586. Brown enviromental changes :Nitrogen Depletion 4 h(1)
(c) 602. Brown enviromental changes :YPD 4 h ypd-2(1)
(c) 612. Brown enviromental changes :YPD stationary phase 4 h ypd-1(1)
(c) DES460 + 0.02% MMS - 5 min
(c) DES460 + 0.02% MMS - 15 min
(c) 100 microM BCS 30 min

GCN4 -*-> YHM1

(c) 89. Expression in response to 3-aminotriazole(1)
(c) 95. Expression in response to 50ug/mL FK506(1)
(c) 389. Rosetta 2000: Expression in cells with HMG2 under tet promoter(1)
(c) 401. Rosetta 2000: Expression in response to HU(1)
(c) 402. Rosetta 2000: Expression in response to Itraconazole(1)
(c) 403. Rosetta 2000: Expression in response to Lovastatin(1)
(c) 406. Rosetta 2000: Expression in response to Terbinafine(1)
(c) 407. Rosetta 2000: Expression in response to Tunicamycin(1)
(c) 429. Expression in strain YHE711 (wild type) in response to 30 min 50 nM treatment with rapamycin in YPD(1)
(c) 441. Expression in wild type versus strain TS19-4c under steady state conditions in YPD(1)
(c) 446. Expression in response to 0.1% MMS for 10 min(1)
(c) 447. Expression in response to 0.1% MMS for 30 min(1)
(c) 449. Expression in response to 0.1% MMS for 60 min(1)
(c) 450. Expression in response to low MNNG (8 microgram/ml) for 60 min(1)
(c) 479. Expression in diploid cells in response to rapamycin (100nM) for: 15min,30min,90min,120min(2)
(c) 479. Expression in diploid cells in response to rapamycin (100nM) for: 15min,30min,90min,120min(4)
(c) 486. Expression in response to NaCl: 15 30 45 60 120 min(4)
(c) 523. Brown enviromental changes :constant 0.32 mM H2O2 (10 min) redo(1)
(c) 551. Brown enviromental changes :dtt 015 min dtt-2(1)
(c) 579. Brown enviromental changes :aa starv 1 h(1)
(c) 580. Brown enviromental changes :aa starv 2 h(1)
(c) 581. Brown enviromental changes :aa starv 4 h(1)
(c) 582. Brown enviromental changes :aa starv 6 h(1)
(c) 583. Brown enviromental changes :Nitrogen Depletion 30 min.(1)
(c) 585. Brown enviromental changes :Nitrogen Depletion 2 h(1)
(c) 586. Brown enviromental changes :Nitrogen Depletion 4 h(1)
(c) 602. Brown enviromental changes :YPD 4 h ypd-2(1)
(c) 612. Brown enviromental changes :YPD stationary phase 4 h ypd-1(1)
(c) DES460 + 0.02% MMS - 5 min
(c) DES460 + 0.02% MMS - 15 min
(c) 100 microM BCS 30 min

GCN4 -*-> YMC1

(c) 5. Expression during the cell cycle (alpha factor arrest and release)(11)
(c) 6. Expression during the cell cycle (cdc15 arrest and release)(15)
(c) 54. Expression in response to overproduction of Ste5p(1)
(c) 89. Expression in response to 3-aminotriazole(1)
(c) 95. Expression in response to 50ug/mL FK506(1)
(c) 402. Rosetta 2000: Expression in response to Itraconazole(1)
(c) 403. Rosetta 2000: Expression in response to Lovastatin(1)
(c) 406. Rosetta 2000: Expression in response to Terbinafine(1)
(c) 407. Rosetta 2000: Expression in response to Tunicamycin(1)
(c) 429. Expression in strain YHE711 (wild type) in response to 30 min 50 nM treatment with rapamycin in YPD(1)
(c) 446. Expression in response to 0.1% MMS for 10 min(1)
(c) 447. Expression in response to 0.1% MMS for 30 min(1)
(c) 452. Expression in response to low 4NQO (2 microgram/ml) for 60 min(1)
(c) 479. Expression in diploid cells in response to rapamycin (100nM) for: 15min,30min,90min,120min(2)
(c) 481. Expression in response to heat shock: 15,30,45,60,120 min(2)
(c) 481. Expression in response to heat shock: 15,30,45,60,120 min(4)
(c) 537. Brown enviromental changes :1 mM Menadione (50 min)redo(1)
(c) 552. Brown enviromental changes :dtt 030 min dtt-2(1)
(c) 564. Brown enviromental changes :1.5 mM diamide (90 min)(1)
(c) 612. Brown enviromental changes :YPD stationary phase 4 h ypd-1(1)
(c) 684. Expression in response to 0.8M NaCl for 10 min in wild type(1)
(c) DES460 + 0.02% MMS - 5 min
(c) DES460 + 0.02% MMS - 15 min
(c) 100 microM BCS 30 min

GCN4 -*-> SFT2

(c) 89. Expression in response to 3-aminotriazole(1)
(c) 95. Expression in response to 50ug/mL FK506(1)
(c) 387. Rosetta 2000: Expression in cells with ERG11 under tet promoter(1)
(c) 401. Rosetta 2000: Expression in response to HU(1)
(c) 402. Rosetta 2000: Expression in response to Itraconazole(1)
(c) 403. Rosetta 2000: Expression in response to Lovastatin(1)
(c) 406. Rosetta 2000: Expression in response to Terbinafine(1)
(c) 407. Rosetta 2000: Expression in response to Tunicamycin(1)
(c) 446. Expression in response to 0.1% MMS for 10 min(1)
(c) 450. Expression in response to low MNNG (8 microgram/ml) for 60 min(1)
(c) 479. Expression in diploid cells in response to rapamycin (100nM) for: 15min,30min,90min,120min(2)
(c) 479. Expression in diploid cells in response to rapamycin (100nM) for: 15min,30min,90min,120min(3)
(c) 523. Brown enviromental changes :constant 0.32 mM H2O2 (10 min) redo(1)
(c) 533. Brown enviromental changes :1 mM Menadione (10 min)redo(1)
(c) 537. Brown enviromental changes :1 mM Menadione (50 min)redo(1)
(c) 538. Brown enviromental changes :1 mM Menadione (80 min) redo(1)
(c) 572. Brown enviromental changes :Hypo-osmotic shock - 5 min(1)
(c) 581. Brown enviromental changes :aa starv 4 h(1)
(c) 582. Brown enviromental changes :aa starv 6 h(1)
(c) 612. Brown enviromental changes :YPD stationary phase 4 h ypd-1(1)
(c) 613. Brown enviromental changes :YPD stationary phase 8 h ypd-1(1)
(c) 684. Expression in response to 0.8M NaCl for 10 min in wild type(1)
(c) DES460 + 0.02% MMS - 5 min
(c) DES460 + 0.02% MMS - 15 min
(c) 100 microM BCS 30 min

GCN4 -*-> ALD5

(c) 89. Expression in response to 3-aminotriazole(1)
(c) 95. Expression in response to 50ug/mL FK506(1)
(c) 387. Rosetta 2000: Expression in cells with ERG11 under tet promoter(1)
(c) 389. Rosetta 2000: Expression in cells with HMG2 under tet promoter(1)
(c) 395. Rosetta 2000: Expression in response to 2-deoxy-D-glucose(1)
(c) 401. Rosetta 2000: Expression in response to HU(1)
(c) 402. Rosetta 2000: Expression in response to Itraconazole(1)
(c) 403. Rosetta 2000: Expression in response to Lovastatin(1)
(c) 406. Rosetta 2000: Expression in response to Terbinafine(1)
(c) 407. Rosetta 2000: Expression in response to Tunicamycin(1)
(c) 446. Expression in response to 0.1% MMS for 10 min(1)
(c) 447. Expression in response to 0.1% MMS for 30 min(1)
(c) 481. Expression in response to heat shock: 15,30,45,60,120 min(4)
(c) 502. Brown enviromental changes :Heat Shock 060 minutes hs-2(1)
(c) 538. Brown enviromental changes :1 mM Menadione (80 min) redo(1)

(c) 540. Brown enviromental changes :1 mM Menadione (120 min)redo(1)
(c) 556. Brown enviromental changes :dtt 480 min dtt-2(1)
(c) 579. Brown enviromental changes :aa starv 1 h(1)
(c) 580. Brown enviromental changes :aa starv 2 h(1)
(c) 581. Brown enviromental changes :aa starv 4 h(1)
(c) 585. Brown enviromental changes :Nitrogen Depletion 2 h(1)
(c) 586. Brown enviromental changes :Nitrogen Depletion 4 h(1)
(c) 602. Brown enviromental changes :YPD 4 h ypd-2(1)
(c) 612. Brown enviromental changes :YPD stationary phase 4 h ypd-1(1)
(c) 613. Brown enviromental changes :YPD stationary phase 8 h ypd-1(1)
(c) 684. Expression in response to 0.8M NaCl for 10 min in wild type(1)
(c) DES460 + 0.02% MMS - 5 min
(c) DES460 + 0.02% MMS - 15 min

GCN4 -*-> ISU1

(c) 6. Expression during the cell cycle (cdc15 arrest and release)(9)
(c) 8. Expression during the cell cycle (cell size selection and release)(3)
(c) 11. Expression during diauxic shift: 9h,11h,13h,15h,17h,19h,21h(3)
(c) 89. Expression in response to 3-aminotriazole(1)
(c) 95. Expression in response to 50ug/mL FK506(1)
(c) 402. Rosetta 2000: Expression in response to Itraconazole(1)
(c) 403. Rosetta 2000: Expression in response to Lovastatin(1)
(c) 406. Rosetta 2000: Expression in response to Terbinafine(1)
(c) 407. Rosetta 2000: Expression in response to Tunicamycin(1)
(c) 441. Expression in wild type versus strain TS19-4c under steady state conditions in YPD(1)
(c) 446. Expression in response to 0.1% MMS for 10 min(1)
(c) 447. Expression in response to 0.1% MMS for 30 min(1)
(c) 479. Expression in diploid cells in response to rapamycin (100nM) for: 15min,30min,90min,120min(2)
(c) 503. Brown enviromental changes :37C to 25C shock - 15 min(1)
(c) 504. Brown enviromental changes :37C to 25C shock - 30 min(1)
(c) 516. Brown enviromental changes :33C vs. 30C - 90 minutes(1)
(c) 533. Brown enviromental changes :1 mM Menadione (10 min)redo(1)
(c) 544. Brown enviromental changes :2.5mM DTT 030 min dtt-1(1)
(c) 572. Brown enviromental changes :Hypo-osmotic shock - 5 min(1)
(c) 573. Brown enviromental changes :Hypo-osmotic shock - 15 min(1)
(c) 580. Brown enviromental changes :aa starv 2 h(1)
(c) 581. Brown enviromental changes :aa starv 4 h(1)
(c) 582. Brown enviromental changes :aa starv 6 h(1)
(c) 585. Brown enviromental changes :Nitrogen Depletion 2 h(1)
(c) 586. Brown enviromental changes :Nitrogen Depletion 4 h(1)
(c) 588. Brown enviromental changes :Nitrogen Depletion 12 h(1)
(c) DES460 + 0.02% MMS - 5 min
(c) 100 microM BCS 30 min

GCN4 -*-> YMC2

(c) 6. Expression during the cell cycle (cdc15 arrest and release)(9)
(c) 8. Expression during the cell cycle (cell size selection and release)(3)
(c) 11. Expression during diauxic shift: 9h,11h,13h,15h,17h,19h,21h(3)
(c) 89. Expression in response to 3-aminotriazole(1)
(c) 95. Expression in response to 50ug/mL FK506(1)
(c) 402. Rosetta 2000: Expression in response to Itraconazole(1)
(c) 403. Rosetta 2000: Expression in response to Lovastatin(1)
(c) 406. Rosetta 2000: Expression in response to Terbinafine(1)
(c) 407. Rosetta 2000: Expression in response to Tunicamycin(1)
(c) 441. Expression in wild type versus strain TS19-4c under steady state conditions in YPD(1)
(c) 446. Expression in response to 0.1% MMS for 10 min(1)
(c) 447. Expression in response to 0.1% MMS for 30 min(1)
(c) 479. Expression in diploid cells in response to rapamycin (100nM) for: 15min,30min,90min,120min(2)
(c) 503. Brown enviromental changes :37C to 25C shock - 15 min(1)
(c) 504. Brown enviromental changes :37C to 25C shock - 30 min(1)
(c) 516. Brown enviromental changes :33C vs. 30C - 90 minutes(1)
(c) 533. Brown enviromental changes :1 mM Menadione (10 min)redo(1)
(c) 544. Brown enviromental changes :2.5mM DTT 030 min dtt-1(1)
(c) 572. Brown enviromental changes :Hypo-osmotic shock - 5 min(1)
(c) 573. Brown enviromental changes :Hypo-osmotic shock - 15 min(1)
(c) 580. Brown enviromental changes :aa starv 2 h(1)
(c) 581. Brown enviromental changes :aa starv 4 h(1)
(c) 582. Brown enviromental changes :aa starv 6 h(1)
(c) 585. Brown enviromental changes :Nitrogen Depletion 2 h(1)
(c) 586. Brown enviromental changes :Nitrogen Depletion 4 h(1)
(c) 588. Brown enviromental changes :Nitrogen Depletion 12 h(1)
(c) DES460 + 0.02% MMS - 5 min
(c) 100 microM BCS 30 min

GCN4 -*-> BNA1

(c) 387. Rosetta 2000: Expression in cells with ERG11 under tet promoter(1)
(c) 392. Rosetta 2000: Expression in cells with PMA1 under tet promoter(1)
(c) 395. Rosetta 2000: Expression in response to 2-deoxy-D-glucose(1)
(c) 402. Rosetta 2000: Expression in response to Itraconazole(1)
(c) 406. Rosetta 2000: Expression in response to Terbinafine(1)
(c) 407. Rosetta 2000: Expression in response to Tunicamycin(1)
(c) 445. Expression in response to 0.1% MMS for 60 min (average of 3 experiments)(1)
(c) 446. Expression in response to 0.1% MMS for 10 min(1)
(c) 447. Expression in response to 0.1% MMS for 30 min(1)
(c) 448. Expression in response to 0.1% MMS for 60 min(1)
(c) 449. Expression in response to 0.1% MMS for 60 min(1)
(c) 450. Expression in response to low MNNG (8 microgram/ml) for 60 min(1)
(c) 462. Expression in response to 0.05% MMS for 60 min(1)
(c) 504. Brown enviromental changes :37C to 25C shock - 30 min(1)
(c) 523. Brown enviromental changes :constant 0.32 mM H2O2 (10 min) redo(1)
(c) 533. Brown enviromental changes :1 mM Menadione (10 min)redo(1)
(c) 534. Brown enviromental changes :1 mM Menadione (20 min) redo(1)
(c) 535. Brown enviromental changes :1 mM Menadione (30 min) redo(1)
(c) 537. Brown enviromental changes :1 mM Menadione (50 min)redo(1)
(c) 538. Brown enviromental changes :1 mM Menadione (80 min) redo(1)
(c) 581. Brown enviromental changes :aa starv 4 h(1)
(c) 582. Brown enviromental changes :aa starv 6 h(1)
(c) 585. Brown enviromental changes :Nitrogen Depletion 2 h(1)
(c) 612. Brown enviromental changes :YPD stationary phase 4 h ypd-1(1)
(c) DES460 + 0.02% MMS - 5 min
(c) DES460 + 0.02% MMS - 15 min

GCN4 -*-> CAF16

(c) 387. Rosetta 2000: Expression in cells with ERG11 under tet promoter(1)
(c) 392. Rosetta 2000: Expression in cells with PMA1 under tet promoter(1)
(c) 395. Rosetta 2000: Expression in response to 2-deoxy-D-glucose(1)
(c) 402. Rosetta 2000: Expression in response to Itraconazole(1)
(c) 406. Rosetta 2000: Expression in response to Terbinafine(1)
(c) 407. Rosetta 2000: Expression in response to Tunicamycin(1)
(c) 445. Expression in response to 0.1% MMS for 60 min (average of 3 experiments)(1)
(c) 446. Expression in response to 0.1% MMS for 10 min(1)
(c) 447. Expression in response to 0.1% MMS for 30 min(1)
(c) 448. Expression in response to 0.1% MMS for 60 min(1)
(c) 449. Expression in response to 0.1% MMS for 60 min(1)
(c) 450. Expression in response to low MNNG (8 microgram/ml) for 60 min(1)
(c) 462. Expression in response to 0.05% MMS for 60 min(1)
(c) 504. Brown enviromental changes :37C to 25C shock - 30 min(1)
(c) 523. Brown enviromental changes :constant 0.32 mM H2O2 (10 min) redo(1)
(c) 533. Brown enviromental changes :1 mM Menadione (10 min)redo(1)
(c) 534. Brown enviromental changes :1 mM Menadione (20 min) redo(1)
(c) 535. Brown enviromental changes :1 mM Menadione (30 min) redo(1)
(c) 537. Brown enviromental changes :1 mM Menadione (50 min)redo(1)
(c) 538. Brown enviromental changes :1 mM Menadione (80 min) redo(1)
(c) 581. Brown enviromental changes :aa starv 4 h(1)
(c) 582. Brown enviromental changes :aa starv 6 h(1)
(c) 585. Brown enviromental changes :Nitrogen Depletion 2 h(1)
(c) 612. Brown enviromental changes :YPD stationary phase 4 h ypd-1(1)
(c) DES460 + 0.02% MMS - 5 min
(c) DES460 + 0.02% MMS - 15 min

GCN4 -*-> MET22

(c) 387. Rosetta 2000: Expression in cells with ERG11 under tet promoter(1)
(c) 392. Rosetta 2000: Expression in cells with PMA1 under tet promoter(1)
(c) 395. Rosetta 2000: Expression in response to 2-deoxy-D-glucose(1)
(c) 402. Rosetta 2000: Expression in response to Itraconazole(1)
(c) 406. Rosetta 2000: Expression in response to Terbinafine(1)
(c) 407. Rosetta 2000: Expression in response to Tunicamycin(1)
(c) 445. Expression in response to 0.1% MMS for 60 min (average of 3 experiments)(1)
(c) 446. Expression in response to 0.1% MMS for 10 min(1)
(c) 447. Expression in response to 0.1% MMS for 30 min(1)
(c) 448. Expression in response to 0.1% MMS for 60 min(1)
(c) 449. Expression in response to 0.1% MMS for 60 min(1)
(c) 450. Expression in response to low MNNG (8 microgram/ml) for 60 min(1)
(c) 462. Expression in response to 0.05% MMS for 60 min(1)
(c) 504. Brown enviromental changes :37C to 25C shock - 30 min(1)
(c) 523. Brown enviromental changes :constant 0.32 mM H2O2 (10 min) redo(1)
(c) 533. Brown enviromental changes :1 mM Menadione (10 min)redo(1)
(c) 534. Brown enviromental changes :1 mM Menadione (20 min) redo(1)
(c) 535. Brown enviromental changes :1 mM Menadione (30 min) redo(1)
(c) 537. Brown enviromental changes :1 mM Menadione (50 min)redo(1)
(c) 538. Brown enviromental changes :1 mM Menadione (80 min) redo(1)
(c) 581. Brown enviromental changes :aa starv 4 h(1)
(c) 582. Brown enviromental changes :aa starv 6 h(1)
(c) 585. Brown enviromental changes :Nitrogen Depletion 2 h(1)
(c) 612. Brown enviromental changes :YPD stationary phase 4 h ypd-1(1)
(c) DES460 + 0.02% MMS - 5 min
(c) DES460 + 0.02% MMS - 15 min

GCN4 -*-> BAT1

(c) 6. Expression during the cell cycle (cdc15 arrest and release)(15)
(c) 11. Expression during diauxic shift: 9h,11h,13h,15h,17h,19h,21h(3)
(c) 89. Expression in response to 3-aminotriazole(1)
(c) 95. Expression in response to 50ug/mL FK506(1)
(c) 387. Rosetta 2000: Expression in cells with ERG11 under tet promoter(1)
(c) 401. Rosetta 2000: Expression in response to HU(1)
(c) 402. Rosetta 2000: Expression in response to Itraconazole(1)
(c) 403. Rosetta 2000: Expression in response to Lovastatin(1)
(c) 406. Rosetta 2000: Expression in response to Terbinafine(1)
(c) 407. Rosetta 2000: Expression in response to Tunicamycin(1)
(c) 446. Expression in response to 0.1% MMS for 10 min(1)
(c) 503. Brown enviromental changes :37C to 25C shock - 15 min(1)
(c) 504. Brown enviromental changes :37C to 25C shock - 30 min(1)
(c) 506. Brown enviromental changes :37C to 25C shock - 60 min(1)
(c) 507. Brown enviromental changes :37C to 25C shock - 90 min(1)
(c) 552. Brown enviromental changes :dtt 030 min dtt-2(1)
(c) 572. Brown enviromental changes :Hypo-osmotic shock - 5 min(1)
(c) 580. Brown enviromental changes :aa starv 2 h(1)
(c) 581. Brown enviromental changes :aa starv 4 h(1)
(c) 582. Brown enviromental changes :aa starv 6 h(1)
(c) 585. Brown enviromental changes :Nitrogen Depletion 2 h(1)
(c) 586. Brown enviromental changes :Nitrogen Depletion 4 h(1)
(c) 595. Brown enviromental changes :diauxic shift timecourse(1)
(c) 601. Brown enviromental changes :YPD 2 h ypd-2(1)
(c) 602. Brown enviromental changes :YPD 4 h ypd-2(1)
(c) 612. Brown enviromental changes :YPD stationary phase 4 h ypd-1(1)
(c) 613. Brown enviromental changes :YPD stationary phase 8 h ypd-1(1)
(c) 684. Expression in response to 0.8M NaCl for 10 min in wild type(1)
(c) DES460 + 0.02% MMS - 5 min
(c) 100 microM BCS 30 min

GCN4 -*-> ORT1

(c) 8. Expression during the cell cycle (cell size selection and release)(3)
(c) 89. Expression in response to 3-aminotriazole(1)
(c) 95. Expression in response to 50ug/mL FK506(1)
(c) 387. Rosetta 2000: Expression in cells with ERG11 under tet promoter(1)
(c) 395. Rosetta 2000: Expression in response to 2-deoxy-D-glucose(1)
(c) 402. Rosetta 2000: Expression in response to Itraconazole(1)
(c) 403. Rosetta 2000: Expression in response to Lovastatin(1)
(c) 406. Rosetta 2000: Expression in response to Terbinafine(1)
(c) 407. Rosetta 2000: Expression in response to Tunicamycin(1)
(c) PHO81c vs WT exp2(1)
(c) 429. Expression in strain YHE711 (wild type) in response to 30 min 50 nM treatment with rapamycin in YPD(1)
(c) 446. Expression in response to 0.1% MMS for 10 min(1)

(c) 447. Expression in response to 0.1% MMS for 30 min(1)
(c) 479. Expression in diploid cells in response to rapamycin (100nM) for: 15min,30min,90min,120min(2)
(c) 479. Expression in diploid cells in response to rapamycin (100nM) for: 15min,30min,90min,120min(3)
(c) 579. Brown enviromental changes :aa starv 1 h(1)
(c) 580. Brown enviromental changes :aa starv 2 h(1)
(c) 581. Brown enviromental changes :aa starv 4 h(1)
(c) 582. Brown enviromental changes :aa starv 6 h(1)
(c) 584. Brown enviromental changes :Nitrogen Depletion 1 h(1)
(c) 585. Brown enviromental changes :Nitrogen Depletion 2 h(1)
(c) 586. Brown enviromental changes :Nitrogen Depletion 4 h(1)
(c) 612. Brown enviromental changes :YPD stationary phase 4 h ypd-1(1)
(c) 684. Expression in response to 0.8M NaCl for 10 min in wild type(1)
(c) DES460 + 0.02% MMS - 5 min

GCN4 -*-> SRY1

(c) 89. Expression in response to 3-aminotriazole(1)
(c) 95. Expression in response to 50ug/mL FK506(1)
(c) 387. Rosetta 2000: Expression in cells with ERG11 under tet promoter(1)
(c) 392. Rosetta 2000: Expression in cells with PMA1 under tet promoter(1)
(c) 395. Rosetta 2000: Expression in response to 2-deoxy-D-glucose(1)
(c) 402. Rosetta 2000: Expression in response to Itraconazole(1)
(c) 403. Rosetta 2000: Expression in response to Lovastatin(1)
(c) 407. Rosetta 2000: Expression in response to Tunicamycin(1)
(c) 446. Expression in response to 0.1% MMS for 10 min(1)
(c) 452. Expression in response to low 4NQO (2 microgram/ml) for 60 min(1)
(c) 481. Expression in response to heat shock: 15,30,45,60,120 min(4)
(c) 523. Brown enviromental changes :constant 0.32 mM H2O2 (10 min) redo(1)
(c) 546. Brown enviromental changes :2.5mM DTT 060 min dtt-1(1)
(c) 572. Brown enviromental changes :Hypo-osmotic shock - 5 min(1)
(c) 579. Brown enviromental changes :aa starv 1 h(1)
(c) 580. Brown enviromental changes :aa starv 2 h(1)
(c) 581. Brown enviromental changes :aa starv 4 h(1)
(c) 582. Brown enviromental changes :aa starv 6 h(1)
(c) 583. Brown enviromental changes :Nitrogen Depletion 30 min.(1)
(c) 584. Brown enviromental changes :Nitrogen Depletion 1 h(1)
(c) 585. Brown enviromental changes :Nitrogen Depletion 2 h(1)
(c) 586. Brown enviromental changes :Nitrogen Depletion 4 h(1)
(c) 591. Brown enviromental changes :Nitrogen Depletion 3 d(1)
(c) 602. Brown enviromental changes :YPD 4 h ypd-2(1)
(c) wt_plus_gamma_60_min

GCN4 -*-> FCY2

(c) 6. Expression during the cell cycle (cdc15 arrest and release)(15)
(c) 11. Expression during diauxic shift: 9h,11h,13h,15h,17h,19h,21h(3)
(c) 89. Expression in response to 3-aminotriazole(1)
(c) 95. Expression in response to 50ug/mL FK506(1)
(c) 395. Rosetta 2000: Expression in response to 2-deoxy-D-glucose(1)
(c) 402. Rosetta 2000: Expression in response to Itraconazole(1)
(c) 407. Rosetta 2000: Expression in response to Tunicamycin(1)
(c) 479. Expression in diploid cells in response to rapamycin (100nM) for: 15min,30min,90min,120min(2)
(c) 538. Brown enviromental changes :1 mM Menadione (80 min) redo(1)
(c) 539. Brown enviromental changes :1 mM Menadione (105 min) redo(1)
(c) 540. Brown enviromental changes :1 mM Menadione (120 min)redo(1)
(c) 541. Brown enviromental changes :1 mM Menadione (160 min) redo(1)
(c) 551. Brown enviromental changes :dtt 015 min dtt-2(1)
(c) 552. Brown enviromental changes :dtt 030 min dtt-2(1)
(c) 556. Brown enviromental changes :dtt 480 min dtt-2(1)
(c) 572. Brown enviromental changes :Hypo-osmotic shock - 5 min(1)
(c) 573. Brown enviromental changes :Hypo-osmotic shock - 15 min(1)
(c) 580. Brown enviromental changes :aa starv 2 h(1)
(c) 581. Brown enviromental changes :aa starv 4 h(1)
(c) 582. Brown enviromental changes :aa starv 6 h(1)
(c) 585. Brown enviromental changes :Nitrogen Depletion 2 h(1)
(c) 586. Brown enviromental changes :Nitrogen Depletion 4 h(1)
(c) 595. Brown enviromental changes :diauxic shift timecourse(1)
(c) 602. Brown enviromental changes :YPD 4 h ypd-2(1)
(c) 611. Brown enviromental changes :YPD stationary phase 2 h ypd-1(1)
(c) 612. Brown enviromental changes :YPD stationary phase 4 h ypd-1(1)
(c) 613. Brown enviromental changes :YPD stationary phase 8 h ypd-1(1)
(c) 681. Expression in response to 0.4M NaCl for 10 min in wild type(1)
(c) 684. Expression in response to 0.8M NaCl for 10 min in wild type(1)
(c) DES460 + 0.02% MMS - 5 min
(c) DES460 (wt) - mock irradiation - 30 min
(c) 100 microM BCS 30 min

GCN4 -*-> FOL2

(c) 5. Expression during the cell cycle (alpha factor arrest and release)(11)
(c) 7. Expression during the cell Cycle (cdc28)(10)
(c) 89. Expression in response to 3-aminotriazole(1)
(c) 95. Expression in response to 50ug/mL FK506(1)
(c) 387. Rosetta 2000: Expression in cells with ERG11 under tet promoter(1)
(c) 401. Rosetta 2000: Expression in response to HU(1)
(c) 402. Rosetta 2000: Expression in response to Itraconazole(1)
(c) 403. Rosetta 2000: Expression in response to Lovastatin(1)
(c) 406. Rosetta 2000: Expression in response to Terbinafine(1)
(c) 407. Rosetta 2000: Expression in response to Tunicamycin(1)
(c) 446. Expression in response to 0.1% MMS for 10 min(1)
(c) 447. Expression in response to 0.1% MMS for 30 min(1)
(c) 448. Expression in response to 0.1% MMS for 60 min(1)
(c) 449. Expression in response to 0.1% MMS for 60 min(1)
(c) 479. Expression in diploid cells in response to rapamycin (100nM) for: 15min,30min,90min,120min(2)
(c) 479. Expression in diploid cells in response to rapamycin (100nM) for: 15min,30min,90min,120min(3)
(c) 504. Brown enviromental changes :37C to 25C shock - 30 min(1)
(c) 506. Brown enviromental changes :37C to 25C shock - 60 min(1)
(c) 523. Brown enviromental changes :constant 0.32 mM H2O2 (10 min) redo(1)
(c) 552. Brown enviromental changes :dtt 030 min dtt-2(1)
(c) 581. Brown enviromental changes :aa starv 4 h(1)
(c) 595. Brown enviromental changes :diauxic shift timecourse(1)
(c) 611. Brown enviromental changes :YPD stationary phase 2 h ypd-1(1)
(c) 612. Brown enviromental changes :YPD stationary phase 4 h ypd-1(1)
(c) 684. Expression in response to 0.8M NaCl for 10 min in wild type(1)
(c) DES460 + 0.02% MMS - 5 min
(c) DES460 + 0.02% MMS - 15 min

```
    (c) 100 microM BCS 30 min


GLN3 -*-> YHR029C


    (c) 5. Expression during the cell cycle (alpha factor arrest and release)(17)
    (c) 89. Expression in response to 3-aminotriazole(1)
    (c) 95. Expression in response to 50ug/mL FK506(1)
    (c) 387. Rosetta 2000: Expression in cells with ERG11 under tet promoter(1)
    (c) 402. Rosetta 2000: Expression in response to Itraconazole(1)
    (c) 406. Rosetta 2000: Expression in response to Terbinafine(1)
    (c) 407. Rosetta 2000: Expression in response to Tunicamycin(1)
    (c) 445. Expression in response to 0.1% MMS for 60 min (average of 3 experiments)(1)
    (c) 449. Expression in response to 0.1% MMS for 60 min(1)
    (c) 451. Expression in response to BCNU (200 micromolar) for 60 min(1)
    (c) 455. Expression in response to high MNNG (27 microgram/ml) for 60 min(1)
    (c) 456. Expression in response to high 4NQO (8 microgram/ml) for 60 min(1)
    (c) 464. Expression in response to 0.2% MMS for 60 min(1)
    (c) 479. Expression in diploid cells in response to rapamycin (100nM) for: 15min,30min,90min,120min(1)
    (c) 479. Expression in diploid cells in response to rapamycin (100nM) for: 15min,30min,90min,120min(2)
    (c) 479. Expression in diploid cells in response to rapamycin (100nM) for: 15min,30min,90min,120min(3)
    (c) 579. Brown enviromental changes :aa starv 1 h(1)
    (c) 581. Brown enviromental changes :aa starv 4 h(1)
    (c) 582. Brown enviromental changes :aa starv 6 h(1)
    (c) 584. Brown enviromental changes :Nitrogen Depletion 1 h(1)
    (c) 585. Brown enviromental changes :Nitrogen Depletion 2 h(1)
    (c) 586. Brown enviromental changes :Nitrogen Depletion 4 h(1)
    (c) 587. Brown enviromental changes :Nitrogen Depletion 8 h(1)
    (c) 588. Brown enviromental changes :Nitrogen Depletion 12 h(1)
    (c) 589. Brown enviromental changes :Nitrogen Depletion 1 d(1)
    (c) 590. Brown enviromental changes :Nitrogen Depletion 2 d(1)
    (c) 591. Brown enviromental changes :Nitrogen Depletion 3 d(1)
    (c) 592. Brown enviromental changes :Nitrogen Depletion 5 d(1)
    (c) 670. Expression in response to antimycin 60min(1)
    (c) 671. Expression in response to antimycin 120min(1)
    (c) 672. Expression in response to carbonyl cyanide m-chlorophenylhydrazone (CCCP) 90min(1)
    (c) DES460 (wt) - mock irradiation - 30 min


GLN3 -*-> ARG5,6


    (c) 6. Expression during the cell cycle (cdc15 arrest and release)(12)
    (c) 7. Expression during the cell Cycle (cdc28)(13)
    (c) 89. Expression in response to 3-aminotriazole(1)
    (c) 95. Expression in response to 50ug/mL FK506(1)
    (c) 387. Rosetta 2000: Expression in cells with ERG11 under tet promoter(1)
    (c) 389. Rosetta 2000: Expression in cells with HMG2 under tet promoter(1)
    (c) 402. Rosetta 2000: Expression in response to Itraconazole(1)
    (c) 403. Rosetta 2000: Expression in response to Lovastatin(1)
    (c) 406. Rosetta 2000: Expression in response to Terbinafine(1)
    (c) 407. Rosetta 2000: Expression in response to Tunicamycin(1)
    (c) 447. Expression in response to 0.1% MMS for 30 min(1)
    (c) 479. Expression in diploid cells in response to rapamycin (100nM) for: 15min,30min,90min,120min(1)
    (c) 481. Expression in response to heat shock: 15,30,45,60,120 min(4)
    (c) 487. Expression in response to sorbitol: 15 30 45 90 120 min(3)
    (c) 578. Brown enviromental changes :aa starv 0.5 h(1)
    (c) 579. Brown enviromental changes :aa starv 1 h(1)
    (c) 580. Brown enviromental changes :aa starv 2 h(1)
    (c) 581. Brown enviromental changes :aa starv 4 h(1)
    (c) 582. Brown enviromental changes :aa starv 6 h(1)
    (c) 583. Brown enviromental changes :Nitrogen Depletion 30 min.(1)
    (c) 584. Brown enviromental changes :Nitrogen Depletion 1 h(1)
    (c) 585. Brown enviromental changes :Nitrogen Depletion 2 h(1)
    (c) 586. Brown enviromental changes :Nitrogen Depletion 4 h(1)
    (c) 587. Brown enviromental changes :Nitrogen Depletion 8 h(1)
    (c) 588. Brown enviromental changes :Nitrogen Depletion 12 h(1)
    (c) 589. Brown enviromental changes :Nitrogen Depletion 1 d(1)
    (c) 590. Brown enviromental changes :Nitrogen Depletion 2 d(1)
    (c) 591. Brown enviromental changes :Nitrogen Depletion 3 d(1)
    (c) 592. Brown enviromental changes :Nitrogen Depletion 5 d(1)
    (c) 670. Expression in response to antimycin 60min(1)
    (c) 671. Expression in response to antimycin 120min(1)
    (c) DES460 + 0.02% MMS - 15 min
    (c) DES460 (wt) - mock irradiation - 30 min


GLN3 -*-> UGA3


    (c) 6. Expression during the cell cycle (cdc15 arrest and release)(12)
    (c) 7. Expression during the cell Cycle (cdc28)(13)
    (c) 89. Expression in response to 3-aminotriazole(1)
    (c) 95. Expression in response to 50ug/mL FK506(1)
    (c) 387. Rosetta 2000: Expression in cells with ERG11 under tet promoter(1)
    (c) 389. Rosetta 2000: Expression in cells with HMG2 under tet promoter(1)
    (c) 402. Rosetta 2000: Expression in response to Itraconazole(1)
    (c) 403. Rosetta 2000: Expression in response to Lovastatin(1)
    (c) 406. Rosetta 2000: Expression in response to Terbinafine(1)
    (c) 407. Rosetta 2000: Expression in response to Tunicamycin(1)
    (c) 447. Expression in response to 0.1% MMS for 30 min(1)
    (c) 479. Expression in diploid cells in response to rapamycin (100nM) for: 15min,30min,90min,120min(1)
    (c) 481. Expression in response to heat shock: 15,30,45,60,120 min(4)
    (c) 487. Expression in response to sorbitol: 15 30 45 90 120 min(3)
    (c) 578. Brown enviromental changes :aa starv 0.5 h(1)
    (c) 579. Brown enviromental changes :aa starv 1 h(1)
    (c) 580. Brown enviromental changes :aa starv 2 h(1)
    (c) 581. Brown enviromental changes :aa starv 4 h(1)
    (c) 582. Brown enviromental changes :aa starv 6 h(1)
    (c) 583. Brown enviromental changes :Nitrogen Depletion 30 min.(1)
    (c) 584. Brown enviromental changes :Nitrogen Depletion 1 h(1)
    (c) 585. Brown enviromental changes :Nitrogen Depletion 2 h(1)
    (c) 586. Brown enviromental changes :Nitrogen Depletion 4 h(1)
    (c) 587. Brown enviromental changes :Nitrogen Depletion 8 h(1)
    (c) 588. Brown enviromental changes :Nitrogen Depletion 12 h(1)
    (c) 589. Brown enviromental changes :Nitrogen Depletion 1 d(1)
    (c) 590. Brown enviromental changes :Nitrogen Depletion 2 d(1)
    (c) 591. Brown enviromental changes :Nitrogen Depletion 3 d(1)
    (c) 592. Brown enviromental changes :Nitrogen Depletion 5 d(1)
    (c) 670. Expression in response to antimycin 60min(1)
    (c) 671. Expression in response to antimycin 120min(1)
```

```
    (c) DES460 + 0.02% MMS - 15 min
    (c) DES460 (wt) - mock irradiation - 30 min


GLN3 -*-> MET16


    (c) 89. Expression in response to 3-aminotriazole(1)
    (c) 95. Expression in response to 50ug/mL FK506(1)
    (c) 387. Rosetta 2000: Expression in cells with ERG11 under tet promoter(1)
    (c) 392. Rosetta 2000: Expression in cells with PMA1 under tet promoter(1)
    (c) 402. Rosetta 2000: Expression in response to Itraconazole(1)
    (c) 406. Rosetta 2000: Expression in response to Terbinafine(1)
    (c) 407. Rosetta 2000: Expression in response to Tunicamycin(1)
    (c) 428. Expression in strain PM38 (wild type) in response to 30 min 50 nM treatment with rapamycin in YPD(1)
    (c) 429. Expression in strain YHE711 (wild type) in response to 30 min 50 nM treatment with rapamycin in YPD(1)
    (c) 442. Expression in strain PM38 (wild type) in response to 30 min 50 nM treatment with rapamycin in YPD(1)
    (c) 578. Brown enviromental changes :aa starv 0.5 h(1)
    (c) 579. Brown enviromental changes :aa starv 1 h(1)
    (c) 580. Brown enviromental changes :aa starv 2 h(1)
    (c) 581. Brown enviromental changes :aa starv 4 h(1)
    (c) 582. Brown enviromental changes :aa starv 6 h(1)
    (c) 583. Brown enviromental changes :Nitrogen Depletion 30 min.(1)
    (c) 584. Brown enviromental changes :Nitrogen Depletion 1 h(1)
    (c) 585. Brown enviromental changes :Nitrogen Depletion 2 h(1)
    (c) 586. Brown enviromental changes :Nitrogen Depletion 4 h(1)
    (c) 587. Brown enviromental changes :Nitrogen Depletion 8 h(1)
    (c) 588. Brown enviromental changes :Nitrogen Depletion 12 h(1)
    (c) 589. Brown enviromental changes :Nitrogen Depletion 1 d(1)
    (c) 590. Brown enviromental changes :Nitrogen Depletion 2 d(1)
    (c) 591. Brown enviromental changes :Nitrogen Depletion 3 d(1)
    (c) 592. Brown enviromental changes :Nitrogen Depletion 5 d(1)
    (c) 670. Expression in response to antimycin 60min(1)
    (c) 671. Expression in response to antimycin 120min(1)
    (c) DES460 + 0.02% MMS - 15 min
    (c) DES460 (wt) - mock irradiation - 30 min


GLN3 -*-> LYS20


    (c) 89. Expression in response to 3-aminotriazole(1)
    (c) 95. Expression in response to 50ug/mL FK506(1)
    (c) 387. Rosetta 2000: Expression in cells with ERG11 under tet promoter(1)
    (c) 395. Rosetta 2000: Expression in response to 2-deoxy-D-glucose(1)
    (c) 401. Rosetta 2000: Expression in response to HU(1)
    (c) 402. Rosetta 2000: Expression in response to Itraconazole(1)
    (c) 406. Rosetta 2000: Expression in response to Terbinafine(1)
    (c) 407. Rosetta 2000: Expression in response to Tunicamycin(1)
    (c) 439. Expression in strain Jk9-3da (wild type) in response to 30 min 50 nM treatment with rapamycin in YPD(1)
    (c) 479. Expression in diploid cells in response to rapamycin (100nM) for: 15min,30min,90min,120min(1)
    (c) 479. Expression in diploid cells in response to rapamycin (100nM) for: 15min,30min,90min,120min(2)
    (c) 556. Brown enviromental changes :dtt 480 min dtt-2(1)
    (c) 578. Brown enviromental changes :aa starv 0.5 h(1)
    (c) 579. Brown enviromental changes :aa starv 1 h(1)
    (c) 580. Brown enviromental changes :aa starv 2 h(1)
    (c) 581. Brown enviromental changes :aa starv 4 h(1)
    (c) 582. Brown enviromental changes :aa starv 6 h(1)
    (c) 583. Brown enviromental changes :Nitrogen Depletion 30 min.(1)
    (c) 584. Brown enviromental changes :Nitrogen Depletion 1 h(1)
    (c) 585. Brown enviromental changes :Nitrogen Depletion 2 h(1)
    (c) 586. Brown enviromental changes :Nitrogen Depletion 4 h(1)
    (c) 670. Expression in response to antimycin 60min(1)
    (c) 671. Expression in response to antimycin 120min(1)
    (c) 672. Expression in response to carbonyl cyanide m-chlorophenylhydrazone (CCCP) 90min(1)
    (c) 675. Expression in response to propionate(1)
    (c) DES460 + 0.02% MMS - 15 min


GLN3 -*-> MEP1


    (c) 89. Expression in response to 3-aminotriazole(1)
    (c) 95. Expression in response to 50ug/mL FK506(1)
    (c) 387. Rosetta 2000: Expression in cells with ERG11 under tet promoter(1)
    (c) 395. Rosetta 2000: Expression in response to 2-deoxy-D-glucose(1)
    (c) 401. Rosetta 2000: Expression in response to HU(1)
    (c) 402. Rosetta 2000: Expression in response to Itraconazole(1)
    (c) 406. Rosetta 2000: Expression in response to Terbinafine(1)
    (c) 407. Rosetta 2000: Expression in response to Tunicamycin(1)
    (c) 439. Expression in strain Jk9-3da (wild type) in response to 30 min 50 nM treatment with rapamycin in YPD(1)
    (c) 479. Expression in diploid cells in response to rapamycin (100nM) for: 15min,30min,90min,120min(1)
    (c) 479. Expression in diploid cells in response to rapamycin (100nM) for: 15min,30min,90min,120min(2)
    (c) 556. Brown enviromental changes :dtt 480 min dtt-2(1)
    (c) 578. Brown enviromental changes :aa starv 0.5 h(1)
    (c) 579. Brown enviromental changes :aa starv 1 h(1)
    (c) 580. Brown enviromental changes :aa starv 2 h(1)
    (c) 581. Brown enviromental changes :aa starv 4 h(1)
    (c) 582. Brown enviromental changes :aa starv 6 h(1)
    (c) 583. Brown enviromental changes :Nitrogen Depletion 30 min.(1)
    (c) 584. Brown enviromental changes :Nitrogen Depletion 1 h(1)
    (c) 585. Brown enviromental changes :Nitrogen Depletion 2 h(1)
    (c) 586. Brown enviromental changes :Nitrogen Depletion 4 h(1)
    (c) 670. Expression in response to antimycin 60min(1)
    (c) 671. Expression in response to antimycin 120min(1)
    (c) 672. Expression in response to carbonyl cyanide m-chlorophenylhydrazone (CCCP) 90min(1)
    (c) 675. Expression in response to propionate(1)
    (c) DES460 + 0.02% MMS - 15 min


GLN3 -*-> SRY1


    (c) 89. Expression in response to 3-aminotriazole(1)
    (c) 95. Expression in response to 50ug/mL FK506(1)
    (c) 387. Rosetta 2000: Expression in cells with ERG11 under tet promoter(1)
    (c) 395. Rosetta 2000: Expression in response to 2-deoxy-D-glucose(1)
    (c) 401. Rosetta 2000: Expression in response to HU(1)
    (c) 402. Rosetta 2000: Expression in response to Itraconazole(1)
    (c) 406. Rosetta 2000: Expression in response to Terbinafine(1)
    (c) 407. Rosetta 2000: Expression in response to Tunicamycin(1)
    (c) 439. Expression in strain Jk9-3da (wild type) in response to 30 min 50 nM treatment with rapamycin in YPD(1)
```

(c) 479. Expression in diploid cells in response to rapamycin (100nM) for: 15min,30min,90min,120min(1)
(c) 479. Expression in diploid cells in response to rapamycin (100nM) for: 15min,30min,90min,120min(2)
(c) 556. Brown enviromental changes :dtt 480 min dtt-2(1)
(c) 578. Brown enviromental changes :aa starv 0.5 h(1)
(c) 579. Brown enviromental changes :aa starv 1 h(1)
(c) 580. Brown enviromental changes :aa starv 2 h(1)
(c) 581. Brown enviromental changes :aa starv 4 h(1)
(c) 582. Brown enviromental changes :aa starv 6 h(1)
(c) 583. Brown enviromental changes :Nitrogen Depletion 30 min.(1)
(c) 584. Brown enviromental changes :Nitrogen Depletion 1 h(1)
(c) 585. Brown enviromental changes :Nitrogen Depletion 2 h(1)
(c) 586. Brown enviromental changes :Nitrogen Depletion 4 h(1)
(c) 670. Expression in response to antimycin 60min(1)
(c) 671. Expression in response to antimycin 120min(1)
(c) 672. Expression in response to carbonyl cyanide m-chlorophenylhydrazone (CCCP) 90min(1)
(c) 675. Expression in response to propionate(1)
(c) DES460 + 0.02% MMS - 15 min

GLN3 -*-> ARG1

(c) 6. Expression during the cell cycle (cdc15 arrest and release)(22)
(c) 6. Expression during the cell cycle (cdc15 arrest and release)(23)
(c) 89. Expression in response to 3-aminotriazole(1)
(c) 95. Expression in response to 50ug/mL FK506(1)
(c) 387. Rosetta 2000: Expression in cells with ERG11 under tet promoter(1)
(c) 402. Rosetta 2000: Expression in response to Itraconazole(1)
(c) 429. Expression in strain YHE711 (wild type) in response to 30 min 50 nM treatment with rapamycin in YPD(1)
(c) 439. Expression in strain Jk9-3da (wild type) in response to 30 min 50 nM treatment with rapamycin in YPD(1)
(c) 479. Expression in diploid cells in response to rapamycin (100nM) for: 15min,30min,90min,120min(1)
(c) 487. Expression in response to sorbitol: 15 30 45 90 120 min(3)
(c) 572. Brown enviromental changes :Hypo-osmotic shock - 5 min(1)
(c) 579. Brown enviromental changes :aa starv 1 h(1)
(c) 580. Brown enviromental changes :aa starv 2 h(1)
(c) 581. Brown enviromental changes :aa starv 4 h(1)
(c) 582. Brown enviromental changes :aa starv 6 h(1)
(c) 583. Brown enviromental changes :Nitrogen Depletion 30 min.(1)
(c) 584. Brown enviromental changes :Nitrogen Depletion 1 h(1)
(c) 585. Brown enviromental changes :Nitrogen Depletion 2 h(1)
(c) 586. Brown enviromental changes :Nitrogen Depletion 4 h(1)
(c) 670. Expression in response to antimycin 60min(1)
(c) 671. Expression in response to antimycin 120min(1)
(c) 672. Expression in response to carbonyl cyanide m-chlorophenylhydrazone (CCCP) 90min(1)

GLN3 -*-> YBR043C

(c) 5. Expression during the cell cycle (alpha factor arrest and release)(16)
(c) 6. Expression during the cell cycle (cdc15 arrest and release)(8)
(c) 6. Expression during the cell cycle (cdc15 arrest and release)(10)
(c) 7. Expression during the cell Cycle (cdc28)(10)
(c) 11. Expression during diauxic shift: 9h,11h,13h,15h,17h,19h,21h(2)
(c) 89. Expression in response to 3-aminotriazole(1)
(c) 95. Expression in response to 50ug/mL FK506(1)
(c) 387. Rosetta 2000: Expression in cells with ERG11 under tet promoter(1)
(c) 395. Rosetta 2000: Expression in response to 2-deoxy-D-glucose(1)
(c) 402. Rosetta 2000: Expression in response to Itraconazole(1)
(c) 403. Rosetta 2000: Expression in response to Lovastatin(1)
(c) 406. Rosetta 2000: Expression in response to Terbinafine(1)
(c) 407. Rosetta 2000: Expression in response to Tunicamycin(1)
(c) 579. Brown enviromental changes :aa starv 1 h(1)
(c) 580. Brown enviromental changes :aa starv 2 h(1)
(c) 581. Brown enviromental changes :aa starv 4 h(1)
(c) 585. Brown enviromental changes :Nitrogen Depletion 2 h(1)
(c) 586. Brown enviromental changes :Nitrogen Depletion 4 h(1)
(c) 588. Brown enviromental changes :Nitrogen Depletion 12 h(1)
(c) 591. Brown enviromental changes :Nitrogen Depletion 3 d(1)
(c) 594. Brown enviromental changes :diauxic shift timecourse(1)
(c) 670. Expression in response to antimycin 60min(1)
(c) 671. Expression in response to antimycin 120min(1)
(c) 672. Expression in response to carbonyl cyanide m-chlorophenylhydrazone (CCCP) 90min(1)
(c) 675. Expression in response to propionate(1)
(c) DES460 (wt) - mock irradiation - 30 min
(c) DES460 (wt) - mock irradiation - 90 min

HAP2 -*-> ASN2

(c) 5. Expression during the cell cycle (alpha factor arrest and release)(17)
(c) 6. Expression during the cell cycle (cdc15 arrest and release)(16)
(c) 8. Expression during the cell cycle (cell size selection and release)(9)
(c) 89. Expression in response to 3-aminotriazole(1)
(c) 95. Expression in response to 50ug/mL FK506(1)
(c) 391. Rosetta 2000: Expression in cells with KAR2 under tet promoter(1)
(c) 393. Rosetta 2000: Expression in cells with RHO1 under tet promoter(1)
(c) 395. Rosetta 2000: Expression in response to 2-deoxy-D-glucose(1)
(c) 400. Rosetta 2000: Expression in response to Glucosamine(1)
(c) 402. Rosetta 2000: Expression in response to Itraconazole(1)
(c) 406. Rosetta 2000: Expression in response to Terbinafine(1)
(c) 445. Expression in response to 0.1% MMS for 60 min (average of 3 experiments)(1)
(c) 446. Expression in response to 0.1% MMS for 10 min(1)
(c) 447. Expression in response to 0.1% MMS for 30 min(1)
(c) 448. Expression in response to 0.1% MMS for 60 min(1)
(c) 449. Expression in response to 0.1% MMS for 60 min(1)
(c) 462. Expression in response to 0.05% MMS for 60 min(1)
(c) 479. Expression in diploid cells in response to rapamycin (100nM) for: 15min,30min,90min,120min(1)
(c) 479. Expression in diploid cells in response to rapamycin (100nM) for: 15min,30min,90min,120min(2)
(c) 486. Expression in response to NaCl: 15 30 45 60 120 min(3)
(c) 635. Brown enviromental changes :YAP1 overexpression(1)
(c) (Var.) Rich Media 2% Glucose YPD-Average wt 5mM aF, 30 min.
(c) DES460 + 0.02% MMS - 5 min
(c) MHY1 (crt1) vs CRY1 (wild type)
(c) 100 microM BCS 30 min

HAP4 -*-> SDH2

(c) 6. Expression during the cell cycle (cdc15 arrest and release)(15)

(c) 11. Expression during diauxic shift: 9h,11h,13h,15h,17h,19h,21h(4)
(c) 11. Expression during diauxic shift: 9h,11h,13h,15h,17h,19h,21h(5)
(c) 11. Expression during diauxic shift: 9h,11h,13h,15h,17h,19h,21h(6)
(c) 11. Expression during diauxic shift: 9h,11h,13h,15h,17h,19h,21h(7)
(c) 388. Rosetta 2000: Expression in cells with FKS1 under tet promoter(1)
(c) 390. Rosetta 2000: Expression in cells with IDI1 under tet promoter(1)
(c) 482. Expression in response to acid: 10,20,40,60,80,100 min(1)
(c) 482. Expression in response to acid: 10,20,40,60,80,100 min(5)
(c) 485. Expression in response to peroxide: 10,20,40,60,120 min(1)
(c) 485. Expression in response to peroxide: 10,20,40,60,120 min(5)
(c) 488. Brown enviromental changes :Heat Shock 05 minutes hs-1(1)
(c) 491. Brown enviromental changes :Heat Shock 20 minutes hs-1(1)
(c) 509. Brown enviromental changes :heat shock 21 to 37, 20 minutes(1)
(c) 557. Brown enviromental changes :1.5 mM diamide (5 min)(1)
(c) 568. Brown enviromental changes :1M sorbitol - 45 min (1)
(c) 596. Brown enviromental changes :diauxic shift timecourse(1)
(c) 597. Brown enviromental changes :diauxic shift timecourse(1)
(c) 598. Brown enviromental changes :diauxic shift timecourse(1)
(c) 599. Brown enviromental changes :diauxic shift timecourse(1)
(c) 600. Brown enviromental changes :diauxic shift timecourse(1)
(c) 602. Brown enviromental changes :YPD 4 h ypd-2(1)
(c) 603. Brown enviromental changes :YPD 6 h ypd-2(1)
(c) 604. Brown enviromental changes :YPD 8 h ypd-2(1)
(c) 605. Brown enviromental changes :YPD 10 h ypd-2(1)
(c) 606. Brown enviromental changes :YPD 12 h ypd-2(1)
(c) 607. Brown enviromental changes :YPD 1 d ypd-2(1)
(c) 608. Brown enviromental changes :YPD 2 d ypd-2(1)
(c) 611. Brown enviromental changes :YPD stationary phase 2 h ypd-1(1)
(c) 612. Brown enviromental changes :YPD stationary phase 4 h ypd-1(1)
(c) 613. Brown enviromental changes :YPD stationary phase 8 h ypd-1(1)
(c) 614. Brown enviromental changes :YPD stationary phase 12 h ypd-1(1)
(c) 615. Brown enviromental changes :YPD stationary phase 1 d ypd-1(1)
(c) 616. Brown enviromental changes :YPD stationary phase 2 d ypd-1(1)
(c) 617. Brown enviromental changes :YPD stationary phase 3 d ypd-1(1)

MOT3 -*-> AAR2

(c) 2. Cell Cycle: Expression in response to Cln3p (set 2)(1)
(c) 5. Expression during the cell cycle (alpha factor arrest and release)(8)
(c) 5. Expression during the cell cycle (alpha factor arrest and release)(16)
(c) 7. Expression during the cell Cycle (cdc28)(8)
(c) 7. Expression during the cell Cycle (cdc28)(15)
(c) 7. Expression during the cell Cycle (cdc28)(16)
(c) 7. Expression during the cell Cycle (cdc28)(17)
(c) 8. Expression during the cell cycle (cell size selection and release)(4)
(c) 11. Expression during diauxic shift: 9h,11h,13h,15h,17h,19h,21h(1)
(c) 49. Expression in response to 50 nM alpha-factor: 0,15,30,45,60,90,120 min(1)
(c) 56. Expression in response to overproduction of Ste12p(1)
(c) 477. Expression in response to trichostatin A (TSA): 15min,30min,60min,120min(1)
(c) 477. Expression in response to trichostatin A (TSA): 15min,30min,60min,120min(3)
(c) 482. Expression in response to acid: 10,20,40,60,80,100 min(5)
(c) 486. Expression in response to NaCl: 15 30 45 60 120 min(4)
(c) 526. Brown enviromental changes :constant 0.32 mM H2O2 (40 min) rescan(1)
(c) 555. Brown enviromental changes :dtt 240 min dtt-2(1)
(c) 593. Brown enviromental changes :Diauxic Shift Timecourse(1)

MOT3 -*-> KRE25

(c) 2. Cell Cycle: Expression in response to Cln3p (set 2)(1)
(c) 5. Expression during the cell cycle (alpha factor arrest and release)(8)
(c) 5. Expression during the cell cycle (alpha factor arrest and release)(16)
(c) 7. Expression during the cell Cycle (cdc28)(8)
(c) 7. Expression during the cell Cycle (cdc28)(15)
(c) 7. Expression during the cell Cycle (cdc28)(16)
(c) 7. Expression during the cell Cycle (cdc28)(17)
(c) 8. Expression during the cell cycle (cell size selection and release)(4)
(c) 11. Expression during diauxic shift: 9h,11h,13h,15h,17h,19h,21h(1)
(c) 49. Expression in response to 50 nM alpha-factor: 0,15,30,45,60,90,120 min(1)
(c) 56. Expression in response to overproduction of Ste12p(1)
(c) 477. Expression in response to trichostatin A (TSA): 15min,30min,60min,120min(1)
(c) 477. Expression in response to trichostatin A (TSA): 15min,30min,60min,120min(3)
(c) 482. Expression in response to acid: 10,20,40,60,80,100 min(5)
(c) 486. Expression in response to NaCl: 15 30 45 60 120 min(4)
(c) 526. Brown enviromental changes :constant 0.32 mM H2O2 (40 min) rescan(1)
(c) 555. Brown enviromental changes :dtt 240 min dtt-2(1)
(c) 593. Brown enviromental changes :Diauxic Shift Timecourse(1)

MOT3 -*-> YDR219C

(c) 5. Expression during the cell cycle (alpha factor arrest and release)(15)
(c) 6. Expression during the cell cycle (cdc15 arrest and release)(11)
(c) 7. Expression during the cell Cycle (cdc28)(8)
(c) 7. Expression during the cell Cycle (cdc28)(15)
(c) 49. Expression in response to 50 nM alpha-factor: 0,15,30,45,60,90,120 min(1)
(c) 446. Expression in response to 0.1% MMS for 10 min(1)
(c) 447. Expression in response to 0.1% MMS for 30 min(1)
(c) 448. Expression in response to 0.1% MMS for 60 min(1)
(c) 449. Expression in response to 0.1% MMS for 60 min(1)
(c) 477. Expression in response to trichostatin A (TSA): 15min,30min,60min,120min(1)
(c) 487. Expression in response to sorbitol: 15 30 45 90 120 min(2)
(c) 526. Brown enviromental changes :constant 0.32 mM H2O2 (40 min) rescan(1)
(c) wt_plus_gamma_30_min

MOT3 -*-> YMR210W

(c) 5. Expression during the cell cycle (alpha factor arrest and release)(15)
(c) 6. Expression during the cell cycle (cdc15 arrest and release)(11)
(c) 7. Expression during the cell Cycle (cdc28)(8)
(c) 7. Expression during the cell Cycle (cdc28)(15)
(c) 49. Expression in response to 50 nM alpha-factor: 0,15,30,45,60,90,120 min(1)
(c) 446. Expression in response to 0.1% MMS for 10 min(1)
(c) 447. Expression in response to 0.1% MMS for 30 min(1)
(c) 448. Expression in response to 0.1% MMS for 60 min(1)
(c) 449. Expression in response to 0.1% MMS for 60 min(1)

(c) 477. Expression in response to trichostatin A (TSA): 15min,30min,60min,120min(1)
(c) 487. Expression in response to sorbitol: 15 30 45 90 120 min(2)
(c) 526. Brown enviromental changes :constant 0.32 mM H2O2 (40 min) rescan(1)
(c) wt_plus_gamma_30_min

MSN2 -*-> RFX1

(c) 4. Cell Cycle: Expression in response to Clb2p (set 2, 30 min)(1)
(c) 5. Expression during the cell cycle (alpha factor arrest and release)(3)
(c) 5. Expression during the cell cycle (alpha factor arrest and release)(5)
(c) 6. Expression during the cell cycle (cdc15 arrest and release)(15)
(c) 6. Expression during the cell cycle (cdc15 arrest and release)(16)
(c) 6. Expression during the cell cycle (cdc15 arrest and release)(18)
(c) 8. Expression during the cell cycle (cell size selection and release)(1)
(c) 73. Expression in response to overproduction of activated Pkc1p(1)
(c) 464. Expression in response to 0.2% MMS for 60 min(1)
(c) 488. Brown enviromental changes :Heat Shock 05 minutes hs-1(1)
(c) 499. Brown enviromental changes :Heat Shock 005 minutes hs-2(1)
(c) 557. Brown enviromental changes :1.5 mM diamide (5 min)(1)
(c) 565. Brown enviromental changes :1M sorbitol - 5 min(1)
(c) 633. Brown enviromental changes :MSN2 overexpression (repeat)(1)
(c) 634. Brown enviromental changes :MSN4 overexpression(1)
(c) 681. Expression in response to 0.4M NaCl for 10 min in wild type(1)
(c) (Var.) Rich Media 2% Glucose YPD-Average wt 5mM aF, 30 min.
(c) DES460 (wt) - mock irradiation - 30 min
(c) wt-gal

MSN4 -*-> TPS2

(c) 401. Rosetta 2000: Expression in response to HU(1)
(c) 462. Expression in response to 0.05% MMS for 60 min(1)
(c) 463. Expression in response to 0.1% MMS for 60 min(1)
(c) 477. Expression in response to trichostatin A (TSA): 15min,30min,60min,120min(1)
(c) 477. Expression in response to trichostatin A (TSA): 15min,30min,60min,120min(2)
(c) 477. Expression in response to trichostatin A (TSA): 15min,30min,60min,120min(3)
(c) 477. Expression in response to trichostatin A (TSA): 15min,30min,60min,120min(4)
(c) 481. Expression in response to heat shock: 15,30,45,60,120 min(1)
(c) 481. Expression in response to heat shock: 15,30,45,60,120 min(2)
(c) 482. Expression in response to acid: 10,20,40,60,80,100 min(1)
(c) 482. Expression in response to acid: 10,20,40,60,80,100 min(2)
(c) 482. Expression in response to acid: 10,20,40,60,80,100 min(3)
(c) 482. Expression in response to acid: 10,20,40,60,80,100 min(4)
(c) 482. Expression in response to acid: 10,20,40,60,80,100 min(5)
(c) 482. Expression in response to acid: 10,20,40,60,80,100 min(6)
(c) 487. Expression in response to sorbitol: 15 30 45 90 120 min(3)
(c) 487. Expression in response to sorbitol: 15 30 45 90 120 min(5)
(c) 565. Brown enviromental changes :1M sorbitol - 5 min(1)
(c) 566. Brown enviromental changes :1M sorbitol - 15 min(1)
(c) 567. Brown enviromental changes :1M sorbitol - 30 min(1)
(c) 568. Brown enviromental changes :1M sorbitol - 45 min (1)
(c) 570. Brown enviromental changes :1M sorbitol - 90 min(1)
(c) 571. Brown enviromental changes :1M sorbitol - 120 min(1)
(c) 612. Brown enviromental changes :YPD stationary phase 4 h ypd-1(1)
(c) 613. Brown enviromental changes :YPD stationary phase 8 h ypd-1(1)
(c) Addition of 1M NaCl (90')

PHO2 -*-> FTH1

(c) 6. Expression during the cell cycle (cdc15 arrest and release)(15)
(c) 6. Expression during the cell cycle (cdc15 arrest and release)(20)
(c) 8. Expression during the cell cycle (cell size selection and release)(9)
(c) 11. Expression during diauxic shift: 9h,11h,13h,15h,17h,19h,21h(2)
(c) 89. Expression in response to 3-aminotriazole(1)
(c) 95. Expression in response to 50ug/mL FK506(1)
(c) 402. Rosetta 2000: Expression in response to Itraconazole(1)
(c) 445. Expression in response to 0.1% MMS for 60 min (average of 3 experiments)(1)
(c) 446. Expression in response to 0.1% MMS for 10 min(1)
(c) 447. Expression in response to 0.1% MMS for 30 min(1)
(c) 448. Expression in response to 0.1% MMS for 60 min(1)
(c) 463. Expression in response to 0.1% MMS for 60 min(1)
(c) 464. Expression in response to 0.2% MMS for 60 min(1)
(c) 479. Expression in diploid cells in response to rapamycin (100nM) for: 15min,30min,90min,120min(3)
(c) 515. Brown enviromental changes :29C to 33C - 30 minutes(1)
(c) 535. Brown enviromental changes :1 mM Menadione (30 min) redo(1)
(c) 538. Brown enviromental changes :1 mM Menadione (80 min) redo(1)
(c) 571. Brown enviromental changes :1M sorbitol - 120 min(1)
(c) 572. Brown enviromental changes :Hypo-osmotic shock - 5 min(1)
(c) 594. Brown enviromental changes :diauxic shift timecourse(1)
(c) 601. Brown enviromental changes :YPD 2 h ypd-2(1)
(c) 612. Brown enviromental changes :YPD stationary phase 4 h ypd-1(1)
(c) DES460 + 0.02% MMS - 15 min
(c) DES460 + 0.02% MMS - 30 min
(c) wt_plus_gamma_120_min
(c) DES460 (wt) - mock irradiation - 30 min
(c) DES460 (wt) - mock irradiation - 60 min
(c) 100 microM BCS 30 min

PHO4 -*-> ADE3

(c) 5. Expression during the cell cycle (alpha factor arrest and release)(12)
(c) 5. Expression during the cell cycle (alpha factor arrest and release)(13)
(c) 5. Expression during the cell cycle (alpha factor arrest and release)(15)
(c) 6. Expression during the cell cycle (cdc15 arrest and release)(12)
(c) 89. Expression in response to 3-aminotriazole(1)
(c) 95. Expression in response to 50ug/mL FK506(1)
(c) 390. Rosetta 2000: Expression in cells with IDI1 under tet promoter(1)
(c) 393. Rosetta 2000: Expression in cells with RHO1 under tet promoter(1)
(c) 406. Rosetta 2000: Expression in response to Terbinafine(1)
(c) PHO4c vs WT(1)
(c) pho80 vs WT(1)
(c) PHO81c vs WT exp1(1)
(c) 481. Expression in response to heat shock: 15,30,45,60,120 min(4)
(c) 493. Brown enviromental changes :Heat Shock 40 minutes hs-1(1)
(c) 494. Brown enviromental changes :Heat Shock 60 minutes hs-1(1)

(c) 519. Brown enviromental changes :29C +1M sorbitol to 33C + 1M sorbitol - 30 minutes(1)
(c) 544. Brown enviromental changes :2.5mM DTT 030 min dtt-1(1)
(c) 579. Brown enviromental changes :aa starv 1 h(1)
(c) 581. Brown enviromental changes :aa starv 4 h(1)
(c) Addition of 1M NaCl (10')
(c) Addition of 1M NaCl (30')
(c) 100 microM CuSO4 30 min
(c) MAC1-up (C)

RCS1 -*-> SIT1

(c) 5. Expression during the cell cycle (alpha factor arrest and release)(11)
(c) 5. Expression during the cell cycle (alpha factor arrest and release)(13)
(c) 5. Expression during the cell cycle (alpha factor arrest and release)(15)
(c) 5. Expression during the cell cycle (alpha factor arrest and release)(17)
(c) 6. Expression during the cell cycle (cdc15 arrest and release)(7)
(c) 6. Expression during the cell cycle (cdc15 arrest and release)(8)
(c) 6. Expression during the cell cycle (cdc15 arrest and release)(10)
(c) 6. Expression during the cell cycle (cdc15 arrest and release)(16)
(c) 6. Expression during the cell cycle (cdc15 arrest and release)(17)
(c) 6. Expression during the cell cycle (cdc15 arrest and release)(18)
(c) 7. Expression during the cell Cycle (cdc28)(13)
(c) 7. Expression during the cell Cycle (cdc28)(14)
(c) 7. Expression during the cell Cycle (cdc28)(16)
(c) 7. Expression during the cell Cycle (cdc28)(17)
(c) 406. Rosetta 2000: Expression in response to Terbinafine(1)
(c) 446. Expression in response to 0.1% MMS for 10 min(1)
(c) 483. Expression in response to alkali: 10,20,40,60,80,100 min(1)
(c) 483. Expression in response to alkali: 10,20,40,60,80,100 min(6)
(c) 487. Expression in response to sorbitol: 15 30 45 90 120 min(3)
(c) 575. Brown enviromental changes :Hypo-osmotic shock - 45 min(1)
(c) DES460 + 0.02% MMS - 15 min
(c) wt_plus_gamma_20_min
(c) DES460 (wt) - mock irradiation - 30 min
(c) 100 microM CuSO4 30 min
(c) 100 microM CuSO4 30 min (B)

ROX1 -*-> YGR223C

(c) 394. Rosetta 2000: Expression in cells with YEF3 under tet promoter(1)
(c) 445. Expression in response to 0.1% MMS for 60 min (average of 3 experiments)(1)
(c) 446. Expression in response to 0.1% MMS for 10 min(1)
(c) 447. Expression in response to 0.1% MMS for 30 min(1)
(c) 448. Expression in response to 0.1% MMS for 60 min(1)
(c) 449. Expression in response to 0.1% MMS for 60 min(1)
(c) 462. Expression in response to 0.05% MMS for 60 min(1)
(c) 463. Expression in response to 0.1% MMS for 60 min(1)
(c) 464. Expression in response to 0.2% MMS for 60 min(1)
(c) 483. Expression in response to alkali: 10,20,40,60,80,100 min(2)
(c) 485. Expression in response to peroxide: 10,20,40,60,120 min(1)
(c) 485. Expression in response to peroxide: 10,20,40,60,120 min(2)
(c) 485. Expression in response to peroxide: 10,20,40,60,120 min(3)
(c) 485. Expression in response to peroxide: 10,20,40,60,120 min(4)
(c) 525. Brown enviromental changes :constant 0.32 mM H2O2 (30 min) redo(1)
(c) 534. Brown enviromental changes :1 mM Menadione (20 min) redo(1)
(c) 535. Brown enviromental changes :1 mM Menadione (30 min) redo(1)
(c) 536. Brown enviromental changes :1mM Menadione (40 min) redo(1)
(c) 545. Brown enviromental changes :2.5mM DTT 045 min dtt-1(1)
(c) 546. Brown enviromental changes :2.5mM DTT 060 min dtt-1(1)
(c) 557. Brown enviromental changes :1.5 mM diamide (5 min)(1)
(c) DES460 (wt) - mock irradiation - 5 min
(c) MAC1-up (C)
(c) wt-gal

ROX1 -*-> CYC1

(c) 6. Expression during the cell cycle (cdc15 arrest and release)(10)
(c) 7. Expression during the cell Cycle (cdc28)(15)
(c) 7. Expression during the cell Cycle (cdc28)(17)
(c) 446. Expression in response to 0.1% MMS for 10 min(1)
(c) 447. Expression in response to 0.1% MMS for 30 min(1)
(c) 463. Expression in response to 0.1% MMS for 60 min(1)
(c) 464. Expression in response to 0.2% MMS for 60 min(1)
(c) 483. Expression in response to alkali: 10,20,40,60,80,100 min(2)
(c) 485. Expression in response to peroxide: 10,20,40,60,120 min(1)
(c) 485. Expression in response to peroxide: 10,20,40,60,120 min(2)
(c) 532. Brown enviromental changes :constant 0.32 mM H2O2 (160 min) redo(1)
(c) 534. Brown enviromental changes :1 mM Menadione (20 min) redo(1)
(c) 535. Brown enviromental changes :1 mM Menadione (30 min) redo(1)
(c) 537. Brown enviromental changes :1 mM Menadione (50 min)redo(1)
(c) 556. Brown enviromental changes :dtt 480 min dtt-2(1)
(c) wt_plus_gamma_30_min
(c) MAC1-up (C)

ROX1 -*-| YLR413W

(c) 5. Expression during the cell cycle (alpha factor arrest and release)(6)
(c) 5. Expression during the cell cycle (alpha factor arrest and release)(7)
(c) 6. Expression during the cell cycle (cdc15 arrest and release)(4)
(c) 6. Expression during the cell cycle (cdc15 arrest and release)(5)
(c) 6. Expression during the cell cycle (cdc15 arrest and release)(6)
(c) 6. Expression during the cell cycle (cdc15 arrest and release)(7)
(c) 6. Expression during the cell cycle (cdc15 arrest and release)(8)
(c) 7. Expression during the cell Cycle (cdc28)(13)
(c) 7. Expression during the cell Cycle (cdc28)(15)
(c) 8. Expression during the cell cycle (cell size selection and release)(4)
(c) 89. Expression in response to 3-aminotriazole(1)
(c) 446. Expression in response to 0.1% MMS for 10 min(1)
(c) 463. Expression in response to 0.1% MMS for 60 min(1)
(c) 464. Expression in response to 0.2% MMS for 60 min(1)
(c) 485. Expression in response to peroxide: 10,20,40,60,120 min(1)
(c) 485. Expression in response to peroxide: 10,20,40,60,120 min(2)
(c) 485. Expression in response to peroxide: 10,20,40,60,120 min(3)
(c) 518. Brown enviromental changes :29C +1M sorbitol to 33C + 1M sorbitol - 15 minutes(1)

```
    (c) 523. Brown enviromental changes :constant 0.32 mM H2O2 (10 min) redo(1)
    (c) 534. Brown enviromental changes :1 mM Menadione (20 min) redo(1)
    (c) 535. Brown enviromental changes :1 mM Menadione (30 min) redo(1)
    (c) 536. Brown enviromental changes :1mM Menadione (40 min) redo(1)
    (c) 545. Brown enviromental changes :2.5mM DTT 045 min dtt-1(1)
    (c) 557. Brown enviromental changes :1.5 mM diamide (5 min)(1)
    (c) 576. Brown enviromental changes :Hypo-osmotic shock - 60 min(1)
    (c) wt_plus_gamma_45_min
    (c) DES460 (wt) - mock irradiation - 5 min
    (c) DES460 (wt) - mock irradiation - 30 min


ROX1 -*-| YOL002C


    (c) 5. Expression during the cell cycle (alpha factor arrest and release)(6)
    (c) 5. Expression during the cell cycle (alpha factor arrest and release)(7)
    (c) 6. Expression during the cell cycle (cdc15 arrest and release)(4)
    (c) 6. Expression during the cell cycle (cdc15 arrest and release)(5)
    (c) 6. Expression during the cell cycle (cdc15 arrest and release)(6)
    (c) 6. Expression during the cell cycle (cdc15 arrest and release)(7)
    (c) 6. Expression during the cell cycle (cdc15 arrest and release)(8)
    (c) 7. Expression during the cell Cycle (cdc28)(13)
    (c) 7. Expression during the cell Cycle (cdc28)(15)
    (c) 8. Expression during the cell cycle (cell size selection and release)(4)
    (c) 89. Expression in response to 3-aminotriazole(1)
    (c) 446. Expression in response to 0.1% MMS for 10 min(1)
    (c) 463. Expression in response to 0.1% MMS for 60 min(1)
    (c) 464. Expression in response to 0.2% MMS for 60 min(1)
    (c) 485. Expression in response to peroxide: 10,20,40,60,120 min(1)
    (c) 485. Expression in response to peroxide: 10,20,40,60,120 min(2)
    (c) 485. Expression in response to peroxide: 10,20,40,60,120 min(3)
    (c) 518. Brown enviromental changes :29C +1M sorbitol to 33C + 1M sorbitol - 15 minutes(1)
    (c) 523. Brown enviromental changes :constant 0.32 mM H2O2 (10 min) redo(1)
    (c) 534. Brown enviromental changes :1 mM Menadione (20 min) redo(1)
    (c) 535. Brown enviromental changes :1 mM Menadione (30 min) redo(1)
    (c) 536. Brown enviromental changes :1mM Menadione (40 min) redo(1)
    (c) 545. Brown enviromental changes :2.5mM DTT 045 min dtt-1(1)
    (c) 557. Brown enviromental changes :1.5 mM diamide (5 min)(1)
    (c) 576. Brown enviromental changes :Hypo-osmotic shock - 60 min(1)
    (c) wt_plus_gamma_45_min
    (c) DES460 (wt) - mock irradiation - 5 min
    (c) DES460 (wt) - mock irradiation - 30 min


SIP4 -*-> PCK1


    (c) 8. Expression during the cell cycle (cell size selection and release)(9)
    (c) 8. Expression during the cell cycle (cell size selection and release)(13)
    (c) 11. Expression during diauxic shift: 9h,11h,13h,15h,17h,19h,21h(7)
    (c) 49. Expression in response to 50 nM alpha-factor: 0,15,30,45,60,90,120 min(2)
    (c) 387. Rosetta 2000: Expression in cells with ERG11 under tet promoter(1)
    (c) 392. Rosetta 2000: Expression in cells with PMA1 under tet promoter(1)
    (c) 528. Brown enviromental changes :constant 0.32 mM H2O2 (60 min) redo(1)
    (c) 567. Brown enviromental changes :1M sorbitol - 30 min(1)
    (c) 599. Brown enviromental changes :diauxic shift timecourse(1)
    (c) 600. Brown enviromental changes :diauxic shift timecourse(1)
    (c) 604. Brown enviromental changes :YPD 8 h ypd-2(1)
    (c) 605. Brown enviromental changes :YPD 10 h  ypd-2(1)
    (c) 606. Brown enviromental changes :YPD 12 h ypd-2(1)
    (c) 607. Brown enviromental changes :YPD 1 d ypd-2(1)
    (c) 608. Brown enviromental changes :YPD 2 d ypd-2(1)
    (c) 614. Brown enviromental changes :YPD stationary phase 12 h ypd-1(1)
    (c) 615. Brown enviromental changes :YPD stationary phase 1 d ypd-1(1)
    (c) 616. Brown enviromental changes :YPD stationary phase 2 d ypd-1(1)
    (c) 617. Brown enviromental changes :YPD stationary phase 3 d ypd-1(1)
    (c) 618. Brown enviromental changes :YPD stationary phase 5 d ypd-1(1)
    (c) 675. Expression in response to propionate(1)
    (c) wt_plus_gamma_20_min
    (c) wt_plus_gamma_120_min


STE12 -*-> ASG7


    (c) 5. Expression during the cell cycle (alpha factor arrest and release)(1)
    (c) 48. Expression in response to 0.15,0.5,1.5,5,15.8,50,158,500 nM alpha-factor(3)
    (c) 48. Expression in response to 0.15,0.5,1.5,5,15.8,50,158,500 nM alpha-factor(4)
    (c) 48. Expression in response to 0.15,0.5,1.5,5,15.8,50,158,500 nM alpha-factor(5)
    (c) 48. Expression in response to 0.15,0.5,1.5,5,15.8,50,158,500 nM alpha-factor(6)
    (c) 48. Expression in response to 0.15,0.5,1.5,5,15.8,50,158,500 nM alpha-factor(7)
    (c) 48. Expression in response to 0.15,0.5,1.5,5,15.8,50,158,500 nM alpha-factor(8)
    (c) 49. Expression in response to 50 nM alpha-factor: 0,15,30,45,60,90,120 min(1)
    (c) 49. Expression in response to 50 nM alpha-factor: 0,15,30,45,60,90,120 min(2)
    (c) 49. Expression in response to 50 nM alpha-factor: 0,15,30,45,60,90,120 min(3)
    (c) 49. Expression in response to 50 nM alpha-factor: 0,15,30,45,60,90,120 min(4)
    (c) 49. Expression in response to 50 nM alpha-factor: 0,15,30,45,60,90,120 min(5)
    (c) 49. Expression in response to 50 nM alpha-factor: 0,15,30,45,60,90,120 min(6)
    (c) 49. Expression in response to 50 nM alpha-factor: 0,15,30,45,60,90,120 min(7)
    (c) 53. Expression in response to overproduction of Ste4p(1)
    (c) 54. Expression in response to overproduction of Ste5p(1)
    (c) 56. Expression in response to overproduction of Ste12p(1)
    (c) 388. Rosetta 2000: Expression in cells with FKS1 under tet promoter(1)
    (c) 565. Brown enviromental changes :1M sorbitol - 5 min(1)
    (c) 571. Brown enviromental changes :1M sorbitol - 120 min(1)
    (c) (Rich Media 2% Glucose YPD-185588) wt  5mM aF, 30 min.
    (c) (Rich Media 2% Glucose YPD-185769) wt  5mM aF, 30 min.
    (c) Rich Media 2% Glucose YPD-Average wt  5mM aF, 30 min.
    (c) 100 microM BCS 60 min


STE12 -*-> KAR4


    (c) 5. Expression during the cell cycle (alpha factor arrest and release)(1)
    (c) 48. Expression in response to 0.15,0.5,1.5,5,15.8,50,158,500 nM alpha-factor(3)
    (c) 48. Expression in response to 0.15,0.5,1.5,5,15.8,50,158,500 nM alpha-factor(4)
    (c) 48. Expression in response to 0.15,0.5,1.5,5,15.8,50,158,500 nM alpha-factor(5)
    (c) 48. Expression in response to 0.15,0.5,1.5,5,15.8,50,158,500 nM alpha-factor(6)
    (c) 48. Expression in response to 0.15,0.5,1.5,5,15.8,50,158,500 nM alpha-factor(7)
    (c) 48. Expression in response to 0.15,0.5,1.5,5,15.8,50,158,500 nM alpha-factor(8)
```

(c) 49. Expression in response to 50 nM alpha-factor: 0,15,30,45,60,90,120 min(1)
(c) 49. Expression in response to 50 nM alpha-factor: 0,15,30,45,60,90,120 min(2)
(c) 49. Expression in response to 50 nM alpha-factor: 0,15,30,45,60,90,120 min(3)
(c) 49. Expression in response to 50 nM alpha-factor: 0,15,30,45,60,90,120 min(4)
(c) 49. Expression in response to 50 nM alpha-factor: 0,15,30,45,60,90,120 min(5)
(c) 49. Expression in response to 50 nM alpha-factor: 0,15,30,45,60,90,120 min(6)
(c) 49. Expression in response to 50 nM alpha-factor: 0,15,30,45,60,90,120 min(7)
(c) 53. Expression in response to overproduction of Ste4p(1)
(c) 54. Expression in response to overproduction of Ste5p(1)
(c) 56. Expression in response to overproduction of Ste12p(1)
(c) 388. Rosetta 2000: Expression in cells with FKS1 under tet promoter(1)
(c) 565. Brown enviromental changes :1M sorbitol - 5 min(1)
(c) 571. Brown enviromental changes :1M sorbitol - 120 min(1)
(c) (Rich Media 2% Glucose YPD-185588) wt 5mM aF, 30 min.
(c) (Rich Media 2% Glucose YPD-185769) wt 5mM aF, 30 min.
(c) Rich Media 2% Glucose YPD-Average wt 5mM aF, 30 min.
(c) 100 microM BCS 60 min

STE12 -*-> PRM4

(c) 5. Expression during the cell cycle (alpha factor arrest and release)(1)
(c) 48. Expression in response to 0.15,0.5,1.5,5,15.8,50,158,500 nM alpha-factor(3)
(c) 48. Expression in response to 0.15,0.5,1.5,5,15.8,50,158,500 nM alpha-factor(4)
(c) 48. Expression in response to 0.15,0.5,1.5,5,15.8,50,158,500 nM alpha-factor(5)
(c) 48. Expression in response to 0.15,0.5,1.5,5,15.8,50,158,500 nM alpha-factor(6)
(c) 48. Expression in response to 0.15,0.5,1.5,5,15.8,50,158,500 nM alpha-factor(7)
(c) 48. Expression in response to 0.15,0.5,1.5,5,15.8,50,158,500 nM alpha-factor(8)
(c) 49. Expression in response to 50 nM alpha-factor: 0,15,30,45,60,90,120 min(1)
(c) 49. Expression in response to 50 nM alpha-factor: 0,15,30,45,60,90,120 min(2)
(c) 49. Expression in response to 50 nM alpha-factor: 0,15,30,45,60,90,120 min(3)
(c) 49. Expression in response to 50 nM alpha-factor: 0,15,30,45,60,90,120 min(4)
(c) 49. Expression in response to 50 nM alpha-factor: 0,15,30,45,60,90,120 min(5)
(c) 49. Expression in response to 50 nM alpha-factor: 0,15,30,45,60,90,120 min(6)
(c) 49. Expression in response to 50 nM alpha-factor: 0,15,30,45,60,90,120 min(7)
(c) 53. Expression in response to overproduction of Ste4p(1)
(c) 54. Expression in response to overproduction of Ste5p(1)
(c) 56. Expression in response to overproduction of Ste12p(1)
(c) 388. Rosetta 2000: Expression in cells with FKS1 under tet promoter(1)
(c) 565. Brown enviromental changes :1M sorbitol - 5 min(1)
(c) 571. Brown enviromental changes :1M sorbitol - 120 min(1)
(c) (Rich Media 2% Glucose YPD-185588) wt 5mM aF, 30 min.
(c) (Rich Media 2% Glucose YPD-185769) wt 5mM aF, 30 min.
(c) Rich Media 2% Glucose YPD-Average wt 5mM aF, 30 min.
(c) 100 microM BCS 60 min

STE12 -*-> PRM6

(c) 5. Expression during the cell cycle (alpha factor arrest and release)(1)
(c) 48. Expression in response to 0.15,0.5,1.5,5,15.8,50,158,500 nM alpha-factor(3)
(c) 48. Expression in response to 0.15,0.5,1.5,5,15.8,50,158,500 nM alpha-factor(4)
(c) 48. Expression in response to 0.15,0.5,1.5,5,15.8,50,158,500 nM alpha-factor(5)
(c) 48. Expression in response to 0.15,0.5,1.5,5,15.8,50,158,500 nM alpha-factor(6)
(c) 48. Expression in response to 0.15,0.5,1.5,5,15.8,50,158,500 nM alpha-factor(7)
(c) 48. Expression in response to 0.15,0.5,1.5,5,15.8,50,158,500 nM alpha-factor(8)
(c) 49. Expression in response to 50 nM alpha-factor: 0,15,30,45,60,90,120 min(1)
(c) 49. Expression in response to 50 nM alpha-factor: 0,15,30,45,60,90,120 min(2)
(c) 49. Expression in response to 50 nM alpha-factor: 0,15,30,45,60,90,120 min(3)
(c) 49. Expression in response to 50 nM alpha-factor: 0,15,30,45,60,90,120 min(4)
(c) 49. Expression in response to 50 nM alpha-factor: 0,15,30,45,60,90,120 min(5)
(c) 49. Expression in response to 50 nM alpha-factor: 0,15,30,45,60,90,120 min(6)
(c) 49. Expression in response to 50 nM alpha-factor: 0,15,30,45,60,90,120 min(7)
(c) 53. Expression in response to overproduction of Ste4p(1)
(c) 54. Expression in response to overproduction of Ste5p(1)
(c) 56. Expression in response to overproduction of Ste12p(1)
(c) 388. Rosetta 2000: Expression in cells with FKS1 under tet promoter(1)
(c) 565. Brown enviromental changes :1M sorbitol - 5 min(1)
(c) 571. Brown enviromental changes :1M sorbitol - 120 min(1)
(c) (Rich Media 2% Glucose YPD-185588) wt 5mM aF, 30 min.
(c) (Rich Media 2% Glucose YPD-185769) wt 5mM aF, 30 min.
(c) Rich Media 2% Glucose YPD-Average wt 5mM aF, 30 min.
(c) 100 microM BCS 60 min

STE12 -*-> PRP39

(c) 5. Expression during the cell cycle (alpha factor arrest and release)(1)
(c) 48. Expression in response to 0.15,0.5,1.5,5,15.8,50,158,500 nM alpha-factor(3)
(c) 48. Expression in response to 0.15,0.5,1.5,5,15.8,50,158,500 nM alpha-factor(4)
(c) 48. Expression in response to 0.15,0.5,1.5,5,15.8,50,158,500 nM alpha-factor(5)
(c) 48. Expression in response to 0.15,0.5,1.5,5,15.8,50,158,500 nM alpha-factor(6)
(c) 48. Expression in response to 0.15,0.5,1.5,5,15.8,50,158,500 nM alpha-factor(7)
(c) 48. Expression in response to 0.15,0.5,1.5,5,15.8,50,158,500 nM alpha-factor(8)
(c) 49. Expression in response to 50 nM alpha-factor: 0,15,30,45,60,90,120 min(1)
(c) 49. Expression in response to 50 nM alpha-factor: 0,15,30,45,60,90,120 min(2)
(c) 49. Expression in response to 50 nM alpha-factor: 0,15,30,45,60,90,120 min(3)
(c) 49. Expression in response to 50 nM alpha-factor: 0,15,30,45,60,90,120 min(4)
(c) 49. Expression in response to 50 nM alpha-factor: 0,15,30,45,60,90,120 min(5)
(c) 49. Expression in response to 50 nM alpha-factor: 0,15,30,45,60,90,120 min(6)
(c) 49. Expression in response to 50 nM alpha-factor: 0,15,30,45,60,90,120 min(7)
(c) 53. Expression in response to overproduction of Ste4p(1)
(c) 54. Expression in response to overproduction of Ste5p(1)
(c) 56. Expression in response to overproduction of Ste12p(1)
(c) 388. Rosetta 2000: Expression in cells with FKS1 under tet promoter(1)
(c) 565. Brown enviromental changes :1M sorbitol - 5 min(1)
(c) 571. Brown enviromental changes :1M sorbitol - 120 min(1)
(c) (Rich Media 2% Glucose YPD-185588) wt 5mM aF, 30 min.
(c) (Rich Media 2% Glucose YPD-185769) wt 5mM aF, 30 min.
(c) Rich Media 2% Glucose YPD-Average wt 5mM aF, 30 min.
(c) 100 microM BCS 60 min

STE12 -*-> YDR249C

(c) 5. Expression during the cell cycle (alpha factor arrest and release)(1)
(c) 48. Expression in response to 0.15,0.5,1.5,5,15.8,50,158,500 nM alpha-factor(3)
(c) 48. Expression in response to 0.15,0.5,1.5,5,15.8,50,158,500 nM alpha-factor(4)

(c) 48. Expression in response to 0.15,0.5,1.5,5,15.8,50,158,500 nM alpha-factor(5)
(c) 48. Expression in response to 0.15,0.5,1.5,5,15.8,50,158,500 nM alpha-factor(6)
(c) 48. Expression in response to 0.15,0.5,1.5,5,15.8,50,158,500 nM alpha-factor(7)
(c) 48. Expression in response to 0.15,0.5,1.5,5,15.8,50,158,500 nM alpha-factor(8)
(c) 49. Expression in response to 50 nM alpha-factor: 0,15,30,45,60,90,120 min(1)
(c) 49. Expression in response to 50 nM alpha-factor: 0,15,30,45,60,90,120 min(2)
(c) 49. Expression in response to 50 nM alpha-factor: 0,15,30,45,60,90,120 min(3)
(c) 49. Expression in response to 50 nM alpha-factor: 0,15,30,45,60,90,120 min(4)
(c) 49. Expression in response to 50 nM alpha-factor: 0,15,30,45,60,90,120 min(5)
(c) 49. Expression in response to 50 nM alpha-factor: 0,15,30,45,60,90,120 min(6)
(c) 49. Expression in response to 50 nM alpha-factor: 0,15,30,45,60,90,120 min(7)
(c) 53. Expression in response to overproduction of Ste4p(1)
(c) 54. Expression in response to overproduction of Ste5p(1)
(c) 56. Expression in response to overproduction of Ste12p(1)
(c) 388. Rosetta 2000: Expression in cells with FKS1 under tet promoter(1)
(c) 565. Brown enviromental changes :1M sorbitol - 5 min(1)
(c) 571. Brown enviromental changes :1M sorbitol - 120 min(1)
(c) (Rich Media 2% Glucose YPD-185588) wt 5mM aF, 30 min.
(c) (Rich Media 2% Glucose YPD-185769) wt 5mM aF, 30 min.
(c) Rich Media 2% Glucose YPD-Average wt 5mM aF, 30 min.
(c) 100 microM BCS 60 min

STE12 -*-> GPA1

(c) 5. Expression during the cell cycle (alpha factor arrest and release)(1)
(c) 6. Expression during the cell cycle (cdc15 arrest and release)(22)
(c) 6. Expression during the cell cycle (cdc15 arrest and release)(23)
(c) 6. Expression during the cell cycle (cdc15 arrest and release)(24)
(c) 48. Expression in response to 0.15,0.5,1.5,5,15.8,50,158,500 nM alpha-factor(3)
(c) 48. Expression in response to 0.15,0.5,1.5,5,15.8,50,158,500 nM alpha-factor(4)
(c) 48. Expression in response to 0.15,0.5,1.5,5,15.8,50,158,500 nM alpha-factor(5)
(c) 48. Expression in response to 0.15,0.5,1.5,5,15.8,50,158,500 nM alpha-factor(6)
(c) 48. Expression in response to 0.15,0.5,1.5,5,15.8,50,158,500 nM alpha-factor(7)
(c) 48. Expression in response to 0.15,0.5,1.5,5,15.8,50,158,500 nM alpha-factor(8)
(c) 49. Expression in response to 50 nM alpha-factor: 0,15,30,45,60,90,120 min(1)
(c) 49. Expression in response to 50 nM alpha-factor: 0,15,30,45,60,90,120 min(2)
(c) 49. Expression in response to 50 nM alpha-factor: 0,15,30,45,60,90,120 min(3)
(c) 49. Expression in response to 50 nM alpha-factor: 0,15,30,45,60,90,120 min(4)
(c) 49. Expression in response to 50 nM alpha-factor: 0,15,30,45,60,90,120 min(6)
(c) 49. Expression in response to 50 nM alpha-factor: 0,15,30,45,60,90,120 min(7)
(c) 53. Expression in response to overproduction of Ste4p(1)
(c) 54. Expression in response to overproduction of Ste5p(1)
(c) 55. Expression in response to overproduction of Ste11p(1)
(c) 56. Expression in response to overproduction of Ste12p(1)
(c) (Rich Media 2% Glucose YPD-185588) wt 5mM aF, 30 min.
(c) (Rich Media 2% Glucose YPD-185769) wt 5mM aF, 30 min.
(c) Rich Media 2% Glucose YPD-Average wt 5mM aF, 30 min.

STE12 -*-> MFA2

(c) 5. Expression during the cell cycle (alpha factor arrest and release)(1)
(c) 6. Expression during the cell cycle (cdc15 arrest and release)(22)
(c) 6. Expression during the cell cycle (cdc15 arrest and release)(23)
(c) 6. Expression during the cell cycle (cdc15 arrest and release)(24)
(c) 48. Expression in response to 0.15,0.5,1.5,5,15.8,50,158,500 nM alpha-factor(3)
(c) 48. Expression in response to 0.15,0.5,1.5,5,15.8,50,158,500 nM alpha-factor(4)
(c) 48. Expression in response to 0.15,0.5,1.5,5,15.8,50,158,500 nM alpha-factor(5)
(c) 48. Expression in response to 0.15,0.5,1.5,5,15.8,50,158,500 nM alpha-factor(6)
(c) 48. Expression in response to 0.15,0.5,1.5,5,15.8,50,158,500 nM alpha-factor(7)
(c) 48. Expression in response to 0.15,0.5,1.5,5,15.8,50,158,500 nM alpha-factor(8)
(c) 49. Expression in response to 50 nM alpha-factor: 0,15,30,45,60,90,120 min(1)
(c) 49. Expression in response to 50 nM alpha-factor: 0,15,30,45,60,90,120 min(2)
(c) 49. Expression in response to 50 nM alpha-factor: 0,15,30,45,60,90,120 min(3)
(c) 49. Expression in response to 50 nM alpha-factor: 0,15,30,45,60,90,120 min(4)
(c) 49. Expression in response to 50 nM alpha-factor: 0,15,30,45,60,90,120 min(6)
(c) 49. Expression in response to 50 nM alpha-factor: 0,15,30,45,60,90,120 min(7)
(c) 53. Expression in response to overproduction of Ste4p(1)
(c) 54. Expression in response to overproduction of Ste5p(1)
(c) 55. Expression in response to overproduction of Ste11p(1)
(c) 56. Expression in response to overproduction of Ste12p(1)
(c) (Rich Media 2% Glucose YPD-185588) wt 5mM aF, 30 min.
(c) (Rich Media 2% Glucose YPD-185769) wt 5mM aF, 30 min.
(c) Rich Media 2% Glucose YPD-Average wt 5mM aF, 30 min.

STE12 -*-> SLI15

(c) 5. Expression during the cell cycle (alpha factor arrest and release)(1)
(c) 6. Expression during the cell cycle (cdc15 arrest and release)(22)
(c) 6. Expression during the cell cycle (cdc15 arrest and release)(23)
(c) 6. Expression during the cell cycle (cdc15 arrest and release)(24)
(c) 48. Expression in response to 0.15,0.5,1.5,5,15.8,50,158,500 nM alpha-factor(3)
(c) 48. Expression in response to 0.15,0.5,1.5,5,15.8,50,158,500 nM alpha-factor(4)
(c) 48. Expression in response to 0.15,0.5,1.5,5,15.8,50,158,500 nM alpha-factor(5)
(c) 48. Expression in response to 0.15,0.5,1.5,5,15.8,50,158,500 nM alpha-factor(6)
(c) 48. Expression in response to 0.15,0.5,1.5,5,15.8,50,158,500 nM alpha-factor(7)
(c) 48. Expression in response to 0.15,0.5,1.5,5,15.8,50,158,500 nM alpha-factor(8)
(c) 49. Expression in response to 50 nM alpha-factor: 0,15,30,45,60,90,120 min(1)
(c) 49. Expression in response to 50 nM alpha-factor: 0,15,30,45,60,90,120 min(2)
(c) 49. Expression in response to 50 nM alpha-factor: 0,15,30,45,60,90,120 min(3)
(c) 49. Expression in response to 50 nM alpha-factor: 0,15,30,45,60,90,120 min(4)
(c) 49. Expression in response to 50 nM alpha-factor: 0,15,30,45,60,90,120 min(6)
(c) 49. Expression in response to 50 nM alpha-factor: 0,15,30,45,60,90,120 min(7)
(c) 53. Expression in response to overproduction of Ste4p(1)
(c) 54. Expression in response to overproduction of Ste5p(1)
(c) 55. Expression in response to overproduction of Ste11p(1)
(c) 56. Expression in response to overproduction of Ste12p(1)
(c) (Rich Media 2% Glucose YPD-185588) wt 5mM aF, 30 min.
(c) (Rich Media 2% Glucose YPD-185769) wt 5mM aF, 30 min.
(c) Rich Media 2% Glucose YPD-Average wt 5mM aF, 30 min.

STE12 -*-> TEC1

(c) 5. Expression during the cell cycle (alpha factor arrest and release)(1)
(c) 6. Expression during the cell cycle (cdc15 arrest and release)(22)

```
    (c) 6. Expression during the cell cycle (cdc15 arrest and release)(23)
    (c) 6. Expression during the cell cycle (cdc15 arrest and release)(24)
    (c) 48. Expression in response to 0.15,0.5,1.5,5,15.8,50,158,500 nM alpha-factor(3)
    (c) 48. Expression in response to 0.15,0.5,1.5,5,15.8,50,158,500 nM alpha-factor(4)
    (c) 48. Expression in response to 0.15,0.5,1.5,5,15.8,50,158,500 nM alpha-factor(5)
    (c) 48. Expression in response to 0.15,0.5,1.5,5,15.8,50,158,500 nM alpha-factor(6)
    (c) 48. Expression in response to 0.15,0.5,1.5,5,15.8,50,158,500 nM alpha-factor(7)
    (c) 48. Expression in response to 0.15,0.5,1.5,5,15.8,50,158,500 nM alpha-factor(8)
    (c) 49. Expression in response to 50 nM alpha-factor: 0,15,30,45,60,90,120 min(1)
    (c) 49. Expression in response to 50 nM alpha-factor: 0,15,30,45,60,90,120 min(2)
    (c) 49. Expression in response to 50 nM alpha-factor: 0,15,30,45,60,90,120 min(3)
    (c) 49. Expression in response to 50 nM alpha-factor: 0,15,30,45,60,90,120 min(4)
    (c) 49. Expression in response to 50 nM alpha-factor: 0,15,30,45,60,90,120 min(6)
    (c) 49. Expression in response to 50 nM alpha-factor: 0,15,30,45,60,90,120 min(7)
    (c) 53. Expression in response to overproduction of Ste4p(1)
    (c) 54. Expression in response to overproduction of Ste5p(1)
    (c) 55. Expression in response to overproduction of Ste11p(1)
    (c) 56. Expression in response to overproduction of Ste12p(1)
    (c) (Rich Media 2% Glucose YPD-185588) wt  5mM aF, 30 min.
    (c) (Rich Media 2% Glucose YPD-185769) wt  5mM aF, 30 min.
    (c) Rich Media 2% Glucose YPD-Average wt  5mM aF, 30 min.


STE12 -*-> FUS2


    (c) 5. Expression during the cell cycle (alpha factor arrest and release)(1)
    (c) 5. Expression during the cell cycle (alpha factor arrest and release)(11)
    (c) 5. Expression during the cell cycle (alpha factor arrest and release)(12)
    (c) 5. Expression during the cell cycle (alpha factor arrest and release)(13)
    (c) 8. Expression during the cell cycle (cell size selection and release)(12)
    (c) 8. Expression during the cell cycle (cell size selection and release)(13)
    (c) 26. Fink: Expression in diploid high copy TEC1(1)
    (c) 48. Expression in response to 0.15,0.5,1.5,5,15.8,50,158,500 nM alpha-factor(4)
    (c) 48. Expression in response to 0.15,0.5,1.5,5,15.8,50,158,500 nM alpha-factor(5)
    (c) 48. Expression in response to 0.15,0.5,1.5,5,15.8,50,158,500 nM alpha-factor(6)
    (c) 48. Expression in response to 0.15,0.5,1.5,5,15.8,50,158,500 nM alpha-factor(7)
    (c) 48. Expression in response to 0.15,0.5,1.5,5,15.8,50,158,500 nM alpha-factor(8)
    (c) 49. Expression in response to 50 nM alpha-factor: 0,15,30,45,60,90,120 min(1)
    (c) 49. Expression in response to 50 nM alpha-factor: 0,15,30,45,60,90,120 min(2)
    (c) 49. Expression in response to 50 nM alpha-factor: 0,15,30,45,60,90,120 min(3)
    (c) 49. Expression in response to 50 nM alpha-factor: 0,15,30,45,60,90,120 min(6)
    (c) 49. Expression in response to 50 nM alpha-factor: 0,15,30,45,60,90,120 min(7)
    (c) 53. Expression in response to overproduction of Ste4p(1)
    (c) 54. Expression in response to overproduction of Ste5p(1)
    (c) 55. Expression in response to overproduction of Ste11p(1)
    (c) 56. Expression in response to overproduction of Ste12p(1)
    (c) 483. Expression in response to alkali: 10,20,40,60,80,100 min(5)
    (c) 571. Brown enviromental changes :1M sorbitol - 120 min(1)
    (c) (Rich Media 2% Glucose YPD-185588) wt  5mM aF, 30 min.
    (c) 100 microM CuSO4 30 min


STE12 -*-> GYP8


    (c) 5. Expression during the cell cycle (alpha factor arrest and release)(1)
    (c) 11. Expression during diauxic shift: 9h,11h,13h,15h,17h,19h,21h(2)
    (c) 11. Expression during diauxic shift: 9h,11h,13h,15h,17h,19h,21h(3)
    (c) 48. Expression in response to 0.15,0.5,1.5,5,15.8,50,158,500 nM alpha-factor(4)
    (c) 48. Expression in response to 0.15,0.5,1.5,5,15.8,50,158,500 nM alpha-factor(5)
    (c) 48. Expression in response to 0.15,0.5,1.5,5,15.8,50,158,500 nM alpha-factor(6)
    (c) 48. Expression in response to 0.15,0.5,1.5,5,15.8,50,158,500 nM alpha-factor(7)
    (c) 48. Expression in response to 0.15,0.5,1.5,5,15.8,50,158,500 nM alpha-factor(8)
    (c) 49. Expression in response to 50 nM alpha-factor: 0,15,30,45,60,90,120 min(1)
    (c) 49. Expression in response to 50 nM alpha-factor: 0,15,30,45,60,90,120 min(2)
    (c) 49. Expression in response to 50 nM alpha-factor: 0,15,30,45,60,90,120 min(3)
    (c) 49. Expression in response to 50 nM alpha-factor: 0,15,30,45,60,90,120 min(4)
    (c) 49. Expression in response to 50 nM alpha-factor: 0,15,30,45,60,90,120 min(6)
    (c) 49. Expression in response to 50 nM alpha-factor: 0,15,30,45,60,90,120 min(7)
    (c) 54. Expression in response to overproduction of Ste5p(1)
    (c) 56. Expression in response to overproduction of Ste12p(1)
    (c) 406. Rosetta 2000: Expression in response to Terbinafine(1)
    (c) 453. Expression in response to gama-ray exposure (30 kilorad) for 60 min(1)
    (c) 455. Expression in response to high MNNG (27 microgram/ml) for 60 min(1)
    (c) 456. Expression in response to high 4NQO (8 microgram/ml) for 60 min(1)
    (c) 533. Brown enviromental changes :1 mM Menadione (10 min)redo(1)
    (c) 534. Brown enviromental changes :1 mM Menadione (20 min) redo(1)
    (c) 594. Brown enviromental changes :diauxic shift timecourse(1)
    (c) 595. Brown enviromental changes :diauxic shift timecourse(1)
    (c) wt_plus_gamma_30_min
    (c) 100 microM BCS 60 min


STE12 -*-> AXL1


    (c) 5. Expression during the cell cycle (alpha factor arrest and release)(1)
    (c) 48. Expression in response to 0.15,0.5,1.5,5,15.8,50,158,500 nM alpha-factor(3)
    (c) 48. Expression in response to 0.15,0.5,1.5,5,15.8,50,158,500 nM alpha-factor(4)
    (c) 48. Expression in response to 0.15,0.5,1.5,5,15.8,50,158,500 nM alpha-factor(5)
    (c) 48. Expression in response to 0.15,0.5,1.5,5,15.8,50,158,500 nM alpha-factor(6)
    (c) 48. Expression in response to 0.15,0.5,1.5,5,15.8,50,158,500 nM alpha-factor(7)
    (c) 48. Expression in response to 0.15,0.5,1.5,5,15.8,50,158,500 nM alpha-factor(8)
    (c) 49. Expression in response to 50 nM alpha-factor: 0,15,30,45,60,90,120 min(1)
    (c) 49. Expression in response to 50 nM alpha-factor: 0,15,30,45,60,90,120 min(2)
    (c) 49. Expression in response to 50 nM alpha-factor: 0,15,30,45,60,90,120 min(3)
    (c) 49. Expression in response to 50 nM alpha-factor: 0,15,30,45,60,90,120 min(4)
    (c) 49. Expression in response to 50 nM alpha-factor: 0,15,30,45,60,90,120 min(6)
    (c) 49. Expression in response to 50 nM alpha-factor: 0,15,30,45,60,90,120 min(7)
    (c) 53. Expression in response to overproduction of Ste4p(1)
    (c) 54. Expression in response to overproduction of Ste5p(1)
    (c) 55. Expression in response to overproduction of Ste11p(1)
    (c) 56. Expression in response to overproduction of Ste12p(1)
    (c) (Rich Media 2% Glucose YPD-185588) wt  5mM aF, 30 min.
    (c) (Rich Media 2% Glucose YPD-185769) wt  5mM aF, 30 min.
    (c) Rich Media 2% Glucose YPD-Average wt  5mM aF, 30 min.
    (c) (Var.) Rich Media 2% Glucose YPD-Average wt  5mM aF, 30 min.
    (c) 100 microM BCS 60 min


STE12 -*-> INP52
```

```
    (c) 5. Expression during the cell cycle (alpha factor arrest and release)(1)
    (c) 6. Expression during the cell cycle (cdc15 arrest and release)(14)
    (c) 7. Expression during the cell Cycle (cdc28)(5)
    (c) 8. Expression during the cell cycle (cell size selection and release)(7)
    (c) 48. Expression in response to 0.15,0.5,1.5,5,15.8,50,158,500 nM alpha-factor(5)
    (c) 48. Expression in response to 0.15,0.5,1.5,5,15.8,50,158,500 nM alpha-factor(6)
    (c) 48. Expression in response to 0.15,0.5,1.5,5,15.8,50,158,500 nM alpha-factor(7)
    (c) 48. Expression in response to 0.15,0.5,1.5,5,15.8,50,158,500 nM alpha-factor(8)
    (c) 49. Expression in response to 50 nM alpha-factor: 0,15,30,45,60,90,120 min(1)
    (c) 49. Expression in response to 50 nM alpha-factor: 0,15,30,45,60,90,120 min(3)
    (c) 49. Expression in response to 50 nM alpha-factor: 0,15,30,45,60,90,120 min(4)
    (c) 49. Expression in response to 50 nM alpha-factor: 0,15,30,45,60,90,120 min(5)
    (c) 49. Expression in response to 50 nM alpha-factor: 0,15,30,45,60,90,120 min(6)
    (c) 49. Expression in response to 50 nM alpha-factor: 0,15,30,45,60,90,120 min(7)
    (c) 53. Expression in response to overproduction of Ste4p(1)
    (c) 56. Expression in response to overproduction of Ste12p(1)
    (c) 502. Brown enviromental changes :Heat Shock 060 minutes  hs-2(1)
    (c) MAC1-up (B)


STE12 -*-> YJL107C


    (c) 5. Expression during the cell cycle (alpha factor arrest and release)(1)
    (c) 6. Expression during the cell cycle (cdc15 arrest and release)(14)
    (c) 7. Expression during the cell Cycle (cdc28)(5)
    (c) 8. Expression during the cell cycle (cell size selection and release)(7)
    (c) 48. Expression in response to 0.15,0.5,1.5,5,15.8,50,158,500 nM alpha-factor(5)
    (c) 48. Expression in response to 0.15,0.5,1.5,5,15.8,50,158,500 nM alpha-factor(6)
    (c) 48. Expression in response to 0.15,0.5,1.5,5,15.8,50,158,500 nM alpha-factor(7)
    (c) 48. Expression in response to 0.15,0.5,1.5,5,15.8,50,158,500 nM alpha-factor(8)
    (c) 49. Expression in response to 50 nM alpha-factor: 0,15,30,45,60,90,120 min(1)
    (c) 49. Expression in response to 50 nM alpha-factor: 0,15,30,45,60,90,120 min(3)
    (c) 49. Expression in response to 50 nM alpha-factor: 0,15,30,45,60,90,120 min(4)
    (c) 49. Expression in response to 50 nM alpha-factor: 0,15,30,45,60,90,120 min(5)
    (c) 49. Expression in response to 50 nM alpha-factor: 0,15,30,45,60,90,120 min(6)
    (c) 49. Expression in response to 50 nM alpha-factor: 0,15,30,45,60,90,120 min(7)
    (c) 53. Expression in response to overproduction of Ste4p(1)
    (c) 56. Expression in response to overproduction of Ste12p(1)
    (c) 502. Brown enviromental changes :Heat Shock 060 minutes  hs-2(1)
    (c) MAC1-up (B)


STE12 -*-> RGD2


    (c) 5. Expression during the cell cycle (alpha factor arrest and release)(16)
    (c) 7. Expression during the cell Cycle (cdc28)(17)
    (c) 8. Expression during the cell cycle (cell size selection and release)(4)
    (c) 8. Expression during the cell cycle (cell size selection and release)(13)
    (c) 8. Expression during the cell cycle (cell size selection and release)(14)
    (c) 11. Expression during diauxic shift: 9h,11h,13h,15h,17h,19h,21h(2)
    (c) 11. Expression during diauxic shift: 9h,11h,13h,15h,17h,19h,21h(3)
    (c) 48. Expression in response to 0.15,0.5,1.5,5,15.8,50,158,500 nM alpha-factor(5)
    (c) 49. Expression in response to 50 nM alpha-factor: 0,15,30,45,60,90,120 min(1)
    (c) 49. Expression in response to 50 nM alpha-factor: 0,15,30,45,60,90,120 min(2)
    (c) 49. Expression in response to 50 nM alpha-factor: 0,15,30,45,60,90,120 min(7)
    (c) 56. Expression in response to overproduction of Ste12p(1)
    (c) 399. Rosetta 2000: Expression in response to FR901,228(1)
    (c) 481. Expression in response to heat shock: 15,30,45,60,120 min(1)
    (c) 486. Expression in response to NaCl: 15 30 45 60 120 min(2)
    (c) 486. Expression in response to NaCl: 15 30 45 60 120 min(3)
    (c) 487. Expression in response to sorbitol: 15 30 45 90 120 min(2)
    (c) 487. Expression in response to sorbitol: 15 30 45 90 120 min(3)
    (c) 487. Expression in response to sorbitol: 15 30 45 90 120 min(5)
    (c) 505. Brown enviromental changes :37C to 25C shock - 45 min(1)
    (c) 518. Brown enviromental changes :29C +1M sorbitol to 33C + 1M sorbitol - 15 minutes(1)
    (c) 519. Brown enviromental changes :29C +1M sorbitol to 33C + 1M sorbitol - 30 minutes(1)
    (c) 594. Brown enviromental changes :diauxic shift timecourse(1)
    (c) 595. Brown enviromental changes :diauxic shift timecourse(1)
    (c) 633. Brown enviromental changes :MSN2 overexpression (repeat)(1)
    (c) Addition of 1M NaCl (90')


STE12 -*-> SCW10


    (c) 2. Cell Cycle: Expression in response to Cln3p (set 2)(1)
    (c) 5. Expression during the cell cycle (alpha factor arrest and release)(1)
    (c) 5. Expression during the cell cycle (alpha factor arrest and release)(7)
    (c) 5. Expression during the cell cycle (alpha factor arrest and release)(13)
    (c) 6. Expression during the cell cycle (cdc15 arrest and release)(14)
    (c) 7. Expression during the cell Cycle (cdc28)(13)
    (c) 8. Expression during the cell cycle (cell size selection and release)(7)
    (c) 8. Expression during the cell cycle (cell size selection and release)(8)
    (c) 8. Expression during the cell cycle (cell size selection and release)(9)
    (c) 8. Expression during the cell cycle (cell size selection and release)(10)
    (c) 8. Expression during the cell cycle (cell size selection and release)(12)
    (c) 11. Expression during diauxic shift: 9h,11h,13h,15h,17h,19h,21h(3)
    (c) 26. Fink: Expression in diploid high copy TEC1(1)
    (c) 48. Expression in response to 0.15,0.5,1.5,5,15.8,50,158,500 nM alpha-factor(4)
    (c) 48. Expression in response to 0.15,0.5,1.5,5,15.8,50,158,500 nM alpha-factor(5)
    (c) 48. Expression in response to 0.15,0.5,1.5,5,15.8,50,158,500 nM alpha-factor(6)
    (c) 48. Expression in response to 0.15,0.5,1.5,5,15.8,50,158,500 nM alpha-factor(7)
    (c) 48. Expression in response to 0.15,0.5,1.5,5,15.8,50,158,500 nM alpha-factor(8)
    (c) 49. Expression in response to 50 nM alpha-factor: 0,15,30,45,60,90,120 min(1)
    (c) 49. Expression in response to 50 nM alpha-factor: 0,15,30,45,60,90,120 min(2)
    (c) 49. Expression in response to 50 nM alpha-factor: 0,15,30,45,60,90,120 min(3)
    (c) 49. Expression in response to 50 nM alpha-factor: 0,15,30,45,60,90,120 min(4)
    (c) 49. Expression in response to 50 nM alpha-factor: 0,15,30,45,60,90,120 min(6)
    (c) 49. Expression in response to 50 nM alpha-factor: 0,15,30,45,60,90,120 min(7)
    (c) 53. Expression in response to overproduction of Ste4p(1)
    (c) 54. Expression in response to overproduction of Ste5p(1)
    (c) 56. Expression in response to overproduction of Ste12p(1)
    (c) 595. Brown enviromental changes :diauxic shift timecourse(1)
    (c) 681. Expression in response to 0.4M NaCl for 10 min in wild type(1)
    (c) 100 microM BCS 30 min


STE12 -*-> NRG2
```

(c) 5. Expression during the cell cycle (alpha factor arrest and release)(10)
(c) 5. Expression during the cell cycle (alpha factor arrest and release)(11)
(c) 5. Expression during the cell cycle (alpha factor arrest and release)(12)
(c) 6. Expression during the cell cycle (cdc15 arrest and release)(20)
(c) 6. Expression during the cell cycle (cdc15 arrest and release)(23)
(c) 7. Expression during the cell Cycle (cdc28)(1)
(c) 7. Expression during the cell Cycle (cdc28)(17)
(c) 8. Expression during the cell cycle (cell size selection and release)(12)
(c) 8. Expression during the cell cycle (cell size selection and release)(14)
(c) 48. Expression in response to 0.15,0.5,1.5,5,15.8,50,158,500 nM alpha-factor(4)
(c) 48. Expression in response to 0.15,0.5,1.5,5,15.8,50,158,500 nM alpha-factor(5)
(c) 49. Expression in response to 50 nM alpha-factor: 0,15,30,45,60,90,120 min(1)
(c) 49. Expression in response to 50 nM alpha-factor: 0,15,30,45,60,90,120 min(2)
(c) 49. Expression in response to 50 nM alpha-factor: 0,15,30,45,60,90,120 min(3)
(c) 49. Expression in response to 50 nM alpha-factor: 0,15,30,45,60,90,120 min(6)
(c) 49. Expression in response to 50 nM alpha-factor: 0,15,30,45,60,90,120 min(7)
(c) 56. Expression in response to overproduction of Ste12p(1)
(c) 405. Rosetta 2000: Expression in response to Nikkomycin Z(1)
(c) 565. Brown enviromental changes :1M sorbitol - 5 min(1)
(c) 674. Expression in response to oligomycin 120min(1)
(c) 681. Expression in response to 0.4M NaCl for 10 min in wild type(1)
(c) 683. Expression in response to 0.4M NaCl for 20 min in wild type(1)
(c) 685. Expression in response to 0.8M NaCl for 20 min in wild type(1)
(c) DES460 (wt) - mock irradiation - 5 min
(c) DES460 (wt) - mock irradiation - 30 min

STE12 -*-> PMU1

(c) 5. Expression during the cell cycle (alpha factor arrest and release)(1)
(c) 5. Expression during the cell cycle (alpha factor arrest and release)(12)
(c) 5. Expression during the cell cycle (alpha factor arrest and release)(13)
(c) 5. Expression during the cell cycle (alpha factor arrest and release)(15)
(c) 5. Expression during the cell cycle (alpha factor arrest and release)(16)
(c) 48. Expression in response to 0.15,0.5,1.5,5,15.8,50,158,500 nM alpha-factor(4)
(c) 48. Expression in response to 0.15,0.5,1.5,5,15.8,50,158,500 nM alpha-factor(5)
(c) 48. Expression in response to 0.15,0.5,1.5,5,15.8,50,158,500 nM alpha-factor(6)
(c) 48. Expression in response to 0.15,0.5,1.5,5,15.8,50,158,500 nM alpha-factor(7)
(c) 48. Expression in response to 0.15,0.5,1.5,5,15.8,50,158,500 nM alpha-factor(8)
(c) 49. Expression in response to 50 nM alpha-factor: 0,15,30,45,60,90,120 min(1)
(c) 49. Expression in response to 50 nM alpha-factor: 0,15,30,45,60,90,120 min(2)
(c) 49. Expression in response to 50 nM alpha-factor: 0,15,30,45,60,90,120 min(3)
(c) 49. Expression in response to 50 nM alpha-factor: 0,15,30,45,60,90,120 min(4)
(c) 49. Expression in response to 50 nM alpha-factor: 0,15,30,45,60,90,120 min(6)
(c) 49. Expression in response to 50 nM alpha-factor: 0,15,30,45,60,90,120 min(7)
(c) 53. Expression in response to overproduction of Ste4p(1)
(c) 56. Expression in response to overproduction of Ste12p(1)
(c) 455. Expression in response to high MNNG (27 microgram/ml) for 60 min(1)
(c) 481. Expression in response to heat shock: 15,30,45,60,120 min(1)
(c) 481. Expression in response to heat shock: 15,30,45,60,120 min(2)
(c) 482. Expression in response to acid: 10,20,40,60,80,100 min(3)
(c) 482. Expression in response to acid: 10,20,40,60,80,100 min(4)
(c) 482. Expression in response to acid: 10,20,40,60,80,100 min(5)
(c) 482. Expression in response to acid: 10,20,40,60,80,100 min(6)

SWI4 -*-> OCH1

(c) 5. Expression during the cell cycle (alpha factor arrest and release)(3)
(c) 5. Expression during the cell cycle (alpha factor arrest and release)(4)
(c) 5. Expression during the cell cycle (alpha factor arrest and release)(5)
(c) 5. Expression during the cell cycle (alpha factor arrest and release)(11)
(c) 5. Expression during the cell cycle (alpha factor arrest and release)(12)
(c) 6. Expression during the cell cycle (cdc15 arrest and release)(12)
(c) 6. Expression during the cell cycle (cdc15 arrest and release)(21)
(c) 7. Expression during the cell Cycle (cdc28)(3)
(c) 7. Expression during the cell Cycle (cdc28)(4)
(c) 7. Expression during the cell Cycle (cdc28)(11)
(c) 7. Expression during the cell Cycle (cdc28)(12)
(c) 482. Expression in response to acid: 10,20,40,60,80,100 min(1)
(c) 482. Expression in response to acid: 10,20,40,60,80,100 min(4)
(c) 482. Expression in response to acid: 10,20,40,60,80,100 min(6)
(c) 483. Expression in response to alkali: 10,20,40,60,80,100 min(6)
(c) 486. Expression in response to NaCl: 15 30 45 60 120 min(4)
(c) 487. Expression in response to sorbitol: 15 30 45 90 120 min(3)
(c) 556. Brown enviromental changes :dtt 480 min dtt-2(1)
(c) wt_plus_gamma_120_min
(c) 100 microM CuSO4 30 min (B)

SWI4 -*-> TOS2

(c) 5. Expression during the cell cycle (alpha factor arrest and release)(3)
(c) 5. Expression during the cell cycle (alpha factor arrest and release)(4)
(c) 5. Expression during the cell cycle (alpha factor arrest and release)(5)
(c) 5. Expression during the cell cycle (alpha factor arrest and release)(11)
(c) 5. Expression during the cell cycle (alpha factor arrest and release)(12)
(c) 6. Expression during the cell cycle (cdc15 arrest and release)(12)
(c) 6. Expression during the cell cycle (cdc15 arrest and release)(21)
(c) 7. Expression during the cell Cycle (cdc28)(3)
(c) 7. Expression during the cell Cycle (cdc28)(4)
(c) 7. Expression during the cell Cycle (cdc28)(11)
(c) 7. Expression during the cell Cycle (cdc28)(12)
(c) 482. Expression in response to acid: 10,20,40,60,80,100 min(1)
(c) 482. Expression in response to acid: 10,20,40,60,80,100 min(4)
(c) 482. Expression in response to acid: 10,20,40,60,80,100 min(6)
(c) 483. Expression in response to alkali: 10,20,40,60,80,100 min(6)
(c) 486. Expression in response to NaCl: 15 30 45 60 120 min(4)
(c) 487. Expression in response to sorbitol: 15 30 45 90 120 min(3)
(c) 556. Brown enviromental changes :dtt 480 min dtt-2(1)
(c) wt_plus_gamma_120_min
(c) 100 microM CuSO4 30 min (B)

SWI4 -*-> YPL267W

(c) 5. Expression during the cell cycle (alpha factor arrest and release)(3)
(c) 5. Expression during the cell cycle (alpha factor arrest and release)(4)
(c) 5. Expression during the cell cycle (alpha factor arrest and release)(5)
(c) 5. Expression during the cell cycle (alpha factor arrest and release)(11)
(c) 5. Expression during the cell cycle (alpha factor arrest and release)(12)
(c) 6. Expression during the cell cycle (cdc15 arrest and release)(12)
(c) 6. Expression during the cell cycle (cdc15 arrest and release)(21)
(c) 7. Expression during the cell Cycle (cdc28)(3)
(c) 7. Expression during the cell Cycle (cdc28)(4)
(c) 7. Expression during the cell Cycle (cdc28)(11)
(c) 7. Expression during the cell Cycle (cdc28)(12)
(c) 482. Expression in response to acid: 10,20,40,60,80,100 min(1)
(c) 482. Expression in response to acid: 10,20,40,60,80,100 min(4)
(c) 482. Expression in response to acid: 10,20,40,60,80,100 min(6)
(c) 483. Expression in response to alkali: 10,20,40,60,80,100 min(6)
(c) 486. Expression in response to NaCl: 15 30 45 60 120 min(4)
(c) 487. Expression in response to sorbitol: 15 30 45 90 120 min(3)
(c) 556. Brown enviromental changes :dtt 480 min dtt-2(1)
(c) wt_plus_gamma_120_min
(c) 100 microM CuSO4 30 min (B)

SWI5 -*-> BUD4

(c) 3. Cell Cycle: Expression in response to Clb2p (set 1, 40 min)(1)
(c) 4. Cell Cycle: Expression in response to Clb2p (set 2, 30 min)(1)
(c) 5. Expression during the cell cycle (alpha factor arrest and release)(9)
(c) 5. Expression during the cell cycle (alpha factor arrest and release)(10)
(c) 5. Expression during the cell cycle (alpha factor arrest and release)(11)
(c) 5. Expression during the cell cycle (alpha factor arrest and release)(12)
(c) 5. Expression during the cell cycle (alpha factor arrest and release)(18)
(c) 6. Expression during the cell cycle (cdc15 arrest and release)(8)
(c) 6. Expression during the cell cycle (cdc15 arrest and release)(9)
(c) 6. Expression during the cell cycle (cdc15 arrest and release)(10)
(c) 6. Expression during the cell cycle (cdc15 arrest and release)(20)
(c) 6. Expression during the cell cycle (cdc15 arrest and release)(21)
(c) 7. Expression during the cell Cycle (cdc28)(9)
(c) 7. Expression during the cell Cycle (cdc28)(10)
(c) 7. Expression during the cell Cycle (cdc28)(11)
(c) 7. Expression during the cell Cycle (cdc28)(16)
(c) 7. Expression during the cell Cycle (cdc28)(17)
(c) 8. Expression during the cell cycle (cell size selection and release)(11)
(c) 8. Expression during the cell cycle (cell size selection and release)(12)
(c) 8. Expression during the cell cycle (cell size selection and release)(13)
(c) 8. Expression during the cell cycle (cell size selection and release)(14)
(c) 556. Brown enviromental changes :dtt 480 min dtt-2(1)
(c) 571. Brown enviromental changes :1M sorbitol - 120 min(1)

SWI5 -*-> CDC20

(c) 3. Cell Cycle: Expression in response to Clb2p (set 1, 40 min)(1)
(c) 4. Cell Cycle: Expression in response to Clb2p (set 2, 30 min)(1)
(c) 5. Expression during the cell cycle (alpha factor arrest and release)(9)
(c) 5. Expression during the cell cycle (alpha factor arrest and release)(10)
(c) 5. Expression during the cell cycle (alpha factor arrest and release)(11)
(c) 5. Expression during the cell cycle (alpha factor arrest and release)(12)
(c) 5. Expression during the cell cycle (alpha factor arrest and release)(18)
(c) 6. Expression during the cell cycle (cdc15 arrest and release)(8)
(c) 6. Expression during the cell cycle (cdc15 arrest and release)(9)
(c) 6. Expression during the cell cycle (cdc15 arrest and release)(10)
(c) 6. Expression during the cell cycle (cdc15 arrest and release)(20)
(c) 6. Expression during the cell cycle (cdc15 arrest and release)(21)
(c) 7. Expression during the cell Cycle (cdc28)(9)
(c) 7. Expression during the cell Cycle (cdc28)(10)
(c) 7. Expression during the cell Cycle (cdc28)(11)
(c) 7. Expression during the cell Cycle (cdc28)(16)
(c) 7. Expression during the cell Cycle (cdc28)(17)
(c) 8. Expression during the cell cycle (cell size selection and release)(11)
(c) 8. Expression during the cell cycle (cell size selection and release)(12)
(c) 8. Expression during the cell cycle (cell size selection and release)(13)
(c) 8. Expression during the cell cycle (cell size selection and release)(14)
(c) 556. Brown enviromental changes :dtt 480 min dtt-2(1)
(c) 571. Brown enviromental changes :1M sorbitol - 120 min(1)

SWI5 -*-> CDC46

(c) 3. Cell Cycle: Expression in response to Clb2p (set 1, 40 min)(1)
(c) 4. Cell Cycle: Expression in response to Clb2p (set 2, 30 min)(1)
(c) 5. Expression during the cell cycle (alpha factor arrest and release)(9)
(c) 5. Expression during the cell cycle (alpha factor arrest and release)(10)
(c) 5. Expression during the cell cycle (alpha factor arrest and release)(11)
(c) 5. Expression during the cell cycle (alpha factor arrest and release)(12)
(c) 5. Expression during the cell cycle (alpha factor arrest and release)(18)
(c) 6. Expression during the cell cycle (cdc15 arrest and release)(8)
(c) 6. Expression during the cell cycle (cdc15 arrest and release)(9)
(c) 6. Expression during the cell cycle (cdc15 arrest and release)(10)
(c) 6. Expression during the cell cycle (cdc15 arrest and release)(20)
(c) 6. Expression during the cell cycle (cdc15 arrest and release)(21)
(c) 7. Expression during the cell Cycle (cdc28)(9)
(c) 7. Expression during the cell Cycle (cdc28)(10)
(c) 7. Expression during the cell Cycle (cdc28)(11)
(c) 7. Expression during the cell Cycle (cdc28)(16)
(c) 7. Expression during the cell Cycle (cdc28)(17)
(c) 8. Expression during the cell cycle (cell size selection and release)(11)
(c) 8. Expression during the cell cycle (cell size selection and release)(12)
(c) 8. Expression during the cell cycle (cell size selection and release)(13)
(c) 8. Expression during the cell cycle (cell size selection and release)(14)
(c) 556. Brown enviromental changes :dtt 480 min dtt-2(1)
(c) 571. Brown enviromental changes :1M sorbitol - 120 min(1)

SWI5 -*-> CDC47

(c) 3. Cell Cycle: Expression in response to Clb2p (set 1, 40 min)(1)
(c) 4. Cell Cycle: Expression in response to Clb2p (set 2, 30 min)(1)
(c) 5. Expression during the cell cycle (alpha factor arrest and release)(9)

(c) 5. Expression during the cell cycle (alpha factor arrest and release)(10)
(c) 5. Expression during the cell cycle (alpha factor arrest and release)(11)
(c) 5. Expression during the cell cycle (alpha factor arrest and release)(12)
(c) 5. Expression during the cell cycle (alpha factor arrest and release)(18)
(c) 6. Expression during the cell cycle (cdc15 arrest and release)(8)
(c) 6. Expression during the cell cycle (cdc15 arrest and release)(9)
(c) 6. Expression during the cell cycle (cdc15 arrest and release)(10)
(c) 6. Expression during the cell cycle (cdc15 arrest and release)(20)
(c) 6. Expression during the cell cycle (cdc15 arrest and release)(21)
(c) 7. Expression during the cell Cycle (cdc28)(9)
(c) 7. Expression during the cell Cycle (cdc28)(10)
(c) 7. Expression during the cell Cycle (cdc28)(11)
(c) 7. Expression during the cell Cycle (cdc28)(16)
(c) 7. Expression during the cell Cycle (cdc28)(17)
(c) 8. Expression during the cell cycle (cell size selection and release)(11)
(c) 8. Expression during the cell cycle (cell size selection and release)(12)
(c) 8. Expression during the cell cycle (cell size selection and release)(13)
(c) 8. Expression during the cell cycle (cell size selection and release)(14)
(c) 556. Brown enviromental changes :dtt 480 min dtt-2(1)
(c) 571. Brown enviromental changes :1M sorbitol - 120 min(1)

SWI5 -*-> CDC5

(c) 3. Cell Cycle: Expression in response to Clb2p (set 1, 40 min)(1)
(c) 4. Cell Cycle: Expression in response to Clb2p (set 2, 30 min)(1)
(c) 5. Expression during the cell cycle (alpha factor arrest and release)(9)
(c) 5. Expression during the cell cycle (alpha factor arrest and release)(10)
(c) 5. Expression during the cell cycle (alpha factor arrest and release)(11)
(c) 5. Expression during the cell cycle (alpha factor arrest and release)(12)
(c) 5. Expression during the cell cycle (alpha factor arrest and release)(18)
(c) 6. Expression during the cell cycle (cdc15 arrest and release)(8)
(c) 6. Expression during the cell cycle (cdc15 arrest and release)(9)
(c) 6. Expression during the cell cycle (cdc15 arrest and release)(10)
(c) 6. Expression during the cell cycle (cdc15 arrest and release)(20)
(c) 6. Expression during the cell cycle (cdc15 arrest and release)(21)
(c) 7. Expression during the cell Cycle (cdc28)(9)
(c) 7. Expression during the cell Cycle (cdc28)(10)
(c) 7. Expression during the cell Cycle (cdc28)(11)
(c) 7. Expression during the cell Cycle (cdc28)(16)
(c) 7. Expression during the cell Cycle (cdc28)(17)
(c) 8. Expression during the cell cycle (cell size selection and release)(11)
(c) 8. Expression during the cell cycle (cell size selection and release)(12)
(c) 8. Expression during the cell cycle (cell size selection and release)(13)
(c) 8. Expression during the cell cycle (cell size selection and release)(14)
(c) 556. Brown enviromental changes :dtt 480 min dtt-2(1)
(c) 571. Brown enviromental changes :1M sorbitol - 120 min(1)

SWI5 -*-> CHS2

(c) 3. Cell Cycle: Expression in response to Clb2p (set 1, 40 min)(1)
(c) 4. Cell Cycle: Expression in response to Clb2p (set 2, 30 min)(1)
(c) 5. Expression during the cell cycle (alpha factor arrest and release)(9)
(c) 5. Expression during the cell cycle (alpha factor arrest and release)(10)
(c) 5. Expression during the cell cycle (alpha factor arrest and release)(11)
(c) 5. Expression during the cell cycle (alpha factor arrest and release)(12)
(c) 5. Expression during the cell cycle (alpha factor arrest and release)(18)
(c) 6. Expression during the cell cycle (cdc15 arrest and release)(8)
(c) 6. Expression during the cell cycle (cdc15 arrest and release)(9)
(c) 6. Expression during the cell cycle (cdc15 arrest and release)(10)
(c) 6. Expression during the cell cycle (cdc15 arrest and release)(20)
(c) 6. Expression during the cell cycle (cdc15 arrest and release)(21)
(c) 7. Expression during the cell Cycle (cdc28)(9)
(c) 7. Expression during the cell Cycle (cdc28)(10)
(c) 7. Expression during the cell Cycle (cdc28)(11)
(c) 7. Expression during the cell Cycle (cdc28)(16)
(c) 7. Expression during the cell Cycle (cdc28)(17)
(c) 8. Expression during the cell cycle (cell size selection and release)(11)
(c) 8. Expression during the cell cycle (cell size selection and release)(12)
(c) 8. Expression during the cell cycle (cell size selection and release)(13)
(c) 8. Expression during the cell cycle (cell size selection and release)(14)
(c) 556. Brown enviromental changes :dtt 480 min dtt-2(1)
(c) 571. Brown enviromental changes :1M sorbitol - 120 min(1)

SWI5 -*-> CLB1

(c) 3. Cell Cycle: Expression in response to Clb2p (set 1, 40 min)(1)
(c) 4. Cell Cycle: Expression in response to Clb2p (set 2, 30 min)(1)
(c) 5. Expression during the cell cycle (alpha factor arrest and release)(9)
(c) 5. Expression during the cell cycle (alpha factor arrest and release)(10)
(c) 5. Expression during the cell cycle (alpha factor arrest and release)(11)
(c) 5. Expression during the cell cycle (alpha factor arrest and release)(12)
(c) 5. Expression during the cell cycle (alpha factor arrest and release)(18)
(c) 6. Expression during the cell cycle (cdc15 arrest and release)(8)
(c) 6. Expression during the cell cycle (cdc15 arrest and release)(9)
(c) 6. Expression during the cell cycle (cdc15 arrest and release)(10)
(c) 6. Expression during the cell cycle (cdc15 arrest and release)(20)
(c) 6. Expression during the cell cycle (cdc15 arrest and release)(21)
(c) 7. Expression during the cell Cycle (cdc28)(9)
(c) 7. Expression during the cell Cycle (cdc28)(10)
(c) 7. Expression during the cell Cycle (cdc28)(11)
(c) 7. Expression during the cell Cycle (cdc28)(16)
(c) 7. Expression during the cell Cycle (cdc28)(17)
(c) 8. Expression during the cell cycle (cell size selection and release)(11)
(c) 8. Expression during the cell cycle (cell size selection and release)(12)
(c) 8. Expression during the cell cycle (cell size selection and release)(13)
(c) 8. Expression during the cell cycle (cell size selection and release)(14)
(c) 556. Brown enviromental changes :dtt 480 min dtt-2(1)
(c) 571. Brown enviromental changes :1M sorbitol - 120 min(1)

SWI5 -*-> FAR1

(c) 3. Cell Cycle: Expression in response to Clb2p (set 1, 40 min)(1)
(c) 4. Cell Cycle: Expression in response to Clb2p (set 2, 30 min)(1)
(c) 5. Expression during the cell cycle (alpha factor arrest and release)(9)

(c) 5. Expression during the cell cycle (alpha factor arrest and release)(10)
(c) 5. Expression during the cell cycle (alpha factor arrest and release)(11)
(c) 5. Expression during the cell cycle (alpha factor arrest and release)(12)
(c) 5. Expression during the cell cycle (alpha factor arrest and release)(18)
(c) 6. Expression during the cell cycle (cdc15 arrest and release)(8)
(c) 6. Expression during the cell cycle (cdc15 arrest and release)(9)
(c) 6. Expression during the cell cycle (cdc15 arrest and release)(10)
(c) 6. Expression during the cell cycle (cdc15 arrest and release)(20)
(c) 6. Expression during the cell cycle (cdc15 arrest and release)(21)
(c) 7. Expression during the cell Cycle (cdc28)(9)
(c) 7. Expression during the cell Cycle (cdc28)(10)
(c) 7. Expression during the cell Cycle (cdc28)(11)
(c) 7. Expression during the cell Cycle (cdc28)(16)
(c) 7. Expression during the cell Cycle (cdc28)(17)
(c) 8. Expression during the cell cycle (cell size selection and release)(11)
(c) 8. Expression during the cell cycle (cell size selection and release)(12)
(c) 8. Expression during the cell cycle (cell size selection and release)(13)
(c) 8. Expression during the cell cycle (cell size selection and release)(14)
(c) 556. Brown enviromental changes :dtt 480 min dtt-2(1)
(c) 571. Brown enviromental changes :1M sorbitol - 120 min(1)

SWI5 -*-> HOF1

(c) 3. Cell Cycle: Expression in response to Clb2p (set 1, 40 min)(1)
(c) 4. Cell Cycle: Expression in response to Clb2p (set 2, 30 min)(1)
(c) 5. Expression during the cell cycle (alpha factor arrest and release)(9)
(c) 5. Expression during the cell cycle (alpha factor arrest and release)(10)
(c) 5. Expression during the cell cycle (alpha factor arrest and release)(11)
(c) 5. Expression during the cell cycle (alpha factor arrest and release)(12)
(c) 5. Expression during the cell cycle (alpha factor arrest and release)(18)
(c) 6. Expression during the cell cycle (cdc15 arrest and release)(8)
(c) 6. Expression during the cell cycle (cdc15 arrest and release)(9)
(c) 6. Expression during the cell cycle (cdc15 arrest and release)(10)
(c) 6. Expression during the cell cycle (cdc15 arrest and release)(20)
(c) 6. Expression during the cell cycle (cdc15 arrest and release)(21)
(c) 7. Expression during the cell Cycle (cdc28)(9)
(c) 7. Expression during the cell Cycle (cdc28)(10)
(c) 7. Expression during the cell Cycle (cdc28)(11)
(c) 7. Expression during the cell Cycle (cdc28)(16)
(c) 7. Expression during the cell Cycle (cdc28)(17)
(c) 8. Expression during the cell cycle (cell size selection and release)(11)
(c) 8. Expression during the cell cycle (cell size selection and release)(12)
(c) 8. Expression during the cell cycle (cell size selection and release)(13)
(c) 8. Expression during the cell cycle (cell size selection and release)(14)
(c) 556. Brown enviromental changes :dtt 480 min dtt-2(1)
(c) 571. Brown enviromental changes :1M sorbitol - 120 min(1)

SWI5 -*-> KIN3

(c) 3. Cell Cycle: Expression in response to Clb2p (set 1, 40 min)(1)
(c) 4. Cell Cycle: Expression in response to Clb2p (set 2, 30 min)(1)
(c) 5. Expression during the cell cycle (alpha factor arrest and release)(9)
(c) 5. Expression during the cell cycle (alpha factor arrest and release)(10)
(c) 5. Expression during the cell cycle (alpha factor arrest and release)(11)
(c) 5. Expression during the cell cycle (alpha factor arrest and release)(12)
(c) 5. Expression during the cell cycle (alpha factor arrest and release)(18)
(c) 6. Expression during the cell cycle (cdc15 arrest and release)(8)
(c) 6. Expression during the cell cycle (cdc15 arrest and release)(9)
(c) 6. Expression during the cell cycle (cdc15 arrest and release)(10)
(c) 6. Expression during the cell cycle (cdc15 arrest and release)(20)
(c) 6. Expression during the cell cycle (cdc15 arrest and release)(21)
(c) 7. Expression during the cell Cycle (cdc28)(9)
(c) 7. Expression during the cell Cycle (cdc28)(10)
(c) 7. Expression during the cell Cycle (cdc28)(11)
(c) 7. Expression during the cell Cycle (cdc28)(16)
(c) 7. Expression during the cell Cycle (cdc28)(17)
(c) 8. Expression during the cell cycle (cell size selection and release)(11)
(c) 8. Expression during the cell cycle (cell size selection and release)(12)
(c) 8. Expression during the cell cycle (cell size selection and release)(13)
(c) 8. Expression during the cell cycle (cell size selection and release)(14)
(c) 556. Brown enviromental changes :dtt 480 min dtt-2(1)
(c) 571. Brown enviromental changes :1M sorbitol - 120 min(1)

SWI5 -*-> MRH1

(c) 3. Cell Cycle: Expression in response to Clb2p (set 1, 40 min)(1)
(c) 4. Cell Cycle: Expression in response to Clb2p (set 2, 30 min)(1)
(c) 5. Expression during the cell cycle (alpha factor arrest and release)(9)
(c) 5. Expression during the cell cycle (alpha factor arrest and release)(10)
(c) 5. Expression during the cell cycle (alpha factor arrest and release)(11)
(c) 5. Expression during the cell cycle (alpha factor arrest and release)(12)
(c) 5. Expression during the cell cycle (alpha factor arrest and release)(18)
(c) 6. Expression during the cell cycle (cdc15 arrest and release)(8)
(c) 6. Expression during the cell cycle (cdc15 arrest and release)(9)
(c) 6. Expression during the cell cycle (cdc15 arrest and release)(10)
(c) 6. Expression during the cell cycle (cdc15 arrest and release)(20)
(c) 6. Expression during the cell cycle (cdc15 arrest and release)(21)
(c) 7. Expression during the cell Cycle (cdc28)(9)
(c) 7. Expression during the cell Cycle (cdc28)(10)
(c) 7. Expression during the cell Cycle (cdc28)(11)
(c) 7. Expression during the cell Cycle (cdc28)(16)
(c) 7. Expression during the cell Cycle (cdc28)(17)
(c) 8. Expression during the cell cycle (cell size selection and release)(11)
(c) 8. Expression during the cell cycle (cell size selection and release)(12)
(c) 8. Expression during the cell cycle (cell size selection and release)(13)
(c) 8. Expression during the cell cycle (cell size selection and release)(14)
(c) 556. Brown enviromental changes :dtt 480 min dtt-2(1)
(c) 571. Brown enviromental changes :1M sorbitol - 120 min(1)

SWI5 -*-> MYO1

(c) 3. Cell Cycle: Expression in response to Clb2p (set 1, 40 min)(1)
(c) 4. Cell Cycle: Expression in response to Clb2p (set 2, 30 min)(1)
(c) 5. Expression during the cell cycle (alpha factor arrest and release)(9)

```
    (c) 5. Expression during the cell cycle (alpha factor arrest and release)(10)
    (c) 5. Expression during the cell cycle (alpha factor arrest and release)(11)
    (c) 5. Expression during the cell cycle (alpha factor arrest and release)(12)
    (c) 5. Expression during the cell cycle (alpha factor arrest and release)(18)
    (c) 6. Expression during the cell cycle (cdc15 arrest and release)(8)
    (c) 6. Expression during the cell cycle (cdc15 arrest and release)(9)
    (c) 6. Expression during the cell cycle (cdc15 arrest and release)(10)
    (c) 6. Expression during the cell cycle (cdc15 arrest and release)(20)
    (c) 6. Expression during the cell cycle (cdc15 arrest and release)(21)
    (c) 7. Expression during the cell Cycle (cdc28)(9)
    (c) 7. Expression during the cell Cycle (cdc28)(10)
    (c) 7. Expression during the cell Cycle (cdc28)(11)
    (c) 7. Expression during the cell Cycle (cdc28)(16)
    (c) 7. Expression during the cell Cycle (cdc28)(17)
    (c) 8. Expression during the cell cycle (cell size selection and release)(11)
    (c) 8. Expression during the cell cycle (cell size selection and release)(12)
    (c) 8. Expression during the cell cycle (cell size selection and release)(13)
    (c) 8. Expression during the cell cycle (cell size selection and release)(14)
    (c) 556. Brown enviromental changes :dtt 480 min dtt-2(1)
    (c) 571. Brown enviromental changes :1M sorbitol - 120 min(1)


SWI5 -*-> PHO3


    (c) 3. Cell Cycle: Expression in response to Clb2p (set 1, 40 min)(1)
    (c) 4. Cell Cycle: Expression in response to Clb2p (set 2, 30 min)(1)
    (c) 5. Expression during the cell cycle (alpha factor arrest and release)(9)
    (c) 5. Expression during the cell cycle (alpha factor arrest and release)(10)
    (c) 5. Expression during the cell cycle (alpha factor arrest and release)(11)
    (c) 5. Expression during the cell cycle (alpha factor arrest and release)(12)
    (c) 5. Expression during the cell cycle (alpha factor arrest and release)(18)
    (c) 6. Expression during the cell cycle (cdc15 arrest and release)(8)
    (c) 6. Expression during the cell cycle (cdc15 arrest and release)(9)
    (c) 6. Expression during the cell cycle (cdc15 arrest and release)(10)
    (c) 6. Expression during the cell cycle (cdc15 arrest and release)(20)
    (c) 6. Expression during the cell cycle (cdc15 arrest and release)(21)
    (c) 7. Expression during the cell Cycle (cdc28)(9)
    (c) 7. Expression during the cell Cycle (cdc28)(10)
    (c) 7. Expression during the cell Cycle (cdc28)(11)
    (c) 7. Expression during the cell Cycle (cdc28)(16)
    (c) 7. Expression during the cell Cycle (cdc28)(17)
    (c) 8. Expression during the cell cycle (cell size selection and release)(11)
    (c) 8. Expression during the cell cycle (cell size selection and release)(12)
    (c) 8. Expression during the cell cycle (cell size selection and release)(13)
    (c) 8. Expression during the cell cycle (cell size selection and release)(14)
    (c) 556. Brown enviromental changes :dtt 480 min dtt-2(1)
    (c) 571. Brown enviromental changes :1M sorbitol - 120 min(1)


SWI5 -*-> YJL051W


    (c) 3. Cell Cycle: Expression in response to Clb2p (set 1, 40 min)(1)
    (c) 4. Cell Cycle: Expression in response to Clb2p (set 2, 30 min)(1)
    (c) 5. Expression during the cell cycle (alpha factor arrest and release)(9)
    (c) 5. Expression during the cell cycle (alpha factor arrest and release)(10)
    (c) 5. Expression during the cell cycle (alpha factor arrest and release)(11)
    (c) 5. Expression during the cell cycle (alpha factor arrest and release)(12)
    (c) 5. Expression during the cell cycle (alpha factor arrest and release)(18)
    (c) 6. Expression during the cell cycle (cdc15 arrest and release)(8)
    (c) 6. Expression during the cell cycle (cdc15 arrest and release)(9)
    (c) 6. Expression during the cell cycle (cdc15 arrest and release)(10)
    (c) 6. Expression during the cell cycle (cdc15 arrest and release)(20)
    (c) 6. Expression during the cell cycle (cdc15 arrest and release)(21)
    (c) 7. Expression during the cell Cycle (cdc28)(9)
    (c) 7. Expression during the cell Cycle (cdc28)(10)
    (c) 7. Expression during the cell Cycle (cdc28)(11)
    (c) 7. Expression during the cell Cycle (cdc28)(16)
    (c) 7. Expression during the cell Cycle (cdc28)(17)
    (c) 8. Expression during the cell cycle (cell size selection and release)(11)
    (c) 8. Expression during the cell cycle (cell size selection and release)(12)
    (c) 8. Expression during the cell cycle (cell size selection and release)(13)
    (c) 8. Expression during the cell cycle (cell size selection and release)(14)
    (c) 556. Brown enviromental changes :dtt 480 min dtt-2(1)
    (c) 571. Brown enviromental changes :1M sorbitol - 120 min(1)


SWI5 -*-> YLR190W


    (c) 3. Cell Cycle: Expression in response to Clb2p (set 1, 40 min)(1)
    (c) 4. Cell Cycle: Expression in response to Clb2p (set 2, 30 min)(1)
    (c) 5. Expression during the cell cycle (alpha factor arrest and release)(9)
    (c) 5. Expression during the cell cycle (alpha factor arrest and release)(10)
    (c) 5. Expression during the cell cycle (alpha factor arrest and release)(11)
    (c) 5. Expression during the cell cycle (alpha factor arrest and release)(12)
    (c) 5. Expression during the cell cycle (alpha factor arrest and release)(18)
    (c) 6. Expression during the cell cycle (cdc15 arrest and release)(8)
    (c) 6. Expression during the cell cycle (cdc15 arrest and release)(9)
    (c) 6. Expression during the cell cycle (cdc15 arrest and release)(10)
    (c) 6. Expression during the cell cycle (cdc15 arrest and release)(20)
    (c) 6. Expression during the cell cycle (cdc15 arrest and release)(21)
    (c) 7. Expression during the cell Cycle (cdc28)(9)
    (c) 7. Expression during the cell Cycle (cdc28)(10)
    (c) 7. Expression during the cell Cycle (cdc28)(11)
    (c) 7. Expression during the cell Cycle (cdc28)(16)
    (c) 7. Expression during the cell Cycle (cdc28)(17)
    (c) 8. Expression during the cell cycle (cell size selection and release)(11)
    (c) 8. Expression during the cell cycle (cell size selection and release)(12)
    (c) 8. Expression during the cell cycle (cell size selection and release)(13)
    (c) 8. Expression during the cell cycle (cell size selection and release)(14)
    (c) 556. Brown enviromental changes :dtt 480 min dtt-2(1)
    (c) 571. Brown enviromental changes :1M sorbitol - 120 min(1)


SWI5 -*-> YML119W


    (c) 3. Cell Cycle: Expression in response to Clb2p (set 1, 40 min)(1)
    (c) 4. Cell Cycle: Expression in response to Clb2p (set 2, 30 min)(1)
    (c) 5. Expression during the cell cycle (alpha factor arrest and release)(9)
```

(c) 5. Expression during the cell cycle (alpha factor arrest and release)(10)
(c) 5. Expression during the cell cycle (alpha factor arrest and release)(11)
(c) 5. Expression during the cell cycle (alpha factor arrest and release)(12)
(c) 5. Expression during the cell cycle (alpha factor arrest and release)(18)
(c) 6. Expression during the cell cycle (cdc15 arrest and release)(8)
(c) 6. Expression during the cell cycle (cdc15 arrest and release)(9)
(c) 6. Expression during the cell cycle (cdc15 arrest and release)(10)
(c) 6. Expression during the cell cycle (cdc15 arrest and release)(20)
(c) 6. Expression during the cell cycle (cdc15 arrest and release)(21)
(c) 7. Expression during the cell Cycle (cdc28)(9)
(c) 7. Expression during the cell Cycle (cdc28)(10)
(c) 7. Expression during the cell Cycle (cdc28)(11)
(c) 7. Expression during the cell Cycle (cdc28)(16)
(c) 7. Expression during the cell Cycle (cdc28)(17)
(c) 8. Expression during the cell cycle (cell size selection and release)(11)
(c) 8. Expression during the cell cycle (cell size selection and release)(12)
(c) 8. Expression during the cell cycle (cell size selection and release)(13)
(c) 8. Expression during the cell cycle (cell size selection and release)(14)
(c) 556. Brown enviromental changes :dtt 480 min dtt-2(1)
(c) 571. Brown enviromental changes :1M sorbitol - 120 min(1)

SWI5 -*-> YNL057W

(c) 3. Cell Cycle: Expression in response to Clb2p (set 1, 40 min)(1)
(c) 4. Cell Cycle: Expression in response to Clb2p (set 2, 30 min)(1)
(c) 5. Expression during the cell cycle (alpha factor arrest and release)(9)
(c) 5. Expression during the cell cycle (alpha factor arrest and release)(10)
(c) 5. Expression during the cell cycle (alpha factor arrest and release)(11)
(c) 5. Expression during the cell cycle (alpha factor arrest and release)(12)
(c) 5. Expression during the cell cycle (alpha factor arrest and release)(18)
(c) 6. Expression during the cell cycle (cdc15 arrest and release)(8)
(c) 6. Expression during the cell cycle (cdc15 arrest and release)(9)
(c) 6. Expression during the cell cycle (cdc15 arrest and release)(10)
(c) 6. Expression during the cell cycle (cdc15 arrest and release)(20)
(c) 6. Expression during the cell cycle (cdc15 arrest and release)(21)
(c) 7. Expression during the cell Cycle (cdc28)(9)
(c) 7. Expression during the cell Cycle (cdc28)(10)
(c) 7. Expression during the cell Cycle (cdc28)(11)
(c) 7. Expression during the cell Cycle (cdc28)(16)
(c) 7. Expression during the cell Cycle (cdc28)(17)
(c) 8. Expression during the cell cycle (cell size selection and release)(11)
(c) 8. Expression during the cell cycle (cell size selection and release)(12)
(c) 8. Expression during the cell cycle (cell size selection and release)(13)
(c) 8. Expression during the cell cycle (cell size selection and release)(14)
(c) 556. Brown enviromental changes :dtt 480 min dtt-2(1)
(c) 571. Brown enviromental changes :1M sorbitol - 120 min(1)

SWI5 -*-> YNL058C

(c) 3. Cell Cycle: Expression in response to Clb2p (set 1, 40 min)(1)
(c) 4. Cell Cycle: Expression in response to Clb2p (set 2, 30 min)(1)
(c) 5. Expression during the cell cycle (alpha factor arrest and release)(9)
(c) 5. Expression during the cell cycle (alpha factor arrest and release)(10)
(c) 5. Expression during the cell cycle (alpha factor arrest and release)(11)
(c) 5. Expression during the cell cycle (alpha factor arrest and release)(12)
(c) 5. Expression during the cell cycle (alpha factor arrest and release)(18)
(c) 6. Expression during the cell cycle (cdc15 arrest and release)(8)
(c) 6. Expression during the cell cycle (cdc15 arrest and release)(9)
(c) 6. Expression during the cell cycle (cdc15 arrest and release)(10)
(c) 6. Expression during the cell cycle (cdc15 arrest and release)(20)
(c) 6. Expression during the cell cycle (cdc15 arrest and release)(21)
(c) 7. Expression during the cell Cycle (cdc28)(9)
(c) 7. Expression during the cell Cycle (cdc28)(10)
(c) 7. Expression during the cell Cycle (cdc28)(11)
(c) 7. Expression during the cell Cycle (cdc28)(16)
(c) 7. Expression during the cell Cycle (cdc28)(17)
(c) 8. Expression during the cell cycle (cell size selection and release)(11)
(c) 8. Expression during the cell cycle (cell size selection and release)(12)
(c) 8. Expression during the cell cycle (cell size selection and release)(13)
(c) 8. Expression during the cell cycle (cell size selection and release)(14)
(c) 556. Brown enviromental changes :dtt 480 min dtt-2(1)
(c) 571. Brown enviromental changes :1M sorbitol - 120 min(1)

SWI5 -*-> YOL070C

(c) 3. Cell Cycle: Expression in response to Clb2p (set 1, 40 min)(1)
(c) 4. Cell Cycle: Expression in response to Clb2p (set 2, 30 min)(1)
(c) 5. Expression during the cell cycle (alpha factor arrest and release)(9)
(c) 5. Expression during the cell cycle (alpha factor arrest and release)(10)
(c) 5. Expression during the cell cycle (alpha factor arrest and release)(11)
(c) 5. Expression during the cell cycle (alpha factor arrest and release)(12)
(c) 5. Expression during the cell cycle (alpha factor arrest and release)(18)
(c) 6. Expression during the cell cycle (cdc15 arrest and release)(8)
(c) 6. Expression during the cell cycle (cdc15 arrest and release)(9)
(c) 6. Expression during the cell cycle (cdc15 arrest and release)(10)
(c) 6. Expression during the cell cycle (cdc15 arrest and release)(20)
(c) 6. Expression during the cell cycle (cdc15 arrest and release)(21)
(c) 7. Expression during the cell Cycle (cdc28)(9)
(c) 7. Expression during the cell Cycle (cdc28)(10)
(c) 7. Expression during the cell Cycle (cdc28)(11)
(c) 7. Expression during the cell Cycle (cdc28)(16)
(c) 7. Expression during the cell Cycle (cdc28)(17)
(c) 8. Expression during the cell cycle (cell size selection and release)(11)
(c) 8. Expression during the cell cycle (cell size selection and release)(12)
(c) 8. Expression during the cell cycle (cell size selection and release)(13)
(c) 8. Expression during the cell cycle (cell size selection and release)(14)
(c) 556. Brown enviromental changes :dtt 480 min dtt-2(1)
(c) 571. Brown enviromental changes :1M sorbitol - 120 min(1)

SWI5 -*-> YOR315W

(c) 3. Cell Cycle: Expression in response to Clb2p (set 1, 40 min)(1)
(c) 4. Cell Cycle: Expression in response to Clb2p (set 2, 30 min)(1)
(c) 5. Expression during the cell cycle (alpha factor arrest and release)(9)

```
    (c) 5. Expression during the cell cycle (alpha factor arrest and release)(10)
    (c) 5. Expression during the cell cycle (alpha factor arrest and release)(11)
    (c) 5. Expression during the cell cycle (alpha factor arrest and release)(12)
    (c) 5. Expression during the cell cycle (alpha factor arrest and release)(18)
    (c) 6. Expression during the cell cycle (cdc15 arrest and release)(8)
    (c) 6. Expression during the cell cycle (cdc15 arrest and release)(9)
    (c) 6. Expression during the cell cycle (cdc15 arrest and release)(10)
    (c) 6. Expression during the cell cycle (cdc15 arrest and release)(20)
    (c) 6. Expression during the cell cycle (cdc15 arrest and release)(21)
    (c) 7. Expression during the cell Cycle (cdc28)(9)
    (c) 7. Expression during the cell Cycle (cdc28)(10)
    (c) 7. Expression during the cell Cycle (cdc28)(11)
    (c) 7. Expression during the cell Cycle (cdc28)(16)
    (c) 7. Expression during the cell Cycle (cdc28)(17)
    (c) 8. Expression during the cell cycle (cell size selection and release)(11)
    (c) 8. Expression during the cell cycle (cell size selection and release)(12)
    (c) 8. Expression during the cell cycle (cell size selection and release)(13)
    (c) 8. Expression during the cell cycle (cell size selection and release)(14)
    (c) 556. Brown enviromental changes :dtt 480 min dtt-2(1)
    (c) 571. Brown enviromental changes :1M sorbitol - 120 min(1)


SWI5 -*-> YPL158C


    (c) 3. Cell Cycle: Expression in response to Clb2p (set 1, 40 min)(1)
    (c) 4. Cell Cycle: Expression in response to Clb2p (set 2, 30 min)(1)
    (c) 5. Expression during the cell cycle (alpha factor arrest and release)(9)
    (c) 5. Expression during the cell cycle (alpha factor arrest and release)(10)
    (c) 5. Expression during the cell cycle (alpha factor arrest and release)(11)
    (c) 5. Expression during the cell cycle (alpha factor arrest and release)(12)
    (c) 5. Expression during the cell cycle (alpha factor arrest and release)(18)
    (c) 6. Expression during the cell cycle (cdc15 arrest and release)(8)
    (c) 6. Expression during the cell cycle (cdc15 arrest and release)(9)
    (c) 6. Expression during the cell cycle (cdc15 arrest and release)(10)
    (c) 6. Expression during the cell cycle (cdc15 arrest and release)(20)
    (c) 6. Expression during the cell cycle (cdc15 arrest and release)(21)
    (c) 7. Expression during the cell Cycle (cdc28)(9)
    (c) 7. Expression during the cell Cycle (cdc28)(10)
    (c) 7. Expression during the cell Cycle (cdc28)(11)
    (c) 7. Expression during the cell Cycle (cdc28)(16)
    (c) 7. Expression during the cell Cycle (cdc28)(17)
    (c) 8. Expression during the cell cycle (cell size selection and release)(11)
    (c) 8. Expression during the cell cycle (cell size selection and release)(12)
    (c) 8. Expression during the cell cycle (cell size selection and release)(13)
    (c) 8. Expression during the cell cycle (cell size selection and release)(14)
    (c) 556. Brown enviromental changes :dtt 480 min dtt-2(1)
    (c) 571. Brown enviromental changes :1M sorbitol - 120 min(1)


SWI5 -*-> YRO2


    (c) 3. Cell Cycle: Expression in response to Clb2p (set 1, 40 min)(1)
    (c) 4. Cell Cycle: Expression in response to Clb2p (set 2, 30 min)(1)
    (c) 5. Expression during the cell cycle (alpha factor arrest and release)(9)
    (c) 5. Expression during the cell cycle (alpha factor arrest and release)(10)
    (c) 5. Expression during the cell cycle (alpha factor arrest and release)(11)
    (c) 5. Expression during the cell cycle (alpha factor arrest and release)(12)
    (c) 5. Expression during the cell cycle (alpha factor arrest and release)(18)
    (c) 6. Expression during the cell cycle (cdc15 arrest and release)(8)
    (c) 6. Expression during the cell cycle (cdc15 arrest and release)(9)
    (c) 6. Expression during the cell cycle (cdc15 arrest and release)(10)
    (c) 6. Expression during the cell cycle (cdc15 arrest and release)(20)
    (c) 6. Expression during the cell cycle (cdc15 arrest and release)(21)
    (c) 7. Expression during the cell Cycle (cdc28)(9)
    (c) 7. Expression during the cell Cycle (cdc28)(10)
    (c) 7. Expression during the cell Cycle (cdc28)(11)
    (c) 7. Expression during the cell Cycle (cdc28)(16)
    (c) 7. Expression during the cell Cycle (cdc28)(17)
    (c) 8. Expression during the cell cycle (cell size selection and release)(11)
    (c) 8. Expression during the cell cycle (cell size selection and release)(12)
    (c) 8. Expression during the cell cycle (cell size selection and release)(13)
    (c) 8. Expression during the cell cycle (cell size selection and release)(14)
    (c) 556. Brown enviromental changes :dtt 480 min dtt-2(1)
    (c) 571. Brown enviromental changes :1M sorbitol - 120 min(1)


SWI5 -*-| YKR012C


    (c) 3. Cell Cycle: Expression in response to Clb2p (set 1, 40 min)(1)
    (c) 4. Cell Cycle: Expression in response to Clb2p (set 2, 30 min)(1)
    (c) 5. Expression during the cell cycle (alpha factor arrest and release)(8)
    (c) 5. Expression during the cell cycle (alpha factor arrest and release)(9)
    (c) 5. Expression during the cell cycle (alpha factor arrest and release)(10)
    (c) 5. Expression during the cell cycle (alpha factor arrest and release)(11)
    (c) 5. Expression during the cell cycle (alpha factor arrest and release)(17)
    (c) 5. Expression during the cell cycle (alpha factor arrest and release)(18)
    (c) 6. Expression during the cell cycle (cdc15 arrest and release)(1)
    (c) 6. Expression during the cell cycle (cdc15 arrest and release)(8)
    (c) 6. Expression during the cell cycle (cdc15 arrest and release)(20)
    (c) 7. Expression during the cell Cycle (cdc28)(9)
    (c) 7. Expression during the cell Cycle (cdc28)(15)
    (c) 7. Expression during the cell Cycle (cdc28)(16)
    (c) 7. Expression during the cell Cycle (cdc28)(17)
    (c) 8. Expression during the cell cycle (cell size selection and release)(10)
    (c) 8. Expression during the cell cycle (cell size selection and release)(11)
    (c) 8. Expression during the cell cycle (cell size selection and release)(12)
    (c) 8. Expression during the cell cycle (cell size selection and release)(14)
    (c) 500. Brown enviromental changes :Heat Shock 015 minutes  hs-2(1)
    (c) 505. Brown enviromental changes :37C to 25C shock - 45 min(1)
    (c) 548. Brown enviromental changes :2.5mM DTT 120 min dtt-1(1)
    (c) 574. Brown enviromental changes :Hypo-osmotic shock - 30 min(1)
    (c) 575. Brown enviromental changes :Hypo-osmotic shock - 45 min(1)
    (c) 576. Brown enviromental changes :Hypo-osmotic shock - 60 min(1)
    (c) wt_plus_gamma_20_min
    (c) DES460 (wt) - mock irradiation - 5 min


SWI5 -*-> SUN4
```

```
    (c) 3. Cell Cycle: Expression in response to Clb2p (set 1, 40 min)(1)
    (c) 4. Cell Cycle: Expression in response to Clb2p (set 2, 30 min)(1)
    (c) 5. Expression during the cell cycle (alpha factor arrest and release)(12)
    (c) 5. Expression during the cell cycle (alpha factor arrest and release)(14)
    (c) 5. Expression during the cell cycle (alpha factor arrest and release)(15)
    (c) 5. Expression during the cell cycle (alpha factor arrest and release)(16)
    (c) 5. Expression during the cell cycle (alpha factor arrest and release)(17)
    (c) 5. Expression during the cell cycle (alpha factor arrest and release)(18)
    (c) 6. Expression during the cell cycle (cdc15 arrest and release)(13)
    (c) 6. Expression during the cell cycle (cdc15 arrest and release)(20)
    (c) 6. Expression during the cell cycle (cdc15 arrest and release)(21)
    (c) 7. Expression during the cell Cycle (cdc28)(11)
    (c) 7. Expression during the cell Cycle (cdc28)(15)
    (c) 7. Expression during the cell Cycle (cdc28)(16)
    (c) 8. Expression during the cell cycle (cell size selection and release)(3)
    (c) 8. Expression during the cell cycle (cell size selection and release)(11)
    (c) 8. Expression during the cell cycle (cell size selection and release)(12)
    (c) 8. Expression during the cell cycle (cell size selection and release)(13)
    (c) 8. Expression during the cell cycle (cell size selection and release)(14)
    (c) 11. Expression during diauxic shift: 9h,11h,13h,15h,17h,19h,21h(3)
    (c) 26. Fink: Expression in diploid high copy TEC1(1)
    (c) 571. Brown enviromental changes :1M sorbitol - 120 min(1)
    (c) 595. Brown enviromental changes :diauxic shift timecourse(1)


SWI5 -*-> YOR264W


    (c) 3. Cell Cycle: Expression in response to Clb2p (set 1, 40 min)(1)
    (c) 4. Cell Cycle: Expression in response to Clb2p (set 2, 30 min)(1)
    (c) 5. Expression during the cell cycle (alpha factor arrest and release)(10)
    (c) 5. Expression during the cell cycle (alpha factor arrest and release)(11)
    (c) 5. Expression during the cell cycle (alpha factor arrest and release)(12)
    (c) 5. Expression during the cell cycle (alpha factor arrest and release)(16)
    (c) 5. Expression during the cell cycle (alpha factor arrest and release)(18)
    (c) 6. Expression during the cell cycle (cdc15 arrest and release)(1)
    (c) 6. Expression during the cell cycle (cdc15 arrest and release)(10)
    (c) 6. Expression during the cell cycle (cdc15 arrest and release)(20)
    (c) 6. Expression during the cell cycle (cdc15 arrest and release)(22)
    (c) 7. Expression during the cell Cycle (cdc28)(9)
    (c) 7. Expression during the cell Cycle (cdc28)(10)
    (c) 7. Expression during the cell Cycle (cdc28)(11)
    (c) 7. Expression during the cell Cycle (cdc28)(16)
    (c) 7. Expression during the cell Cycle (cdc28)(17)
    (c) 8. Expression during the cell cycle (cell size selection and release)(11)
    (c) 8. Expression during the cell cycle (cell size selection and release)(12)
    (c) 8. Expression during the cell cycle (cell size selection and release)(13)
    (c) 8. Expression during the cell cycle (cell size selection and release)(14)
    (c) 487. Expression in response to sorbitol: 15 30 45 90 120 min(5)


TEC1 -*-> GIC2


    (c) 5. Expression during the cell cycle (alpha factor arrest and release)(11)
    (c) 5. Expression during the cell cycle (alpha factor arrest and release)(12)
    (c) 5. Expression during the cell cycle (alpha factor arrest and release)(13)
    (c) 5. Expression during the cell cycle (alpha factor arrest and release)(15)
    (c) 6. Expression during the cell cycle (cdc15 arrest and release)(2)
    (c) 6. Expression during the cell cycle (cdc15 arrest and release)(10)
    (c) 6. Expression during the cell cycle (cdc15 arrest and release)(11)
    (c) 6. Expression during the cell cycle (cdc15 arrest and release)(17)
    (c) 6. Expression during the cell cycle (cdc15 arrest and release)(20)
    (c) 6. Expression during the cell cycle (cdc15 arrest and release)(22)
    (c) 7. Expression during the cell Cycle (cdc28)(10)
    (c) 7. Expression during the cell Cycle (cdc28)(11)
    (c) 7. Expression during the cell Cycle (cdc28)(12)
    (c) 8. Expression during the cell cycle (cell size selection and release)(9)
    (c) 8. Expression during the cell cycle (cell size selection and release)(10)
    (c) 8. Expression during the cell cycle (cell size selection and release)(12)
    (c) 26. Fink: Expression in diploid high copy TEC1(1)
    (c) 49. Expression in response to 50 nM alpha-factor: 0,15,30,45,60,90,120 min(1)
    (c) 523. Brown enviromental changes :constant 0.32 mM H2O2 (10 min) redo(1)
    (c) (Var.) Rich Media 2% Glucose YPD-Average wt  5mM aF, 30 min.
    (c) DES460 (wt) - mock irradiation - 30 min
    (c) 100 microM BCS 60 min


TEC1 -*-> YLR049C


    (c) 5. Expression during the cell cycle (alpha factor arrest and release)(11)
    (c) 5. Expression during the cell cycle (alpha factor arrest and release)(12)
    (c) 5. Expression during the cell cycle (alpha factor arrest and release)(13)
    (c) 5. Expression during the cell cycle (alpha factor arrest and release)(15)
    (c) 6. Expression during the cell cycle (cdc15 arrest and release)(2)
    (c) 6. Expression during the cell cycle (cdc15 arrest and release)(10)
    (c) 6. Expression during the cell cycle (cdc15 arrest and release)(11)
    (c) 6. Expression during the cell cycle (cdc15 arrest and release)(17)
    (c) 6. Expression during the cell cycle (cdc15 arrest and release)(20)
    (c) 6. Expression during the cell cycle (cdc15 arrest and release)(22)
    (c) 7. Expression during the cell Cycle (cdc28)(10)
    (c) 7. Expression during the cell Cycle (cdc28)(11)
    (c) 7. Expression during the cell Cycle (cdc28)(12)
    (c) 8. Expression during the cell cycle (cell size selection and release)(9)
    (c) 8. Expression during the cell cycle (cell size selection and release)(10)
    (c) 8. Expression during the cell cycle (cell size selection and release)(12)
    (c) 26. Fink: Expression in diploid high copy TEC1(1)
    (c) 49. Expression in response to 50 nM alpha-factor: 0,15,30,45,60,90,120 min(1)
    (c) 523. Brown enviromental changes :constant 0.32 mM H2O2 (10 min) redo(1)
    (c) (Var.) Rich Media 2% Glucose YPD-Average wt  5mM aF, 30 min.
    (c) DES460 (wt) - mock irradiation - 30 min
    (c) 100 microM BCS 60 min


TEC1 -*-> FAR1


    (c) 6. Expression during the cell cycle (cdc15 arrest and release)(14)
    (c) 6. Expression during the cell cycle (cdc15 arrest and release)(20)
    (c) 6. Expression during the cell cycle (cdc15 arrest and release)(22)
```

(c) 6. Expression during the cell cycle (cdc15 arrest and release)(23)
(c) 7. Expression during the cell Cycle (cdc28)(10)
(c) 7. Expression during the cell Cycle (cdc28)(16)
(c) 7. Expression during the cell Cycle (cdc28)(17)
(c) 8. Expression during the cell cycle (cell size selection and release)(12)
(c) 26. Fink: Expression in diploid high copy TEC1(1)
(c) 48. Expression in response to 0.15,0.5,1.5,5,15.8,50,158,500 nM alpha-factor(5)
(c) 49. Expression in response to 50 nM alpha-factor: 0,15,30,45,60,90,120 min(1)
(c) 396. Rosetta 2000: Expression in response to Calcofluor white(1)
(c) 405. Rosetta 2000: Expression in response to Nikkomycin Z(1)
(c) 477. Expression in response to trichostatin A (TSA): 15min,30min,60min,120min(1)
(c) 482. Expression in response to acid: 10,20,40,60,80,100 min(6)
(c) 523. Brown enviromental changes :constant 0.32 mM H2O2 (10 min) redo(1)
(c) (Rich Media 2% Glucose YPD-185588) wt 5mM aF, 30 min.
(c) (Rich Media 2% Glucose YPD-185769) wt 5mM aF, 30 min.
(c) Rich Media 2% Glucose YPD-Average wt 5mM aF, 30 min.
(c) DES460 (wt) - mock irradiation - 5 min
(c) DES460 (wt) - mock irradiation - 30 min
(c) MHY1 (crt1) vs CRY1 (wild type)
(c) 100 microM BCS 30 min
(c) 100 microM BCS 60 min

TEC1 -*-> RGD2

(c) 5. Expression during the cell cycle (alpha factor arrest and release)(13)
(c) 6. Expression during the cell cycle (cdc15 arrest and release)(2)
(c) 6. Expression during the cell cycle (cdc15 arrest and release)(20)
(c) 7. Expression during the cell Cycle (cdc28)(10)
(c) 7. Expression during the cell Cycle (cdc28)(12)
(c) 7. Expression during the cell Cycle (cdc28)(15)
(c) 8. Expression during the cell cycle (cell size selection and release)(3)
(c) 26. Fink: Expression in diploid high copy TEC1(1)
(c) 49. Expression in response to 50 nM alpha-factor: 0,15,30,45,60,90,120 min(1)
(c) 49. Expression in response to 50 nM alpha-factor: 0,15,30,45,60,90,120 min(2)
(c) 56. Expression in response to overproduction of Ste12p(1)
(c) 523. Brown enviromental changes :constant 0.32 mM H2O2 (10 min) redo(1)
(c) (Rich Media 2% Glucose YPD-185588) wt 5mM aF, 30 min.
(c) (Rich Media 2% Glucose YPD-185769) wt 5mM aF, 30 min.
(c) Rich Media 2% Glucose YPD-Average wt 5mM aF, 30 min.
(c) MAC1-up (B)

TEC1 -*-> ASG7

(c) 5. Expression during the cell cycle (alpha factor arrest and release)(1)
(c) 7. Expression during the cell Cycle (cdc28)(17)
(c) 26. Fink: Expression in diploid high copy TEC1(1)
(c) 48. Expression in response to 0.15,0.5,1.5,5,15.8,50,158,500 nM alpha-factor(4)
(c) 48. Expression in response to 0.15,0.5,1.5,5,15.8,50,158,500 nM alpha-factor(5)
(c) 48. Expression in response to 0.15,0.5,1.5,5,15.8,50,158,500 nM alpha-factor(8)
(c) 49. Expression in response to 50 nM alpha-factor: 0,15,30,45,60,90,120 min(1)
(c) 49. Expression in response to 50 nM alpha-factor: 0,15,30,45,60,90,120 min(3)
(c) 49. Expression in response to 50 nM alpha-factor: 0,15,30,45,60,90,120 min(4)
(c) 49. Expression in response to 50 nM alpha-factor: 0,15,30,45,60,90,120 min(6)
(c) 49. Expression in response to 50 nM alpha-factor: 0,15,30,45,60,90,120 min(7)
(c) 53. Expression in response to overproduction of Ste4p(1)
(c) 54. Expression in response to overproduction of Ste5p(1)
(c) 55. Expression in response to overproduction of Ste11p(1)
(c) 74. Expression in response to overproduction of activated Rho1p(1)
(c) 388. Rosetta 2000: Expression in cells with FKS1 under tet promoter(1)
(c) 395. Rosetta 2000: Expression in response to 2-deoxy-D-glucose(1)
(c) 407. Rosetta 2000: Expression in response to Tunicamycin(1)
(c) 481. Expression in response to heat shock: 15,30,45,60,120 min(1)
(c) 481. Expression in response to heat shock: 15,30,45,60,120 min(2)
(c) 481. Expression in response to heat shock: 15,30,45,60,120 min(3)
(c) 481. Expression in response to heat shock: 15,30,45,60,120 min(4)
(c) 523. Brown enviromental changes :constant 0.32 mM H2O2 (10 min) redo(1)
(c) 534. Brown enviromental changes :1 mM Menadione (20 min) redo(1)
(c) 536. Brown enviromental changes :1mM Menadione (40 min) redo(1)
(c) 563. Brown enviromental changes :1.5 mM diamide (60 min)(1)
(c) 564. Brown enviromental changes :1.5 mM diamide (90 min)(1)

TEC1 -*-> FUS3

(c) 5. Expression during the cell cycle (alpha factor arrest and release)(1)
(c) 7. Expression during the cell Cycle (cdc28)(17)
(c) 26. Fink: Expression in diploid high copy TEC1(1)
(c) 48. Expression in response to 0.15,0.5,1.5,5,15.8,50,158,500 nM alpha-factor(4)
(c) 48. Expression in response to 0.15,0.5,1.5,5,15.8,50,158,500 nM alpha-factor(5)
(c) 48. Expression in response to 0.15,0.5,1.5,5,15.8,50,158,500 nM alpha-factor(8)
(c) 49. Expression in response to 50 nM alpha-factor: 0,15,30,45,60,90,120 min(1)
(c) 49. Expression in response to 50 nM alpha-factor: 0,15,30,45,60,90,120 min(3)
(c) 49. Expression in response to 50 nM alpha-factor: 0,15,30,45,60,90,120 min(4)
(c) 49. Expression in response to 50 nM alpha-factor: 0,15,30,45,60,90,120 min(6)
(c) 49. Expression in response to 50 nM alpha-factor: 0,15,30,45,60,90,120 min(7)
(c) 53. Expression in response to overproduction of Ste4p(1)
(c) 54. Expression in response to overproduction of Ste5p(1)
(c) 55. Expression in response to overproduction of Ste11p(1)
(c) 74. Expression in response to overproduction of activated Rho1p(1)
(c) 388. Rosetta 2000: Expression in cells with FKS1 under tet promoter(1)
(c) 395. Rosetta 2000: Expression in response to 2-deoxy-D-glucose(1)
(c) 407. Rosetta 2000: Expression in response to Tunicamycin(1)
(c) 481. Expression in response to heat shock: 15,30,45,60,120 min(1)
(c) 481. Expression in response to heat shock: 15,30,45,60,120 min(2)
(c) 481. Expression in response to heat shock: 15,30,45,60,120 min(3)
(c) 481. Expression in response to heat shock: 15,30,45,60,120 min(4)
(c) 523. Brown enviromental changes :constant 0.32 mM H2O2 (10 min) redo(1)
(c) 534. Brown enviromental changes :1 mM Menadione (20 min) redo(1)
(c) 536. Brown enviromental changes :1mM Menadione (40 min) redo(1)
(c) 563. Brown enviromental changes :1.5 mM diamide (60 min)(1)
(c) 564. Brown enviromental changes :1.5 mM diamide (90 min)(1)

TEC1 -*-> PRM1

(c) 5. Expression during the cell cycle (alpha factor arrest and release)(1)

(c) 7. Expression during the cell Cycle (cdc28)(17)
(c) 26. Fink: Expression in diploid high copy TEC1(1)
(c) 48. Expression in response to 0.15,0.5,1.5,5,15.8,50,158,500 nM alpha-factor(4)
(c) 48. Expression in response to 0.15,0.5,1.5,5,15.8,50,158,500 nM alpha-factor(5)
(c) 48. Expression in response to 0.15,0.5,1.5,5,15.8,50,158,500 nM alpha-factor(8)
(c) 49. Expression in response to 50 nM alpha-factor: 0,15,30,45,60,90,120 min(1)
(c) 49. Expression in response to 50 nM alpha-factor: 0,15,30,45,60,90,120 min(3)
(c) 49. Expression in response to 50 nM alpha-factor: 0,15,30,45,60,90,120 min(4)
(c) 49. Expression in response to 50 nM alpha-factor: 0,15,30,45,60,90,120 min(6)
(c) 49. Expression in response to 50 nM alpha-factor: 0,15,30,45,60,90,120 min(7)
(c) 53. Expression in response to overproduction of Ste4p(1)
(c) 54. Expression in response to overproduction of Ste5p(1)
(c) 55. Expression in response to overproduction of Ste11p(1)
(c) 74. Expression in response to overproduction of activated Rho1p(1)
(c) 388. Rosetta 2000: Expression in cells with FKS1 under tet promoter(1)
(c) 395. Rosetta 2000: Expression in response to 2-deoxy-D-glucose(1)
(c) 407. Rosetta 2000: Expression in response to Tunicamycin(1)
(c) 481. Expression in response to heat shock: 15,30,45,60,120 min(1)
(c) 481. Expression in response to heat shock: 15,30,45,60,120 min(2)
(c) 481. Expression in response to heat shock: 15,30,45,60,120 min(3)
(c) 481. Expression in response to heat shock: 15,30,45,60,120 min(4)
(c) 523. Brown enviromental changes :constant 0.32 mM H2O2 (10 min) redo(1)
(c) 534. Brown enviromental changes :1 mM Menadione (20 min) redo(1)
(c) 536. Brown enviromental changes :1mM Menadione (40 min) redo(1)
(c) 563. Brown enviromental changes :1.5 mM diamide (60 min)(1)
(c) 564. Brown enviromental changes :1.5 mM diamide (90 min)(1)

TEC1 -*-> PCL2

(c) 5. Expression during the cell cycle (alpha factor arrest and release)(1)
(c) 6. Expression during the cell cycle (cdc15 arrest and release)(20)
(c) 26. Fink: Expression in diploid high copy TEC1(1)
(c) 48. Expression in response to 0.15,0.5,1.5,5,15.8,50,158,500 nM alpha-factor(4)
(c) 48. Expression in response to 0.15,0.5,1.5,5,15.8,50,158,500 nM alpha-factor(5)
(c) 48. Expression in response to 0.15,0.5,1.5,5,15.8,50,158,500 nM alpha-factor(6)
(c) 48. Expression in response to 0.15,0.5,1.5,5,15.8,50,158,500 nM alpha-factor(7)
(c) 48. Expression in response to 0.15,0.5,1.5,5,15.8,50,158,500 nM alpha-factor(8)
(c) 49. Expression in response to 50 nM alpha-factor: 0,15,30,45,60,90,120 min(1)
(c) 49. Expression in response to 50 nM alpha-factor: 0,15,30,45,60,90,120 min(2)
(c) 49. Expression in response to 50 nM alpha-factor: 0,15,30,45,60,90,120 min(3)
(c) 49. Expression in response to 50 nM alpha-factor: 0,15,30,45,60,90,120 min(4)
(c) 49. Expression in response to 50 nM alpha-factor: 0,15,30,45,60,90,120 min(6)
(c) 49. Expression in response to 50 nM alpha-factor: 0,15,30,45,60,90,120 min(7)
(c) 53. Expression in response to overproduction of Ste4p(1)
(c) 56. Expression in response to overproduction of Ste12p(1)
(c) 407. Rosetta 2000: Expression in response to Tunicamycin(1)
(c) 481. Expression in response to heat shock: 15,30,45,60,120 min(1)
(c) 523. Brown enviromental changes :constant 0.32 mM H2O2 (10 min) redo(1)
(c) 556. Brown enviromental changes :dtt 480 min dtt-2(1)
(c) 572. Brown enviromental changes :Hypo-osmotic shock - 5 min(1)
(c) (Rich Media 2% Glucose YPD-185588) wt 5mM aF, 30 min.
(c) Rich Media 2% Glucose YPD-Average wt 5mM aF, 30 min.
(c) 100 microM BCS 30 min
(c) 100 microM BCS 60 min

TEC1 -*-> PRM6

(c) 5. Expression during the cell cycle (alpha factor arrest and release)(1)
(c) 6. Expression during the cell cycle (cdc15 arrest and release)(20)
(c) 26. Fink: Expression in diploid high copy TEC1(1)
(c) 48. Expression in response to 0.15,0.5,1.5,5,15.8,50,158,500 nM alpha-factor(4)
(c) 48. Expression in response to 0.15,0.5,1.5,5,15.8,50,158,500 nM alpha-factor(5)
(c) 48. Expression in response to 0.15,0.5,1.5,5,15.8,50,158,500 nM alpha-factor(6)
(c) 48. Expression in response to 0.15,0.5,1.5,5,15.8,50,158,500 nM alpha-factor(7)
(c) 48. Expression in response to 0.15,0.5,1.5,5,15.8,50,158,500 nM alpha-factor(8)
(c) 49. Expression in response to 50 nM alpha-factor: 0,15,30,45,60,90,120 min(1)
(c) 49. Expression in response to 50 nM alpha-factor: 0,15,30,45,60,90,120 min(2)
(c) 49. Expression in response to 50 nM alpha-factor: 0,15,30,45,60,90,120 min(3)
(c) 49. Expression in response to 50 nM alpha-factor: 0,15,30,45,60,90,120 min(4)
(c) 49. Expression in response to 50 nM alpha-factor: 0,15,30,45,60,90,120 min(6)
(c) 49. Expression in response to 50 nM alpha-factor: 0,15,30,45,60,90,120 min(7)
(c) 53. Expression in response to overproduction of Ste4p(1)
(c) 56. Expression in response to overproduction of Ste12p(1)
(c) 407. Rosetta 2000: Expression in response to Tunicamycin(1)
(c) 481. Expression in response to heat shock: 15,30,45,60,120 min(1)
(c) 523. Brown enviromental changes :constant 0.32 mM H2O2 (10 min) redo(1)
(c) 556. Brown enviromental changes :dtt 480 min dtt-2(1)
(c) 572. Brown enviromental changes :Hypo-osmotic shock - 5 min(1)
(c) (Rich Media 2% Glucose YPD-185588) wt 5mM aF, 30 min.
(c) Rich Media 2% Glucose YPD-Average wt 5mM aF, 30 min.
(c) 100 microM BCS 30 min
(c) 100 microM BCS 60 min

TEC1 -*-> STE12

(c) 5. Expression during the cell cycle (alpha factor arrest and release)(1)
(c) 6. Expression during the cell cycle (cdc15 arrest and release)(20)
(c) 26. Fink: Expression in diploid high copy TEC1(1)
(c) 48. Expression in response to 0.15,0.5,1.5,5,15.8,50,158,500 nM alpha-factor(4)
(c) 48. Expression in response to 0.15,0.5,1.5,5,15.8,50,158,500 nM alpha-factor(5)
(c) 48. Expression in response to 0.15,0.5,1.5,5,15.8,50,158,500 nM alpha-factor(6)
(c) 48. Expression in response to 0.15,0.5,1.5,5,15.8,50,158,500 nM alpha-factor(7)
(c) 48. Expression in response to 0.15,0.5,1.5,5,15.8,50,158,500 nM alpha-factor(8)
(c) 49. Expression in response to 50 nM alpha-factor: 0,15,30,45,60,90,120 min(1)
(c) 49. Expression in response to 50 nM alpha-factor: 0,15,30,45,60,90,120 min(2)
(c) 49. Expression in response to 50 nM alpha-factor: 0,15,30,45,60,90,120 min(3)
(c) 49. Expression in response to 50 nM alpha-factor: 0,15,30,45,60,90,120 min(4)
(c) 49. Expression in response to 50 nM alpha-factor: 0,15,30,45,60,90,120 min(6)
(c) 49. Expression in response to 50 nM alpha-factor: 0,15,30,45,60,90,120 min(7)
(c) 53. Expression in response to overproduction of Ste4p(1)
(c) 56. Expression in response to overproduction of Ste12p(1)
(c) 407. Rosetta 2000: Expression in response to Tunicamycin(1)
(c) 481. Expression in response to heat shock: 15,30,45,60,120 min(1)
(c) 523. Brown enviromental changes :constant 0.32 mM H2O2 (10 min) redo(1)
(c) 556. Brown enviromental changes :dtt 480 min dtt-2(1)
(c) 572. Brown enviromental changes :Hypo-osmotic shock - 5 min(1)

(c) (Rich Media 2% Glucose YPD-185588) wt 5mM aF, 30 min.
(c) Rich Media 2% Glucose YPD-Average wt 5mM aF, 30 min.
(c) 100 microM BCS 30 min
(c) 100 microM BCS 60 min

TEC1 -*-> GFA1

(c) 5. Expression during the cell cycle (alpha factor arrest and release)(1)
(c) 6. Expression during the cell cycle (cdc15 arrest and release)(22)
(c) 6. Expression during the cell cycle (cdc15 arrest and release)(23)
(c) 6. Expression during the cell cycle (cdc15 arrest and release)(24)
(c) 7. Expression during the cell Cycle (cdc28)(17)
(c) 26. Fink: Expression in diploid high copy TEC1(1)
(c) 48. Expression in response to 0.15,0.5,1.5,5,15.8,50,158,500 nM alpha-factor(4)
(c) 48. Expression in response to 0.15,0.5,1.5,5,15.8,50,158,500 nM alpha-factor(5)
(c) 48. Expression in response to 0.15,0.5,1.5,5,15.8,50,158,500 nM alpha-factor(6)
(c) 49. Expression in response to 50 nM alpha-factor: 0,15,30,45,60,90,120 min(1)
(c) 49. Expression in response to 50 nM alpha-factor: 0,15,30,45,60,90,120 min(2)
(c) 49. Expression in response to 50 nM alpha-factor: 0,15,30,45,60,90,120 min(3)
(c) 49. Expression in response to 50 nM alpha-factor: 0,15,30,45,60,90,120 min(7)
(c) 53. Expression in response to overproduction of Ste4p(1)
(c) 54. Expression in response to overproduction of Ste5p(1)
(c) 332. Rosetta 2000: Expression in cells with CMD1 under tet promoter(1)
(c) 395. Rosetta 2000: Expression in response to 2-deoxy-D-glucose(1)
(c) 400. Rosetta 2000: Expression in response to Glucosamine(1)
(c) 407. Rosetta 2000: Expression in response to Tunicamycin(1)
(c) 493. Brown enviromental changes :Heat Shock 40 minutes hs-1(1)
(c) 523. Brown enviromental changes :constant 0.32 mM H2O2 (10 min) redo(1)
(c) 561. Brown enviromental changes :1.5 mM diamide (40 min)(1)
(c) 562. Brown enviromental changes :1.5 mM diamide (50 min)(1)
(c) 564. Brown enviromental changes :1.5 mM diamide (90 min)(1)
(c) 572. Brown enviromental changes :Hypo-osmotic shock - 5 min(1)
(c) (Rich Media 2% Glucose YPD-185588) wt 5mM aF, 30 min.
(c) (Rich Media 2% Glucose YPD-185769) wt 5mM aF, 30 min.
(c) Rich Media 2% Glucose YPD-Average wt 5mM aF, 30 min.
(c) wt+gal

TEC1 -*-> ASH1

(c) 5. Expression during the cell cycle (alpha factor arrest and release)(10)
(c) 5. Expression during the cell cycle (alpha factor arrest and release)(11)
(c) 5. Expression during the cell cycle (alpha factor arrest and release)(12)
(c) 5. Expression during the cell cycle (alpha factor arrest and release)(13)
(c) 6. Expression during the cell cycle (cdc15 arrest and release)(2)
(c) 6. Expression during the cell cycle (cdc15 arrest and release)(11)
(c) 6. Expression during the cell cycle (cdc15 arrest and release)(20)
(c) 7. Expression during the cell Cycle (cdc28)(10)
(c) 7. Expression during the cell Cycle (cdc28)(11)
(c) 7. Expression during the cell Cycle (cdc28)(17)
(c) 8. Expression during the cell cycle (cell size selection and release)(12)
(c) 8. Expression during the cell cycle (cell size selection and release)(13)
(c) 8. Expression during the cell cycle (cell size selection and release)(14)
(c) 11. Expression during diauxic shift: 9h,11h,13h,15h,17h,19h,21h(3)
(c) 26. Fink: Expression in diploid high copy TEC1(1)
(c) 49. Expression in response to 50 nM alpha-factor: 0,15,30,45,60,90,120 min(1)
(c) 407. Rosetta 2000: Expression in response to Tunicamycin(1)
(c) 481. Expression in response to heat shock: 15,30,45,60,120 min(1)
(c) 481. Expression in response to heat shock: 15,30,45,60,120 min(2)
(c) 493. Brown enviromental changes :Heat Shock 40 minutes hs-1(1)
(c) 523. Brown enviromental changes :constant 0.32 mM H2O2 (10 min) redo(1)
(c) 595. Brown enviromental changes :diauxic shift timecourse(1)

TEC1 -*-> CST13

(c) 5. Expression during the cell cycle (alpha factor arrest and release)(10)
(c) 5. Expression during the cell cycle (alpha factor arrest and release)(11)
(c) 5. Expression during the cell cycle (alpha factor arrest and release)(12)
(c) 5. Expression during the cell cycle (alpha factor arrest and release)(13)
(c) 6. Expression during the cell cycle (cdc15 arrest and release)(2)
(c) 6. Expression during the cell cycle (cdc15 arrest and release)(11)
(c) 6. Expression during the cell cycle (cdc15 arrest and release)(20)
(c) 7. Expression during the cell Cycle (cdc28)(10)
(c) 7. Expression during the cell Cycle (cdc28)(11)
(c) 7. Expression during the cell Cycle (cdc28)(17)
(c) 8. Expression during the cell cycle (cell size selection and release)(12)
(c) 8. Expression during the cell cycle (cell size selection and release)(13)
(c) 8. Expression during the cell cycle (cell size selection and release)(14)
(c) 11. Expression during diauxic shift: 9h,11h,13h,15h,17h,19h,21h(3)
(c) 26. Fink: Expression in diploid high copy TEC1(1)
(c) 49. Expression in response to 50 nM alpha-factor: 0,15,30,45,60,90,120 min(1)
(c) 407. Rosetta 2000: Expression in response to Tunicamycin(1)
(c) 481. Expression in response to heat shock: 15,30,45,60,120 min(1)
(c) 481. Expression in response to heat shock: 15,30,45,60,120 min(2)
(c) 493. Brown enviromental changes :Heat Shock 40 minutes hs-1(1)
(c) 523. Brown enviromental changes :constant 0.32 mM H2O2 (10 min) redo(1)
(c) 595. Brown enviromental changes :diauxic shift timecourse(1)

TEC1 -*-> FAA3

(c) 5. Expression during the cell cycle (alpha factor arrest and release)(10)
(c) 5. Expression during the cell cycle (alpha factor arrest and release)(11)
(c) 5. Expression during the cell cycle (alpha factor arrest and release)(12)
(c) 5. Expression during the cell cycle (alpha factor arrest and release)(13)
(c) 6. Expression during the cell cycle (cdc15 arrest and release)(2)
(c) 6. Expression during the cell cycle (cdc15 arrest and release)(11)
(c) 6. Expression during the cell cycle (cdc15 arrest and release)(20)
(c) 7. Expression during the cell Cycle (cdc28)(10)
(c) 7. Expression during the cell Cycle (cdc28)(11)
(c) 7. Expression during the cell Cycle (cdc28)(17)
(c) 8. Expression during the cell cycle (cell size selection and release)(12)
(c) 8. Expression during the cell cycle (cell size selection and release)(13)
(c) 8. Expression during the cell cycle (cell size selection and release)(14)
(c) 11. Expression during diauxic shift: 9h,11h,13h,15h,17h,19h,21h(3)
(c) 26. Fink: Expression in diploid high copy TEC1(1)

(c) 49. Expression in response to 50 nM alpha-factor: 0,15,30,45,60,90,120 min(1)
(c) 407. Rosetta 2000: Expression in response to Tunicamycin(1)
(c) 481. Expression in response to heat shock: 15,30,45,60,120 min(1)
(c) 481. Expression in response to heat shock: 15,30,45,60,120 min(2)
(c) 493. Brown enviromental changes :Heat Shock 40 minutes hs-1(1)
(c) 523. Brown enviromental changes :constant 0.32 mM H2O2 (10 min) redo(1)
(c) 595. Brown enviromental changes :diauxic shift timecourse(1)

TEC1 -*-> HSP150

(c) 5. Expression during the cell cycle (alpha factor arrest and release)(10)
(c) 5. Expression during the cell cycle (alpha factor arrest and release)(11)
(c) 5. Expression during the cell cycle (alpha factor arrest and release)(12)
(c) 5. Expression during the cell cycle (alpha factor arrest and release)(13)
(c) 6. Expression during the cell cycle (cdc15 arrest and release)(2)
(c) 6. Expression during the cell cycle (cdc15 arrest and release)(11)
(c) 6. Expression during the cell cycle (cdc15 arrest and release)(20)
(c) 7. Expression during the cell Cycle (cdc28)(10)
(c) 7. Expression during the cell Cycle (cdc28)(11)
(c) 7. Expression during the cell Cycle (cdc28)(17)
(c) 8. Expression during the cell cycle (cell size selection and release)(12)
(c) 8. Expression during the cell cycle (cell size selection and release)(13)
(c) 8. Expression during the cell cycle (cell size selection and release)(14)
(c) 11. Expression during diauxic shift: 9h,11h,13h,15h,17h,19h,21h(3)
(c) 26. Fink: Expression in diploid high copy TEC1(1)
(c) 49. Expression in response to 50 nM alpha-factor: 0,15,30,45,60,90,120 min(1)
(c) 407. Rosetta 2000: Expression in response to Tunicamycin(1)
(c) 481. Expression in response to heat shock: 15,30,45,60,120 min(1)
(c) 481. Expression in response to heat shock: 15,30,45,60,120 min(2)
(c) 493. Brown enviromental changes :Heat Shock 40 minutes hs-1(1)
(c) 523. Brown enviromental changes :constant 0.32 mM H2O2 (10 min) redo(1)
(c) 595. Brown enviromental changes :diauxic shift timecourse(1)

TEC1 -*-> PIR1

(c) 5. Expression during the cell cycle (alpha factor arrest and release)(10)
(c) 5. Expression during the cell cycle (alpha factor arrest and release)(11)
(c) 5. Expression during the cell cycle (alpha factor arrest and release)(12)
(c) 5. Expression during the cell cycle (alpha factor arrest and release)(13)
(c) 6. Expression during the cell cycle (cdc15 arrest and release)(2)
(c) 6. Expression during the cell cycle (cdc15 arrest and release)(11)
(c) 6. Expression during the cell cycle (cdc15 arrest and release)(20)
(c) 7. Expression during the cell Cycle (cdc28)(10)
(c) 7. Expression during the cell Cycle (cdc28)(11)
(c) 7. Expression during the cell Cycle (cdc28)(17)
(c) 8. Expression during the cell cycle (cell size selection and release)(12)
(c) 8. Expression during the cell cycle (cell size selection and release)(13)
(c) 8. Expression during the cell cycle (cell size selection and release)(14)
(c) 11. Expression during diauxic shift: 9h,11h,13h,15h,17h,19h,21h(3)
(c) 26. Fink: Expression in diploid high copy TEC1(1)
(c) 49. Expression in response to 50 nM alpha-factor: 0,15,30,45,60,90,120 min(1)
(c) 407. Rosetta 2000: Expression in response to Tunicamycin(1)
(c) 481. Expression in response to heat shock: 15,30,45,60,120 min(1)
(c) 481. Expression in response to heat shock: 15,30,45,60,120 min(2)
(c) 493. Brown enviromental changes :Heat Shock 40 minutes hs-1(1)
(c) 523. Brown enviromental changes :constant 0.32 mM H2O2 (10 min) redo(1)
(c) 595. Brown enviromental changes :diauxic shift timecourse(1)

TEC1 -*-> PIR3

(c) 5. Expression during the cell cycle (alpha factor arrest and release)(10)
(c) 5. Expression during the cell cycle (alpha factor arrest and release)(11)
(c) 5. Expression during the cell cycle (alpha factor arrest and release)(12)
(c) 5. Expression during the cell cycle (alpha factor arrest and release)(13)
(c) 6. Expression during the cell cycle (cdc15 arrest and release)(2)
(c) 6. Expression during the cell cycle (cdc15 arrest and release)(11)
(c) 6. Expression during the cell cycle (cdc15 arrest and release)(20)
(c) 7. Expression during the cell Cycle (cdc28)(10)
(c) 7. Expression during the cell Cycle (cdc28)(11)
(c) 7. Expression during the cell Cycle (cdc28)(17)
(c) 8. Expression during the cell cycle (cell size selection and release)(12)
(c) 8. Expression during the cell cycle (cell size selection and release)(13)
(c) 8. Expression during the cell cycle (cell size selection and release)(14)
(c) 11. Expression during diauxic shift: 9h,11h,13h,15h,17h,19h,21h(3)
(c) 26. Fink: Expression in diploid high copy TEC1(1)
(c) 49. Expression in response to 50 nM alpha-factor: 0,15,30,45,60,90,120 min(1)
(c) 407. Rosetta 2000: Expression in response to Tunicamycin(1)
(c) 481. Expression in response to heat shock: 15,30,45,60,120 min(1)
(c) 481. Expression in response to heat shock: 15,30,45,60,120 min(2)
(c) 493. Brown enviromental changes :Heat Shock 40 minutes hs-1(1)
(c) 523. Brown enviromental changes :constant 0.32 mM H2O2 (10 min) redo(1)
(c) 595. Brown enviromental changes :diauxic shift timecourse(1)

TEC1 -*-> AGA2

(c) 3. Cell Cycle: Expression in response to Clb2p (set 1, 40 min)(1)
(c) 4. Cell Cycle: Expression in response to Clb2p (set 2, 30 min)(1)
(c) 6. Expression during the cell cycle (cdc15 arrest and release)(11)
(c) 6. Expression during the cell cycle (cdc15 arrest and release)(20)
(c) 7. Expression during the cell Cycle (cdc28)(10)
(c) 7. Expression during the cell Cycle (cdc28)(17)
(c) 8. Expression during the cell cycle (cell size selection and release)(12)
(c) 26. Fink: Expression in diploid high copy TEC1(1)
(c) 48. Expression in response to 0.15,0.5,1.5,5,15.8,50,158,500 nM alpha-factor(3)
(c) 48. Expression in response to 0.15,0.5,1.5,5,15.8,50,158,500 nM alpha-factor(4)
(c) 48. Expression in response to 0.15,0.5,1.5,5,15.8,50,158,500 nM alpha-factor(5)
(c) 48. Expression in response to 0.15,0.5,1.5,5,15.8,50,158,500 nM alpha-factor(6)
(c) 49. Expression in response to 50 nM alpha-factor: 0,15,30,45,60,90,120 min(1)
(c) 49. Expression in response to 50 nM alpha-factor: 0,15,30,45,60,90,120 min(2)
(c) 49. Expression in response to 50 nM alpha-factor: 0,15,30,45,60,90,120 min(3)
(c) 49. Expression in response to 50 nM alpha-factor: 0,15,30,45,60,90,120 min(4)
(c) 49. Expression in response to 50 nM alpha-factor: 0,15,30,45,60,90,120 min(7)
(c) 332. Rosetta 2000: Expression in cells with CMD1 under tet promoter(1)
(c) 495. Brown enviromental changes :Heat Shock 80 minutes hs-1(1)

(c) 523. Brown enviromental changes :constant 0.32 mM H2O2 (10 min) redo(1)
(c) (Rich Media 2% Glucose YPD-185588) wt 5mM aF, 30 min.
(c) (Rich Media 2% Glucose YPD-185769) wt 5mM aF, 30 min.
(c) Rich Media 2% Glucose YPD-Average wt 5mM aF, 30 min.
(c) 100 microM BCS 60 min

TEC1 -*-> DBF2

(c) 3. Cell Cycle: Expression in response to Clb2p (set 1, 40 min)(1)
(c) 4. Cell Cycle: Expression in response to Clb2p (set 2, 30 min)(1)
(c) 6. Expression during the cell cycle (cdc15 arrest and release)(11)
(c) 6. Expression during the cell cycle (cdc15 arrest and release)(20)
(c) 7. Expression during the cell Cycle (cdc28)(10)
(c) 7. Expression during the cell Cycle (cdc28)(17)
(c) 8. Expression during the cell cycle (cell size selection and release)(12)
(c) 26. Fink: Expression in diploid high copy TEC1(1)
(c) 48. Expression in response to 0.15,0.5,1.5,5,15.8,50,158,500 nM alpha-factor(3)
(c) 48. Expression in response to 0.15,0.5,1.5,5,15.8,50,158,500 nM alpha-factor(4)
(c) 48. Expression in response to 0.15,0.5,1.5,5,15.8,50,158,500 nM alpha-factor(5)
(c) 48. Expression in response to 0.15,0.5,1.5,5,15.8,50,158,500 nM alpha-factor(6)
(c) 49. Expression in response to 50 nM alpha-factor: 0,15,30,45,60,90,120 min(1)
(c) 49. Expression in response to 50 nM alpha-factor: 0,15,30,45,60,90,120 min(2)
(c) 49. Expression in response to 50 nM alpha-factor: 0,15,30,45,60,90,120 min(3)
(c) 49. Expression in response to 50 nM alpha-factor: 0,15,30,45,60,90,120 min(4)
(c) 49. Expression in response to 50 nM alpha-factor: 0,15,30,45,60,90,120 min(7)
(c) 332. Rosetta 2000: Expression in cells with CMD1 under tet promoter(1)
(c) 495. Brown enviromental changes :Heat Shock 80 minutes hs-1(1)
(c) 523. Brown enviromental changes :constant 0.32 mM H2O2 (10 min) redo(1)
(c) (Rich Media 2% Glucose YPD-185588) wt 5mM aF, 30 min.
(c) (Rich Media 2% Glucose YPD-185769) wt 5mM aF, 30 min.
(c) Rich Media 2% Glucose YPD-Average wt 5mM aF, 30 min.
(c) 100 microM BCS 60 min

TEC1 -*-> KAR5

(c) 3. Cell Cycle: Expression in response to Clb2p (set 1, 40 min)(1)
(c) 4. Cell Cycle: Expression in response to Clb2p (set 2, 30 min)(1)
(c) 6. Expression during the cell cycle (cdc15 arrest and release)(11)
(c) 6. Expression during the cell cycle (cdc15 arrest and release)(20)
(c) 7. Expression during the cell Cycle (cdc28)(10)
(c) 7. Expression during the cell Cycle (cdc28)(17)
(c) 8. Expression during the cell cycle (cell size selection and release)(12)
(c) 26. Fink: Expression in diploid high copy TEC1(1)
(c) 48. Expression in response to 0.15,0.5,1.5,5,15.8,50,158,500 nM alpha-factor(3)
(c) 48. Expression in response to 0.15,0.5,1.5,5,15.8,50,158,500 nM alpha-factor(4)
(c) 48. Expression in response to 0.15,0.5,1.5,5,15.8,50,158,500 nM alpha-factor(5)
(c) 48. Expression in response to 0.15,0.5,1.5,5,15.8,50,158,500 nM alpha-factor(6)
(c) 49. Expression in response to 50 nM alpha-factor: 0,15,30,45,60,90,120 min(1)
(c) 49. Expression in response to 50 nM alpha-factor: 0,15,30,45,60,90,120 min(2)
(c) 49. Expression in response to 50 nM alpha-factor: 0,15,30,45,60,90,120 min(3)
(c) 49. Expression in response to 50 nM alpha-factor: 0,15,30,45,60,90,120 min(4)
(c) 49. Expression in response to 50 nM alpha-factor: 0,15,30,45,60,90,120 min(7)
(c) 332. Rosetta 2000: Expression in cells with CMD1 under tet promoter(1)
(c) 495. Brown enviromental changes :Heat Shock 80 minutes hs-1(1)
(c) 523. Brown enviromental changes :constant 0.32 mM H2O2 (10 min) redo(1)
(c) (Rich Media 2% Glucose YPD-185588) wt 5mM aF, 30 min.
(c) (Rich Media 2% Glucose YPD-185769) wt 5mM aF, 30 min.
(c) Rich Media 2% Glucose YPD-Average wt 5mM aF, 30 min.
(c) 100 microM BCS 60 min

TEC1 -*-> PRP39

(c) 3. Cell Cycle: Expression in response to Clb2p (set 1, 40 min)(1)
(c) 4. Cell Cycle: Expression in response to Clb2p (set 2, 30 min)(1)
(c) 6. Expression during the cell cycle (cdc15 arrest and release)(11)
(c) 6. Expression during the cell cycle (cdc15 arrest and release)(20)
(c) 7. Expression during the cell Cycle (cdc28)(10)
(c) 7. Expression during the cell Cycle (cdc28)(17)
(c) 8. Expression during the cell cycle (cell size selection and release)(12)
(c) 26. Fink: Expression in diploid high copy TEC1(1)
(c) 48. Expression in response to 0.15,0.5,1.5,5,15.8,50,158,500 nM alpha-factor(3)
(c) 48. Expression in response to 0.15,0.5,1.5,5,15.8,50,158,500 nM alpha-factor(4)
(c) 48. Expression in response to 0.15,0.5,1.5,5,15.8,50,158,500 nM alpha-factor(5)
(c) 48. Expression in response to 0.15,0.5,1.5,5,15.8,50,158,500 nM alpha-factor(6)
(c) 49. Expression in response to 50 nM alpha-factor: 0,15,30,45,60,90,120 min(1)
(c) 49. Expression in response to 50 nM alpha-factor: 0,15,30,45,60,90,120 min(2)
(c) 49. Expression in response to 50 nM alpha-factor: 0,15,30,45,60,90,120 min(3)
(c) 49. Expression in response to 50 nM alpha-factor: 0,15,30,45,60,90,120 min(4)
(c) 49. Expression in response to 50 nM alpha-factor: 0,15,30,45,60,90,120 min(7)
(c) 332. Rosetta 2000: Expression in cells with CMD1 under tet promoter(1)
(c) 495. Brown enviromental changes :Heat Shock 80 minutes hs-1(1)
(c) 523. Brown enviromental changes :constant 0.32 mM H2O2 (10 min) redo(1)
(c) (Rich Media 2% Glucose YPD-185588) wt 5mM aF, 30 min.
(c) (Rich Media 2% Glucose YPD-185769) wt 5mM aF, 30 min.
(c) Rich Media 2% Glucose YPD-Average wt 5mM aF, 30 min.
(c) 100 microM BCS 60 min

TEC1 -*-> SCW10

(c) 5. Expression during the cell cycle (alpha factor arrest and release)(1)
(c) 5. Expression during the cell cycle (alpha factor arrest and release)(13)
(c) 5. Expression during the cell cycle (alpha factor arrest and release)(15)
(c) 7. Expression during the cell Cycle (cdc28)(11)
(c) 8. Expression during the cell cycle (cell size selection and release)(8)
(c) 8. Expression during the cell cycle (cell size selection and release)(9)
(c) 8. Expression during the cell cycle (cell size selection and release)(10)
(c) 8. Expression during the cell cycle (cell size selection and release)(12)
(c) 11. Expression during diauxic shift: 9h,11h,13h,15h,17h,19h,21h(3)
(c) 26. Fink: Expression in diploid high copy TEC1(1)
(c) 48. Expression in response to 0.15,0.5,1.5,5,15.8,50,158,500 nM alpha-factor(5)
(c) 48. Expression in response to 0.15,0.5,1.5,5,15.8,50,158,500 nM alpha-factor(6)
(c) 48. Expression in response to 0.15,0.5,1.5,5,15.8,50,158,500 nM alpha-factor(8)
(c) 49. Expression in response to 50 nM alpha-factor: 0,15,30,45,60,90,120 min(1)
(c) 49. Expression in response to 50 nM alpha-factor: 0,15,30,45,60,90,120 min(2)

```
    (c) 49. Expression in response to 50 nM alpha-factor: 0,15,30,45,60,90,120 min(3)
    (c) 49. Expression in response to 50 nM alpha-factor: 0,15,30,45,60,90,120 min(7)
    (c) 53. Expression in response to overproduction of Ste4p(1)
    (c) 407. Rosetta 2000: Expression in response to Tunicamycin(1)
    (c) 523. Brown enviromental changes :constant 0.32 mM H2O2 (10 min) redo(1)
    (c) 595. Brown enviromental changes :diauxic shift timecourse(1)
    (c) (Rich Media 2% Glucose YPD-185769) wt  5mM aF, 30 min.
    (c) Rich Media 2% Glucose YPD-Average wt  5mM aF, 30 min.
    (c) wt_plus_gamma_10_min
    (c) DES460 (wt) - mock irradiation - 30 min
    (c) 100 microM BCS 60 min


TEC1 -*-> YHR097C


    (c) 5. Expression during the cell cycle (alpha factor arrest and release)(1)
    (c) 7. Expression during the cell Cycle (cdc28)(10)
    (c) 11. Expression during diauxic shift: 9h,11h,13h,15h,17h,19h,21h(3)
    (c) 26. Fink: Expression in diploid high copy TEC1(1)
    (c) 49. Expression in response to 50 nM alpha-factor: 0,15,30,45,60,90,120 min(1)
    (c) 49. Expression in response to 50 nM alpha-factor: 0,15,30,45,60,90,120 min(3)
    (c) 49. Expression in response to 50 nM alpha-factor: 0,15,30,45,60,90,120 min(6)
    (c) 49. Expression in response to 50 nM alpha-factor: 0,15,30,45,60,90,120 min(7)
    (c) 53. Expression in response to overproduction of Ste4p(1)
    (c) 407. Rosetta 2000: Expression in response to Tunicamycin(1)
    (c) 513. Brown enviromental changes :29C to 33C - 5 minutes(1)
    (c) 523. Brown enviromental changes :constant 0.32 mM H2O2 (10 min) redo(1)
    (c) 564. Brown enviromental changes :1.5 mM diamide (90 min)(1)
    (c) 572. Brown enviromental changes :Hypo-osmotic shock - 5 min(1)
    (c) 595. Brown enviromental changes :diauxic shift timecourse(1)
    (c) 681. Expression in response to 0.4M NaCl for 10 min in wild type(1)
    (c) 685. Expression in response to 0.8M NaCl for 20 min in wild type(1)
    (c) (Rich Media 2% Glucose YPD-185588) wt  5mM aF, 30 min.
    (c) 100 microM BCS 30 min


UME6 -*-> YOR291W


    (c) 5. Expression during the cell cycle (alpha factor arrest and release)(3)
    (c) 5. Expression during the cell cycle (alpha factor arrest and release)(11)
    (c) 5. Expression during the cell cycle (alpha factor arrest and release)(15)
    (c) 8. Expression during the cell cycle (cell size selection and release)(3)
    (c) 11. Expression during diauxic shift: 9h,11h,13h,15h,17h,19h,21h(3)
    (c) 385. Rosetta 2000: Expression in cells with AUR1 under tet promoter(1)
    (c) 452. Expression in response to low 4NQO (2 microgram/ml) for 60 min(1)
    (c) 453. Expression in response to gama-ray exposure (30 kilorad) for 60 min(1)
    (c) 454. Expression in response to tBuOOH (5mM) for 60 min(1)
    (c) 455. Expression in response to high MNNG (27 microgram/ml) for 60 min(1)
    (c) 456. Expression in response to high 4NQO (8 microgram/ml) for 60 min(1)
    (c) 482. Expression in response to acid: 10,20,40,60,80,100 min(4)
    (c) 487. Expression in response to sorbitol: 15 30 45 90 120 min(3)
    (c) 487. Expression in response to sorbitol: 15 30 45 90 120 min(5)
    (c) 515. Brown enviromental changes :29C to 33C - 30 minutes(1)
    (c) 595. Brown enviromental changes :diauxic shift timecourse(1)
    (c) 611. Brown enviromental changes :YPD stationary phase 2 h ypd-1(1)
    (c) (Rich Media 2% Glucose YPD-185588) wt  5mM aF, 30 min.
    (c) Rich Media 2% Glucose YPD-Average wt  5mM aF, 30 min.
    (c) (Var.) Rich Media 2% Glucose YPD-Average wt  5mM aF, 30 min.
    (c) 100 microM BCS 30 min


YAP1 -*-> AAD6


    (c) 6. Expression during the cell cycle (cdc15 arrest and release)(22)
    (c) 6. Expression during the cell cycle (cdc15 arrest and release)(23)
    (c) 6. Expression during the cell cycle (cdc15 arrest and release)(24)
    (c) 8. Expression during the cell cycle (cell size selection and release)(14)
    (c) 13. Expression in cells overexpressing Yap1p(1)
    (c) 445. Expression in response to 0.1% MMS for 60 min (average of 3 experiments)(1)
    (c) 447. Expression in response to 0.1% MMS for 30 min(1)
    (c) 448. Expression in response to 0.1% MMS for 60 min(1)
    (c) 449. Expression in response to 0.1% MMS for 60 min(1)
    (c) 451. Expression in response to BCNU (200 micromolar) for 60 min(1)
    (c) 464. Expression in response to 0.2% MMS for 60 min(1)
    (c) 518. Brown enviromental changes :29C +1M sorbitol to 33C + 1M sorbitol - 15 minutes(1)
    (c) 523. Brown enviromental changes :constant 0.32 mM H2O2 (10 min) redo(1)
    (c) 524. Brown enviromental changes :constant 0.32 mM H2O2 (20 min) redo(1)
    (c) 525. Brown enviromental changes :constant 0.32 mM H2O2 (30 min) redo(1)
    (c) 526. Brown enviromental changes :constant 0.32 mM H2O2 (40 min) rescan(1)
    (c) 527. Brown enviromental changes :constant 0.32 mM H2O2 (50 min) redo(1)
    (c) 528. Brown enviromental changes :constant 0.32 mM H2O2 (60 min) redo(1)
    (c) 529. Brown enviromental changes :constant 0.32 mM H2O2 (80 min) redo(1)
    (c) 530. Brown enviromental changes :constant 0.32 mM H2O2 (100 min) redo(1)
    (c) 531. Brown enviromental changes :constant 0.32 mM H2O2 (120 min) redo(1)
    (c) 532. Brown enviromental changes :constant 0.32 mM H2O2 (160 min) redo(1)
    (c) 534. Brown enviromental changes :1 mM Menadione (20 min) redo(1)
    (c) 535. Brown enviromental changes :1 mM Menadione (30 min) redo(1)
    (c) 536. Brown enviromental changes :1mM Menadione (40 min) redo(1)
    (c) 537. Brown enviromental changes :1 mM Menadione (50 min)redo(1)
    (c) 539. Brown enviromental changes :1 mM Menadione (105 min) redo(1)
    (c) 541. Brown enviromental changes :1 mM Menadione (160 min) redo(1)
    (c) 628. Brown enviromental changes :DBY7286 + 0.3 mM H2O2 (20 min)(1)
    (c) 635. Brown enviromental changes :YAP1 overexpression(1)
    (c) DES460 + 0.02% MMS - 15 min
    (c) DES460 + 0.02% MMS - 120 min


YAP1 -*-> ECM4


    (c) 6. Expression during the cell cycle (cdc15 arrest and release)(22)
    (c) 6. Expression during the cell cycle (cdc15 arrest and release)(23)
    (c) 6. Expression during the cell cycle (cdc15 arrest and release)(24)
    (c) 8. Expression during the cell cycle (cell size selection and release)(14)
    (c) 13. Expression in cells overexpressing Yap1p(1)
    (c) 445. Expression in response to 0.1% MMS for 60 min (average of 3 experiments)(1)
    (c) 447. Expression in response to 0.1% MMS for 30 min(1)
    (c) 448. Expression in response to 0.1% MMS for 60 min(1)
    (c) 449. Expression in response to 0.1% MMS for 60 min(1)
```

(c) 451. Expression in response to BCNU (200 micromolar) for 60 min(1)
(c) 464. Expression in response to 0.2% MMS for 60 min(1)
(c) 518. Brown enviromental changes :29C +1M sorbitol to 33C + 1M sorbitol - 15 minutes(1)
(c) 523. Brown enviromental changes :constant 0.32 mM H2O2 (10 min) redo(1)
(c) 524. Brown enviromental changes :constant 0.32 mM H2O2 (20 min) redo(1)
(c) 525. Brown enviromental changes :constant 0.32 mM H2O2 (30 min) redo(1)
(c) 526. Brown enviromental changes :constant 0.32 mM H2O2 (40 min) rescan(1)
(c) 527. Brown enviromental changes :constant 0.32 mM H2O2 (50 min) redo(1)
(c) 528. Brown enviromental changes :constant 0.32 mM H2O2 (60 min) redo(1)
(c) 529. Brown enviromental changes :constant 0.32 mM H2O2 (80 min) redo(1)
(c) 530. Brown enviromental changes :constant 0.32 mM H2O2 (100 min) redo(1)
(c) 531. Brown enviromental changes :constant 0.32 mM H2O2 (120 min) redo(1)
(c) 532. Brown enviromental changes :constant 0.32 mM H2O2 (160 min) redo(1)
(c) 534. Brown enviromental changes :1 mM Menadione (20 min) redo(1)
(c) 535. Brown enviromental changes :1 mM Menadione (30 min) redo(1)
(c) 536. Brown enviromental changes :1mM Menadione (40 min) redo(1)
(c) 537. Brown enviromental changes :1 mM Menadione (50 min)redo(1)
(c) 539. Brown enviromental changes :1 mM Menadione (105 min) redo(1)
(c) 541. Brown enviromental changes :1 mM Menadione (160 min) redo(1)
(c) 628. Brown enviromental changes :DBY7286 + 0.3 mM H2O2 (20 min)(1)
(c) 635. Brown enviromental changes :YAP1 overexpression(1)
(c) DES460 + 0.02% MMS - 15 min
(c) DES460 + 0.02% MMS - 120 min

YAP1 -*-> GTT2

(c) 13. Expression in cells overexpressing Yap1p(1)
(c) 445. Expression in response to 0.1% MMS for 60 min (average of 3 experiments)(1)
(c) 447. Expression in response to 0.1% MMS for 30 min(1)
(c) 448. Expression in response to 0.1% MMS for 60 min(1)
(c) 449. Expression in response to 0.1% MMS for 60 min(1)
(c) 450. Expression in response to low MNNG (8 microgram/ml) for 60 min(1)
(c) 452. Expression in response to low 4NQO (2 microgram/ml) for 60 min(1)
(c) 464. Expression in response to 0.2% MMS for 60 min(1)
(c) 485. Expression in response to peroxide: 10,20,40,60,120 min(3)
(c) 513. Brown enviromental changes :29C to 33C - 5 minutes(1)
(c) 523. Brown enviromental changes :constant 0.32 mM H2O2 (10 min) redo(1)
(c) 524. Brown enviromental changes :constant 0.32 mM H2O2 (20 min) redo(1)
(c) 525. Brown enviromental changes :constant 0.32 mM H2O2 (30 min) redo(1)
(c) 526. Brown enviromental changes :constant 0.32 mM H2O2 (40 min) rescan(1)
(c) 527. Brown enviromental changes :constant 0.32 mM H2O2 (50 min) redo(1)
(c) 528. Brown enviromental changes :constant 0.32 mM H2O2 (60 min) redo(1)
(c) 529. Brown enviromental changes :constant 0.32 mM H2O2 (80 min) redo(1)
(c) 530. Brown enviromental changes :constant 0.32 mM H2O2 (100 min) redo(1)
(c) 531. Brown enviromental changes :constant 0.32 mM H2O2 (120 min) redo(1)
(c) 532. Brown enviromental changes :constant 0.32 mM H2O2 (160 min) redo(1)
(c) 534. Brown enviromental changes :1 mM Menadione (20 min) redo(1)
(c) 535. Brown enviromental changes :1 mM Menadione (30 min) redo(1)
(c) 536. Brown enviromental changes :1mM Menadione (40 min) redo(1)
(c) 537. Brown enviromental changes :1 mM Menadione (50 min)redo(1)
(c) 538. Brown enviromental changes :1 mM Menadione (80 min) redo(1)
(c) 539. Brown enviromental changes :1 mM Menadione (105 min) redo(1)
(c) 628. Brown enviromental changes :DBY7286 + 0.3 mM H2O2 (20 min)(1)
(c) 635. Brown enviromental changes :YAP1 overexpression(1)
(c) DES460 + 0.2% MMS - 45 min

YAP1 -*-> YFL057C

(c) 13. Expression in cells overexpressing Yap1p(1)
(c) 445. Expression in response to 0.1% MMS for 60 min (average of 3 experiments)(1)
(c) 447. Expression in response to 0.1% MMS for 30 min(1)
(c) 448. Expression in response to 0.1% MMS for 60 min(1)
(c) 449. Expression in response to 0.1% MMS for 60 min(1)
(c) 450. Expression in response to low MNNG (8 microgram/ml) for 60 min(1)
(c) 452. Expression in response to low 4NQO (2 microgram/ml) for 60 min(1)
(c) 464. Expression in response to 0.2% MMS for 60 min(1)
(c) 485. Expression in response to peroxide: 10,20,40,60,120 min(3)
(c) 513. Brown enviromental changes :29C to 33C - 5 minutes(1)
(c) 523. Brown enviromental changes :constant 0.32 mM H2O2 (10 min) redo(1)
(c) 524. Brown enviromental changes :constant 0.32 mM H2O2 (20 min) redo(1)
(c) 525. Brown enviromental changes :constant 0.32 mM H2O2 (30 min) redo(1)
(c) 526. Brown enviromental changes :constant 0.32 mM H2O2 (40 min) rescan(1)
(c) 527. Brown enviromental changes :constant 0.32 mM H2O2 (50 min) redo(1)
(c) 528. Brown enviromental changes :constant 0.32 mM H2O2 (60 min) redo(1)
(c) 529. Brown enviromental changes :constant 0.32 mM H2O2 (80 min) redo(1)
(c) 530. Brown enviromental changes :constant 0.32 mM H2O2 (100 min) redo(1)
(c) 531. Brown enviromental changes :constant 0.32 mM H2O2 (120 min) redo(1)
(c) 532. Brown enviromental changes :constant 0.32 mM H2O2 (160 min) redo(1)
(c) 534. Brown enviromental changes :1 mM Menadione (20 min) redo(1)
(c) 535. Brown enviromental changes :1 mM Menadione (30 min) redo(1)
(c) 536. Brown enviromental changes :1mM Menadione (40 min) redo(1)
(c) 537. Brown enviromental changes :1 mM Menadione (50 min)redo(1)
(c) 538. Brown enviromental changes :1 mM Menadione (80 min) redo(1)
(c) 539. Brown enviromental changes :1 mM Menadione (105 min) redo(1)
(c) 628. Brown enviromental changes :DBY7286 + 0.3 mM H2O2 (20 min)(1)
(c) 635. Brown enviromental changes :YAP1 overexpression(1)
(c) DES460 + 0.2% MMS - 45 min

YAP1 -*-> YLR387C

(c) 13. Expression in cells overexpressing Yap1p(1)
(c) 399. Rosetta 2000: Expression in response to FR901,228(1)
(c) 445. Expression in response to 0.1% MMS for 60 min (average of 3 experiments)(1)
(c) 446. Expression in response to 0.1% MMS for 10 min(1)
(c) 447. Expression in response to 0.1% MMS for 30 min(1)
(c) 448. Expression in response to 0.1% MMS for 60 min(1)
(c) 449. Expression in response to 0.1% MMS for 60 min(1)
(c) 451. Expression in response to BCNU (200 micromolar) for 60 min(1)
(c) 462. Expression in response to 0.05% MMS for 60 min(1)
(c) 463. Expression in response to 0.1% MMS for 60 min(1)
(c) 464. Expression in response to 0.2% MMS for 60 min(1)
(c) 523. Brown enviromental changes :constant 0.32 mM H2O2 (10 min) redo(1)
(c) 524. Brown enviromental changes :constant 0.32 mM H2O2 (20 min) redo(1)
(c) 525. Brown enviromental changes :constant 0.32 mM H2O2 (30 min) redo(1)
(c) 528. Brown enviromental changes :constant 0.32 mM H2O2 (60 min) redo(1)
(c) 534. Brown enviromental changes :1 mM Menadione (20 min) redo(1)

(c) 535. Brown enviromental changes :1 mM Menadione (30 min) redo(1)
(c) 557. Brown enviromental changes :1.5 mM diamide (5 min)(1)
(c) 558. Brown enviromental changes :1.5 mM diamide (10 min)(1)
(c) 628. Brown enviromental changes :DBY7286 + 0.3 mM H2O2 (20 min)(1)
(c) 635. Brown enviromental changes :YAP1 overexpression(1)
(c) DES460 + 0.02% MMS - 30 min
(c) DES460 + 0.2% MMS - 45 min
(c) DES460 + 0.02% MMS - 60 min
(c) DES460 + 0.02% MMS - 90 min

YAP1 -*-> YLR460C

(c) 13. Expression in cells overexpressing Yap1p(1)
(c) 445. Expression in response to 0.1% MMS for 60 min (average of 3 experiments)(1)
(c) 446. Expression in response to 0.1% MMS for 10 min(1)
(c) 447. Expression in response to 0.1% MMS for 30 min(1)
(c) 448. Expression in response to 0.1% MMS for 60 min(1)
(c) 449. Expression in response to 0.1% MMS for 60 min(1)
(c) 450. Expression in response to low MNNG (8 microgram/ml) for 60 min(1)
(c) 451. Expression in response to BCNU (200 micromolar) for 60 min(1)
(c) 452. Expression in response to low 4NQO (2 microgram/ml) for 60 min(1)
(c) 454. Expression in response to tBuOOH (5mM) for 60 min(1)
(c) 463. Expression in response to 0.1% MMS for 60 min(1)
(c) 464. Expression in response to 0.2% MMS for 60 min(1)
(c) 485. Expression in response to peroxide: 10,20,40,60,120 min(3)
(c) 524. Brown enviromental changes :constant 0.32 mM H2O2 (20 min) redo(1)
(c) 525. Brown enviromental changes :constant 0.32 mM H2O2 (30 min) redo(1)
(c) 527. Brown enviromental changes :constant 0.32 mM H2O2 (50 min) redo(1)
(c) 528. Brown enviromental changes :constant 0.32 mM H2O2 (60 min) redo(1)
(c) 534. Brown enviromental changes :1 mM Menadione (20 min) redo(1)
(c) 535. Brown enviromental changes :1 mM Menadione (30 min) redo(1)
(c) 536. Brown enviromental changes :1mM Menadione (40 min) redo(1)
(c) 537. Brown enviromental changes :1 mM Menadione (50 min)redo(1)
(c) 539. Brown enviromental changes :1 mM Menadione (105 min) redo(1)
(c) 540. Brown enviromental changes :1 mM Menadione (120 min)redo(1)
(c) 557. Brown enviromental changes :1.5 mM diamide (5 min)(1)
(c) 558. Brown enviromental changes :1.5 mM diamide (10 min)(1)
(c) 628. Brown enviromental changes :DBY7286 + 0.3 mM H2O2 (20 min)(1)
(c) 635. Brown enviromental changes :YAP1 overexpression(1)
(c) DES460 + 0.02% MMS - 30 min
(c) DES460 + 0.2% MMS - 45 min

YAP1 -*-> CYT2

(c) 13. Expression in cells overexpressing Yap1p(1)
(c) 445. Expression in response to 0.1% MMS for 60 min (average of 3 experiments)(1)
(c) 447. Expression in response to 0.1% MMS for 30 min(1)
(c) 448. Expression in response to 0.1% MMS for 60 min(1)
(c) 449. Expression in response to 0.1% MMS for 60 min(1)
(c) 464. Expression in response to 0.2% MMS for 60 min(1)
(c) 481. Expression in response to heat shock: 15,30,45,60,120 min(2)
(c) 481. Expression in response to heat shock: 15,30,45,60,120 min(4)
(c) 523. Brown enviromental changes :constant 0.32 mM H2O2 (10 min) redo(1)
(c) 525. Brown enviromental changes :constant 0.32 mM H2O2 (30 min) redo(1)
(c) 527. Brown enviromental changes :constant 0.32 mM H2O2 (50 min) redo(1)
(c) 528. Brown enviromental changes :constant 0.32 mM H2O2 (60 min) redo(1)
(c) 529. Brown enviromental changes :constant 0.32 mM H2O2 (80 min) redo(1)
(c) 530. Brown enviromental changes :constant 0.32 mM H2O2 (100 min) redo(1)
(c) 531. Brown enviromental changes :constant 0.32 mM H2O2 (120 min) redo(1)
(c) 532. Brown enviromental changes :constant 0.32 mM H2O2 (160 min) redo(1)
(c) 534. Brown enviromental changes :1 mM Menadione (20 min) redo(1)
(c) 535. Brown enviromental changes :1 mM Menadione (30 min) redo(1)
(c) 536. Brown enviromental changes :1mM Menadione (40 min) redo(1)
(c) 537. Brown enviromental changes :1 mM Menadione (50 min)redo(1)
(c) 544. Brown enviromental changes :2.5mM DTT 030 min dtt-1(1)
(c) 545. Brown enviromental changes :2.5mM DTT 045 min dtt-1(1)
(c) 546. Brown enviromental changes :2.5mM DTT 060 min dtt-1(1)
(c) 547. Brown enviromental changes :2.5mM DTT 090 min dtt-1(1)
(c) 557. Brown enviromental changes :1.5 mM diamide (5 min)(1)
(c) 558. Brown enviromental changes :1.5 mM diamide (10 min)(1)
(c) 559. Brown enviromental changes :1.5 mM diamide (20 min)(1)
(c) 561. Brown enviromental changes :1.5 mM diamide (40 min)(1)
(c) 562. Brown enviromental changes :1.5 mM diamide (50 min)(1)
(c) 563. Brown enviromental changes :1.5 mM diamide (60 min)(1)
(c) 564. Brown enviromental changes :1.5 mM diamide (90 min)(1)
(c) 628. Brown enviromental changes :DBY7286 + 0.3 mM H2O2 (20 min)(1)
(c) 635. Brown enviromental changes :YAP1 overexpression(1)
(c) DES460 + 0.02% MMS - 30 min
(c) DES460 + 0.02% MMS - 60 min
