# Supplementary material for: Unraveling condition specific gene transcriptional regulatory networks in Saccharomyces cerevisiae
Source: BMC Bioinformatics. 2006 Mar 21;7:165. doi: 10.1186/1471-2105-7-165 (PMC1488875; doi:10.1186/1471-2105-7-165)
Supplement: Additional File 8 — Predicted transcriptional regulatory links obtained by applying the STAR model to Alon's network. Each link is accompanied by a list of experiments in which it is likely to be functional. [file 1471-2105-7-165-S8.pdf]

Predicted transcriptional regulatory links obtained by applying the STAR model to Alon's network (Milo, R. et al. Science 298, 824-7, 2002).

Each link in this file (e.g. ABF1 -*-> EFB1) is followed by a list of the predicted experimental conditions in which it is likely to be active.

ABF1 -*-> EFB1

(c) 7. Expression during the cell Cycle (cdc28)(5)
(c) 7. Expression during the cell Cycle (cdc28)(14)
(c) 7. Expression during the cell Cycle (cdc28)(15)
(c) 7. Expression during the cell Cycle (cdc28)(16)
(c) 7. Expression during the cell Cycle (cdc28)(17)
(c) 8. Expression during the cell cycle (cell size selection and release)(9)
(c) 8. Expression during the cell cycle (cell size selection and release)(10)
(c) 11. Expression during diauxic shift: 9h,11h,13h,15h,17h,19h,21h(3)
(c) 388. Rosetta 2000: Expression in cells with FKS1 under tet promoter(1)
(c) 390. Rosetta 2000: Expression in cells with IDI1 under tet promoter(1)
(c) 391. Rosetta 2000: Expression in cells with KAR2 under tet promoter(1)
(c) 393. Rosetta 2000: Expression in cells with RHO1 under tet promoter(1)
(c) 496. Brown enviromental changes :Heat Shock 000 minutes hs-2(1)
(c) 497. Brown enviromental changes :Heat Shock 000 minutes hs-2(1)
(c) 498. Brown enviromental changes :Heat Shock 000 minutes hs-2(1)
(c) 506. Brown enviromental changes :37C to 25C shock - 60 min(1)
(c) 507. Brown enviromental changes :37C to 25C shock - 90 min(1)
(c) 516. Brown enviromental changes :33C vs. 30C - 90 minutes(1)
(c) 550. Brown enviromental changes :dtt 000 min dtt-2(1)
(c) 551. Brown enviromental changes :dtt 015 min dtt-2(1)
(c) 552. Brown enviromental changes :dtt 030 min dtt-2(1)
(c) 595. Brown enviromental changes :diauxic shift timecourse(1)
(c) 672. Expression in response to carbonyl cyanide m-chlorophenylhydrazone (CCCP) 90min(1)
(c) 674. Expression in response to oligomycin 120min(1)
(c) DES460 (wt) - mock irradiation - 30 min

ABF1 -*-> RPP0

(c) 7. Expression during the cell Cycle (cdc28)(5)
(c) 7. Expression during the cell Cycle (cdc28)(14)
(c) 7. Expression during the cell Cycle (cdc28)(15)
(c) 7. Expression during the cell Cycle (cdc28)(16)
(c) 7. Expression during the cell Cycle (cdc28)(17)
(c) 8. Expression during the cell cycle (cell size selection and release)(9)
(c) 8. Expression during the cell cycle (cell size selection and release)(10)
(c) 11. Expression during diauxic shift: 9h,11h,13h,15h,17h,19h,21h(3)
(c) 388. Rosetta 2000: Expression in cells with FKS1 under tet promoter(1)
(c) 390. Rosetta 2000: Expression in cells with IDI1 under tet promoter(1)
(c) 391. Rosetta 2000: Expression in cells with KAR2 under tet promoter(1)
(c) 393. Rosetta 2000: Expression in cells with RHO1 under tet promoter(1)
(c) 496. Brown enviromental changes :Heat Shock 000 minutes hs-2(1)
(c) 497. Brown enviromental changes :Heat Shock 000 minutes hs-2(1)
(c) 498. Brown enviromental changes :Heat Shock 000 minutes hs-2(1)
(c) 506. Brown enviromental changes :37C to 25C shock - 60 min(1)
(c) 507. Brown enviromental changes :37C to 25C shock - 90 min(1)
(c) 516. Brown enviromental changes :33C vs. 30C - 90 minutes(1)
(c) 550. Brown enviromental changes :dtt 000 min dtt-2(1)
(c) 551. Brown enviromental changes :dtt 015 min dtt-2(1)
(c) 552. Brown enviromental changes :dtt 030 min dtt-2(1)
(c) 595. Brown enviromental changes :diauxic shift timecourse(1)
(c) 672. Expression in response to carbonyl cyanide m-chlorophenylhydrazone (CCCP) 90min(1)
(c) 674. Expression in response to oligomycin 120min(1)
(c) DES460 (wt) - mock irradiation - 30 min

ABF1 -*-> RPS28A

(c) 7. Expression during the cell Cycle (cdc28)(5)
(c) 7. Expression during the cell Cycle (cdc28)(14)
(c) 7. Expression during the cell Cycle (cdc28)(15)
(c) 7. Expression during the cell Cycle (cdc28)(16)
(c) 7. Expression during the cell Cycle (cdc28)(17)
(c) 8. Expression during the cell cycle (cell size selection and release)(9)
(c) 8. Expression during the cell cycle (cell size selection and release)(10)
(c) 11. Expression during diauxic shift: 9h,11h,13h,15h,17h,19h,21h(3)
(c) 388. Rosetta 2000: Expression in cells with FKS1 under tet promoter(1)
(c) 390. Rosetta 2000: Expression in cells with IDI1 under tet promoter(1)
(c) 391. Rosetta 2000: Expression in cells with KAR2 under tet promoter(1)
(c) 393. Rosetta 2000: Expression in cells with RHO1 under tet promoter(1)
(c) 496. Brown enviromental changes :Heat Shock 000 minutes hs-2(1)
(c) 497. Brown enviromental changes :Heat Shock 000 minutes hs-2(1)
(c) 498. Brown enviromental changes :Heat Shock 000 minutes hs-2(1)
(c) 506. Brown enviromental changes :37C to 25C shock - 60 min(1)
(c) 507. Brown enviromental changes :37C to 25C shock - 90 min(1)
(c) 516. Brown enviromental changes :33C vs. 30C - 90 minutes(1)
(c) 550. Brown enviromental changes :dtt 000 min dtt-2(1)
(c) 551. Brown enviromental changes :dtt 015 min dtt-2(1)
(c) 552. Brown enviromental changes :dtt 030 min dtt-2(1)
(c) 595. Brown enviromental changes :diauxic shift timecourse(1)
(c) 672. Expression in response to carbonyl cyanide m-chlorophenylhydrazone (CCCP) 90min(1)
(c) 674. Expression in response to oligomycin 120min(1)
(c) DES460 (wt) - mock irradiation - 30 min

ACE2 -*-| BUD9

(c) 5. Expression during the cell cycle (alpha factor arrest and release)(11)
(c) 5. Expression during the cell cycle (alpha factor arrest and release)(12)
(c) 5. Expression during the cell cycle (alpha factor arrest and release)(13)
(c) 5. Expression during the cell cycle (alpha factor arrest and release)(15)
(c) 5. Expression during the cell cycle (alpha factor arrest and release)(16)
(c) 6. Expression during the cell cycle (cdc15 arrest and release)(2)
(c) 6. Expression during the cell cycle (cdc15 arrest and release)(10)
(c) 6. Expression during the cell cycle (cdc15 arrest and release)(11)
(c) 6. Expression during the cell cycle (cdc15 arrest and release)(12)
(c) 6. Expression during the cell cycle (cdc15 arrest and release)(13)
(c) 6. Expression during the cell cycle (cdc15 arrest and release)(22)
(c) 6. Expression during the cell cycle (cdc15 arrest and release)(23)
(c) 6. Expression during the cell cycle (cdc15 arrest and release)(24)
(c) 7. Expression during the cell Cycle (cdc28)(10)

(c) 7. Expression during the cell Cycle (cdc28)(11)
(c) 7. Expression during the cell Cycle (cdc28)(12)
(c) 8. Expression during the cell cycle (cell size selection and release)(1)
(c) 8. Expression during the cell cycle (cell size selection and release)(12)
(c) 8. Expression during the cell cycle (cell size selection and release)(13)
(c) 8. Expression during the cell cycle (cell size selection and release)(14)

ACE2 -*-| CST13

(c) 5. Expression during the cell cycle (alpha factor arrest and release)(11)
(c) 5. Expression during the cell cycle (alpha factor arrest and release)(12)
(c) 5. Expression during the cell cycle (alpha factor arrest and release)(13)
(c) 5. Expression during the cell cycle (alpha factor arrest and release)(15)
(c) 5. Expression during the cell cycle (alpha factor arrest and release)(16)
(c) 6. Expression during the cell cycle (cdc15 arrest and release)(2)
(c) 6. Expression during the cell cycle (cdc15 arrest and release)(10)
(c) 6. Expression during the cell cycle (cdc15 arrest and release)(11)
(c) 6. Expression during the cell cycle (cdc15 arrest and release)(12)
(c) 6. Expression during the cell cycle (cdc15 arrest and release)(13)
(c) 6. Expression during the cell cycle (cdc15 arrest and release)(22)
(c) 6. Expression during the cell cycle (cdc15 arrest and release)(23)
(c) 6. Expression during the cell cycle (cdc15 arrest and release)(24)
(c) 7. Expression during the cell Cycle (cdc28)(10)
(c) 7. Expression during the cell Cycle (cdc28)(11)
(c) 7. Expression during the cell Cycle (cdc28)(12)
(c) 8. Expression during the cell cycle (cell size selection and release)(1)
(c) 8. Expression during the cell cycle (cell size selection and release)(12)
(c) 8. Expression during the cell cycle (cell size selection and release)(13)
(c) 8. Expression during the cell cycle (cell size selection and release)(14)

ACE2 -*-| FAA3

(c) 5. Expression during the cell cycle (alpha factor arrest and release)(11)
(c) 5. Expression during the cell cycle (alpha factor arrest and release)(12)
(c) 5. Expression during the cell cycle (alpha factor arrest and release)(13)
(c) 5. Expression during the cell cycle (alpha factor arrest and release)(15)
(c) 5. Expression during the cell cycle (alpha factor arrest and release)(16)
(c) 6. Expression during the cell cycle (cdc15 arrest and release)(2)
(c) 6. Expression during the cell cycle (cdc15 arrest and release)(10)
(c) 6. Expression during the cell cycle (cdc15 arrest and release)(11)
(c) 6. Expression during the cell cycle (cdc15 arrest and release)(12)
(c) 6. Expression during the cell cycle (cdc15 arrest and release)(13)
(c) 6. Expression during the cell cycle (cdc15 arrest and release)(22)
(c) 6. Expression during the cell cycle (cdc15 arrest and release)(23)
(c) 6. Expression during the cell cycle (cdc15 arrest and release)(24)
(c) 7. Expression during the cell Cycle (cdc28)(10)
(c) 7. Expression during the cell Cycle (cdc28)(11)
(c) 7. Expression during the cell Cycle (cdc28)(12)
(c) 8. Expression during the cell cycle (cell size selection and release)(1)
(c) 8. Expression during the cell cycle (cell size selection and release)(12)
(c) 8. Expression during the cell cycle (cell size selection and release)(13)
(c) 8. Expression during the cell cycle (cell size selection and release)(14)

ACE2 -*-| JIP1

(c) 5. Expression during the cell cycle (alpha factor arrest and release)(11)
(c) 5. Expression during the cell cycle (alpha factor arrest and release)(12)
(c) 5. Expression during the cell cycle (alpha factor arrest and release)(13)
(c) 5. Expression during the cell cycle (alpha factor arrest and release)(15)
(c) 5. Expression during the cell cycle (alpha factor arrest and release)(16)
(c) 6. Expression during the cell cycle (cdc15 arrest and release)(2)
(c) 6. Expression during the cell cycle (cdc15 arrest and release)(10)
(c) 6. Expression during the cell cycle (cdc15 arrest and release)(11)
(c) 6. Expression during the cell cycle (cdc15 arrest and release)(12)
(c) 6. Expression during the cell cycle (cdc15 arrest and release)(13)
(c) 6. Expression during the cell cycle (cdc15 arrest and release)(22)
(c) 6. Expression during the cell cycle (cdc15 arrest and release)(23)
(c) 6. Expression during the cell cycle (cdc15 arrest and release)(24)
(c) 7. Expression during the cell Cycle (cdc28)(10)
(c) 7. Expression during the cell Cycle (cdc28)(11)
(c) 7. Expression during the cell Cycle (cdc28)(12)
(c) 8. Expression during the cell cycle (cell size selection and release)(1)
(c) 8. Expression during the cell cycle (cell size selection and release)(12)
(c) 8. Expression during the cell cycle (cell size selection and release)(13)
(c) 8. Expression during the cell cycle (cell size selection and release)(14)

ACE2 -*-| PIR1

(c) 5. Expression during the cell cycle (alpha factor arrest and release)(11)
(c) 5. Expression during the cell cycle (alpha factor arrest and release)(12)
(c) 5. Expression during the cell cycle (alpha factor arrest and release)(13)
(c) 5. Expression during the cell cycle (alpha factor arrest and release)(15)
(c) 5. Expression during the cell cycle (alpha factor arrest and release)(16)
(c) 6. Expression during the cell cycle (cdc15 arrest and release)(2)
(c) 6. Expression during the cell cycle (cdc15 arrest and release)(10)
(c) 6. Expression during the cell cycle (cdc15 arrest and release)(11)
(c) 6. Expression during the cell cycle (cdc15 arrest and release)(12)
(c) 6. Expression during the cell cycle (cdc15 arrest and release)(13)
(c) 6. Expression during the cell cycle (cdc15 arrest and release)(22)
(c) 6. Expression during the cell cycle (cdc15 arrest and release)(23)
(c) 6. Expression during the cell cycle (cdc15 arrest and release)(24)
(c) 7. Expression during the cell Cycle (cdc28)(10)
(c) 7. Expression during the cell Cycle (cdc28)(11)
(c) 7. Expression during the cell Cycle (cdc28)(12)
(c) 8. Expression during the cell cycle (cell size selection and release)(1)
(c) 8. Expression during the cell cycle (cell size selection and release)(12)
(c) 8. Expression during the cell cycle (cell size selection and release)(13)
(c) 8. Expression during the cell cycle (cell size selection and release)(14)

ACE2 -*-| PRY3

(c) 5. Expression during the cell cycle (alpha factor arrest and release)(11)

(c) 5. Expression during the cell cycle (alpha factor arrest and release)(12)
(c) 5. Expression during the cell cycle (alpha factor arrest and release)(13)
(c) 5. Expression during the cell cycle (alpha factor arrest and release)(15)
(c) 5. Expression during the cell cycle (alpha factor arrest and release)(16)
(c) 6. Expression during the cell cycle (cdc15 arrest and release)(2)
(c) 6. Expression during the cell cycle (cdc15 arrest and release)(10)
(c) 6. Expression during the cell cycle (cdc15 arrest and release)(11)
(c) 6. Expression during the cell cycle (cdc15 arrest and release)(12)
(c) 6. Expression during the cell cycle (cdc15 arrest and release)(13)
(c) 6. Expression during the cell cycle (cdc15 arrest and release)(22)
(c) 6. Expression during the cell cycle (cdc15 arrest and release)(23)
(c) 6. Expression during the cell cycle (cdc15 arrest and release)(24)
(c) 7. Expression during the cell Cycle (cdc28)(10)
(c) 7. Expression during the cell Cycle (cdc28)(11)
(c) 7. Expression during the cell Cycle (cdc28)(12)
(c) 8. Expression during the cell cycle (cell size selection and release)(1)
(c) 8. Expression during the cell cycle (cell size selection and release)(12)
(c) 8. Expression during the cell cycle (cell size selection and release)(13)
(c) 8. Expression during the cell cycle (cell size selection and release)(14)

ACE2 -*-| SCW11

(c) 5. Expression during the cell cycle (alpha factor arrest and release)(11)
(c) 5. Expression during the cell cycle (alpha factor arrest and release)(12)
(c) 5. Expression during the cell cycle (alpha factor arrest and release)(13)
(c) 5. Expression during the cell cycle (alpha factor arrest and release)(15)
(c) 5. Expression during the cell cycle (alpha factor arrest and release)(16)
(c) 6. Expression during the cell cycle (cdc15 arrest and release)(2)
(c) 6. Expression during the cell cycle (cdc15 arrest and release)(10)
(c) 6. Expression during the cell cycle (cdc15 arrest and release)(11)
(c) 6. Expression during the cell cycle (cdc15 arrest and release)(12)
(c) 6. Expression during the cell cycle (cdc15 arrest and release)(13)
(c) 6. Expression during the cell cycle (cdc15 arrest and release)(22)
(c) 6. Expression during the cell cycle (cdc15 arrest and release)(23)
(c) 6. Expression during the cell cycle (cdc15 arrest and release)(24)
(c) 7. Expression during the cell Cycle (cdc28)(10)
(c) 7. Expression during the cell Cycle (cdc28)(11)
(c) 7. Expression during the cell Cycle (cdc28)(12)
(c) 8. Expression during the cell cycle (cell size selection and release)(1)
(c) 8. Expression during the cell cycle (cell size selection and release)(12)
(c) 8. Expression during the cell cycle (cell size selection and release)(13)
(c) 8. Expression during the cell cycle (cell size selection and release)(14)

ACE2 -*-| YER124C

(c) 5. Expression during the cell cycle (alpha factor arrest and release)(11)
(c) 5. Expression during the cell cycle (alpha factor arrest and release)(12)
(c) 5. Expression during the cell cycle (alpha factor arrest and release)(13)
(c) 5. Expression during the cell cycle (alpha factor arrest and release)(15)
(c) 5. Expression during the cell cycle (alpha factor arrest and release)(16)
(c) 6. Expression during the cell cycle (cdc15 arrest and release)(2)
(c) 6. Expression during the cell cycle (cdc15 arrest and release)(10)
(c) 6. Expression during the cell cycle (cdc15 arrest and release)(11)
(c) 6. Expression during the cell cycle (cdc15 arrest and release)(12)
(c) 6. Expression during the cell cycle (cdc15 arrest and release)(13)
(c) 6. Expression during the cell cycle (cdc15 arrest and release)(22)
(c) 6. Expression during the cell cycle (cdc15 arrest and release)(23)
(c) 6. Expression during the cell cycle (cdc15 arrest and release)(24)
(c) 7. Expression during the cell Cycle (cdc28)(10)
(c) 7. Expression during the cell Cycle (cdc28)(11)
(c) 7. Expression during the cell Cycle (cdc28)(12)
(c) 8. Expression during the cell cycle (cell size selection and release)(1)
(c) 8. Expression during the cell cycle (cell size selection and release)(12)
(c) 8. Expression during the cell cycle (cell size selection and release)(13)
(c) 8. Expression during the cell cycle (cell size selection and release)(14)

ACE2 -*-| YHR143W

(c) 5. Expression during the cell cycle (alpha factor arrest and release)(11)
(c) 5. Expression during the cell cycle (alpha factor arrest and release)(12)
(c) 5. Expression during the cell cycle (alpha factor arrest and release)(13)
(c) 5. Expression during the cell cycle (alpha factor arrest and release)(15)
(c) 5. Expression during the cell cycle (alpha factor arrest and release)(16)
(c) 6. Expression during the cell cycle (cdc15 arrest and release)(2)
(c) 6. Expression during the cell cycle (cdc15 arrest and release)(10)
(c) 6. Expression during the cell cycle (cdc15 arrest and release)(11)
(c) 6. Expression during the cell cycle (cdc15 arrest and release)(12)
(c) 6. Expression during the cell cycle (cdc15 arrest and release)(13)
(c) 6. Expression during the cell cycle (cdc15 arrest and release)(22)
(c) 6. Expression during the cell cycle (cdc15 arrest and release)(23)
(c) 6. Expression during the cell cycle (cdc15 arrest and release)(24)
(c) 7. Expression during the cell Cycle (cdc28)(10)
(c) 7. Expression during the cell Cycle (cdc28)(11)
(c) 7. Expression during the cell Cycle (cdc28)(12)
(c) 8. Expression during the cell cycle (cell size selection and release)(1)
(c) 8. Expression during the cell cycle (cell size selection and release)(12)
(c) 8. Expression during the cell cycle (cell size selection and release)(13)
(c) 8. Expression during the cell cycle (cell size selection and release)(14)

ACE2 -*-| YLR049C

(c) 5. Expression during the cell cycle (alpha factor arrest and release)(11)
(c) 5. Expression during the cell cycle (alpha factor arrest and release)(12)
(c) 5. Expression during the cell cycle (alpha factor arrest and release)(13)
(c) 5. Expression during the cell cycle (alpha factor arrest and release)(15)
(c) 5. Expression during the cell cycle (alpha factor arrest and release)(16)
(c) 6. Expression during the cell cycle (cdc15 arrest and release)(2)
(c) 6. Expression during the cell cycle (cdc15 arrest and release)(10)
(c) 6. Expression during the cell cycle (cdc15 arrest and release)(11)
(c) 6. Expression during the cell cycle (cdc15 arrest and release)(12)
(c) 6. Expression during the cell cycle (cdc15 arrest and release)(13)
(c) 6. Expression during the cell cycle (cdc15 arrest and release)(22)
(c) 6. Expression during the cell cycle (cdc15 arrest and release)(23)
(c) 6. Expression during the cell cycle (cdc15 arrest and release)(24)

```
    (c) 7. Expression during the cell Cycle (cdc28)(10)
    (c) 7. Expression during the cell Cycle (cdc28)(11)
    (c) 7. Expression during the cell Cycle (cdc28)(12)
    (c) 8. Expression during the cell cycle (cell size selection and release)(1)
    (c) 8. Expression during the cell cycle (cell size selection and release)(12)
    (c) 8. Expression during the cell cycle (cell size selection and release)(13)
    (c) 8. Expression during the cell cycle (cell size selection and release)(14)


ADR1 -*-> CAT2


    (c) 6. Expression during the cell cycle (cdc15 arrest and release)(15)
    (c) 8. Expression during the cell cycle (cell size selection and release)(9)
    (c) 8. Expression during the cell cycle (cell size selection and release)(12)
    (c) 8. Expression during the cell cycle (cell size selection and release)(13)
    (c) 8. Expression during the cell cycle (cell size selection and release)(14)
    (c) 11. Expression during diauxic shift: 9h,11h,13h,15h,17h,19h,21h(7)
    (c) 392. Rosetta 2000: Expression in cells with PMA1 under tet promoter(1)
    (c) 399. Rosetta 2000: Expression in response to FR901,228(1)
    (c) 430. Expression in strain PM38 (wild type), glucose versus ethanol: strain was shifted from medium containing dextrose as carbon source, ammonium sulfate as nirogen source, 
supplemented with leucine and uracil to same medium for 30 min, compared to a shift to a medium with synthetic ethanol instead of glucose for 30 min(1)
    (c) 588. Brown enviromental changes :Nitrogen Depletion 12 h(1)
    (c) 589. Brown enviromental changes :Nitrogen Depletion 1 d(1)
    (c) 590. Brown enviromental changes :Nitrogen Depletion 2 d(1)
    (c) 591. Brown enviromental changes :Nitrogen Depletion 3 d(1)
    (c) 599. Brown enviromental changes :diauxic shift timecourse(1)
    (c) 600. Brown enviromental changes :diauxic shift timecourse(1)
    (c) 604. Brown enviromental changes :YPD 8 h ypd-2(1)
    (c) 605. Brown enviromental changes :YPD 10 h  ypd-2(1)
    (c) 606. Brown enviromental changes :YPD 12 h ypd-2(1)
    (c) 607. Brown enviromental changes :YPD 1 d ypd-2(1)
    (c) 608. Brown enviromental changes :YPD 2 d ypd-2(1)
    (c) 609. Brown enviromental changes :YPD 3 d ypd-2(1)
    (c) 610. Brown enviromental changes :YPD 5 d ypd-2(1)
    (c) 615. Brown enviromental changes :YPD stationary phase 1 d ypd-1(1)
    (c) 616. Brown enviromental changes :YPD stationary phase 2 d ypd-1(1)
    (c) 618. Brown enviromental changes :YPD stationary phase 5 d ypd-1(1)
    (c) 619. Brown enviromental changes :YPD stationary phase 7 d ypd-1(1)
    (c) 620. Brown enviromental changes :YPD stationary phase 13 d ypd-1(1)
    (c) 621. Brown enviromental changes :YPD stationary phase 22 d ypd-1(1)
    (c) 622. Brown enviromental changes :YPD stationary phase 28 d ypd-1(1)
    (c) 670. Expression in response to antimycin 60min(1)
    (c) (Var.) Rich Media 2% Glucose YPD-Average wt  5mM aF, 30 min.
    (c) wt-gal


ADR1 -*-> CIT3


    (c) 6. Expression during the cell cycle (cdc15 arrest and release)(15)
    (c) 8. Expression during the cell cycle (cell size selection and release)(9)
    (c) 8. Expression during the cell cycle (cell size selection and release)(12)
    (c) 8. Expression during the cell cycle (cell size selection and release)(13)
    (c) 8. Expression during the cell cycle (cell size selection and release)(14)
    (c) 11. Expression during diauxic shift: 9h,11h,13h,15h,17h,19h,21h(7)
    (c) 392. Rosetta 2000: Expression in cells with PMA1 under tet promoter(1)
    (c) 399. Rosetta 2000: Expression in response to FR901,228(1)
    (c) 430. Expression in strain PM38 (wild type), glucose versus ethanol: strain was shifted from medium containing dextrose as carbon source, ammonium sulfate as nirogen source, 
supplemented with leucine and uracil to same medium for 30 min, compared to a shift to a medium with synthetic ethanol instead of glucose for 30 min(1)
    (c) 588. Brown enviromental changes :Nitrogen Depletion 12 h(1)
    (c) 589. Brown enviromental changes :Nitrogen Depletion 1 d(1)
    (c) 590. Brown enviromental changes :Nitrogen Depletion 2 d(1)
    (c) 591. Brown enviromental changes :Nitrogen Depletion 3 d(1)
    (c) 599. Brown enviromental changes :diauxic shift timecourse(1)
    (c) 600. Brown enviromental changes :diauxic shift timecourse(1)
    (c) 604. Brown enviromental changes :YPD 8 h ypd-2(1)
    (c) 605. Brown enviromental changes :YPD 10 h  ypd-2(1)
    (c) 606. Brown enviromental changes :YPD 12 h ypd-2(1)
    (c) 607. Brown enviromental changes :YPD 1 d ypd-2(1)
    (c) 608. Brown enviromental changes :YPD 2 d ypd-2(1)
    (c) 609. Brown enviromental changes :YPD 3 d ypd-2(1)
    (c) 610. Brown enviromental changes :YPD 5 d ypd-2(1)
    (c) 615. Brown enviromental changes :YPD stationary phase 1 d ypd-1(1)
    (c) 616. Brown enviromental changes :YPD stationary phase 2 d ypd-1(1)
    (c) 618. Brown enviromental changes :YPD stationary phase 5 d ypd-1(1)
    (c) 619. Brown enviromental changes :YPD stationary phase 7 d ypd-1(1)
    (c) 620. Brown enviromental changes :YPD stationary phase 13 d ypd-1(1)
    (c) 621. Brown enviromental changes :YPD stationary phase 22 d ypd-1(1)
    (c) 622. Brown enviromental changes :YPD stationary phase 28 d ypd-1(1)
    (c) 670. Expression in response to antimycin 60min(1)
    (c) (Var.) Rich Media 2% Glucose YPD-Average wt  5mM aF, 30 min.
    (c) wt-gal


ADR1 -*-> FBP1


    (c) 6. Expression during the cell cycle (cdc15 arrest and release)(15)
    (c) 8. Expression during the cell cycle (cell size selection and release)(9)
    (c) 8. Expression during the cell cycle (cell size selection and release)(12)
    (c) 8. Expression during the cell cycle (cell size selection and release)(13)
    (c) 8. Expression during the cell cycle (cell size selection and release)(14)
    (c) 11. Expression during diauxic shift: 9h,11h,13h,15h,17h,19h,21h(7)
    (c) 392. Rosetta 2000: Expression in cells with PMA1 under tet promoter(1)
    (c) 399. Rosetta 2000: Expression in response to FR901,228(1)
    (c) 430. Expression in strain PM38 (wild type), glucose versus ethanol: strain was shifted from medium containing dextrose as carbon source, ammonium sulfate as nirogen source, 
supplemented with leucine and uracil to same medium for 30 min, compared to a shift to a medium with synthetic ethanol instead of glucose for 30 min(1)
    (c) 588. Brown enviromental changes :Nitrogen Depletion 12 h(1)
    (c) 589. Brown enviromental changes :Nitrogen Depletion 1 d(1)
    (c) 590. Brown enviromental changes :Nitrogen Depletion 2 d(1)
    (c) 591. Brown enviromental changes :Nitrogen Depletion 3 d(1)
    (c) 599. Brown enviromental changes :diauxic shift timecourse(1)
    (c) 600. Brown enviromental changes :diauxic shift timecourse(1)
    (c) 604. Brown enviromental changes :YPD 8 h ypd-2(1)
    (c) 605. Brown enviromental changes :YPD 10 h  ypd-2(1)
    (c) 606. Brown enviromental changes :YPD 12 h ypd-2(1)
    (c) 607. Brown enviromental changes :YPD 1 d ypd-2(1)
    (c) 608. Brown enviromental changes :YPD 2 d ypd-2(1)
    (c) 609. Brown enviromental changes :YPD 3 d ypd-2(1)
    (c) 610. Brown enviromental changes :YPD 5 d ypd-2(1)
    (c) 615. Brown enviromental changes :YPD stationary phase 1 d ypd-1(1)
```

(c) 616. Brown enviromental changes :YPD stationary phase 2 d ypd-1(1)
(c) 618. Brown enviromental changes :YPD stationary phase 5 d ypd-1(1)
(c) 619. Brown enviromental changes :YPD stationary phase 7 d ypd-1(1)
(c) 620. Brown enviromental changes :YPD stationary phase 13 d ypd-1(1)
(c) 621. Brown enviromental changes :YPD stationary phase 22 d ypd-1(1)
(c) 622. Brown enviromental changes :YPD stationary phase 28 d ypd-1(1)
(c) 670. Expression in response to antimycin 60min(1)
(c) (Var.) Rich Media 2% Glucose YPD-Average wt 5mM aF, 30 min.
(c) wt-gal

ADR1 -*-> IDP2

(c) 6. Expression during the cell cycle (cdc15 arrest and release)(15)
(c) 8. Expression during the cell cycle (cell size selection and release)(9)
(c) 8. Expression during the cell cycle (cell size selection and release)(12)
(c) 8. Expression during the cell cycle (cell size selection and release)(13)
(c) 8. Expression during the cell cycle (cell size selection and release)(14)
(c) 11. Expression during diauxic shift: 9h,11h,13h,15h,17h,19h,21h(7)
(c) 392. Rosetta 2000: Expression in cells with PMA1 under tet promoter(1)
(c) 399. Rosetta 2000: Expression in response to FR901,228(1)
(c) 430. Expression in strain PM38 (wild type), glucose versus ethanol: strain was shifted from medium containing dextrose as carbon source, ammonium sulfate as nirogen source, supplemented with leucine and uracil to same medium for 30 min, compared to a shift to a medium with synthetic ethanol instead of glucose for 30 min(1)
(c) 588. Brown enviromental changes :Nitrogen Depletion 12 h(1)
(c) 589. Brown enviromental changes :Nitrogen Depletion 1 d(1)
(c) 590. Brown enviromental changes :Nitrogen Depletion 2 d(1)
(c) 591. Brown enviromental changes :Nitrogen Depletion 3 d(1)
(c) 599. Brown enviromental changes :diauxic shift timecourse(1)
(c) 600. Brown enviromental changes :diauxic shift timecourse(1)
(c) 604. Brown enviromental changes :YPD 8 h ypd-2(1)
(c) 605. Brown enviromental changes :YPD 10 h ypd-2(1)
(c) 606. Brown enviromental changes :YPD 12 h ypd-2(1)
(c) 607. Brown enviromental changes :YPD 1 d ypd-2(1)
(c) 608. Brown enviromental changes :YPD 2 d ypd-2(1)
(c) 609. Brown enviromental changes :YPD 3 d ypd-2(1)
(c) 610. Brown enviromental changes :YPD 5 d ypd-2(1)
(c) 615. Brown enviromental changes :YPD stationary phase 1 d ypd-1(1)
(c) 616. Brown enviromental changes :YPD stationary phase 2 d ypd-1(1)
(c) 618. Brown enviromental changes :YPD stationary phase 5 d ypd-1(1)
(c) 619. Brown enviromental changes :YPD stationary phase 7 d ypd-1(1)
(c) 620. Brown enviromental changes :YPD stationary phase 13 d ypd-1(1)
(c) 621. Brown enviromental changes :YPD stationary phase 22 d ypd-1(1)
(c) 622. Brown enviromental changes :YPD stationary phase 28 d ypd-1(1)
(c) 670. Expression in response to antimycin 60min(1)
(c) (Var.) Rich Media 2% Glucose YPD-Average wt 5mM aF, 30 min.
(c) wt-gal

ADR1 -*-> IDP3

(c) 6. Expression during the cell cycle (cdc15 arrest and release)(15)
(c) 8. Expression during the cell cycle (cell size selection and release)(9)
(c) 8. Expression during the cell cycle (cell size selection and release)(12)
(c) 8. Expression during the cell cycle (cell size selection and release)(13)
(c) 8. Expression during the cell cycle (cell size selection and release)(14)
(c) 11. Expression during diauxic shift: 9h,11h,13h,15h,17h,19h,21h(7)
(c) 392. Rosetta 2000: Expression in cells with PMA1 under tet promoter(1)
(c) 399. Rosetta 2000: Expression in response to FR901,228(1)
(c) 430. Expression in strain PM38 (wild type), glucose versus ethanol: strain was shifted from medium containing dextrose as carbon source, ammonium sulfate as nirogen source, supplemented with leucine and uracil to same medium for 30 min, compared to a shift to a medium with synthetic ethanol instead of glucose for 30 min(1)
(c) 588. Brown enviromental changes :Nitrogen Depletion 12 h(1)
(c) 589. Brown enviromental changes :Nitrogen Depletion 1 d(1)
(c) 590. Brown enviromental changes :Nitrogen Depletion 2 d(1)
(c) 591. Brown enviromental changes :Nitrogen Depletion 3 d(1)
(c) 599. Brown enviromental changes :diauxic shift timecourse(1)
(c) 600. Brown enviromental changes :diauxic shift timecourse(1)
(c) 604. Brown enviromental changes :YPD 8 h ypd-2(1)
(c) 605. Brown enviromental changes :YPD 10 h ypd-2(1)
(c) 606. Brown enviromental changes :YPD 12 h ypd-2(1)
(c) 607. Brown enviromental changes :YPD 1 d ypd-2(1)
(c) 608. Brown enviromental changes :YPD 2 d ypd-2(1)
(c) 609. Brown enviromental changes :YPD 3 d ypd-2(1)
(c) 610. Brown enviromental changes :YPD 5 d ypd-2(1)
(c) 615. Brown enviromental changes :YPD stationary phase 1 d ypd-1(1)
(c) 616. Brown enviromental changes :YPD stationary phase 2 d ypd-1(1)
(c) 618. Brown enviromental changes :YPD stationary phase 5 d ypd-1(1)
(c) 619. Brown enviromental changes :YPD stationary phase 7 d ypd-1(1)
(c) 620. Brown enviromental changes :YPD stationary phase 13 d ypd-1(1)
(c) 621. Brown enviromental changes :YPD stationary phase 22 d ypd-1(1)
(c) 622. Brown enviromental changes :YPD stationary phase 28 d ypd-1(1)
(c) 670. Expression in response to antimycin 60min(1)
(c) (Var.) Rich Media 2% Glucose YPD-Average wt 5mM aF, 30 min.
(c) wt-gal

ADR1 -*-> JEN1

(c) 6. Expression during the cell cycle (cdc15 arrest and release)(15)
(c) 8. Expression during the cell cycle (cell size selection and release)(9)
(c) 8. Expression during the cell cycle (cell size selection and release)(12)
(c) 8. Expression during the cell cycle (cell size selection and release)(13)
(c) 8. Expression during the cell cycle (cell size selection and release)(14)
(c) 11. Expression during diauxic shift: 9h,11h,13h,15h,17h,19h,21h(7)
(c) 392. Rosetta 2000: Expression in cells with PMA1 under tet promoter(1)
(c) 399. Rosetta 2000: Expression in response to FR901,228(1)
(c) 430. Expression in strain PM38 (wild type), glucose versus ethanol: strain was shifted from medium containing dextrose as carbon source, ammonium sulfate as nirogen source, supplemented with leucine and uracil to same medium for 30 min, compared to a shift to a medium with synthetic ethanol instead of glucose for 30 min(1)
(c) 588. Brown enviromental changes :Nitrogen Depletion 12 h(1)
(c) 589. Brown enviromental changes :Nitrogen Depletion 1 d(1)
(c) 590. Brown enviromental changes :Nitrogen Depletion 2 d(1)
(c) 591. Brown enviromental changes :Nitrogen Depletion 3 d(1)
(c) 599. Brown enviromental changes :diauxic shift timecourse(1)
(c) 600. Brown enviromental changes :diauxic shift timecourse(1)
(c) 604. Brown enviromental changes :YPD 8 h ypd-2(1)
(c) 605. Brown enviromental changes :YPD 10 h ypd-2(1)
(c) 606. Brown enviromental changes :YPD 12 h ypd-2(1)
(c) 607. Brown enviromental changes :YPD 1 d ypd-2(1)
(c) 608. Brown enviromental changes :YPD 2 d ypd-2(1)
(c) 609. Brown enviromental changes :YPD 3 d ypd-2(1)

(c) 610. Brown enviromental changes :YPD 5 d ypd-2(1)
(c) 615. Brown enviromental changes :YPD stationary phase 1 d ypd-1(1)
(c) 616. Brown enviromental changes :YPD stationary phase 2 d ypd-1(1)
(c) 618. Brown enviromental changes :YPD stationary phase 5 d ypd-1(1)
(c) 619. Brown enviromental changes :YPD stationary phase 7 d ypd-1(1)
(c) 620. Brown enviromental changes :YPD stationary phase 13 d ypd-1(1)
(c) 621. Brown enviromental changes :YPD stationary phase 22 d ypd-1(1)
(c) 622. Brown enviromental changes :YPD stationary phase 28 d ypd-1(1)
(c) 670. Expression in response to antimycin 60min(1)
(c) (Var.) Rich Media 2% Glucose YPD-Average wt 5mM aF, 30 min.
(c) wt-gal

ADR1 -*-> MLS1

(c) 6. Expression during the cell cycle (cdc15 arrest and release)(15)
(c) 8. Expression during the cell cycle (cell size selection and release)(9)
(c) 8. Expression during the cell cycle (cell size selection and release)(12)
(c) 8. Expression during the cell cycle (cell size selection and release)(13)
(c) 8. Expression during the cell cycle (cell size selection and release)(14)
(c) 11. Expression during diauxic shift: 9h,11h,13h,15h,17h,19h,21h(7)
(c) 392. Rosetta 2000: Expression in cells with PMA1 under tet promoter(1)
(c) 399. Rosetta 2000: Expression in response to FR901,228(1)
(c) 430. Expression in strain PM38 (wild type), glucose versus ethanol: strain was shifted from medium containing dextrose as carbon source, ammonium sulfate as nirogen source, supplemented with leucine and uracil to same medium for 30 min, compared to a shift to a medium with synthetic ethanol instead of glucose for 30 min(1)
(c) 588. Brown enviromental changes :Nitrogen Depletion 12 h(1)
(c) 589. Brown enviromental changes :Nitrogen Depletion 1 d(1)
(c) 590. Brown enviromental changes :Nitrogen Depletion 2 d(1)
(c) 591. Brown enviromental changes :Nitrogen Depletion 3 d(1)
(c) 599. Brown enviromental changes :diauxic shift timecourse(1)
(c) 600. Brown enviromental changes :diauxic shift timecourse(1)
(c) 604. Brown enviromental changes :YPD 8 h ypd-2(1)
(c) 605. Brown enviromental changes :YPD 10 h ypd-2(1)
(c) 606. Brown enviromental changes :YPD 12 h ypd-2(1)
(c) 607. Brown enviromental changes :YPD 1 d ypd-2(1)
(c) 608. Brown enviromental changes :YPD 2 d ypd-2(1)
(c) 609. Brown enviromental changes :YPD 3 d ypd-2(1)
(c) 610. Brown enviromental changes :YPD 5 d ypd-2(1)
(c) 615. Brown enviromental changes :YPD stationary phase 1 d ypd-1(1)
(c) 616. Brown enviromental changes :YPD stationary phase 2 d ypd-1(1)
(c) 618. Brown enviromental changes :YPD stationary phase 5 d ypd-1(1)
(c) 619. Brown enviromental changes :YPD stationary phase 7 d ypd-1(1)
(c) 620. Brown enviromental changes :YPD stationary phase 13 d ypd-1(1)
(c) 621. Brown enviromental changes :YPD stationary phase 22 d ypd-1(1)
(c) 622. Brown enviromental changes :YPD stationary phase 28 d ypd-1(1)
(c) 670. Expression in response to antimycin 60min(1)
(c) (Var.) Rich Media 2% Glucose YPD-Average wt 5mM aF, 30 min.
(c) wt-gal

ADR1 -*-> PXA1

(c) 6. Expression during the cell cycle (cdc15 arrest and release)(15)
(c) 8. Expression during the cell cycle (cell size selection and release)(9)
(c) 8. Expression during the cell cycle (cell size selection and release)(12)
(c) 8. Expression during the cell cycle (cell size selection and release)(13)
(c) 8. Expression during the cell cycle (cell size selection and release)(14)
(c) 11. Expression during diauxic shift: 9h,11h,13h,15h,17h,19h,21h(7)
(c) 392. Rosetta 2000: Expression in cells with PMA1 under tet promoter(1)
(c) 399. Rosetta 2000: Expression in response to FR901,228(1)
(c) 430. Expression in strain PM38 (wild type), glucose versus ethanol: strain was shifted from medium containing dextrose as carbon source, ammonium sulfate as nirogen source, supplemented with leucine and uracil to same medium for 30 min, compared to a shift to a medium with synthetic ethanol instead of glucose for 30 min(1)
(c) 588. Brown enviromental changes :Nitrogen Depletion 12 h(1)
(c) 589. Brown enviromental changes :Nitrogen Depletion 1 d(1)
(c) 590. Brown enviromental changes :Nitrogen Depletion 2 d(1)
(c) 591. Brown enviromental changes :Nitrogen Depletion 3 d(1)
(c) 599. Brown enviromental changes :diauxic shift timecourse(1)
(c) 600. Brown enviromental changes :diauxic shift timecourse(1)
(c) 604. Brown enviromental changes :YPD 8 h ypd-2(1)
(c) 605. Brown enviromental changes :YPD 10 h ypd-2(1)
(c) 606. Brown enviromental changes :YPD 12 h ypd-2(1)
(c) 607. Brown enviromental changes :YPD 1 d ypd-2(1)
(c) 608. Brown enviromental changes :YPD 2 d ypd-2(1)
(c) 609. Brown enviromental changes :YPD 3 d ypd-2(1)
(c) 610. Brown enviromental changes :YPD 5 d ypd-2(1)
(c) 615. Brown enviromental changes :YPD stationary phase 1 d ypd-1(1)
(c) 616. Brown enviromental changes :YPD stationary phase 2 d ypd-1(1)
(c) 618. Brown enviromental changes :YPD stationary phase 5 d ypd-1(1)
(c) 619. Brown enviromental changes :YPD stationary phase 7 d ypd-1(1)
(c) 620. Brown enviromental changes :YPD stationary phase 13 d ypd-1(1)
(c) 621. Brown enviromental changes :YPD stationary phase 22 d ypd-1(1)
(c) 622. Brown enviromental changes :YPD stationary phase 28 d ypd-1(1)
(c) 670. Expression in response to antimycin 60min(1)
(c) (Var.) Rich Media 2% Glucose YPD-Average wt 5mM aF, 30 min.
(c) wt-gal

ADR1 -*-> YIL057C

(c) 6. Expression during the cell cycle (cdc15 arrest and release)(15)
(c) 8. Expression during the cell cycle (cell size selection and release)(9)
(c) 8. Expression during the cell cycle (cell size selection and release)(12)
(c) 8. Expression during the cell cycle (cell size selection and release)(13)
(c) 8. Expression during the cell cycle (cell size selection and release)(14)
(c) 11. Expression during diauxic shift: 9h,11h,13h,15h,17h,19h,21h(7)
(c) 392. Rosetta 2000: Expression in cells with PMA1 under tet promoter(1)
(c) 399. Rosetta 2000: Expression in response to FR901,228(1)
(c) 430. Expression in strain PM38 (wild type), glucose versus ethanol: strain was shifted from medium containing dextrose as carbon source, ammonium sulfate as nirogen source, supplemented with leucine and uracil to same medium for 30 min, compared to a shift to a medium with synthetic ethanol instead of glucose for 30 min(1)
(c) 588. Brown enviromental changes :Nitrogen Depletion 12 h(1)
(c) 589. Brown enviromental changes :Nitrogen Depletion 1 d(1)
(c) 590. Brown enviromental changes :Nitrogen Depletion 2 d(1)
(c) 591. Brown enviromental changes :Nitrogen Depletion 3 d(1)
(c) 599. Brown enviromental changes :diauxic shift timecourse(1)
(c) 600. Brown enviromental changes :diauxic shift timecourse(1)
(c) 604. Brown enviromental changes :YPD 8 h ypd-2(1)
(c) 605. Brown enviromental changes :YPD 10 h ypd-2(1)
(c) 606. Brown enviromental changes :YPD 12 h ypd-2(1)
(c) 607. Brown enviromental changes :YPD 1 d ypd-2(1)

(c) 608. Brown enviromental changes :YPD 2 d ypd-2(1)
(c) 609. Brown enviromental changes :YPD 3 d ypd-2(1)
(c) 610. Brown enviromental changes :YPD 5 d ypd-2(1)
(c) 615. Brown enviromental changes :YPD stationary phase 1 d ypd-1(1)
(c) 616. Brown enviromental changes :YPD stationary phase 2 d ypd-1(1)
(c) 618. Brown enviromental changes :YPD stationary phase 5 d ypd-1(1)
(c) 619. Brown enviromental changes :YPD stationary phase 7 d ypd-1(1)
(c) 620. Brown enviromental changes :YPD stationary phase 13 d ypd-1(1)
(c) 621. Brown enviromental changes :YPD stationary phase 22 d ypd-1(1)
(c) 622. Brown enviromental changes :YPD stationary phase 28 d ypd-1(1)
(c) 670. Expression in response to antimycin 60min(1)
(c) (Var.) Rich Media 2% Glucose YPD-Average wt 5mM aF, 30 min.
(c) wt-gal

ADR1 -*-> YKL187C

(c) 6. Expression during the cell cycle (cdc15 arrest and release)(15)
(c) 8. Expression during the cell cycle (cell size selection and release)(9)
(c) 8. Expression during the cell cycle (cell size selection and release)(12)
(c) 8. Expression during the cell cycle (cell size selection and release)(13)
(c) 8. Expression during the cell cycle (cell size selection and release)(14)
(c) 11. Expression during diauxic shift: 9h,11h,13h,15h,17h,19h,21h(7)
(c) 392. Rosetta 2000: Expression in cells with PMA1 under tet promoter(1)
(c) 399. Rosetta 2000: Expression in response to FR901,228(1)
(c) 430. Expression in strain PM38 (wild type), glucose versus ethanol: strain was shifted from medium containing dextrose as carbon source, ammonium sulfate as nirogen source, supplemented with leucine and uracil to same medium for 30 min, compared to a shift to a medium with synthetic ethanol instead of glucose for 30 min(1)
(c) 588. Brown enviromental changes :Nitrogen Depletion 12 h(1)
(c) 589. Brown enviromental changes :Nitrogen Depletion 1 d(1)
(c) 590. Brown enviromental changes :Nitrogen Depletion 2 d(1)
(c) 591. Brown enviromental changes :Nitrogen Depletion 3 d(1)
(c) 599. Brown enviromental changes :diauxic shift timecourse(1)
(c) 600. Brown enviromental changes :diauxic shift timecourse(1)
(c) 604. Brown enviromental changes :YPD 8 h ypd-2(1)
(c) 605. Brown enviromental changes :YPD 10 h ypd-2(1)
(c) 606. Brown enviromental changes :YPD 12 h ypd-2(1)
(c) 607. Brown enviromental changes :YPD 1 d ypd-2(1)
(c) 608. Brown enviromental changes :YPD 2 d ypd-2(1)
(c) 609. Brown enviromental changes :YPD 3 d ypd-2(1)
(c) 610. Brown enviromental changes :YPD 5 d ypd-2(1)
(c) 615. Brown enviromental changes :YPD stationary phase 1 d ypd-1(1)
(c) 616. Brown enviromental changes :YPD stationary phase 2 d ypd-1(1)
(c) 618. Brown enviromental changes :YPD stationary phase 5 d ypd-1(1)
(c) 619. Brown enviromental changes :YPD stationary phase 7 d ypd-1(1)
(c) 620. Brown enviromental changes :YPD stationary phase 13 d ypd-1(1)
(c) 621. Brown enviromental changes :YPD stationary phase 22 d ypd-1(1)
(c) 622. Brown enviromental changes :YPD stationary phase 28 d ypd-1(1)
(c) 670. Expression in response to antimycin 60min(1)
(c) (Var.) Rich Media 2% Glucose YPD-Average wt 5mM aF, 30 min.
(c) wt-gal

BAS1 -*-> ADE1

(c) 5. Expression during the cell cycle (alpha factor arrest and release)(16)
(c) 8. Expression during the cell cycle (cell size selection and release)(10)
(c) 11. Expression during diauxic shift: 9h,11h,13h,15h,17h,19h,21h(2)
(c) 11. Expression during diauxic shift: 9h,11h,13h,15h,17h,19h,21h(3)
(c) 89. Expression in response to 3-aminotriazole(1)
(c) 95. Expression in response to 50ug/mL FK506(1)
(c) 393. Rosetta 2000: Expression in cells with RHO1 under tet promoter(1)
(c) 395. Rosetta 2000: Expression in response to 2-deoxy-D-glucose(1)
(c) 402. Rosetta 2000: Expression in response to Itraconazole(1)
(c) 407. Rosetta 2000: Expression in response to Tunicamycin(1)
(c) PHO4c vs WT(1)
(c) 483. Expression in response to alkali: 10,20,40,60,80,100 min(1)
(c) 483. Expression in response to alkali: 10,20,40,60,80,100 min(2)
(c) 540. Brown enviromental changes :1 mM Menadione (120 min)redo(1)
(c) 572. Brown enviromental changes :Hypo-osmotic shock - 5 min(1)
(c) 579. Brown enviromental changes :aa starv 1 h(1)
(c) 580. Brown enviromental changes :aa starv 2 h(1)
(c) 581. Brown enviromental changes :aa starv 4 h(1)
(c) 583. Brown enviromental changes :Nitrogen Depletion 30 min.(1)
(c) 584. Brown enviromental changes :Nitrogen Depletion 1 h(1)
(c) 585. Brown enviromental changes :Nitrogen Depletion 2 h(1)
(c) 586. Brown enviromental changes :Nitrogen Depletion 4 h(1)
(c) 594. Brown enviromental changes :diauxic shift timecourse(1)
(c) 595. Brown enviromental changes :diauxic shift timecourse(1)
(c) 613. Brown enviromental changes :YPD stationary phase 8 h ypd-1(1)
(c) wt_plus_gamma_10_min

BAS1 -*-> ADE13

(c) 5. Expression during the cell cycle (alpha factor arrest and release)(16)
(c) 8. Expression during the cell cycle (cell size selection and release)(10)
(c) 11. Expression during diauxic shift: 9h,11h,13h,15h,17h,19h,21h(2)
(c) 11. Expression during diauxic shift: 9h,11h,13h,15h,17h,19h,21h(3)
(c) 89. Expression in response to 3-aminotriazole(1)
(c) 95. Expression in response to 50ug/mL FK506(1)
(c) 393. Rosetta 2000: Expression in cells with RHO1 under tet promoter(1)
(c) 395. Rosetta 2000: Expression in response to 2-deoxy-D-glucose(1)
(c) 402. Rosetta 2000: Expression in response to Itraconazole(1)
(c) 407. Rosetta 2000: Expression in response to Tunicamycin(1)
(c) PHO4c vs WT(1)
(c) 483. Expression in response to alkali: 10,20,40,60,80,100 min(1)
(c) 483. Expression in response to alkali: 10,20,40,60,80,100 min(2)
(c) 540. Brown enviromental changes :1 mM Menadione (120 min)redo(1)
(c) 572. Brown enviromental changes :Hypo-osmotic shock - 5 min(1)
(c) 579. Brown enviromental changes :aa starv 1 h(1)
(c) 580. Brown enviromental changes :aa starv 2 h(1)
(c) 581. Brown enviromental changes :aa starv 4 h(1)
(c) 583. Brown enviromental changes :Nitrogen Depletion 30 min.(1)
(c) 584. Brown enviromental changes :Nitrogen Depletion 1 h(1)
(c) 585. Brown enviromental changes :Nitrogen Depletion 2 h(1)
(c) 586. Brown enviromental changes :Nitrogen Depletion 4 h(1)
(c) 594. Brown enviromental changes :diauxic shift timecourse(1)
(c) 595. Brown enviromental changes :diauxic shift timecourse(1)
(c) 613. Brown enviromental changes :YPD stationary phase 8 h ypd-1(1)

(c) wt_plus_gamma_10_min

BAS1 -*-> ADE17

(c) 5. Expression during the cell cycle (alpha factor arrest and release)(16)
(c) 8. Expression during the cell cycle (cell size selection and release)(10)
(c) 11. Expression during diauxic shift: 9h,11h,13h,15h,17h,19h,21h(2)
(c) 11. Expression during diauxic shift: 9h,11h,13h,15h,17h,19h,21h(3)
(c) 89. Expression in response to 3-aminotriazole(1)
(c) 95. Expression in response to 50ug/mL FK506(1)
(c) 393. Rosetta 2000: Expression in cells with RHO1 under tet promoter(1)
(c) 395. Rosetta 2000: Expression in response to 2-deoxy-D-glucose(1)
(c) 402. Rosetta 2000: Expression in response to Itraconazole(1)
(c) 407. Rosetta 2000: Expression in response to Tunicamycin(1)
(c) PHO4c vs WT(1)
(c) 483. Expression in response to alkali: 10,20,40,60,80,100 min(1)
(c) 483. Expression in response to alkali: 10,20,40,60,80,100 min(2)
(c) 540. Brown enviromental changes :1 mM Menadione (120 min)redo(1)
(c) 572. Brown enviromental changes :Hypo-osmotic shock - 5 min(1)
(c) 579. Brown enviromental changes :aa starv 1 h(1)
(c) 580. Brown enviromental changes :aa starv 2 h(1)
(c) 581. Brown enviromental changes :aa starv 4 h(1)
(c) 583. Brown enviromental changes :Nitrogen Depletion 30 min.(1)
(c) 584. Brown enviromental changes :Nitrogen Depletion 1 h(1)
(c) 585. Brown enviromental changes :Nitrogen Depletion 2 h(1)
(c) 586. Brown enviromental changes :Nitrogen Depletion 4 h(1)
(c) 594. Brown enviromental changes :diauxic shift timecourse(1)
(c) 595. Brown enviromental changes :diauxic shift timecourse(1)
(c) 613. Brown enviromental changes :YPD stationary phase 8 h ypd-1(1)
(c) wt_plus_gamma_10_min

BAS1 -*-> ADE2

(c) 5. Expression during the cell cycle (alpha factor arrest and release)(16)
(c) 8. Expression during the cell cycle (cell size selection and release)(10)
(c) 11. Expression during diauxic shift: 9h,11h,13h,15h,17h,19h,21h(2)
(c) 11. Expression during diauxic shift: 9h,11h,13h,15h,17h,19h,21h(3)
(c) 89. Expression in response to 3-aminotriazole(1)
(c) 95. Expression in response to 50ug/mL FK506(1)
(c) 393. Rosetta 2000: Expression in cells with RHO1 under tet promoter(1)
(c) 395. Rosetta 2000: Expression in response to 2-deoxy-D-glucose(1)
(c) 402. Rosetta 2000: Expression in response to Itraconazole(1)
(c) 407. Rosetta 2000: Expression in response to Tunicamycin(1)
(c) PHO4c vs WT(1)
(c) 483. Expression in response to alkali: 10,20,40,60,80,100 min(1)
(c) 483. Expression in response to alkali: 10,20,40,60,80,100 min(2)
(c) 540. Brown enviromental changes :1 mM Menadione (120 min)redo(1)
(c) 572. Brown enviromental changes :Hypo-osmotic shock - 5 min(1)
(c) 579. Brown enviromental changes :aa starv 1 h(1)
(c) 580. Brown enviromental changes :aa starv 2 h(1)
(c) 581. Brown enviromental changes :aa starv 4 h(1)
(c) 583. Brown enviromental changes :Nitrogen Depletion 30 min.(1)
(c) 584. Brown enviromental changes :Nitrogen Depletion 1 h(1)
(c) 585. Brown enviromental changes :Nitrogen Depletion 2 h(1)
(c) 586. Brown enviromental changes :Nitrogen Depletion 4 h(1)
(c) 594. Brown enviromental changes :diauxic shift timecourse(1)
(c) 595. Brown enviromental changes :diauxic shift timecourse(1)
(c) 613. Brown enviromental changes :YPD stationary phase 8 h ypd-1(1)
(c) wt_plus_gamma_10_min

BAS1 -*-> ADE5,7

(c) 5. Expression during the cell cycle (alpha factor arrest and release)(16)
(c) 8. Expression during the cell cycle (cell size selection and release)(10)
(c) 11. Expression during diauxic shift: 9h,11h,13h,15h,17h,19h,21h(2)
(c) 11. Expression during diauxic shift: 9h,11h,13h,15h,17h,19h,21h(3)
(c) 89. Expression in response to 3-aminotriazole(1)
(c) 95. Expression in response to 50ug/mL FK506(1)
(c) 393. Rosetta 2000: Expression in cells with RHO1 under tet promoter(1)
(c) 395. Rosetta 2000: Expression in response to 2-deoxy-D-glucose(1)
(c) 402. Rosetta 2000: Expression in response to Itraconazole(1)
(c) 407. Rosetta 2000: Expression in response to Tunicamycin(1)
(c) PHO4c vs WT(1)
(c) 483. Expression in response to alkali: 10,20,40,60,80,100 min(1)
(c) 483. Expression in response to alkali: 10,20,40,60,80,100 min(2)
(c) 540. Brown enviromental changes :1 mM Menadione (120 min)redo(1)
(c) 572. Brown enviromental changes :Hypo-osmotic shock - 5 min(1)
(c) 579. Brown enviromental changes :aa starv 1 h(1)
(c) 580. Brown enviromental changes :aa starv 2 h(1)
(c) 581. Brown enviromental changes :aa starv 4 h(1)
(c) 583. Brown enviromental changes :Nitrogen Depletion 30 min.(1)
(c) 584. Brown enviromental changes :Nitrogen Depletion 1 h(1)
(c) 585. Brown enviromental changes :Nitrogen Depletion 2 h(1)
(c) 586. Brown enviromental changes :Nitrogen Depletion 4 h(1)
(c) 594. Brown enviromental changes :diauxic shift timecourse(1)
(c) 595. Brown enviromental changes :diauxic shift timecourse(1)
(c) 613. Brown enviromental changes :YPD stationary phase 8 h ypd-1(1)
(c) wt_plus_gamma_10_min

BAS1 -*-> BAT1

(c) 5. Expression during the cell cycle (alpha factor arrest and release)(16)
(c) 8. Expression during the cell cycle (cell size selection and release)(10)
(c) 11. Expression during diauxic shift: 9h,11h,13h,15h,17h,19h,21h(2)
(c) 11. Expression during diauxic shift: 9h,11h,13h,15h,17h,19h,21h(3)
(c) 89. Expression in response to 3-aminotriazole(1)
(c) 95. Expression in response to 50ug/mL FK506(1)
(c) 393. Rosetta 2000: Expression in cells with RHO1 under tet promoter(1)
(c) 395. Rosetta 2000: Expression in response to 2-deoxy-D-glucose(1)
(c) 402. Rosetta 2000: Expression in response to Itraconazole(1)
(c) 407. Rosetta 2000: Expression in response to Tunicamycin(1)
(c) PHO4c vs WT(1)
(c) 483. Expression in response to alkali: 10,20,40,60,80,100 min(1)
(c) 483. Expression in response to alkali: 10,20,40,60,80,100 min(2)

```
    (c) 540. Brown enviromental changes :1 mM Menadione (120 min)redo(1)
    (c) 572. Brown enviromental changes :Hypo-osmotic shock - 5 min(1)
    (c) 579. Brown enviromental changes :aa starv 1 h(1)
    (c) 580. Brown enviromental changes :aa starv 2 h(1)
    (c) 581. Brown enviromental changes :aa starv 4 h(1)
    (c) 583. Brown enviromental changes :Nitrogen Depletion 30 min.(1)
    (c) 584. Brown enviromental changes :Nitrogen Depletion 1 h(1)
    (c) 585. Brown enviromental changes :Nitrogen Depletion 2 h(1)
    (c) 586. Brown enviromental changes :Nitrogen Depletion 4 h(1)
    (c) 594. Brown enviromental changes :diauxic shift timecourse(1)
    (c) 595. Brown enviromental changes :diauxic shift timecourse(1)
    (c) 613. Brown enviromental changes :YPD stationary phase 8 h ypd-1(1)
    (c) wt_plus_gamma_10_min


BAS1 -*-> HIS1


    (c) 5. Expression during the cell cycle (alpha factor arrest and release)(16)
    (c) 8. Expression during the cell cycle (cell size selection and release)(10)
    (c) 11. Expression during diauxic shift: 9h,11h,13h,15h,17h,19h,21h(2)
    (c) 11. Expression during diauxic shift: 9h,11h,13h,15h,17h,19h,21h(3)
    (c) 89. Expression in response to 3-aminotriazole(1)
    (c) 95. Expression in response to 50ug/mL FK506(1)
    (c) 393. Rosetta 2000: Expression in cells with RHO1 under tet promoter(1)
    (c) 395. Rosetta 2000: Expression in response to 2-deoxy-D-glucose(1)
    (c) 402. Rosetta 2000: Expression in response to Itraconazole(1)
    (c) 407. Rosetta 2000: Expression in response to Tunicamycin(1)
    (c) PHO4c vs WT(1)
    (c) 483. Expression in response to alkali: 10,20,40,60,80,100 min(1)
    (c) 483. Expression in response to alkali: 10,20,40,60,80,100 min(2)
    (c) 540. Brown enviromental changes :1 mM Menadione (120 min)redo(1)
    (c) 572. Brown enviromental changes :Hypo-osmotic shock - 5 min(1)
    (c) 579. Brown enviromental changes :aa starv 1 h(1)
    (c) 580. Brown enviromental changes :aa starv 2 h(1)
    (c) 581. Brown enviromental changes :aa starv 4 h(1)
    (c) 583. Brown enviromental changes :Nitrogen Depletion 30 min.(1)
    (c) 584. Brown enviromental changes :Nitrogen Depletion 1 h(1)
    (c) 585. Brown enviromental changes :Nitrogen Depletion 2 h(1)
    (c) 586. Brown enviromental changes :Nitrogen Depletion 4 h(1)
    (c) 594. Brown enviromental changes :diauxic shift timecourse(1)
    (c) 595. Brown enviromental changes :diauxic shift timecourse(1)
    (c) 613. Brown enviromental changes :YPD stationary phase 8 h ypd-1(1)
    (c) wt_plus_gamma_10_min


BAS1 -*-> HIS4


    (c) 5. Expression during the cell cycle (alpha factor arrest and release)(16)
    (c) 8. Expression during the cell cycle (cell size selection and release)(10)
    (c) 11. Expression during diauxic shift: 9h,11h,13h,15h,17h,19h,21h(2)
    (c) 11. Expression during diauxic shift: 9h,11h,13h,15h,17h,19h,21h(3)
    (c) 89. Expression in response to 3-aminotriazole(1)
    (c) 95. Expression in response to 50ug/mL FK506(1)
    (c) 393. Rosetta 2000: Expression in cells with RHO1 under tet promoter(1)
    (c) 395. Rosetta 2000: Expression in response to 2-deoxy-D-glucose(1)
    (c) 402. Rosetta 2000: Expression in response to Itraconazole(1)
    (c) 407. Rosetta 2000: Expression in response to Tunicamycin(1)
    (c) PHO4c vs WT(1)
    (c) 483. Expression in response to alkali: 10,20,40,60,80,100 min(1)
    (c) 483. Expression in response to alkali: 10,20,40,60,80,100 min(2)
    (c) 540. Brown enviromental changes :1 mM Menadione (120 min)redo(1)
    (c) 572. Brown enviromental changes :Hypo-osmotic shock - 5 min(1)
    (c) 579. Brown enviromental changes :aa starv 1 h(1)
    (c) 580. Brown enviromental changes :aa starv 2 h(1)
    (c) 581. Brown enviromental changes :aa starv 4 h(1)
    (c) 583. Brown enviromental changes :Nitrogen Depletion 30 min.(1)
    (c) 584. Brown enviromental changes :Nitrogen Depletion 1 h(1)
    (c) 585. Brown enviromental changes :Nitrogen Depletion 2 h(1)
    (c) 586. Brown enviromental changes :Nitrogen Depletion 4 h(1)
    (c) 594. Brown enviromental changes :diauxic shift timecourse(1)
    (c) 595. Brown enviromental changes :diauxic shift timecourse(1)
    (c) 613. Brown enviromental changes :YPD stationary phase 8 h ypd-1(1)
    (c) wt_plus_gamma_10_min


BAS1 -*-> HIS5


    (c) 5. Expression during the cell cycle (alpha factor arrest and release)(16)
    (c) 8. Expression during the cell cycle (cell size selection and release)(10)
    (c) 11. Expression during diauxic shift: 9h,11h,13h,15h,17h,19h,21h(2)
    (c) 11. Expression during diauxic shift: 9h,11h,13h,15h,17h,19h,21h(3)
    (c) 89. Expression in response to 3-aminotriazole(1)
    (c) 95. Expression in response to 50ug/mL FK506(1)
    (c) 393. Rosetta 2000: Expression in cells with RHO1 under tet promoter(1)
    (c) 395. Rosetta 2000: Expression in response to 2-deoxy-D-glucose(1)
    (c) 402. Rosetta 2000: Expression in response to Itraconazole(1)
    (c) 407. Rosetta 2000: Expression in response to Tunicamycin(1)
    (c) PHO4c vs WT(1)
    (c) 483. Expression in response to alkali: 10,20,40,60,80,100 min(1)
    (c) 483. Expression in response to alkali: 10,20,40,60,80,100 min(2)
    (c) 540. Brown enviromental changes :1 mM Menadione (120 min)redo(1)
    (c) 572. Brown enviromental changes :Hypo-osmotic shock - 5 min(1)
    (c) 579. Brown enviromental changes :aa starv 1 h(1)
    (c) 580. Brown enviromental changes :aa starv 2 h(1)
    (c) 581. Brown enviromental changes :aa starv 4 h(1)
    (c) 583. Brown enviromental changes :Nitrogen Depletion 30 min.(1)
    (c) 584. Brown enviromental changes :Nitrogen Depletion 1 h(1)
    (c) 585. Brown enviromental changes :Nitrogen Depletion 2 h(1)
    (c) 586. Brown enviromental changes :Nitrogen Depletion 4 h(1)
    (c) 594. Brown enviromental changes :diauxic shift timecourse(1)
    (c) 595. Brown enviromental changes :diauxic shift timecourse(1)
    (c) 613. Brown enviromental changes :YPD stationary phase 8 h ypd-1(1)
    (c) wt_plus_gamma_10_min


BAS1 -*-> HIS7


    (c) 5. Expression during the cell cycle (alpha factor arrest and release)(16)
```

(c) 8. Expression during the cell cycle (cell size selection and release)(10)
(c) 11. Expression during diauxic shift: 9h,11h,13h,15h,17h,19h,21h(2)
(c) 11. Expression during diauxic shift: 9h,11h,13h,15h,17h,19h,21h(3)
(c) 89. Expression in response to 3-aminotriazole(1)
(c) 95. Expression in response to 50ug/mL FK506(1)
(c) 393. Rosetta 2000: Expression in cells with RHO1 under tet promoter(1)
(c) 395. Rosetta 2000: Expression in response to 2-deoxy-D-glucose(1)
(c) 402. Rosetta 2000: Expression in response to Itraconazole(1)
(c) 407. Rosetta 2000: Expression in response to Tunicamycin(1)
(c) PHO4c vs WT(1)
(c) 483. Expression in response to alkali: 10,20,40,60,80,100 min(1)
(c) 483. Expression in response to alkali: 10,20,40,60,80,100 min(2)
(c) 540. Brown enviromental changes :1 mM Menadione (120 min)redo(1)
(c) 572. Brown enviromental changes :Hypo-osmotic shock - 5 min(1)
(c) 579. Brown enviromental changes :aa starv 1 h(1)
(c) 580. Brown enviromental changes :aa starv 2 h(1)
(c) 581. Brown enviromental changes :aa starv 4 h(1)
(c) 583. Brown enviromental changes :Nitrogen Depletion 30 min.(1)
(c) 584. Brown enviromental changes :Nitrogen Depletion 1 h(1)
(c) 585. Brown enviromental changes :Nitrogen Depletion 2 h(1)
(c) 586. Brown enviromental changes :Nitrogen Depletion 4 h(1)
(c) 594. Brown enviromental changes :diauxic shift timecourse(1)
(c) 595. Brown enviromental changes :diauxic shift timecourse(1)
(c) 613. Brown enviromental changes :YPD stationary phase 8 h ypd-1(1)
(c) wt_plus_gamma_10_min

BAS1 -*-> MTD1

(c) 5. Expression during the cell cycle (alpha factor arrest and release)(16)
(c) 8. Expression during the cell cycle (cell size selection and release)(10)
(c) 11. Expression during diauxic shift: 9h,11h,13h,15h,17h,19h,21h(2)
(c) 11. Expression during diauxic shift: 9h,11h,13h,15h,17h,19h,21h(3)
(c) 89. Expression in response to 3-aminotriazole(1)
(c) 95. Expression in response to 50ug/mL FK506(1)
(c) 393. Rosetta 2000: Expression in cells with RHO1 under tet promoter(1)
(c) 395. Rosetta 2000: Expression in response to 2-deoxy-D-glucose(1)
(c) 402. Rosetta 2000: Expression in response to Itraconazole(1)
(c) 407. Rosetta 2000: Expression in response to Tunicamycin(1)
(c) PHO4c vs WT(1)
(c) 483. Expression in response to alkali: 10,20,40,60,80,100 min(1)
(c) 483. Expression in response to alkali: 10,20,40,60,80,100 min(2)
(c) 540. Brown enviromental changes :1 mM Menadione (120 min)redo(1)
(c) 572. Brown enviromental changes :Hypo-osmotic shock - 5 min(1)
(c) 579. Brown enviromental changes :aa starv 1 h(1)
(c) 580. Brown enviromental changes :aa starv 2 h(1)
(c) 581. Brown enviromental changes :aa starv 4 h(1)
(c) 583. Brown enviromental changes :Nitrogen Depletion 30 min.(1)
(c) 584. Brown enviromental changes :Nitrogen Depletion 1 h(1)
(c) 585. Brown enviromental changes :Nitrogen Depletion 2 h(1)
(c) 586. Brown enviromental changes :Nitrogen Depletion 4 h(1)
(c) 594. Brown enviromental changes :diauxic shift timecourse(1)
(c) 595. Brown enviromental changes :diauxic shift timecourse(1)
(c) 613. Brown enviromental changes :YPD stationary phase 8 h ypd-1(1)
(c) wt_plus_gamma_10_min

BAS1 -*-> SER33

(c) 5. Expression during the cell cycle (alpha factor arrest and release)(16)
(c) 8. Expression during the cell cycle (cell size selection and release)(10)
(c) 11. Expression during diauxic shift: 9h,11h,13h,15h,17h,19h,21h(2)
(c) 11. Expression during diauxic shift: 9h,11h,13h,15h,17h,19h,21h(3)
(c) 89. Expression in response to 3-aminotriazole(1)
(c) 95. Expression in response to 50ug/mL FK506(1)
(c) 393. Rosetta 2000: Expression in cells with RHO1 under tet promoter(1)
(c) 395. Rosetta 2000: Expression in response to 2-deoxy-D-glucose(1)
(c) 402. Rosetta 2000: Expression in response to Itraconazole(1)
(c) 407. Rosetta 2000: Expression in response to Tunicamycin(1)
(c) PHO4c vs WT(1)
(c) 483. Expression in response to alkali: 10,20,40,60,80,100 min(1)
(c) 483. Expression in response to alkali: 10,20,40,60,80,100 min(2)
(c) 540. Brown enviromental changes :1 mM Menadione (120 min)redo(1)
(c) 572. Brown enviromental changes :Hypo-osmotic shock - 5 min(1)
(c) 579. Brown enviromental changes :aa starv 1 h(1)
(c) 580. Brown enviromental changes :aa starv 2 h(1)
(c) 581. Brown enviromental changes :aa starv 4 h(1)
(c) 583. Brown enviromental changes :Nitrogen Depletion 30 min.(1)
(c) 584. Brown enviromental changes :Nitrogen Depletion 1 h(1)
(c) 585. Brown enviromental changes :Nitrogen Depletion 2 h(1)
(c) 586. Brown enviromental changes :Nitrogen Depletion 4 h(1)
(c) 594. Brown enviromental changes :diauxic shift timecourse(1)
(c) 595. Brown enviromental changes :diauxic shift timecourse(1)
(c) 613. Brown enviromental changes :YPD stationary phase 8 h ypd-1(1)
(c) wt_plus_gamma_10_min

BAS1 -*-> SHM2

(c) 5. Expression during the cell cycle (alpha factor arrest and release)(16)
(c) 8. Expression during the cell cycle (cell size selection and release)(10)
(c) 11. Expression during diauxic shift: 9h,11h,13h,15h,17h,19h,21h(2)
(c) 11. Expression during diauxic shift: 9h,11h,13h,15h,17h,19h,21h(3)
(c) 89. Expression in response to 3-aminotriazole(1)
(c) 95. Expression in response to 50ug/mL FK506(1)
(c) 393. Rosetta 2000: Expression in cells with RHO1 under tet promoter(1)
(c) 395. Rosetta 2000: Expression in response to 2-deoxy-D-glucose(1)
(c) 402. Rosetta 2000: Expression in response to Itraconazole(1)
(c) 407. Rosetta 2000: Expression in response to Tunicamycin(1)
(c) PHO4c vs WT(1)
(c) 483. Expression in response to alkali: 10,20,40,60,80,100 min(1)
(c) 483. Expression in response to alkali: 10,20,40,60,80,100 min(2)
(c) 540. Brown enviromental changes :1 mM Menadione (120 min)redo(1)
(c) 572. Brown enviromental changes :Hypo-osmotic shock - 5 min(1)
(c) 579. Brown enviromental changes :aa starv 1 h(1)
(c) 580. Brown enviromental changes :aa starv 2 h(1)
(c) 581. Brown enviromental changes :aa starv 4 h(1)
(c) 583. Brown enviromental changes :Nitrogen Depletion 30 min.(1)
(c) 584. Brown enviromental changes :Nitrogen Depletion 1 h(1)

```
    (c) 585. Brown enviromental changes :Nitrogen Depletion 2 h(1)
    (c) 586. Brown enviromental changes :Nitrogen Depletion 4 h(1)
    (c) 594. Brown enviromental changes :diauxic shift timecourse(1)
    (c) 595. Brown enviromental changes :diauxic shift timecourse(1)
    (c) 613. Brown enviromental changes :YPD stationary phase 8 h ypd-1(1)
    (c) wt_plus_gamma_10_min


CBF1 -*-| RIP1


    (c) 5. Expression during the cell cycle (alpha factor arrest and release)(3)
    (c) 7. Expression during the cell Cycle (cdc28)(16)
    (c) 11. Expression during diauxic shift: 9h,11h,13h,15h,17h,19h,21h(5)
    (c) 11. Expression during diauxic shift: 9h,11h,13h,15h,17h,19h,21h(6)
    (c) 11. Expression during diauxic shift: 9h,11h,13h,15h,17h,19h,21h(7)
    (c) 428. Expression in strain PM38 (wild type) in response to 30 min 50 nM treatment with rapamycin in YPD(1)
    (c) 446. Expression in response to 0.1% MMS for 10 min(1)
    (c) 447. Expression in response to 0.1% MMS for 30 min(1)
    (c) 455. Expression in response to high MNNG (27 microgram/ml) for 60 min(1)
    (c) 556. Brown enviromental changes :dtt 480 min dtt-2(1)
    (c) 597. Brown enviromental changes :diauxic shift timecourse(1)
    (c) 598. Brown enviromental changes :diauxic shift timecourse(1)
    (c) 599. Brown enviromental changes :diauxic shift timecourse(1)
    (c) 600. Brown enviromental changes :diauxic shift timecourse(1)
    (c) 602. Brown enviromental changes :YPD 4 h ypd-2(1)
    (c) 603. Brown enviromental changes :YPD 6 h ypd-2(1)
    (c) 605. Brown enviromental changes :YPD 10 h  ypd-2(1)
    (c) 613. Brown enviromental changes :YPD stationary phase 8 h ypd-1(1)
    (c) 614. Brown enviromental changes :YPD stationary phase 12 h ypd-1(1)
    (c) 615. Brown enviromental changes :YPD stationary phase 1 d ypd-1(1)
    (c) 100 microM BCS 60 min


DAL80 -*-| BAP2


    (c) 2. Cell Cycle: Expression in response to Cln3p (set 2)(1)
    (c) 11. Expression during diauxic shift: 9h,11h,13h,15h,17h,19h,21h(1)
    (c) pho80 vs WT(1)
    (c) 503. Brown enviromental changes :37C to 25C shock - 15 min(1)
    (c) 505. Brown enviromental changes :37C to 25C shock - 45 min(1)
    (c) 550. Brown enviromental changes :dtt 000 min  dtt-2(1)
    (c) 551. Brown enviromental changes :dtt 015 min dtt-2(1)
    (c) 552. Brown enviromental changes :dtt 030 min  dtt-2(1)
    (c) 574. Brown enviromental changes :Hypo-osmotic shock - 30 min(1)
    (c) 575. Brown enviromental changes :Hypo-osmotic shock - 45 min(1)
    (c) 576. Brown enviromental changes :Hypo-osmotic shock - 60 min(1)
    (c) 593. Brown enviromental changes :Diauxic Shift Timecourse(1)


DAL80 -*-| BAP3


    (c) 2. Cell Cycle: Expression in response to Cln3p (set 2)(1)
    (c) 11. Expression during diauxic shift: 9h,11h,13h,15h,17h,19h,21h(1)
    (c) pho80 vs WT(1)
    (c) 503. Brown enviromental changes :37C to 25C shock - 15 min(1)
    (c) 505. Brown enviromental changes :37C to 25C shock - 45 min(1)
    (c) 550. Brown enviromental changes :dtt 000 min  dtt-2(1)
    (c) 551. Brown enviromental changes :dtt 015 min dtt-2(1)
    (c) 552. Brown enviromental changes :dtt 030 min  dtt-2(1)
    (c) 574. Brown enviromental changes :Hypo-osmotic shock - 30 min(1)
    (c) 575. Brown enviromental changes :Hypo-osmotic shock - 45 min(1)
    (c) 576. Brown enviromental changes :Hypo-osmotic shock - 60 min(1)
    (c) 593. Brown enviromental changes :Diauxic Shift Timecourse(1)


DAL80 -*-| TAT1


    (c) 2. Cell Cycle: Expression in response to Cln3p (set 2)(1)
    (c) 11. Expression during diauxic shift: 9h,11h,13h,15h,17h,19h,21h(1)
    (c) pho80 vs WT(1)
    (c) 503. Brown enviromental changes :37C to 25C shock - 15 min(1)
    (c) 505. Brown enviromental changes :37C to 25C shock - 45 min(1)
    (c) 550. Brown enviromental changes :dtt 000 min  dtt-2(1)
    (c) 551. Brown enviromental changes :dtt 015 min dtt-2(1)
    (c) 552. Brown enviromental changes :dtt 030 min  dtt-2(1)
    (c) 574. Brown enviromental changes :Hypo-osmotic shock - 30 min(1)
    (c) 575. Brown enviromental changes :Hypo-osmotic shock - 45 min(1)
    (c) 576. Brown enviromental changes :Hypo-osmotic shock - 60 min(1)
    (c) 593. Brown enviromental changes :Diauxic Shift Timecourse(1)


DAL80 -*-| TAT2


    (c) 2. Cell Cycle: Expression in response to Cln3p (set 2)(1)
    (c) 11. Expression during diauxic shift: 9h,11h,13h,15h,17h,19h,21h(1)
    (c) pho80 vs WT(1)
    (c) 503. Brown enviromental changes :37C to 25C shock - 15 min(1)
    (c) 505. Brown enviromental changes :37C to 25C shock - 45 min(1)
    (c) 550. Brown enviromental changes :dtt 000 min  dtt-2(1)
    (c) 551. Brown enviromental changes :dtt 015 min dtt-2(1)
    (c) 552. Brown enviromental changes :dtt 030 min  dtt-2(1)
    (c) 574. Brown enviromental changes :Hypo-osmotic shock - 30 min(1)
    (c) 575. Brown enviromental changes :Hypo-osmotic shock - 45 min(1)
    (c) 576. Brown enviromental changes :Hypo-osmotic shock - 60 min(1)
    (c) 593. Brown enviromental changes :Diauxic Shift Timecourse(1)


DAL80 -*-> ERF2


    (c) 2. Cell Cycle: Expression in response to Cln3p (set 2)(1)
    (c) 11. Expression during diauxic shift: 9h,11h,13h,15h,17h,19h,21h(1)
    (c) pho80 vs WT(1)
    (c) 503. Brown enviromental changes :37C to 25C shock - 15 min(1)
    (c) 505. Brown enviromental changes :37C to 25C shock - 45 min(1)
    (c) 550. Brown enviromental changes :dtt 000 min  dtt-2(1)
    (c) 551. Brown enviromental changes :dtt 015 min dtt-2(1)
```

```
    (c) 552. Brown enviromental changes :dtt 030 min  dtt-2(1)
    (c) 574. Brown enviromental changes :Hypo-osmotic shock - 30 min(1)
    (c) 575. Brown enviromental changes :Hypo-osmotic shock - 45 min(1)
    (c) 576. Brown enviromental changes :Hypo-osmotic shock - 60 min(1)
    (c) 593. Brown enviromental changes :Diauxic Shift Timecourse(1)


DAL80 -*-> GAP1


    (c) 2. Cell Cycle: Expression in response to Cln3p (set 2)(1)
    (c) 11. Expression during diauxic shift: 9h,11h,13h,15h,17h,19h,21h(1)
    (c) pho80 vs WT(1)
    (c) 503. Brown enviromental changes :37C to 25C shock - 15 min(1)
    (c) 505. Brown enviromental changes :37C to 25C shock - 45 min(1)
    (c) 550. Brown enviromental changes :dtt 000 min  dtt-2(1)
    (c) 551. Brown enviromental changes :dtt 015 min dtt-2(1)
    (c) 552. Brown enviromental changes :dtt 030 min  dtt-2(1)
    (c) 574. Brown enviromental changes :Hypo-osmotic shock - 30 min(1)
    (c) 575. Brown enviromental changes :Hypo-osmotic shock - 45 min(1)
    (c) 576. Brown enviromental changes :Hypo-osmotic shock - 60 min(1)
    (c) 593. Brown enviromental changes :Diauxic Shift Timecourse(1)


DAL80 -*-> LEE1


    (c) 2. Cell Cycle: Expression in response to Cln3p (set 2)(1)
    (c) 11. Expression during diauxic shift: 9h,11h,13h,15h,17h,19h,21h(1)
    (c) pho80 vs WT(1)
    (c) 503. Brown enviromental changes :37C to 25C shock - 15 min(1)
    (c) 505. Brown enviromental changes :37C to 25C shock - 45 min(1)
    (c) 550. Brown enviromental changes :dtt 000 min  dtt-2(1)
    (c) 551. Brown enviromental changes :dtt 015 min dtt-2(1)
    (c) 552. Brown enviromental changes :dtt 030 min  dtt-2(1)
    (c) 574. Brown enviromental changes :Hypo-osmotic shock - 30 min(1)
    (c) 575. Brown enviromental changes :Hypo-osmotic shock - 45 min(1)
    (c) 576. Brown enviromental changes :Hypo-osmotic shock - 60 min(1)
    (c) 593. Brown enviromental changes :Diauxic Shift Timecourse(1)


DAL80 -*-> MEP1


    (c) 2. Cell Cycle: Expression in response to Cln3p (set 2)(1)
    (c) 11. Expression during diauxic shift: 9h,11h,13h,15h,17h,19h,21h(1)
    (c) pho80 vs WT(1)
    (c) 503. Brown enviromental changes :37C to 25C shock - 15 min(1)
    (c) 505. Brown enviromental changes :37C to 25C shock - 45 min(1)
    (c) 550. Brown enviromental changes :dtt 000 min  dtt-2(1)
    (c) 551. Brown enviromental changes :dtt 015 min dtt-2(1)
    (c) 552. Brown enviromental changes :dtt 030 min  dtt-2(1)
    (c) 574. Brown enviromental changes :Hypo-osmotic shock - 30 min(1)
    (c) 575. Brown enviromental changes :Hypo-osmotic shock - 45 min(1)
    (c) 576. Brown enviromental changes :Hypo-osmotic shock - 60 min(1)
    (c) 593. Brown enviromental changes :Diauxic Shift Timecourse(1)


DAL80 -*-> MEP2


    (c) 2. Cell Cycle: Expression in response to Cln3p (set 2)(1)
    (c) 11. Expression during diauxic shift: 9h,11h,13h,15h,17h,19h,21h(1)
    (c) pho80 vs WT(1)
    (c) 503. Brown enviromental changes :37C to 25C shock - 15 min(1)
    (c) 505. Brown enviromental changes :37C to 25C shock - 45 min(1)
    (c) 550. Brown enviromental changes :dtt 000 min  dtt-2(1)
    (c) 551. Brown enviromental changes :dtt 015 min dtt-2(1)
    (c) 552. Brown enviromental changes :dtt 030 min  dtt-2(1)
    (c) 574. Brown enviromental changes :Hypo-osmotic shock - 30 min(1)
    (c) 575. Brown enviromental changes :Hypo-osmotic shock - 45 min(1)
    (c) 576. Brown enviromental changes :Hypo-osmotic shock - 60 min(1)
    (c) 593. Brown enviromental changes :Diauxic Shift Timecourse(1)


DAL80 -*-> MET16


    (c) 2. Cell Cycle: Expression in response to Cln3p (set 2)(1)
    (c) 11. Expression during diauxic shift: 9h,11h,13h,15h,17h,19h,21h(1)
    (c) pho80 vs WT(1)
    (c) 503. Brown enviromental changes :37C to 25C shock - 15 min(1)
    (c) 505. Brown enviromental changes :37C to 25C shock - 45 min(1)
    (c) 550. Brown enviromental changes :dtt 000 min  dtt-2(1)
    (c) 551. Brown enviromental changes :dtt 015 min dtt-2(1)
    (c) 552. Brown enviromental changes :dtt 030 min  dtt-2(1)
    (c) 574. Brown enviromental changes :Hypo-osmotic shock - 30 min(1)
    (c) 575. Brown enviromental changes :Hypo-osmotic shock - 45 min(1)
    (c) 576. Brown enviromental changes :Hypo-osmotic shock - 60 min(1)
    (c) 593. Brown enviromental changes :Diauxic Shift Timecourse(1)


DAL80 -*-> MET2


    (c) 2. Cell Cycle: Expression in response to Cln3p (set 2)(1)
    (c) 11. Expression during diauxic shift: 9h,11h,13h,15h,17h,19h,21h(1)
    (c) pho80 vs WT(1)
    (c) 503. Brown enviromental changes :37C to 25C shock - 15 min(1)
    (c) 505. Brown enviromental changes :37C to 25C shock - 45 min(1)
    (c) 550. Brown enviromental changes :dtt 000 min  dtt-2(1)
    (c) 551. Brown enviromental changes :dtt 015 min dtt-2(1)
    (c) 552. Brown enviromental changes :dtt 030 min  dtt-2(1)
    (c) 574. Brown enviromental changes :Hypo-osmotic shock - 30 min(1)
    (c) 575. Brown enviromental changes :Hypo-osmotic shock - 45 min(1)
    (c) 576. Brown enviromental changes :Hypo-osmotic shock - 60 min(1)
    (c) 593. Brown enviromental changes :Diauxic Shift Timecourse(1)


DAL80 -*-> MET28
```

(c) 2. Cell Cycle: Expression in response to Cln3p (set 2)(1)
(c) 11. Expression during diauxic shift: 9h,11h,13h,15h,17h,19h,21h(1)
(c) pho80 vs WT(1)
(c) 503. Brown enviromental changes :37C to 25C shock - 15 min(1)
(c) 505. Brown enviromental changes :37C to 25C shock - 45 min(1)
(c) 550. Brown enviromental changes :dtt 000 min dtt-2(1)
(c) 551. Brown enviromental changes :dtt 015 min dtt-2(1)
(c) 552. Brown enviromental changes :dtt 030 min dtt-2(1)
(c) 574. Brown enviromental changes :Hypo-osmotic shock - 30 min(1)
(c) 575. Brown enviromental changes :Hypo-osmotic shock - 45 min(1)
(c) 576. Brown enviromental changes :Hypo-osmotic shock - 60 min(1)
(c) 593. Brown enviromental changes :Diauxic Shift Timecourse(1)

DAL80 -*-> OPT2

(c) 2. Cell Cycle: Expression in response to Cln3p (set 2)(1)
(c) 11. Expression during diauxic shift: 9h,11h,13h,15h,17h,19h,21h(1)
(c) pho80 vs WT(1)
(c) 503. Brown enviromental changes :37C to 25C shock - 15 min(1)
(c) 505. Brown enviromental changes :37C to 25C shock - 45 min(1)
(c) 550. Brown enviromental changes :dtt 000 min dtt-2(1)
(c) 551. Brown enviromental changes :dtt 015 min dtt-2(1)
(c) 552. Brown enviromental changes :dtt 030 min dtt-2(1)
(c) 574. Brown enviromental changes :Hypo-osmotic shock - 30 min(1)
(c) 575. Brown enviromental changes :Hypo-osmotic shock - 45 min(1)
(c) 576. Brown enviromental changes :Hypo-osmotic shock - 60 min(1)
(c) 593. Brown enviromental changes :Diauxic Shift Timecourse(1)

DAL80 -*-> YEL072W

(c) 2. Cell Cycle: Expression in response to Cln3p (set 2)(1)
(c) 11. Expression during diauxic shift: 9h,11h,13h,15h,17h,19h,21h(1)
(c) pho80 vs WT(1)
(c) 503. Brown enviromental changes :37C to 25C shock - 15 min(1)
(c) 505. Brown enviromental changes :37C to 25C shock - 45 min(1)
(c) 550. Brown enviromental changes :dtt 000 min dtt-2(1)
(c) 551. Brown enviromental changes :dtt 015 min dtt-2(1)
(c) 552. Brown enviromental changes :dtt 030 min dtt-2(1)
(c) 574. Brown enviromental changes :Hypo-osmotic shock - 30 min(1)
(c) 575. Brown enviromental changes :Hypo-osmotic shock - 45 min(1)
(c) 576. Brown enviromental changes :Hypo-osmotic shock - 60 min(1)
(c) 593. Brown enviromental changes :Diauxic Shift Timecourse(1)

DAL80 -*-> YGR125W

(c) 2. Cell Cycle: Expression in response to Cln3p (set 2)(1)
(c) 11. Expression during diauxic shift: 9h,11h,13h,15h,17h,19h,21h(1)
(c) pho80 vs WT(1)
(c) 503. Brown enviromental changes :37C to 25C shock - 15 min(1)
(c) 505. Brown enviromental changes :37C to 25C shock - 45 min(1)
(c) 550. Brown enviromental changes :dtt 000 min dtt-2(1)
(c) 551. Brown enviromental changes :dtt 015 min dtt-2(1)
(c) 552. Brown enviromental changes :dtt 030 min dtt-2(1)
(c) 574. Brown enviromental changes :Hypo-osmotic shock - 30 min(1)
(c) 575. Brown enviromental changes :Hypo-osmotic shock - 45 min(1)
(c) 576. Brown enviromental changes :Hypo-osmotic shock - 60 min(1)
(c) 593. Brown enviromental changes :Diauxic Shift Timecourse(1)

DAL80 -*-> YIR042C

(c) 2. Cell Cycle: Expression in response to Cln3p (set 2)(1)
(c) 11. Expression during diauxic shift: 9h,11h,13h,15h,17h,19h,21h(1)
(c) pho80 vs WT(1)
(c) 503. Brown enviromental changes :37C to 25C shock - 15 min(1)
(c) 505. Brown enviromental changes :37C to 25C shock - 45 min(1)
(c) 550. Brown enviromental changes :dtt 000 min dtt-2(1)
(c) 551. Brown enviromental changes :dtt 015 min dtt-2(1)
(c) 552. Brown enviromental changes :dtt 030 min dtt-2(1)
(c) 574. Brown enviromental changes :Hypo-osmotic shock - 30 min(1)
(c) 575. Brown enviromental changes :Hypo-osmotic shock - 45 min(1)
(c) 576. Brown enviromental changes :Hypo-osmotic shock - 60 min(1)
(c) 593. Brown enviromental changes :Diauxic Shift Timecourse(1)

DAL80 -*-> YKR033C

(c) 2. Cell Cycle: Expression in response to Cln3p (set 2)(1)
(c) 11. Expression during diauxic shift: 9h,11h,13h,15h,17h,19h,21h(1)
(c) pho80 vs WT(1)
(c) 503. Brown enviromental changes :37C to 25C shock - 15 min(1)
(c) 505. Brown enviromental changes :37C to 25C shock - 45 min(1)
(c) 550. Brown enviromental changes :dtt 000 min dtt-2(1)
(c) 551. Brown enviromental changes :dtt 015 min dtt-2(1)
(c) 552. Brown enviromental changes :dtt 030 min dtt-2(1)
(c) 574. Brown enviromental changes :Hypo-osmotic shock - 30 min(1)
(c) 575. Brown enviromental changes :Hypo-osmotic shock - 45 min(1)
(c) 576. Brown enviromental changes :Hypo-osmotic shock - 60 min(1)
(c) 593. Brown enviromental changes :Diauxic Shift Timecourse(1)

DAL80 -*-> YLR053C

(c) 2. Cell Cycle: Expression in response to Cln3p (set 2)(1)
(c) 11. Expression during diauxic shift: 9h,11h,13h,15h,17h,19h,21h(1)
(c) pho80 vs WT(1)
(c) 503. Brown enviromental changes :37C to 25C shock - 15 min(1)
(c) 505. Brown enviromental changes :37C to 25C shock - 45 min(1)
(c) 550. Brown enviromental changes :dtt 000 min dtt-2(1)
(c) 551. Brown enviromental changes :dtt 015 min dtt-2(1)
(c) 552. Brown enviromental changes :dtt 030 min dtt-2(1)
(c) 574. Brown enviromental changes :Hypo-osmotic shock - 30 min(1)
(c) 575. Brown enviromental changes :Hypo-osmotic shock - 45 min(1)

(c) 576. Brown enviromental changes :Hypo-osmotic shock - 60 min(1)
(c) 593. Brown enviromental changes :Diauxic Shift Timecourse(1)

DAL80 -*-> YMR088C

(c) 2. Cell Cycle: Expression in response to Cln3p (set 2)(1)
(c) 11. Expression during diauxic shift: 9h,11h,13h,15h,17h,19h,21h(1)
(c) pho80 vs WT(1)
(c) 503. Brown enviromental changes :37C to 25C shock - 15 min(1)
(c) 505. Brown enviromental changes :37C to 25C shock - 45 min(1)
(c) 550. Brown enviromental changes :dtt 000 min dtt-2(1)
(c) 551. Brown enviromental changes :dtt 015 min dtt-2(1)
(c) 552. Brown enviromental changes :dtt 030 min dtt-2(1)
(c) 574. Brown enviromental changes :Hypo-osmotic shock - 30 min(1)
(c) 575. Brown enviromental changes :Hypo-osmotic shock - 45 min(1)
(c) 576. Brown enviromental changes :Hypo-osmotic shock - 60 min(1)
(c) 593. Brown enviromental changes :Diauxic Shift Timecourse(1)

FKH2 -*-> ACE2

(c) 5. Expression during the cell cycle (alpha factor arrest and release)(1)
(c) 5. Expression during the cell cycle (alpha factor arrest and release)(3)
(c) 5. Expression during the cell cycle (alpha factor arrest and release)(4)
(c) 5. Expression during the cell cycle (alpha factor arrest and release)(5)
(c) 6. Expression during the cell cycle (cdc15 arrest and release)(3)
(c) 6. Expression during the cell cycle (cdc15 arrest and release)(11)
(c) 8. Expression during the cell cycle (cell size selection and release)(2)
(c) 8. Expression during the cell cycle (cell size selection and release)(4)
(c) 8. Expression during the cell cycle (cell size selection and release)(5)
(c) 49. Expression in response to 50 nM alpha-factor: 0,15,30,45,60,90,120 min(4)
(c) 49. Expression in response to 50 nM alpha-factor: 0,15,30,45,60,90,120 min(6)
(c) 387. Rosetta 2000: Expression in cells with ERG11 under tet promoter(1)
(c) 430. Expression in strain PM38 (wild type), glucose versus ethanol: strain was shifted from medium containing dextrose as carbon source, ammonium sulfate as nirogen source, supplemented with leucine and uracil to same medium for 30 min, compared to a shift to a medium with synthetic ethanol instead of glucose for 30 min(1)
(c) 590. Brown enviromental changes :Nitrogen Depletion 2 d(1)
(c) 592. Brown enviromental changes :Nitrogen Depletion 5 d(1)
(c) 607. Brown enviromental changes :YPD 1 d ypd-2(1)
(c) 617. Brown enviromental changes :YPD stationary phase 3 d ypd-1(1)
(c) DES460 + 0.02% MMS - 60 min

FKH2 -*-> ALK1

(c) 5. Expression during the cell cycle (alpha factor arrest and release)(1)
(c) 5. Expression during the cell cycle (alpha factor arrest and release)(3)
(c) 5. Expression during the cell cycle (alpha factor arrest and release)(4)
(c) 5. Expression during the cell cycle (alpha factor arrest and release)(5)
(c) 6. Expression during the cell cycle (cdc15 arrest and release)(3)
(c) 6. Expression during the cell cycle (cdc15 arrest and release)(11)
(c) 8. Expression during the cell cycle (cell size selection and release)(2)
(c) 8. Expression during the cell cycle (cell size selection and release)(4)
(c) 8. Expression during the cell cycle (cell size selection and release)(5)
(c) 49. Expression in response to 50 nM alpha-factor: 0,15,30,45,60,90,120 min(4)
(c) 49. Expression in response to 50 nM alpha-factor: 0,15,30,45,60,90,120 min(6)
(c) 387. Rosetta 2000: Expression in cells with ERG11 under tet promoter(1)
(c) 430. Expression in strain PM38 (wild type), glucose versus ethanol: strain was shifted from medium containing dextrose as carbon source, ammonium sulfate as nirogen source, supplemented with leucine and uracil to same medium for 30 min, compared to a shift to a medium with synthetic ethanol instead of glucose for 30 min(1)
(c) 590. Brown enviromental changes :Nitrogen Depletion 2 d(1)
(c) 592. Brown enviromental changes :Nitrogen Depletion 5 d(1)
(c) 607. Brown enviromental changes :YPD 1 d ypd-2(1)
(c) 617. Brown enviromental changes :YPD stationary phase 3 d ypd-1(1)
(c) DES460 + 0.02% MMS - 60 min

FKH2 -*-> BUD4

(c) 5. Expression during the cell cycle (alpha factor arrest and release)(1)
(c) 5. Expression during the cell cycle (alpha factor arrest and release)(3)
(c) 5. Expression during the cell cycle (alpha factor arrest and release)(4)
(c) 5. Expression during the cell cycle (alpha factor arrest and release)(5)
(c) 6. Expression during the cell cycle (cdc15 arrest and release)(3)
(c) 6. Expression during the cell cycle (cdc15 arrest and release)(11)
(c) 8. Expression during the cell cycle (cell size selection and release)(2)
(c) 8. Expression during the cell cycle (cell size selection and release)(4)
(c) 8. Expression during the cell cycle (cell size selection and release)(5)
(c) 49. Expression in response to 50 nM alpha-factor: 0,15,30,45,60,90,120 min(4)
(c) 49. Expression in response to 50 nM alpha-factor: 0,15,30,45,60,90,120 min(6)
(c) 387. Rosetta 2000: Expression in cells with ERG11 under tet promoter(1)
(c) 430. Expression in strain PM38 (wild type), glucose versus ethanol: strain was shifted from medium containing dextrose as carbon source, ammonium sulfate as nirogen source, supplemented with leucine and uracil to same medium for 30 min, compared to a shift to a medium with synthetic ethanol instead of glucose for 30 min(1)
(c) 590. Brown enviromental changes :Nitrogen Depletion 2 d(1)
(c) 592. Brown enviromental changes :Nitrogen Depletion 5 d(1)
(c) 607. Brown enviromental changes :YPD 1 d ypd-2(1)
(c) 617. Brown enviromental changes :YPD stationary phase 3 d ypd-1(1)
(c) DES460 + 0.02% MMS - 60 min

FKH2 -*-> HOF1

(c) 5. Expression during the cell cycle (alpha factor arrest and release)(1)
(c) 5. Expression during the cell cycle (alpha factor arrest and release)(3)
(c) 5. Expression during the cell cycle (alpha factor arrest and release)(4)
(c) 5. Expression during the cell cycle (alpha factor arrest and release)(5)
(c) 6. Expression during the cell cycle (cdc15 arrest and release)(3)
(c) 6. Expression during the cell cycle (cdc15 arrest and release)(11)
(c) 8. Expression during the cell cycle (cell size selection and release)(2)
(c) 8. Expression during the cell cycle (cell size selection and release)(4)
(c) 8. Expression during the cell cycle (cell size selection and release)(5)
(c) 49. Expression in response to 50 nM alpha-factor: 0,15,30,45,60,90,120 min(4)
(c) 49. Expression in response to 50 nM alpha-factor: 0,15,30,45,60,90,120 min(6)
(c) 387. Rosetta 2000: Expression in cells with ERG11 under tet promoter(1)
(c) 430. Expression in strain PM38 (wild type), glucose versus ethanol: strain was shifted from medium containing dextrose as carbon source, ammonium sulfate as nirogen source, supplemented with leucine and uracil to same medium for 30 min, compared to a shift to a medium with synthetic ethanol instead of glucose for 30 min(1)
(c) 590. Brown enviromental changes :Nitrogen Depletion 2 d(1)
(c) 592. Brown enviromental changes :Nitrogen Depletion 5 d(1)

(c) 607. Brown enviromental changes :YPD 1 d ypd-2(1)
(c) 617. Brown enviromental changes :YPD stationary phase 3 d ypd-1(1)
(c) DES460 + 0.02% MMS - 60 min

FKH2 -*-> IQG1

(c) 5. Expression during the cell cycle (alpha factor arrest and release)(1)
(c) 5. Expression during the cell cycle (alpha factor arrest and release)(3)
(c) 5. Expression during the cell cycle (alpha factor arrest and release)(4)
(c) 5. Expression during the cell cycle (alpha factor arrest and release)(5)
(c) 6. Expression during the cell cycle (cdc15 arrest and release)(3)
(c) 6. Expression during the cell cycle (cdc15 arrest and release)(11)
(c) 8. Expression during the cell cycle (cell size selection and release)(2)
(c) 8. Expression during the cell cycle (cell size selection and release)(4)
(c) 8. Expression during the cell cycle (cell size selection and release)(5)
(c) 49. Expression in response to 50 nM alpha-factor: 0,15,30,45,60,90,120 min(4)
(c) 49. Expression in response to 50 nM alpha-factor: 0,15,30,45,60,90,120 min(6)
(c) 387. Rosetta 2000: Expression in cells with ERG11 under tet promoter(1)
(c) 430. Expression in strain PM38 (wild type), glucose versus ethanol: strain was shifted from medium containing dextrose as carbon source, ammonium sulfate as nirogen source, supplemented with leucine and uracil to same medium for 30 min, compared to a shift to a medium with synthetic ethanol instead of glucose for 30 min(1)
(c) 590. Brown enviromental changes :Nitrogen Depletion 2 d(1)
(c) 592. Brown enviromental changes :Nitrogen Depletion 5 d(1)
(c) 607. Brown enviromental changes :YPD 1 d ypd-2(1)
(c) 617. Brown enviromental changes :YPD stationary phase 3 d ypd-1(1)
(c) DES460 + 0.02% MMS - 60 min

FKH2 -*-> SWI5

(c) 5. Expression during the cell cycle (alpha factor arrest and release)(1)
(c) 5. Expression during the cell cycle (alpha factor arrest and release)(3)
(c) 5. Expression during the cell cycle (alpha factor arrest and release)(4)
(c) 5. Expression during the cell cycle (alpha factor arrest and release)(5)
(c) 6. Expression during the cell cycle (cdc15 arrest and release)(3)
(c) 6. Expression during the cell cycle (cdc15 arrest and release)(11)
(c) 8. Expression during the cell cycle (cell size selection and release)(2)
(c) 8. Expression during the cell cycle (cell size selection and release)(4)
(c) 8. Expression during the cell cycle (cell size selection and release)(5)
(c) 49. Expression in response to 50 nM alpha-factor: 0,15,30,45,60,90,120 min(4)
(c) 49. Expression in response to 50 nM alpha-factor: 0,15,30,45,60,90,120 min(6)
(c) 387. Rosetta 2000: Expression in cells with ERG11 under tet promoter(1)
(c) 430. Expression in strain PM38 (wild type), glucose versus ethanol: strain was shifted from medium containing dextrose as carbon source, ammonium sulfate as nirogen source, supplemented with leucine and uracil to same medium for 30 min, compared to a shift to a medium with synthetic ethanol instead of glucose for 30 min(1)
(c) 590. Brown enviromental changes :Nitrogen Depletion 2 d(1)
(c) 592. Brown enviromental changes :Nitrogen Depletion 5 d(1)
(c) 607. Brown enviromental changes :YPD 1 d ypd-2(1)
(c) 617. Brown enviromental changes :YPD stationary phase 3 d ypd-1(1)
(c) DES460 + 0.02% MMS - 60 min

FKH2 -*-> YJL051W

(c) 5. Expression during the cell cycle (alpha factor arrest and release)(1)
(c) 5. Expression during the cell cycle (alpha factor arrest and release)(3)
(c) 5. Expression during the cell cycle (alpha factor arrest and release)(4)
(c) 5. Expression during the cell cycle (alpha factor arrest and release)(5)
(c) 6. Expression during the cell cycle (cdc15 arrest and release)(3)
(c) 6. Expression during the cell cycle (cdc15 arrest and release)(11)
(c) 8. Expression during the cell cycle (cell size selection and release)(2)
(c) 8. Expression during the cell cycle (cell size selection and release)(4)
(c) 8. Expression during the cell cycle (cell size selection and release)(5)
(c) 49. Expression in response to 50 nM alpha-factor: 0,15,30,45,60,90,120 min(4)
(c) 49. Expression in response to 50 nM alpha-factor: 0,15,30,45,60,90,120 min(6)
(c) 387. Rosetta 2000: Expression in cells with ERG11 under tet promoter(1)
(c) 430. Expression in strain PM38 (wild type), glucose versus ethanol: strain was shifted from medium containing dextrose as carbon source, ammonium sulfate as nirogen source, supplemented with leucine and uracil to same medium for 30 min, compared to a shift to a medium with synthetic ethanol instead of glucose for 30 min(1)
(c) 590. Brown enviromental changes :Nitrogen Depletion 2 d(1)
(c) 592. Brown enviromental changes :Nitrogen Depletion 5 d(1)
(c) 607. Brown enviromental changes :YPD 1 d ypd-2(1)
(c) 617. Brown enviromental changes :YPD stationary phase 3 d ypd-1(1)
(c) DES460 + 0.02% MMS - 60 min

FKH2 -*-> YLR190W

(c) 5. Expression during the cell cycle (alpha factor arrest and release)(1)
(c) 5. Expression during the cell cycle (alpha factor arrest and release)(3)
(c) 5. Expression during the cell cycle (alpha factor arrest and release)(4)
(c) 5. Expression during the cell cycle (alpha factor arrest and release)(5)
(c) 6. Expression during the cell cycle (cdc15 arrest and release)(3)
(c) 6. Expression during the cell cycle (cdc15 arrest and release)(11)
(c) 8. Expression during the cell cycle (cell size selection and release)(2)
(c) 8. Expression during the cell cycle (cell size selection and release)(4)
(c) 8. Expression during the cell cycle (cell size selection and release)(5)
(c) 49. Expression in response to 50 nM alpha-factor: 0,15,30,45,60,90,120 min(4)
(c) 49. Expression in response to 50 nM alpha-factor: 0,15,30,45,60,90,120 min(6)
(c) 387. Rosetta 2000: Expression in cells with ERG11 under tet promoter(1)
(c) 430. Expression in strain PM38 (wild type), glucose versus ethanol: strain was shifted from medium containing dextrose as carbon source, ammonium sulfate as nirogen source, supplemented with leucine and uracil to same medium for 30 min, compared to a shift to a medium with synthetic ethanol instead of glucose for 30 min(1)
(c) 590. Brown enviromental changes :Nitrogen Depletion 2 d(1)
(c) 592. Brown enviromental changes :Nitrogen Depletion 5 d(1)
(c) 607. Brown enviromental changes :YPD 1 d ypd-2(1)
(c) 617. Brown enviromental changes :YPD stationary phase 3 d ypd-1(1)
(c) DES460 + 0.02% MMS - 60 min

FKH2 -*-> YNL058C

(c) 5. Expression during the cell cycle (alpha factor arrest and release)(1)
(c) 5. Expression during the cell cycle (alpha factor arrest and release)(3)
(c) 5. Expression during the cell cycle (alpha factor arrest and release)(4)
(c) 5. Expression during the cell cycle (alpha factor arrest and release)(5)
(c) 6. Expression during the cell cycle (cdc15 arrest and release)(3)
(c) 6. Expression during the cell cycle (cdc15 arrest and release)(11)
(c) 8. Expression during the cell cycle (cell size selection and release)(2)
(c) 8. Expression during the cell cycle (cell size selection and release)(4)

(c) 8. Expression during the cell cycle (cell size selection and release)(5)
(c) 49. Expression in response to 50 nM alpha-factor: 0,15,30,45,60,90,120 min(4)
(c) 49. Expression in response to 50 nM alpha-factor: 0,15,30,45,60,90,120 min(6)
(c) 387. Rosetta 2000: Expression in cells with ERG11 under tet promoter(1)
(c) 430. Expression in strain PM38 (wild type), glucose versus ethanol: strain was shifted from medium containing dextrose as carbon source, ammonium sulfate as nirogen source, supplemented with leucine and uracil to same medium for 30 min, compared to a shift to a medium with synthetic ethanol instead of glucose for 30 min(1)
(c) 590. Brown enviromental changes :Nitrogen Depletion 2 d(1)
(c) 592. Brown enviromental changes :Nitrogen Depletion 5 d(1)
(c) 607. Brown enviromental changes :YPD 1 d ypd-2(1)
(c) 617. Brown enviromental changes :YPD stationary phase 3 d ypd-1(1)
(c) DES460 + 0.02% MMS - 60 min

FKH2 -*-> YOR315W

(c) 5. Expression during the cell cycle (alpha factor arrest and release)(1)
(c) 5. Expression during the cell cycle (alpha factor arrest and release)(3)
(c) 5. Expression during the cell cycle (alpha factor arrest and release)(4)
(c) 5. Expression during the cell cycle (alpha factor arrest and release)(5)
(c) 6. Expression during the cell cycle (cdc15 arrest and release)(3)
(c) 6. Expression during the cell cycle (cdc15 arrest and release)(11)
(c) 8. Expression during the cell cycle (cell size selection and release)(2)
(c) 8. Expression during the cell cycle (cell size selection and release)(4)
(c) 8. Expression during the cell cycle (cell size selection and release)(5)
(c) 49. Expression in response to 50 nM alpha-factor: 0,15,30,45,60,90,120 min(4)
(c) 49. Expression in response to 50 nM alpha-factor: 0,15,30,45,60,90,120 min(6)
(c) 387. Rosetta 2000: Expression in cells with ERG11 under tet promoter(1)
(c) 430. Expression in strain PM38 (wild type), glucose versus ethanol: strain was shifted from medium containing dextrose as carbon source, ammonium sulfate as nirogen source, supplemented with leucine and uracil to same medium for 30 min, compared to a shift to a medium with synthetic ethanol instead of glucose for 30 min(1)
(c) 590. Brown enviromental changes :Nitrogen Depletion 2 d(1)
(c) 592. Brown enviromental changes :Nitrogen Depletion 5 d(1)
(c) 607. Brown enviromental changes :YPD 1 d ypd-2(1)
(c) 617. Brown enviromental changes :YPD stationary phase 3 d ypd-1(1)
(c) DES460 + 0.02% MMS - 60 min

FKH2 -*-> YPL141C

(c) 5. Expression during the cell cycle (alpha factor arrest and release)(1)
(c) 5. Expression during the cell cycle (alpha factor arrest and release)(3)
(c) 5. Expression during the cell cycle (alpha factor arrest and release)(4)
(c) 5. Expression during the cell cycle (alpha factor arrest and release)(5)
(c) 6. Expression during the cell cycle (cdc15 arrest and release)(3)
(c) 6. Expression during the cell cycle (cdc15 arrest and release)(11)
(c) 8. Expression during the cell cycle (cell size selection and release)(2)
(c) 8. Expression during the cell cycle (cell size selection and release)(4)
(c) 8. Expression during the cell cycle (cell size selection and release)(5)
(c) 49. Expression in response to 50 nM alpha-factor: 0,15,30,45,60,90,120 min(4)
(c) 49. Expression in response to 50 nM alpha-factor: 0,15,30,45,60,90,120 min(6)
(c) 387. Rosetta 2000: Expression in cells with ERG11 under tet promoter(1)
(c) 430. Expression in strain PM38 (wild type), glucose versus ethanol: strain was shifted from medium containing dextrose as carbon source, ammonium sulfate as nirogen source, supplemented with leucine and uracil to same medium for 30 min, compared to a shift to a medium with synthetic ethanol instead of glucose for 30 min(1)
(c) 590. Brown enviromental changes :Nitrogen Depletion 2 d(1)
(c) 592. Brown enviromental changes :Nitrogen Depletion 5 d(1)
(c) 607. Brown enviromental changes :YPD 1 d ypd-2(1)
(c) 617. Brown enviromental changes :YPD stationary phase 3 d ypd-1(1)
(c) DES460 + 0.02% MMS - 60 min

GAT1 -*-> DAL2

(c) 428. Expression in strain PM38 (wild type) in response to 30 min 50 nM treatment with rapamycin in YPD(1)
(c) 429. Expression in strain YHE711 (wild type) in response to 30 min 50 nM treatment with rapamycin in YPD(1)
(c) 439. Expression in strain Jk9-3da (wild type) in response to 30 min 50 nM treatment with rapamycin in YPD(1)
(c) 442. Expression in strain PM38 (wild type) in response to 30 min 50 nM treatment with rapamycin in YPD(1)
(c) 479. Expression in diploid cells in response to rapamycin (100nM) for: 15min,30min,90min,120min(2)
(c) 578. Brown enviromental changes :aa starv 0.5 h(1)
(c) 579. Brown enviromental changes :aa starv 1 h(1)
(c) 580. Brown enviromental changes :aa starv 2 h(1)
(c) 581. Brown enviromental changes :aa starv 4 h(1)
(c) 582. Brown enviromental changes :aa starv 6 h(1)
(c) 583. Brown enviromental changes :Nitrogen Depletion 30 min.(1)
(c) 584. Brown enviromental changes :Nitrogen Depletion 1 h(1)
(c) 585. Brown enviromental changes :Nitrogen Depletion 2 h(1)
(c) 586. Brown enviromental changes :Nitrogen Depletion 4 h(1)
(c) 587. Brown enviromental changes :Nitrogen Depletion 8 h(1)
(c) 588. Brown enviromental changes :Nitrogen Depletion 12 h(1)
(c) 589. Brown enviromental changes :Nitrogen Depletion 1 d(1)
(c) 590. Brown enviromental changes :Nitrogen Depletion 2 d(1)
(c) 591. Brown enviromental changes :Nitrogen Depletion 3 d(1)

GAT1 -*-> DAL3

(c) 428. Expression in strain PM38 (wild type) in response to 30 min 50 nM treatment with rapamycin in YPD(1)
(c) 429. Expression in strain YHE711 (wild type) in response to 30 min 50 nM treatment with rapamycin in YPD(1)
(c) 439. Expression in strain Jk9-3da (wild type) in response to 30 min 50 nM treatment with rapamycin in YPD(1)
(c) 442. Expression in strain PM38 (wild type) in response to 30 min 50 nM treatment with rapamycin in YPD(1)
(c) 479. Expression in diploid cells in response to rapamycin (100nM) for: 15min,30min,90min,120min(2)
(c) 578. Brown enviromental changes :aa starv 0.5 h(1)
(c) 579. Brown enviromental changes :aa starv 1 h(1)
(c) 580. Brown enviromental changes :aa starv 2 h(1)
(c) 581. Brown enviromental changes :aa starv 4 h(1)
(c) 582. Brown enviromental changes :aa starv 6 h(1)
(c) 583. Brown enviromental changes :Nitrogen Depletion 30 min.(1)
(c) 584. Brown enviromental changes :Nitrogen Depletion 1 h(1)
(c) 585. Brown enviromental changes :Nitrogen Depletion 2 h(1)
(c) 586. Brown enviromental changes :Nitrogen Depletion 4 h(1)
(c) 587. Brown enviromental changes :Nitrogen Depletion 8 h(1)
(c) 588. Brown enviromental changes :Nitrogen Depletion 12 h(1)
(c) 589. Brown enviromental changes :Nitrogen Depletion 1 d(1)
(c) 590. Brown enviromental changes :Nitrogen Depletion 2 d(1)
(c) 591. Brown enviromental changes :Nitrogen Depletion 3 d(1)

GAT1 -*-> DAL5

(c) 428. Expression in strain PM38 (wild type) in response to 30 min 50 nM treatment with rapamycin in YPD(1)
(c) 429. Expression in strain YHE711 (wild type) in response to 30 min 50 nM treatment with rapamycin in YPD(1)
(c) 439. Expression in strain Jk9-3da (wild type) in response to 30 min 50 nM treatment with rapamycin in YPD(1)
(c) 442. Expression in strain PM38 (wild type) in response to 30 min 50 nM treatment with rapamycin in YPD(1)
(c) 479. Expression in diploid cells in response to rapamycin (100nM) for: 15min,30min,90min,120min(2)
(c) 578. Brown enviromental changes :aa starv 0.5 h(1)
(c) 579. Brown enviromental changes :aa starv 1 h(1)
(c) 580. Brown enviromental changes :aa starv 2 h(1)
(c) 581. Brown enviromental changes :aa starv 4 h(1)
(c) 582. Brown enviromental changes :aa starv 6 h(1)
(c) 583. Brown enviromental changes :Nitrogen Depletion 30 min.(1)
(c) 584. Brown enviromental changes :Nitrogen Depletion 1 h(1)
(c) 585. Brown enviromental changes :Nitrogen Depletion 2 h(1)
(c) 586. Brown enviromental changes :Nitrogen Depletion 4 h(1)
(c) 587. Brown enviromental changes :Nitrogen Depletion 8 h(1)
(c) 588. Brown enviromental changes :Nitrogen Depletion 12 h(1)
(c) 589. Brown enviromental changes :Nitrogen Depletion 1 d(1)
(c) 590. Brown enviromental changes :Nitrogen Depletion 2 d(1)
(c) 591. Brown enviromental changes :Nitrogen Depletion 3 d(1)

GAT1 -*-> DAL7

(c) 428. Expression in strain PM38 (wild type) in response to 30 min 50 nM treatment with rapamycin in YPD(1)
(c) 429. Expression in strain YHE711 (wild type) in response to 30 min 50 nM treatment with rapamycin in YPD(1)
(c) 439. Expression in strain Jk9-3da (wild type) in response to 30 min 50 nM treatment with rapamycin in YPD(1)
(c) 442. Expression in strain PM38 (wild type) in response to 30 min 50 nM treatment with rapamycin in YPD(1)
(c) 479. Expression in diploid cells in response to rapamycin (100nM) for: 15min,30min,90min,120min(2)
(c) 578. Brown enviromental changes :aa starv 0.5 h(1)
(c) 579. Brown enviromental changes :aa starv 1 h(1)
(c) 580. Brown enviromental changes :aa starv 2 h(1)
(c) 581. Brown enviromental changes :aa starv 4 h(1)
(c) 582. Brown enviromental changes :aa starv 6 h(1)
(c) 583. Brown enviromental changes :Nitrogen Depletion 30 min.(1)
(c) 584. Brown enviromental changes :Nitrogen Depletion 1 h(1)
(c) 585. Brown enviromental changes :Nitrogen Depletion 2 h(1)
(c) 586. Brown enviromental changes :Nitrogen Depletion 4 h(1)
(c) 587. Brown enviromental changes :Nitrogen Depletion 8 h(1)
(c) 588. Brown enviromental changes :Nitrogen Depletion 12 h(1)
(c) 589. Brown enviromental changes :Nitrogen Depletion 1 d(1)
(c) 590. Brown enviromental changes :Nitrogen Depletion 2 d(1)
(c) 591. Brown enviromental changes :Nitrogen Depletion 3 d(1)

GAT1 -*-> DUR3

(c) 428. Expression in strain PM38 (wild type) in response to 30 min 50 nM treatment with rapamycin in YPD(1)
(c) 429. Expression in strain YHE711 (wild type) in response to 30 min 50 nM treatment with rapamycin in YPD(1)
(c) 439. Expression in strain Jk9-3da (wild type) in response to 30 min 50 nM treatment with rapamycin in YPD(1)
(c) 442. Expression in strain PM38 (wild type) in response to 30 min 50 nM treatment with rapamycin in YPD(1)
(c) 479. Expression in diploid cells in response to rapamycin (100nM) for: 15min,30min,90min,120min(2)
(c) 578. Brown enviromental changes :aa starv 0.5 h(1)
(c) 579. Brown enviromental changes :aa starv 1 h(1)
(c) 580. Brown enviromental changes :aa starv 2 h(1)
(c) 581. Brown enviromental changes :aa starv 4 h(1)
(c) 582. Brown enviromental changes :aa starv 6 h(1)
(c) 583. Brown enviromental changes :Nitrogen Depletion 30 min.(1)
(c) 584. Brown enviromental changes :Nitrogen Depletion 1 h(1)
(c) 585. Brown enviromental changes :Nitrogen Depletion 2 h(1)
(c) 586. Brown enviromental changes :Nitrogen Depletion 4 h(1)
(c) 587. Brown enviromental changes :Nitrogen Depletion 8 h(1)
(c) 588. Brown enviromental changes :Nitrogen Depletion 12 h(1)
(c) 589. Brown enviromental changes :Nitrogen Depletion 1 d(1)
(c) 590. Brown enviromental changes :Nitrogen Depletion 2 d(1)
(c) 591. Brown enviromental changes :Nitrogen Depletion 3 d(1)

GAT1 -*-> MEP1

(c) 428. Expression in strain PM38 (wild type) in response to 30 min 50 nM treatment with rapamycin in YPD(1)
(c) 429. Expression in strain YHE711 (wild type) in response to 30 min 50 nM treatment with rapamycin in YPD(1)
(c) 439. Expression in strain Jk9-3da (wild type) in response to 30 min 50 nM treatment with rapamycin in YPD(1)
(c) 442. Expression in strain PM38 (wild type) in response to 30 min 50 nM treatment with rapamycin in YPD(1)
(c) 479. Expression in diploid cells in response to rapamycin (100nM) for: 15min,30min,90min,120min(2)
(c) 578. Brown enviromental changes :aa starv 0.5 h(1)
(c) 579. Brown enviromental changes :aa starv 1 h(1)
(c) 580. Brown enviromental changes :aa starv 2 h(1)
(c) 581. Brown enviromental changes :aa starv 4 h(1)
(c) 582. Brown enviromental changes :aa starv 6 h(1)
(c) 583. Brown enviromental changes :Nitrogen Depletion 30 min.(1)
(c) 584. Brown enviromental changes :Nitrogen Depletion 1 h(1)
(c) 585. Brown enviromental changes :Nitrogen Depletion 2 h(1)
(c) 586. Brown enviromental changes :Nitrogen Depletion 4 h(1)
(c) 587. Brown enviromental changes :Nitrogen Depletion 8 h(1)
(c) 588. Brown enviromental changes :Nitrogen Depletion 12 h(1)
(c) 589. Brown enviromental changes :Nitrogen Depletion 1 d(1)
(c) 590. Brown enviromental changes :Nitrogen Depletion 2 d(1)
(c) 591. Brown enviromental changes :Nitrogen Depletion 3 d(1)

GAT1 -*-> MEP2

(c) 428. Expression in strain PM38 (wild type) in response to 30 min 50 nM treatment with rapamycin in YPD(1)
(c) 429. Expression in strain YHE711 (wild type) in response to 30 min 50 nM treatment with rapamycin in YPD(1)
(c) 439. Expression in strain Jk9-3da (wild type) in response to 30 min 50 nM treatment with rapamycin in YPD(1)
(c) 442. Expression in strain PM38 (wild type) in response to 30 min 50 nM treatment with rapamycin in YPD(1)
(c) 479. Expression in diploid cells in response to rapamycin (100nM) for: 15min,30min,90min,120min(2)
(c) 578. Brown enviromental changes :aa starv 0.5 h(1)
(c) 579. Brown enviromental changes :aa starv 1 h(1)
(c) 580. Brown enviromental changes :aa starv 2 h(1)
(c) 581. Brown enviromental changes :aa starv 4 h(1)
(c) 582. Brown enviromental changes :aa starv 6 h(1)
(c) 583. Brown enviromental changes :Nitrogen Depletion 30 min.(1)
(c) 584. Brown enviromental changes :Nitrogen Depletion 1 h(1)
(c) 585. Brown enviromental changes :Nitrogen Depletion 2 h(1)
(c) 586. Brown enviromental changes :Nitrogen Depletion 4 h(1)
(c) 587. Brown enviromental changes :Nitrogen Depletion 8 h(1)
(c) 588. Brown enviromental changes :Nitrogen Depletion 12 h(1)

(c) 589. Brown enviromental changes :Nitrogen Depletion 1 d(1)
(c) 590. Brown enviromental changes :Nitrogen Depletion 2 d(1)
(c) 591. Brown enviromental changes :Nitrogen Depletion 3 d(1)

GAT1 -*-> OPT2

(c) 428. Expression in strain PM38 (wild type) in response to 30 min 50 nM treatment with rapamycin in YPD(1)
(c) 429. Expression in strain YHE711 (wild type) in response to 30 min 50 nM treatment with rapamycin in YPD(1)
(c) 439. Expression in strain Jk9-3da (wild type) in response to 30 min 50 nM treatment with rapamycin in YPD(1)
(c) 442. Expression in strain PM38 (wild type) in response to 30 min 50 nM treatment with rapamycin in YPD(1)
(c) 479. Expression in diploid cells in response to rapamycin (100nM) for: 15min,30min,90min,120min(2)
(c) 578. Brown enviromental changes :aa starv 0.5 h(1)
(c) 579. Brown enviromental changes :aa starv 1 h(1)
(c) 580. Brown enviromental changes :aa starv 2 h(1)
(c) 581. Brown enviromental changes :aa starv 4 h(1)
(c) 582. Brown enviromental changes :aa starv 6 h(1)
(c) 583. Brown enviromental changes :Nitrogen Depletion 30 min.(1)
(c) 584. Brown enviromental changes :Nitrogen Depletion 1 h(1)
(c) 585. Brown enviromental changes :Nitrogen Depletion 2 h(1)
(c) 586. Brown enviromental changes :Nitrogen Depletion 4 h(1)
(c) 587. Brown enviromental changes :Nitrogen Depletion 8 h(1)
(c) 588. Brown enviromental changes :Nitrogen Depletion 12 h(1)
(c) 589. Brown enviromental changes :Nitrogen Depletion 1 d(1)
(c) 590. Brown enviromental changes :Nitrogen Depletion 2 d(1)
(c) 591. Brown enviromental changes :Nitrogen Depletion 3 d(1)

GAT1 -*-> PUT1

(c) 428. Expression in strain PM38 (wild type) in response to 30 min 50 nM treatment with rapamycin in YPD(1)
(c) 429. Expression in strain YHE711 (wild type) in response to 30 min 50 nM treatment with rapamycin in YPD(1)
(c) 439. Expression in strain Jk9-3da (wild type) in response to 30 min 50 nM treatment with rapamycin in YPD(1)
(c) 442. Expression in strain PM38 (wild type) in response to 30 min 50 nM treatment with rapamycin in YPD(1)
(c) 479. Expression in diploid cells in response to rapamycin (100nM) for: 15min,30min,90min,120min(2)
(c) 578. Brown enviromental changes :aa starv 0.5 h(1)
(c) 579. Brown enviromental changes :aa starv 1 h(1)
(c) 580. Brown enviromental changes :aa starv 2 h(1)
(c) 581. Brown enviromental changes :aa starv 4 h(1)
(c) 582. Brown enviromental changes :aa starv 6 h(1)
(c) 583. Brown enviromental changes :Nitrogen Depletion 30 min.(1)
(c) 584. Brown enviromental changes :Nitrogen Depletion 1 h(1)
(c) 585. Brown enviromental changes :Nitrogen Depletion 2 h(1)
(c) 586. Brown enviromental changes :Nitrogen Depletion 4 h(1)
(c) 587. Brown enviromental changes :Nitrogen Depletion 8 h(1)
(c) 588. Brown enviromental changes :Nitrogen Depletion 12 h(1)
(c) 589. Brown enviromental changes :Nitrogen Depletion 1 d(1)
(c) 590. Brown enviromental changes :Nitrogen Depletion 2 d(1)
(c) 591. Brown enviromental changes :Nitrogen Depletion 3 d(1)

GAT1 -*-> YEL072W

(c) 428. Expression in strain PM38 (wild type) in response to 30 min 50 nM treatment with rapamycin in YPD(1)
(c) 429. Expression in strain YHE711 (wild type) in response to 30 min 50 nM treatment with rapamycin in YPD(1)
(c) 439. Expression in strain Jk9-3da (wild type) in response to 30 min 50 nM treatment with rapamycin in YPD(1)
(c) 442. Expression in strain PM38 (wild type) in response to 30 min 50 nM treatment with rapamycin in YPD(1)
(c) 479. Expression in diploid cells in response to rapamycin (100nM) for: 15min,30min,90min,120min(2)
(c) 578. Brown enviromental changes :aa starv 0.5 h(1)
(c) 579. Brown enviromental changes :aa starv 1 h(1)
(c) 580. Brown enviromental changes :aa starv 2 h(1)
(c) 581. Brown enviromental changes :aa starv 4 h(1)
(c) 582. Brown enviromental changes :aa starv 6 h(1)
(c) 583. Brown enviromental changes :Nitrogen Depletion 30 min.(1)
(c) 584. Brown enviromental changes :Nitrogen Depletion 1 h(1)
(c) 585. Brown enviromental changes :Nitrogen Depletion 2 h(1)
(c) 586. Brown enviromental changes :Nitrogen Depletion 4 h(1)
(c) 587. Brown enviromental changes :Nitrogen Depletion 8 h(1)
(c) 588. Brown enviromental changes :Nitrogen Depletion 12 h(1)
(c) 589. Brown enviromental changes :Nitrogen Depletion 1 d(1)
(c) 590. Brown enviromental changes :Nitrogen Depletion 2 d(1)
(c) 591. Brown enviromental changes :Nitrogen Depletion 3 d(1)

GAT1 -*-> YKR033C

(c) 428. Expression in strain PM38 (wild type) in response to 30 min 50 nM treatment with rapamycin in YPD(1)
(c) 429. Expression in strain YHE711 (wild type) in response to 30 min 50 nM treatment with rapamycin in YPD(1)
(c) 439. Expression in strain Jk9-3da (wild type) in response to 30 min 50 nM treatment with rapamycin in YPD(1)
(c) 442. Expression in strain PM38 (wild type) in response to 30 min 50 nM treatment with rapamycin in YPD(1)
(c) 479. Expression in diploid cells in response to rapamycin (100nM) for: 15min,30min,90min,120min(2)
(c) 578. Brown enviromental changes :aa starv 0.5 h(1)
(c) 579. Brown enviromental changes :aa starv 1 h(1)
(c) 580. Brown enviromental changes :aa starv 2 h(1)
(c) 581. Brown enviromental changes :aa starv 4 h(1)
(c) 582. Brown enviromental changes :aa starv 6 h(1)
(c) 583. Brown enviromental changes :Nitrogen Depletion 30 min.(1)
(c) 584. Brown enviromental changes :Nitrogen Depletion 1 h(1)
(c) 585. Brown enviromental changes :Nitrogen Depletion 2 h(1)
(c) 586. Brown enviromental changes :Nitrogen Depletion 4 h(1)
(c) 587. Brown enviromental changes :Nitrogen Depletion 8 h(1)
(c) 588. Brown enviromental changes :Nitrogen Depletion 12 h(1)
(c) 589. Brown enviromental changes :Nitrogen Depletion 1 d(1)
(c) 590. Brown enviromental changes :Nitrogen Depletion 2 d(1)
(c) 591. Brown enviromental changes :Nitrogen Depletion 3 d(1)

GAT1 -*-> YMR088C

(c) 428. Expression in strain PM38 (wild type) in response to 30 min 50 nM treatment with rapamycin in YPD(1)
(c) 429. Expression in strain YHE711 (wild type) in response to 30 min 50 nM treatment with rapamycin in YPD(1)
(c) 439. Expression in strain Jk9-3da (wild type) in response to 30 min 50 nM treatment with rapamycin in YPD(1)
(c) 442. Expression in strain PM38 (wild type) in response to 30 min 50 nM treatment with rapamycin in YPD(1)
(c) 479. Expression in diploid cells in response to rapamycin (100nM) for: 15min,30min,90min,120min(2)
(c) 578. Brown enviromental changes :aa starv 0.5 h(1)
(c) 579. Brown enviromental changes :aa starv 1 h(1)
(c) 580. Brown enviromental changes :aa starv 2 h(1)

(c) 581. Brown enviromental changes :aa starv 4 h(1)
(c) 582. Brown enviromental changes :aa starv 6 h(1)
(c) 583. Brown enviromental changes :Nitrogen Depletion 30 min.(1)
(c) 584. Brown enviromental changes :Nitrogen Depletion 1 h(1)
(c) 585. Brown enviromental changes :Nitrogen Depletion 2 h(1)
(c) 586. Brown enviromental changes :Nitrogen Depletion 4 h(1)
(c) 587. Brown enviromental changes :Nitrogen Depletion 8 h(1)
(c) 588. Brown enviromental changes :Nitrogen Depletion 12 h(1)
(c) 589. Brown enviromental changes :Nitrogen Depletion 1 d(1)
(c) 590. Brown enviromental changes :Nitrogen Depletion 2 d(1)
(c) 591. Brown enviromental changes :Nitrogen Depletion 3 d(1)

GCN4 -*-> AAT2

(c) 89. Expression in response to 3-aminotriazole(1)
(c) 95. Expression in response to 50ug/mL FK506(1)
(c) 332. Rosetta 2000: Expression in cells with CMD1 under tet promoter(1)
(c) 387. Rosetta 2000: Expression in cells with ERG11 under tet promoter(1)
(c) 395. Rosetta 2000: Expression in response to 2-deoxy-D-glucose(1)
(c) 401. Rosetta 2000: Expression in response to HU(1)
(c) 402. Rosetta 2000: Expression in response to Itraconazole(1)
(c) 403. Rosetta 2000: Expression in response to Lovastatin(1)
(c) 406. Rosetta 2000: Expression in response to Terbinafine(1)
(c) 407. Rosetta 2000: Expression in response to Tunicamycin(1)
(c) 445. Expression in response to 0.1% MMS for 60 min (average of 3 experiments)(1)
(c) 446. Expression in response to 0.1% MMS for 10 min(1)
(c) 447. Expression in response to 0.1% MMS for 30 min(1)
(c) 448. Expression in response to 0.1% MMS for 60 min(1)
(c) 449. Expression in response to 0.1% MMS for 60 min(1)
(c) 462. Expression in response to 0.05% MMS for 60 min(1)
(c) 463. Expression in response to 0.1% MMS for 60 min(1)
(c) 479. Expression in diploid cells in response to rapamycin (100nM) for: 15min,30min,90min,120min(3)
(c) 533. Brown enviromental changes :1 mM Menadione (10 min)redo(1)
(c) 579. Brown enviromental changes :aa starv 1 h(1)
(c) 580. Brown enviromental changes :aa starv 2 h(1)
(c) 581. Brown enviromental changes :aa starv 4 h(1)
(c) 584. Brown enviromental changes :Nitrogen Depletion 1 h(1)
(c) 585. Brown enviromental changes :Nitrogen Depletion 2 h(1)
(c) 586. Brown enviromental changes :Nitrogen Depletion 4 h(1)
(c) DES460 + 0.02% MMS - 15 min

GCN4 -*-> ADE12

(c) 89. Expression in response to 3-aminotriazole(1)
(c) 95. Expression in response to 50ug/mL FK506(1)
(c) 332. Rosetta 2000: Expression in cells with CMD1 under tet promoter(1)
(c) 387. Rosetta 2000: Expression in cells with ERG11 under tet promoter(1)
(c) 395. Rosetta 2000: Expression in response to 2-deoxy-D-glucose(1)
(c) 401. Rosetta 2000: Expression in response to HU(1)
(c) 402. Rosetta 2000: Expression in response to Itraconazole(1)
(c) 403. Rosetta 2000: Expression in response to Lovastatin(1)
(c) 406. Rosetta 2000: Expression in response to Terbinafine(1)
(c) 407. Rosetta 2000: Expression in response to Tunicamycin(1)
(c) 445. Expression in response to 0.1% MMS for 60 min (average of 3 experiments)(1)
(c) 446. Expression in response to 0.1% MMS for 10 min(1)
(c) 447. Expression in response to 0.1% MMS for 30 min(1)
(c) 448. Expression in response to 0.1% MMS for 60 min(1)
(c) 449. Expression in response to 0.1% MMS for 60 min(1)
(c) 462. Expression in response to 0.05% MMS for 60 min(1)
(c) 463. Expression in response to 0.1% MMS for 60 min(1)
(c) 479. Expression in diploid cells in response to rapamycin (100nM) for: 15min,30min,90min,120min(3)
(c) 533. Brown enviromental changes :1 mM Menadione (10 min)redo(1)
(c) 579. Brown enviromental changes :aa starv 1 h(1)
(c) 580. Brown enviromental changes :aa starv 2 h(1)
(c) 581. Brown enviromental changes :aa starv 4 h(1)
(c) 584. Brown enviromental changes :Nitrogen Depletion 1 h(1)
(c) 585. Brown enviromental changes :Nitrogen Depletion 2 h(1)
(c) 586. Brown enviromental changes :Nitrogen Depletion 4 h(1)
(c) DES460 + 0.02% MMS - 15 min

GCN4 -*-> ALD5

(c) 89. Expression in response to 3-aminotriazole(1)
(c) 95. Expression in response to 50ug/mL FK506(1)
(c) 332. Rosetta 2000: Expression in cells with CMD1 under tet promoter(1)
(c) 387. Rosetta 2000: Expression in cells with ERG11 under tet promoter(1)
(c) 395. Rosetta 2000: Expression in response to 2-deoxy-D-glucose(1)
(c) 401. Rosetta 2000: Expression in response to HU(1)
(c) 402. Rosetta 2000: Expression in response to Itraconazole(1)
(c) 403. Rosetta 2000: Expression in response to Lovastatin(1)
(c) 406. Rosetta 2000: Expression in response to Terbinafine(1)
(c) 407. Rosetta 2000: Expression in response to Tunicamycin(1)
(c) 445. Expression in response to 0.1% MMS for 60 min (average of 3 experiments)(1)
(c) 446. Expression in response to 0.1% MMS for 10 min(1)
(c) 447. Expression in response to 0.1% MMS for 30 min(1)
(c) 448. Expression in response to 0.1% MMS for 60 min(1)
(c) 449. Expression in response to 0.1% MMS for 60 min(1)
(c) 462. Expression in response to 0.05% MMS for 60 min(1)
(c) 463. Expression in response to 0.1% MMS for 60 min(1)
(c) 479. Expression in diploid cells in response to rapamycin (100nM) for: 15min,30min,90min,120min(3)
(c) 533. Brown enviromental changes :1 mM Menadione (10 min)redo(1)
(c) 579. Brown enviromental changes :aa starv 1 h(1)
(c) 580. Brown enviromental changes :aa starv 2 h(1)
(c) 581. Brown enviromental changes :aa starv 4 h(1)
(c) 584. Brown enviromental changes :Nitrogen Depletion 1 h(1)
(c) 585. Brown enviromental changes :Nitrogen Depletion 2 h(1)
(c) 586. Brown enviromental changes :Nitrogen Depletion 4 h(1)
(c) DES460 + 0.02% MMS - 15 min

GCN4 -*-> ATR1

(c) 89. Expression in response to 3-aminotriazole(1)
(c) 95. Expression in response to 50ug/mL FK506(1)
(c) 332. Rosetta 2000: Expression in cells with CMD1 under tet promoter(1)

(c) 387. Rosetta 2000: Expression in cells with ERG11 under tet promoter(1)
(c) 395. Rosetta 2000: Expression in response to 2-deoxy-D-glucose(1)
(c) 401. Rosetta 2000: Expression in response to HU(1)
(c) 402. Rosetta 2000: Expression in response to Itraconazole(1)
(c) 403. Rosetta 2000: Expression in response to Lovastatin(1)
(c) 406. Rosetta 2000: Expression in response to Terbinafine(1)
(c) 407. Rosetta 2000: Expression in response to Tunicamycin(1)
(c) 445. Expression in response to 0.1% MMS for 60 min (average of 3 experiments)(1)
(c) 446. Expression in response to 0.1% MMS for 10 min(1)
(c) 447. Expression in response to 0.1% MMS for 30 min(1)
(c) 448. Expression in response to 0.1% MMS for 60 min(1)
(c) 449. Expression in response to 0.1% MMS for 60 min(1)
(c) 462. Expression in response to 0.05% MMS for 60 min(1)
(c) 463. Expression in response to 0.1% MMS for 60 min(1)
(c) 479. Expression in diploid cells in response to rapamycin (100nM) for: 15min,30min,90min,120min(3)
(c) 533. Brown enviromental changes :1 mM Menadione (10 min)redo(1)
(c) 579. Brown enviromental changes :aa starv 1 h(1)
(c) 580. Brown enviromental changes :aa starv 2 h(1)
(c) 581. Brown enviromental changes :aa starv 4 h(1)
(c) 584. Brown enviromental changes :Nitrogen Depletion 1 h(1)
(c) 585. Brown enviromental changes :Nitrogen Depletion 2 h(1)
(c) 586. Brown enviromental changes :Nitrogen Depletion 4 h(1)
(c) DES460 + 0.02% MMS - 15 min

GCN4 -*-> BAT1

(c) 89. Expression in response to 3-aminotriazole(1)
(c) 95. Expression in response to 50ug/mL FK506(1)
(c) 332. Rosetta 2000: Expression in cells with CMD1 under tet promoter(1)
(c) 387. Rosetta 2000: Expression in cells with ERG11 under tet promoter(1)
(c) 395. Rosetta 2000: Expression in response to 2-deoxy-D-glucose(1)
(c) 401. Rosetta 2000: Expression in response to HU(1)
(c) 402. Rosetta 2000: Expression in response to Itraconazole(1)
(c) 403. Rosetta 2000: Expression in response to Lovastatin(1)
(c) 406. Rosetta 2000: Expression in response to Terbinafine(1)
(c) 407. Rosetta 2000: Expression in response to Tunicamycin(1)
(c) 445. Expression in response to 0.1% MMS for 60 min (average of 3 experiments)(1)
(c) 446. Expression in response to 0.1% MMS for 10 min(1)
(c) 447. Expression in response to 0.1% MMS for 30 min(1)
(c) 448. Expression in response to 0.1% MMS for 60 min(1)
(c) 449. Expression in response to 0.1% MMS for 60 min(1)
(c) 462. Expression in response to 0.05% MMS for 60 min(1)
(c) 463. Expression in response to 0.1% MMS for 60 min(1)
(c) 479. Expression in diploid cells in response to rapamycin (100nM) for: 15min,30min,90min,120min(3)
(c) 533. Brown enviromental changes :1 mM Menadione (10 min)redo(1)
(c) 579. Brown enviromental changes :aa starv 1 h(1)
(c) 580. Brown enviromental changes :aa starv 2 h(1)
(c) 581. Brown enviromental changes :aa starv 4 h(1)
(c) 584. Brown enviromental changes :Nitrogen Depletion 1 h(1)
(c) 585. Brown enviromental changes :Nitrogen Depletion 2 h(1)
(c) 586. Brown enviromental changes :Nitrogen Depletion 4 h(1)
(c) DES460 + 0.02% MMS - 15 min

GCN4 -*-> BNA1

(c) 89. Expression in response to 3-aminotriazole(1)
(c) 95. Expression in response to 50ug/mL FK506(1)
(c) 332. Rosetta 2000: Expression in cells with CMD1 under tet promoter(1)
(c) 387. Rosetta 2000: Expression in cells with ERG11 under tet promoter(1)
(c) 395. Rosetta 2000: Expression in response to 2-deoxy-D-glucose(1)
(c) 401. Rosetta 2000: Expression in response to HU(1)
(c) 402. Rosetta 2000: Expression in response to Itraconazole(1)
(c) 403. Rosetta 2000: Expression in response to Lovastatin(1)
(c) 406. Rosetta 2000: Expression in response to Terbinafine(1)
(c) 407. Rosetta 2000: Expression in response to Tunicamycin(1)
(c) 445. Expression in response to 0.1% MMS for 60 min (average of 3 experiments)(1)
(c) 446. Expression in response to 0.1% MMS for 10 min(1)
(c) 447. Expression in response to 0.1% MMS for 30 min(1)
(c) 448. Expression in response to 0.1% MMS for 60 min(1)
(c) 449. Expression in response to 0.1% MMS for 60 min(1)
(c) 462. Expression in response to 0.05% MMS for 60 min(1)
(c) 463. Expression in response to 0.1% MMS for 60 min(1)
(c) 479. Expression in diploid cells in response to rapamycin (100nM) for: 15min,30min,90min,120min(3)
(c) 533. Brown enviromental changes :1 mM Menadione (10 min)redo(1)
(c) 579. Brown enviromental changes :aa starv 1 h(1)
(c) 580. Brown enviromental changes :aa starv 2 h(1)
(c) 581. Brown enviromental changes :aa starv 4 h(1)
(c) 584. Brown enviromental changes :Nitrogen Depletion 1 h(1)
(c) 585. Brown enviromental changes :Nitrogen Depletion 2 h(1)
(c) 586. Brown enviromental changes :Nitrogen Depletion 4 h(1)
(c) DES460 + 0.02% MMS - 15 min

GCN4 -*-> CAF16

(c) 89. Expression in response to 3-aminotriazole(1)
(c) 95. Expression in response to 50ug/mL FK506(1)
(c) 332. Rosetta 2000: Expression in cells with CMD1 under tet promoter(1)
(c) 387. Rosetta 2000: Expression in cells with ERG11 under tet promoter(1)
(c) 395. Rosetta 2000: Expression in response to 2-deoxy-D-glucose(1)
(c) 401. Rosetta 2000: Expression in response to HU(1)
(c) 402. Rosetta 2000: Expression in response to Itraconazole(1)
(c) 403. Rosetta 2000: Expression in response to Lovastatin(1)
(c) 406. Rosetta 2000: Expression in response to Terbinafine(1)
(c) 407. Rosetta 2000: Expression in response to Tunicamycin(1)
(c) 445. Expression in response to 0.1% MMS for 60 min (average of 3 experiments)(1)
(c) 446. Expression in response to 0.1% MMS for 10 min(1)
(c) 447. Expression in response to 0.1% MMS for 30 min(1)
(c) 448. Expression in response to 0.1% MMS for 60 min(1)
(c) 449. Expression in response to 0.1% MMS for 60 min(1)
(c) 462. Expression in response to 0.05% MMS for 60 min(1)
(c) 463. Expression in response to 0.1% MMS for 60 min(1)
(c) 479. Expression in diploid cells in response to rapamycin (100nM) for: 15min,30min,90min,120min(3)
(c) 533. Brown enviromental changes :1 mM Menadione (10 min)redo(1)
(c) 579. Brown enviromental changes :aa starv 1 h(1)
(c) 580. Brown enviromental changes :aa starv 2 h(1)
(c) 581. Brown enviromental changes :aa starv 4 h(1)

(c) 584. Brown enviromental changes :Nitrogen Depletion 1 h(1)
(c) 585. Brown enviromental changes :Nitrogen Depletion 2 h(1)
(c) 586. Brown enviromental changes :Nitrogen Depletion 4 h(1)
(c) DES460 + 0.02% MMS - 15 min

GCN4 -*-> FOL2

(c) 89. Expression in response to 3-aminotriazole(1)
(c) 95. Expression in response to 50ug/mL FK506(1)
(c) 332. Rosetta 2000: Expression in cells with CMD1 under tet promoter(1)
(c) 387. Rosetta 2000: Expression in cells with ERG11 under tet promoter(1)
(c) 395. Rosetta 2000: Expression in response to 2-deoxy-D-glucose(1)
(c) 401. Rosetta 2000: Expression in response to HU(1)
(c) 402. Rosetta 2000: Expression in response to Itraconazole(1)
(c) 403. Rosetta 2000: Expression in response to Lovastatin(1)
(c) 406. Rosetta 2000: Expression in response to Terbinafine(1)
(c) 407. Rosetta 2000: Expression in response to Tunicamycin(1)
(c) 445. Expression in response to 0.1% MMS for 60 min (average of 3 experiments)(1)
(c) 446. Expression in response to 0.1% MMS for 10 min(1)
(c) 447. Expression in response to 0.1% MMS for 30 min(1)
(c) 448. Expression in response to 0.1% MMS for 60 min(1)
(c) 449. Expression in response to 0.1% MMS for 60 min(1)
(c) 462. Expression in response to 0.05% MMS for 60 min(1)
(c) 463. Expression in response to 0.1% MMS for 60 min(1)
(c) 479. Expression in diploid cells in response to rapamycin (100nM) for: 15min,30min,90min,120min(3)
(c) 533. Brown enviromental changes :1 mM Menadione (10 min)redo(1)
(c) 579. Brown enviromental changes :aa starv 1 h(1)
(c) 580. Brown enviromental changes :aa starv 2 h(1)
(c) 581. Brown enviromental changes :aa starv 4 h(1)
(c) 584. Brown enviromental changes :Nitrogen Depletion 1 h(1)
(c) 585. Brown enviromental changes :Nitrogen Depletion 2 h(1)
(c) 586. Brown enviromental changes :Nitrogen Depletion 4 h(1)
(c) DES460 + 0.02% MMS - 15 min

GCN4 -*-> IDP1

(c) 89. Expression in response to 3-aminotriazole(1)
(c) 95. Expression in response to 50ug/mL FK506(1)
(c) 332. Rosetta 2000: Expression in cells with CMD1 under tet promoter(1)
(c) 387. Rosetta 2000: Expression in cells with ERG11 under tet promoter(1)
(c) 395. Rosetta 2000: Expression in response to 2-deoxy-D-glucose(1)
(c) 401. Rosetta 2000: Expression in response to HU(1)
(c) 402. Rosetta 2000: Expression in response to Itraconazole(1)
(c) 403. Rosetta 2000: Expression in response to Lovastatin(1)
(c) 406. Rosetta 2000: Expression in response to Terbinafine(1)
(c) 407. Rosetta 2000: Expression in response to Tunicamycin(1)
(c) 445. Expression in response to 0.1% MMS for 60 min (average of 3 experiments)(1)
(c) 446. Expression in response to 0.1% MMS for 10 min(1)
(c) 447. Expression in response to 0.1% MMS for 30 min(1)
(c) 448. Expression in response to 0.1% MMS for 60 min(1)
(c) 449. Expression in response to 0.1% MMS for 60 min(1)
(c) 462. Expression in response to 0.05% MMS for 60 min(1)
(c) 463. Expression in response to 0.1% MMS for 60 min(1)
(c) 479. Expression in diploid cells in response to rapamycin (100nM) for: 15min,30min,90min,120min(3)
(c) 533. Brown enviromental changes :1 mM Menadione (10 min)redo(1)
(c) 579. Brown enviromental changes :aa starv 1 h(1)
(c) 580. Brown enviromental changes :aa starv 2 h(1)
(c) 581. Brown enviromental changes :aa starv 4 h(1)
(c) 584. Brown enviromental changes :Nitrogen Depletion 1 h(1)
(c) 585. Brown enviromental changes :Nitrogen Depletion 2 h(1)
(c) 586. Brown enviromental changes :Nitrogen Depletion 4 h(1)
(c) DES460 + 0.02% MMS - 15 min

GCN4 -*-> ILV3

(c) 89. Expression in response to 3-aminotriazole(1)
(c) 95. Expression in response to 50ug/mL FK506(1)
(c) 332. Rosetta 2000: Expression in cells with CMD1 under tet promoter(1)
(c) 387. Rosetta 2000: Expression in cells with ERG11 under tet promoter(1)
(c) 395. Rosetta 2000: Expression in response to 2-deoxy-D-glucose(1)
(c) 401. Rosetta 2000: Expression in response to HU(1)
(c) 402. Rosetta 2000: Expression in response to Itraconazole(1)
(c) 403. Rosetta 2000: Expression in response to Lovastatin(1)
(c) 406. Rosetta 2000: Expression in response to Terbinafine(1)
(c) 407. Rosetta 2000: Expression in response to Tunicamycin(1)
(c) 445. Expression in response to 0.1% MMS for 60 min (average of 3 experiments)(1)
(c) 446. Expression in response to 0.1% MMS for 10 min(1)
(c) 447. Expression in response to 0.1% MMS for 30 min(1)
(c) 448. Expression in response to 0.1% MMS for 60 min(1)
(c) 449. Expression in response to 0.1% MMS for 60 min(1)
(c) 462. Expression in response to 0.05% MMS for 60 min(1)
(c) 463. Expression in response to 0.1% MMS for 60 min(1)
(c) 479. Expression in diploid cells in response to rapamycin (100nM) for: 15min,30min,90min,120min(3)
(c) 533. Brown enviromental changes :1 mM Menadione (10 min)redo(1)
(c) 579. Brown enviromental changes :aa starv 1 h(1)
(c) 580. Brown enviromental changes :aa starv 2 h(1)
(c) 581. Brown enviromental changes :aa starv 4 h(1)
(c) 584. Brown enviromental changes :Nitrogen Depletion 1 h(1)
(c) 585. Brown enviromental changes :Nitrogen Depletion 2 h(1)
(c) 586. Brown enviromental changes :Nitrogen Depletion 4 h(1)
(c) DES460 + 0.02% MMS - 15 min

GCN4 -*-> MET22

(c) 89. Expression in response to 3-aminotriazole(1)
(c) 95. Expression in response to 50ug/mL FK506(1)
(c) 332. Rosetta 2000: Expression in cells with CMD1 under tet promoter(1)
(c) 387. Rosetta 2000: Expression in cells with ERG11 under tet promoter(1)
(c) 395. Rosetta 2000: Expression in response to 2-deoxy-D-glucose(1)
(c) 401. Rosetta 2000: Expression in response to HU(1)
(c) 402. Rosetta 2000: Expression in response to Itraconazole(1)
(c) 403. Rosetta 2000: Expression in response to Lovastatin(1)
(c) 406. Rosetta 2000: Expression in response to Terbinafine(1)
(c) 407. Rosetta 2000: Expression in response to Tunicamycin(1)

(c) 445. Expression in response to 0.1% MMS for 60 min (average of 3 experiments)(1)
(c) 446. Expression in response to 0.1% MMS for 10 min(1)
(c) 447. Expression in response to 0.1% MMS for 30 min(1)
(c) 448. Expression in response to 0.1% MMS for 60 min(1)
(c) 449. Expression in response to 0.1% MMS for 60 min(1)
(c) 462. Expression in response to 0.05% MMS for 60 min(1)
(c) 463. Expression in response to 0.1% MMS for 60 min(1)
(c) 479. Expression in diploid cells in response to rapamycin (100nM) for: 15min,30min,90min,120min(3)
(c) 533. Brown enviromental changes :1 mM Menadione (10 min)redo(1)
(c) 579. Brown enviromental changes :aa starv 1 h(1)
(c) 580. Brown enviromental changes :aa starv 2 h(1)
(c) 581. Brown enviromental changes :aa starv 4 h(1)
(c) 584. Brown enviromental changes :Nitrogen Depletion 1 h(1)
(c) 585. Brown enviromental changes :Nitrogen Depletion 2 h(1)
(c) 586. Brown enviromental changes :Nitrogen Depletion 4 h(1)
(c) DES460 + 0.02% MMS - 15 min

GCN4 -*-> MTD1

(c) 89. Expression in response to 3-aminotriazole(1)
(c) 95. Expression in response to 50ug/mL FK506(1)
(c) 332. Rosetta 2000: Expression in cells with CMD1 under tet promoter(1)
(c) 387. Rosetta 2000: Expression in cells with ERG11 under tet promoter(1)
(c) 395. Rosetta 2000: Expression in response to 2-deoxy-D-glucose(1)
(c) 401. Rosetta 2000: Expression in response to HU(1)
(c) 402. Rosetta 2000: Expression in response to Itraconazole(1)
(c) 403. Rosetta 2000: Expression in response to Lovastatin(1)
(c) 406. Rosetta 2000: Expression in response to Terbinafine(1)
(c) 407. Rosetta 2000: Expression in response to Tunicamycin(1)
(c) 445. Expression in response to 0.1% MMS for 60 min (average of 3 experiments)(1)
(c) 446. Expression in response to 0.1% MMS for 10 min(1)
(c) 447. Expression in response to 0.1% MMS for 30 min(1)
(c) 448. Expression in response to 0.1% MMS for 60 min(1)
(c) 449. Expression in response to 0.1% MMS for 60 min(1)
(c) 462. Expression in response to 0.05% MMS for 60 min(1)
(c) 463. Expression in response to 0.1% MMS for 60 min(1)
(c) 479. Expression in diploid cells in response to rapamycin (100nM) for: 15min,30min,90min,120min(3)
(c) 533. Brown enviromental changes :1 mM Menadione (10 min)redo(1)
(c) 579. Brown enviromental changes :aa starv 1 h(1)
(c) 580. Brown enviromental changes :aa starv 2 h(1)
(c) 581. Brown enviromental changes :aa starv 4 h(1)
(c) 584. Brown enviromental changes :Nitrogen Depletion 1 h(1)
(c) 585. Brown enviromental changes :Nitrogen Depletion 2 h(1)
(c) 586. Brown enviromental changes :Nitrogen Depletion 4 h(1)
(c) DES460 + 0.02% MMS - 15 min

GCN4 -*-> ODC2

(c) 89. Expression in response to 3-aminotriazole(1)
(c) 95. Expression in response to 50ug/mL FK506(1)
(c) 332. Rosetta 2000: Expression in cells with CMD1 under tet promoter(1)
(c) 387. Rosetta 2000: Expression in cells with ERG11 under tet promoter(1)
(c) 395. Rosetta 2000: Expression in response to 2-deoxy-D-glucose(1)
(c) 401. Rosetta 2000: Expression in response to HU(1)
(c) 402. Rosetta 2000: Expression in response to Itraconazole(1)
(c) 403. Rosetta 2000: Expression in response to Lovastatin(1)
(c) 406. Rosetta 2000: Expression in response to Terbinafine(1)
(c) 407. Rosetta 2000: Expression in response to Tunicamycin(1)
(c) 445. Expression in response to 0.1% MMS for 60 min (average of 3 experiments)(1)
(c) 446. Expression in response to 0.1% MMS for 10 min(1)
(c) 447. Expression in response to 0.1% MMS for 30 min(1)
(c) 448. Expression in response to 0.1% MMS for 60 min(1)
(c) 449. Expression in response to 0.1% MMS for 60 min(1)
(c) 462. Expression in response to 0.05% MMS for 60 min(1)
(c) 463. Expression in response to 0.1% MMS for 60 min(1)
(c) 479. Expression in diploid cells in response to rapamycin (100nM) for: 15min,30min,90min,120min(3)
(c) 533. Brown enviromental changes :1 mM Menadione (10 min)redo(1)
(c) 579. Brown enviromental changes :aa starv 1 h(1)
(c) 580. Brown enviromental changes :aa starv 2 h(1)
(c) 581. Brown enviromental changes :aa starv 4 h(1)
(c) 584. Brown enviromental changes :Nitrogen Depletion 1 h(1)
(c) 585. Brown enviromental changes :Nitrogen Depletion 2 h(1)
(c) 586. Brown enviromental changes :Nitrogen Depletion 4 h(1)
(c) DES460 + 0.02% MMS - 15 min

GCN4 -*-> ORT1

(c) 89. Expression in response to 3-aminotriazole(1)
(c) 95. Expression in response to 50ug/mL FK506(1)
(c) 332. Rosetta 2000: Expression in cells with CMD1 under tet promoter(1)
(c) 387. Rosetta 2000: Expression in cells with ERG11 under tet promoter(1)
(c) 395. Rosetta 2000: Expression in response to 2-deoxy-D-glucose(1)
(c) 401. Rosetta 2000: Expression in response to HU(1)
(c) 402. Rosetta 2000: Expression in response to Itraconazole(1)
(c) 403. Rosetta 2000: Expression in response to Lovastatin(1)
(c) 406. Rosetta 2000: Expression in response to Terbinafine(1)
(c) 407. Rosetta 2000: Expression in response to Tunicamycin(1)
(c) 445. Expression in response to 0.1% MMS for 60 min (average of 3 experiments)(1)
(c) 446. Expression in response to 0.1% MMS for 10 min(1)
(c) 447. Expression in response to 0.1% MMS for 30 min(1)
(c) 448. Expression in response to 0.1% MMS for 60 min(1)
(c) 449. Expression in response to 0.1% MMS for 60 min(1)
(c) 462. Expression in response to 0.05% MMS for 60 min(1)
(c) 463. Expression in response to 0.1% MMS for 60 min(1)
(c) 479. Expression in diploid cells in response to rapamycin (100nM) for: 15min,30min,90min,120min(3)
(c) 533. Brown enviromental changes :1 mM Menadione (10 min)redo(1)
(c) 579. Brown enviromental changes :aa starv 1 h(1)
(c) 580. Brown enviromental changes :aa starv 2 h(1)
(c) 581. Brown enviromental changes :aa starv 4 h(1)
(c) 584. Brown enviromental changes :Nitrogen Depletion 1 h(1)
(c) 585. Brown enviromental changes :Nitrogen Depletion 2 h(1)
(c) 586. Brown enviromental changes :Nitrogen Depletion 4 h(1)
(c) DES460 + 0.02% MMS - 15 min

GCN4 -*-> PET56

(c) 89. Expression in response to 3-aminotriazole(1)
(c) 95. Expression in response to 50ug/mL FK506(1)
(c) 332. Rosetta 2000: Expression in cells with CMD1 under tet promoter(1)
(c) 387. Rosetta 2000: Expression in cells with ERG11 under tet promoter(1)
(c) 395. Rosetta 2000: Expression in response to 2-deoxy-D-glucose(1)
(c) 401. Rosetta 2000: Expression in response to HU(1)
(c) 402. Rosetta 2000: Expression in response to Itraconazole(1)
(c) 403. Rosetta 2000: Expression in response to Lovastatin(1)
(c) 406. Rosetta 2000: Expression in response to Terbinafine(1)
(c) 407. Rosetta 2000: Expression in response to Tunicamycin(1)
(c) 445. Expression in response to 0.1% MMS for 60 min (average of 3 experiments)(1)
(c) 446. Expression in response to 0.1% MMS for 10 min(1)
(c) 447. Expression in response to 0.1% MMS for 30 min(1)
(c) 448. Expression in response to 0.1% MMS for 60 min(1)
(c) 449. Expression in response to 0.1% MMS for 60 min(1)
(c) 462. Expression in response to 0.05% MMS for 60 min(1)
(c) 463. Expression in response to 0.1% MMS for 60 min(1)
(c) 479. Expression in diploid cells in response to rapamycin (100nM) for: 15min,30min,90min,120min(3)
(c) 533. Brown enviromental changes :1 mM Menadione (10 min)redo(1)
(c) 579. Brown enviromental changes :aa starv 1 h(1)
(c) 580. Brown enviromental changes :aa starv 2 h(1)
(c) 581. Brown enviromental changes :aa starv 4 h(1)
(c) 584. Brown enviromental changes :Nitrogen Depletion 1 h(1)
(c) 585. Brown enviromental changes :Nitrogen Depletion 2 h(1)
(c) 586. Brown enviromental changes :Nitrogen Depletion 4 h(1)
(c) DES460 + 0.02% MMS - 15 min

GCN4 -*-> SRY1

(c) 89. Expression in response to 3-aminotriazole(1)
(c) 95. Expression in response to 50ug/mL FK506(1)
(c) 332. Rosetta 2000: Expression in cells with CMD1 under tet promoter(1)
(c) 387. Rosetta 2000: Expression in cells with ERG11 under tet promoter(1)
(c) 395. Rosetta 2000: Expression in response to 2-deoxy-D-glucose(1)
(c) 401. Rosetta 2000: Expression in response to HU(1)
(c) 402. Rosetta 2000: Expression in response to Itraconazole(1)
(c) 403. Rosetta 2000: Expression in response to Lovastatin(1)
(c) 406. Rosetta 2000: Expression in response to Terbinafine(1)
(c) 407. Rosetta 2000: Expression in response to Tunicamycin(1)
(c) 445. Expression in response to 0.1% MMS for 60 min (average of 3 experiments)(1)
(c) 446. Expression in response to 0.1% MMS for 10 min(1)
(c) 447. Expression in response to 0.1% MMS for 30 min(1)
(c) 448. Expression in response to 0.1% MMS for 60 min(1)
(c) 449. Expression in response to 0.1% MMS for 60 min(1)
(c) 462. Expression in response to 0.05% MMS for 60 min(1)
(c) 463. Expression in response to 0.1% MMS for 60 min(1)
(c) 479. Expression in diploid cells in response to rapamycin (100nM) for: 15min,30min,90min,120min(3)
(c) 533. Brown enviromental changes :1 mM Menadione (10 min)redo(1)
(c) 579. Brown enviromental changes :aa starv 1 h(1)
(c) 580. Brown enviromental changes :aa starv 2 h(1)
(c) 581. Brown enviromental changes :aa starv 4 h(1)
(c) 584. Brown enviromental changes :Nitrogen Depletion 1 h(1)
(c) 585. Brown enviromental changes :Nitrogen Depletion 2 h(1)
(c) 586. Brown enviromental changes :Nitrogen Depletion 4 h(1)
(c) DES460 + 0.02% MMS - 15 min

GCN4 -*-> TMT1

(c) 89. Expression in response to 3-aminotriazole(1)
(c) 95. Expression in response to 50ug/mL FK506(1)
(c) 332. Rosetta 2000: Expression in cells with CMD1 under tet promoter(1)
(c) 387. Rosetta 2000: Expression in cells with ERG11 under tet promoter(1)
(c) 395. Rosetta 2000: Expression in response to 2-deoxy-D-glucose(1)
(c) 401. Rosetta 2000: Expression in response to HU(1)
(c) 402. Rosetta 2000: Expression in response to Itraconazole(1)
(c) 403. Rosetta 2000: Expression in response to Lovastatin(1)
(c) 406. Rosetta 2000: Expression in response to Terbinafine(1)
(c) 407. Rosetta 2000: Expression in response to Tunicamycin(1)
(c) 445. Expression in response to 0.1% MMS for 60 min (average of 3 experiments)(1)
(c) 446. Expression in response to 0.1% MMS for 10 min(1)
(c) 447. Expression in response to 0.1% MMS for 30 min(1)
(c) 448. Expression in response to 0.1% MMS for 60 min(1)
(c) 449. Expression in response to 0.1% MMS for 60 min(1)
(c) 462. Expression in response to 0.05% MMS for 60 min(1)
(c) 463. Expression in response to 0.1% MMS for 60 min(1)
(c) 479. Expression in diploid cells in response to rapamycin (100nM) for: 15min,30min,90min,120min(3)
(c) 533. Brown enviromental changes :1 mM Menadione (10 min)redo(1)
(c) 579. Brown enviromental changes :aa starv 1 h(1)
(c) 580. Brown enviromental changes :aa starv 2 h(1)
(c) 581. Brown enviromental changes :aa starv 4 h(1)
(c) 584. Brown enviromental changes :Nitrogen Depletion 1 h(1)
(c) 585. Brown enviromental changes :Nitrogen Depletion 2 h(1)
(c) 586. Brown enviromental changes :Nitrogen Depletion 4 h(1)
(c) DES460 + 0.02% MMS - 15 min

GCN4 -*-> UGA3

(c) 89. Expression in response to 3-aminotriazole(1)
(c) 95. Expression in response to 50ug/mL FK506(1)
(c) 332. Rosetta 2000: Expression in cells with CMD1 under tet promoter(1)
(c) 387. Rosetta 2000: Expression in cells with ERG11 under tet promoter(1)
(c) 395. Rosetta 2000: Expression in response to 2-deoxy-D-glucose(1)
(c) 401. Rosetta 2000: Expression in response to HU(1)
(c) 402. Rosetta 2000: Expression in response to Itraconazole(1)
(c) 403. Rosetta 2000: Expression in response to Lovastatin(1)
(c) 406. Rosetta 2000: Expression in response to Terbinafine(1)
(c) 407. Rosetta 2000: Expression in response to Tunicamycin(1)
(c) 445. Expression in response to 0.1% MMS for 60 min (average of 3 experiments)(1)
(c) 446. Expression in response to 0.1% MMS for 10 min(1)
(c) 447. Expression in response to 0.1% MMS for 30 min(1)
(c) 448. Expression in response to 0.1% MMS for 60 min(1)
(c) 449. Expression in response to 0.1% MMS for 60 min(1)
(c) 462. Expression in response to 0.05% MMS for 60 min(1)
(c) 463. Expression in response to 0.1% MMS for 60 min(1)

```
    (c) 479. Expression in diploid cells in response to rapamycin (100nM) for: 15min,30min,90min,120min(3)
    (c) 533. Brown enviromental changes :1 mM Menadione (10 min)redo(1)
    (c) 579. Brown enviromental changes :aa starv 1 h(1)
    (c) 580. Brown enviromental changes :aa starv 2 h(1)
    (c) 581. Brown enviromental changes :aa starv 4 h(1)
    (c) 584. Brown enviromental changes :Nitrogen Depletion 1 h(1)
    (c) 585. Brown enviromental changes :Nitrogen Depletion 2 h(1)
    (c) 586. Brown enviromental changes :Nitrogen Depletion 4 h(1)
    (c) DES460 + 0.02% MMS - 15 min


GCN4 -*-> YHM1


    (c) 89. Expression in response to 3-aminotriazole(1)
    (c) 95. Expression in response to 50ug/mL FK506(1)
    (c) 332. Rosetta 2000: Expression in cells with CMD1 under tet promoter(1)
    (c) 387. Rosetta 2000: Expression in cells with ERG11 under tet promoter(1)
    (c) 395. Rosetta 2000: Expression in response to 2-deoxy-D-glucose(1)
    (c) 401. Rosetta 2000: Expression in response to HU(1)
    (c) 402. Rosetta 2000: Expression in response to Itraconazole(1)
    (c) 403. Rosetta 2000: Expression in response to Lovastatin(1)
    (c) 406. Rosetta 2000: Expression in response to Terbinafine(1)
    (c) 407. Rosetta 2000: Expression in response to Tunicamycin(1)
    (c) 445. Expression in response to 0.1% MMS for 60 min (average of 3 experiments)(1)
    (c) 446. Expression in response to 0.1% MMS for 10 min(1)
    (c) 447. Expression in response to 0.1% MMS for 30 min(1)
    (c) 448. Expression in response to 0.1% MMS for 60 min(1)
    (c) 449. Expression in response to 0.1% MMS for 60 min(1)
    (c) 462. Expression in response to 0.05% MMS for 60 min(1)
    (c) 463. Expression in response to 0.1% MMS for 60 min(1)
    (c) 479. Expression in diploid cells in response to rapamycin (100nM) for: 15min,30min,90min,120min(3)
    (c) 533. Brown enviromental changes :1 mM Menadione (10 min)redo(1)
    (c) 579. Brown enviromental changes :aa starv 1 h(1)
    (c) 580. Brown enviromental changes :aa starv 2 h(1)
    (c) 581. Brown enviromental changes :aa starv 4 h(1)
    (c) 584. Brown enviromental changes :Nitrogen Depletion 1 h(1)
    (c) 585. Brown enviromental changes :Nitrogen Depletion 2 h(1)
    (c) 586. Brown enviromental changes :Nitrogen Depletion 4 h(1)
    (c) DES460 + 0.02% MMS - 15 min


GCN4 -*-> YHR162W


    (c) 89. Expression in response to 3-aminotriazole(1)
    (c) 95. Expression in response to 50ug/mL FK506(1)
    (c) 332. Rosetta 2000: Expression in cells with CMD1 under tet promoter(1)
    (c) 387. Rosetta 2000: Expression in cells with ERG11 under tet promoter(1)
    (c) 395. Rosetta 2000: Expression in response to 2-deoxy-D-glucose(1)
    (c) 401. Rosetta 2000: Expression in response to HU(1)
    (c) 402. Rosetta 2000: Expression in response to Itraconazole(1)
    (c) 403. Rosetta 2000: Expression in response to Lovastatin(1)
    (c) 406. Rosetta 2000: Expression in response to Terbinafine(1)
    (c) 407. Rosetta 2000: Expression in response to Tunicamycin(1)
    (c) 445. Expression in response to 0.1% MMS for 60 min (average of 3 experiments)(1)
    (c) 446. Expression in response to 0.1% MMS for 10 min(1)
    (c) 447. Expression in response to 0.1% MMS for 30 min(1)
    (c) 448. Expression in response to 0.1% MMS for 60 min(1)
    (c) 449. Expression in response to 0.1% MMS for 60 min(1)
    (c) 462. Expression in response to 0.05% MMS for 60 min(1)
    (c) 463. Expression in response to 0.1% MMS for 60 min(1)
    (c) 479. Expression in diploid cells in response to rapamycin (100nM) for: 15min,30min,90min,120min(3)
    (c) 533. Brown enviromental changes :1 mM Menadione (10 min)redo(1)
    (c) 579. Brown enviromental changes :aa starv 1 h(1)
    (c) 580. Brown enviromental changes :aa starv 2 h(1)
    (c) 581. Brown enviromental changes :aa starv 4 h(1)
    (c) 584. Brown enviromental changes :Nitrogen Depletion 1 h(1)
    (c) 585. Brown enviromental changes :Nitrogen Depletion 2 h(1)
    (c) 586. Brown enviromental changes :Nitrogen Depletion 4 h(1)
    (c) DES460 + 0.02% MMS - 15 min


GCN4 -*-> YIL165C


    (c) 89. Expression in response to 3-aminotriazole(1)
    (c) 95. Expression in response to 50ug/mL FK506(1)
    (c) 332. Rosetta 2000: Expression in cells with CMD1 under tet promoter(1)
    (c) 387. Rosetta 2000: Expression in cells with ERG11 under tet promoter(1)
    (c) 395. Rosetta 2000: Expression in response to 2-deoxy-D-glucose(1)
    (c) 401. Rosetta 2000: Expression in response to HU(1)
    (c) 402. Rosetta 2000: Expression in response to Itraconazole(1)
    (c) 403. Rosetta 2000: Expression in response to Lovastatin(1)
    (c) 406. Rosetta 2000: Expression in response to Terbinafine(1)
    (c) 407. Rosetta 2000: Expression in response to Tunicamycin(1)
    (c) 445. Expression in response to 0.1% MMS for 60 min (average of 3 experiments)(1)
    (c) 446. Expression in response to 0.1% MMS for 10 min(1)
    (c) 447. Expression in response to 0.1% MMS for 30 min(1)
    (c) 448. Expression in response to 0.1% MMS for 60 min(1)
    (c) 449. Expression in response to 0.1% MMS for 60 min(1)
    (c) 462. Expression in response to 0.05% MMS for 60 min(1)
    (c) 463. Expression in response to 0.1% MMS for 60 min(1)
    (c) 479. Expression in diploid cells in response to rapamycin (100nM) for: 15min,30min,90min,120min(3)
    (c) 533. Brown enviromental changes :1 mM Menadione (10 min)redo(1)
    (c) 579. Brown enviromental changes :aa starv 1 h(1)
    (c) 580. Brown enviromental changes :aa starv 2 h(1)
    (c) 581. Brown enviromental changes :aa starv 4 h(1)
    (c) 584. Brown enviromental changes :Nitrogen Depletion 1 h(1)
    (c) 585. Brown enviromental changes :Nitrogen Depletion 2 h(1)
    (c) 586. Brown enviromental changes :Nitrogen Depletion 4 h(1)
    (c) DES460 + 0.02% MMS - 15 min


GCN4 -*-> YMC1


    (c) 89. Expression in response to 3-aminotriazole(1)
    (c) 95. Expression in response to 50ug/mL FK506(1)
    (c) 332. Rosetta 2000: Expression in cells with CMD1 under tet promoter(1)
    (c) 387. Rosetta 2000: Expression in cells with ERG11 under tet promoter(1)
    (c) 395. Rosetta 2000: Expression in response to 2-deoxy-D-glucose(1)
```

(c) 401. Rosetta 2000: Expression in response to HU(1)
(c) 402. Rosetta 2000: Expression in response to Itraconazole(1)
(c) 403. Rosetta 2000: Expression in response to Lovastatin(1)
(c) 406. Rosetta 2000: Expression in response to Terbinafine(1)
(c) 407. Rosetta 2000: Expression in response to Tunicamycin(1)
(c) 445. Expression in response to 0.1% MMS for 60 min (average of 3 experiments)(1)
(c) 446. Expression in response to 0.1% MMS for 10 min(1)
(c) 447. Expression in response to 0.1% MMS for 30 min(1)
(c) 448. Expression in response to 0.1% MMS for 60 min(1)
(c) 449. Expression in response to 0.1% MMS for 60 min(1)
(c) 462. Expression in response to 0.05% MMS for 60 min(1)
(c) 463. Expression in response to 0.1% MMS for 60 min(1)
(c) 479. Expression in diploid cells in response to rapamycin (100nM) for: 15min,30min,90min,120min(3)
(c) 533. Brown enviromental changes :1 mM Menadione (10 min)redo(1)
(c) 579. Brown enviromental changes :aa starv 1 h(1)
(c) 580. Brown enviromental changes :aa starv 2 h(1)
(c) 581. Brown enviromental changes :aa starv 4 h(1)
(c) 584. Brown enviromental changes :Nitrogen Depletion 1 h(1)
(c) 585. Brown enviromental changes :Nitrogen Depletion 2 h(1)
(c) 586. Brown enviromental changes :Nitrogen Depletion 4 h(1)
(c) DES460 + 0.02% MMS - 15 min

GCN4 -*-> YMC2

(c) 89. Expression in response to 3-aminotriazole(1)
(c) 95. Expression in response to 50ug/mL FK506(1)
(c) 332. Rosetta 2000: Expression in cells with CMD1 under tet promoter(1)
(c) 387. Rosetta 2000: Expression in cells with ERG11 under tet promoter(1)
(c) 395. Rosetta 2000: Expression in response to 2-deoxy-D-glucose(1)
(c) 401. Rosetta 2000: Expression in response to HU(1)
(c) 402. Rosetta 2000: Expression in response to Itraconazole(1)
(c) 403. Rosetta 2000: Expression in response to Lovastatin(1)
(c) 406. Rosetta 2000: Expression in response to Terbinafine(1)
(c) 407. Rosetta 2000: Expression in response to Tunicamycin(1)
(c) 445. Expression in response to 0.1% MMS for 60 min (average of 3 experiments)(1)
(c) 446. Expression in response to 0.1% MMS for 10 min(1)
(c) 447. Expression in response to 0.1% MMS for 30 min(1)
(c) 448. Expression in response to 0.1% MMS for 60 min(1)
(c) 449. Expression in response to 0.1% MMS for 60 min(1)
(c) 462. Expression in response to 0.05% MMS for 60 min(1)
(c) 463. Expression in response to 0.1% MMS for 60 min(1)
(c) 479. Expression in diploid cells in response to rapamycin (100nM) for: 15min,30min,90min,120min(3)
(c) 533. Brown enviromental changes :1 mM Menadione (10 min)redo(1)
(c) 579. Brown enviromental changes :aa starv 1 h(1)
(c) 580. Brown enviromental changes :aa starv 2 h(1)
(c) 581. Brown enviromental changes :aa starv 4 h(1)
(c) 584. Brown enviromental changes :Nitrogen Depletion 1 h(1)
(c) 585. Brown enviromental changes :Nitrogen Depletion 2 h(1)
(c) 586. Brown enviromental changes :Nitrogen Depletion 4 h(1)
(c) DES460 + 0.02% MMS - 15 min

GCN4 -*-> YNL129W

(c) 89. Expression in response to 3-aminotriazole(1)
(c) 95. Expression in response to 50ug/mL FK506(1)
(c) 332. Rosetta 2000: Expression in cells with CMD1 under tet promoter(1)
(c) 387. Rosetta 2000: Expression in cells with ERG11 under tet promoter(1)
(c) 395. Rosetta 2000: Expression in response to 2-deoxy-D-glucose(1)
(c) 401. Rosetta 2000: Expression in response to HU(1)
(c) 402. Rosetta 2000: Expression in response to Itraconazole(1)
(c) 403. Rosetta 2000: Expression in response to Lovastatin(1)
(c) 406. Rosetta 2000: Expression in response to Terbinafine(1)
(c) 407. Rosetta 2000: Expression in response to Tunicamycin(1)
(c) 445. Expression in response to 0.1% MMS for 60 min (average of 3 experiments)(1)
(c) 446. Expression in response to 0.1% MMS for 10 min(1)
(c) 447. Expression in response to 0.1% MMS for 30 min(1)
(c) 448. Expression in response to 0.1% MMS for 60 min(1)
(c) 449. Expression in response to 0.1% MMS for 60 min(1)
(c) 462. Expression in response to 0.05% MMS for 60 min(1)
(c) 463. Expression in response to 0.1% MMS for 60 min(1)
(c) 479. Expression in diploid cells in response to rapamycin (100nM) for: 15min,30min,90min,120min(3)
(c) 533. Brown enviromental changes :1 mM Menadione (10 min)redo(1)
(c) 579. Brown enviromental changes :aa starv 1 h(1)
(c) 580. Brown enviromental changes :aa starv 2 h(1)
(c) 581. Brown enviromental changes :aa starv 4 h(1)
(c) 584. Brown enviromental changes :Nitrogen Depletion 1 h(1)
(c) 585. Brown enviromental changes :Nitrogen Depletion 2 h(1)
(c) 586. Brown enviromental changes :Nitrogen Depletion 4 h(1)
(c) DES460 + 0.02% MMS - 15 min

GCN4 -*-> YOR203W

(c) 89. Expression in response to 3-aminotriazole(1)
(c) 95. Expression in response to 50ug/mL FK506(1)
(c) 332. Rosetta 2000: Expression in cells with CMD1 under tet promoter(1)
(c) 387. Rosetta 2000: Expression in cells with ERG11 under tet promoter(1)
(c) 395. Rosetta 2000: Expression in response to 2-deoxy-D-glucose(1)
(c) 401. Rosetta 2000: Expression in response to HU(1)
(c) 402. Rosetta 2000: Expression in response to Itraconazole(1)
(c) 403. Rosetta 2000: Expression in response to Lovastatin(1)
(c) 406. Rosetta 2000: Expression in response to Terbinafine(1)
(c) 407. Rosetta 2000: Expression in response to Tunicamycin(1)
(c) 445. Expression in response to 0.1% MMS for 60 min (average of 3 experiments)(1)
(c) 446. Expression in response to 0.1% MMS for 10 min(1)
(c) 447. Expression in response to 0.1% MMS for 30 min(1)
(c) 448. Expression in response to 0.1% MMS for 60 min(1)
(c) 449. Expression in response to 0.1% MMS for 60 min(1)
(c) 462. Expression in response to 0.05% MMS for 60 min(1)
(c) 463. Expression in response to 0.1% MMS for 60 min(1)
(c) 479. Expression in diploid cells in response to rapamycin (100nM) for: 15min,30min,90min,120min(3)
(c) 533. Brown enviromental changes :1 mM Menadione (10 min)redo(1)
(c) 579. Brown enviromental changes :aa starv 1 h(1)
(c) 580. Brown enviromental changes :aa starv 2 h(1)
(c) 581. Brown enviromental changes :aa starv 4 h(1)
(c) 584. Brown enviromental changes :Nitrogen Depletion 1 h(1)
(c) 585. Brown enviromental changes :Nitrogen Depletion 2 h(1)

(c) 586. Brown enviromental changes :Nitrogen Depletion 4 h(1)
(c) DES460 + 0.02% MMS - 15 min

GCR1 -*-> CDC19

(c) 5. Expression during the cell cycle (alpha factor arrest and release)(15)
(c) 6. Expression during the cell cycle (cdc15 arrest and release)(18)
(c) 7. Expression during the cell Cycle (cdc28)(5)
(c) 7. Expression during the cell Cycle (cdc28)(14)
(c) 7. Expression during the cell Cycle (cdc28)(15)
(c) 7. Expression during the cell Cycle (cdc28)(16)
(c) 7. Expression during the cell Cycle (cdc28)(17)
(c) 8. Expression during the cell cycle (cell size selection and release)(5)
(c) 8. Expression during the cell cycle (cell size selection and release)(8)
(c) 441. Expression in wild type versus strain TS19-4c under steady state conditions in YPD(1)
(c) 477. Expression in response to trichostatin A (TSA): 15min,30min,60min,120min(4)
(c) 496. Brown enviromental changes :Heat Shock 000 minutes hs-2(1)
(c) 497. Brown enviromental changes :Heat Shock 000 minutes hs-2(1)
(c) 498. Brown enviromental changes :Heat Shock 000 minutes hs-2(1)
(c) 506. Brown enviromental changes :37C to 25C shock - 60 min(1)
(c) 507. Brown enviromental changes :37C to 25C shock - 90 min(1)
(c) 516. Brown enviromental changes :33C vs. 30C - 90 minutes(1)
(c) 544. Brown enviromental changes :2.5mM DTT 030 min dtt-1(1)
(c) 550. Brown enviromental changes :dtt 000 min dtt-2(1)
(c) 551. Brown enviromental changes :dtt 015 min dtt-2(1)
(c) 552. Brown enviromental changes :dtt 030 min dtt-2(1)
(c) 553. Brown enviromental changes :dtt 060 min dtt-2(1)
(c) 611. Brown enviromental changes :YPD stationary phase 2 h ypd-1(1)
(c) 612. Brown enviromental changes :YPD stationary phase 4 h ypd-1(1)
(c) 681. Expression in response to 0.4M NaCl for 10 min in wild type(1)
(c) 684. Expression in response to 0.8M NaCl for 10 min in wild type(1)

GLN3 -*-> CPS1

(c) 428. Expression in strain PM38 (wild type) in response to 30 min 50 nM treatment with rapamycin in YPD(1)
(c) 429. Expression in strain YHE711 (wild type) in response to 30 min 50 nM treatment with rapamycin in YPD(1)
(c) 439. Expression in strain Jk9-3da (wild type) in response to 30 min 50 nM treatment with rapamycin in YPD(1)
(c) 442. Expression in strain PM38 (wild type) in response to 30 min 50 nM treatment with rapamycin in YPD(1)
(c) 479. Expression in diploid cells in response to rapamycin (100nM) for: 15min,30min,90min,120min(2)
(c) 578. Brown enviromental changes :aa starv 0.5 h(1)
(c) 579. Brown enviromental changes :aa starv 1 h(1)
(c) 580. Brown enviromental changes :aa starv 2 h(1)
(c) 581. Brown enviromental changes :aa starv 4 h(1)
(c) 582. Brown enviromental changes :aa starv 6 h(1)
(c) 583. Brown enviromental changes :Nitrogen Depletion 30 min.(1)
(c) 584. Brown enviromental changes :Nitrogen Depletion 1 h(1)
(c) 585. Brown enviromental changes :Nitrogen Depletion 2 h(1)
(c) 586. Brown enviromental changes :Nitrogen Depletion 4 h(1)
(c) 587. Brown enviromental changes :Nitrogen Depletion 8 h(1)
(c) 588. Brown enviromental changes :Nitrogen Depletion 12 h(1)
(c) 589. Brown enviromental changes :Nitrogen Depletion 1 d(1)
(c) 590. Brown enviromental changes :Nitrogen Depletion 2 d(1)
(c) 591. Brown enviromental changes :Nitrogen Depletion 3 d(1)
(c) 592. Brown enviromental changes :Nitrogen Depletion 5 d(1)

GLN3 -*-> LEE1

(c) 428. Expression in strain PM38 (wild type) in response to 30 min 50 nM treatment with rapamycin in YPD(1)
(c) 429. Expression in strain YHE711 (wild type) in response to 30 min 50 nM treatment with rapamycin in YPD(1)
(c) 439. Expression in strain Jk9-3da (wild type) in response to 30 min 50 nM treatment with rapamycin in YPD(1)
(c) 442. Expression in strain PM38 (wild type) in response to 30 min 50 nM treatment with rapamycin in YPD(1)
(c) 479. Expression in diploid cells in response to rapamycin (100nM) for: 15min,30min,90min,120min(2)
(c) 578. Brown enviromental changes :aa starv 0.5 h(1)
(c) 579. Brown enviromental changes :aa starv 1 h(1)
(c) 580. Brown enviromental changes :aa starv 2 h(1)
(c) 581. Brown enviromental changes :aa starv 4 h(1)
(c) 582. Brown enviromental changes :aa starv 6 h(1)
(c) 583. Brown enviromental changes :Nitrogen Depletion 30 min.(1)
(c) 584. Brown enviromental changes :Nitrogen Depletion 1 h(1)
(c) 585. Brown enviromental changes :Nitrogen Depletion 2 h(1)
(c) 586. Brown enviromental changes :Nitrogen Depletion 4 h(1)
(c) 587. Brown enviromental changes :Nitrogen Depletion 8 h(1)
(c) 588. Brown enviromental changes :Nitrogen Depletion 12 h(1)
(c) 589. Brown enviromental changes :Nitrogen Depletion 1 d(1)
(c) 590. Brown enviromental changes :Nitrogen Depletion 2 d(1)
(c) 591. Brown enviromental changes :Nitrogen Depletion 3 d(1)
(c) 592. Brown enviromental changes :Nitrogen Depletion 5 d(1)

GLN3 -*-> MEP1

(c) 428. Expression in strain PM38 (wild type) in response to 30 min 50 nM treatment with rapamycin in YPD(1)
(c) 429. Expression in strain YHE711 (wild type) in response to 30 min 50 nM treatment with rapamycin in YPD(1)
(c) 439. Expression in strain Jk9-3da (wild type) in response to 30 min 50 nM treatment with rapamycin in YPD(1)
(c) 442. Expression in strain PM38 (wild type) in response to 30 min 50 nM treatment with rapamycin in YPD(1)
(c) 479. Expression in diploid cells in response to rapamycin (100nM) for: 15min,30min,90min,120min(2)
(c) 578. Brown enviromental changes :aa starv 0.5 h(1)
(c) 579. Brown enviromental changes :aa starv 1 h(1)
(c) 580. Brown enviromental changes :aa starv 2 h(1)
(c) 581. Brown enviromental changes :aa starv 4 h(1)
(c) 582. Brown enviromental changes :aa starv 6 h(1)
(c) 583. Brown enviromental changes :Nitrogen Depletion 30 min.(1)
(c) 584. Brown enviromental changes :Nitrogen Depletion 1 h(1)
(c) 585. Brown enviromental changes :Nitrogen Depletion 2 h(1)
(c) 586. Brown enviromental changes :Nitrogen Depletion 4 h(1)
(c) 587. Brown enviromental changes :Nitrogen Depletion 8 h(1)
(c) 588. Brown enviromental changes :Nitrogen Depletion 12 h(1)
(c) 589. Brown enviromental changes :Nitrogen Depletion 1 d(1)
(c) 590. Brown enviromental changes :Nitrogen Depletion 2 d(1)
(c) 591. Brown enviromental changes :Nitrogen Depletion 3 d(1)
(c) 592. Brown enviromental changes :Nitrogen Depletion 5 d(1)

GLN3 -*-> MEP2

(c) 428. Expression in strain PM38 (wild type) in response to 30 min 50 nM treatment with rapamycin in YPD(1)
(c) 429. Expression in strain YHE711 (wild type) in response to 30 min 50 nM treatment with rapamycin in YPD(1)
(c) 439. Expression in strain Jk9-3da (wild type) in response to 30 min 50 nM treatment with rapamycin in YPD(1)
(c) 442. Expression in strain PM38 (wild type) in response to 30 min 50 nM treatment with rapamycin in YPD(1)
(c) 479. Expression in diploid cells in response to rapamycin (100nM) for: 15min,30min,90min,120min(2)
(c) 578. Brown enviromental changes :aa starv 0.5 h(1)
(c) 579. Brown enviromental changes :aa starv 1 h(1)
(c) 580. Brown enviromental changes :aa starv 2 h(1)
(c) 581. Brown enviromental changes :aa starv 4 h(1)
(c) 582. Brown enviromental changes :aa starv 6 h(1)
(c) 583. Brown enviromental changes :Nitrogen Depletion 30 min.(1)
(c) 584. Brown enviromental changes :Nitrogen Depletion 1 h(1)
(c) 585. Brown enviromental changes :Nitrogen Depletion 2 h(1)
(c) 586. Brown enviromental changes :Nitrogen Depletion 4 h(1)
(c) 587. Brown enviromental changes :Nitrogen Depletion 8 h(1)
(c) 588. Brown enviromental changes :Nitrogen Depletion 12 h(1)
(c) 589. Brown enviromental changes :Nitrogen Depletion 1 d(1)
(c) 590. Brown enviromental changes :Nitrogen Depletion 2 d(1)
(c) 591. Brown enviromental changes :Nitrogen Depletion 3 d(1)
(c) 592. Brown enviromental changes :Nitrogen Depletion 5 d(1)

GLN3 -*-> MET16

(c) 428. Expression in strain PM38 (wild type) in response to 30 min 50 nM treatment with rapamycin in YPD(1)
(c) 429. Expression in strain YHE711 (wild type) in response to 30 min 50 nM treatment with rapamycin in YPD(1)
(c) 439. Expression in strain Jk9-3da (wild type) in response to 30 min 50 nM treatment with rapamycin in YPD(1)
(c) 442. Expression in strain PM38 (wild type) in response to 30 min 50 nM treatment with rapamycin in YPD(1)
(c) 479. Expression in diploid cells in response to rapamycin (100nM) for: 15min,30min,90min,120min(2)
(c) 578. Brown enviromental changes :aa starv 0.5 h(1)
(c) 579. Brown enviromental changes :aa starv 1 h(1)
(c) 580. Brown enviromental changes :aa starv 2 h(1)
(c) 581. Brown enviromental changes :aa starv 4 h(1)
(c) 582. Brown enviromental changes :aa starv 6 h(1)
(c) 583. Brown enviromental changes :Nitrogen Depletion 30 min.(1)
(c) 584. Brown enviromental changes :Nitrogen Depletion 1 h(1)
(c) 585. Brown enviromental changes :Nitrogen Depletion 2 h(1)
(c) 586. Brown enviromental changes :Nitrogen Depletion 4 h(1)
(c) 587. Brown enviromental changes :Nitrogen Depletion 8 h(1)
(c) 588. Brown enviromental changes :Nitrogen Depletion 12 h(1)
(c) 589. Brown enviromental changes :Nitrogen Depletion 1 d(1)
(c) 590. Brown enviromental changes :Nitrogen Depletion 2 d(1)
(c) 591. Brown enviromental changes :Nitrogen Depletion 3 d(1)
(c) 592. Brown enviromental changes :Nitrogen Depletion 5 d(1)

GLN3 -*-> OPT2

(c) 428. Expression in strain PM38 (wild type) in response to 30 min 50 nM treatment with rapamycin in YPD(1)
(c) 429. Expression in strain YHE711 (wild type) in response to 30 min 50 nM treatment with rapamycin in YPD(1)
(c) 439. Expression in strain Jk9-3da (wild type) in response to 30 min 50 nM treatment with rapamycin in YPD(1)
(c) 442. Expression in strain PM38 (wild type) in response to 30 min 50 nM treatment with rapamycin in YPD(1)
(c) 479. Expression in diploid cells in response to rapamycin (100nM) for: 15min,30min,90min,120min(2)
(c) 578. Brown enviromental changes :aa starv 0.5 h(1)
(c) 579. Brown enviromental changes :aa starv 1 h(1)
(c) 580. Brown enviromental changes :aa starv 2 h(1)
(c) 581. Brown enviromental changes :aa starv 4 h(1)
(c) 582. Brown enviromental changes :aa starv 6 h(1)
(c) 583. Brown enviromental changes :Nitrogen Depletion 30 min.(1)
(c) 584. Brown enviromental changes :Nitrogen Depletion 1 h(1)
(c) 585. Brown enviromental changes :Nitrogen Depletion 2 h(1)
(c) 586. Brown enviromental changes :Nitrogen Depletion 4 h(1)
(c) 587. Brown enviromental changes :Nitrogen Depletion 8 h(1)
(c) 588. Brown enviromental changes :Nitrogen Depletion 12 h(1)
(c) 589. Brown enviromental changes :Nitrogen Depletion 1 d(1)
(c) 590. Brown enviromental changes :Nitrogen Depletion 2 d(1)
(c) 591. Brown enviromental changes :Nitrogen Depletion 3 d(1)
(c) 592. Brown enviromental changes :Nitrogen Depletion 5 d(1)

GLN3 -*-> YBR147W

(c) 428. Expression in strain PM38 (wild type) in response to 30 min 50 nM treatment with rapamycin in YPD(1)
(c) 429. Expression in strain YHE711 (wild type) in response to 30 min 50 nM treatment with rapamycin in YPD(1)
(c) 439. Expression in strain Jk9-3da (wild type) in response to 30 min 50 nM treatment with rapamycin in YPD(1)
(c) 442. Expression in strain PM38 (wild type) in response to 30 min 50 nM treatment with rapamycin in YPD(1)
(c) 479. Expression in diploid cells in response to rapamycin (100nM) for: 15min,30min,90min,120min(2)
(c) 578. Brown enviromental changes :aa starv 0.5 h(1)
(c) 579. Brown enviromental changes :aa starv 1 h(1)
(c) 580. Brown enviromental changes :aa starv 2 h(1)
(c) 581. Brown enviromental changes :aa starv 4 h(1)
(c) 582. Brown enviromental changes :aa starv 6 h(1)
(c) 583. Brown enviromental changes :Nitrogen Depletion 30 min.(1)
(c) 584. Brown enviromental changes :Nitrogen Depletion 1 h(1)
(c) 585. Brown enviromental changes :Nitrogen Depletion 2 h(1)
(c) 586. Brown enviromental changes :Nitrogen Depletion 4 h(1)
(c) 587. Brown enviromental changes :Nitrogen Depletion 8 h(1)
(c) 588. Brown enviromental changes :Nitrogen Depletion 12 h(1)
(c) 589. Brown enviromental changes :Nitrogen Depletion 1 d(1)
(c) 590. Brown enviromental changes :Nitrogen Depletion 2 d(1)
(c) 591. Brown enviromental changes :Nitrogen Depletion 3 d(1)
(c) 592. Brown enviromental changes :Nitrogen Depletion 5 d(1)

GLN3 -*-> YEL072W

(c) 428. Expression in strain PM38 (wild type) in response to 30 min 50 nM treatment with rapamycin in YPD(1)
(c) 429. Expression in strain YHE711 (wild type) in response to 30 min 50 nM treatment with rapamycin in YPD(1)
(c) 439. Expression in strain Jk9-3da (wild type) in response to 30 min 50 nM treatment with rapamycin in YPD(1)
(c) 442. Expression in strain PM38 (wild type) in response to 30 min 50 nM treatment with rapamycin in YPD(1)
(c) 479. Expression in diploid cells in response to rapamycin (100nM) for: 15min,30min,90min,120min(2)
(c) 578. Brown enviromental changes :aa starv 0.5 h(1)
(c) 579. Brown enviromental changes :aa starv 1 h(1)
(c) 580. Brown enviromental changes :aa starv 2 h(1)
(c) 581. Brown enviromental changes :aa starv 4 h(1)
(c) 582. Brown enviromental changes :aa starv 6 h(1)
(c) 583. Brown enviromental changes :Nitrogen Depletion 30 min.(1)

(c) 584. Brown enviromental changes :Nitrogen Depletion 1 h(1)
(c) 585. Brown enviromental changes :Nitrogen Depletion 2 h(1)
(c) 586. Brown enviromental changes :Nitrogen Depletion 4 h(1)
(c) 587. Brown enviromental changes :Nitrogen Depletion 8 h(1)
(c) 588. Brown enviromental changes :Nitrogen Depletion 12 h(1)
(c) 589. Brown enviromental changes :Nitrogen Depletion 1 d(1)
(c) 590. Brown enviromental changes :Nitrogen Depletion 2 d(1)
(c) 591. Brown enviromental changes :Nitrogen Depletion 3 d(1)
(c) 592. Brown enviromental changes :Nitrogen Depletion 5 d(1)

GLN3 -*-> YGR125W

(c) 428. Expression in strain PM38 (wild type) in response to 30 min 50 nM treatment with rapamycin in YPD(1)
(c) 429. Expression in strain YHE711 (wild type) in response to 30 min 50 nM treatment with rapamycin in YPD(1)
(c) 439. Expression in strain Jk9-3da (wild type) in response to 30 min 50 nM treatment with rapamycin in YPD(1)
(c) 442. Expression in strain PM38 (wild type) in response to 30 min 50 nM treatment with rapamycin in YPD(1)
(c) 479. Expression in diploid cells in response to rapamycin (100nM) for: 15min,30min,90min,120min(2)
(c) 578. Brown enviromental changes :aa starv 0.5 h(1)
(c) 579. Brown enviromental changes :aa starv 1 h(1)
(c) 580. Brown enviromental changes :aa starv 2 h(1)
(c) 581. Brown enviromental changes :aa starv 4 h(1)
(c) 582. Brown enviromental changes :aa starv 6 h(1)
(c) 583. Brown enviromental changes :Nitrogen Depletion 30 min.(1)
(c) 584. Brown enviromental changes :Nitrogen Depletion 1 h(1)
(c) 585. Brown enviromental changes :Nitrogen Depletion 2 h(1)
(c) 586. Brown enviromental changes :Nitrogen Depletion 4 h(1)
(c) 587. Brown enviromental changes :Nitrogen Depletion 8 h(1)
(c) 588. Brown enviromental changes :Nitrogen Depletion 12 h(1)
(c) 589. Brown enviromental changes :Nitrogen Depletion 1 d(1)
(c) 590. Brown enviromental changes :Nitrogen Depletion 2 d(1)
(c) 591. Brown enviromental changes :Nitrogen Depletion 3 d(1)
(c) 592. Brown enviromental changes :Nitrogen Depletion 5 d(1)

GLN3 -*-> YIR042C

(c) 428. Expression in strain PM38 (wild type) in response to 30 min 50 nM treatment with rapamycin in YPD(1)
(c) 429. Expression in strain YHE711 (wild type) in response to 30 min 50 nM treatment with rapamycin in YPD(1)
(c) 439. Expression in strain Jk9-3da (wild type) in response to 30 min 50 nM treatment with rapamycin in YPD(1)
(c) 442. Expression in strain PM38 (wild type) in response to 30 min 50 nM treatment with rapamycin in YPD(1)
(c) 479. Expression in diploid cells in response to rapamycin (100nM) for: 15min,30min,90min,120min(2)
(c) 578. Brown enviromental changes :aa starv 0.5 h(1)
(c) 579. Brown enviromental changes :aa starv 1 h(1)
(c) 580. Brown enviromental changes :aa starv 2 h(1)
(c) 581. Brown enviromental changes :aa starv 4 h(1)
(c) 582. Brown enviromental changes :aa starv 6 h(1)
(c) 583. Brown enviromental changes :Nitrogen Depletion 30 min.(1)
(c) 584. Brown enviromental changes :Nitrogen Depletion 1 h(1)
(c) 585. Brown enviromental changes :Nitrogen Depletion 2 h(1)
(c) 586. Brown enviromental changes :Nitrogen Depletion 4 h(1)
(c) 587. Brown enviromental changes :Nitrogen Depletion 8 h(1)
(c) 588. Brown enviromental changes :Nitrogen Depletion 12 h(1)
(c) 589. Brown enviromental changes :Nitrogen Depletion 1 d(1)
(c) 590. Brown enviromental changes :Nitrogen Depletion 2 d(1)
(c) 591. Brown enviromental changes :Nitrogen Depletion 3 d(1)
(c) 592. Brown enviromental changes :Nitrogen Depletion 5 d(1)

GLN3 -*-> YKR033C

(c) 428. Expression in strain PM38 (wild type) in response to 30 min 50 nM treatment with rapamycin in YPD(1)
(c) 429. Expression in strain YHE711 (wild type) in response to 30 min 50 nM treatment with rapamycin in YPD(1)
(c) 439. Expression in strain Jk9-3da (wild type) in response to 30 min 50 nM treatment with rapamycin in YPD(1)
(c) 442. Expression in strain PM38 (wild type) in response to 30 min 50 nM treatment with rapamycin in YPD(1)
(c) 479. Expression in diploid cells in response to rapamycin (100nM) for: 15min,30min,90min,120min(2)
(c) 578. Brown enviromental changes :aa starv 0.5 h(1)
(c) 579. Brown enviromental changes :aa starv 1 h(1)
(c) 580. Brown enviromental changes :aa starv 2 h(1)
(c) 581. Brown enviromental changes :aa starv 4 h(1)
(c) 582. Brown enviromental changes :aa starv 6 h(1)
(c) 583. Brown enviromental changes :Nitrogen Depletion 30 min.(1)
(c) 584. Brown enviromental changes :Nitrogen Depletion 1 h(1)
(c) 585. Brown enviromental changes :Nitrogen Depletion 2 h(1)
(c) 586. Brown enviromental changes :Nitrogen Depletion 4 h(1)
(c) 587. Brown enviromental changes :Nitrogen Depletion 8 h(1)
(c) 588. Brown enviromental changes :Nitrogen Depletion 12 h(1)
(c) 589. Brown enviromental changes :Nitrogen Depletion 1 d(1)
(c) 590. Brown enviromental changes :Nitrogen Depletion 2 d(1)
(c) 591. Brown enviromental changes :Nitrogen Depletion 3 d(1)
(c) 592. Brown enviromental changes :Nitrogen Depletion 5 d(1)

GLN3 -*-> YMR088C

(c) 428. Expression in strain PM38 (wild type) in response to 30 min 50 nM treatment with rapamycin in YPD(1)
(c) 429. Expression in strain YHE711 (wild type) in response to 30 min 50 nM treatment with rapamycin in YPD(1)
(c) 439. Expression in strain Jk9-3da (wild type) in response to 30 min 50 nM treatment with rapamycin in YPD(1)
(c) 442. Expression in strain PM38 (wild type) in response to 30 min 50 nM treatment with rapamycin in YPD(1)
(c) 479. Expression in diploid cells in response to rapamycin (100nM) for: 15min,30min,90min,120min(2)
(c) 578. Brown enviromental changes :aa starv 0.5 h(1)
(c) 579. Brown enviromental changes :aa starv 1 h(1)
(c) 580. Brown enviromental changes :aa starv 2 h(1)
(c) 581. Brown enviromental changes :aa starv 4 h(1)
(c) 582. Brown enviromental changes :aa starv 6 h(1)
(c) 583. Brown enviromental changes :Nitrogen Depletion 30 min.(1)
(c) 584. Brown enviromental changes :Nitrogen Depletion 1 h(1)
(c) 585. Brown enviromental changes :Nitrogen Depletion 2 h(1)
(c) 586. Brown enviromental changes :Nitrogen Depletion 4 h(1)
(c) 587. Brown enviromental changes :Nitrogen Depletion 8 h(1)
(c) 588. Brown enviromental changes :Nitrogen Depletion 12 h(1)
(c) 589. Brown enviromental changes :Nitrogen Depletion 1 d(1)
(c) 590. Brown enviromental changes :Nitrogen Depletion 2 d(1)
(c) 591. Brown enviromental changes :Nitrogen Depletion 3 d(1)
(c) 592. Brown enviromental changes :Nitrogen Depletion 5 d(1)

GLN3 -*-> YNL129W

(c) 428. Expression in strain PM38 (wild type) in response to 30 min 50 nM treatment with rapamycin in YPD(1)
(c) 429. Expression in strain YHE711 (wild type) in response to 30 min 50 nM treatment with rapamycin in YPD(1)
(c) 439. Expression in strain Jk9-3da (wild type) in response to 30 min 50 nM treatment with rapamycin in YPD(1)
(c) 442. Expression in strain PM38 (wild type) in response to 30 min 50 nM treatment with rapamycin in YPD(1)
(c) 479. Expression in diploid cells in response to rapamycin (100nM) for: 15min,30min,90min,120min(2)
(c) 578. Brown enviromental changes :aa starv 0.5 h(1)
(c) 579. Brown enviromental changes :aa starv 1 h(1)
(c) 580. Brown enviromental changes :aa starv 2 h(1)
(c) 581. Brown enviromental changes :aa starv 4 h(1)
(c) 582. Brown enviromental changes :aa starv 6 h(1)
(c) 583. Brown enviromental changes :Nitrogen Depletion 30 min.(1)
(c) 584. Brown enviromental changes :Nitrogen Depletion 1 h(1)
(c) 585. Brown enviromental changes :Nitrogen Depletion 2 h(1)
(c) 586. Brown enviromental changes :Nitrogen Depletion 4 h(1)
(c) 587. Brown enviromental changes :Nitrogen Depletion 8 h(1)
(c) 588. Brown enviromental changes :Nitrogen Depletion 12 h(1)
(c) 589. Brown enviromental changes :Nitrogen Depletion 1 d(1)
(c) 590. Brown enviromental changes :Nitrogen Depletion 2 d(1)
(c) 591. Brown enviromental changes :Nitrogen Depletion 3 d(1)
(c) 592. Brown enviromental changes :Nitrogen Depletion 5 d(1)

HAP3 -*-> AAP1'

(c) 8. Expression during the cell cycle (cell size selection and release)(8)
(c) 89. Expression in response to 3-aminotriazole(1)
(c) 95. Expression in response to 50ug/mL FK506(1)
(c) 386. Rosetta 2000: Expression in cells with CDC42 under tet promoter(1)
(c) 387. Rosetta 2000: Expression in cells with ERG11 under tet promoter(1)
(c) 402. Rosetta 2000: Expression in response to Itraconazole(1)
(c) 403. Rosetta 2000: Expression in response to Lovastatin(1)
(c) 406. Rosetta 2000: Expression in response to Terbinafine(1)
(c) 429. Expression in strain YHE711 (wild type) in response to 30 min 50 nM treatment with rapamycin in YPD(1)
(c) 446. Expression in response to 0.1% MMS for 10 min(1)
(c) 447. Expression in response to 0.1% MMS for 30 min(1)
(c) 448. Expression in response to 0.1% MMS for 60 min(1)
(c) 449. Expression in response to 0.1% MMS for 60 min(1)
(c) 479. Expression in diploid cells in response to rapamycin (100nM) for: 15min,30min,90min,120min(2)
(c) 503. Brown enviromental changes :37C to 25C shock - 15 min(1)
(c) 504. Brown enviromental changes :37C to 25C shock - 30 min(1)
(c) 506. Brown enviromental changes :37C to 25C shock - 60 min(1)
(c) 533. Brown enviromental changes :1 mM Menadione (10 min)redo(1)
(c) 570. Brown enviromental changes :1M sorbitol - 90 min(1)
(c) 635. Brown enviromental changes :YAP1 overexpression(1)
(c) MHY1 (crt1) vs CRY1 (wild type)
(c) 100 microM BCS 30 min

HAP3 -*-> ARO4

(c) 8. Expression during the cell cycle (cell size selection and release)(8)
(c) 89. Expression in response to 3-aminotriazole(1)
(c) 95. Expression in response to 50ug/mL FK506(1)
(c) 386. Rosetta 2000: Expression in cells with CDC42 under tet promoter(1)
(c) 387. Rosetta 2000: Expression in cells with ERG11 under tet promoter(1)
(c) 402. Rosetta 2000: Expression in response to Itraconazole(1)
(c) 403. Rosetta 2000: Expression in response to Lovastatin(1)
(c) 406. Rosetta 2000: Expression in response to Terbinafine(1)
(c) 429. Expression in strain YHE711 (wild type) in response to 30 min 50 nM treatment with rapamycin in YPD(1)
(c) 446. Expression in response to 0.1% MMS for 10 min(1)
(c) 447. Expression in response to 0.1% MMS for 30 min(1)
(c) 448. Expression in response to 0.1% MMS for 60 min(1)
(c) 449. Expression in response to 0.1% MMS for 60 min(1)
(c) 479. Expression in diploid cells in response to rapamycin (100nM) for: 15min,30min,90min,120min(2)
(c) 503. Brown enviromental changes :37C to 25C shock - 15 min(1)
(c) 504. Brown enviromental changes :37C to 25C shock - 30 min(1)
(c) 506. Brown enviromental changes :37C to 25C shock - 60 min(1)
(c) 533. Brown enviromental changes :1 mM Menadione (10 min)redo(1)
(c) 570. Brown enviromental changes :1M sorbitol - 90 min(1)
(c) 635. Brown enviromental changes :YAP1 overexpression(1)
(c) MHY1 (crt1) vs CRY1 (wild type)
(c) 100 microM BCS 30 min

HAP3 -*-> ARO8

(c) 8. Expression during the cell cycle (cell size selection and release)(8)
(c) 89. Expression in response to 3-aminotriazole(1)
(c) 95. Expression in response to 50ug/mL FK506(1)
(c) 386. Rosetta 2000: Expression in cells with CDC42 under tet promoter(1)
(c) 387. Rosetta 2000: Expression in cells with ERG11 under tet promoter(1)
(c) 402. Rosetta 2000: Expression in response to Itraconazole(1)
(c) 403. Rosetta 2000: Expression in response to Lovastatin(1)
(c) 406. Rosetta 2000: Expression in response to Terbinafine(1)
(c) 429. Expression in strain YHE711 (wild type) in response to 30 min 50 nM treatment with rapamycin in YPD(1)
(c) 446. Expression in response to 0.1% MMS for 10 min(1)
(c) 447. Expression in response to 0.1% MMS for 30 min(1)
(c) 448. Expression in response to 0.1% MMS for 60 min(1)
(c) 449. Expression in response to 0.1% MMS for 60 min(1)
(c) 479. Expression in diploid cells in response to rapamycin (100nM) for: 15min,30min,90min,120min(2)
(c) 503. Brown enviromental changes :37C to 25C shock - 15 min(1)
(c) 504. Brown enviromental changes :37C to 25C shock - 30 min(1)
(c) 506. Brown enviromental changes :37C to 25C shock - 60 min(1)
(c) 533. Brown enviromental changes :1 mM Menadione (10 min)redo(1)
(c) 570. Brown enviromental changes :1M sorbitol - 90 min(1)
(c) 635. Brown enviromental changes :YAP1 overexpression(1)
(c) MHY1 (crt1) vs CRY1 (wild type)
(c) 100 microM BCS 30 min

HAP3 -*-> ASN2

(c) 8. Expression during the cell cycle (cell size selection and release)(8)
(c) 89. Expression in response to 3-aminotriazole(1)
(c) 95. Expression in response to 50ug/mL FK506(1)
(c) 386. Rosetta 2000: Expression in cells with CDC42 under tet promoter(1)

(c) 387. Rosetta 2000: Expression in cells with ERG11 under tet promoter(1)
(c) 402. Rosetta 2000: Expression in response to Itraconazole(1)
(c) 403. Rosetta 2000: Expression in response to Lovastatin(1)
(c) 406. Rosetta 2000: Expression in response to Terbinafine(1)
(c) 429. Expression in strain YHE711 (wild type) in response to 30 min 50 nM treatment with rapamycin in YPD(1)
(c) 446. Expression in response to 0.1% MMS for 10 min(1)
(c) 447. Expression in response to 0.1% MMS for 30 min(1)
(c) 448. Expression in response to 0.1% MMS for 60 min(1)
(c) 449. Expression in response to 0.1% MMS for 60 min(1)
(c) 479. Expression in diploid cells in response to rapamycin (100nM) for: 15min,30min,90min,120min(2)
(c) 503. Brown enviromental changes :37C to 25C shock - 15 min(1)
(c) 504. Brown enviromental changes :37C to 25C shock - 30 min(1)
(c) 506. Brown enviromental changes :37C to 25C shock - 60 min(1)
(c) 533. Brown enviromental changes :1 mM Menadione (10 min)redo(1)
(c) 570. Brown enviromental changes :1M sorbitol - 90 min(1)
(c) 635. Brown enviromental changes :YAP1 overexpression(1)
(c) MHY1 (crt1) vs CRY1 (wild type)
(c) 100 microM BCS 30 min

HAP3 -*-> CPA2

(c) 8. Expression during the cell cycle (cell size selection and release)(8)
(c) 89. Expression in response to 3-aminotriazole(1)
(c) 95. Expression in response to 50ug/mL FK506(1)
(c) 386. Rosetta 2000: Expression in cells with CDC42 under tet promoter(1)
(c) 387. Rosetta 2000: Expression in cells with ERG11 under tet promoter(1)
(c) 402. Rosetta 2000: Expression in response to Itraconazole(1)
(c) 403. Rosetta 2000: Expression in response to Lovastatin(1)
(c) 406. Rosetta 2000: Expression in response to Terbinafine(1)
(c) 429. Expression in strain YHE711 (wild type) in response to 30 min 50 nM treatment with rapamycin in YPD(1)
(c) 446. Expression in response to 0.1% MMS for 10 min(1)
(c) 447. Expression in response to 0.1% MMS for 30 min(1)
(c) 448. Expression in response to 0.1% MMS for 60 min(1)
(c) 449. Expression in response to 0.1% MMS for 60 min(1)
(c) 479. Expression in diploid cells in response to rapamycin (100nM) for: 15min,30min,90min,120min(2)
(c) 503. Brown enviromental changes :37C to 25C shock - 15 min(1)
(c) 504. Brown enviromental changes :37C to 25C shock - 30 min(1)
(c) 506. Brown enviromental changes :37C to 25C shock - 60 min(1)
(c) 533. Brown enviromental changes :1 mM Menadione (10 min)redo(1)
(c) 570. Brown enviromental changes :1M sorbitol - 90 min(1)
(c) 635. Brown enviromental changes :YAP1 overexpression(1)
(c) MHY1 (crt1) vs CRY1 (wild type)
(c) 100 microM BCS 30 min

HAP3 -*-> HIS7

(c) 8. Expression during the cell cycle (cell size selection and release)(8)
(c) 89. Expression in response to 3-aminotriazole(1)
(c) 95. Expression in response to 50ug/mL FK506(1)
(c) 386. Rosetta 2000: Expression in cells with CDC42 under tet promoter(1)
(c) 387. Rosetta 2000: Expression in cells with ERG11 under tet promoter(1)
(c) 402. Rosetta 2000: Expression in response to Itraconazole(1)
(c) 403. Rosetta 2000: Expression in response to Lovastatin(1)
(c) 406. Rosetta 2000: Expression in response to Terbinafine(1)
(c) 429. Expression in strain YHE711 (wild type) in response to 30 min 50 nM treatment with rapamycin in YPD(1)
(c) 446. Expression in response to 0.1% MMS for 10 min(1)
(c) 447. Expression in response to 0.1% MMS for 30 min(1)
(c) 448. Expression in response to 0.1% MMS for 60 min(1)
(c) 449. Expression in response to 0.1% MMS for 60 min(1)
(c) 479. Expression in diploid cells in response to rapamycin (100nM) for: 15min,30min,90min,120min(2)
(c) 503. Brown enviromental changes :37C to 25C shock - 15 min(1)
(c) 504. Brown enviromental changes :37C to 25C shock - 30 min(1)
(c) 506. Brown enviromental changes :37C to 25C shock - 60 min(1)
(c) 533. Brown enviromental changes :1 mM Menadione (10 min)redo(1)
(c) 570. Brown enviromental changes :1M sorbitol - 90 min(1)
(c) 635. Brown enviromental changes :YAP1 overexpression(1)
(c) MHY1 (crt1) vs CRY1 (wild type)
(c) 100 microM BCS 30 min

HAP3 -*-> HOM3

(c) 8. Expression during the cell cycle (cell size selection and release)(8)
(c) 89. Expression in response to 3-aminotriazole(1)
(c) 95. Expression in response to 50ug/mL FK506(1)
(c) 386. Rosetta 2000: Expression in cells with CDC42 under tet promoter(1)
(c) 387. Rosetta 2000: Expression in cells with ERG11 under tet promoter(1)
(c) 402. Rosetta 2000: Expression in response to Itraconazole(1)
(c) 403. Rosetta 2000: Expression in response to Lovastatin(1)
(c) 406. Rosetta 2000: Expression in response to Terbinafine(1)
(c) 429. Expression in strain YHE711 (wild type) in response to 30 min 50 nM treatment with rapamycin in YPD(1)
(c) 446. Expression in response to 0.1% MMS for 10 min(1)
(c) 447. Expression in response to 0.1% MMS for 30 min(1)
(c) 448. Expression in response to 0.1% MMS for 60 min(1)
(c) 449. Expression in response to 0.1% MMS for 60 min(1)
(c) 479. Expression in diploid cells in response to rapamycin (100nM) for: 15min,30min,90min,120min(2)
(c) 503. Brown enviromental changes :37C to 25C shock - 15 min(1)
(c) 504. Brown enviromental changes :37C to 25C shock - 30 min(1)
(c) 506. Brown enviromental changes :37C to 25C shock - 60 min(1)
(c) 533. Brown enviromental changes :1 mM Menadione (10 min)redo(1)
(c) 570. Brown enviromental changes :1M sorbitol - 90 min(1)
(c) 635. Brown enviromental changes :YAP1 overexpression(1)
(c) MHY1 (crt1) vs CRY1 (wild type)
(c) 100 microM BCS 30 min

HAP3 -*-| KRS1

(c) 8. Expression during the cell cycle (cell size selection and release)(8)
(c) 89. Expression in response to 3-aminotriazole(1)
(c) 95. Expression in response to 50ug/mL FK506(1)
(c) 386. Rosetta 2000: Expression in cells with CDC42 under tet promoter(1)
(c) 387. Rosetta 2000: Expression in cells with ERG11 under tet promoter(1)
(c) 402. Rosetta 2000: Expression in response to Itraconazole(1)
(c) 403. Rosetta 2000: Expression in response to Lovastatin(1)
(c) 406. Rosetta 2000: Expression in response to Terbinafine(1)

(c) 429. Expression in strain YHE711 (wild type) in response to 30 min 50 nM treatment with rapamycin in YPD(1)
(c) 446. Expression in response to 0.1% MMS for 10 min(1)
(c) 447. Expression in response to 0.1% MMS for 30 min(1)
(c) 448. Expression in response to 0.1% MMS for 60 min(1)
(c) 449. Expression in response to 0.1% MMS for 60 min(1)
(c) 479. Expression in diploid cells in response to rapamycin (100nM) for: 15min,30min,90min,120min(2)
(c) 503. Brown enviromental changes :37C to 25C shock - 15 min(1)
(c) 504. Brown enviromental changes :37C to 25C shock - 30 min(1)
(c) 506. Brown enviromental changes :37C to 25C shock - 60 min(1)
(c) 533. Brown enviromental changes :1 mM Menadione (10 min)redo(1)
(c) 570. Brown enviromental changes :1M sorbitol - 90 min(1)
(c) 635. Brown enviromental changes :YAP1 overexpression(1)
(c) MHY1 (crt1) vs CRY1 (wild type)
(c) 100 microM BCS 30 min

HAP3 -*-> LEU4

(c) 8. Expression during the cell cycle (cell size selection and release)(8)
(c) 89. Expression in response to 3-aminotriazole(1)
(c) 95. Expression in response to 50ug/mL FK506(1)
(c) 386. Rosetta 2000: Expression in cells with CDC42 under tet promoter(1)
(c) 387. Rosetta 2000: Expression in cells with ERG11 under tet promoter(1)
(c) 402. Rosetta 2000: Expression in response to Itraconazole(1)
(c) 403. Rosetta 2000: Expression in response to Lovastatin(1)
(c) 406. Rosetta 2000: Expression in response to Terbinafine(1)
(c) 429. Expression in strain YHE711 (wild type) in response to 30 min 50 nM treatment with rapamycin in YPD(1)
(c) 446. Expression in response to 0.1% MMS for 10 min(1)
(c) 447. Expression in response to 0.1% MMS for 30 min(1)
(c) 448. Expression in response to 0.1% MMS for 60 min(1)
(c) 449. Expression in response to 0.1% MMS for 60 min(1)
(c) 479. Expression in diploid cells in response to rapamycin (100nM) for: 15min,30min,90min,120min(2)
(c) 503. Brown enviromental changes :37C to 25C shock - 15 min(1)
(c) 504. Brown enviromental changes :37C to 25C shock - 30 min(1)
(c) 506. Brown enviromental changes :37C to 25C shock - 60 min(1)
(c) 533. Brown enviromental changes :1 mM Menadione (10 min)redo(1)
(c) 570. Brown enviromental changes :1M sorbitol - 90 min(1)
(c) 635. Brown enviromental changes :YAP1 overexpression(1)
(c) MHY1 (crt1) vs CRY1 (wild type)
(c) 100 microM BCS 30 min

HAP3 -*-> PRO2

(c) 8. Expression during the cell cycle (cell size selection and release)(8)
(c) 89. Expression in response to 3-aminotriazole(1)
(c) 95. Expression in response to 50ug/mL FK506(1)
(c) 386. Rosetta 2000: Expression in cells with CDC42 under tet promoter(1)
(c) 387. Rosetta 2000: Expression in cells with ERG11 under tet promoter(1)
(c) 402. Rosetta 2000: Expression in response to Itraconazole(1)
(c) 403. Rosetta 2000: Expression in response to Lovastatin(1)
(c) 406. Rosetta 2000: Expression in response to Terbinafine(1)
(c) 429. Expression in strain YHE711 (wild type) in response to 30 min 50 nM treatment with rapamycin in YPD(1)
(c) 446. Expression in response to 0.1% MMS for 10 min(1)
(c) 447. Expression in response to 0.1% MMS for 30 min(1)
(c) 448. Expression in response to 0.1% MMS for 60 min(1)
(c) 449. Expression in response to 0.1% MMS for 60 min(1)
(c) 479. Expression in diploid cells in response to rapamycin (100nM) for: 15min,30min,90min,120min(2)
(c) 503. Brown enviromental changes :37C to 25C shock - 15 min(1)
(c) 504. Brown enviromental changes :37C to 25C shock - 30 min(1)
(c) 506. Brown enviromental changes :37C to 25C shock - 60 min(1)
(c) 533. Brown enviromental changes :1 mM Menadione (10 min)redo(1)
(c) 570. Brown enviromental changes :1M sorbitol - 90 min(1)
(c) 635. Brown enviromental changes :YAP1 overexpression(1)
(c) MHY1 (crt1) vs CRY1 (wild type)
(c) 100 microM BCS 30 min

HAP3 -*-> TRP2

(c) 8. Expression during the cell cycle (cell size selection and release)(8)
(c) 89. Expression in response to 3-aminotriazole(1)
(c) 95. Expression in response to 50ug/mL FK506(1)
(c) 386. Rosetta 2000: Expression in cells with CDC42 under tet promoter(1)
(c) 387. Rosetta 2000: Expression in cells with ERG11 under tet promoter(1)
(c) 402. Rosetta 2000: Expression in response to Itraconazole(1)
(c) 403. Rosetta 2000: Expression in response to Lovastatin(1)
(c) 406. Rosetta 2000: Expression in response to Terbinafine(1)
(c) 429. Expression in strain YHE711 (wild type) in response to 30 min 50 nM treatment with rapamycin in YPD(1)
(c) 446. Expression in response to 0.1% MMS for 10 min(1)
(c) 447. Expression in response to 0.1% MMS for 30 min(1)
(c) 448. Expression in response to 0.1% MMS for 60 min(1)
(c) 449. Expression in response to 0.1% MMS for 60 min(1)
(c) 479. Expression in diploid cells in response to rapamycin (100nM) for: 15min,30min,90min,120min(2)
(c) 503. Brown enviromental changes :37C to 25C shock - 15 min(1)
(c) 504. Brown enviromental changes :37C to 25C shock - 30 min(1)
(c) 506. Brown enviromental changes :37C to 25C shock - 60 min(1)
(c) 533. Brown enviromental changes :1 mM Menadione (10 min)redo(1)
(c) 570. Brown enviromental changes :1M sorbitol - 90 min(1)
(c) 635. Brown enviromental changes :YAP1 overexpression(1)
(c) MHY1 (crt1) vs CRY1 (wild type)
(c) 100 microM BCS 30 min

HAP3 -*-> TRP4

(c) 8. Expression during the cell cycle (cell size selection and release)(8)
(c) 89. Expression in response to 3-aminotriazole(1)
(c) 95. Expression in response to 50ug/mL FK506(1)
(c) 386. Rosetta 2000: Expression in cells with CDC42 under tet promoter(1)
(c) 387. Rosetta 2000: Expression in cells with ERG11 under tet promoter(1)
(c) 402. Rosetta 2000: Expression in response to Itraconazole(1)
(c) 403. Rosetta 2000: Expression in response to Lovastatin(1)
(c) 406. Rosetta 2000: Expression in response to Terbinafine(1)
(c) 429. Expression in strain YHE711 (wild type) in response to 30 min 50 nM treatment with rapamycin in YPD(1)
(c) 446. Expression in response to 0.1% MMS for 10 min(1)
(c) 447. Expression in response to 0.1% MMS for 30 min(1)
(c) 448. Expression in response to 0.1% MMS for 60 min(1)

(c) 449. Expression in response to 0.1% MMS for 60 min(1)
(c) 479. Expression in diploid cells in response to rapamycin (100nM) for: 15min,30min,90min,120min(2)
(c) 503. Brown enviromental changes :37C to 25C shock - 15 min(1)
(c) 504. Brown enviromental changes :37C to 25C shock - 30 min(1)
(c) 506. Brown enviromental changes :37C to 25C shock - 60 min(1)
(c) 533. Brown enviromental changes :1 mM Menadione (10 min)redo(1)
(c) 570. Brown enviromental changes :1M sorbitol - 90 min(1)
(c) 635. Brown enviromental changes :YAP1 overexpression(1)
(c) MHY1 (crt1) vs CRY1 (wild type)
(c) 100 microM BCS 30 min

HAP3 -*-> YHM1

(c) 8. Expression during the cell cycle (cell size selection and release)(8)
(c) 89. Expression in response to 3-aminotriazole(1)
(c) 95. Expression in response to 50ug/mL FK506(1)
(c) 386. Rosetta 2000: Expression in cells with CDC42 under tet promoter(1)
(c) 387. Rosetta 2000: Expression in cells with ERG11 under tet promoter(1)
(c) 402. Rosetta 2000: Expression in response to Itraconazole(1)
(c) 403. Rosetta 2000: Expression in response to Lovastatin(1)
(c) 406. Rosetta 2000: Expression in response to Terbinafine(1)
(c) 429. Expression in strain YHE711 (wild type) in response to 30 min 50 nM treatment with rapamycin in YPD(1)
(c) 446. Expression in response to 0.1% MMS for 10 min(1)
(c) 447. Expression in response to 0.1% MMS for 30 min(1)
(c) 448. Expression in response to 0.1% MMS for 60 min(1)
(c) 449. Expression in response to 0.1% MMS for 60 min(1)
(c) 479. Expression in diploid cells in response to rapamycin (100nM) for: 15min,30min,90min,120min(2)
(c) 503. Brown enviromental changes :37C to 25C shock - 15 min(1)
(c) 504. Brown enviromental changes :37C to 25C shock - 30 min(1)
(c) 506. Brown enviromental changes :37C to 25C shock - 60 min(1)
(c) 533. Brown enviromental changes :1 mM Menadione (10 min)redo(1)
(c) 570. Brown enviromental changes :1M sorbitol - 90 min(1)
(c) 635. Brown enviromental changes :YAP1 overexpression(1)
(c) MHY1 (crt1) vs CRY1 (wild type)
(c) 100 microM BCS 30 min

HAP4 -*-> ACH1

(c) 11. Expression during diauxic shift: 9h,11h,13h,15h,17h,19h,21h(4)
(c) 11. Expression during diauxic shift: 9h,11h,13h,15h,17h,19h,21h(5)
(c) 11. Expression during diauxic shift: 9h,11h,13h,15h,17h,19h,21h(6)
(c) 11. Expression during diauxic shift: 9h,11h,13h,15h,17h,19h,21h(7)
(c) 390. Rosetta 2000: Expression in cells with IDI1 under tet promoter(1)
(c) 481. Expression in response to heat shock: 15,30,45,60,120 min(1)
(c) 482. Expression in response to acid: 10,20,40,60,80,100 min(1)
(c) 485. Expression in response to peroxide: 10,20,40,60,120 min(1)
(c) 485. Expression in response to peroxide: 10,20,40,60,120 min(2)
(c) 485. Expression in response to peroxide: 10,20,40,60,120 min(3)
(c) 485. Expression in response to peroxide: 10,20,40,60,120 min(4)
(c) 485. Expression in response to peroxide: 10,20,40,60,120 min(5)
(c) 517. Brown enviromental changes :29C +1M sorbitol to 33C + 1M sorbitol - 5 minutes(1)
(c) 596. Brown enviromental changes :diauxic shift timecourse(1)
(c) 597. Brown enviromental changes :diauxic shift timecourse(1)
(c) 598. Brown enviromental changes :diauxic shift timecourse(1)
(c) 599. Brown enviromental changes :diauxic shift timecourse(1)
(c) 600. Brown enviromental changes :diauxic shift timecourse(1)
(c) 602. Brown enviromental changes :YPD 4 h ypd-2(1)
(c) 603. Brown enviromental changes :YPD 6 h ypd-2(1)
(c) 604. Brown enviromental changes :YPD 8 h ypd-2(1)
(c) 605. Brown enviromental changes :YPD 10 h ypd-2(1)
(c) 606. Brown enviromental changes :YPD 12 h ypd-2(1)
(c) 613. Brown enviromental changes :YPD stationary phase 8 h ypd-1(1)
(c) 614. Brown enviromental changes :YPD stationary phase 12 h ypd-1(1)
(c) 615. Brown enviromental changes :YPD stationary phase 1 d ypd-1(1)
(c) 616. Brown enviromental changes :YPD stationary phase 2 d ypd-1(1)
(c) 683. Expression in response to 0.4M NaCl for 20 min in wild type(1)

HAP4 -*-> ATP1

(c) 11. Expression during diauxic shift: 9h,11h,13h,15h,17h,19h,21h(4)
(c) 11. Expression during diauxic shift: 9h,11h,13h,15h,17h,19h,21h(5)
(c) 11. Expression during diauxic shift: 9h,11h,13h,15h,17h,19h,21h(6)
(c) 11. Expression during diauxic shift: 9h,11h,13h,15h,17h,19h,21h(7)
(c) 390. Rosetta 2000: Expression in cells with IDI1 under tet promoter(1)
(c) 481. Expression in response to heat shock: 15,30,45,60,120 min(1)
(c) 482. Expression in response to acid: 10,20,40,60,80,100 min(1)
(c) 485. Expression in response to peroxide: 10,20,40,60,120 min(1)
(c) 485. Expression in response to peroxide: 10,20,40,60,120 min(2)
(c) 485. Expression in response to peroxide: 10,20,40,60,120 min(3)
(c) 485. Expression in response to peroxide: 10,20,40,60,120 min(4)
(c) 485. Expression in response to peroxide: 10,20,40,60,120 min(5)
(c) 517. Brown enviromental changes :29C +1M sorbitol to 33C + 1M sorbitol - 5 minutes(1)
(c) 596. Brown enviromental changes :diauxic shift timecourse(1)
(c) 597. Brown enviromental changes :diauxic shift timecourse(1)
(c) 598. Brown enviromental changes :diauxic shift timecourse(1)
(c) 599. Brown enviromental changes :diauxic shift timecourse(1)
(c) 600. Brown enviromental changes :diauxic shift timecourse(1)
(c) 602. Brown enviromental changes :YPD 4 h ypd-2(1)
(c) 603. Brown enviromental changes :YPD 6 h ypd-2(1)
(c) 604. Brown enviromental changes :YPD 8 h ypd-2(1)
(c) 605. Brown enviromental changes :YPD 10 h ypd-2(1)
(c) 606. Brown enviromental changes :YPD 12 h ypd-2(1)
(c) 613. Brown enviromental changes :YPD stationary phase 8 h ypd-1(1)
(c) 614. Brown enviromental changes :YPD stationary phase 12 h ypd-1(1)
(c) 615. Brown enviromental changes :YPD stationary phase 1 d ypd-1(1)
(c) 616. Brown enviromental changes :YPD stationary phase 2 d ypd-1(1)
(c) 683. Expression in response to 0.4M NaCl for 20 min in wild type(1)

HAP4 -*-> NDI1

(c) 11. Expression during diauxic shift: 9h,11h,13h,15h,17h,19h,21h(4)
(c) 11. Expression during diauxic shift: 9h,11h,13h,15h,17h,19h,21h(5)
(c) 11. Expression during diauxic shift: 9h,11h,13h,15h,17h,19h,21h(6)
(c) 11. Expression during diauxic shift: 9h,11h,13h,15h,17h,19h,21h(7)

(c) 390. Rosetta 2000: Expression in cells with IDI1 under tet promoter(1)
(c) 481. Expression in response to heat shock: 15,30,45,60,120 min(1)
(c) 482. Expression in response to acid: 10,20,40,60,80,100 min(1)
(c) 485. Expression in response to peroxide: 10,20,40,60,120 min(1)
(c) 485. Expression in response to peroxide: 10,20,40,60,120 min(2)
(c) 485. Expression in response to peroxide: 10,20,40,60,120 min(3)
(c) 485. Expression in response to peroxide: 10,20,40,60,120 min(4)
(c) 485. Expression in response to peroxide: 10,20,40,60,120 min(5)
(c) 517. Brown enviromental changes :29C +1M sorbitol to 33C + 1M sorbitol - 5 minutes(1)
(c) 596. Brown enviromental changes :diauxic shift timecourse(1)
(c) 597. Brown enviromental changes :diauxic shift timecourse(1)
(c) 598. Brown enviromental changes :diauxic shift timecourse(1)
(c) 599. Brown enviromental changes :diauxic shift timecourse(1)
(c) 600. Brown enviromental changes :diauxic shift timecourse(1)
(c) 602. Brown enviromental changes :YPD 4 h ypd-2(1)
(c) 603. Brown enviromental changes :YPD 6 h ypd-2(1)
(c) 604. Brown enviromental changes :YPD 8 h ypd-2(1)
(c) 605. Brown enviromental changes :YPD 10 h ypd-2(1)
(c) 606. Brown enviromental changes :YPD 12 h ypd-2(1)
(c) 613. Brown enviromental changes :YPD stationary phase 8 h ypd-1(1)
(c) 614. Brown enviromental changes :YPD stationary phase 12 h ypd-1(1)
(c) 615. Brown enviromental changes :YPD stationary phase 1 d ypd-1(1)
(c) 616. Brown enviromental changes :YPD stationary phase 2 d ypd-1(1)
(c) 683. Expression in response to 0.4M NaCl for 20 min in wild type(1)

HAP4 -*-> SDH1

(c) 11. Expression during diauxic shift: 9h,11h,13h,15h,17h,19h,21h(4)
(c) 11. Expression during diauxic shift: 9h,11h,13h,15h,17h,19h,21h(5)
(c) 11. Expression during diauxic shift: 9h,11h,13h,15h,17h,19h,21h(6)
(c) 11. Expression during diauxic shift: 9h,11h,13h,15h,17h,19h,21h(7)
(c) 390. Rosetta 2000: Expression in cells with IDI1 under tet promoter(1)
(c) 481. Expression in response to heat shock: 15,30,45,60,120 min(1)
(c) 482. Expression in response to acid: 10,20,40,60,80,100 min(1)
(c) 485. Expression in response to peroxide: 10,20,40,60,120 min(1)
(c) 485. Expression in response to peroxide: 10,20,40,60,120 min(2)
(c) 485. Expression in response to peroxide: 10,20,40,60,120 min(3)
(c) 485. Expression in response to peroxide: 10,20,40,60,120 min(4)
(c) 485. Expression in response to peroxide: 10,20,40,60,120 min(5)
(c) 517. Brown enviromental changes :29C +1M sorbitol to 33C + 1M sorbitol - 5 minutes(1)
(c) 596. Brown enviromental changes :diauxic shift timecourse(1)
(c) 597. Brown enviromental changes :diauxic shift timecourse(1)
(c) 598. Brown enviromental changes :diauxic shift timecourse(1)
(c) 599. Brown enviromental changes :diauxic shift timecourse(1)
(c) 600. Brown enviromental changes :diauxic shift timecourse(1)
(c) 602. Brown enviromental changes :YPD 4 h ypd-2(1)
(c) 603. Brown enviromental changes :YPD 6 h ypd-2(1)
(c) 604. Brown enviromental changes :YPD 8 h ypd-2(1)
(c) 605. Brown enviromental changes :YPD 10 h ypd-2(1)
(c) 606. Brown enviromental changes :YPD 12 h ypd-2(1)
(c) 613. Brown enviromental changes :YPD stationary phase 8 h ypd-1(1)
(c) 614. Brown enviromental changes :YPD stationary phase 12 h ypd-1(1)
(c) 615. Brown enviromental changes :YPD stationary phase 1 d ypd-1(1)
(c) 616. Brown enviromental changes :YPD stationary phase 2 d ypd-1(1)
(c) 683. Expression in response to 0.4M NaCl for 20 min in wild type(1)

HAP4 -*-> SDH2

(c) 11. Expression during diauxic shift: 9h,11h,13h,15h,17h,19h,21h(4)
(c) 11. Expression during diauxic shift: 9h,11h,13h,15h,17h,19h,21h(5)
(c) 11. Expression during diauxic shift: 9h,11h,13h,15h,17h,19h,21h(6)
(c) 11. Expression during diauxic shift: 9h,11h,13h,15h,17h,19h,21h(7)
(c) 390. Rosetta 2000: Expression in cells with IDI1 under tet promoter(1)
(c) 481. Expression in response to heat shock: 15,30,45,60,120 min(1)
(c) 482. Expression in response to acid: 10,20,40,60,80,100 min(1)
(c) 485. Expression in response to peroxide: 10,20,40,60,120 min(1)
(c) 485. Expression in response to peroxide: 10,20,40,60,120 min(2)
(c) 485. Expression in response to peroxide: 10,20,40,60,120 min(3)
(c) 485. Expression in response to peroxide: 10,20,40,60,120 min(4)
(c) 485. Expression in response to peroxide: 10,20,40,60,120 min(5)
(c) 517. Brown enviromental changes :29C +1M sorbitol to 33C + 1M sorbitol - 5 minutes(1)
(c) 596. Brown enviromental changes :diauxic shift timecourse(1)
(c) 597. Brown enviromental changes :diauxic shift timecourse(1)
(c) 598. Brown enviromental changes :diauxic shift timecourse(1)
(c) 599. Brown enviromental changes :diauxic shift timecourse(1)
(c) 600. Brown enviromental changes :diauxic shift timecourse(1)
(c) 602. Brown enviromental changes :YPD 4 h ypd-2(1)
(c) 603. Brown enviromental changes :YPD 6 h ypd-2(1)
(c) 604. Brown enviromental changes :YPD 8 h ypd-2(1)
(c) 605. Brown enviromental changes :YPD 10 h ypd-2(1)
(c) 606. Brown enviromental changes :YPD 12 h ypd-2(1)
(c) 613. Brown enviromental changes :YPD stationary phase 8 h ypd-1(1)
(c) 614. Brown enviromental changes :YPD stationary phase 12 h ypd-1(1)
(c) 615. Brown enviromental changes :YPD stationary phase 1 d ypd-1(1)
(c) 616. Brown enviromental changes :YPD stationary phase 2 d ypd-1(1)
(c) 683. Expression in response to 0.4M NaCl for 20 min in wild type(1)

HSF1 -*-> CPR6

(c) 6. Expression during the cell cycle (cdc15 arrest and release)(23)
(c) 6. Expression during the cell cycle (cdc15 arrest and release)(24)
(c) 481. Expression in response to heat shock: 15,30,45,60,120 min(1)
(c) 481. Expression in response to heat shock: 15,30,45,60,120 min(2)
(c) 488. Brown enviromental changes :Heat Shock 05 minutes hs-1(1)
(c) 490. Brown enviromental changes :Heat Shock 15 minutes hs-1(1)
(c) 491. Brown enviromental changes :Heat Shock 20 minutes hs-1(1)
(c) 492. Brown enviromental changes :Heat Shock 30 minutes hs-1(1)
(c) 493. Brown enviromental changes :Heat Shock 40 minutes hs-1(1)
(c) 494. Brown enviromental changes :Heat Shock 60 minutes hs-1(1)
(c) 495. Brown enviromental changes :Heat Shock 80 minutes hs-1(1)
(c) 508. Brown enviromental changes :heat shock 17 to 37, 20 minutes(1)
(c) 509. Brown enviromental changes :heat shock 21 to 37, 20 minutes(1)
(c) 510. Brown enviromental changes :heat shock 25 to 37, 20 minutes(1)
(c) 511. Brown enviromental changes :heat shock 29 to 37, 20 minutes(1)
(c) 512. Brown enviromental changes :heat shock 33 to 37, 20 minutes(1)
(c) 514. Brown enviromental changes :29C to 33C - 15 minutes(1)

(c) 515. Brown enviromental changes :29C to 33C - 30 minutes(1)
(c) 517. Brown enviromental changes :29C +1M sorbitol to 33C + 1M sorbitol - 5 minutes(1)
(c) 518. Brown enviromental changes :29C +1M sorbitol to 33C + 1M sorbitol - 15 minutes(1)
(c) 558. Brown enviromental changes :1.5 mM diamide (10 min)(1)
(c) 559. Brown enviromental changes :1.5 mM diamide (20 min)(1)
(c) 561. Brown enviromental changes :1.5 mM diamide (40 min)(1)
(c) 562. Brown enviromental changes :1.5 mM diamide (50 min)(1)
(c) 563. Brown enviromental changes :1.5 mM diamide (60 min)(1)
(c) 564. Brown enviromental changes :1.5 mM diamide (90 min)(1)
(c) 568. Brown enviromental changes :1M sorbitol - 45 min (1)
(c) 623. Brown enviromental changes :DBY7286 37degree heat - 20 min(1)

HSF1 -*-> HSP10

(c) 6. Expression during the cell cycle (cdc15 arrest and release)(23)
(c) 6. Expression during the cell cycle (cdc15 arrest and release)(24)
(c) 481. Expression in response to heat shock: 15,30,45,60,120 min(1)
(c) 481. Expression in response to heat shock: 15,30,45,60,120 min(2)
(c) 488. Brown enviromental changes :Heat Shock 05 minutes hs-1(1)
(c) 490. Brown enviromental changes :Heat Shock 15 minutes hs-1(1)
(c) 491. Brown enviromental changes :Heat Shock 20 minutes hs-1(1)
(c) 492. Brown enviromental changes :Heat Shock 30 minutes hs-1(1)
(c) 493. Brown enviromental changes :Heat Shock 40 minutes hs-1(1)
(c) 494. Brown enviromental changes :Heat Shock 60 minutes hs-1(1)
(c) 495. Brown enviromental changes :Heat Shock 80 minutes hs-1(1)
(c) 508. Brown enviromental changes :heat shock 17 to 37, 20 minutes(1)
(c) 509. Brown enviromental changes :heat shock 21 to 37, 20 minutes(1)
(c) 510. Brown enviromental changes :heat shock 25 to 37, 20 minutes(1)
(c) 511. Brown enviromental changes :heat shock 29 to 37, 20 minutes(1)
(c) 512. Brown enviromental changes :heat shock 33 to 37, 20 minutes(1)
(c) 514. Brown enviromental changes :29C to 33C - 15 minutes(1)
(c) 515. Brown enviromental changes :29C to 33C - 30 minutes(1)
(c) 517. Brown enviromental changes :29C +1M sorbitol to 33C + 1M sorbitol - 5 minutes(1)
(c) 518. Brown enviromental changes :29C +1M sorbitol to 33C + 1M sorbitol - 15 minutes(1)
(c) 558. Brown enviromental changes :1.5 mM diamide (10 min)(1)
(c) 559. Brown enviromental changes :1.5 mM diamide (20 min)(1)
(c) 561. Brown enviromental changes :1.5 mM diamide (40 min)(1)
(c) 562. Brown enviromental changes :1.5 mM diamide (50 min)(1)
(c) 563. Brown enviromental changes :1.5 mM diamide (60 min)(1)
(c) 564. Brown enviromental changes :1.5 mM diamide (90 min)(1)
(c) 568. Brown enviromental changes :1M sorbitol - 45 min (1)
(c) 623. Brown enviromental changes :DBY7286 37degree heat - 20 min(1)

HSF1 -*-> HSP42

(c) 6. Expression during the cell cycle (cdc15 arrest and release)(23)
(c) 6. Expression during the cell cycle (cdc15 arrest and release)(24)
(c) 481. Expression in response to heat shock: 15,30,45,60,120 min(1)
(c) 481. Expression in response to heat shock: 15,30,45,60,120 min(2)
(c) 488. Brown enviromental changes :Heat Shock 05 minutes hs-1(1)
(c) 490. Brown enviromental changes :Heat Shock 15 minutes hs-1(1)
(c) 491. Brown enviromental changes :Heat Shock 20 minutes hs-1(1)
(c) 492. Brown enviromental changes :Heat Shock 30 minutes hs-1(1)
(c) 493. Brown enviromental changes :Heat Shock 40 minutes hs-1(1)
(c) 494. Brown enviromental changes :Heat Shock 60 minutes hs-1(1)
(c) 495. Brown enviromental changes :Heat Shock 80 minutes hs-1(1)
(c) 508. Brown enviromental changes :heat shock 17 to 37, 20 minutes(1)
(c) 509. Brown enviromental changes :heat shock 21 to 37, 20 minutes(1)
(c) 510. Brown enviromental changes :heat shock 25 to 37, 20 minutes(1)
(c) 511. Brown enviromental changes :heat shock 29 to 37, 20 minutes(1)
(c) 512. Brown enviromental changes :heat shock 33 to 37, 20 minutes(1)
(c) 514. Brown enviromental changes :29C to 33C - 15 minutes(1)
(c) 515. Brown enviromental changes :29C to 33C - 30 minutes(1)
(c) 517. Brown enviromental changes :29C +1M sorbitol to 33C + 1M sorbitol - 5 minutes(1)
(c) 518. Brown enviromental changes :29C +1M sorbitol to 33C + 1M sorbitol - 15 minutes(1)
(c) 558. Brown enviromental changes :1.5 mM diamide (10 min)(1)
(c) 559. Brown enviromental changes :1.5 mM diamide (20 min)(1)
(c) 561. Brown enviromental changes :1.5 mM diamide (40 min)(1)
(c) 562. Brown enviromental changes :1.5 mM diamide (50 min)(1)
(c) 563. Brown enviromental changes :1.5 mM diamide (60 min)(1)
(c) 564. Brown enviromental changes :1.5 mM diamide (90 min)(1)
(c) 568. Brown enviromental changes :1M sorbitol - 45 min (1)
(c) 623. Brown enviromental changes :DBY7286 37degree heat - 20 min(1)

HSF1 -*-> HSP60

(c) 6. Expression during the cell cycle (cdc15 arrest and release)(23)
(c) 6. Expression during the cell cycle (cdc15 arrest and release)(24)
(c) 481. Expression in response to heat shock: 15,30,45,60,120 min(1)
(c) 481. Expression in response to heat shock: 15,30,45,60,120 min(2)
(c) 488. Brown enviromental changes :Heat Shock 05 minutes hs-1(1)
(c) 490. Brown enviromental changes :Heat Shock 15 minutes hs-1(1)
(c) 491. Brown enviromental changes :Heat Shock 20 minutes hs-1(1)
(c) 492. Brown enviromental changes :Heat Shock 30 minutes hs-1(1)
(c) 493. Brown enviromental changes :Heat Shock 40 minutes hs-1(1)
(c) 494. Brown enviromental changes :Heat Shock 60 minutes hs-1(1)
(c) 495. Brown enviromental changes :Heat Shock 80 minutes hs-1(1)
(c) 508. Brown enviromental changes :heat shock 17 to 37, 20 minutes(1)
(c) 509. Brown enviromental changes :heat shock 21 to 37, 20 minutes(1)
(c) 510. Brown enviromental changes :heat shock 25 to 37, 20 minutes(1)
(c) 511. Brown enviromental changes :heat shock 29 to 37, 20 minutes(1)
(c) 512. Brown enviromental changes :heat shock 33 to 37, 20 minutes(1)
(c) 514. Brown enviromental changes :29C to 33C - 15 minutes(1)
(c) 515. Brown enviromental changes :29C to 33C - 30 minutes(1)
(c) 517. Brown enviromental changes :29C +1M sorbitol to 33C + 1M sorbitol - 5 minutes(1)
(c) 518. Brown enviromental changes :29C +1M sorbitol to 33C + 1M sorbitol - 15 minutes(1)
(c) 558. Brown enviromental changes :1.5 mM diamide (10 min)(1)
(c) 559. Brown enviromental changes :1.5 mM diamide (20 min)(1)
(c) 561. Brown enviromental changes :1.5 mM diamide (40 min)(1)
(c) 562. Brown enviromental changes :1.5 mM diamide (50 min)(1)
(c) 563. Brown enviromental changes :1.5 mM diamide (60 min)(1)
(c) 564. Brown enviromental changes :1.5 mM diamide (90 min)(1)
(c) 568. Brown enviromental changes :1M sorbitol - 45 min (1)
(c) 623. Brown enviromental changes :DBY7286 37degree heat - 20 min(1)

HSF1 -*-> LAP4

(c) 6. Expression during the cell cycle (cdc15 arrest and release)(23)
(c) 6. Expression during the cell cycle (cdc15 arrest and release)(24)
(c) 481. Expression in response to heat shock: 15,30,45,60,120 min(1)
(c) 481. Expression in response to heat shock: 15,30,45,60,120 min(2)
(c) 488. Brown enviromental changes :Heat Shock 05 minutes hs-1(1)
(c) 490. Brown enviromental changes :Heat Shock 15 minutes hs-1(1)
(c) 491. Brown enviromental changes :Heat Shock 20 minutes hs-1(1)
(c) 492. Brown enviromental changes :Heat Shock 30 minutes hs-1(1)
(c) 493. Brown enviromental changes :Heat Shock 40 minutes hs-1(1)
(c) 494. Brown enviromental changes :Heat Shock 60 minutes hs-1(1)
(c) 495. Brown enviromental changes :Heat Shock 80 minutes hs-1(1)
(c) 508. Brown enviromental changes :heat shock 17 to 37, 20 minutes(1)
(c) 509. Brown enviromental changes :heat shock 21 to 37, 20 minutes(1)
(c) 510. Brown enviromental changes :heat shock 25 to 37, 20 minutes(1)
(c) 511. Brown enviromental changes :heat shock 29 to 37, 20 minutes(1)
(c) 512. Brown enviromental changes :heat shock 33 to 37, 20 minutes(1)
(c) 514. Brown enviromental changes :29C to 33C - 15 minutes(1)
(c) 515. Brown enviromental changes :29C to 33C - 30 minutes(1)
(c) 517. Brown enviromental changes :29C +1M sorbitol to 33C + 1M sorbitol - 5 minutes(1)
(c) 518. Brown enviromental changes :29C +1M sorbitol to 33C + 1M sorbitol - 15 minutes(1)
(c) 558. Brown enviromental changes :1.5 mM diamide (10 min)(1)
(c) 559. Brown enviromental changes :1.5 mM diamide (20 min)(1)
(c) 561. Brown enviromental changes :1.5 mM diamide (40 min)(1)
(c) 562. Brown enviromental changes :1.5 mM diamide (50 min)(1)
(c) 563. Brown enviromental changes :1.5 mM diamide (60 min)(1)
(c) 564. Brown enviromental changes :1.5 mM diamide (90 min)(1)
(c) 568. Brown enviromental changes :1M sorbitol - 45 min (1)
(c) 623. Brown enviromental changes :DBY7286 37degree heat - 20 min(1)

HSF1 -*-> SSA4

(c) 6. Expression during the cell cycle (cdc15 arrest and release)(23)
(c) 6. Expression during the cell cycle (cdc15 arrest and release)(24)
(c) 481. Expression in response to heat shock: 15,30,45,60,120 min(1)
(c) 481. Expression in response to heat shock: 15,30,45,60,120 min(2)
(c) 488. Brown enviromental changes :Heat Shock 05 minutes hs-1(1)
(c) 490. Brown enviromental changes :Heat Shock 15 minutes hs-1(1)
(c) 491. Brown enviromental changes :Heat Shock 20 minutes hs-1(1)
(c) 492. Brown enviromental changes :Heat Shock 30 minutes hs-1(1)
(c) 493. Brown enviromental changes :Heat Shock 40 minutes hs-1(1)
(c) 494. Brown enviromental changes :Heat Shock 60 minutes hs-1(1)
(c) 495. Brown enviromental changes :Heat Shock 80 minutes hs-1(1)
(c) 508. Brown enviromental changes :heat shock 17 to 37, 20 minutes(1)
(c) 509. Brown enviromental changes :heat shock 21 to 37, 20 minutes(1)
(c) 510. Brown enviromental changes :heat shock 25 to 37, 20 minutes(1)
(c) 511. Brown enviromental changes :heat shock 29 to 37, 20 minutes(1)
(c) 512. Brown enviromental changes :heat shock 33 to 37, 20 minutes(1)
(c) 514. Brown enviromental changes :29C to 33C - 15 minutes(1)
(c) 515. Brown enviromental changes :29C to 33C - 30 minutes(1)
(c) 517. Brown enviromental changes :29C +1M sorbitol to 33C + 1M sorbitol - 5 minutes(1)
(c) 518. Brown enviromental changes :29C +1M sorbitol to 33C + 1M sorbitol - 15 minutes(1)
(c) 558. Brown enviromental changes :1.5 mM diamide (10 min)(1)
(c) 559. Brown enviromental changes :1.5 mM diamide (20 min)(1)
(c) 561. Brown enviromental changes :1.5 mM diamide (40 min)(1)
(c) 562. Brown enviromental changes :1.5 mM diamide (50 min)(1)
(c) 563. Brown enviromental changes :1.5 mM diamide (60 min)(1)
(c) 564. Brown enviromental changes :1.5 mM diamide (90 min)(1)
(c) 568. Brown enviromental changes :1M sorbitol - 45 min (1)
(c) 623. Brown enviromental changes :DBY7286 37degree heat - 20 min(1)

HSF1 -*-> SSE2

(c) 6. Expression during the cell cycle (cdc15 arrest and release)(23)
(c) 6. Expression during the cell cycle (cdc15 arrest and release)(24)
(c) 481. Expression in response to heat shock: 15,30,45,60,120 min(1)
(c) 481. Expression in response to heat shock: 15,30,45,60,120 min(2)
(c) 488. Brown enviromental changes :Heat Shock 05 minutes hs-1(1)
(c) 490. Brown enviromental changes :Heat Shock 15 minutes hs-1(1)
(c) 491. Brown enviromental changes :Heat Shock 20 minutes hs-1(1)
(c) 492. Brown enviromental changes :Heat Shock 30 minutes hs-1(1)
(c) 493. Brown enviromental changes :Heat Shock 40 minutes hs-1(1)
(c) 494. Brown enviromental changes :Heat Shock 60 minutes hs-1(1)
(c) 495. Brown enviromental changes :Heat Shock 80 minutes hs-1(1)
(c) 508. Brown enviromental changes :heat shock 17 to 37, 20 minutes(1)
(c) 509. Brown enviromental changes :heat shock 21 to 37, 20 minutes(1)
(c) 510. Brown enviromental changes :heat shock 25 to 37, 20 minutes(1)
(c) 511. Brown enviromental changes :heat shock 29 to 37, 20 minutes(1)
(c) 512. Brown enviromental changes :heat shock 33 to 37, 20 minutes(1)
(c) 514. Brown enviromental changes :29C to 33C - 15 minutes(1)
(c) 515. Brown enviromental changes :29C to 33C - 30 minutes(1)
(c) 517. Brown enviromental changes :29C +1M sorbitol to 33C + 1M sorbitol - 5 minutes(1)
(c) 518. Brown enviromental changes :29C +1M sorbitol to 33C + 1M sorbitol - 15 minutes(1)
(c) 558. Brown enviromental changes :1.5 mM diamide (10 min)(1)
(c) 559. Brown enviromental changes :1.5 mM diamide (20 min)(1)
(c) 561. Brown enviromental changes :1.5 mM diamide (40 min)(1)
(c) 562. Brown enviromental changes :1.5 mM diamide (50 min)(1)
(c) 563. Brown enviromental changes :1.5 mM diamide (60 min)(1)
(c) 564. Brown enviromental changes :1.5 mM diamide (90 min)(1)
(c) 568. Brown enviromental changes :1M sorbitol - 45 min (1)
(c) 623. Brown enviromental changes :DBY7286 37degree heat - 20 min(1)

HSF1 -*-> STI1

(c) 6. Expression during the cell cycle (cdc15 arrest and release)(23)
(c) 6. Expression during the cell cycle (cdc15 arrest and release)(24)
(c) 481. Expression in response to heat shock: 15,30,45,60,120 min(1)
(c) 481. Expression in response to heat shock: 15,30,45,60,120 min(2)
(c) 488. Brown enviromental changes :Heat Shock 05 minutes hs-1(1)
(c) 490. Brown enviromental changes :Heat Shock 15 minutes hs-1(1)
(c) 491. Brown enviromental changes :Heat Shock 20 minutes hs-1(1)
(c) 492. Brown enviromental changes :Heat Shock 30 minutes hs-1(1)
(c) 493. Brown enviromental changes :Heat Shock 40 minutes hs-1(1)
(c) 494. Brown enviromental changes :Heat Shock 60 minutes hs-1(1)

```
    (c) 495. Brown enviromental changes :Heat Shock 80 minutes hs-1(1)
    (c) 508. Brown enviromental changes :heat shock 17 to 37, 20 minutes(1)
    (c) 509. Brown enviromental changes :heat shock 21 to 37, 20 minutes(1)
    (c) 510. Brown enviromental changes :heat shock 25 to 37, 20 minutes(1)
    (c) 511. Brown enviromental changes :heat shock 29 to 37, 20 minutes(1)
    (c) 512. Brown enviromental changes :heat shock 33 to 37, 20 minutes(1)
    (c) 514. Brown enviromental changes :29C to 33C - 15 minutes(1)
    (c) 515. Brown enviromental changes :29C to 33C - 30 minutes(1)
    (c) 517. Brown enviromental changes :29C +1M sorbitol to 33C + 1M sorbitol - 5 minutes(1)
    (c) 518. Brown enviromental changes :29C +1M sorbitol to 33C + 1M sorbitol - 15 minutes(1)
    (c) 558. Brown enviromental changes :1.5 mM diamide (10 min)(1)
    (c) 559. Brown enviromental changes :1.5 mM diamide (20 min)(1)
    (c) 561. Brown enviromental changes :1.5 mM diamide (40 min)(1)
    (c) 562. Brown enviromental changes :1.5 mM diamide (50 min)(1)
    (c) 563. Brown enviromental changes :1.5 mM diamide (60 min)(1)
    (c) 564. Brown enviromental changes :1.5 mM diamide (90 min)(1)
    (c) 568. Brown enviromental changes :1M sorbitol - 45 min (1)
    (c) 623. Brown enviromental changes :DBY7286 37degree heat - 20 min(1)


HSF1 -*-> TSL1


    (c) 6. Expression during the cell cycle (cdc15 arrest and release)(23)
    (c) 6. Expression during the cell cycle (cdc15 arrest and release)(24)
    (c) 481. Expression in response to heat shock: 15,30,45,60,120 min(1)
    (c) 481. Expression in response to heat shock: 15,30,45,60,120 min(2)
    (c) 488. Brown enviromental changes :Heat Shock 05 minutes hs-1(1)
    (c) 490. Brown enviromental changes :Heat Shock 15 minutes hs-1(1)
    (c) 491. Brown enviromental changes :Heat Shock 20 minutes hs-1(1)
    (c) 492. Brown enviromental changes :Heat Shock 30 minutes hs-1(1)
    (c) 493. Brown enviromental changes :Heat Shock 40 minutes hs-1(1)
    (c) 494. Brown enviromental changes :Heat Shock 60 minutes hs-1(1)
    (c) 495. Brown enviromental changes :Heat Shock 80 minutes hs-1(1)
    (c) 508. Brown enviromental changes :heat shock 17 to 37, 20 minutes(1)
    (c) 509. Brown enviromental changes :heat shock 21 to 37, 20 minutes(1)
    (c) 510. Brown enviromental changes :heat shock 25 to 37, 20 minutes(1)
    (c) 511. Brown enviromental changes :heat shock 29 to 37, 20 minutes(1)
    (c) 512. Brown enviromental changes :heat shock 33 to 37, 20 minutes(1)
    (c) 514. Brown enviromental changes :29C to 33C - 15 minutes(1)
    (c) 515. Brown enviromental changes :29C to 33C - 30 minutes(1)
    (c) 517. Brown enviromental changes :29C +1M sorbitol to 33C + 1M sorbitol - 5 minutes(1)
    (c) 518. Brown enviromental changes :29C +1M sorbitol to 33C + 1M sorbitol - 15 minutes(1)
    (c) 558. Brown enviromental changes :1.5 mM diamide (10 min)(1)
    (c) 559. Brown enviromental changes :1.5 mM diamide (20 min)(1)
    (c) 561. Brown enviromental changes :1.5 mM diamide (40 min)(1)
    (c) 562. Brown enviromental changes :1.5 mM diamide (50 min)(1)
    (c) 563. Brown enviromental changes :1.5 mM diamide (60 min)(1)
    (c) 564. Brown enviromental changes :1.5 mM diamide (90 min)(1)
    (c) 568. Brown enviromental changes :1M sorbitol - 45 min (1)
    (c) 623. Brown enviromental changes :DBY7286 37degree heat - 20 min(1)


HSF1 -*-> YDR214W


    (c) 6. Expression during the cell cycle (cdc15 arrest and release)(23)
    (c) 6. Expression during the cell cycle (cdc15 arrest and release)(24)
    (c) 481. Expression in response to heat shock: 15,30,45,60,120 min(1)
    (c) 481. Expression in response to heat shock: 15,30,45,60,120 min(2)
    (c) 488. Brown enviromental changes :Heat Shock 05 minutes hs-1(1)
    (c) 490. Brown enviromental changes :Heat Shock 15 minutes hs-1(1)
    (c) 491. Brown enviromental changes :Heat Shock 20 minutes hs-1(1)
    (c) 492. Brown enviromental changes :Heat Shock 30 minutes hs-1(1)
    (c) 493. Brown enviromental changes :Heat Shock 40 minutes hs-1(1)
    (c) 494. Brown enviromental changes :Heat Shock 60 minutes hs-1(1)
    (c) 495. Brown enviromental changes :Heat Shock 80 minutes hs-1(1)
    (c) 508. Brown enviromental changes :heat shock 17 to 37, 20 minutes(1)
    (c) 509. Brown enviromental changes :heat shock 21 to 37, 20 minutes(1)
    (c) 510. Brown enviromental changes :heat shock 25 to 37, 20 minutes(1)
    (c) 511. Brown enviromental changes :heat shock 29 to 37, 20 minutes(1)
    (c) 512. Brown enviromental changes :heat shock 33 to 37, 20 minutes(1)
    (c) 514. Brown enviromental changes :29C to 33C - 15 minutes(1)
    (c) 515. Brown enviromental changes :29C to 33C - 30 minutes(1)
    (c) 517. Brown enviromental changes :29C +1M sorbitol to 33C + 1M sorbitol - 5 minutes(1)
    (c) 518. Brown enviromental changes :29C +1M sorbitol to 33C + 1M sorbitol - 15 minutes(1)
    (c) 558. Brown enviromental changes :1.5 mM diamide (10 min)(1)
    (c) 559. Brown enviromental changes :1.5 mM diamide (20 min)(1)
    (c) 561. Brown enviromental changes :1.5 mM diamide (40 min)(1)
    (c) 562. Brown enviromental changes :1.5 mM diamide (50 min)(1)
    (c) 563. Brown enviromental changes :1.5 mM diamide (60 min)(1)
    (c) 564. Brown enviromental changes :1.5 mM diamide (90 min)(1)
    (c) 568. Brown enviromental changes :1M sorbitol - 45 min (1)
    (c) 623. Brown enviromental changes :DBY7286 37degree heat - 20 min(1)


LEU3 -*-> BAT1


    (c) 7. Expression during the cell Cycle (cdc28)(16)
    (c) 8. Expression during the cell cycle (cell size selection and release)(8)
    (c) 8. Expression during the cell cycle (cell size selection and release)(9)
    (c) 8. Expression during the cell cycle (cell size selection and release)(10)
    (c) 8. Expression during the cell cycle (cell size selection and release)(14)
    (c) 11. Expression during diauxic shift: 9h,11h,13h,15h,17h,19h,21h(1)
    (c) 89. Expression in response to 3-aminotriazole(1)
    (c) 95. Expression in response to 50ug/mL FK506(1)
    (c) 332. Rosetta 2000: Expression in cells with CMD1 under tet promoter(1)
    (c) 387. Rosetta 2000: Expression in cells with ERG11 under tet promoter(1)
    (c) 389. Rosetta 2000: Expression in cells with HMG2 under tet promoter(1)
    (c) 393. Rosetta 2000: Expression in cells with RHO1 under tet promoter(1)
    (c) 395. Rosetta 2000: Expression in response to 2-deoxy-D-glucose(1)
    (c) 402. Rosetta 2000: Expression in response to Itraconazole(1)
    (c) 403. Rosetta 2000: Expression in response to Lovastatin(1)
    (c) 406. Rosetta 2000: Expression in response to Terbinafine(1)
    (c) 407. Rosetta 2000: Expression in response to Tunicamycin(1)
    (c) 446. Expression in response to 0.1% MMS for 10 min(1)
    (c) 447. Expression in response to 0.1% MMS for 30 min(1)
    (c) 456. Expression in response to high 4NQO (8 microgram/ml) for 60 min(1)
    (c) 504. Brown enviromental changes :37C to 25C shock - 30 min(1)
    (c) 585. Brown enviromental changes :Nitrogen Depletion 2 h(1)
    (c) 586. Brown enviromental changes :Nitrogen Depletion 4 h(1)
```

(c) 593. Brown enviromental changes :Diauxic Shift Timecourse(1)
(c) 611. Brown enviromental changes :YPD stationary phase 2 h ypd-1(1)
(c) 612. Brown enviromental changes :YPD stationary phase 4 h ypd-1(1)
(c) 684. Expression in response to 0.8M NaCl for 10 min in wild type(1)
(c) DES460 (wt) - mock irradiation - 30 min
(c) 100 microM BCS 30 min

MAC1 -*-| TPO4

(c) 5. Expression during the cell cycle (alpha factor arrest and release)(11)
(c) 5. Expression during the cell cycle (alpha factor arrest and release)(15)
(c) 8. Expression during the cell cycle (cell size selection and release)(11)
(c) 8. Expression during the cell cycle (cell size selection and release)(14)
(c) 447. Expression in response to 0.1% MMS for 30 min(1)
(c) 448. Expression in response to 0.1% MMS for 60 min(1)
(c) 451. Expression in response to BCNU (200 micromolar) for 60 min(1)
(c) 452. Expression in response to low 4NQO (2 microgram/ml) for 60 min(1)
(c) 456. Expression in response to high 4NQO (8 microgram/ml) for 60 min(1)
(c) 516. Brown enviromental changes :33C vs. 30C - 90 minutes(1)
(c) 531. Brown enviromental changes :constant 0.32 mM H2O2 (120 min) redo(1)
(c) MHY1 (crt1) vs. CRY1 (wild type) - log phase
(c) 100 microM BCS 30 min
(c) 100 microM BCS 60 min
(c) MAC1-up (B)
(c) MAC1-up (C)

MCM1 -*-> BUD4

(c) 3. Cell Cycle: Expression in response to Clb2p (set 1, 40 min)(1)
(c) 4. Cell Cycle: Expression in response to Clb2p (set 2, 30 min)(1)
(c) 5. Expression during the cell cycle (alpha factor arrest and release)(7)
(c) 5. Expression during the cell cycle (alpha factor arrest and release)(8)
(c) 5. Expression during the cell cycle (alpha factor arrest and release)(9)
(c) 5. Expression during the cell cycle (alpha factor arrest and release)(10)
(c) 5. Expression during the cell cycle (alpha factor arrest and release)(16)
(c) 5. Expression during the cell cycle (alpha factor arrest and release)(17)
(c) 5. Expression during the cell cycle (alpha factor arrest and release)(18)
(c) 6. Expression during the cell cycle (cdc15 arrest and release)(5)
(c) 6. Expression during the cell cycle (cdc15 arrest and release)(7)
(c) 6. Expression during the cell cycle (cdc15 arrest and release)(8)
(c) 6. Expression during the cell cycle (cdc15 arrest and release)(16)
(c) 6. Expression during the cell cycle (cdc15 arrest and release)(18)
(c) 6. Expression during the cell cycle (cdc15 arrest and release)(19)
(c) 6. Expression during the cell cycle (cdc15 arrest and release)(20)
(c) 7. Expression during the cell Cycle (cdc28)(7)
(c) 7. Expression during the cell Cycle (cdc28)(8)
(c) 7. Expression during the cell Cycle (cdc28)(9)
(c) 7. Expression during the cell Cycle (cdc28)(16)
(c) 8. Expression during the cell cycle (cell size selection and release)(8)
(c) 8. Expression during the cell cycle (cell size selection and release)(9)
(c) 8. Expression during the cell cycle (cell size selection and release)(10)
(c) 8. Expression during the cell cycle (cell size selection and release)(11)
(c) 8. Expression during the cell cycle (cell size selection and release)(12)
(c) 8. Expression during the cell cycle (cell size selection and release)(13)
(c) 8. Expression during the cell cycle (cell size selection and release)(14)
(c) 556. Brown enviromental changes :dtt 480 min dtt-2(1)
(c) 570. Brown enviromental changes :1M sorbitol - 90 min(1)

MCM1 -*-> KIN3

(c) 3. Cell Cycle: Expression in response to Clb2p (set 1, 40 min)(1)
(c) 4. Cell Cycle: Expression in response to Clb2p (set 2, 30 min)(1)
(c) 5. Expression during the cell cycle (alpha factor arrest and release)(7)
(c) 5. Expression during the cell cycle (alpha factor arrest and release)(8)
(c) 5. Expression during the cell cycle (alpha factor arrest and release)(9)
(c) 5. Expression during the cell cycle (alpha factor arrest and release)(10)
(c) 5. Expression during the cell cycle (alpha factor arrest and release)(16)
(c) 5. Expression during the cell cycle (alpha factor arrest and release)(17)
(c) 5. Expression during the cell cycle (alpha factor arrest and release)(18)
(c) 6. Expression during the cell cycle (cdc15 arrest and release)(5)
(c) 6. Expression during the cell cycle (cdc15 arrest and release)(7)
(c) 6. Expression during the cell cycle (cdc15 arrest and release)(8)
(c) 6. Expression during the cell cycle (cdc15 arrest and release)(16)
(c) 6. Expression during the cell cycle (cdc15 arrest and release)(18)
(c) 6. Expression during the cell cycle (cdc15 arrest and release)(19)
(c) 6. Expression during the cell cycle (cdc15 arrest and release)(20)
(c) 7. Expression during the cell Cycle (cdc28)(7)
(c) 7. Expression during the cell Cycle (cdc28)(8)
(c) 7. Expression during the cell Cycle (cdc28)(9)
(c) 7. Expression during the cell Cycle (cdc28)(16)
(c) 8. Expression during the cell cycle (cell size selection and release)(8)
(c) 8. Expression during the cell cycle (cell size selection and release)(9)
(c) 8. Expression during the cell cycle (cell size selection and release)(10)
(c) 8. Expression during the cell cycle (cell size selection and release)(11)
(c) 8. Expression during the cell cycle (cell size selection and release)(12)
(c) 8. Expression during the cell cycle (cell size selection and release)(13)
(c) 8. Expression during the cell cycle (cell size selection and release)(14)
(c) 556. Brown enviromental changes :dtt 480 min dtt-2(1)
(c) 570. Brown enviromental changes :1M sorbitol - 90 min(1)

MCM1 -*-> PHO3

(c) 3. Cell Cycle: Expression in response to Clb2p (set 1, 40 min)(1)
(c) 4. Cell Cycle: Expression in response to Clb2p (set 2, 30 min)(1)
(c) 5. Expression during the cell cycle (alpha factor arrest and release)(7)
(c) 5. Expression during the cell cycle (alpha factor arrest and release)(8)
(c) 5. Expression during the cell cycle (alpha factor arrest and release)(9)
(c) 5. Expression during the cell cycle (alpha factor arrest and release)(10)
(c) 5. Expression during the cell cycle (alpha factor arrest and release)(16)
(c) 5. Expression during the cell cycle (alpha factor arrest and release)(17)
(c) 5. Expression during the cell cycle (alpha factor arrest and release)(18)
(c) 6. Expression during the cell cycle (cdc15 arrest and release)(5)
(c) 6. Expression during the cell cycle (cdc15 arrest and release)(7)
(c) 6. Expression during the cell cycle (cdc15 arrest and release)(8)

(c) 6. Expression during the cell cycle (cdc15 arrest and release)(16)
(c) 6. Expression during the cell cycle (cdc15 arrest and release)(18)
(c) 6. Expression during the cell cycle (cdc15 arrest and release)(19)
(c) 6. Expression during the cell cycle (cdc15 arrest and release)(20)
(c) 7. Expression during the cell Cycle (cdc28)(7)
(c) 7. Expression during the cell Cycle (cdc28)(8)
(c) 7. Expression during the cell Cycle (cdc28)(9)
(c) 7. Expression during the cell Cycle (cdc28)(16)
(c) 8. Expression during the cell cycle (cell size selection and release)(8)
(c) 8. Expression during the cell cycle (cell size selection and release)(9)
(c) 8. Expression during the cell cycle (cell size selection and release)(10)
(c) 8. Expression during the cell cycle (cell size selection and release)(11)
(c) 8. Expression during the cell cycle (cell size selection and release)(12)
(c) 8. Expression during the cell cycle (cell size selection and release)(13)
(c) 8. Expression during the cell cycle (cell size selection and release)(14)
(c) 556. Brown enviromental changes :dtt 480 min dtt-2(1)
(c) 570. Brown enviromental changes :1M sorbitol - 90 min(1)

MCM1 -*-> RAX2

(c) 3. Cell Cycle: Expression in response to Clb2p (set 1, 40 min)(1)
(c) 4. Cell Cycle: Expression in response to Clb2p (set 2, 30 min)(1)
(c) 5. Expression during the cell cycle (alpha factor arrest and release)(7)
(c) 5. Expression during the cell cycle (alpha factor arrest and release)(8)
(c) 5. Expression during the cell cycle (alpha factor arrest and release)(9)
(c) 5. Expression during the cell cycle (alpha factor arrest and release)(10)
(c) 5. Expression during the cell cycle (alpha factor arrest and release)(16)
(c) 5. Expression during the cell cycle (alpha factor arrest and release)(17)
(c) 5. Expression during the cell cycle (alpha factor arrest and release)(18)
(c) 6. Expression during the cell cycle (cdc15 arrest and release)(5)
(c) 6. Expression during the cell cycle (cdc15 arrest and release)(7)
(c) 6. Expression during the cell cycle (cdc15 arrest and release)(8)
(c) 6. Expression during the cell cycle (cdc15 arrest and release)(16)
(c) 6. Expression during the cell cycle (cdc15 arrest and release)(18)
(c) 6. Expression during the cell cycle (cdc15 arrest and release)(19)
(c) 6. Expression during the cell cycle (cdc15 arrest and release)(20)
(c) 7. Expression during the cell Cycle (cdc28)(7)
(c) 7. Expression during the cell Cycle (cdc28)(8)
(c) 7. Expression during the cell Cycle (cdc28)(9)
(c) 7. Expression during the cell Cycle (cdc28)(16)
(c) 8. Expression during the cell cycle (cell size selection and release)(8)
(c) 8. Expression during the cell cycle (cell size selection and release)(9)
(c) 8. Expression during the cell cycle (cell size selection and release)(10)
(c) 8. Expression during the cell cycle (cell size selection and release)(11)
(c) 8. Expression during the cell cycle (cell size selection and release)(12)
(c) 8. Expression during the cell cycle (cell size selection and release)(13)
(c) 8. Expression during the cell cycle (cell size selection and release)(14)
(c) 556. Brown enviromental changes :dtt 480 min dtt-2(1)
(c) 570. Brown enviromental changes :1M sorbitol - 90 min(1)

MCM1 -*-| YJL051W

(c) 3. Cell Cycle: Expression in response to Clb2p (set 1, 40 min)(1)
(c) 4. Cell Cycle: Expression in response to Clb2p (set 2, 30 min)(1)
(c) 5. Expression during the cell cycle (alpha factor arrest and release)(7)
(c) 5. Expression during the cell cycle (alpha factor arrest and release)(8)
(c) 5. Expression during the cell cycle (alpha factor arrest and release)(9)
(c) 5. Expression during the cell cycle (alpha factor arrest and release)(10)
(c) 5. Expression during the cell cycle (alpha factor arrest and release)(16)
(c) 5. Expression during the cell cycle (alpha factor arrest and release)(17)
(c) 5. Expression during the cell cycle (alpha factor arrest and release)(18)
(c) 6. Expression during the cell cycle (cdc15 arrest and release)(5)
(c) 6. Expression during the cell cycle (cdc15 arrest and release)(7)
(c) 6. Expression during the cell cycle (cdc15 arrest and release)(8)
(c) 6. Expression during the cell cycle (cdc15 arrest and release)(16)
(c) 6. Expression during the cell cycle (cdc15 arrest and release)(18)
(c) 6. Expression during the cell cycle (cdc15 arrest and release)(19)
(c) 6. Expression during the cell cycle (cdc15 arrest and release)(20)
(c) 7. Expression during the cell Cycle (cdc28)(7)
(c) 7. Expression during the cell Cycle (cdc28)(8)
(c) 7. Expression during the cell Cycle (cdc28)(9)
(c) 7. Expression during the cell Cycle (cdc28)(16)
(c) 8. Expression during the cell cycle (cell size selection and release)(8)
(c) 8. Expression during the cell cycle (cell size selection and release)(9)
(c) 8. Expression during the cell cycle (cell size selection and release)(10)
(c) 8. Expression during the cell cycle (cell size selection and release)(11)
(c) 8. Expression during the cell cycle (cell size selection and release)(12)
(c) 8. Expression during the cell cycle (cell size selection and release)(13)
(c) 8. Expression during the cell cycle (cell size selection and release)(14)
(c) 556. Brown enviromental changes :dtt 480 min dtt-2(1)
(c) 570. Brown enviromental changes :1M sorbitol - 90 min(1)

MCM1 -*-> YML119W

(c) 3. Cell Cycle: Expression in response to Clb2p (set 1, 40 min)(1)
(c) 4. Cell Cycle: Expression in response to Clb2p (set 2, 30 min)(1)
(c) 5. Expression during the cell cycle (alpha factor arrest and release)(7)
(c) 5. Expression during the cell cycle (alpha factor arrest and release)(8)
(c) 5. Expression during the cell cycle (alpha factor arrest and release)(9)
(c) 5. Expression during the cell cycle (alpha factor arrest and release)(10)
(c) 5. Expression during the cell cycle (alpha factor arrest and release)(16)
(c) 5. Expression during the cell cycle (alpha factor arrest and release)(17)
(c) 5. Expression during the cell cycle (alpha factor arrest and release)(18)
(c) 6. Expression during the cell cycle (cdc15 arrest and release)(5)
(c) 6. Expression during the cell cycle (cdc15 arrest and release)(7)
(c) 6. Expression during the cell cycle (cdc15 arrest and release)(8)
(c) 6. Expression during the cell cycle (cdc15 arrest and release)(16)
(c) 6. Expression during the cell cycle (cdc15 arrest and release)(18)
(c) 6. Expression during the cell cycle (cdc15 arrest and release)(19)
(c) 6. Expression during the cell cycle (cdc15 arrest and release)(20)
(c) 7. Expression during the cell Cycle (cdc28)(7)
(c) 7. Expression during the cell Cycle (cdc28)(8)
(c) 7. Expression during the cell Cycle (cdc28)(9)
(c) 7. Expression during the cell Cycle (cdc28)(16)
(c) 8. Expression during the cell cycle (cell size selection and release)(8)
(c) 8. Expression during the cell cycle (cell size selection and release)(9)

(c) 8. Expression during the cell cycle (cell size selection and release)(10)
(c) 8. Expression during the cell cycle (cell size selection and release)(11)
(c) 8. Expression during the cell cycle (cell size selection and release)(12)
(c) 8. Expression during the cell cycle (cell size selection and release)(13)
(c) 8. Expression during the cell cycle (cell size selection and release)(14)
(c) 556. Brown enviromental changes :dtt 480 min dtt-2(1)
(c) 570. Brown enviromental changes :1M sorbitol - 90 min(1)

MCM1 -*-> YNL058C

(c) 3. Cell Cycle: Expression in response to Clb2p (set 1, 40 min)(1)
(c) 4. Cell Cycle: Expression in response to Clb2p (set 2, 30 min)(1)
(c) 5. Expression during the cell cycle (alpha factor arrest and release)(7)
(c) 5. Expression during the cell cycle (alpha factor arrest and release)(8)
(c) 5. Expression during the cell cycle (alpha factor arrest and release)(9)
(c) 5. Expression during the cell cycle (alpha factor arrest and release)(10)
(c) 5. Expression during the cell cycle (alpha factor arrest and release)(16)
(c) 5. Expression during the cell cycle (alpha factor arrest and release)(17)
(c) 5. Expression during the cell cycle (alpha factor arrest and release)(18)
(c) 6. Expression during the cell cycle (cdc15 arrest and release)(5)
(c) 6. Expression during the cell cycle (cdc15 arrest and release)(7)
(c) 6. Expression during the cell cycle (cdc15 arrest and release)(8)
(c) 6. Expression during the cell cycle (cdc15 arrest and release)(16)
(c) 6. Expression during the cell cycle (cdc15 arrest and release)(18)
(c) 6. Expression during the cell cycle (cdc15 arrest and release)(19)
(c) 6. Expression during the cell cycle (cdc15 arrest and release)(20)
(c) 7. Expression during the cell Cycle (cdc28)(7)
(c) 7. Expression during the cell Cycle (cdc28)(8)
(c) 7. Expression during the cell Cycle (cdc28)(9)
(c) 7. Expression during the cell Cycle (cdc28)(16)
(c) 8. Expression during the cell cycle (cell size selection and release)(8)
(c) 8. Expression during the cell cycle (cell size selection and release)(9)
(c) 8. Expression during the cell cycle (cell size selection and release)(10)
(c) 8. Expression during the cell cycle (cell size selection and release)(11)
(c) 8. Expression during the cell cycle (cell size selection and release)(12)
(c) 8. Expression during the cell cycle (cell size selection and release)(13)
(c) 8. Expression during the cell cycle (cell size selection and release)(14)
(c) 556. Brown enviromental changes :dtt 480 min dtt-2(1)
(c) 570. Brown enviromental changes :1M sorbitol - 90 min(1)

MCM1 -*-> YOL070C

(c) 3. Cell Cycle: Expression in response to Clb2p (set 1, 40 min)(1)
(c) 4. Cell Cycle: Expression in response to Clb2p (set 2, 30 min)(1)
(c) 5. Expression during the cell cycle (alpha factor arrest and release)(7)
(c) 5. Expression during the cell cycle (alpha factor arrest and release)(8)
(c) 5. Expression during the cell cycle (alpha factor arrest and release)(9)
(c) 5. Expression during the cell cycle (alpha factor arrest and release)(10)
(c) 5. Expression during the cell cycle (alpha factor arrest and release)(16)
(c) 5. Expression during the cell cycle (alpha factor arrest and release)(17)
(c) 5. Expression during the cell cycle (alpha factor arrest and release)(18)
(c) 6. Expression during the cell cycle (cdc15 arrest and release)(5)
(c) 6. Expression during the cell cycle (cdc15 arrest and release)(7)
(c) 6. Expression during the cell cycle (cdc15 arrest and release)(8)
(c) 6. Expression during the cell cycle (cdc15 arrest and release)(16)
(c) 6. Expression during the cell cycle (cdc15 arrest and release)(18)
(c) 6. Expression during the cell cycle (cdc15 arrest and release)(19)
(c) 6. Expression during the cell cycle (cdc15 arrest and release)(20)
(c) 7. Expression during the cell Cycle (cdc28)(7)
(c) 7. Expression during the cell Cycle (cdc28)(8)
(c) 7. Expression during the cell Cycle (cdc28)(9)
(c) 7. Expression during the cell Cycle (cdc28)(16)
(c) 8. Expression during the cell cycle (cell size selection and release)(8)
(c) 8. Expression during the cell cycle (cell size selection and release)(9)
(c) 8. Expression during the cell cycle (cell size selection and release)(10)
(c) 8. Expression during the cell cycle (cell size selection and release)(11)
(c) 8. Expression during the cell cycle (cell size selection and release)(12)
(c) 8. Expression during the cell cycle (cell size selection and release)(13)
(c) 8. Expression during the cell cycle (cell size selection and release)(14)
(c) 556. Brown enviromental changes :dtt 480 min dtt-2(1)
(c) 570. Brown enviromental changes :1M sorbitol - 90 min(1)

MCM1 -*-> YOR315W

(c) 3. Cell Cycle: Expression in response to Clb2p (set 1, 40 min)(1)
(c) 4. Cell Cycle: Expression in response to Clb2p (set 2, 30 min)(1)
(c) 5. Expression during the cell cycle (alpha factor arrest and release)(7)
(c) 5. Expression during the cell cycle (alpha factor arrest and release)(8)
(c) 5. Expression during the cell cycle (alpha factor arrest and release)(9)
(c) 5. Expression during the cell cycle (alpha factor arrest and release)(10)
(c) 5. Expression during the cell cycle (alpha factor arrest and release)(16)
(c) 5. Expression during the cell cycle (alpha factor arrest and release)(17)
(c) 5. Expression during the cell cycle (alpha factor arrest and release)(18)
(c) 6. Expression during the cell cycle (cdc15 arrest and release)(5)
(c) 6. Expression during the cell cycle (cdc15 arrest and release)(7)
(c) 6. Expression during the cell cycle (cdc15 arrest and release)(8)
(c) 6. Expression during the cell cycle (cdc15 arrest and release)(16)
(c) 6. Expression during the cell cycle (cdc15 arrest and release)(18)
(c) 6. Expression during the cell cycle (cdc15 arrest and release)(19)
(c) 6. Expression during the cell cycle (cdc15 arrest and release)(20)
(c) 7. Expression during the cell Cycle (cdc28)(7)
(c) 7. Expression during the cell Cycle (cdc28)(8)
(c) 7. Expression during the cell Cycle (cdc28)(9)
(c) 7. Expression during the cell Cycle (cdc28)(16)
(c) 8. Expression during the cell cycle (cell size selection and release)(8)
(c) 8. Expression during the cell cycle (cell size selection and release)(9)
(c) 8. Expression during the cell cycle (cell size selection and release)(10)
(c) 8. Expression during the cell cycle (cell size selection and release)(11)
(c) 8. Expression during the cell cycle (cell size selection and release)(12)
(c) 8. Expression during the cell cycle (cell size selection and release)(13)
(c) 8. Expression during the cell cycle (cell size selection and release)(14)
(c) 556. Brown enviromental changes :dtt 480 min dtt-2(1)
(c) 570. Brown enviromental changes :1M sorbitol - 90 min(1)

MSN2 -*-> SOL4

(c) 481. Expression in response to heat shock: 15,30,45,60,120 min(1)
(c) 481. Expression in response to heat shock: 15,30,45,60,120 min(2)
(c) 482. Expression in response to acid: 10,20,40,60,80,100 min(1)
(c) 482. Expression in response to acid: 10,20,40,60,80,100 min(2)
(c) 486. Expression in response to NaCl: 15 30 45 60 120 min(2)
(c) 486. Expression in response to NaCl: 15 30 45 60 120 min(3)
(c) 486. Expression in response to NaCl: 15 30 45 60 120 min(4)
(c) 487. Expression in response to sorbitol: 15 30 45 90 120 min(2)
(c) 487. Expression in response to sorbitol: 15 30 45 90 120 min(3)
(c) 488. Brown enviromental changes :Heat Shock 05 minutes hs-1(1)
(c) 489. Brown enviromental changes :Heat Shock 10 minutes hs-1(1)
(c) 490. Brown enviromental changes :Heat Shock 15 minutes hs-1(1)
(c) 491. Brown enviromental changes :Heat Shock 20 minutes hs-1(1)
(c) 492. Brown enviromental changes :Heat Shock 30 minutes hs-1(1)
(c) 493. Brown enviromental changes :Heat Shock 40 minutes hs-1(1)
(c) 494. Brown enviromental changes :Heat Shock 60 minutes hs-1(1)
(c) 508. Brown enviromental changes :heat shock 17 to 37, 20 minutes(1)
(c) 509. Brown enviromental changes :heat shock 21 to 37, 20 minutes(1)
(c) 510. Brown enviromental changes :heat shock 25 to 37, 20 minutes(1)
(c) 511. Brown enviromental changes :heat shock 29 to 37, 20 minutes(1)
(c) 512. Brown enviromental changes :heat shock 33 to 37, 20 minutes(1)
(c) 514. Brown enviromental changes :29C to 33C - 15 minutes(1)
(c) 517. Brown enviromental changes :29C +1M sorbitol to 33C + 1M sorbitol - 5 minutes(1)
(c) 518. Brown enviromental changes :29C +1M sorbitol to 33C + 1M sorbitol - 15 minutes(1)
(c) 558. Brown enviromental changes :1.5 mM diamide (10 min)(1)
(c) 559. Brown enviromental changes :1.5 mM diamide (20 min)(1)
(c) 560. Brown enviromental changes :1.5 mM diamide (30 min)(1)
(c) 561. Brown enviromental changes :1.5 mM diamide (40 min)(1)
(c) 566. Brown enviromental changes :1M sorbitol - 15 min(1)
(c) 567. Brown enviromental changes :1M sorbitol - 30 min(1)
(c) 568. Brown enviromental changes :1M sorbitol - 45 min (1)
(c) 623. Brown enviromental changes :DBY7286 37degree heat - 20 min(1)
(c) Addition of 1M NaCl (90')

MSN2 -*-> TSL1

(c) 481. Expression in response to heat shock: 15,30,45,60,120 min(1)
(c) 481. Expression in response to heat shock: 15,30,45,60,120 min(2)
(c) 482. Expression in response to acid: 10,20,40,60,80,100 min(1)
(c) 482. Expression in response to acid: 10,20,40,60,80,100 min(2)
(c) 486. Expression in response to NaCl: 15 30 45 60 120 min(2)
(c) 486. Expression in response to NaCl: 15 30 45 60 120 min(3)
(c) 486. Expression in response to NaCl: 15 30 45 60 120 min(4)
(c) 487. Expression in response to sorbitol: 15 30 45 90 120 min(2)
(c) 487. Expression in response to sorbitol: 15 30 45 90 120 min(3)
(c) 488. Brown enviromental changes :Heat Shock 05 minutes hs-1(1)
(c) 489. Brown enviromental changes :Heat Shock 10 minutes hs-1(1)
(c) 490. Brown enviromental changes :Heat Shock 15 minutes hs-1(1)
(c) 491. Brown enviromental changes :Heat Shock 20 minutes hs-1(1)
(c) 492. Brown enviromental changes :Heat Shock 30 minutes hs-1(1)
(c) 493. Brown enviromental changes :Heat Shock 40 minutes hs-1(1)
(c) 494. Brown enviromental changes :Heat Shock 60 minutes hs-1(1)
(c) 508. Brown enviromental changes :heat shock 17 to 37, 20 minutes(1)
(c) 509. Brown enviromental changes :heat shock 21 to 37, 20 minutes(1)
(c) 510. Brown enviromental changes :heat shock 25 to 37, 20 minutes(1)
(c) 511. Brown enviromental changes :heat shock 29 to 37, 20 minutes(1)
(c) 512. Brown enviromental changes :heat shock 33 to 37, 20 minutes(1)
(c) 514. Brown enviromental changes :29C to 33C - 15 minutes(1)
(c) 517. Brown enviromental changes :29C +1M sorbitol to 33C + 1M sorbitol - 5 minutes(1)
(c) 518. Brown enviromental changes :29C +1M sorbitol to 33C + 1M sorbitol - 15 minutes(1)
(c) 558. Brown enviromental changes :1.5 mM diamide (10 min)(1)
(c) 559. Brown enviromental changes :1.5 mM diamide (20 min)(1)
(c) 560. Brown enviromental changes :1.5 mM diamide (30 min)(1)
(c) 561. Brown enviromental changes :1.5 mM diamide (40 min)(1)
(c) 566. Brown enviromental changes :1M sorbitol - 15 min(1)
(c) 567. Brown enviromental changes :1M sorbitol - 30 min(1)
(c) 568. Brown enviromental changes :1M sorbitol - 45 min (1)
(c) 623. Brown enviromental changes :DBY7286 37degree heat - 20 min(1)
(c) Addition of 1M NaCl (90')

MSN4 -*-> MSC1

(c) 481. Expression in response to heat shock: 15,30,45,60,120 min(1)
(c) 481. Expression in response to heat shock: 15,30,45,60,120 min(2)
(c) 482. Expression in response to acid: 10,20,40,60,80,100 min(1)
(c) 482. Expression in response to acid: 10,20,40,60,80,100 min(2)
(c) 486. Expression in response to NaCl: 15 30 45 60 120 min(2)
(c) 486. Expression in response to NaCl: 15 30 45 60 120 min(3)
(c) 486. Expression in response to NaCl: 15 30 45 60 120 min(4)
(c) 487. Expression in response to sorbitol: 15 30 45 90 120 min(2)
(c) 487. Expression in response to sorbitol: 15 30 45 90 120 min(3)
(c) 488. Brown enviromental changes :Heat Shock 05 minutes hs-1(1)
(c) 489. Brown enviromental changes :Heat Shock 10 minutes hs-1(1)
(c) 490. Brown enviromental changes :Heat Shock 15 minutes hs-1(1)
(c) 491. Brown enviromental changes :Heat Shock 20 minutes hs-1(1)
(c) 492. Brown enviromental changes :Heat Shock 30 minutes hs-1(1)
(c) 493. Brown enviromental changes :Heat Shock 40 minutes hs-1(1)
(c) 494. Brown enviromental changes :Heat Shock 60 minutes hs-1(1)
(c) 508. Brown enviromental changes :heat shock 17 to 37, 20 minutes(1)
(c) 509. Brown enviromental changes :heat shock 21 to 37, 20 minutes(1)
(c) 510. Brown enviromental changes :heat shock 25 to 37, 20 minutes(1)
(c) 511. Brown enviromental changes :heat shock 29 to 37, 20 minutes(1)
(c) 512. Brown enviromental changes :heat shock 33 to 37, 20 minutes(1)
(c) 514. Brown enviromental changes :29C to 33C - 15 minutes(1)
(c) 517. Brown enviromental changes :29C +1M sorbitol to 33C + 1M sorbitol - 5 minutes(1)
(c) 518. Brown enviromental changes :29C +1M sorbitol to 33C + 1M sorbitol - 15 minutes(1)
(c) 558. Brown enviromental changes :1.5 mM diamide (10 min)(1)
(c) 559. Brown enviromental changes :1.5 mM diamide (20 min)(1)
(c) 560. Brown enviromental changes :1.5 mM diamide (30 min)(1)
(c) 561. Brown enviromental changes :1.5 mM diamide (40 min)(1)
(c) 562. Brown enviromental changes :1.5 mM diamide (50 min)(1)
(c) 566. Brown enviromental changes :1M sorbitol - 15 min(1)
(c) 567. Brown enviromental changes :1M sorbitol - 30 min(1)
(c) 568. Brown enviromental changes :1M sorbitol - 45 min (1)
(c) 623. Brown enviromental changes :DBY7286 37degree heat - 20 min(1)
(c) Addition of 1M NaCl (90')

(c) DES460 (wild type) + heat 20 min

MSN4 -*-> SOL4

(c) 481. Expression in response to heat shock: 15,30,45,60,120 min(1)
(c) 481. Expression in response to heat shock: 15,30,45,60,120 min(2)
(c) 482. Expression in response to acid: 10,20,40,60,80,100 min(1)
(c) 482. Expression in response to acid: 10,20,40,60,80,100 min(2)
(c) 486. Expression in response to NaCl: 15 30 45 60 120 min(2)
(c) 486. Expression in response to NaCl: 15 30 45 60 120 min(3)
(c) 486. Expression in response to NaCl: 15 30 45 60 120 min(4)
(c) 487. Expression in response to sorbitol: 15 30 45 90 120 min(2)
(c) 487. Expression in response to sorbitol: 15 30 45 90 120 min(3)
(c) 488. Brown enviromental changes :Heat Shock 05 minutes hs-1(1)
(c) 489. Brown enviromental changes :Heat Shock 10 minutes hs-1(1)
(c) 490. Brown enviromental changes :Heat Shock 15 minutes hs-1(1)
(c) 491. Brown enviromental changes :Heat Shock 20 minutes hs-1(1)
(c) 492. Brown enviromental changes :Heat Shock 30 minutes hs-1(1)
(c) 493. Brown enviromental changes :Heat Shock 40 minutes hs-1(1)
(c) 494. Brown enviromental changes :Heat Shock 60 minutes hs-1(1)
(c) 508. Brown enviromental changes :heat shock 17 to 37, 20 minutes(1)
(c) 509. Brown enviromental changes :heat shock 21 to 37, 20 minutes(1)
(c) 510. Brown enviromental changes :heat shock 25 to 37, 20 minutes(1)
(c) 511. Brown enviromental changes :heat shock 29 to 37, 20 minutes(1)
(c) 512. Brown enviromental changes :heat shock 33 to 37, 20 minutes(1)
(c) 514. Brown enviromental changes :29C to 33C - 15 minutes(1)
(c) 517. Brown enviromental changes :29C +1M sorbitol to 33C + 1M sorbitol - 5 minutes(1)
(c) 518. Brown enviromental changes :29C +1M sorbitol to 33C + 1M sorbitol - 15 minutes(1)
(c) 558. Brown enviromental changes :1.5 mM diamide (10 min)(1)
(c) 559. Brown enviromental changes :1.5 mM diamide (20 min)(1)
(c) 560. Brown enviromental changes :1.5 mM diamide (30 min)(1)
(c) 561. Brown enviromental changes :1.5 mM diamide (40 min)(1)
(c) 562. Brown enviromental changes :1.5 mM diamide (50 min)(1)
(c) 566. Brown enviromental changes :1M sorbitol - 15 min(1)
(c) 567. Brown enviromental changes :1M sorbitol - 30 min(1)
(c) 568. Brown enviromental changes :1M sorbitol - 45 min (1)
(c) 623. Brown enviromental changes :DBY7286 37degree heat - 20 min(1)
(c) Addition of 1M NaCl (90')
(c) DES460 (wild type) + heat 20 min

MSN4 -*-> SSE2

(c) 481. Expression in response to heat shock: 15,30,45,60,120 min(1)
(c) 481. Expression in response to heat shock: 15,30,45,60,120 min(2)
(c) 482. Expression in response to acid: 10,20,40,60,80,100 min(1)
(c) 482. Expression in response to acid: 10,20,40,60,80,100 min(2)
(c) 486. Expression in response to NaCl: 15 30 45 60 120 min(2)
(c) 486. Expression in response to NaCl: 15 30 45 60 120 min(3)
(c) 486. Expression in response to NaCl: 15 30 45 60 120 min(4)
(c) 487. Expression in response to sorbitol: 15 30 45 90 120 min(2)
(c) 487. Expression in response to sorbitol: 15 30 45 90 120 min(3)
(c) 488. Brown enviromental changes :Heat Shock 05 minutes hs-1(1)
(c) 489. Brown enviromental changes :Heat Shock 10 minutes hs-1(1)
(c) 490. Brown enviromental changes :Heat Shock 15 minutes hs-1(1)
(c) 491. Brown enviromental changes :Heat Shock 20 minutes hs-1(1)
(c) 492. Brown enviromental changes :Heat Shock 30 minutes hs-1(1)
(c) 493. Brown enviromental changes :Heat Shock 40 minutes hs-1(1)
(c) 494. Brown enviromental changes :Heat Shock 60 minutes hs-1(1)
(c) 508. Brown enviromental changes :heat shock 17 to 37, 20 minutes(1)
(c) 509. Brown enviromental changes :heat shock 21 to 37, 20 minutes(1)
(c) 510. Brown enviromental changes :heat shock 25 to 37, 20 minutes(1)
(c) 511. Brown enviromental changes :heat shock 29 to 37, 20 minutes(1)
(c) 512. Brown enviromental changes :heat shock 33 to 37, 20 minutes(1)
(c) 514. Brown enviromental changes :29C to 33C - 15 minutes(1)
(c) 517. Brown enviromental changes :29C +1M sorbitol to 33C + 1M sorbitol - 5 minutes(1)
(c) 518. Brown enviromental changes :29C +1M sorbitol to 33C + 1M sorbitol - 15 minutes(1)
(c) 558. Brown enviromental changes :1.5 mM diamide (10 min)(1)
(c) 559. Brown enviromental changes :1.5 mM diamide (20 min)(1)
(c) 560. Brown enviromental changes :1.5 mM diamide (30 min)(1)
(c) 561. Brown enviromental changes :1.5 mM diamide (40 min)(1)
(c) 562. Brown enviromental changes :1.5 mM diamide (50 min)(1)
(c) 566. Brown enviromental changes :1M sorbitol - 15 min(1)
(c) 567. Brown enviromental changes :1M sorbitol - 30 min(1)
(c) 568. Brown enviromental changes :1M sorbitol - 45 min (1)
(c) 623. Brown enviromental changes :DBY7286 37degree heat - 20 min(1)
(c) Addition of 1M NaCl (90')
(c) DES460 (wild type) + heat 20 min

MSN4 -*-> TSL1

(c) 481. Expression in response to heat shock: 15,30,45,60,120 min(1)
(c) 481. Expression in response to heat shock: 15,30,45,60,120 min(2)
(c) 482. Expression in response to acid: 10,20,40,60,80,100 min(1)
(c) 482. Expression in response to acid: 10,20,40,60,80,100 min(2)
(c) 486. Expression in response to NaCl: 15 30 45 60 120 min(2)
(c) 486. Expression in response to NaCl: 15 30 45 60 120 min(3)
(c) 486. Expression in response to NaCl: 15 30 45 60 120 min(4)
(c) 487. Expression in response to sorbitol: 15 30 45 90 120 min(2)
(c) 487. Expression in response to sorbitol: 15 30 45 90 120 min(3)
(c) 488. Brown enviromental changes :Heat Shock 05 minutes hs-1(1)
(c) 489. Brown enviromental changes :Heat Shock 10 minutes hs-1(1)
(c) 490. Brown enviromental changes :Heat Shock 15 minutes hs-1(1)
(c) 491. Brown enviromental changes :Heat Shock 20 minutes hs-1(1)
(c) 492. Brown enviromental changes :Heat Shock 30 minutes hs-1(1)
(c) 493. Brown enviromental changes :Heat Shock 40 minutes hs-1(1)
(c) 494. Brown enviromental changes :Heat Shock 60 minutes hs-1(1)
(c) 508. Brown enviromental changes :heat shock 17 to 37, 20 minutes(1)
(c) 509. Brown enviromental changes :heat shock 21 to 37, 20 minutes(1)
(c) 510. Brown enviromental changes :heat shock 25 to 37, 20 minutes(1)
(c) 511. Brown enviromental changes :heat shock 29 to 37, 20 minutes(1)
(c) 512. Brown enviromental changes :heat shock 33 to 37, 20 minutes(1)
(c) 514. Brown enviromental changes :29C to 33C - 15 minutes(1)
(c) 517. Brown enviromental changes :29C +1M sorbitol to 33C + 1M sorbitol - 5 minutes(1)
(c) 518. Brown enviromental changes :29C +1M sorbitol to 33C + 1M sorbitol - 15 minutes(1)
(c) 558. Brown enviromental changes :1.5 mM diamide (10 min)(1)
(c) 559. Brown enviromental changes :1.5 mM diamide (20 min)(1)

(c) 560. Brown enviromental changes :1.5 mM diamide (30 min)(1)
(c) 561. Brown enviromental changes :1.5 mM diamide (40 min)(1)
(c) 562. Brown enviromental changes :1.5 mM diamide (50 min)(1)
(c) 566. Brown enviromental changes :1M sorbitol - 15 min(1)
(c) 567. Brown enviromental changes :1M sorbitol - 30 min(1)
(c) 568. Brown enviromental changes :1M sorbitol - 45 min (1)
(c) 623. Brown enviromental changes :DBY7286 37degree heat - 20 min(1)
(c) Addition of 1M NaCl (90')
(c) DES460 (wild type) + heat 20 min

MSN4 -*-> YHL021C

(c) 481. Expression in response to heat shock: 15,30,45,60,120 min(1)
(c) 481. Expression in response to heat shock: 15,30,45,60,120 min(2)
(c) 482. Expression in response to acid: 10,20,40,60,80,100 min(1)
(c) 482. Expression in response to acid: 10,20,40,60,80,100 min(2)
(c) 486. Expression in response to NaCl: 15 30 45 60 120 min(2)
(c) 486. Expression in response to NaCl: 15 30 45 60 120 min(3)
(c) 486. Expression in response to NaCl: 15 30 45 60 120 min(4)
(c) 487. Expression in response to sorbitol: 15 30 45 90 120 min(2)
(c) 487. Expression in response to sorbitol: 15 30 45 90 120 min(3)
(c) 488. Brown enviromental changes :Heat Shock 05 minutes hs-1(1)
(c) 489. Brown enviromental changes :Heat Shock 10 minutes hs-1(1)
(c) 490. Brown enviromental changes :Heat Shock 15 minutes hs-1(1)
(c) 491. Brown enviromental changes :Heat Shock 20 minutes hs-1(1)
(c) 492. Brown enviromental changes :Heat Shock 30 minutes hs-1(1)
(c) 493. Brown enviromental changes :Heat Shock 40 minutes hs-1(1)
(c) 494. Brown enviromental changes :Heat Shock 60 minutes hs-1(1)
(c) 508. Brown enviromental changes :heat shock 17 to 37, 20 minutes(1)
(c) 509. Brown enviromental changes :heat shock 21 to 37, 20 minutes(1)
(c) 510. Brown enviromental changes :heat shock 25 to 37, 20 minutes(1)
(c) 511. Brown enviromental changes :heat shock 29 to 37, 20 minutes(1)
(c) 512. Brown enviromental changes :heat shock 33 to 37, 20 minutes(1)
(c) 514. Brown enviromental changes :29C to 33C - 15 minutes(1)
(c) 517. Brown enviromental changes :29C +1M sorbitol to 33C + 1M sorbitol - 5 minutes(1)
(c) 518. Brown enviromental changes :29C +1M sorbitol to 33C + 1M sorbitol - 15 minutes(1)
(c) 558. Brown enviromental changes :1.5 mM diamide (10 min)(1)
(c) 559. Brown enviromental changes :1.5 mM diamide (20 min)(1)
(c) 560. Brown enviromental changes :1.5 mM diamide (30 min)(1)
(c) 561. Brown enviromental changes :1.5 mM diamide (40 min)(1)
(c) 562. Brown enviromental changes :1.5 mM diamide (50 min)(1)
(c) 566. Brown enviromental changes :1M sorbitol - 15 min(1)
(c) 567. Brown enviromental changes :1M sorbitol - 30 min(1)
(c) 568. Brown enviromental changes :1M sorbitol - 45 min (1)
(c) 623. Brown enviromental changes :DBY7286 37degree heat - 20 min(1)
(c) Addition of 1M NaCl (90')
(c) DES460 (wild type) + heat 20 min

PHO4 -*-> CTF19

(c) 3. Cell Cycle: Expression in response to Clb2p (set 1, 40 min)(1)
(c) 5. Expression during the cell cycle (alpha factor arrest and release)(9)
(c) 5. Expression during the cell cycle (alpha factor arrest and release)(10)
(c) 5. Expression during the cell cycle (alpha factor arrest and release)(11)
(c) 5. Expression during the cell cycle (alpha factor arrest and release)(12)
(c) PHO4c vs WT(1)
(c) pho80 vs WT(1)
(c) pho85 vs WT(1)
(c) PHO81c vs WT exp1(1)
(c) PHO81c vs WT exp2(1)
(c) 537. Brown enviromental changes :1 mM Menadione (50 min)redo(1)
(c) 573. Brown enviromental changes :Hypo-osmotic shock - 15 min(1)
(c) 574. Brown enviromental changes :Hypo-osmotic shock - 30 min(1)
(c) 575. Brown enviromental changes :Hypo-osmotic shock - 45 min(1)
(c) 576. Brown enviromental changes :Hypo-osmotic shock - 60 min(1)
(c) MAC1-up (B)
(c) MAC1-up (C)

PHO4 -*-| HIS1

(c) 3. Cell Cycle: Expression in response to Clb2p (set 1, 40 min)(1)
(c) 5. Expression during the cell cycle (alpha factor arrest and release)(9)
(c) 5. Expression during the cell cycle (alpha factor arrest and release)(10)
(c) 5. Expression during the cell cycle (alpha factor arrest and release)(11)
(c) 5. Expression during the cell cycle (alpha factor arrest and release)(12)
(c) PHO4c vs WT(1)
(c) pho80 vs WT(1)
(c) pho85 vs WT(1)
(c) PHO81c vs WT exp1(1)
(c) PHO81c vs WT exp2(1)
(c) 537. Brown enviromental changes :1 mM Menadione (50 min)redo(1)
(c) 573. Brown enviromental changes :Hypo-osmotic shock - 15 min(1)
(c) 574. Brown enviromental changes :Hypo-osmotic shock - 30 min(1)
(c) 575. Brown enviromental changes :Hypo-osmotic shock - 45 min(1)
(c) 576. Brown enviromental changes :Hypo-osmotic shock - 60 min(1)
(c) MAC1-up (B)
(c) MAC1-up (C)

PHO4 -*-> PHM6

(c) 3. Cell Cycle: Expression in response to Clb2p (set 1, 40 min)(1)
(c) 5. Expression during the cell cycle (alpha factor arrest and release)(9)
(c) 5. Expression during the cell cycle (alpha factor arrest and release)(10)
(c) 5. Expression during the cell cycle (alpha factor arrest and release)(11)
(c) 5. Expression during the cell cycle (alpha factor arrest and release)(12)
(c) PHO4c vs WT(1)
(c) pho80 vs WT(1)
(c) pho85 vs WT(1)
(c) PHO81c vs WT exp1(1)
(c) PHO81c vs WT exp2(1)
(c) 537. Brown enviromental changes :1 mM Menadione (50 min)redo(1)
(c) 573. Brown enviromental changes :Hypo-osmotic shock - 15 min(1)
(c) 574. Brown enviromental changes :Hypo-osmotic shock - 30 min(1)
(c) 575. Brown enviromental changes :Hypo-osmotic shock - 45 min(1)

(c) 576. Brown enviromental changes :Hypo-osmotic shock - 60 min(1)
(c) MAC1-up (B)
(c) MAC1-up (C)

PHO4 -*-> PHO86

(c) 3. Cell Cycle: Expression in response to Clb2p (set 1, 40 min)(1)
(c) 5. Expression during the cell cycle (alpha factor arrest and release)(9)
(c) 5. Expression during the cell cycle (alpha factor arrest and release)(10)
(c) 5. Expression during the cell cycle (alpha factor arrest and release)(11)
(c) 5. Expression during the cell cycle (alpha factor arrest and release)(12)
(c) PHO4c vs WT(1)
(c) pho80 vs WT(1)
(c) pho85 vs WT(1)
(c) PHO81c vs WT exp1(1)
(c) PHO81c vs WT exp2(1)
(c) 537. Brown enviromental changes :1 mM Menadione (50 min)redo(1)
(c) 573. Brown enviromental changes :Hypo-osmotic shock - 15 min(1)
(c) 574. Brown enviromental changes :Hypo-osmotic shock - 30 min(1)
(c) 575. Brown enviromental changes :Hypo-osmotic shock - 45 min(1)
(c) 576. Brown enviromental changes :Hypo-osmotic shock - 60 min(1)
(c) MAC1-up (B)
(c) MAC1-up (C)

PHO4 -*-> VIP1

(c) 3. Cell Cycle: Expression in response to Clb2p (set 1, 40 min)(1)
(c) 5. Expression during the cell cycle (alpha factor arrest and release)(9)
(c) 5. Expression during the cell cycle (alpha factor arrest and release)(10)
(c) 5. Expression during the cell cycle (alpha factor arrest and release)(11)
(c) 5. Expression during the cell cycle (alpha factor arrest and release)(12)
(c) PHO4c vs WT(1)
(c) pho80 vs WT(1)
(c) pho85 vs WT(1)
(c) PHO81c vs WT exp1(1)
(c) PHO81c vs WT exp2(1)
(c) 537. Brown enviromental changes :1 mM Menadione (50 min)redo(1)
(c) 573. Brown enviromental changes :Hypo-osmotic shock - 15 min(1)
(c) 574. Brown enviromental changes :Hypo-osmotic shock - 30 min(1)
(c) 575. Brown enviromental changes :Hypo-osmotic shock - 45 min(1)
(c) 576. Brown enviromental changes :Hypo-osmotic shock - 60 min(1)
(c) MAC1-up (B)
(c) MAC1-up (C)

PHO4 -*-> VTC1

(c) 3. Cell Cycle: Expression in response to Clb2p (set 1, 40 min)(1)
(c) 5. Expression during the cell cycle (alpha factor arrest and release)(9)
(c) 5. Expression during the cell cycle (alpha factor arrest and release)(10)
(c) 5. Expression during the cell cycle (alpha factor arrest and release)(11)
(c) 5. Expression during the cell cycle (alpha factor arrest and release)(12)
(c) PHO4c vs WT(1)
(c) pho80 vs WT(1)
(c) pho85 vs WT(1)
(c) PHO81c vs WT exp1(1)
(c) PHO81c vs WT exp2(1)
(c) 537. Brown enviromental changes :1 mM Menadione (50 min)redo(1)
(c) 573. Brown enviromental changes :Hypo-osmotic shock - 15 min(1)
(c) 574. Brown enviromental changes :Hypo-osmotic shock - 30 min(1)
(c) 575. Brown enviromental changes :Hypo-osmotic shock - 45 min(1)
(c) 576. Brown enviromental changes :Hypo-osmotic shock - 60 min(1)
(c) MAC1-up (B)
(c) MAC1-up (C)

PHO4 -*-> VTC2

(c) 3. Cell Cycle: Expression in response to Clb2p (set 1, 40 min)(1)
(c) 5. Expression during the cell cycle (alpha factor arrest and release)(9)
(c) 5. Expression during the cell cycle (alpha factor arrest and release)(10)
(c) 5. Expression during the cell cycle (alpha factor arrest and release)(11)
(c) 5. Expression during the cell cycle (alpha factor arrest and release)(12)
(c) PHO4c vs WT(1)
(c) pho80 vs WT(1)
(c) pho85 vs WT(1)
(c) PHO81c vs WT exp1(1)
(c) PHO81c vs WT exp2(1)
(c) 537. Brown enviromental changes :1 mM Menadione (50 min)redo(1)
(c) 573. Brown enviromental changes :Hypo-osmotic shock - 15 min(1)
(c) 574. Brown enviromental changes :Hypo-osmotic shock - 30 min(1)
(c) 575. Brown enviromental changes :Hypo-osmotic shock - 45 min(1)
(c) 576. Brown enviromental changes :Hypo-osmotic shock - 60 min(1)
(c) MAC1-up (B)
(c) MAC1-up (C)

PHO4 -*-> VTC3

(c) 3. Cell Cycle: Expression in response to Clb2p (set 1, 40 min)(1)
(c) 5. Expression during the cell cycle (alpha factor arrest and release)(9)
(c) 5. Expression during the cell cycle (alpha factor arrest and release)(10)
(c) 5. Expression during the cell cycle (alpha factor arrest and release)(11)
(c) 5. Expression during the cell cycle (alpha factor arrest and release)(12)
(c) PHO4c vs WT(1)
(c) pho80 vs WT(1)
(c) pho85 vs WT(1)
(c) PHO81c vs WT exp1(1)
(c) PHO81c vs WT exp2(1)
(c) 537. Brown enviromental changes :1 mM Menadione (50 min)redo(1)
(c) 573. Brown enviromental changes :Hypo-osmotic shock - 15 min(1)
(c) 574. Brown enviromental changes :Hypo-osmotic shock - 30 min(1)
(c) 575. Brown enviromental changes :Hypo-osmotic shock - 45 min(1)
(c) 576. Brown enviromental changes :Hypo-osmotic shock - 60 min(1)
(c) MAC1-up (B)

(c) MAC1-up (C)

PHO4 -*-> VTC4

(c) 3. Cell Cycle: Expression in response to Clb2p (set 1, 40 min)(1)
(c) 5. Expression during the cell cycle (alpha factor arrest and release)(9)
(c) 5. Expression during the cell cycle (alpha factor arrest and release)(10)
(c) 5. Expression during the cell cycle (alpha factor arrest and release)(11)
(c) 5. Expression during the cell cycle (alpha factor arrest and release)(12)
(c) PHO4c vs WT(1)
(c) pho80 vs WT(1)
(c) pho85 vs WT(1)
(c) PHO81c vs WT exp1(1)
(c) PHO81c vs WT exp2(1)
(c) 537. Brown enviromental changes :1 mM Menadione (50 min)redo(1)
(c) 573. Brown enviromental changes :Hypo-osmotic shock - 15 min(1)
(c) 574. Brown enviromental changes :Hypo-osmotic shock - 30 min(1)
(c) 575. Brown enviromental changes :Hypo-osmotic shock - 45 min(1)
(c) 576. Brown enviromental changes :Hypo-osmotic shock - 60 min(1)
(c) MAC1-up (B)
(c) MAC1-up (C)

RAP1 -*-| GPM1

(c) 6. Expression during the cell cycle (cdc15 arrest and release)(17)
(c) 6. Expression during the cell cycle (cdc15 arrest and release)(18)
(c) 7. Expression during the cell Cycle (cdc28)(10)
(c) 7. Expression during the cell Cycle (cdc28)(15)
(c) 7. Expression during the cell Cycle (cdc28)(16)
(c) 390. Rosetta 2000: Expression in cells with IDI1 under tet promoter(1)
(c) 391. Rosetta 2000: Expression in cells with KAR2 under tet promoter(1)
(c) 393. Rosetta 2000: Expression in cells with RHO1 under tet promoter(1)
(c) 477. Expression in response to trichostatin A (TSA): 15min,30min,60min,120min(2)
(c) 477. Expression in response to trichostatin A (TSA): 15min,30min,60min,120min(3)
(c) 477. Expression in response to trichostatin A (TSA): 15min,30min,60min,120min(4)
(c) 544. Brown enviromental changes :2.5mM DTT 030 min dtt-1(1)
(c) 553. Brown enviromental changes :dtt 060 min dtt-2(1)
(c) 554. Brown enviromental changes :dtt 120 min dtt-2(1)
(c) 563. Brown enviromental changes :1.5 mM diamide (60 min)(1)
(c) 611. Brown enviromental changes :YPD stationary phase 2 h ypd-1(1)
(c) 612. Brown enviromental changes :YPD stationary phase 4 h ypd-1(1)
(c) 613. Brown enviromental changes :YPD stationary phase 8 h ypd-1(1)

RAP1 -*-| PGI1

(c) 6. Expression during the cell cycle (cdc15 arrest and release)(17)
(c) 6. Expression during the cell cycle (cdc15 arrest and release)(18)
(c) 7. Expression during the cell Cycle (cdc28)(10)
(c) 7. Expression during the cell Cycle (cdc28)(15)
(c) 7. Expression during the cell Cycle (cdc28)(16)
(c) 390. Rosetta 2000: Expression in cells with IDI1 under tet promoter(1)
(c) 391. Rosetta 2000: Expression in cells with KAR2 under tet promoter(1)
(c) 393. Rosetta 2000: Expression in cells with RHO1 under tet promoter(1)
(c) 477. Expression in response to trichostatin A (TSA): 15min,30min,60min,120min(2)
(c) 477. Expression in response to trichostatin A (TSA): 15min,30min,60min,120min(3)
(c) 477. Expression in response to trichostatin A (TSA): 15min,30min,60min,120min(4)
(c) 544. Brown enviromental changes :2.5mM DTT 030 min dtt-1(1)
(c) 553. Brown enviromental changes :dtt 060 min dtt-2(1)
(c) 554. Brown enviromental changes :dtt 120 min dtt-2(1)
(c) 563. Brown enviromental changes :1.5 mM diamide (60 min)(1)
(c) 611. Brown enviromental changes :YPD stationary phase 2 h ypd-1(1)
(c) 612. Brown enviromental changes :YPD stationary phase 4 h ypd-1(1)
(c) 613. Brown enviromental changes :YPD stationary phase 8 h ypd-1(1)

RCS1 -*-> ARN1

(c) 8. Expression during the cell cycle (cell size selection and release)(1)
(c) 8. Expression during the cell cycle (cell size selection and release)(2)
(c) 447. Expression in response to 0.1% MMS for 30 min(1)
(c) 449. Expression in response to 0.1% MMS for 60 min(1)
(c) 451. Expression in response to BCNU (200 micromolar) for 60 min(1)
(c) 454. Expression in response to tBuOOH (5mM) for 60 min(1)
(c) 481. Expression in response to heat shock: 15,30,45,60,120 min(1)
(c) 519. Brown enviromental changes :29C +1M sorbitol to 33C + 1M sorbitol - 30 minutes(1)
(c) 527. Brown enviromental changes :constant 0.32 mM H2O2 (50 min) redo(1)
(c) 528. Brown enviromental changes :constant 0.32 mM H2O2 (60 min) redo(1)
(c) 529. Brown enviromental changes :constant 0.32 mM H2O2 (80 min) redo(1)
(c) 531. Brown enviromental changes :constant 0.32 mM H2O2 (120 min) redo(1)
(c) 537. Brown enviromental changes :1 mM Menadione (50 min)redo(1)
(c) 538. Brown enviromental changes :1 mM Menadione (80 min) redo(1)
(c) 556. Brown enviromental changes :dtt 480 min dtt-2(1)
(c) 562. Brown enviromental changes :1.5 mM diamide (50 min)(1)
(c) 563. Brown enviromental changes :1.5 mM diamide (60 min)(1)
(c) 564. Brown enviromental changes :1.5 mM diamide (90 min)(1)
(c) 576. Brown enviromental changes :Hypo-osmotic shock - 60 min(1)
(c) 615. Brown enviromental changes :YPD stationary phase 1 d ypd-1(1)
(c) DES460 + 0.02% MMS - 30 min
(c) DES460 + 0.2% MMS - 45 min
(c) DES460 + 0.02% MMS - 60 min
(c) DES460 + 0.02% MMS - 90 min
(c) DES460 + 0.02% MMS - 120 min
(c) wt_plus_gamma_30_min
(c) wt_plus_gamma_45_min
(c) wt_plus_gamma_60_min
(c) MHY1 (crt1) vs CRY1 (wild type)

RCS1 -*-> TAF1

(c) 8. Expression during the cell cycle (cell size selection and release)(1)
(c) 8. Expression during the cell cycle (cell size selection and release)(2)
(c) 447. Expression in response to 0.1% MMS for 30 min(1)
(c) 449. Expression in response to 0.1% MMS for 60 min(1)

(c) 451. Expression in response to BCNU (200 micromolar) for 60 min(1)
(c) 454. Expression in response to tBuOOH (5mM) for 60 min(1)
(c) 481. Expression in response to heat shock: 15,30,45,60,120 min(1)
(c) 519. Brown enviromental changes :29C +1M sorbitol to 33C + 1M sorbitol - 30 minutes(1)
(c) 527. Brown enviromental changes :constant 0.32 mM H2O2 (50 min) redo(1)
(c) 528. Brown enviromental changes :constant 0.32 mM H2O2 (60 min) redo(1)
(c) 529. Brown enviromental changes :constant 0.32 mM H2O2 (80 min) redo(1)
(c) 531. Brown enviromental changes :constant 0.32 mM H2O2 (120 min) redo(1)
(c) 537. Brown enviromental changes :1 mM Menadione (50 min)redo(1)
(c) 538. Brown enviromental changes :1 mM Menadione (80 min) redo(1)
(c) 556. Brown enviromental changes :dtt 480 min dtt-2(1)
(c) 562. Brown enviromental changes :1.5 mM diamide (50 min)(1)
(c) 563. Brown enviromental changes :1.5 mM diamide (60 min)(1)
(c) 564. Brown enviromental changes :1.5 mM diamide (90 min)(1)
(c) 576. Brown enviromental changes :Hypo-osmotic shock - 60 min(1)
(c) 615. Brown enviromental changes :YPD stationary phase 1 d ypd-1(1)
(c) DES460 + 0.02% MMS - 30 min
(c) DES460 + 0.2% MMS - 45 min
(c) DES460 + 0.02% MMS - 60 min
(c) DES460 + 0.02% MMS - 90 min
(c) DES460 + 0.02% MMS - 120 min
(c) wt_plus_gamma_30_min
(c) wt_plus_gamma_45_min
(c) wt_plus_gamma_60_min
(c) MHY1 (crt1) vs CRY1 (wild type)

RGT1 -*-> HXT6

(c) 2. Cell Cycle: Expression in response to Cln3p (set 2)(1)
(c) 5. Expression during the cell cycle (alpha factor arrest and release)(8)
(c) 8. Expression during the cell cycle (cell size selection and release)(3)
(c) 8. Expression during the cell cycle (cell size selection and release)(6)
(c) 8. Expression during the cell cycle (cell size selection and release)(7)
(c) 13. Expression in cells overexpressing Yap1p(1)
(c) 26. Fink: Expression in diploid high copy TEC1(1)
(c) 450. Expression in response to low MNNG (8 microgram/ml) for 60 min(1)
(c) 483. Expression in response to alkali: 10,20,40,60,80,100 min(2)
(c) 483. Expression in response to alkali: 10,20,40,60,80,100 min(3)
(c) 483. Expression in response to alkali: 10,20,40,60,80,100 min(4)
(c) 483. Expression in response to alkali: 10,20,40,60,80,100 min(5)
(c) 485. Expression in response to peroxide: 10,20,40,60,120 min(1)
(c) 485. Expression in response to peroxide: 10,20,40,60,120 min(2)
(c) 485. Expression in response to peroxide: 10,20,40,60,120 min(3)
(c) 485. Expression in response to peroxide: 10,20,40,60,120 min(4)
(c) 519. Brown enviromental changes :29C +1M sorbitol to 33C + 1M sorbitol - 30 minutes(1)
(c) 534. Brown enviromental changes :1 mM Menadione (20 min) redo(1)
(c) 546. Brown enviromental changes :2.5mM DTT 060 min dtt-1(1)
(c) 573. Brown enviromental changes :Hypo-osmotic shock - 15 min(1)
(c) 574. Brown enviromental changes :Hypo-osmotic shock - 30 min(1)
(c) DES460 (wild type) + heat 20 min
(c) wt+gal

RGT1 -*-> HXT7

(c) 2. Cell Cycle: Expression in response to Cln3p (set 2)(1)
(c) 5. Expression during the cell cycle (alpha factor arrest and release)(8)
(c) 8. Expression during the cell cycle (cell size selection and release)(3)
(c) 8. Expression during the cell cycle (cell size selection and release)(6)
(c) 8. Expression during the cell cycle (cell size selection and release)(7)
(c) 13. Expression in cells overexpressing Yap1p(1)
(c) 26. Fink: Expression in diploid high copy TEC1(1)
(c) 450. Expression in response to low MNNG (8 microgram/ml) for 60 min(1)
(c) 483. Expression in response to alkali: 10,20,40,60,80,100 min(2)
(c) 483. Expression in response to alkali: 10,20,40,60,80,100 min(3)
(c) 483. Expression in response to alkali: 10,20,40,60,80,100 min(4)
(c) 483. Expression in response to alkali: 10,20,40,60,80,100 min(5)
(c) 485. Expression in response to peroxide: 10,20,40,60,120 min(1)
(c) 485. Expression in response to peroxide: 10,20,40,60,120 min(2)
(c) 485. Expression in response to peroxide: 10,20,40,60,120 min(3)
(c) 485. Expression in response to peroxide: 10,20,40,60,120 min(4)
(c) 519. Brown enviromental changes :29C +1M sorbitol to 33C + 1M sorbitol - 30 minutes(1)
(c) 534. Brown enviromental changes :1 mM Menadione (20 min) redo(1)
(c) 546. Brown enviromental changes :2.5mM DTT 060 min dtt-1(1)
(c) 573. Brown enviromental changes :Hypo-osmotic shock - 15 min(1)
(c) 574. Brown enviromental changes :Hypo-osmotic shock - 30 min(1)
(c) DES460 (wild type) + heat 20 min
(c) wt+gal

RIM101 -*-> SMA1

(c) 5. Expression during the cell cycle (alpha factor arrest and release)(9)
(c) 6. Expression during the cell cycle (cdc15 arrest and release)(7)
(c) 7. Expression during the cell Cycle (cdc28)(2)
(c) 48. Expression in response to 0.15,0.5,1.5,5,15.8,50,158,500 nM alpha-factor(1)
(c) 49. Expression in response to 50 nM alpha-factor: 0,15,30,45,60,90,120 min(2)
(c) 401. Rosetta 2000: Expression in response to HU(1)
(c) 525. Brown enviromental changes :constant 0.32 mM H2O2 (30 min) redo(1)
(c) 531. Brown enviromental changes :constant 0.32 mM H2O2 (120 min) redo(1)
(c) 565. Brown enviromental changes :1M sorbitol - 5 min(1)
(c) 566. Brown enviromental changes :1M sorbitol - 15 min(1)
(c) 569. Brown enviromental changes :1M sorbitol - 60 min(1)
(c) 570. Brown enviromental changes :1M sorbitol - 90 min(1)
(c) 628. Brown enviromental changes :DBY7286 + 0.3 mM H2O2 (20 min)(1)
(c) MAC1-up (C)
(c) wt-gal

RIM101 -*-> YER184C

(c) 5. Expression during the cell cycle (alpha factor arrest and release)(9)
(c) 6. Expression during the cell cycle (cdc15 arrest and release)(7)
(c) 7. Expression during the cell Cycle (cdc28)(2)
(c) 48. Expression in response to 0.15,0.5,1.5,5,15.8,50,158,500 nM alpha-factor(1)
(c) 49. Expression in response to 50 nM alpha-factor: 0,15,30,45,60,90,120 min(2)
(c) 401. Rosetta 2000: Expression in response to HU(1)

(c) 525. Brown enviromental changes :constant 0.32 mM H2O2 (30 min) redo(1)
(c) 531. Brown enviromental changes :constant 0.32 mM H2O2 (120 min) redo(1)
(c) 565. Brown enviromental changes :1M sorbitol - 5 min(1)
(c) 566. Brown enviromental changes :1M sorbitol - 15 min(1)
(c) 569. Brown enviromental changes :1M sorbitol - 60 min(1)
(c) 570. Brown enviromental changes :1M sorbitol - 90 min(1)
(c) 628. Brown enviromental changes :DBY7286 + 0.3 mM H2O2 (20 min)(1)
(c) MAC1-up (C)
(c) wt-gal

ROX1 -*-> ACN9

(c) 5. Expression during the cell cycle (alpha factor arrest and release)(7)
(c) 6. Expression during the cell cycle (cdc15 arrest and release)(19)
(c) 8. Expression during the cell cycle (cell size selection and release)(13)
(c) 49. Expression in response to 50 nM alpha-factor: 0,15,30,45,60,90,120 min(7)
(c) 391. Rosetta 2000: Expression in cells with KAR2 under tet promoter(1)
(c) 479. Expression in diploid cells in response to rapamycin (100nM) for: 15min,30min,90min,120min(3)
(c) 501. Brown enviromental changes :Heat Shock 030inutes hs-2(1)
(c) 545. Brown enviromental changes :2.5mM DTT 045 min dtt-1(1)
(c) 547. Brown enviromental changes :2.5mM DTT 090 min dtt-1(1)
(c) 561. Brown enviromental changes :1.5 mM diamide (40 min)(1)
(c) 562. Brown enviromental changes :1.5 mM diamide (50 min)(1)
(c) 563. Brown enviromental changes :1.5 mM diamide (60 min)(1)
(c) 564. Brown enviromental changes :1.5 mM diamide (90 min)(1)
(c) 611. Brown enviromental changes :YPD stationary phase 2 h ypd-1(1)
(c) 623. Brown enviromental changes :DBY7286 37degree heat - 20 min(1)
(c) 628. Brown enviromental changes :DBY7286 + 0.3 mM H2O2 (20 min)(1)
(c) (Var.) Rich Media 2% Glucose YPD-Average wt 5mM aF, 30 min.
(c) DES460 + 0.02% MMS - 5 min
(c) DES460 + 0.02% MMS - 15 min
(c) DES460 + 0.02% MMS - 60 min
(c) 100 microM BCS 60 min
(c) 100 microM CuSO4 30 min
(c) 100 microM CuSO4 30 min (B)
(c) MAC1-up (C)

ROX1 -*-| PDI1

(c) 5. Expression during the cell cycle (alpha factor arrest and release)(7)
(c) 6. Expression during the cell cycle (cdc15 arrest and release)(19)
(c) 8. Expression during the cell cycle (cell size selection and release)(13)
(c) 49. Expression in response to 50 nM alpha-factor: 0,15,30,45,60,90,120 min(7)
(c) 391. Rosetta 2000: Expression in cells with KAR2 under tet promoter(1)
(c) 479. Expression in diploid cells in response to rapamycin (100nM) for: 15min,30min,90min,120min(3)
(c) 501. Brown enviromental changes :Heat Shock 030inutes hs-2(1)
(c) 545. Brown enviromental changes :2.5mM DTT 045 min dtt-1(1)
(c) 547. Brown enviromental changes :2.5mM DTT 090 min dtt-1(1)
(c) 561. Brown enviromental changes :1.5 mM diamide (40 min)(1)
(c) 562. Brown enviromental changes :1.5 mM diamide (50 min)(1)
(c) 563. Brown enviromental changes :1.5 mM diamide (60 min)(1)
(c) 564. Brown enviromental changes :1.5 mM diamide (90 min)(1)
(c) 611. Brown enviromental changes :YPD stationary phase 2 h ypd-1(1)
(c) 623. Brown enviromental changes :DBY7286 37degree heat - 20 min(1)
(c) 628. Brown enviromental changes :DBY7286 + 0.3 mM H2O2 (20 min)(1)
(c) (Var.) Rich Media 2% Glucose YPD-Average wt 5mM aF, 30 min.
(c) DES460 + 0.02% MMS - 5 min
(c) DES460 + 0.02% MMS - 15 min
(c) DES460 + 0.02% MMS - 60 min
(c) 100 microM BCS 60 min
(c) 100 microM CuSO4 30 min
(c) 100 microM CuSO4 30 min (B)
(c) MAC1-up (C)

RPN4 -*-> PRE1

(c) 6. Expression during the cell cycle (cdc15 arrest and release)(22)
(c) 6. Expression during the cell cycle (cdc15 arrest and release)(23)
(c) 6. Expression during the cell cycle (cdc15 arrest and release)(24)
(c) 7. Expression during the cell Cycle (cdc28)(10)
(c) 7. Expression during the cell Cycle (cdc28)(11)
(c) 8. Expression during the cell cycle (cell size selection and release)(2)
(c) 445. Expression in response to 0.1% MMS for 60 min (average of 3 experiments)(1)
(c) 447. Expression in response to 0.1% MMS for 30 min(1)
(c) 448. Expression in response to 0.1% MMS for 60 min(1)
(c) 449. Expression in response to 0.1% MMS for 60 min(1)
(c) 450. Expression in response to low MNNG (8 microgram/ml) for 60 min(1)
(c) 451. Expression in response to BCNU (200 micromolar) for 60 min(1)
(c) 462. Expression in response to 0.05% MMS for 60 min(1)
(c) 463. Expression in response to 0.1% MMS for 60 min(1)
(c) 464. Expression in response to 0.2% MMS for 60 min(1)
(c) 479. Expression in diploid cells in response to rapamycin (100nM) for: 15min,30min,90min,120min(2)
(c) 481. Expression in response to heat shock: 15,30,45,60,120 min(1)
(c) 481. Expression in response to heat shock: 15,30,45,60,120 min(2)
(c) 481. Expression in response to heat shock: 15,30,45,60,120 min(3)
(c) 481. Expression in response to heat shock: 15,30,45,60,120 min(4)
(c) 561. Brown enviromental changes :1.5 mM diamide (40 min)(1)
(c) 562. Brown enviromental changes :1.5 mM diamide (50 min)(1)
(c) 563. Brown enviromental changes :1.5 mM diamide (60 min)(1)
(c) 685. Expression in response to 0.8M NaCl for 20 min in wild type(1)
(c) DES460 + 0.02% MMS - 5 min
(c) DES460 + 0.02% MMS - 30 min
(c) DES460 + 0.2% MMS - 45 min
(c) DES460 + 0.02% MMS - 60 min
(c) DES460 + 0.02% MMS - 90 min
(c) DES460 + 0.02% MMS - 120 min
(c) wt_plus_gamma_10_min
(c) wt_plus_gamma_30_min
(c) DES460 (wild type) + heat 20 min

RPN4 -*-> PRE2

(c) 6. Expression during the cell cycle (cdc15 arrest and release)(22)
(c) 6. Expression during the cell cycle (cdc15 arrest and release)(23)

```
    (c) 6. Expression during the cell cycle (cdc15 arrest and release)(24)
    (c) 7. Expression during the cell Cycle (cdc28)(10)
    (c) 7. Expression during the cell Cycle (cdc28)(11)
    (c) 8. Expression during the cell cycle (cell size selection and release)(2)
    (c) 445. Expression in response to 0.1% MMS for 60 min (average of 3 experiments)(1)
    (c) 447. Expression in response to 0.1% MMS for 30 min(1)
    (c) 448. Expression in response to 0.1% MMS for 60 min(1)
    (c) 449. Expression in response to 0.1% MMS for 60 min(1)
    (c) 450. Expression in response to low MNNG (8 microgram/ml) for 60 min(1)
    (c) 451. Expression in response to BCNU (200 micromolar) for 60 min(1)
    (c) 462. Expression in response to 0.05% MMS for 60 min(1)
    (c) 463. Expression in response to 0.1% MMS for 60 min(1)
    (c) 464. Expression in response to 0.2% MMS for 60 min(1)
    (c) 479. Expression in diploid cells in response to rapamycin (100nM) for: 15min,30min,90min,120min(2)
    (c) 481. Expression in response to heat shock: 15,30,45,60,120 min(1)
    (c) 481. Expression in response to heat shock: 15,30,45,60,120 min(2)
    (c) 481. Expression in response to heat shock: 15,30,45,60,120 min(3)
    (c) 481. Expression in response to heat shock: 15,30,45,60,120 min(4)
    (c) 561. Brown enviromental changes :1.5 mM diamide (40 min)(1)
    (c) 562. Brown enviromental changes :1.5 mM diamide (50 min)(1)
    (c) 563. Brown enviromental changes :1.5 mM diamide (60 min)(1)
    (c) 685. Expression in response to 0.8M NaCl for 20 min in wild type(1)
    (c) DES460 + 0.02% MMS - 5 min
    (c) DES460 + 0.02% MMS - 30 min
    (c) DES460 + 0.2% MMS - 45 min
    (c) DES460 + 0.02% MMS - 60 min
    (c) DES460 + 0.02% MMS - 90 min
    (c) DES460 + 0.02% MMS - 120 min
    (c) wt_plus_gamma_10_min
    (c) wt_plus_gamma_30_min
    (c) DES460 (wild type) + heat 20 min


RPN4 -*-> PRE3


    (c) 6. Expression during the cell cycle (cdc15 arrest and release)(22)
    (c) 6. Expression during the cell cycle (cdc15 arrest and release)(23)
    (c) 6. Expression during the cell cycle (cdc15 arrest and release)(24)
    (c) 7. Expression during the cell Cycle (cdc28)(10)
    (c) 7. Expression during the cell Cycle (cdc28)(11)
    (c) 8. Expression during the cell cycle (cell size selection and release)(2)
    (c) 445. Expression in response to 0.1% MMS for 60 min (average of 3 experiments)(1)
    (c) 447. Expression in response to 0.1% MMS for 30 min(1)
    (c) 448. Expression in response to 0.1% MMS for 60 min(1)
    (c) 449. Expression in response to 0.1% MMS for 60 min(1)
    (c) 450. Expression in response to low MNNG (8 microgram/ml) for 60 min(1)
    (c) 451. Expression in response to BCNU (200 micromolar) for 60 min(1)
    (c) 462. Expression in response to 0.05% MMS for 60 min(1)
    (c) 463. Expression in response to 0.1% MMS for 60 min(1)
    (c) 464. Expression in response to 0.2% MMS for 60 min(1)
    (c) 479. Expression in diploid cells in response to rapamycin (100nM) for: 15min,30min,90min,120min(2)
    (c) 481. Expression in response to heat shock: 15,30,45,60,120 min(1)
    (c) 481. Expression in response to heat shock: 15,30,45,60,120 min(2)
    (c) 481. Expression in response to heat shock: 15,30,45,60,120 min(3)
    (c) 481. Expression in response to heat shock: 15,30,45,60,120 min(4)
    (c) 561. Brown enviromental changes :1.5 mM diamide (40 min)(1)
    (c) 562. Brown enviromental changes :1.5 mM diamide (50 min)(1)
    (c) 563. Brown enviromental changes :1.5 mM diamide (60 min)(1)
    (c) 685. Expression in response to 0.8M NaCl for 20 min in wild type(1)
    (c) DES460 + 0.02% MMS - 5 min
    (c) DES460 + 0.02% MMS - 30 min
    (c) DES460 + 0.2% MMS - 45 min
    (c) DES460 + 0.02% MMS - 60 min
    (c) DES460 + 0.02% MMS - 90 min
    (c) DES460 + 0.02% MMS - 120 min
    (c) wt_plus_gamma_10_min
    (c) wt_plus_gamma_30_min
    (c) DES460 (wild type) + heat 20 min


RPN4 -*-> PRE8


    (c) 6. Expression during the cell cycle (cdc15 arrest and release)(22)
    (c) 6. Expression during the cell cycle (cdc15 arrest and release)(23)
    (c) 6. Expression during the cell cycle (cdc15 arrest and release)(24)
    (c) 7. Expression during the cell Cycle (cdc28)(10)
    (c) 7. Expression during the cell Cycle (cdc28)(11)
    (c) 8. Expression during the cell cycle (cell size selection and release)(2)
    (c) 445. Expression in response to 0.1% MMS for 60 min (average of 3 experiments)(1)
    (c) 447. Expression in response to 0.1% MMS for 30 min(1)
    (c) 448. Expression in response to 0.1% MMS for 60 min(1)
    (c) 449. Expression in response to 0.1% MMS for 60 min(1)
    (c) 450. Expression in response to low MNNG (8 microgram/ml) for 60 min(1)
    (c) 451. Expression in response to BCNU (200 micromolar) for 60 min(1)
    (c) 462. Expression in response to 0.05% MMS for 60 min(1)
    (c) 463. Expression in response to 0.1% MMS for 60 min(1)
    (c) 464. Expression in response to 0.2% MMS for 60 min(1)
    (c) 479. Expression in diploid cells in response to rapamycin (100nM) for: 15min,30min,90min,120min(2)
    (c) 481. Expression in response to heat shock: 15,30,45,60,120 min(1)
    (c) 481. Expression in response to heat shock: 15,30,45,60,120 min(2)
    (c) 481. Expression in response to heat shock: 15,30,45,60,120 min(3)
    (c) 481. Expression in response to heat shock: 15,30,45,60,120 min(4)
    (c) 561. Brown enviromental changes :1.5 mM diamide (40 min)(1)
    (c) 562. Brown enviromental changes :1.5 mM diamide (50 min)(1)
    (c) 563. Brown enviromental changes :1.5 mM diamide (60 min)(1)
    (c) 685. Expression in response to 0.8M NaCl for 20 min in wild type(1)
    (c) DES460 + 0.02% MMS - 5 min
    (c) DES460 + 0.02% MMS - 30 min
    (c) DES460 + 0.2% MMS - 45 min
    (c) DES460 + 0.02% MMS - 60 min
    (c) DES460 + 0.02% MMS - 90 min
    (c) DES460 + 0.02% MMS - 120 min
    (c) wt_plus_gamma_10_min
    (c) wt_plus_gamma_30_min
    (c) DES460 (wild type) + heat 20 min


RPN4 -*-> PUP1
```

(c) 6. Expression during the cell cycle (cdc15 arrest and release)(22)
(c) 6. Expression during the cell cycle (cdc15 arrest and release)(23)
(c) 6. Expression during the cell cycle (cdc15 arrest and release)(24)
(c) 7. Expression during the cell Cycle (cdc28)(10)
(c) 7. Expression during the cell Cycle (cdc28)(11)
(c) 8. Expression during the cell cycle (cell size selection and release)(2)
(c) 445. Expression in response to 0.1% MMS for 60 min (average of 3 experiments)(1)
(c) 447. Expression in response to 0.1% MMS for 30 min(1)
(c) 448. Expression in response to 0.1% MMS for 60 min(1)
(c) 449. Expression in response to 0.1% MMS for 60 min(1)
(c) 450. Expression in response to low MNNG (8 microgram/ml) for 60 min(1)
(c) 451. Expression in response to BCNU (200 micromolar) for 60 min(1)
(c) 462. Expression in response to 0.05% MMS for 60 min(1)
(c) 463. Expression in response to 0.1% MMS for 60 min(1)
(c) 464. Expression in response to 0.2% MMS for 60 min(1)
(c) 479. Expression in diploid cells in response to rapamycin (100nM) for: 15min,30min,90min,120min(2)
(c) 481. Expression in response to heat shock: 15,30,45,60,120 min(1)
(c) 481. Expression in response to heat shock: 15,30,45,60,120 min(2)
(c) 481. Expression in response to heat shock: 15,30,45,60,120 min(3)
(c) 481. Expression in response to heat shock: 15,30,45,60,120 min(4)
(c) 561. Brown enviromental changes :1.5 mM diamide (40 min)(1)
(c) 562. Brown enviromental changes :1.5 mM diamide (50 min)(1)
(c) 563. Brown enviromental changes :1.5 mM diamide (60 min)(1)
(c) 685. Expression in response to 0.8M NaCl for 20 min in wild type(1)
(c) DES460 + 0.02% MMS - 5 min
(c) DES460 + 0.02% MMS - 30 min
(c) DES460 + 0.2% MMS - 45 min
(c) DES460 + 0.02% MMS - 60 min
(c) DES460 + 0.02% MMS - 90 min
(c) DES460 + 0.02% MMS - 120 min
(c) wt_plus_gamma_10_min
(c) wt_plus_gamma_30_min
(c) DES460 (wild type) + heat 20 min

RPN4 -*-> PUP2

(c) 6. Expression during the cell cycle (cdc15 arrest and release)(22)
(c) 6. Expression during the cell cycle (cdc15 arrest and release)(23)
(c) 6. Expression during the cell cycle (cdc15 arrest and release)(24)
(c) 7. Expression during the cell Cycle (cdc28)(10)
(c) 7. Expression during the cell Cycle (cdc28)(11)
(c) 8. Expression during the cell cycle (cell size selection and release)(2)
(c) 445. Expression in response to 0.1% MMS for 60 min (average of 3 experiments)(1)
(c) 447. Expression in response to 0.1% MMS for 30 min(1)
(c) 448. Expression in response to 0.1% MMS for 60 min(1)
(c) 449. Expression in response to 0.1% MMS for 60 min(1)
(c) 450. Expression in response to low MNNG (8 microgram/ml) for 60 min(1)
(c) 451. Expression in response to BCNU (200 micromolar) for 60 min(1)
(c) 462. Expression in response to 0.05% MMS for 60 min(1)
(c) 463. Expression in response to 0.1% MMS for 60 min(1)
(c) 464. Expression in response to 0.2% MMS for 60 min(1)
(c) 479. Expression in diploid cells in response to rapamycin (100nM) for: 15min,30min,90min,120min(2)
(c) 481. Expression in response to heat shock: 15,30,45,60,120 min(1)
(c) 481. Expression in response to heat shock: 15,30,45,60,120 min(2)
(c) 481. Expression in response to heat shock: 15,30,45,60,120 min(3)
(c) 481. Expression in response to heat shock: 15,30,45,60,120 min(4)
(c) 561. Brown enviromental changes :1.5 mM diamide (40 min)(1)
(c) 562. Brown enviromental changes :1.5 mM diamide (50 min)(1)
(c) 563. Brown enviromental changes :1.5 mM diamide (60 min)(1)
(c) 685. Expression in response to 0.8M NaCl for 20 min in wild type(1)
(c) DES460 + 0.02% MMS - 5 min
(c) DES460 + 0.02% MMS - 30 min
(c) DES460 + 0.2% MMS - 45 min
(c) DES460 + 0.02% MMS - 60 min
(c) DES460 + 0.02% MMS - 90 min
(c) DES460 + 0.02% MMS - 120 min
(c) wt_plus_gamma_10_min
(c) wt_plus_gamma_30_min
(c) DES460 (wild type) + heat 20 min

RPN4 -*-> RPN12

(c) 6. Expression during the cell cycle (cdc15 arrest and release)(22)
(c) 6. Expression during the cell cycle (cdc15 arrest and release)(23)
(c) 6. Expression during the cell cycle (cdc15 arrest and release)(24)
(c) 7. Expression during the cell Cycle (cdc28)(10)
(c) 7. Expression during the cell Cycle (cdc28)(11)
(c) 8. Expression during the cell cycle (cell size selection and release)(2)
(c) 445. Expression in response to 0.1% MMS for 60 min (average of 3 experiments)(1)
(c) 447. Expression in response to 0.1% MMS for 30 min(1)
(c) 448. Expression in response to 0.1% MMS for 60 min(1)
(c) 449. Expression in response to 0.1% MMS for 60 min(1)
(c) 450. Expression in response to low MNNG (8 microgram/ml) for 60 min(1)
(c) 451. Expression in response to BCNU (200 micromolar) for 60 min(1)
(c) 462. Expression in response to 0.05% MMS for 60 min(1)
(c) 463. Expression in response to 0.1% MMS for 60 min(1)
(c) 464. Expression in response to 0.2% MMS for 60 min(1)
(c) 479. Expression in diploid cells in response to rapamycin (100nM) for: 15min,30min,90min,120min(2)
(c) 481. Expression in response to heat shock: 15,30,45,60,120 min(1)
(c) 481. Expression in response to heat shock: 15,30,45,60,120 min(2)
(c) 481. Expression in response to heat shock: 15,30,45,60,120 min(3)
(c) 481. Expression in response to heat shock: 15,30,45,60,120 min(4)
(c) 561. Brown enviromental changes :1.5 mM diamide (40 min)(1)
(c) 562. Brown enviromental changes :1.5 mM diamide (50 min)(1)
(c) 563. Brown enviromental changes :1.5 mM diamide (60 min)(1)
(c) 685. Expression in response to 0.8M NaCl for 20 min in wild type(1)
(c) DES460 + 0.02% MMS - 5 min
(c) DES460 + 0.02% MMS - 30 min
(c) DES460 + 0.2% MMS - 45 min
(c) DES460 + 0.02% MMS - 60 min
(c) DES460 + 0.02% MMS - 90 min
(c) DES460 + 0.02% MMS - 120 min
(c) wt_plus_gamma_10_min
(c) wt_plus_gamma_30_min
(c) DES460 (wild type) + heat 20 min

RPN4 -*-> RPN6

(c) 6. Expression during the cell cycle (cdc15 arrest and release)(22)
(c) 6. Expression during the cell cycle (cdc15 arrest and release)(23)
(c) 6. Expression during the cell cycle (cdc15 arrest and release)(24)
(c) 7. Expression during the cell Cycle (cdc28)(10)
(c) 7. Expression during the cell Cycle (cdc28)(11)
(c) 8. Expression during the cell cycle (cell size selection and release)(2)
(c) 445. Expression in response to 0.1% MMS for 60 min (average of 3 experiments)(1)
(c) 447. Expression in response to 0.1% MMS for 30 min(1)
(c) 448. Expression in response to 0.1% MMS for 60 min(1)
(c) 449. Expression in response to 0.1% MMS for 60 min(1)
(c) 450. Expression in response to low MNNG (8 microgram/ml) for 60 min(1)
(c) 451. Expression in response to BCNU (200 micromolar) for 60 min(1)
(c) 462. Expression in response to 0.05% MMS for 60 min(1)
(c) 463. Expression in response to 0.1% MMS for 60 min(1)
(c) 464. Expression in response to 0.2% MMS for 60 min(1)
(c) 479. Expression in diploid cells in response to rapamycin (100nM) for: 15min,30min,90min,120min(2)
(c) 481. Expression in response to heat shock: 15,30,45,60,120 min(1)
(c) 481. Expression in response to heat shock: 15,30,45,60,120 min(2)
(c) 481. Expression in response to heat shock: 15,30,45,60,120 min(3)
(c) 481. Expression in response to heat shock: 15,30,45,60,120 min(4)
(c) 561. Brown enviromental changes :1.5 mM diamide (40 min)(1)
(c) 562. Brown enviromental changes :1.5 mM diamide (50 min)(1)
(c) 563. Brown enviromental changes :1.5 mM diamide (60 min)(1)
(c) 685. Expression in response to 0.8M NaCl for 20 min in wild type(1)
(c) DES460 + 0.02% MMS - 5 min
(c) DES460 + 0.02% MMS - 30 min
(c) DES460 + 0.2% MMS - 45 min
(c) DES460 + 0.02% MMS - 60 min
(c) DES460 + 0.02% MMS - 90 min
(c) DES460 + 0.02% MMS - 120 min
(c) wt_plus_gamma_10_min
(c) wt_plus_gamma_30_min
(c) DES460 (wild type) + heat 20 min

RPN4 -*-> RPN7

(c) 6. Expression during the cell cycle (cdc15 arrest and release)(22)
(c) 6. Expression during the cell cycle (cdc15 arrest and release)(23)
(c) 6. Expression during the cell cycle (cdc15 arrest and release)(24)
(c) 7. Expression during the cell Cycle (cdc28)(10)
(c) 7. Expression during the cell Cycle (cdc28)(11)
(c) 8. Expression during the cell cycle (cell size selection and release)(2)
(c) 445. Expression in response to 0.1% MMS for 60 min (average of 3 experiments)(1)
(c) 447. Expression in response to 0.1% MMS for 30 min(1)
(c) 448. Expression in response to 0.1% MMS for 60 min(1)
(c) 449. Expression in response to 0.1% MMS for 60 min(1)
(c) 450. Expression in response to low MNNG (8 microgram/ml) for 60 min(1)
(c) 451. Expression in response to BCNU (200 micromolar) for 60 min(1)
(c) 462. Expression in response to 0.05% MMS for 60 min(1)
(c) 463. Expression in response to 0.1% MMS for 60 min(1)
(c) 464. Expression in response to 0.2% MMS for 60 min(1)
(c) 479. Expression in diploid cells in response to rapamycin (100nM) for: 15min,30min,90min,120min(2)
(c) 481. Expression in response to heat shock: 15,30,45,60,120 min(1)
(c) 481. Expression in response to heat shock: 15,30,45,60,120 min(2)
(c) 481. Expression in response to heat shock: 15,30,45,60,120 min(3)
(c) 481. Expression in response to heat shock: 15,30,45,60,120 min(4)
(c) 561. Brown enviromental changes :1.5 mM diamide (40 min)(1)
(c) 562. Brown enviromental changes :1.5 mM diamide (50 min)(1)
(c) 563. Brown enviromental changes :1.5 mM diamide (60 min)(1)
(c) 685. Expression in response to 0.8M NaCl for 20 min in wild type(1)
(c) DES460 + 0.02% MMS - 5 min
(c) DES460 + 0.02% MMS - 30 min
(c) DES460 + 0.2% MMS - 45 min
(c) DES460 + 0.02% MMS - 60 min
(c) DES460 + 0.02% MMS - 90 min
(c) DES460 + 0.02% MMS - 120 min
(c) wt_plus_gamma_10_min
(c) wt_plus_gamma_30_min
(c) DES460 (wild type) + heat 20 min

RPN4 -*-> RPT1

(c) 6. Expression during the cell cycle (cdc15 arrest and release)(22)
(c) 6. Expression during the cell cycle (cdc15 arrest and release)(23)
(c) 6. Expression during the cell cycle (cdc15 arrest and release)(24)
(c) 7. Expression during the cell Cycle (cdc28)(10)
(c) 7. Expression during the cell Cycle (cdc28)(11)
(c) 8. Expression during the cell cycle (cell size selection and release)(2)
(c) 445. Expression in response to 0.1% MMS for 60 min (average of 3 experiments)(1)
(c) 447. Expression in response to 0.1% MMS for 30 min(1)
(c) 448. Expression in response to 0.1% MMS for 60 min(1)
(c) 449. Expression in response to 0.1% MMS for 60 min(1)
(c) 450. Expression in response to low MNNG (8 microgram/ml) for 60 min(1)
(c) 451. Expression in response to BCNU (200 micromolar) for 60 min(1)
(c) 462. Expression in response to 0.05% MMS for 60 min(1)
(c) 463. Expression in response to 0.1% MMS for 60 min(1)
(c) 464. Expression in response to 0.2% MMS for 60 min(1)
(c) 479. Expression in diploid cells in response to rapamycin (100nM) for: 15min,30min,90min,120min(2)
(c) 481. Expression in response to heat shock: 15,30,45,60,120 min(1)
(c) 481. Expression in response to heat shock: 15,30,45,60,120 min(2)
(c) 481. Expression in response to heat shock: 15,30,45,60,120 min(3)
(c) 481. Expression in response to heat shock: 15,30,45,60,120 min(4)
(c) 561. Brown enviromental changes :1.5 mM diamide (40 min)(1)
(c) 562. Brown enviromental changes :1.5 mM diamide (50 min)(1)
(c) 563. Brown enviromental changes :1.5 mM diamide (60 min)(1)
(c) 685. Expression in response to 0.8M NaCl for 20 min in wild type(1)
(c) DES460 + 0.02% MMS - 5 min
(c) DES460 + 0.02% MMS - 30 min
(c) DES460 + 0.2% MMS - 45 min
(c) DES460 + 0.02% MMS - 60 min
(c) DES460 + 0.02% MMS - 90 min
(c) DES460 + 0.02% MMS - 120 min
(c) wt_plus_gamma_10_min
(c) wt_plus_gamma_30_min
(c) DES460 (wild type) + heat 20 min

RPN4 -*-> RPT3

(c) 6. Expression during the cell cycle (cdc15 arrest and release)(22)
(c) 6. Expression during the cell cycle (cdc15 arrest and release)(23)
(c) 6. Expression during the cell cycle (cdc15 arrest and release)(24)
(c) 7. Expression during the cell Cycle (cdc28)(10)
(c) 7. Expression during the cell Cycle (cdc28)(11)
(c) 8. Expression during the cell cycle (cell size selection and release)(2)
(c) 445. Expression in response to 0.1% MMS for 60 min (average of 3 experiments)(1)
(c) 447. Expression in response to 0.1% MMS for 30 min(1)
(c) 448. Expression in response to 0.1% MMS for 60 min(1)
(c) 449. Expression in response to 0.1% MMS for 60 min(1)
(c) 450. Expression in response to low MNNG (8 microgram/ml) for 60 min(1)
(c) 451. Expression in response to BCNU (200 micromolar) for 60 min(1)
(c) 462. Expression in response to 0.05% MMS for 60 min(1)
(c) 463. Expression in response to 0.1% MMS for 60 min(1)
(c) 464. Expression in response to 0.2% MMS for 60 min(1)
(c) 479. Expression in diploid cells in response to rapamycin (100nM) for: 15min,30min,90min,120min(2)
(c) 481. Expression in response to heat shock: 15,30,45,60,120 min(1)
(c) 481. Expression in response to heat shock: 15,30,45,60,120 min(2)
(c) 481. Expression in response to heat shock: 15,30,45,60,120 min(3)
(c) 481. Expression in response to heat shock: 15,30,45,60,120 min(4)
(c) 561. Brown enviromental changes :1.5 mM diamide (40 min)(1)
(c) 562. Brown enviromental changes :1.5 mM diamide (50 min)(1)
(c) 563. Brown enviromental changes :1.5 mM diamide (60 min)(1)
(c) 685. Expression in response to 0.8M NaCl for 20 min in wild type(1)
(c) DES460 + 0.02% MMS - 5 min
(c) DES460 + 0.02% MMS - 30 min
(c) DES460 + 0.2% MMS - 45 min
(c) DES460 + 0.02% MMS - 60 min
(c) DES460 + 0.02% MMS - 90 min
(c) DES460 + 0.02% MMS - 120 min
(c) wt_plus_gamma_10_min
(c) wt_plus_gamma_30_min
(c) DES460 (wild type) + heat 20 min

RPN4 -*-> RPT4

(c) 6. Expression during the cell cycle (cdc15 arrest and release)(22)
(c) 6. Expression during the cell cycle (cdc15 arrest and release)(23)
(c) 6. Expression during the cell cycle (cdc15 arrest and release)(24)
(c) 7. Expression during the cell Cycle (cdc28)(10)
(c) 7. Expression during the cell Cycle (cdc28)(11)
(c) 8. Expression during the cell cycle (cell size selection and release)(2)
(c) 445. Expression in response to 0.1% MMS for 60 min (average of 3 experiments)(1)
(c) 447. Expression in response to 0.1% MMS for 30 min(1)
(c) 448. Expression in response to 0.1% MMS for 60 min(1)
(c) 449. Expression in response to 0.1% MMS for 60 min(1)
(c) 450. Expression in response to low MNNG (8 microgram/ml) for 60 min(1)
(c) 451. Expression in response to BCNU (200 micromolar) for 60 min(1)
(c) 462. Expression in response to 0.05% MMS for 60 min(1)
(c) 463. Expression in response to 0.1% MMS for 60 min(1)
(c) 464. Expression in response to 0.2% MMS for 60 min(1)
(c) 479. Expression in diploid cells in response to rapamycin (100nM) for: 15min,30min,90min,120min(2)
(c) 481. Expression in response to heat shock: 15,30,45,60,120 min(1)
(c) 481. Expression in response to heat shock: 15,30,45,60,120 min(2)
(c) 481. Expression in response to heat shock: 15,30,45,60,120 min(3)
(c) 481. Expression in response to heat shock: 15,30,45,60,120 min(4)
(c) 561. Brown enviromental changes :1.5 mM diamide (40 min)(1)
(c) 562. Brown enviromental changes :1.5 mM diamide (50 min)(1)
(c) 563. Brown enviromental changes :1.5 mM diamide (60 min)(1)
(c) 685. Expression in response to 0.8M NaCl for 20 min in wild type(1)
(c) DES460 + 0.02% MMS - 5 min
(c) DES460 + 0.02% MMS - 30 min
(c) DES460 + 0.2% MMS - 45 min
(c) DES460 + 0.02% MMS - 60 min
(c) DES460 + 0.02% MMS - 90 min
(c) DES460 + 0.02% MMS - 120 min
(c) wt_plus_gamma_10_min
(c) wt_plus_gamma_30_min
(c) DES460 (wild type) + heat 20 min

RPN4 -*-> RPT6

(c) 6. Expression during the cell cycle (cdc15 arrest and release)(22)
(c) 6. Expression during the cell cycle (cdc15 arrest and release)(23)
(c) 6. Expression during the cell cycle (cdc15 arrest and release)(24)
(c) 7. Expression during the cell Cycle (cdc28)(10)
(c) 7. Expression during the cell Cycle (cdc28)(11)
(c) 8. Expression during the cell cycle (cell size selection and release)(2)
(c) 445. Expression in response to 0.1% MMS for 60 min (average of 3 experiments)(1)
(c) 447. Expression in response to 0.1% MMS for 30 min(1)
(c) 448. Expression in response to 0.1% MMS for 60 min(1)
(c) 449. Expression in response to 0.1% MMS for 60 min(1)
(c) 450. Expression in response to low MNNG (8 microgram/ml) for 60 min(1)
(c) 451. Expression in response to BCNU (200 micromolar) for 60 min(1)
(c) 462. Expression in response to 0.05% MMS for 60 min(1)
(c) 463. Expression in response to 0.1% MMS for 60 min(1)
(c) 464. Expression in response to 0.2% MMS for 60 min(1)
(c) 479. Expression in diploid cells in response to rapamycin (100nM) for: 15min,30min,90min,120min(2)
(c) 481. Expression in response to heat shock: 15,30,45,60,120 min(1)
(c) 481. Expression in response to heat shock: 15,30,45,60,120 min(2)
(c) 481. Expression in response to heat shock: 15,30,45,60,120 min(3)
(c) 481. Expression in response to heat shock: 15,30,45,60,120 min(4)
(c) 561. Brown enviromental changes :1.5 mM diamide (40 min)(1)
(c) 562. Brown enviromental changes :1.5 mM diamide (50 min)(1)
(c) 563. Brown enviromental changes :1.5 mM diamide (60 min)(1)
(c) 685. Expression in response to 0.8M NaCl for 20 min in wild type(1)
(c) DES460 + 0.02% MMS - 5 min
(c) DES460 + 0.02% MMS - 30 min
(c) DES460 + 0.2% MMS - 45 min
(c) DES460 + 0.02% MMS - 60 min
(c) DES460 + 0.02% MMS - 90 min
(c) DES460 + 0.02% MMS - 120 min
(c) wt_plus_gamma_10_min
(c) wt_plus_gamma_30_min

(c) DES460 (wild type) + heat 20 min

SKN7 -*-| DDR48

(c) 7. Expression during the cell Cycle (cdc28)(10)
(c) 523. Brown enviromental changes :constant 0.32 mM H2O2 (10 min) redo(1)
(c) 524. Brown enviromental changes :constant 0.32 mM H2O2 (20 min) redo(1)
(c) 525. Brown enviromental changes :constant 0.32 mM H2O2 (30 min) redo(1)
(c) 527. Brown enviromental changes :constant 0.32 mM H2O2 (50 min) redo(1)
(c) 528. Brown enviromental changes :constant 0.32 mM H2O2 (60 min) redo(1)
(c) 529. Brown enviromental changes :constant 0.32 mM H2O2 (80 min) redo(1)
(c) 530. Brown enviromental changes :constant 0.32 mM H2O2 (100 min) redo(1)
(c) 531. Brown enviromental changes :constant 0.32 mM H2O2 (120 min) redo(1)
(c) 532. Brown enviromental changes :constant 0.32 mM H2O2 (160 min) redo(1)
(c) 534. Brown enviromental changes :1 mM Menadione (20 min) redo(1)
(c) 535. Brown enviromental changes :1 mM Menadione (30 min) redo(1)
(c) 536. Brown enviromental changes :1mM Menadione (40 min) redo(1)
(c) 537. Brown enviromental changes :1 mM Menadione (50 min)redo(1)
(c) 538. Brown enviromental changes :1 mM Menadione (80 min) redo(1)
(c) 539. Brown enviromental changes :1 mM Menadione (105 min) redo(1)
(c) 541. Brown enviromental changes :1 mM Menadione (160 min) redo(1)
(c) 544. Brown enviromental changes :2.5mM DTT 030 min dtt-1(1)
(c) 545. Brown enviromental changes :2.5mM DTT 045 min dtt-1(1)
(c) 546. Brown enviromental changes :2.5mM DTT 060 min dtt-1(1)
(c) 547. Brown enviromental changes :2.5mM DTT 090 min dtt-1(1)
(c) 548. Brown enviromental changes :2.5mM DTT 120 min dtt-1(1)
(c) 553. Brown enviromental changes :dtt 060 min dtt-2(1)
(c) 557. Brown enviromental changes :1.5 mM diamide (5 min)(1)
(c) 558. Brown enviromental changes :1.5 mM diamide (10 min)(1)
(c) 559. Brown enviromental changes :1.5 mM diamide (20 min)(1)
(c) 560. Brown enviromental changes :1.5 mM diamide (30 min)(1)
(c) 561. Brown enviromental changes :1.5 mM diamide (40 min)(1)
(c) 562. Brown enviromental changes :1.5 mM diamide (50 min)(1)
(c) 563. Brown enviromental changes :1.5 mM diamide (60 min)(1)

SKN7 -*-| GPX2

(c) 7. Expression during the cell Cycle (cdc28)(10)
(c) 523. Brown enviromental changes :constant 0.32 mM H2O2 (10 min) redo(1)
(c) 524. Brown enviromental changes :constant 0.32 mM H2O2 (20 min) redo(1)
(c) 525. Brown enviromental changes :constant 0.32 mM H2O2 (30 min) redo(1)
(c) 527. Brown enviromental changes :constant 0.32 mM H2O2 (50 min) redo(1)
(c) 528. Brown enviromental changes :constant 0.32 mM H2O2 (60 min) redo(1)
(c) 529. Brown enviromental changes :constant 0.32 mM H2O2 (80 min) redo(1)
(c) 530. Brown enviromental changes :constant 0.32 mM H2O2 (100 min) redo(1)
(c) 531. Brown enviromental changes :constant 0.32 mM H2O2 (120 min) redo(1)
(c) 532. Brown enviromental changes :constant 0.32 mM H2O2 (160 min) redo(1)
(c) 534. Brown enviromental changes :1 mM Menadione (20 min) redo(1)
(c) 535. Brown enviromental changes :1 mM Menadione (30 min) redo(1)
(c) 536. Brown enviromental changes :1mM Menadione (40 min) redo(1)
(c) 537. Brown enviromental changes :1 mM Menadione (50 min)redo(1)
(c) 538. Brown enviromental changes :1 mM Menadione (80 min) redo(1)
(c) 539. Brown enviromental changes :1 mM Menadione (105 min) redo(1)
(c) 541. Brown enviromental changes :1 mM Menadione (160 min) redo(1)
(c) 544. Brown enviromental changes :2.5mM DTT 030 min dtt-1(1)
(c) 545. Brown enviromental changes :2.5mM DTT 045 min dtt-1(1)
(c) 546. Brown enviromental changes :2.5mM DTT 060 min dtt-1(1)
(c) 547. Brown enviromental changes :2.5mM DTT 090 min dtt-1(1)
(c) 548. Brown enviromental changes :2.5mM DTT 120 min dtt-1(1)
(c) 553. Brown enviromental changes :dtt 060 min dtt-2(1)
(c) 557. Brown enviromental changes :1.5 mM diamide (5 min)(1)
(c) 558. Brown enviromental changes :1.5 mM diamide (10 min)(1)
(c) 559. Brown enviromental changes :1.5 mM diamide (20 min)(1)
(c) 560. Brown enviromental changes :1.5 mM diamide (30 min)(1)
(c) 561. Brown enviromental changes :1.5 mM diamide (40 min)(1)
(c) 562. Brown enviromental changes :1.5 mM diamide (50 min)(1)
(c) 563. Brown enviromental changes :1.5 mM diamide (60 min)(1)

SKN7 -*-| SOD2

(c) 7. Expression during the cell Cycle (cdc28)(10)
(c) 523. Brown enviromental changes :constant 0.32 mM H2O2 (10 min) redo(1)
(c) 524. Brown enviromental changes :constant 0.32 mM H2O2 (20 min) redo(1)
(c) 525. Brown enviromental changes :constant 0.32 mM H2O2 (30 min) redo(1)
(c) 527. Brown enviromental changes :constant 0.32 mM H2O2 (50 min) redo(1)
(c) 528. Brown enviromental changes :constant 0.32 mM H2O2 (60 min) redo(1)
(c) 529. Brown enviromental changes :constant 0.32 mM H2O2 (80 min) redo(1)
(c) 530. Brown enviromental changes :constant 0.32 mM H2O2 (100 min) redo(1)
(c) 531. Brown enviromental changes :constant 0.32 mM H2O2 (120 min) redo(1)
(c) 532. Brown enviromental changes :constant 0.32 mM H2O2 (160 min) redo(1)
(c) 534. Brown enviromental changes :1 mM Menadione (20 min) redo(1)
(c) 535. Brown enviromental changes :1 mM Menadione (30 min) redo(1)
(c) 536. Brown enviromental changes :1mM Menadione (40 min) redo(1)
(c) 537. Brown enviromental changes :1 mM Menadione (50 min)redo(1)
(c) 538. Brown enviromental changes :1 mM Menadione (80 min) redo(1)
(c) 539. Brown enviromental changes :1 mM Menadione (105 min) redo(1)
(c) 541. Brown enviromental changes :1 mM Menadione (160 min) redo(1)
(c) 544. Brown enviromental changes :2.5mM DTT 030 min dtt-1(1)
(c) 545. Brown enviromental changes :2.5mM DTT 045 min dtt-1(1)
(c) 546. Brown enviromental changes :2.5mM DTT 060 min dtt-1(1)
(c) 547. Brown enviromental changes :2.5mM DTT 090 min dtt-1(1)
(c) 548. Brown enviromental changes :2.5mM DTT 120 min dtt-1(1)
(c) 553. Brown enviromental changes :dtt 060 min dtt-2(1)
(c) 557. Brown enviromental changes :1.5 mM diamide (5 min)(1)
(c) 558. Brown enviromental changes :1.5 mM diamide (10 min)(1)
(c) 559. Brown enviromental changes :1.5 mM diamide (20 min)(1)
(c) 560. Brown enviromental changes :1.5 mM diamide (30 min)(1)
(c) 561. Brown enviromental changes :1.5 mM diamide (40 min)(1)
(c) 562. Brown enviromental changes :1.5 mM diamide (50 min)(1)
(c) 563. Brown enviromental changes :1.5 mM diamide (60 min)(1)

SKN7 -*-| YDL124W

(c) 7. Expression during the cell Cycle (cdc28)(10)

(c) 523. Brown enviromental changes :constant 0.32 mM H2O2 (10 min) redo(1)
(c) 524. Brown enviromental changes :constant 0.32 mM H2O2 (20 min) redo(1)
(c) 525. Brown enviromental changes :constant 0.32 mM H2O2 (30 min) redo(1)
(c) 527. Brown enviromental changes :constant 0.32 mM H2O2 (50 min) redo(1)
(c) 528. Brown enviromental changes :constant 0.32 mM H2O2 (60 min) redo(1)
(c) 529. Brown enviromental changes :constant 0.32 mM H2O2 (80 min) redo(1)
(c) 530. Brown enviromental changes :constant 0.32 mM H2O2 (100 min) redo(1)
(c) 531. Brown enviromental changes :constant 0.32 mM H2O2 (120 min) redo(1)
(c) 532. Brown enviromental changes :constant 0.32 mM H2O2 (160 min) redo(1)
(c) 534. Brown enviromental changes :1 mM Menadione (20 min) redo(1)
(c) 535. Brown enviromental changes :1 mM Menadione (30 min) redo(1)
(c) 536. Brown enviromental changes :1mM Menadione (40 min) redo(1)
(c) 537. Brown enviromental changes :1 mM Menadione (50 min)redo(1)
(c) 538. Brown enviromental changes :1 mM Menadione (80 min) redo(1)
(c) 539. Brown enviromental changes :1 mM Menadione (105 min) redo(1)
(c) 541. Brown enviromental changes :1 mM Menadione (160 min) redo(1)
(c) 544. Brown enviromental changes :2.5mM DTT 030 min dtt-1(1)
(c) 545. Brown enviromental changes :2.5mM DTT 045 min dtt-1(1)
(c) 546. Brown enviromental changes :2.5mM DTT 060 min dtt-1(1)
(c) 547. Brown enviromental changes :2.5mM DTT 090 min dtt-1(1)
(c) 548. Brown enviromental changes :2.5mM DTT 120 min dtt-1(1)
(c) 553. Brown enviromental changes :dtt 060 min dtt-2(1)
(c) 557. Brown enviromental changes :1.5 mM diamide (5 min)(1)
(c) 558. Brown enviromental changes :1.5 mM diamide (10 min)(1)
(c) 559. Brown enviromental changes :1.5 mM diamide (20 min)(1)
(c) 560. Brown enviromental changes :1.5 mM diamide (30 min)(1)
(c) 561. Brown enviromental changes :1.5 mM diamide (40 min)(1)
(c) 562. Brown enviromental changes :1.5 mM diamide (50 min)(1)
(c) 563. Brown enviromental changes :1.5 mM diamide (60 min)(1)

SKN7 -*-| YLR108C

(c) 7. Expression during the cell Cycle (cdc28)(10)
(c) 523. Brown enviromental changes :constant 0.32 mM H2O2 (10 min) redo(1)
(c) 524. Brown enviromental changes :constant 0.32 mM H2O2 (20 min) redo(1)
(c) 525. Brown enviromental changes :constant 0.32 mM H2O2 (30 min) redo(1)
(c) 527. Brown enviromental changes :constant 0.32 mM H2O2 (50 min) redo(1)
(c) 528. Brown enviromental changes :constant 0.32 mM H2O2 (60 min) redo(1)
(c) 529. Brown enviromental changes :constant 0.32 mM H2O2 (80 min) redo(1)
(c) 530. Brown enviromental changes :constant 0.32 mM H2O2 (100 min) redo(1)
(c) 531. Brown enviromental changes :constant 0.32 mM H2O2 (120 min) redo(1)
(c) 532. Brown enviromental changes :constant 0.32 mM H2O2 (160 min) redo(1)
(c) 534. Brown enviromental changes :1 mM Menadione (20 min) redo(1)
(c) 535. Brown enviromental changes :1 mM Menadione (30 min) redo(1)
(c) 536. Brown enviromental changes :1mM Menadione (40 min) redo(1)
(c) 537. Brown enviromental changes :1 mM Menadione (50 min)redo(1)
(c) 538. Brown enviromental changes :1 mM Menadione (80 min) redo(1)
(c) 539. Brown enviromental changes :1 mM Menadione (105 min) redo(1)
(c) 541. Brown enviromental changes :1 mM Menadione (160 min) redo(1)
(c) 544. Brown enviromental changes :2.5mM DTT 030 min dtt-1(1)
(c) 545. Brown enviromental changes :2.5mM DTT 045 min dtt-1(1)
(c) 546. Brown enviromental changes :2.5mM DTT 060 min dtt-1(1)
(c) 547. Brown enviromental changes :2.5mM DTT 090 min dtt-1(1)
(c) 548. Brown enviromental changes :2.5mM DTT 120 min dtt-1(1)
(c) 553. Brown enviromental changes :dtt 060 min dtt-2(1)
(c) 557. Brown enviromental changes :1.5 mM diamide (5 min)(1)
(c) 558. Brown enviromental changes :1.5 mM diamide (10 min)(1)
(c) 559. Brown enviromental changes :1.5 mM diamide (20 min)(1)
(c) 560. Brown enviromental changes :1.5 mM diamide (30 min)(1)
(c) 561. Brown enviromental changes :1.5 mM diamide (40 min)(1)
(c) 562. Brown enviromental changes :1.5 mM diamide (50 min)(1)
(c) 563. Brown enviromental changes :1.5 mM diamide (60 min)(1)

SKN7 -*-| YMR090W

(c) 7. Expression during the cell Cycle (cdc28)(10)
(c) 523. Brown enviromental changes :constant 0.32 mM H2O2 (10 min) redo(1)
(c) 524. Brown enviromental changes :constant 0.32 mM H2O2 (20 min) redo(1)
(c) 525. Brown enviromental changes :constant 0.32 mM H2O2 (30 min) redo(1)
(c) 527. Brown enviromental changes :constant 0.32 mM H2O2 (50 min) redo(1)
(c) 528. Brown enviromental changes :constant 0.32 mM H2O2 (60 min) redo(1)
(c) 529. Brown enviromental changes :constant 0.32 mM H2O2 (80 min) redo(1)
(c) 530. Brown enviromental changes :constant 0.32 mM H2O2 (100 min) redo(1)
(c) 531. Brown enviromental changes :constant 0.32 mM H2O2 (120 min) redo(1)
(c) 532. Brown enviromental changes :constant 0.32 mM H2O2 (160 min) redo(1)
(c) 534. Brown enviromental changes :1 mM Menadione (20 min) redo(1)
(c) 535. Brown enviromental changes :1 mM Menadione (30 min) redo(1)
(c) 536. Brown enviromental changes :1mM Menadione (40 min) redo(1)
(c) 537. Brown enviromental changes :1 mM Menadione (50 min)redo(1)
(c) 538. Brown enviromental changes :1 mM Menadione (80 min) redo(1)
(c) 539. Brown enviromental changes :1 mM Menadione (105 min) redo(1)
(c) 541. Brown enviromental changes :1 mM Menadione (160 min) redo(1)
(c) 544. Brown enviromental changes :2.5mM DTT 030 min dtt-1(1)
(c) 545. Brown enviromental changes :2.5mM DTT 045 min dtt-1(1)
(c) 546. Brown enviromental changes :2.5mM DTT 060 min dtt-1(1)
(c) 547. Brown enviromental changes :2.5mM DTT 090 min dtt-1(1)
(c) 548. Brown enviromental changes :2.5mM DTT 120 min dtt-1(1)
(c) 553. Brown enviromental changes :dtt 060 min dtt-2(1)
(c) 557. Brown enviromental changes :1.5 mM diamide (5 min)(1)
(c) 558. Brown enviromental changes :1.5 mM diamide (10 min)(1)
(c) 559. Brown enviromental changes :1.5 mM diamide (20 min)(1)
(c) 560. Brown enviromental changes :1.5 mM diamide (30 min)(1)
(c) 561. Brown enviromental changes :1.5 mM diamide (40 min)(1)
(c) 562. Brown enviromental changes :1.5 mM diamide (50 min)(1)
(c) 563. Brown enviromental changes :1.5 mM diamide (60 min)(1)

SKN7 -*-| YMR315W

(c) 7. Expression during the cell Cycle (cdc28)(10)
(c) 523. Brown enviromental changes :constant 0.32 mM H2O2 (10 min) redo(1)
(c) 524. Brown enviromental changes :constant 0.32 mM H2O2 (20 min) redo(1)
(c) 525. Brown enviromental changes :constant 0.32 mM H2O2 (30 min) redo(1)
(c) 527. Brown enviromental changes :constant 0.32 mM H2O2 (50 min) redo(1)
(c) 528. Brown enviromental changes :constant 0.32 mM H2O2 (60 min) redo(1)
(c) 529. Brown enviromental changes :constant 0.32 mM H2O2 (80 min) redo(1)
(c) 530. Brown enviromental changes :constant 0.32 mM H2O2 (100 min) redo(1)

(c) 531. Brown enviromental changes :constant 0.32 mM H2O2 (120 min) redo(1)
(c) 532. Brown enviromental changes :constant 0.32 mM H2O2 (160 min) redo(1)
(c) 534. Brown enviromental changes :1 mM Menadione (20 min) redo(1)
(c) 535. Brown enviromental changes :1 mM Menadione (30 min) redo(1)
(c) 536. Brown enviromental changes :1mM Menadione (40 min) redo(1)
(c) 537. Brown enviromental changes :1 mM Menadione (50 min)redo(1)
(c) 538. Brown enviromental changes :1 mM Menadione (80 min) redo(1)
(c) 539. Brown enviromental changes :1 mM Menadione (105 min) redo(1)
(c) 541. Brown enviromental changes :1 mM Menadione (160 min) redo(1)
(c) 544. Brown enviromental changes :2.5mM DTT 030 min dtt-1(1)
(c) 545. Brown enviromental changes :2.5mM DTT 045 min dtt-1(1)
(c) 546. Brown enviromental changes :2.5mM DTT 060 min dtt-1(1)
(c) 547. Brown enviromental changes :2.5mM DTT 090 min dtt-1(1)
(c) 548. Brown enviromental changes :2.5mM DTT 120 min dtt-1(1)
(c) 553. Brown enviromental changes :dtt 060 min dtt-2(1)
(c) 557. Brown enviromental changes :1.5 mM diamide (5 min)(1)
(c) 558. Brown enviromental changes :1.5 mM diamide (10 min)(1)
(c) 559. Brown enviromental changes :1.5 mM diamide (20 min)(1)
(c) 560. Brown enviromental changes :1.5 mM diamide (30 min)(1)
(c) 561. Brown enviromental changes :1.5 mM diamide (40 min)(1)
(c) 562. Brown enviromental changes :1.5 mM diamide (50 min)(1)
(c) 563. Brown enviromental changes :1.5 mM diamide (60 min)(1)

SKO1 -*-> YGL046W

(c) 3. Cell Cycle: Expression in response to Clb2p (set 1, 40 min)(1)
(c) 6. Expression during the cell cycle (cdc15 arrest and release)(20)
(c) 7. Expression during the cell Cycle (cdc28)(16)
(c) 8. Expression during the cell cycle (cell size selection and release)(2)
(c) 11. Expression during diauxic shift: 9h,11h,13h,15h,17h,19h,21h(1)
(c) 11. Expression during diauxic shift: 9h,11h,13h,15h,17h,19h,21h(2)
(c) pho80 vs WT(1)
(c) pho85 vs WT(1)
(c) 496. Brown enviromental changes :Heat Shock 000 minutes hs-2(1)
(c) 497. Brown enviromental changes :Heat Shock 000 minutes hs-2(1)
(c) 519. Brown enviromental changes :29C +1M sorbitol to 33C + 1M sorbitol - 30 minutes(1)
(c) 533. Brown enviromental changes :1 mM Menadione (10 min)redo(1)
(c) 542. Brown enviromental changes :2.5mM DTT 005 min dtt-1(1)
(c) 550. Brown enviromental changes :dtt 000 min dtt-2(1)
(c) 552. Brown enviromental changes :dtt 030 min dtt-2(1)
(c) 571. Brown enviromental changes :1M sorbitol - 120 min(1)
(c) 573. Brown enviromental changes :Hypo-osmotic shock - 15 min(1)
(c) 576. Brown enviromental changes :Hypo-osmotic shock - 60 min(1)
(c) 593. Brown enviromental changes :Diauxic Shift Timecourse(1)
(c) 594. Brown enviromental changes :diauxic shift timecourse(1)
(c) 100 microM CuSO4 30 min

STE12 -*-> ASG7

(c) 5. Expression during the cell cycle (alpha factor arrest and release)(1)
(c) 48. Expression in response to 0.15,0.5,1.5,5,15.8,50,158,500 nM alpha-factor(3)
(c) 48. Expression in response to 0.15,0.5,1.5,5,15.8,50,158,500 nM alpha-factor(4)
(c) 48. Expression in response to 0.15,0.5,1.5,5,15.8,50,158,500 nM alpha-factor(5)
(c) 48. Expression in response to 0.15,0.5,1.5,5,15.8,50,158,500 nM alpha-factor(6)
(c) 48. Expression in response to 0.15,0.5,1.5,5,15.8,50,158,500 nM alpha-factor(7)
(c) 48. Expression in response to 0.15,0.5,1.5,5,15.8,50,158,500 nM alpha-factor(8)
(c) 49. Expression in response to 50 nM alpha-factor: 0,15,30,45,60,90,120 min(1)
(c) 49. Expression in response to 50 nM alpha-factor: 0,15,30,45,60,90,120 min(2)
(c) 49. Expression in response to 50 nM alpha-factor: 0,15,30,45,60,90,120 min(3)
(c) 49. Expression in response to 50 nM alpha-factor: 0,15,30,45,60,90,120 min(4)
(c) 49. Expression in response to 50 nM alpha-factor: 0,15,30,45,60,90,120 min(5)
(c) 49. Expression in response to 50 nM alpha-factor: 0,15,30,45,60,90,120 min(6)
(c) 49. Expression in response to 50 nM alpha-factor: 0,15,30,45,60,90,120 min(7)
(c) 53. Expression in response to overproduction of Ste4p(1)
(c) 54. Expression in response to overproduction of Ste5p(1)
(c) 55. Expression in response to overproduction of Ste11p(1)
(c) 56. Expression in response to overproduction of Ste12p(1)
(c) (Rich Media 2% Glucose YPD-185588) wt 5mM aF, 30 min.
(c) (Rich Media 2% Glucose YPD-185769) wt 5mM aF, 30 min.
(c) Rich Media 2% Glucose YPD-Average wt 5mM aF, 30 min.

STE12 -*-> FUS2

(c) 5. Expression during the cell cycle (alpha factor arrest and release)(1)
(c) 48. Expression in response to 0.15,0.5,1.5,5,15.8,50,158,500 nM alpha-factor(3)
(c) 48. Expression in response to 0.15,0.5,1.5,5,15.8,50,158,500 nM alpha-factor(4)
(c) 48. Expression in response to 0.15,0.5,1.5,5,15.8,50,158,500 nM alpha-factor(5)
(c) 48. Expression in response to 0.15,0.5,1.5,5,15.8,50,158,500 nM alpha-factor(6)
(c) 48. Expression in response to 0.15,0.5,1.5,5,15.8,50,158,500 nM alpha-factor(7)
(c) 48. Expression in response to 0.15,0.5,1.5,5,15.8,50,158,500 nM alpha-factor(8)
(c) 49. Expression in response to 50 nM alpha-factor: 0,15,30,45,60,90,120 min(1)
(c) 49. Expression in response to 50 nM alpha-factor: 0,15,30,45,60,90,120 min(2)
(c) 49. Expression in response to 50 nM alpha-factor: 0,15,30,45,60,90,120 min(3)
(c) 49. Expression in response to 50 nM alpha-factor: 0,15,30,45,60,90,120 min(4)
(c) 49. Expression in response to 50 nM alpha-factor: 0,15,30,45,60,90,120 min(5)
(c) 49. Expression in response to 50 nM alpha-factor: 0,15,30,45,60,90,120 min(6)
(c) 49. Expression in response to 50 nM alpha-factor: 0,15,30,45,60,90,120 min(7)
(c) 53. Expression in response to overproduction of Ste4p(1)
(c) 54. Expression in response to overproduction of Ste5p(1)
(c) 55. Expression in response to overproduction of Ste11p(1)
(c) 56. Expression in response to overproduction of Ste12p(1)
(c) (Rich Media 2% Glucose YPD-185588) wt 5mM aF, 30 min.
(c) (Rich Media 2% Glucose YPD-185769) wt 5mM aF, 30 min.
(c) Rich Media 2% Glucose YPD-Average wt 5mM aF, 30 min.

STE12 -*-> GFA1

(c) 5. Expression during the cell cycle (alpha factor arrest and release)(1)
(c) 48. Expression in response to 0.15,0.5,1.5,5,15.8,50,158,500 nM alpha-factor(3)
(c) 48. Expression in response to 0.15,0.5,1.5,5,15.8,50,158,500 nM alpha-factor(4)
(c) 48. Expression in response to 0.15,0.5,1.5,5,15.8,50,158,500 nM alpha-factor(5)
(c) 48. Expression in response to 0.15,0.5,1.5,5,15.8,50,158,500 nM alpha-factor(6)
(c) 48. Expression in response to 0.15,0.5,1.5,5,15.8,50,158,500 nM alpha-factor(7)
(c) 48. Expression in response to 0.15,0.5,1.5,5,15.8,50,158,500 nM alpha-factor(8)

(c) 49. Expression in response to 50 nM alpha-factor: 0,15,30,45,60,90,120 min(1)
(c) 49. Expression in response to 50 nM alpha-factor: 0,15,30,45,60,90,120 min(2)
(c) 49. Expression in response to 50 nM alpha-factor: 0,15,30,45,60,90,120 min(3)
(c) 49. Expression in response to 50 nM alpha-factor: 0,15,30,45,60,90,120 min(4)
(c) 49. Expression in response to 50 nM alpha-factor: 0,15,30,45,60,90,120 min(5)
(c) 49. Expression in response to 50 nM alpha-factor: 0,15,30,45,60,90,120 min(6)
(c) 49. Expression in response to 50 nM alpha-factor: 0,15,30,45,60,90,120 min(7)
(c) 53. Expression in response to overproduction of Ste4p(1)
(c) 54. Expression in response to overproduction of Ste5p(1)
(c) 55. Expression in response to overproduction of Ste11p(1)
(c) 56. Expression in response to overproduction of Ste12p(1)
(c) (Rich Media 2% Glucose YPD-185588) wt 5mM aF, 30 min.
(c) (Rich Media 2% Glucose YPD-185769) wt 5mM aF, 30 min.
(c) Rich Media 2% Glucose YPD-Average wt 5mM aF, 30 min.

STE12 -*-> GPA1

(c) 5. Expression during the cell cycle (alpha factor arrest and release)(1)
(c) 48. Expression in response to 0.15,0.5,1.5,5,15.8,50,158,500 nM alpha-factor(3)
(c) 48. Expression in response to 0.15,0.5,1.5,5,15.8,50,158,500 nM alpha-factor(4)
(c) 48. Expression in response to 0.15,0.5,1.5,5,15.8,50,158,500 nM alpha-factor(5)
(c) 48. Expression in response to 0.15,0.5,1.5,5,15.8,50,158,500 nM alpha-factor(6)
(c) 48. Expression in response to 0.15,0.5,1.5,5,15.8,50,158,500 nM alpha-factor(7)
(c) 48. Expression in response to 0.15,0.5,1.5,5,15.8,50,158,500 nM alpha-factor(8)
(c) 49. Expression in response to 50 nM alpha-factor: 0,15,30,45,60,90,120 min(1)
(c) 49. Expression in response to 50 nM alpha-factor: 0,15,30,45,60,90,120 min(2)
(c) 49. Expression in response to 50 nM alpha-factor: 0,15,30,45,60,90,120 min(3)
(c) 49. Expression in response to 50 nM alpha-factor: 0,15,30,45,60,90,120 min(4)
(c) 49. Expression in response to 50 nM alpha-factor: 0,15,30,45,60,90,120 min(5)
(c) 49. Expression in response to 50 nM alpha-factor: 0,15,30,45,60,90,120 min(6)
(c) 49. Expression in response to 50 nM alpha-factor: 0,15,30,45,60,90,120 min(7)
(c) 53. Expression in response to overproduction of Ste4p(1)
(c) 54. Expression in response to overproduction of Ste5p(1)
(c) 55. Expression in response to overproduction of Ste11p(1)
(c) 56. Expression in response to overproduction of Ste12p(1)
(c) (Rich Media 2% Glucose YPD-185588) wt 5mM aF, 30 min.
(c) (Rich Media 2% Glucose YPD-185769) wt 5mM aF, 30 min.
(c) Rich Media 2% Glucose YPD-Average wt 5mM aF, 30 min.

STE12 -*-> GYP8

(c) 5. Expression during the cell cycle (alpha factor arrest and release)(1)
(c) 48. Expression in response to 0.15,0.5,1.5,5,15.8,50,158,500 nM alpha-factor(3)
(c) 48. Expression in response to 0.15,0.5,1.5,5,15.8,50,158,500 nM alpha-factor(4)
(c) 48. Expression in response to 0.15,0.5,1.5,5,15.8,50,158,500 nM alpha-factor(5)
(c) 48. Expression in response to 0.15,0.5,1.5,5,15.8,50,158,500 nM alpha-factor(6)
(c) 48. Expression in response to 0.15,0.5,1.5,5,15.8,50,158,500 nM alpha-factor(7)
(c) 48. Expression in response to 0.15,0.5,1.5,5,15.8,50,158,500 nM alpha-factor(8)
(c) 49. Expression in response to 50 nM alpha-factor: 0,15,30,45,60,90,120 min(1)
(c) 49. Expression in response to 50 nM alpha-factor: 0,15,30,45,60,90,120 min(2)
(c) 49. Expression in response to 50 nM alpha-factor: 0,15,30,45,60,90,120 min(3)
(c) 49. Expression in response to 50 nM alpha-factor: 0,15,30,45,60,90,120 min(4)
(c) 49. Expression in response to 50 nM alpha-factor: 0,15,30,45,60,90,120 min(5)
(c) 49. Expression in response to 50 nM alpha-factor: 0,15,30,45,60,90,120 min(6)
(c) 49. Expression in response to 50 nM alpha-factor: 0,15,30,45,60,90,120 min(7)
(c) 53. Expression in response to overproduction of Ste4p(1)
(c) 54. Expression in response to overproduction of Ste5p(1)
(c) 55. Expression in response to overproduction of Ste11p(1)
(c) 56. Expression in response to overproduction of Ste12p(1)
(c) (Rich Media 2% Glucose YPD-185588) wt 5mM aF, 30 min.
(c) (Rich Media 2% Glucose YPD-185769) wt 5mM aF, 30 min.
(c) Rich Media 2% Glucose YPD-Average wt 5mM aF, 30 min.

STE12 -*-> INP52

(c) 5. Expression during the cell cycle (alpha factor arrest and release)(1)
(c) 48. Expression in response to 0.15,0.5,1.5,5,15.8,50,158,500 nM alpha-factor(3)
(c) 48. Expression in response to 0.15,0.5,1.5,5,15.8,50,158,500 nM alpha-factor(4)
(c) 48. Expression in response to 0.15,0.5,1.5,5,15.8,50,158,500 nM alpha-factor(5)
(c) 48. Expression in response to 0.15,0.5,1.5,5,15.8,50,158,500 nM alpha-factor(6)
(c) 48. Expression in response to 0.15,0.5,1.5,5,15.8,50,158,500 nM alpha-factor(7)
(c) 48. Expression in response to 0.15,0.5,1.5,5,15.8,50,158,500 nM alpha-factor(8)
(c) 49. Expression in response to 50 nM alpha-factor: 0,15,30,45,60,90,120 min(1)
(c) 49. Expression in response to 50 nM alpha-factor: 0,15,30,45,60,90,120 min(2)
(c) 49. Expression in response to 50 nM alpha-factor: 0,15,30,45,60,90,120 min(3)
(c) 49. Expression in response to 50 nM alpha-factor: 0,15,30,45,60,90,120 min(4)
(c) 49. Expression in response to 50 nM alpha-factor: 0,15,30,45,60,90,120 min(5)
(c) 49. Expression in response to 50 nM alpha-factor: 0,15,30,45,60,90,120 min(6)
(c) 49. Expression in response to 50 nM alpha-factor: 0,15,30,45,60,90,120 min(7)
(c) 53. Expression in response to overproduction of Ste4p(1)
(c) 54. Expression in response to overproduction of Ste5p(1)
(c) 55. Expression in response to overproduction of Ste11p(1)
(c) 56. Expression in response to overproduction of Ste12p(1)
(c) (Rich Media 2% Glucose YPD-185588) wt 5mM aF, 30 min.
(c) (Rich Media 2% Glucose YPD-185769) wt 5mM aF, 30 min.
(c) Rich Media 2% Glucose YPD-Average wt 5mM aF, 30 min.

STE12 -*-> KAR4

(c) 5. Expression during the cell cycle (alpha factor arrest and release)(1)
(c) 48. Expression in response to 0.15,0.5,1.5,5,15.8,50,158,500 nM alpha-factor(3)
(c) 48. Expression in response to 0.15,0.5,1.5,5,15.8,50,158,500 nM alpha-factor(4)
(c) 48. Expression in response to 0.15,0.5,1.5,5,15.8,50,158,500 nM alpha-factor(5)
(c) 48. Expression in response to 0.15,0.5,1.5,5,15.8,50,158,500 nM alpha-factor(6)
(c) 48. Expression in response to 0.15,0.5,1.5,5,15.8,50,158,500 nM alpha-factor(7)
(c) 48. Expression in response to 0.15,0.5,1.5,5,15.8,50,158,500 nM alpha-factor(8)
(c) 49. Expression in response to 50 nM alpha-factor: 0,15,30,45,60,90,120 min(1)
(c) 49. Expression in response to 50 nM alpha-factor: 0,15,30,45,60,90,120 min(2)
(c) 49. Expression in response to 50 nM alpha-factor: 0,15,30,45,60,90,120 min(3)
(c) 49. Expression in response to 50 nM alpha-factor: 0,15,30,45,60,90,120 min(4)
(c) 49. Expression in response to 50 nM alpha-factor: 0,15,30,45,60,90,120 min(5)
(c) 49. Expression in response to 50 nM alpha-factor: 0,15,30,45,60,90,120 min(6)
(c) 49. Expression in response to 50 nM alpha-factor: 0,15,30,45,60,90,120 min(7)
(c) 53. Expression in response to overproduction of Ste4p(1)

(c) 54. Expression in response to overproduction of Ste5p(1)
(c) 55. Expression in response to overproduction of Ste11p(1)
(c) 56. Expression in response to overproduction of Ste12p(1)
(c) (Rich Media 2% Glucose YPD-185588) wt 5mM aF, 30 min.
(c) (Rich Media 2% Glucose YPD-185769) wt 5mM aF, 30 min.
(c) Rich Media 2% Glucose YPD-Average wt 5mM aF, 30 min.

STE12 -*-> MFA2

(c) 5. Expression during the cell cycle (alpha factor arrest and release)(1)
(c) 48. Expression in response to 0.15,0.5,1.5,5,15.8,50,158,500 nM alpha-factor(3)
(c) 48. Expression in response to 0.15,0.5,1.5,5,15.8,50,158,500 nM alpha-factor(4)
(c) 48. Expression in response to 0.15,0.5,1.5,5,15.8,50,158,500 nM alpha-factor(5)
(c) 48. Expression in response to 0.15,0.5,1.5,5,15.8,50,158,500 nM alpha-factor(6)
(c) 48. Expression in response to 0.15,0.5,1.5,5,15.8,50,158,500 nM alpha-factor(7)
(c) 48. Expression in response to 0.15,0.5,1.5,5,15.8,50,158,500 nM alpha-factor(8)
(c) 49. Expression in response to 50 nM alpha-factor: 0,15,30,45,60,90,120 min(1)
(c) 49. Expression in response to 50 nM alpha-factor: 0,15,30,45,60,90,120 min(2)
(c) 49. Expression in response to 50 nM alpha-factor: 0,15,30,45,60,90,120 min(3)
(c) 49. Expression in response to 50 nM alpha-factor: 0,15,30,45,60,90,120 min(4)
(c) 49. Expression in response to 50 nM alpha-factor: 0,15,30,45,60,90,120 min(5)
(c) 49. Expression in response to 50 nM alpha-factor: 0,15,30,45,60,90,120 min(6)
(c) 49. Expression in response to 50 nM alpha-factor: 0,15,30,45,60,90,120 min(7)
(c) 53. Expression in response to overproduction of Ste4p(1)
(c) 54. Expression in response to overproduction of Ste5p(1)
(c) 55. Expression in response to overproduction of Ste11p(1)
(c) 56. Expression in response to overproduction of Ste12p(1)
(c) (Rich Media 2% Glucose YPD-185588) wt 5mM aF, 30 min.
(c) (Rich Media 2% Glucose YPD-185769) wt 5mM aF, 30 min.
(c) Rich Media 2% Glucose YPD-Average wt 5mM aF, 30 min.

STE12 -*-> PRM4

(c) 5. Expression during the cell cycle (alpha factor arrest and release)(1)
(c) 48. Expression in response to 0.15,0.5,1.5,5,15.8,50,158,500 nM alpha-factor(3)
(c) 48. Expression in response to 0.15,0.5,1.5,5,15.8,50,158,500 nM alpha-factor(4)
(c) 48. Expression in response to 0.15,0.5,1.5,5,15.8,50,158,500 nM alpha-factor(5)
(c) 48. Expression in response to 0.15,0.5,1.5,5,15.8,50,158,500 nM alpha-factor(6)
(c) 48. Expression in response to 0.15,0.5,1.5,5,15.8,50,158,500 nM alpha-factor(7)
(c) 48. Expression in response to 0.15,0.5,1.5,5,15.8,50,158,500 nM alpha-factor(8)
(c) 49. Expression in response to 50 nM alpha-factor: 0,15,30,45,60,90,120 min(1)
(c) 49. Expression in response to 50 nM alpha-factor: 0,15,30,45,60,90,120 min(2)
(c) 49. Expression in response to 50 nM alpha-factor: 0,15,30,45,60,90,120 min(3)
(c) 49. Expression in response to 50 nM alpha-factor: 0,15,30,45,60,90,120 min(4)
(c) 49. Expression in response to 50 nM alpha-factor: 0,15,30,45,60,90,120 min(5)
(c) 49. Expression in response to 50 nM alpha-factor: 0,15,30,45,60,90,120 min(6)
(c) 49. Expression in response to 50 nM alpha-factor: 0,15,30,45,60,90,120 min(7)
(c) 53. Expression in response to overproduction of Ste4p(1)
(c) 54. Expression in response to overproduction of Ste5p(1)
(c) 55. Expression in response to overproduction of Ste11p(1)
(c) 56. Expression in response to overproduction of Ste12p(1)
(c) (Rich Media 2% Glucose YPD-185588) wt 5mM aF, 30 min.
(c) (Rich Media 2% Glucose YPD-185769) wt 5mM aF, 30 min.
(c) Rich Media 2% Glucose YPD-Average wt 5mM aF, 30 min.

STE12 -*-> PRM6

(c) 5. Expression during the cell cycle (alpha factor arrest and release)(1)
(c) 48. Expression in response to 0.15,0.5,1.5,5,15.8,50,158,500 nM alpha-factor(3)
(c) 48. Expression in response to 0.15,0.5,1.5,5,15.8,50,158,500 nM alpha-factor(4)
(c) 48. Expression in response to 0.15,0.5,1.5,5,15.8,50,158,500 nM alpha-factor(5)
(c) 48. Expression in response to 0.15,0.5,1.5,5,15.8,50,158,500 nM alpha-factor(6)
(c) 48. Expression in response to 0.15,0.5,1.5,5,15.8,50,158,500 nM alpha-factor(7)
(c) 48. Expression in response to 0.15,0.5,1.5,5,15.8,50,158,500 nM alpha-factor(8)
(c) 49. Expression in response to 50 nM alpha-factor: 0,15,30,45,60,90,120 min(1)
(c) 49. Expression in response to 50 nM alpha-factor: 0,15,30,45,60,90,120 min(2)
(c) 49. Expression in response to 50 nM alpha-factor: 0,15,30,45,60,90,120 min(3)
(c) 49. Expression in response to 50 nM alpha-factor: 0,15,30,45,60,90,120 min(4)
(c) 49. Expression in response to 50 nM alpha-factor: 0,15,30,45,60,90,120 min(5)
(c) 49. Expression in response to 50 nM alpha-factor: 0,15,30,45,60,90,120 min(6)
(c) 49. Expression in response to 50 nM alpha-factor: 0,15,30,45,60,90,120 min(7)
(c) 53. Expression in response to overproduction of Ste4p(1)
(c) 54. Expression in response to overproduction of Ste5p(1)
(c) 55. Expression in response to overproduction of Ste11p(1)
(c) 56. Expression in response to overproduction of Ste12p(1)
(c) (Rich Media 2% Glucose YPD-185588) wt 5mM aF, 30 min.
(c) (Rich Media 2% Glucose YPD-185769) wt 5mM aF, 30 min.
(c) Rich Media 2% Glucose YPD-Average wt 5mM aF, 30 min.

STE12 -*-> PRP39

(c) 5. Expression during the cell cycle (alpha factor arrest and release)(1)
(c) 48. Expression in response to 0.15,0.5,1.5,5,15.8,50,158,500 nM alpha-factor(3)
(c) 48. Expression in response to 0.15,0.5,1.5,5,15.8,50,158,500 nM alpha-factor(4)
(c) 48. Expression in response to 0.15,0.5,1.5,5,15.8,50,158,500 nM alpha-factor(5)
(c) 48. Expression in response to 0.15,0.5,1.5,5,15.8,50,158,500 nM alpha-factor(6)
(c) 48. Expression in response to 0.15,0.5,1.5,5,15.8,50,158,500 nM alpha-factor(7)
(c) 48. Expression in response to 0.15,0.5,1.5,5,15.8,50,158,500 nM alpha-factor(8)
(c) 49. Expression in response to 50 nM alpha-factor: 0,15,30,45,60,90,120 min(1)
(c) 49. Expression in response to 50 nM alpha-factor: 0,15,30,45,60,90,120 min(2)
(c) 49. Expression in response to 50 nM alpha-factor: 0,15,30,45,60,90,120 min(3)
(c) 49. Expression in response to 50 nM alpha-factor: 0,15,30,45,60,90,120 min(4)
(c) 49. Expression in response to 50 nM alpha-factor: 0,15,30,45,60,90,120 min(5)
(c) 49. Expression in response to 50 nM alpha-factor: 0,15,30,45,60,90,120 min(6)
(c) 49. Expression in response to 50 nM alpha-factor: 0,15,30,45,60,90,120 min(7)
(c) 53. Expression in response to overproduction of Ste4p(1)
(c) 54. Expression in response to overproduction of Ste5p(1)
(c) 55. Expression in response to overproduction of Ste11p(1)
(c) 56. Expression in response to overproduction of Ste12p(1)
(c) (Rich Media 2% Glucose YPD-185588) wt 5mM aF, 30 min.
(c) (Rich Media 2% Glucose YPD-185769) wt 5mM aF, 30 min.
(c) Rich Media 2% Glucose YPD-Average wt 5mM aF, 30 min.

STE12 -*-> RDI1

(c) 5. Expression during the cell cycle (alpha factor arrest and release)(1)
(c) 48. Expression in response to 0.15,0.5,1.5,5,15.8,50,158,500 nM alpha-factor(3)
(c) 48. Expression in response to 0.15,0.5,1.5,5,15.8,50,158,500 nM alpha-factor(4)
(c) 48. Expression in response to 0.15,0.5,1.5,5,15.8,50,158,500 nM alpha-factor(5)
(c) 48. Expression in response to 0.15,0.5,1.5,5,15.8,50,158,500 nM alpha-factor(6)
(c) 48. Expression in response to 0.15,0.5,1.5,5,15.8,50,158,500 nM alpha-factor(7)
(c) 48. Expression in response to 0.15,0.5,1.5,5,15.8,50,158,500 nM alpha-factor(8)
(c) 49. Expression in response to 50 nM alpha-factor: 0,15,30,45,60,90,120 min(1)
(c) 49. Expression in response to 50 nM alpha-factor: 0,15,30,45,60,90,120 min(2)
(c) 49. Expression in response to 50 nM alpha-factor: 0,15,30,45,60,90,120 min(3)
(c) 49. Expression in response to 50 nM alpha-factor: 0,15,30,45,60,90,120 min(4)
(c) 49. Expression in response to 50 nM alpha-factor: 0,15,30,45,60,90,120 min(5)
(c) 49. Expression in response to 50 nM alpha-factor: 0,15,30,45,60,90,120 min(6)
(c) 49. Expression in response to 50 nM alpha-factor: 0,15,30,45,60,90,120 min(7)
(c) 53. Expression in response to overproduction of Ste4p(1)
(c) 54. Expression in response to overproduction of Ste5p(1)
(c) 55. Expression in response to overproduction of Ste11p(1)
(c) 56. Expression in response to overproduction of Ste12p(1)
(c) (Rich Media 2% Glucose YPD-185588) wt 5mM aF, 30 min.
(c) (Rich Media 2% Glucose YPD-185769) wt 5mM aF, 30 min.
(c) Rich Media 2% Glucose YPD-Average wt 5mM aF, 30 min.

STE12 -*-> TEC1

(c) 5. Expression during the cell cycle (alpha factor arrest and release)(1)
(c) 48. Expression in response to 0.15,0.5,1.5,5,15.8,50,158,500 nM alpha-factor(3)
(c) 48. Expression in response to 0.15,0.5,1.5,5,15.8,50,158,500 nM alpha-factor(4)
(c) 48. Expression in response to 0.15,0.5,1.5,5,15.8,50,158,500 nM alpha-factor(5)
(c) 48. Expression in response to 0.15,0.5,1.5,5,15.8,50,158,500 nM alpha-factor(6)
(c) 48. Expression in response to 0.15,0.5,1.5,5,15.8,50,158,500 nM alpha-factor(7)
(c) 48. Expression in response to 0.15,0.5,1.5,5,15.8,50,158,500 nM alpha-factor(8)
(c) 49. Expression in response to 50 nM alpha-factor: 0,15,30,45,60,90,120 min(1)
(c) 49. Expression in response to 50 nM alpha-factor: 0,15,30,45,60,90,120 min(2)
(c) 49. Expression in response to 50 nM alpha-factor: 0,15,30,45,60,90,120 min(3)
(c) 49. Expression in response to 50 nM alpha-factor: 0,15,30,45,60,90,120 min(4)
(c) 49. Expression in response to 50 nM alpha-factor: 0,15,30,45,60,90,120 min(5)
(c) 49. Expression in response to 50 nM alpha-factor: 0,15,30,45,60,90,120 min(6)
(c) 49. Expression in response to 50 nM alpha-factor: 0,15,30,45,60,90,120 min(7)
(c) 53. Expression in response to overproduction of Ste4p(1)
(c) 54. Expression in response to overproduction of Ste5p(1)
(c) 55. Expression in response to overproduction of Ste11p(1)
(c) 56. Expression in response to overproduction of Ste12p(1)
(c) (Rich Media 2% Glucose YPD-185588) wt 5mM aF, 30 min.
(c) (Rich Media 2% Glucose YPD-185769) wt 5mM aF, 30 min.
(c) Rich Media 2% Glucose YPD-Average wt 5mM aF, 30 min.

STE12 -*-> YJL107C

(c) 5. Expression during the cell cycle (alpha factor arrest and release)(1)
(c) 48. Expression in response to 0.15,0.5,1.5,5,15.8,50,158,500 nM alpha-factor(3)
(c) 48. Expression in response to 0.15,0.5,1.5,5,15.8,50,158,500 nM alpha-factor(4)
(c) 48. Expression in response to 0.15,0.5,1.5,5,15.8,50,158,500 nM alpha-factor(5)
(c) 48. Expression in response to 0.15,0.5,1.5,5,15.8,50,158,500 nM alpha-factor(6)
(c) 48. Expression in response to 0.15,0.5,1.5,5,15.8,50,158,500 nM alpha-factor(7)
(c) 48. Expression in response to 0.15,0.5,1.5,5,15.8,50,158,500 nM alpha-factor(8)
(c) 49. Expression in response to 50 nM alpha-factor: 0,15,30,45,60,90,120 min(1)
(c) 49. Expression in response to 50 nM alpha-factor: 0,15,30,45,60,90,120 min(2)
(c) 49. Expression in response to 50 nM alpha-factor: 0,15,30,45,60,90,120 min(3)
(c) 49. Expression in response to 50 nM alpha-factor: 0,15,30,45,60,90,120 min(4)
(c) 49. Expression in response to 50 nM alpha-factor: 0,15,30,45,60,90,120 min(5)
(c) 49. Expression in response to 50 nM alpha-factor: 0,15,30,45,60,90,120 min(6)
(c) 49. Expression in response to 50 nM alpha-factor: 0,15,30,45,60,90,120 min(7)
(c) 53. Expression in response to overproduction of Ste4p(1)
(c) 54. Expression in response to overproduction of Ste5p(1)
(c) 55. Expression in response to overproduction of Ste11p(1)
(c) 56. Expression in response to overproduction of Ste12p(1)
(c) (Rich Media 2% Glucose YPD-185588) wt 5mM aF, 30 min.
(c) (Rich Media 2% Glucose YPD-185769) wt 5mM aF, 30 min.
(c) Rich Media 2% Glucose YPD-Average wt 5mM aF, 30 min.

SWI5 -*-| BUD9

(c) 5. Expression during the cell cycle (alpha factor arrest and release)(10)
(c) 5. Expression during the cell cycle (alpha factor arrest and release)(11)
(c) 5. Expression during the cell cycle (alpha factor arrest and release)(12)
(c) 5. Expression during the cell cycle (alpha factor arrest and release)(13)
(c) 6. Expression during the cell cycle (cdc15 arrest and release)(1)
(c) 6. Expression during the cell cycle (cdc15 arrest and release)(2)
(c) 6. Expression during the cell cycle (cdc15 arrest and release)(10)
(c) 6. Expression during the cell cycle (cdc15 arrest and release)(11)
(c) 6. Expression during the cell cycle (cdc15 arrest and release)(12)
(c) 6. Expression during the cell cycle (cdc15 arrest and release)(22)
(c) 6. Expression during the cell cycle (cdc15 arrest and release)(23)
(c) 7. Expression during the cell Cycle (cdc28)(10)
(c) 7. Expression during the cell Cycle (cdc28)(11)
(c) 7. Expression during the cell Cycle (cdc28)(12)
(c) 7. Expression during the cell Cycle (cdc28)(17)
(c) 8. Expression during the cell cycle (cell size selection and release)(12)
(c) 8. Expression during the cell cycle (cell size selection and release)(14)

SWI5 -*-| CDC46

(c) 5. Expression during the cell cycle (alpha factor arrest and release)(10)
(c) 5. Expression during the cell cycle (alpha factor arrest and release)(11)
(c) 5. Expression during the cell cycle (alpha factor arrest and release)(12)
(c) 5. Expression during the cell cycle (alpha factor arrest and release)(13)
(c) 6. Expression during the cell cycle (cdc15 arrest and release)(1)
(c) 6. Expression during the cell cycle (cdc15 arrest and release)(2)
(c) 6. Expression during the cell cycle (cdc15 arrest and release)(10)
(c) 6. Expression during the cell cycle (cdc15 arrest and release)(11)
(c) 6. Expression during the cell cycle (cdc15 arrest and release)(12)

(c) 6. Expression during the cell cycle (cdc15 arrest and release)(22)
(c) 6. Expression during the cell cycle (cdc15 arrest and release)(23)
(c) 7. Expression during the cell Cycle (cdc28)(10)
(c) 7. Expression during the cell Cycle (cdc28)(11)
(c) 7. Expression during the cell Cycle (cdc28)(12)
(c) 7. Expression during the cell Cycle (cdc28)(17)
(c) 8. Expression during the cell cycle (cell size selection and release)(12)
(c) 8. Expression during the cell cycle (cell size selection and release)(14)

SWI5 -*-| CST13

(c) 5. Expression during the cell cycle (alpha factor arrest and release)(10)
(c) 5. Expression during the cell cycle (alpha factor arrest and release)(11)
(c) 5. Expression during the cell cycle (alpha factor arrest and release)(12)
(c) 5. Expression during the cell cycle (alpha factor arrest and release)(13)
(c) 6. Expression during the cell cycle (cdc15 arrest and release)(1)
(c) 6. Expression during the cell cycle (cdc15 arrest and release)(2)
(c) 6. Expression during the cell cycle (cdc15 arrest and release)(10)
(c) 6. Expression during the cell cycle (cdc15 arrest and release)(11)
(c) 6. Expression during the cell cycle (cdc15 arrest and release)(12)
(c) 6. Expression during the cell cycle (cdc15 arrest and release)(22)
(c) 6. Expression during the cell cycle (cdc15 arrest and release)(23)
(c) 7. Expression during the cell Cycle (cdc28)(10)
(c) 7. Expression during the cell Cycle (cdc28)(11)
(c) 7. Expression during the cell Cycle (cdc28)(12)
(c) 7. Expression during the cell Cycle (cdc28)(17)
(c) 8. Expression during the cell cycle (cell size selection and release)(12)
(c) 8. Expression during the cell cycle (cell size selection and release)(14)

SWI5 -*-| CYK3

(c) 5. Expression during the cell cycle (alpha factor arrest and release)(10)
(c) 5. Expression during the cell cycle (alpha factor arrest and release)(11)
(c) 5. Expression during the cell cycle (alpha factor arrest and release)(12)
(c) 5. Expression during the cell cycle (alpha factor arrest and release)(13)
(c) 6. Expression during the cell cycle (cdc15 arrest and release)(1)
(c) 6. Expression during the cell cycle (cdc15 arrest and release)(2)
(c) 6. Expression during the cell cycle (cdc15 arrest and release)(10)
(c) 6. Expression during the cell cycle (cdc15 arrest and release)(11)
(c) 6. Expression during the cell cycle (cdc15 arrest and release)(12)
(c) 6. Expression during the cell cycle (cdc15 arrest and release)(22)
(c) 6. Expression during the cell cycle (cdc15 arrest and release)(23)
(c) 7. Expression during the cell Cycle (cdc28)(10)
(c) 7. Expression during the cell Cycle (cdc28)(11)
(c) 7. Expression during the cell Cycle (cdc28)(12)
(c) 7. Expression during the cell Cycle (cdc28)(17)
(c) 8. Expression during the cell cycle (cell size selection and release)(12)
(c) 8. Expression during the cell cycle (cell size selection and release)(14)

SWI5 -*-| FAA3

(c) 5. Expression during the cell cycle (alpha factor arrest and release)(10)
(c) 5. Expression during the cell cycle (alpha factor arrest and release)(11)
(c) 5. Expression during the cell cycle (alpha factor arrest and release)(12)
(c) 5. Expression during the cell cycle (alpha factor arrest and release)(13)
(c) 6. Expression during the cell cycle (cdc15 arrest and release)(1)
(c) 6. Expression during the cell cycle (cdc15 arrest and release)(2)
(c) 6. Expression during the cell cycle (cdc15 arrest and release)(10)
(c) 6. Expression during the cell cycle (cdc15 arrest and release)(11)
(c) 6. Expression during the cell cycle (cdc15 arrest and release)(12)
(c) 6. Expression during the cell cycle (cdc15 arrest and release)(22)
(c) 6. Expression during the cell cycle (cdc15 arrest and release)(23)
(c) 7. Expression during the cell Cycle (cdc28)(10)
(c) 7. Expression during the cell Cycle (cdc28)(11)
(c) 7. Expression during the cell Cycle (cdc28)(12)
(c) 7. Expression during the cell Cycle (cdc28)(17)
(c) 8. Expression during the cell cycle (cell size selection and release)(12)
(c) 8. Expression during the cell cycle (cell size selection and release)(14)

SWI5 -*-| HSP150

(c) 5. Expression during the cell cycle (alpha factor arrest and release)(10)
(c) 5. Expression during the cell cycle (alpha factor arrest and release)(11)
(c) 5. Expression during the cell cycle (alpha factor arrest and release)(12)
(c) 5. Expression during the cell cycle (alpha factor arrest and release)(13)
(c) 6. Expression during the cell cycle (cdc15 arrest and release)(1)
(c) 6. Expression during the cell cycle (cdc15 arrest and release)(2)
(c) 6. Expression during the cell cycle (cdc15 arrest and release)(10)
(c) 6. Expression during the cell cycle (cdc15 arrest and release)(11)
(c) 6. Expression during the cell cycle (cdc15 arrest and release)(12)
(c) 6. Expression during the cell cycle (cdc15 arrest and release)(22)
(c) 6. Expression during the cell cycle (cdc15 arrest and release)(23)
(c) 7. Expression during the cell Cycle (cdc28)(10)
(c) 7. Expression during the cell Cycle (cdc28)(11)
(c) 7. Expression during the cell Cycle (cdc28)(12)
(c) 7. Expression during the cell Cycle (cdc28)(17)
(c) 8. Expression during the cell cycle (cell size selection and release)(12)
(c) 8. Expression during the cell cycle (cell size selection and release)(14)

SWI5 -*-| JIP1

(c) 5. Expression during the cell cycle (alpha factor arrest and release)(10)
(c) 5. Expression during the cell cycle (alpha factor arrest and release)(11)
(c) 5. Expression during the cell cycle (alpha factor arrest and release)(12)
(c) 5. Expression during the cell cycle (alpha factor arrest and release)(13)
(c) 6. Expression during the cell cycle (cdc15 arrest and release)(1)
(c) 6. Expression during the cell cycle (cdc15 arrest and release)(2)
(c) 6. Expression during the cell cycle (cdc15 arrest and release)(10)
(c) 6. Expression during the cell cycle (cdc15 arrest and release)(11)
(c) 6. Expression during the cell cycle (cdc15 arrest and release)(12)
(c) 6. Expression during the cell cycle (cdc15 arrest and release)(22)
(c) 6. Expression during the cell cycle (cdc15 arrest and release)(23)

(c) 7. Expression during the cell Cycle (cdc28)(10)
(c) 7. Expression during the cell Cycle (cdc28)(11)
(c) 7. Expression during the cell Cycle (cdc28)(12)
(c) 7. Expression during the cell Cycle (cdc28)(17)
(c) 8. Expression during the cell cycle (cell size selection and release)(12)
(c) 8. Expression during the cell cycle (cell size selection and release)(14)

SWI5 -*-| KCC4

(c) 5. Expression during the cell cycle (alpha factor arrest and release)(10)
(c) 5. Expression during the cell cycle (alpha factor arrest and release)(11)
(c) 5. Expression during the cell cycle (alpha factor arrest and release)(12)
(c) 5. Expression during the cell cycle (alpha factor arrest and release)(13)
(c) 6. Expression during the cell cycle (cdc15 arrest and release)(1)
(c) 6. Expression during the cell cycle (cdc15 arrest and release)(2)
(c) 6. Expression during the cell cycle (cdc15 arrest and release)(10)
(c) 6. Expression during the cell cycle (cdc15 arrest and release)(11)
(c) 6. Expression during the cell cycle (cdc15 arrest and release)(12)
(c) 6. Expression during the cell cycle (cdc15 arrest and release)(22)
(c) 6. Expression during the cell cycle (cdc15 arrest and release)(23)
(c) 7. Expression during the cell Cycle (cdc28)(10)
(c) 7. Expression during the cell Cycle (cdc28)(11)
(c) 7. Expression during the cell Cycle (cdc28)(12)
(c) 7. Expression during the cell Cycle (cdc28)(17)
(c) 8. Expression during the cell cycle (cell size selection and release)(12)
(c) 8. Expression during the cell cycle (cell size selection and release)(14)

SWI5 -*-| PIR1

(c) 5. Expression during the cell cycle (alpha factor arrest and release)(10)
(c) 5. Expression during the cell cycle (alpha factor arrest and release)(11)
(c) 5. Expression during the cell cycle (alpha factor arrest and release)(12)
(c) 5. Expression during the cell cycle (alpha factor arrest and release)(13)
(c) 6. Expression during the cell cycle (cdc15 arrest and release)(1)
(c) 6. Expression during the cell cycle (cdc15 arrest and release)(2)
(c) 6. Expression during the cell cycle (cdc15 arrest and release)(10)
(c) 6. Expression during the cell cycle (cdc15 arrest and release)(11)
(c) 6. Expression during the cell cycle (cdc15 arrest and release)(12)
(c) 6. Expression during the cell cycle (cdc15 arrest and release)(22)
(c) 6. Expression during the cell cycle (cdc15 arrest and release)(23)
(c) 7. Expression during the cell Cycle (cdc28)(10)
(c) 7. Expression during the cell Cycle (cdc28)(11)
(c) 7. Expression during the cell Cycle (cdc28)(12)
(c) 7. Expression during the cell Cycle (cdc28)(17)
(c) 8. Expression during the cell cycle (cell size selection and release)(12)
(c) 8. Expression during the cell cycle (cell size selection and release)(14)

SWI5 -*-| PRY3

(c) 5. Expression during the cell cycle (alpha factor arrest and release)(10)
(c) 5. Expression during the cell cycle (alpha factor arrest and release)(11)
(c) 5. Expression during the cell cycle (alpha factor arrest and release)(12)
(c) 5. Expression during the cell cycle (alpha factor arrest and release)(13)
(c) 6. Expression during the cell cycle (cdc15 arrest and release)(1)
(c) 6. Expression during the cell cycle (cdc15 arrest and release)(2)
(c) 6. Expression during the cell cycle (cdc15 arrest and release)(10)
(c) 6. Expression during the cell cycle (cdc15 arrest and release)(11)
(c) 6. Expression during the cell cycle (cdc15 arrest and release)(12)
(c) 6. Expression during the cell cycle (cdc15 arrest and release)(22)
(c) 6. Expression during the cell cycle (cdc15 arrest and release)(23)
(c) 7. Expression during the cell Cycle (cdc28)(10)
(c) 7. Expression during the cell Cycle (cdc28)(11)
(c) 7. Expression during the cell Cycle (cdc28)(12)
(c) 7. Expression during the cell Cycle (cdc28)(17)
(c) 8. Expression during the cell cycle (cell size selection and release)(12)
(c) 8. Expression during the cell cycle (cell size selection and release)(14)

SWI5 -*-| PST1

(c) 5. Expression during the cell cycle (alpha factor arrest and release)(10)
(c) 5. Expression during the cell cycle (alpha factor arrest and release)(11)
(c) 5. Expression during the cell cycle (alpha factor arrest and release)(12)
(c) 5. Expression during the cell cycle (alpha factor arrest and release)(13)
(c) 6. Expression during the cell cycle (cdc15 arrest and release)(1)
(c) 6. Expression during the cell cycle (cdc15 arrest and release)(2)
(c) 6. Expression during the cell cycle (cdc15 arrest and release)(10)
(c) 6. Expression during the cell cycle (cdc15 arrest and release)(11)
(c) 6. Expression during the cell cycle (cdc15 arrest and release)(12)
(c) 6. Expression during the cell cycle (cdc15 arrest and release)(22)
(c) 6. Expression during the cell cycle (cdc15 arrest and release)(23)
(c) 7. Expression during the cell Cycle (cdc28)(10)
(c) 7. Expression during the cell Cycle (cdc28)(11)
(c) 7. Expression during the cell Cycle (cdc28)(12)
(c) 7. Expression during the cell Cycle (cdc28)(17)
(c) 8. Expression during the cell cycle (cell size selection and release)(12)
(c) 8. Expression during the cell cycle (cell size selection and release)(14)

SWI5 -*-| SCW11

(c) 5. Expression during the cell cycle (alpha factor arrest and release)(10)
(c) 5. Expression during the cell cycle (alpha factor arrest and release)(11)
(c) 5. Expression during the cell cycle (alpha factor arrest and release)(12)
(c) 5. Expression during the cell cycle (alpha factor arrest and release)(13)
(c) 6. Expression during the cell cycle (cdc15 arrest and release)(1)
(c) 6. Expression during the cell cycle (cdc15 arrest and release)(2)
(c) 6. Expression during the cell cycle (cdc15 arrest and release)(10)
(c) 6. Expression during the cell cycle (cdc15 arrest and release)(11)
(c) 6. Expression during the cell cycle (cdc15 arrest and release)(12)
(c) 6. Expression during the cell cycle (cdc15 arrest and release)(22)
(c) 6. Expression during the cell cycle (cdc15 arrest and release)(23)
(c) 7. Expression during the cell Cycle (cdc28)(10)
(c) 7. Expression during the cell Cycle (cdc28)(11)

(c) 7. Expression during the cell Cycle (cdc28)(12)
(c) 7. Expression during the cell Cycle (cdc28)(17)
(c) 8. Expression during the cell cycle (cell size selection and release)(12)
(c) 8. Expression during the cell cycle (cell size selection and release)(14)

SWI5 -*-| SIT4

(c) 5. Expression during the cell cycle (alpha factor arrest and release)(10)
(c) 5. Expression during the cell cycle (alpha factor arrest and release)(11)
(c) 5. Expression during the cell cycle (alpha factor arrest and release)(12)
(c) 5. Expression during the cell cycle (alpha factor arrest and release)(13)
(c) 6. Expression during the cell cycle (cdc15 arrest and release)(1)
(c) 6. Expression during the cell cycle (cdc15 arrest and release)(2)
(c) 6. Expression during the cell cycle (cdc15 arrest and release)(10)
(c) 6. Expression during the cell cycle (cdc15 arrest and release)(11)
(c) 6. Expression during the cell cycle (cdc15 arrest and release)(12)
(c) 6. Expression during the cell cycle (cdc15 arrest and release)(22)
(c) 6. Expression during the cell cycle (cdc15 arrest and release)(23)
(c) 7. Expression during the cell Cycle (cdc28)(10)
(c) 7. Expression during the cell Cycle (cdc28)(11)
(c) 7. Expression during the cell Cycle (cdc28)(12)
(c) 7. Expression during the cell Cycle (cdc28)(17)
(c) 8. Expression during the cell cycle (cell size selection and release)(12)
(c) 8. Expression during the cell cycle (cell size selection and release)(14)

SWI5 -*-| TEC1

(c) 5. Expression during the cell cycle (alpha factor arrest and release)(10)
(c) 5. Expression during the cell cycle (alpha factor arrest and release)(11)
(c) 5. Expression during the cell cycle (alpha factor arrest and release)(12)
(c) 5. Expression during the cell cycle (alpha factor arrest and release)(13)
(c) 6. Expression during the cell cycle (cdc15 arrest and release)(1)
(c) 6. Expression during the cell cycle (cdc15 arrest and release)(2)
(c) 6. Expression during the cell cycle (cdc15 arrest and release)(10)
(c) 6. Expression during the cell cycle (cdc15 arrest and release)(11)
(c) 6. Expression during the cell cycle (cdc15 arrest and release)(12)
(c) 6. Expression during the cell cycle (cdc15 arrest and release)(22)
(c) 6. Expression during the cell cycle (cdc15 arrest and release)(23)
(c) 7. Expression during the cell Cycle (cdc28)(10)
(c) 7. Expression during the cell Cycle (cdc28)(11)
(c) 7. Expression during the cell Cycle (cdc28)(12)
(c) 7. Expression during the cell Cycle (cdc28)(17)
(c) 8. Expression during the cell cycle (cell size selection and release)(12)
(c) 8. Expression during the cell cycle (cell size selection and release)(14)

SWI5 -*-| TOS7

(c) 5. Expression during the cell cycle (alpha factor arrest and release)(10)
(c) 5. Expression during the cell cycle (alpha factor arrest and release)(11)
(c) 5. Expression during the cell cycle (alpha factor arrest and release)(12)
(c) 5. Expression during the cell cycle (alpha factor arrest and release)(13)
(c) 6. Expression during the cell cycle (cdc15 arrest and release)(1)
(c) 6. Expression during the cell cycle (cdc15 arrest and release)(2)
(c) 6. Expression during the cell cycle (cdc15 arrest and release)(10)
(c) 6. Expression during the cell cycle (cdc15 arrest and release)(11)
(c) 6. Expression during the cell cycle (cdc15 arrest and release)(12)
(c) 6. Expression during the cell cycle (cdc15 arrest and release)(22)
(c) 6. Expression during the cell cycle (cdc15 arrest and release)(23)
(c) 7. Expression during the cell Cycle (cdc28)(10)
(c) 7. Expression during the cell Cycle (cdc28)(11)
(c) 7. Expression during the cell Cycle (cdc28)(12)
(c) 7. Expression during the cell Cycle (cdc28)(17)
(c) 8. Expression during the cell cycle (cell size selection and release)(12)
(c) 8. Expression during the cell cycle (cell size selection and release)(14)

SWI5 -*-| YBR071W

(c) 5. Expression during the cell cycle (alpha factor arrest and release)(10)
(c) 5. Expression during the cell cycle (alpha factor arrest and release)(11)
(c) 5. Expression during the cell cycle (alpha factor arrest and release)(12)
(c) 5. Expression during the cell cycle (alpha factor arrest and release)(13)
(c) 6. Expression during the cell cycle (cdc15 arrest and release)(1)
(c) 6. Expression during the cell cycle (cdc15 arrest and release)(2)
(c) 6. Expression during the cell cycle (cdc15 arrest and release)(10)
(c) 6. Expression during the cell cycle (cdc15 arrest and release)(11)
(c) 6. Expression during the cell cycle (cdc15 arrest and release)(12)
(c) 6. Expression during the cell cycle (cdc15 arrest and release)(22)
(c) 6. Expression during the cell cycle (cdc15 arrest and release)(23)
(c) 7. Expression during the cell Cycle (cdc28)(10)
(c) 7. Expression during the cell Cycle (cdc28)(11)
(c) 7. Expression during the cell Cycle (cdc28)(12)
(c) 7. Expression during the cell Cycle (cdc28)(17)
(c) 8. Expression during the cell cycle (cell size selection and release)(12)
(c) 8. Expression during the cell cycle (cell size selection and release)(14)

SWI5 -*-| YGR086C

(c) 5. Expression during the cell cycle (alpha factor arrest and release)(10)
(c) 5. Expression during the cell cycle (alpha factor arrest and release)(11)
(c) 5. Expression during the cell cycle (alpha factor arrest and release)(12)
(c) 5. Expression during the cell cycle (alpha factor arrest and release)(13)
(c) 6. Expression during the cell cycle (cdc15 arrest and release)(1)
(c) 6. Expression during the cell cycle (cdc15 arrest and release)(2)
(c) 6. Expression during the cell cycle (cdc15 arrest and release)(10)
(c) 6. Expression during the cell cycle (cdc15 arrest and release)(11)
(c) 6. Expression during the cell cycle (cdc15 arrest and release)(12)
(c) 6. Expression during the cell cycle (cdc15 arrest and release)(22)
(c) 6. Expression during the cell cycle (cdc15 arrest and release)(23)
(c) 7. Expression during the cell Cycle (cdc28)(10)
(c) 7. Expression during the cell Cycle (cdc28)(11)
(c) 7. Expression during the cell Cycle (cdc28)(12)
(c) 7. Expression during the cell Cycle (cdc28)(17)

(c) 8. Expression during the cell cycle (cell size selection and release)(12)
(c) 8. Expression during the cell cycle (cell size selection and release)(14)

SWI5 -*-| YHR218W

(c) 5. Expression during the cell cycle (alpha factor arrest and release)(10)
(c) 5. Expression during the cell cycle (alpha factor arrest and release)(11)
(c) 5. Expression during the cell cycle (alpha factor arrest and release)(12)
(c) 5. Expression during the cell cycle (alpha factor arrest and release)(13)
(c) 6. Expression during the cell cycle (cdc15 arrest and release)(1)
(c) 6. Expression during the cell cycle (cdc15 arrest and release)(2)
(c) 6. Expression during the cell cycle (cdc15 arrest and release)(10)
(c) 6. Expression during the cell cycle (cdc15 arrest and release)(11)
(c) 6. Expression during the cell cycle (cdc15 arrest and release)(12)
(c) 6. Expression during the cell cycle (cdc15 arrest and release)(22)
(c) 6. Expression during the cell cycle (cdc15 arrest and release)(23)
(c) 7. Expression during the cell Cycle (cdc28)(10)
(c) 7. Expression during the cell Cycle (cdc28)(11)
(c) 7. Expression during the cell Cycle (cdc28)(12)
(c) 7. Expression during the cell Cycle (cdc28)(17)
(c) 8. Expression during the cell cycle (cell size selection and release)(12)
(c) 8. Expression during the cell cycle (cell size selection and release)(14)

SWI5 -*-| YIL177C

(c) 5. Expression during the cell cycle (alpha factor arrest and release)(10)
(c) 5. Expression during the cell cycle (alpha factor arrest and release)(11)
(c) 5. Expression during the cell cycle (alpha factor arrest and release)(12)
(c) 5. Expression during the cell cycle (alpha factor arrest and release)(13)
(c) 6. Expression during the cell cycle (cdc15 arrest and release)(1)
(c) 6. Expression during the cell cycle (cdc15 arrest and release)(2)
(c) 6. Expression during the cell cycle (cdc15 arrest and release)(10)
(c) 6. Expression during the cell cycle (cdc15 arrest and release)(11)
(c) 6. Expression during the cell cycle (cdc15 arrest and release)(12)
(c) 6. Expression during the cell cycle (cdc15 arrest and release)(22)
(c) 6. Expression during the cell cycle (cdc15 arrest and release)(23)
(c) 7. Expression during the cell Cycle (cdc28)(10)
(c) 7. Expression during the cell Cycle (cdc28)(11)
(c) 7. Expression during the cell Cycle (cdc28)(12)
(c) 7. Expression during the cell Cycle (cdc28)(17)
(c) 8. Expression during the cell cycle (cell size selection and release)(12)
(c) 8. Expression during the cell cycle (cell size selection and release)(14)

SWI5 -*-| YLR049C

(c) 5. Expression during the cell cycle (alpha factor arrest and release)(10)
(c) 5. Expression during the cell cycle (alpha factor arrest and release)(11)
(c) 5. Expression during the cell cycle (alpha factor arrest and release)(12)
(c) 5. Expression during the cell cycle (alpha factor arrest and release)(13)
(c) 6. Expression during the cell cycle (cdc15 arrest and release)(1)
(c) 6. Expression during the cell cycle (cdc15 arrest and release)(2)
(c) 6. Expression during the cell cycle (cdc15 arrest and release)(10)
(c) 6. Expression during the cell cycle (cdc15 arrest and release)(11)
(c) 6. Expression during the cell cycle (cdc15 arrest and release)(12)
(c) 6. Expression during the cell cycle (cdc15 arrest and release)(22)
(c) 6. Expression during the cell cycle (cdc15 arrest and release)(23)
(c) 7. Expression during the cell Cycle (cdc28)(10)
(c) 7. Expression during the cell Cycle (cdc28)(11)
(c) 7. Expression during the cell Cycle (cdc28)(12)
(c) 7. Expression during the cell Cycle (cdc28)(17)
(c) 8. Expression during the cell cycle (cell size selection and release)(12)
(c) 8. Expression during the cell cycle (cell size selection and release)(14)

SWI5 -*-| YLR194C

(c) 5. Expression during the cell cycle (alpha factor arrest and release)(10)
(c) 5. Expression during the cell cycle (alpha factor arrest and release)(11)
(c) 5. Expression during the cell cycle (alpha factor arrest and release)(12)
(c) 5. Expression during the cell cycle (alpha factor arrest and release)(13)
(c) 6. Expression during the cell cycle (cdc15 arrest and release)(1)
(c) 6. Expression during the cell cycle (cdc15 arrest and release)(2)
(c) 6. Expression during the cell cycle (cdc15 arrest and release)(10)
(c) 6. Expression during the cell cycle (cdc15 arrest and release)(11)
(c) 6. Expression during the cell cycle (cdc15 arrest and release)(12)
(c) 6. Expression during the cell cycle (cdc15 arrest and release)(22)
(c) 6. Expression during the cell cycle (cdc15 arrest and release)(23)
(c) 7. Expression during the cell Cycle (cdc28)(10)
(c) 7. Expression during the cell Cycle (cdc28)(11)
(c) 7. Expression during the cell Cycle (cdc28)(12)
(c) 7. Expression during the cell Cycle (cdc28)(17)
(c) 8. Expression during the cell cycle (cell size selection and release)(12)
(c) 8. Expression during the cell cycle (cell size selection and release)(14)

SWI5 -*-| YNR067C

(c) 5. Expression during the cell cycle (alpha factor arrest and release)(10)
(c) 5. Expression during the cell cycle (alpha factor arrest and release)(11)
(c) 5. Expression during the cell cycle (alpha factor arrest and release)(12)
(c) 5. Expression during the cell cycle (alpha factor arrest and release)(13)
(c) 6. Expression during the cell cycle (cdc15 arrest and release)(1)
(c) 6. Expression during the cell cycle (cdc15 arrest and release)(2)
(c) 6. Expression during the cell cycle (cdc15 arrest and release)(10)
(c) 6. Expression during the cell cycle (cdc15 arrest and release)(11)
(c) 6. Expression during the cell cycle (cdc15 arrest and release)(12)
(c) 6. Expression during the cell cycle (cdc15 arrest and release)(22)
(c) 6. Expression during the cell cycle (cdc15 arrest and release)(23)
(c) 7. Expression during the cell Cycle (cdc28)(10)
(c) 7. Expression during the cell Cycle (cdc28)(11)
(c) 7. Expression during the cell Cycle (cdc28)(12)
(c) 7. Expression during the cell Cycle (cdc28)(17)
(c) 8. Expression during the cell cycle (cell size selection and release)(12)
(c) 8. Expression during the cell cycle (cell size selection and release)(14)

```
SWI5 -*-> YOR066W

    (c) 5. Expression during the cell cycle (alpha factor arrest and release)(10)
    (c) 5. Expression during the cell cycle (alpha factor arrest and release)(11)
    (c) 5. Expression during the cell cycle (alpha factor arrest and release)(12)
    (c) 5. Expression during the cell cycle (alpha factor arrest and release)(13)
    (c) 6. Expression during the cell cycle (cdc15 arrest and release)(1)
    (c) 6. Expression during the cell cycle (cdc15 arrest and release)(2)
    (c) 6. Expression during the cell cycle (cdc15 arrest and release)(10)
    (c) 6. Expression during the cell cycle (cdc15 arrest and release)(11)
    (c) 6. Expression during the cell cycle (cdc15 arrest and release)(12)
    (c) 6. Expression during the cell cycle (cdc15 arrest and release)(22)
    (c) 6. Expression during the cell cycle (cdc15 arrest and release)(23)
    (c) 7. Expression during the cell Cycle (cdc28)(10)
    (c) 7. Expression during the cell Cycle (cdc28)(11)
    (c) 7. Expression during the cell Cycle (cdc28)(12)
    (c) 7. Expression during the cell Cycle (cdc28)(17)
    (c) 8. Expression during the cell cycle (cell size selection and release)(12)
    (c) 8. Expression during the cell cycle (cell size selection and release)(14)

SWI5 -*-> YPL158C

    (c) 5. Expression during the cell cycle (alpha factor arrest and release)(10)
    (c) 5. Expression during the cell cycle (alpha factor arrest and release)(11)
    (c) 5. Expression during the cell cycle (alpha factor arrest and release)(12)
    (c) 5. Expression during the cell cycle (alpha factor arrest and release)(13)
    (c) 6. Expression during the cell cycle (cdc15 arrest and release)(1)
    (c) 6. Expression during the cell cycle (cdc15 arrest and release)(2)
    (c) 6. Expression during the cell cycle (cdc15 arrest and release)(10)
    (c) 6. Expression during the cell cycle (cdc15 arrest and release)(11)
    (c) 6. Expression during the cell cycle (cdc15 arrest and release)(12)
    (c) 6. Expression during the cell cycle (cdc15 arrest and release)(22)
    (c) 6. Expression during the cell cycle (cdc15 arrest and release)(23)
    (c) 7. Expression during the cell Cycle (cdc28)(10)
    (c) 7. Expression during the cell Cycle (cdc28)(11)
    (c) 7. Expression during the cell Cycle (cdc28)(12)
    (c) 7. Expression during the cell Cycle (cdc28)(17)
    (c) 8. Expression during the cell cycle (cell size selection and release)(12)
    (c) 8. Expression during the cell cycle (cell size selection and release)(14)

SWI5 -*-| YRF1-3

    (c) 5. Expression during the cell cycle (alpha factor arrest and release)(10)
    (c) 5. Expression during the cell cycle (alpha factor arrest and release)(11)
    (c) 5. Expression during the cell cycle (alpha factor arrest and release)(12)
    (c) 5. Expression during the cell cycle (alpha factor arrest and release)(13)
    (c) 6. Expression during the cell cycle (cdc15 arrest and release)(1)
    (c) 6. Expression during the cell cycle (cdc15 arrest and release)(2)
    (c) 6. Expression during the cell cycle (cdc15 arrest and release)(10)
    (c) 6. Expression during the cell cycle (cdc15 arrest and release)(11)
    (c) 6. Expression during the cell cycle (cdc15 arrest and release)(12)
    (c) 6. Expression during the cell cycle (cdc15 arrest and release)(22)
    (c) 6. Expression during the cell cycle (cdc15 arrest and release)(23)
    (c) 7. Expression during the cell Cycle (cdc28)(10)
    (c) 7. Expression during the cell Cycle (cdc28)(11)
    (c) 7. Expression during the cell Cycle (cdc28)(12)
    (c) 7. Expression during the cell Cycle (cdc28)(17)
    (c) 8. Expression during the cell cycle (cell size selection and release)(12)
    (c) 8. Expression during the cell cycle (cell size selection and release)(14)

SWI5 -*-| YRF1-5

    (c) 5. Expression during the cell cycle (alpha factor arrest and release)(10)
    (c) 5. Expression during the cell cycle (alpha factor arrest and release)(11)
    (c) 5. Expression during the cell cycle (alpha factor arrest and release)(12)
    (c) 5. Expression during the cell cycle (alpha factor arrest and release)(13)
    (c) 6. Expression during the cell cycle (cdc15 arrest and release)(1)
    (c) 6. Expression during the cell cycle (cdc15 arrest and release)(2)
    (c) 6. Expression during the cell cycle (cdc15 arrest and release)(10)
    (c) 6. Expression during the cell cycle (cdc15 arrest and release)(11)
    (c) 6. Expression during the cell cycle (cdc15 arrest and release)(12)
    (c) 6. Expression during the cell cycle (cdc15 arrest and release)(22)
    (c) 6. Expression during the cell cycle (cdc15 arrest and release)(23)
    (c) 7. Expression during the cell Cycle (cdc28)(10)
    (c) 7. Expression during the cell Cycle (cdc28)(11)
    (c) 7. Expression during the cell Cycle (cdc28)(12)
    (c) 7. Expression during the cell Cycle (cdc28)(17)
    (c) 8. Expression during the cell cycle (cell size selection and release)(12)
    (c) 8. Expression during the cell cycle (cell size selection and release)(14)

SWI5 -*-| YRF1-6

    (c) 5. Expression during the cell cycle (alpha factor arrest and release)(10)
    (c) 5. Expression during the cell cycle (alpha factor arrest and release)(11)
    (c) 5. Expression during the cell cycle (alpha factor arrest and release)(12)
    (c) 5. Expression during the cell cycle (alpha factor arrest and release)(13)
    (c) 6. Expression during the cell cycle (cdc15 arrest and release)(1)
    (c) 6. Expression during the cell cycle (cdc15 arrest and release)(2)
    (c) 6. Expression during the cell cycle (cdc15 arrest and release)(10)
    (c) 6. Expression during the cell cycle (cdc15 arrest and release)(11)
    (c) 6. Expression during the cell cycle (cdc15 arrest and release)(12)
    (c) 6. Expression during the cell cycle (cdc15 arrest and release)(22)
    (c) 6. Expression during the cell cycle (cdc15 arrest and release)(23)
    (c) 7. Expression during the cell Cycle (cdc28)(10)
    (c) 7. Expression during the cell Cycle (cdc28)(11)
    (c) 7. Expression during the cell Cycle (cdc28)(12)
    (c) 7. Expression during the cell Cycle (cdc28)(17)
    (c) 8. Expression during the cell cycle (cell size selection and release)(12)
    (c) 8. Expression during the cell cycle (cell size selection and release)(14)
```

SWI5 -*-> YOL030W

(c) 5. Expression during the cell cycle (alpha factor arrest and release)(10)
(c) 5. Expression during the cell cycle (alpha factor arrest and release)(11)
(c) 5. Expression during the cell cycle (alpha factor arrest and release)(12)
(c) 5. Expression during the cell cycle (alpha factor arrest and release)(13)
(c) 6. Expression during the cell cycle (cdc15 arrest and release)(1)
(c) 6. Expression during the cell cycle (cdc15 arrest and release)(2)
(c) 6. Expression during the cell cycle (cdc15 arrest and release)(10)
(c) 6. Expression during the cell cycle (cdc15 arrest and release)(11)
(c) 6. Expression during the cell cycle (cdc15 arrest and release)(12)
(c) 6. Expression during the cell cycle (cdc15 arrest and release)(22)
(c) 6. Expression during the cell cycle (cdc15 arrest and release)(23)
(c) 7. Expression during the cell Cycle (cdc28)(10)
(c) 7. Expression during the cell Cycle (cdc28)(11)
(c) 7. Expression during the cell Cycle (cdc28)(12)
(c) 7. Expression during the cell Cycle (cdc28)(17)
(c) 8. Expression during the cell cycle (cell size selection and release)(12)
(c) 8. Expression during the cell cycle (cell size selection and release)(14)

TEC1 -*-> GAD1

(c) 26. Fink: Expression in diploid high copy TEC1(1)
(c) 481. Expression in response to heat shock: 15,30,45,60,120 min(1)
(c) 481. Expression in response to heat shock: 15,30,45,60,120 min(2)
(c) 482. Expression in response to acid: 10,20,40,60,80,100 min(2)
(c) 483. Expression in response to alkali: 10,20,40,60,80,100 min(1)
(c) 491. Brown enviromental changes :Heat Shock 20 minutes hs-1(1)
(c) 492. Brown enviromental changes :Heat Shock 30 minutes hs-1(1)
(c) 493. Brown enviromental changes :Heat Shock 40 minutes hs-1(1)
(c) 494. Brown enviromental changes :Heat Shock 60 minutes hs-1(1)
(c) 508. Brown enviromental changes :heat shock 17 to 37, 20 minutes(1)
(c) 509. Brown enviromental changes :heat shock 21 to 37, 20 minutes(1)
(c) 510. Brown enviromental changes :heat shock 25 to 37, 20 minutes(1)
(c) 511. Brown enviromental changes :heat shock 29 to 37, 20 minutes(1)
(c) 512. Brown enviromental changes :heat shock 33 to 37, 20 minutes(1)
(c) 514. Brown enviromental changes :29C to 33C - 15 minutes(1)
(c) 518. Brown enviromental changes :29C +1M sorbitol to 33C + 1M sorbitol - 15 minutes(1)
(c) 561. Brown enviromental changes :1.5 mM diamide (40 min)(1)
(c) 567. Brown enviromental changes :1M sorbitol - 30 min(1)
(c) 568. Brown enviromental changes :1M sorbitol - 45 min (1)
(c) 623. Brown enviromental changes :DBY7286 37degree heat - 20 min(1)
(c) DES460 (wild type) + heat 20 min

XBP1 -*-| BUD4

(c) 5. Expression during the cell cycle (alpha factor arrest and release)(1)
(c) 5. Expression during the cell cycle (alpha factor arrest and release)(3)
(c) 5. Expression during the cell cycle (alpha factor arrest and release)(4)
(c) 5. Expression during the cell cycle (alpha factor arrest and release)(5)
(c) 6. Expression during the cell cycle (cdc15 arrest and release)(3)
(c) 6. Expression during the cell cycle (cdc15 arrest and release)(11)
(c) 8. Expression during the cell cycle (cell size selection and release)(2)
(c) 8. Expression during the cell cycle (cell size selection and release)(4)
(c) 8. Expression during the cell cycle (cell size selection and release)(5)
(c) 49. Expression in response to 50 nM alpha-factor: 0,15,30,45,60,90,120 min(4)
(c) 49. Expression in response to 50 nM alpha-factor: 0,15,30,45,60,90,120 min(6)
(c) 387. Rosetta 2000: Expression in cells with ERG11 under tet promoter(1)
(c) 430. Expression in strain PM38 (wild type), glucose versus ethanol: strain was shifted from medium containing dextrose as carbon source, ammonium sulfate as nirogen source, supplemented with leucine and uracil to same medium for 30 min, compared to a shift to a medium with synthetic ethanol instead of glucose for 30 min(1)
(c) 590. Brown enviromental changes :Nitrogen Depletion 2 d(1)
(c) 592. Brown enviromental changes :Nitrogen Depletion 5 d(1)
(c) 607. Brown enviromental changes :YPD 1 d ypd-2(1)
(c) 617. Brown enviromental changes :YPD stationary phase 3 d ypd-1(1)
(c) DES460 + 0.02% MMS - 60 min

YAP1 -*-> AAD6

(c) 451. Expression in response to BCNU (200 micromolar) for 60 min(1)
(c) 523. Brown enviromental changes :constant 0.32 mM H2O2 (10 min) redo(1)
(c) 524. Brown enviromental changes :constant 0.32 mM H2O2 (20 min) redo(1)
(c) 525. Brown enviromental changes :constant 0.32 mM H2O2 (30 min) redo(1)
(c) 527. Brown enviromental changes :constant 0.32 mM H2O2 (50 min) redo(1)
(c) 528. Brown enviromental changes :constant 0.32 mM H2O2 (60 min) redo(1)
(c) 529. Brown enviromental changes :constant 0.32 mM H2O2 (80 min) redo(1)
(c) 530. Brown enviromental changes :constant 0.32 mM H2O2 (100 min) redo(1)
(c) 531. Brown enviromental changes :constant 0.32 mM H2O2 (120 min) redo(1)
(c) 532. Brown enviromental changes :constant 0.32 mM H2O2 (160 min) redo(1)
(c) 534. Brown enviromental changes :1 mM Menadione (20 min) redo(1)
(c) 535. Brown enviromental changes :1 mM Menadione (30 min) redo(1)
(c) 536. Brown enviromental changes :1mM Menadione (40 min) redo(1)
(c) 537. Brown enviromental changes :1 mM Menadione (50 min)redo(1)
(c) 538. Brown enviromental changes :1 mM Menadione (80 min) redo(1)
(c) 539. Brown enviromental changes :1 mM Menadione (105 min) redo(1)
(c) 540. Brown enviromental changes :1 mM Menadione (120 min)redo(1)
(c) 541. Brown enviromental changes :1 mM Menadione (160 min) redo(1)
(c) 545. Brown enviromental changes :2.5mM DTT 045 min dtt-1(1)
(c) 557. Brown enviromental changes :1.5 mM diamide (5 min)(1)
(c) 558. Brown enviromental changes :1.5 mM diamide (10 min)(1)
(c) 559. Brown enviromental changes :1.5 mM diamide (20 min)(1)
(c) 560. Brown enviromental changes :1.5 mM diamide (30 min)(1)
(c) 561. Brown enviromental changes :1.5 mM diamide (40 min)(1)
(c) 562. Brown enviromental changes :1.5 mM diamide (50 min)(1)
(c) 563. Brown enviromental changes :1.5 mM diamide (60 min)(1)
(c) 564. Brown enviromental changes :1.5 mM diamide (90 min)(1)
(c) 568. Brown enviromental changes :1M sorbitol - 45 min (1)
(c) 628. Brown enviromental changes :DBY7286 + 0.3 mM H2O2 (20 min)(1)
(c) DES460 + 0.02% MMS - 120 min

YAP1 -*-> ECM4

(c) 451. Expression in response to BCNU (200 micromolar) for 60 min(1)
(c) 523. Brown enviromental changes :constant 0.32 mM H2O2 (10 min) redo(1)

(c) 524. Brown enviromental changes :constant 0.32 mM H2O2 (20 min) redo(1)
(c) 525. Brown enviromental changes :constant 0.32 mM H2O2 (30 min) redo(1)
(c) 527. Brown enviromental changes :constant 0.32 mM H2O2 (50 min) redo(1)
(c) 528. Brown enviromental changes :constant 0.32 mM H2O2 (60 min) redo(1)
(c) 529. Brown enviromental changes :constant 0.32 mM H2O2 (80 min) redo(1)
(c) 530. Brown enviromental changes :constant 0.32 mM H2O2 (100 min) redo(1)
(c) 531. Brown enviromental changes :constant 0.32 mM H2O2 (120 min) redo(1)
(c) 532. Brown enviromental changes :constant 0.32 mM H2O2 (160 min) redo(1)
(c) 534. Brown enviromental changes :1 mM Menadione (20 min) redo(1)
(c) 535. Brown enviromental changes :1 mM Menadione (30 min) redo(1)
(c) 536. Brown enviromental changes :1mM Menadione (40 min) redo(1)
(c) 537. Brown enviromental changes :1 mM Menadione (50 min)redo(1)
(c) 538. Brown enviromental changes :1 mM Menadione (80 min) redo(1)
(c) 539. Brown enviromental changes :1 mM Menadione (105 min) redo(1)
(c) 540. Brown enviromental changes :1 mM Menadione (120 min)redo(1)
(c) 541. Brown enviromental changes :1 mM Menadione (160 min) redo(1)
(c) 545. Brown enviromental changes :2.5mM DTT 045 min dtt-1(1)
(c) 557. Brown enviromental changes :1.5 mM diamide (5 min)(1)
(c) 558. Brown enviromental changes :1.5 mM diamide (10 min)(1)
(c) 559. Brown enviromental changes :1.5 mM diamide (20 min)(1)
(c) 560. Brown enviromental changes :1.5 mM diamide (30 min)(1)
(c) 561. Brown enviromental changes :1.5 mM diamide (40 min)(1)
(c) 562. Brown enviromental changes :1.5 mM diamide (50 min)(1)
(c) 563. Brown enviromental changes :1.5 mM diamide (60 min)(1)
(c) 564. Brown enviromental changes :1.5 mM diamide (90 min)(1)
(c) 568. Brown enviromental changes :1M sorbitol - 45 min (1)
(c) 628. Brown enviromental changes :DBY7286 + 0.3 mM H2O2 (20 min)(1)
(c) DES460 + 0.02% MMS - 120 min

YAP1 -*-> LAP4

(c) 451. Expression in response to BCNU (200 micromolar) for 60 min(1)
(c) 523. Brown enviromental changes :constant 0.32 mM H2O2 (10 min) redo(1)
(c) 524. Brown enviromental changes :constant 0.32 mM H2O2 (20 min) redo(1)
(c) 525. Brown enviromental changes :constant 0.32 mM H2O2 (30 min) redo(1)
(c) 527. Brown enviromental changes :constant 0.32 mM H2O2 (50 min) redo(1)
(c) 528. Brown enviromental changes :constant 0.32 mM H2O2 (60 min) redo(1)
(c) 529. Brown enviromental changes :constant 0.32 mM H2O2 (80 min) redo(1)
(c) 530. Brown enviromental changes :constant 0.32 mM H2O2 (100 min) redo(1)
(c) 531. Brown enviromental changes :constant 0.32 mM H2O2 (120 min) redo(1)
(c) 532. Brown enviromental changes :constant 0.32 mM H2O2 (160 min) redo(1)
(c) 534. Brown enviromental changes :1 mM Menadione (20 min) redo(1)
(c) 535. Brown enviromental changes :1 mM Menadione (30 min) redo(1)
(c) 536. Brown enviromental changes :1mM Menadione (40 min) redo(1)
(c) 537. Brown enviromental changes :1 mM Menadione (50 min)redo(1)
(c) 538. Brown enviromental changes :1 mM Menadione (80 min) redo(1)
(c) 539. Brown enviromental changes :1 mM Menadione (105 min) redo(1)
(c) 540. Brown enviromental changes :1 mM Menadione (120 min)redo(1)
(c) 541. Brown enviromental changes :1 mM Menadione (160 min) redo(1)
(c) 545. Brown enviromental changes :2.5mM DTT 045 min dtt-1(1)
(c) 557. Brown enviromental changes :1.5 mM diamide (5 min)(1)
(c) 558. Brown enviromental changes :1.5 mM diamide (10 min)(1)
(c) 559. Brown enviromental changes :1.5 mM diamide (20 min)(1)
(c) 560. Brown enviromental changes :1.5 mM diamide (30 min)(1)
(c) 561. Brown enviromental changes :1.5 mM diamide (40 min)(1)
(c) 562. Brown enviromental changes :1.5 mM diamide (50 min)(1)
(c) 563. Brown enviromental changes :1.5 mM diamide (60 min)(1)
(c) 564. Brown enviromental changes :1.5 mM diamide (90 min)(1)
(c) 568. Brown enviromental changes :1M sorbitol - 45 min (1)
(c) 628. Brown enviromental changes :DBY7286 + 0.3 mM H2O2 (20 min)(1)
(c) DES460 + 0.02% MMS - 120 min

YAP1 -*-> YDL124W

(c) 451. Expression in response to BCNU (200 micromolar) for 60 min(1)
(c) 523. Brown enviromental changes :constant 0.32 mM H2O2 (10 min) redo(1)
(c) 524. Brown enviromental changes :constant 0.32 mM H2O2 (20 min) redo(1)
(c) 525. Brown enviromental changes :constant 0.32 mM H2O2 (30 min) redo(1)
(c) 527. Brown enviromental changes :constant 0.32 mM H2O2 (50 min) redo(1)
(c) 528. Brown enviromental changes :constant 0.32 mM H2O2 (60 min) redo(1)
(c) 529. Brown enviromental changes :constant 0.32 mM H2O2 (80 min) redo(1)
(c) 530. Brown enviromental changes :constant 0.32 mM H2O2 (100 min) redo(1)
(c) 531. Brown enviromental changes :constant 0.32 mM H2O2 (120 min) redo(1)
(c) 532. Brown enviromental changes :constant 0.32 mM H2O2 (160 min) redo(1)
(c) 534. Brown enviromental changes :1 mM Menadione (20 min) redo(1)
(c) 535. Brown enviromental changes :1 mM Menadione (30 min) redo(1)
(c) 536. Brown enviromental changes :1mM Menadione (40 min) redo(1)
(c) 537. Brown enviromental changes :1 mM Menadione (50 min)redo(1)
(c) 538. Brown enviromental changes :1 mM Menadione (80 min) redo(1)
(c) 539. Brown enviromental changes :1 mM Menadione (105 min) redo(1)
(c) 540. Brown enviromental changes :1 mM Menadione (120 min)redo(1)
(c) 541. Brown enviromental changes :1 mM Menadione (160 min) redo(1)
(c) 545. Brown enviromental changes :2.5mM DTT 045 min dtt-1(1)
(c) 557. Brown enviromental changes :1.5 mM diamide (5 min)(1)
(c) 558. Brown enviromental changes :1.5 mM diamide (10 min)(1)
(c) 559. Brown enviromental changes :1.5 mM diamide (20 min)(1)
(c) 560. Brown enviromental changes :1.5 mM diamide (30 min)(1)
(c) 561. Brown enviromental changes :1.5 mM diamide (40 min)(1)
(c) 562. Brown enviromental changes :1.5 mM diamide (50 min)(1)
(c) 563. Brown enviromental changes :1.5 mM diamide (60 min)(1)
(c) 564. Brown enviromental changes :1.5 mM diamide (90 min)(1)
(c) 568. Brown enviromental changes :1M sorbitol - 45 min (1)
(c) 628. Brown enviromental changes :DBY7286 + 0.3 mM H2O2 (20 min)(1)
(c) DES460 + 0.02% MMS - 120 min
