# Supplementary material for: Unraveling condition specific gene transcriptional regulatory networks in Saccharomyces cerevisiae
Source: BMC Bioinformatics. 2006 Mar 21;7:165. doi: 10.1186/1471-2105-7-165 (PMC1488875; doi:10.1186/1471-2105-7-165)
Supplement: Additional File 10 — Exploring the parameter space. Overlap between our condition specific predicted networks and condition specific ChIP-on-chip data. [file 1471-2105-7-165-S10.pdf]

LINK **Vary tg**

tg=3.0	tc=1.5	|R|>0.5	overlap with ChIP-on-chip data (P <= 0.005): 217/940=0.2309
tg=4.0	tc=1.5	|R|>0.5	overlap with ChIP-on-chip data (P <= 0.005): 97/244=0.3975 (our choice of tg)
tg=5.0	tc=1.5	|R|>0.5	overlap with ChIP-on-chip data (P <= 0.005): 34/82=0.4146

At tg=5.0 the overlap with ChIP-on-chip is slightly bigger than at tg=4.0.
However, the number of predictions decreases by factor of three.
Our choice of tg=4.0, which is more restrictive than the one used by Ihmels et al (tg=3.0),
leads to a better overlap with currently available ChIP-on-chip data

STAR **Vary tg**

tg=3.0	tc=1.5	|R|>0.7	overlap with ChIP-on-chip data (P <= 0.005): 234/539=0.4341
tg=4.0	tc=1.5	|R|>0.7	overlap with ChIP-on-chip data (P <= 0.005): 190/419=0.4535 (our choice of tg)
tg=5.0	tc=1.5	|R|>0.7	overlap with ChIP-on-chip data (P <= 0.005): 180/413=0.4358

LINK **Vary tc**

tg=4.0	tc=1.0	|R|>0.5	overlap with ChIP-on-chip data (P <= 0.005): 62/159=0.3899
tg=4.0	tc=1.5	|R|>0.5	overlap with ChIP-on-chip data (P <= 0.005): 97/244=0.3975 (our choice of tc)
tg=4.0	tc=2.0	|R|>0.5	overlap with ChIP-on-chip data (P <= 0.005): 118/333=0.3544

STAR **Vary tc**

tg=4.0	tc=1.0	|R|>0.7	overlap with ChIP-on-chip data (P <= 0.005): 154/345=0.4464
tg=4.0	tc=1.5	|R|>0.7	overlap with ChIP-on-chip data (P <= 0.005): 190/419=0.4535 (our choice of tc)
tg=4.0	tc=2.0	|R|>0.7	overlap with ChIP-on-chip data (P <= 0.005): 190/464=0.4095

LINK **Vary R**

tg=4.0	tc=1.5	|R|>0.3	overlap with ChIP-on-chip data (P <= 0.005): 161/434=0.3710
tg=4.0	tc=1.5	|R|>0.5	overlap with ChIP-on-chip data (P <= 0.005): 97/244=0.3975 (our choice of R)
tg=4.0	tc=1.5	|R|>0.7	overlap with ChIP-on-chip data (P <= 0.005): 32/84=0.3810

STAR **Vary R**

tg=4.0	tc=1.5	|R|>0.5	overlap with ChIP-on-chip data (P <= 0.005): 261/681=0.3833
tg=4.0	tc=1.5	|R|>0.7	overlap with ChIP-on-chip data (P <= 0.005): 190/419=0.4535 (our choice of R)
tg=4.0	tc=1.5	|R|>0.9	overlap with ChIP-on-chip data (P <= 0.005): 10/25=0.4

For the restrictive values we choose for tc and tg, the cutoff R has a noticeable (monotonic) effect
on the number of predictions (coverage), but a smaller effect on the overlap with ChIP-on-chip data.
